# Gene Expression Profiling Reveals New Aspects of *PIK3CA* Mutation in ERalpha-Positive Breast Cancer: Major Implication of the Wnt Signaling Pathway

**DOI:** 10.1371/journal.pone.0015647

**Published:** 2010-12-30

**Authors:** Magdalena Cizkova, Géraldine Cizeron-Clairac, Sophie Vacher, Aurélie Susini, Catherine Andrieu, Rosette Lidereau, Ivan Bièche

**Affiliations:** 1 Laboratoire d'Oncogénétique, Institut National de la Santé et de la Recherche, U745, Institut Curie/Hôpital René Huguenin, St-Cloud, France; 2 Laboratory of Experimental Medicine, Department of Paediatrics, Faculty of Medicine and Dentistry, Palacky University and University Hospital Olomouc, Olomouc, Czech Republic; East Carolina University, United States of America

## Abstract

**Background:**

The PI3K/AKT pathway plays a pivotal role in breast cancer development and maintenance. *PIK3CA*, encoding the PI3K catalytic subunit, is the oncogene exhibiting a high frequency of gain-of-function mutations leading to PI3K/AKT pathway activation in breast cancer. *PIK3CA* mutations have been observed in 30% to 40% of ERα-positive breast tumors. However the physiopathological role of *PIK3CA* mutations in breast tumorigenesis remains largely unclear.

**Methodology/Principal Findings:**

To identify relevant downstream target genes and signaling activated by aberrant PI3K/AKT pathway in breast tumors, we first analyzed gene expression with a pangenomic oligonucleotide microarray in a series of 43 ERα-positive tumors with and without *PIK3CA* mutations. Genes of interest were then investigated in 249 ERα-positive breast tumors by real-time quantitative RT-PCR. A robust collection of 19 genes was found to be differently expressed in *PIK3CA-*mutated tumors. *PIK3CA* mutations were associated with over-expression of several genes involved in the Wnt signaling pathway (*WNT5A*, *TCF7L2*, *MSX2*, *TNFRSF11B*), regulation of gene transcription (*SEC14L2*, *MSX2*, *TFAP2B*, *NRIP3*) and metal ion binding (*CYP4Z1*, *CYP4Z2P*, *SLC40A1*, *LTF*, *LIMCH1*).

**Conclusion/Significance:**

This new gene set should help to understand the behavior of *PIK3CA*-mutated cancers and detailed knowledge of Wnt signaling activation could lead to novel therapeutic strategies.

## Introduction

Deregulation of the phosphatidylinositol 3-kinase (PI3K) signaling pathway is frequent in human cancers. Activation of PI3K, which catalyzes inositol lipid phosphorylation to produce phosphatidylinositol-3,4,5-trisphosphate, is one of the most important downstream molecular events following tyrosine kinase receptor activation. Phosphatidylinositol-3,4,5-trisphosphate activates the serine/threonine kinase AKT, which in turn regulates several signaling pathways controlling cell survival, apoptosis, proliferation, motility, and adhesion [1]. PI3K is a heterodimeric enzyme composed of a p110α catalytic subunit encoded by the *PIK3CA* gene, and a p85 regulatory subunit encoded by the *PIK3R1* gene [2].

Gain-of-function mutations in *PIK3CA* have recently been found in several malignancies, including breast cancer [1], [3], [4]. *PIK3CA* is frequently mutated at “hotspots” in exons 9 and 20, corresponding to the helical (E542K and E545K) and kinase (H1047R) domains, respectively. P110α carrying a hotspot mutation has oncogenic activity, transforming primary fibroblasts in culture, inducing anchorage-independent cell growth, and causing tumors in animals [5], [6].

After the *TP53* suppressor gene, the *PIK3CA* oncogene is the most frequently mutated gene in human breast cancers (up to 40% of breast tumors) [7], [8]. Activating somatic mutations of other oncogenes (*EGFR, KRAS, HRAS, NRAF, BRAF* and *AKT1*) involved in downstream molecular events following tyrosine kinase receptor activation are frequent in several malignancies but rare in breast cancer. Several studies suggest that *PIK3CA* mutations are more frequent in estrogen receptor alpha (ERα)-positive breast tumors (30–40%) than in ERα-negative breast tumors (10–20%) [7].

The pathological role of these gain-of-function *PIK3CA* mutations in breast tumors, and particularly in ERα-positive breast tumors, is largely unknown. Better knowledge of *PIK3CA* mutation impact requires the identification of downstream target genes and signaling pathways activated by aberrant PI3K/AKT signaling. Here, we compared gene expression in *PIK3CA*-mutated and *PIK3CA* wild-type ERα-positive breast tumors, using a genome-wide microarray and subsequently real-time quantitative reverse transcriptase-polymerase chain reaction (RT-PCR).

## Materials and Methods

### Patients and Samples

We analyzed samples of 292 primary unilateral non metastatic ERα-positive postmenopausal breast tumors excised from women at René Huguenin Hospital (Saint-Cloud, France) from 1978 to 2008. Other characteristics of the patients are listed in Table S1. Each patient gave written informed consent and this study was approved by the Local Ethical Committee (Breast Group of René Huguenin Hospital). Immediately after surgery the tumor samples were stored in liquid nitrogen until RNA extraction. The samples analyzed contained more than 70% of tumor cells. ERα status was determined at the protein level by using biochemical methods (Dextran-coated charcoal method until 1988 and enzyme immunoassay thereafter) and was confirmed at mRNA level by real-time RT-PCR. Forty-three samples were used as a microarray and RT-PCR screening set to identify differentially expressed genes. These genes were then validated in the remaining 249 ERα-positive tumors by means of RT-PCR. Control samples consisted of eight specimens of normal breast tissue collected from women undergoing cosmetic breast surgery or adjacent normal breast tissue from breast cancer patients.

### RNA extraction

Total RNA was extracted from breast tissue by using the acid-phenol guanidium method, and its quality was determined by agarose gel electrophoresis and ethidium bromide staining. The 18S and 28S RNA bands were visualized under ultraviolet light.

### *PIK3CA* mutation screening

*PIK3CA* mutation screening was performed on cDNA fragments obtained by RT-PCR amplification of exons 9 and 20 and their flanking exons. Details of the primers and PCR conditions are available on request. The amplified products were sequenced with the BigDye Terminator kit on an ABI Prism 3130 automatic DNA sequencer (Applied Biosystems, Courtabœuf, France). Sequences thus obtained were compared with the corresponding cDNA reference sequence (NM_006218).

### Microarray analysis

Microarray experiments used Human Genome U133 Plus 2.0 arrays from Affymetrix, containing 54675 probe sets. Gene chips were hybridized and scanned using standard Affymetrix protocols. Expression data were obtained as CEL files. BRB ArrayTools (version 3.6.0 available on http://linus.nci.nih.gov/BRB-ArrayTools.html) were used to import CEL files with Robust Method Average (RMA) normalization, and to analyze gene expression. A class comparison based on a univariate *t* test applied to log-normalized data was used to identify genes differentially expressed in breast tumors with and without *PIK3CA* mutations. Supervised class prediction analysis was implemented with the Prediction Analysis for Microarrays (PAM) algorithm to identify genes required for optimal prediction [9].

The Database for Annotation, Visualization and Integrated Discovery (DAVID, available on http://david.abcc.ncifcrf.gov/) was used to interpret the lists of differentially expressed probes and to identify statistically overrepresented biological function categories of Gene Ontology (GO) and biological pathways, as defined in the Kyoto Encyclopedia of Genes and Genomes (KEGG).

In compliance with the Minimun Information About a Microarray Experiment (MIAME) recommendations, raw data were deposited in the Gene Expression Omnibus (GEO) database (http://www.ncbi.nlm.nih.gov/geo/) under series accession number GSE22035.

### Real-time quantitative RT-PCR

RT-PCR was applied to the selected genes, as well as *ER*α (NM_000125), *MKI67* (NM_002417), and *TBP* (NM_003194; endogenous mRNA control). Primers and PCR conditions are available on request, and the RT-PCR protocol using the SYBR Green Master Mix kit on the ABI Prism 7900 Sequence Detection System (Perkin-Elmer Applied Biosystems, Foster City, CA, USA) is described in detail elsewhere [10]. The relative mRNA expression level of each gene, expressed as the N-fold difference in target gene expression relative to the *TBP* gene, and termed “N*target*”, was calculated as N*target* = 2^ΔCt^ *_sample_*. The value of the cycle threshold (ΔCt) of a given sample was determined by subtracting the average Ct value of the target gene from the average Ct value of the *TBP* gene. The N*target* values of the samples were subsequently normalized such that the median N*target* value of the normal breast samples was 1. The relative expression of each gene was characterized by the median and range, and the differences in gene expression between tumors with and without *PIK3CA* mutations were analysed for significance with the non parametric Mann-Whitney *U* test.

### Clustering analysis

Hierarchical clustering analyses of gene expression and samples were performed using BRB ArrayTools. Classification performance was calculated as overall accuracy, defined as the proportion of correctly classified tumors in each cluster, using Matthews' correlation coefficient (MCC) [11]. This parameter was used to discriminate identical accuracies. The chi-square test was used to determine the statistical significance of the clustering.

## Results

### Analysis of differentially expressed genes in 43 ERα-positive tumors

#### Overview of transcriptome changes in PIK3CA-mutated tumors

To identify *PIK3CA* mutation-related genes, microarray analysis (Affymetrix U133 Plus 2.0 arrays) was first applied to 43 ERα-positive breast tumors, of which 14 were *PIK3CA*-mutated and 29 were wild-type (Table S1). We found that 6124 probes were differentially expressed between breast tumors with and without *PIK3CA* mutations, with *P* values <0.05. Of these, 2538 probes (1630 unique genes) were up-regulated (Table S2) and 3586 (2672 unique genes) were down-regulated (Table S3). Only 216 up-regulated probes (153 unique genes) and 28 down-regulated probes (18 unique genes) showed at least a 2-fold change (FC).

#### Gene ontology analysis of differentially expressed genes

To identify families of genes that might have significant roles related to specific biological or molecular processes, we used the DAVID database to annotate the 6124 probes and categorize them by function. As shown in Table 1, these genes were mainly involved in the regulation of transcription, cell cycling, proliferation, death, adhesion and cytoskeleton organization, and also ion binding and transport, and ATP and RNA binding activity.

**Table 1:** Selected categories significantly over-represented in *PIK3CA*-mutated breast tumors.

	Up- and down-regulated genes		Up-regulated genes		Down-regulated genes	
Gene Category	Number of genes	*P* value	Number of genes	*P* value	Number of genes	*P* value
GENE ONTOLOGY						
• Biological Process						
Regulation of transcription	581 (14%)	0.0100	282 (17%)	<0.0001	−	−
Regulation of cell cycle and proliferation	203 (4.8%)	0.0002	94 (5.8%)	0.0004	115 (4.3%)	ns
Regulation of cell death	198 (4.7%)	0.0052	84 (5.2%)	0.0360	120 (4.5%)	0.0430
Cell adhesion	171 (4.1%)	0.0073	81 (5.0%)	0.0027	94 (3.5%)	ns
Ion transport	169 (4.0%)	ns	−	−	130 (4.9%)	0.0003
Cytoskeleton organization	116 (2.8%)	0.0014	58 (3.6%)	0.0004	63 (2.4%)	ns
• Molecular Function						
Ion binding	936 (22%)	0.0040	417 (26%)	0.0007	543 (20%)	ns
Metal ion binding	920 (15%)	0.0019	411 (25%)	0.0003	533 (20%)	ns
Zinc ion binding	518 (22%)	0.0140	268 (16%)	<0.0001	269 (10%)	ns
ATP binding	339 (8.1%)	0.0130	130 (8.0%)	ns	218 (8.2%)	0.0048
RNA binding	182 (4.4%)	0.0009	87 (5.3%)	0.0008	108 (4.0%)	0.0260
Acetylation	−	−	−	−	378 (14%)	0.0004
KEGG PATHWAY						
Pathways in cancer	100 (2.4%)	<0.0001	55 (3.4%)	<0.0001	47 (1.1%)	ns
MAPK signaling pathway	76 (1.8%)	0.0011	32 (2.0%)	0.0200	47 (1.1%)	0.0190
Calcium signaling pathway	50 (1.2%)	0.0093	10 (0.6%)	ns	44 (1.0%)	<0.0001
Jak-STAT signaling pathway	43 (1.0%)	0.0210	17 (1.0%)	ns	28 (0.7%)	0.0470
Wnt signaling pathway	41 (1.0%)	0.0370	24 (1.5%)	0.0015	17 (0.4%)	ns
Apoptosis	27 (0.6%)	0.0130	12 (0.7%)	ns	15 (0.4%)	ns

ns: not significant.

The biological processes, molecular functions and physiological pathways of genes were obtained from the DAVID database using GOTERM_BP_FAT, GOTERM_MF_FAT and KEGG PATHWAY, respectively. The two first tools (Gene Ontology) annotated 4202 genes (1630 up- and 2672 down-regulated genes) while KEGG annotated 960 genes (385 up- and 601 down-regulated genes). The gene enrichment of a given class was measured by determining the number of genes belonging to the class in the list of significantly altered genes, weighed against the total human genome, and was tested using Fisher exact probability test. Not all significant categories are included here in order to reduce redundancy. A given gene can belong to several processes.

The 2672 down-regulated genes were mainly associated with ATP binding, acetylation and ion transport (Table 1). Among the down-regulated genes with FC≥2, no significantly overrepresented GO categories appeared.

**Figure 1:**
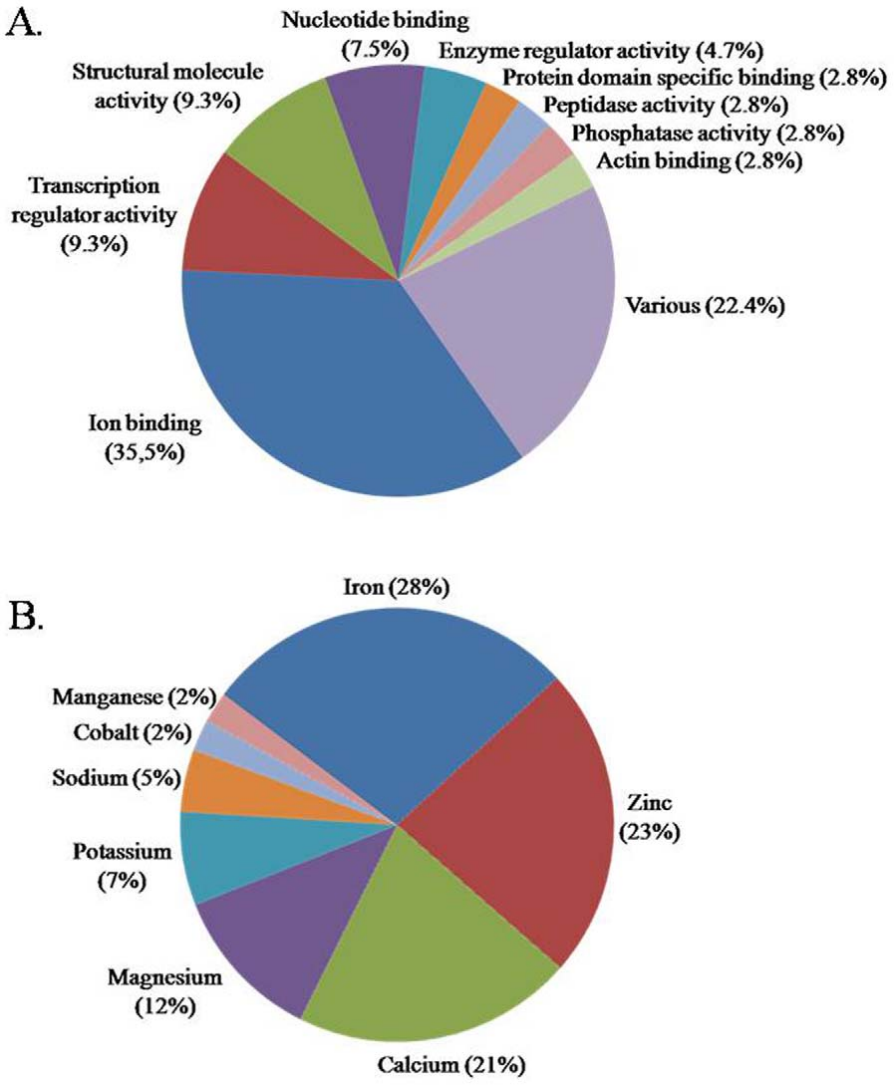
Molecular function classifications of genes up-regulated with a FC≥2 in the *PIK3CA*-mutated tumors. Molecular functions were attributed to 107 of the 153 genes using GOTERM_MF_FAT from the DAVID database. Categories with at least three genes are represented in A. Subclassification of the 36 genes belonging to the metal ion-binding category is shown in B. All categories were represented and several genes were common to more than one category. Genes belonging to the metal ion-binding and transcription activity categories are listed in Table 2.

Most of the 1630 up-regulated genes were involved in transcriptional regulation (17.3%) (biological process) and ion binding (25.6%) (molecular function) (Table 1). The latter included the metal ion-binding and zinc ion-binding categories (Table 1). As shown in Figure 1A, the 216 probes most strongly up-regulated in *PIK3CA*-mutated tumors (153 unique genes) belonged mainly to the ion-binding category (35.5%) but also to categories of structural molecule activity (including structural cytoskeleton constituents) (9.3%), transcription regulatory activity (9.3%) and nucleotide binding (including ATP and GTP binding) (7.5%).

**Table 2:** List of genes belonging to the metal ion-binding and transcription regulation categories.

Probe set	FC	*P* value	Gene symbol	Probe set	FC	*P* value	Gene symbol
METAL ION BINDING				221584_s_at	2.11	0.0023	*KCNMA1*
Iron ion binding				1564241_at	2.07	0.0257	*ATP1A4*
202018_s_at*	10.52	0.0005	*LTF*	230364_at	2.00	0.0217	*CHPT1*
237395_at*	7.76	0.0035	*CYP4Z1*	Sodium ion binding			
227702_at*	5.57	0.0032	*CYP4X1*	203908_at*	4.81	0.0005	*SLC4A4*
239723_at*	4.42	0.0005	*SLC40A1*	201242_s_at	2.76	0.0001	*ATP1B1*
210096_at*	4.12	0.0011	*CYP4B1*	201243_s_at	2.71	0.0002	*ATP1B1*
1553434_at*	3.80	0.0009	*CYP4Z2P*	210738_s_at*	2.13	0.0023	*SLC4A4*
225871_at	2.34	0.0188	*STEAP2*	211494_s_at*	2.13	0.0025	*SLC4A4*
1555497_a_at*	2.34	0.0061	*CYP4B1*	Potassium ion binding			
233123_at*	2.29	0.0139	*SLC40A1*	244623_at	2.30	0.0152	*KCNQ5*
223044_at*	2.26	0.0066	*SLC40A1*	221584_s_at	2.11	0.0023	*KCNMA1*
205542_at	2.17	0.0266	*STEAP1*	1564241_at	2.07	0.0257	*ATP1A4*
219232_s_at	2.15	0.0006	*EGLN3*	Cobalt ion binding			
222453_at	2.14	0.0119	*CYBRD1*	205513_at	2.87	0.0009	*TCN1*
204446_s_at	2.19	0.0003	*ALOX5*	Manganese ion binding			
224996_at	2.10	0.0135	*ASPH*	230364_at	2.00	0.0217	*CHPT1*
Zinc ion binding							
202888_s_at*	3.52	0.0008	*ANPEP*	TRANSCRIPTION REGULATION			
212774_at	2.97	0.0320	*ZNF238*	214451_at*	6.68	0.0020	*TFAP2B*
212325_at*	2.96	0.0002	*LIMCH1*	1553394_a_at*	4.34	0.0035	*TFAP2B*
225728-at	2.72	0.0141	*SORBS2*	223864_at	4.25	0.0399	*ANKRD30A*
207981_s_at	2.69	0.0213	*ESRRG*	230316_at*	3.05	0.0006	*SEC14L2*
212328_at*	2.69	0.0001	*LIMCH1*	204541_at*	3.03	0.0004	*SEC14L2*
204288_s_at	2.69	0.0073	*SORBS2*	209292_at*	3.03	0.0002	*ID4*
212327_at*	2.49	0.0008	*LIMCH1*	212774_at	2.97	0.0320	*ZNF238*
241459_at*	2.35	0.0003	*LIMCH1*	209291_at*	2.96	0.0001	*ID4*
227811_at	2.20	0.0051	*FGD3*	207981_s_at	2.69	0.0213	*ESRRG*
211965_at	2.18	0.0002	*ZFP36L1*	226847_at	2.61	0.0020	*FST*
215073_s_at*	2.08	0.0063	*NR2F2*	243030_at	2.49	0.0006	*MAP3K1*
231929_at	2.07	0.0039	*IKZF2*	226992_at	2.23	0.0064	*NOSTRIN*
214761_at	2.05	0.0016	*ZNF423*	212762_s_at*	2.18	0.0000	*TCF7L2*
Calcium ion binding				210319_x_at*	2.17	0.0011	*MSX2*
219197_s_at	3.08	0.0173	*SCUBE2*	216511_s_at*	2.16	0.0000	*TCF7L2*
204455_at	2.70	0.0065	*DST*	224975_at	2.13	0.0003	*NFIA*
229030_at	2.42	0.0370	*CAPN8*	240024_at*	2.12	0.0016	*SEC14L2*
209369_at	2.42	0.0174	*ANXA3*	209706_at	2.12	0.0292	*NKX361*
203887_s_at	2.20	0.0006	*THBD*	221666_s_at	2.09	0.0050	*PYCARD*
204446_s_at	2.19	0.0003	*ALOX5*	215073_s_at*	2.08	0.0063	*NR2F2*
224996_at	2.10	0.0135	*ASPH*	216035_x_at*	2.08	0.0000	*TCF7L2*
221584_s_at	2.11	0.0023	*KCNMA1*	231929_at	2.07	0.0039	*IKZF2*
1564241_at	2.07	0.0257	*ATP1A4*	214761_at	2.05	0.0016	*ZNF423*
Magnesium ion binding				220625_s_at	2.02	0.0286	*ELF5*
227556_at	2.99	0.0007	*NME7*	226806_s_at	2.02	0.0006	*NFIA*
243030_at	2.49	0.0006	*MAP3K1*				

These genes are ranked according to the fold change (FC) in tumors with *PIK3CA* mutations relative to non mutated tumors. Several genes were common to more than one category. The genes marked with an asterisk were selected for RT-PCR validation.

In the ion-binding category, the genes corresponded to genes encoding metal ion-binding proteins in 95% of cases: 28% encoding iron ion-binding and 23% with zinc ion-binding proteins (Figure 1B), pointing to a role of ion-binding proteins, and especially iron ion-binding proteins, in breast cancer with *PIK3CA* mutations. Interestingly, the genes belonging to the metal ion-binding category (Table 2) included two families of genes that were among the most strongly up-regulated in *PIK3CA*-mutated tumors. They comprised four genes of cytochrome P450 family 4 (*CYP4Z1*, *CYP4X1*, *CYP4B1* and the pseudogene (*CYP4Z2P*) and two solute carrier genes (*SLC4A4* and *SLC40A1*). All these genes, with exception of *SLC4A4*, are associated with iron ion binding. In addition to these genes, we found on the top of the list *lactoferrin* (*LTF*), also known to be involved in iron metabolism. Among the genes encoding zinc ion-binding proteins, three (*ANPEP*, *LIMCH1* and *NR2F2*) are known to be cancer-related.

Besides *NR2F2*, five other transcription factors, all known to be involved in tumorigenesis, were identified (Table 2): (a) *TFAP2B*, a tumor suppressor gene in breast cancer [12], (b) *SEC14L2*, a gene possibly involved in the antiproliferative effect of vitamin E in cancer [13], (c) *ID4*, which has been proposed to be involved in breast cancer, inhibiting mammary epithelial cell differentiation and stimulating mammary epithelial cell growth [14], (d) *TCF7L2*, also named *TCF4*, a cancer-promoting gene involved in the Wnt signaling pathway [15], and (e) *MSX2*, a gene implicated in mammary gland and breast cancer development [16], and which is also activated by Wnt signaling [17].

These five transcriptional factors (*TFAP2B*, *SEC14L2*, *ID4*, *TCF7L2* and *MSX2*), as well as ten genes involved in metal ion binding (*CYP4Z1*, *CYP4X1*, *CYP4B1*, *CYP4Z2P*, *SLC4A4*, *SLC40A1*, *LTF*, *ANPEP*, *LIMCH1* and *NR2F2*), were selected for validation by RT-PCR.

#### Pathway analysis of differentially expressed genes

By applying KEGG pathway analysis to the 6124 probes differentially expressed in *PIK3CA*-mutated tumors, we identified physiological pathways directly or indirectly associated with *PIK3CA* mutations. The most significantly overrepresented pathways are shown in Table 1. In addition to signaling pathways in cancer cells, the following five signaling networks were thus identified: MAPK, Calcium, Jak-STAT, Wnt and apoptosis. The Calcium signaling pathway was specifically altered by the down-regulated genes, whereas the Wnt signaling pathway was specifically altered by the up-regulated genes. The same method applied to the 216 probes (153 unique genes) that were up-regulated with FC≥2 also revealed the Wnt signaling pathway (*P = *0.015) (data not shown), highlighting the importance of this pathway in *PIK3CA*-mutated tumors. Five major genes of the Wnt signaling were thus recognized among the 216 probes (Table S2): *MSX2* and *TCF7L2* (already cited), and *WNT5A*, *VANGL2* and *TNFRSF11B*/*osteoprotegerin*. These genes were also selected for RT-PCR validation.

Finally, among the genes up-regulated with FC≥2 (Table S2), we identified *PIK3R1*, the gene encoding the PI3K regulatory subunit, and two other genes of interest: *HMGCS2*, a nuclear gene encoding a mitochondrial matrix enzyme involved in ketogenesis and cholesterol synthesis, processes possibly implicated in the etiology or progression of breast cancer [18] and *MAPT*, a protein enhancing microtubule assembly and stability, that might be involved in taxane resistance [19]. These three genes were added to the RT-PCR validation set.

#### Two-class prediction analysis of differentially expressed genes

Two-class prediction analysis with the PAM algorithm was used to identify the group of genes that best characterized *PIK3CA*-mutated and wild-type tumors and that classified the tumors with the smallest number of predictive features. A threshold of 2.81, that minimized the error, identified 56 differentially expressed probes corresponding to 39 unique genes (Table S4). Thirty-eight of these 39 unique genes were over-expressed in ERα-positive breast tumors with *PIK3CA* mutations, 16 being up-regulated at least 3-fold, while only one gene (*NKAIN1*, encoding Na+/K+ ATPase interacting protein) was down-regulated, with a FC of 3.52. Interestingly, two major genes involved in the Wnt signaling pathway were also identified by PAM, namely *WNT5A* (the most predictive gene) and *TCF7L2*, further confirming the importance of this pathway in *PIK3CA*-mutated tumors. The previously selected up-regulated genes were almost all included in the list of the most predictive genes. PAM analysis identified five interesting new genes that were up-regulated with FC≥3, namely *VTCN1*, *TMC5, NTN4*, *REEP1* and *NRIP3*, which were added to the RT-PCR validation set.

Among the down-regulated genes, *NKAIN1* was selected for RT-PCR validation, along with two other genes known to be involved in cancer biology: *TUSC3* and *TPD52*, that were among the 28 most strongly down-regulated probes (FC≥2) (Table S3) and that were also among the most predictive genes in PAM analysis with a lower FC threshold of 2.5 (data not shown).

Combined analysis of the GO, KEGG and PAM approaches identified 29 most promising genes (26 up-regulated and 3 down-regulated) for RT-PCR validation. The expression status of these genes was first confirmed in the same series of 43 breast tumors (Table 3). Strong positive correlations were observed between the microarray and RT-PCR expression levels of each gene (Spearman's correlation coefficients ranged from 0.69 to 0.97 and were all significant, at *P<*0.0001; data not shown).

**Table 3:** Microarray and RT-PCR analyses of the 29 genes in 43 ERα-positive breast tumors.

		Microarray analysis		RT-PCR analysis			
Symbol Gene	GenBank	FC	*P* value	*PIK3CA* non mutated (n = 29)	*PIK3CA* mutated (n = 14)	FC	*P* value
UP-REGULATED GENES							
ANPEP*	NM_001150	3.52	0.0008	0.17 (0.03–1.52)	0.37 (0.10–23.7)	2.16	0.0033
CYP4B1*	NM_000779	4.12	0.0011	3.13 (0.11–71.5)	10.6 (2.14–431)	3.40	0.0033
*CYP4X1*	NM_178033	5.57	0.0032	1.04 (0.05–73.3)	5.85 (0.63–97.7)	5.62	0.0124
*CYP4Z1*	NM_171834	7.76	0.0035	0.36 (0.01–220)	9.17 (0.10–311)	25.15	0.0085
CYP4Z2P*	NR_002788	3.80	0.0009	34.8 (0.12–1457)	160 (22.9–2103)	4.59	0.0007
HMGCS2*	NM_005518	5.31	0.0003	0.10 (0.00–11.1)	3.40 (0.07–16.3)	32.56	0.0011
ID4*	NM_001546	3.03	0.0002	0.07 (0.02–0.61)	0.16 (0.05–1.03)	2.13	0.0133
LIMCH1*	NM_014988	2.96	0.0002	0.54 (0.10–3.83)	1.66 (0.48–2.87)	3.06	0.0014
LTF*	NM_002343	10.52	0.0005	0.03 (0.00–11.3)	0.86 (0.00–37.4)	31.54	0.0012
MAPT*	NM_016835	2.82	0.0004	1.09 (0.02–12.1)	4.40 (0.04–10.2)	4.02	0.0010
*MSX2*	NM_002449	2.17	0.0011	1.74 (0.09–4.56)	3.32 (1.56–8.57)	1.91	0.0025
*NR2F2*	NM_021005	2.08	0.0063	0.51 (0.14–2.02)	1.06 (0.58–2.20)	2.09	0.0009
NRIP3*	NM_020645	3.28	0.0002	0.94 (0.05–18.9)	3.64 (0.64–33.9)	3.87	0.0025
NTN4*	NM_021229	4.21	0.0008	0.48 (0.05–3.07)	1.87 (0.68–3.19)	3.91	0.0004
PIK3R1*	NM_181523	2.45	<0.0001	0.28 (0.07–0.89)	0.49 (0.18–1.61)	1.74	0.0053
REEP1*	NM_022912	3.30	0.0005	1.15 (0.16–14.4)	3.49 (1.36–8.99)	3.04	0.0446
SEC14L2*	NM_012429	3.03	0.0006	0.98 (0.13–16.1)	5.54 (0.37–24.4)	5.68	0.0049
SLC4A4*	NM_003759	4.81	0.0005	0.28 (0.10–8.45)	3.45 (0.00–116)	12.15	0.0190
SLC40A1*	NM_014585	4.42	0.0005	0.37 (0.11–7.79)	1.14 (0.26–6.62)	3.12	0.0068
*TCF7L2*	NM_030756	2.08	<0.0001	0.24 (0.00–0.64)	0.35 (0.23–0.91)	1.45	0.0010
TFAP2B*	NM_003221	6.68	0.0020	0.09 (0.00–26.0)	1.32 (0.00–34.7)	15.23	0.0164
TMC5*	NM_024780	4.27	0.0022	2.53 (0.05–36.4)	9.45 (1.26–37.8)	3.74	0.0177
*TNFRSF11B*	NM_002546	2.12	0.0023	0.67 (0.13–10.6)	2.64 (0.44–31.3)	3.91	0.0004
*VANGL2*	NM_020335	2.49	0.0009	0.64 (0.03–3.44)	1.90 (0.13–5.37)	2.99	0.0018
VTCN1*	NM_024626	5.47	0.0007	0.19 (0.00–4.89)	1.12 (0.22–23.3)	5.97	0.0025
WNT5A*	NM_003392	3.43	<0.0001	0.56 (0.05–6.03)	2.10 (0.37–6.17)	3.75	0.0013
DOWN-REGULATED GENES							
NKAIN1*	NM_024522	−3.52	0.0006	137.8 (0.94–560)	12.13 (1.39–389)	−11.36	0.0124
*TPD52*	NM_005079	−2.17	0.0014	6.29 (3.13–81.8)	3.88 (1.20–11.68)	−1.62	0.0020
*TUSC3*	NM_006765	−2.48	0.0026	0.58 (0.09–9.35)	0.32 (0.12–0.63)	−1.83	0.0092

For each gene, we report the fold change (FC) between tumors with and without *PIK3CA* mutations. RT-PCR results are expressed as the median (range) mRNA level for each gene relative to normal breast tissues. Genes identified by PAM analysis are marked with an asterisk.

### mRNA expression of the 29 genes of interest in 249 ERα-positive breast tumors

#### Overall expression of the 29 differentially expressed genes

The expression levels of the 29 genes selected by microarray analysis were then verified by RT-PCR in a large independent cohort of 249 ERα-positive breast tumors, of which 157 were *PIK3CA* wild-type and 92 were *PIK3CA-*mutated (Table S1). This *PIK3CA* mutation frequency of 37% was in keeping with the results of previous studies showing a mutation rate of up to 40% in ERα-positive breast tumors [7], [8]. Almost all the tumors had a single mutation, 44 (47.8%) in exon 9 (helical domain) and 46 (50%) in exon 20 (kinase domain) [7]. Two tumors (2.2%) carried two mutations, located in exons 9 and 20 in one case, and in exon 20 in the second case.

Nineteen (66%) of the 29 selected genes showed significantly different expression between mutated and wild-type tumors in the validation cohort, with a distribution similar to that observed in the screening cohort (Table 4). Among the three down-regulated genes of interest in the screening set, only *NKAIN1* was significantly down-regulated in the validation set. Among the 26 up-regulated genes, 18 were also up-regulated in the validation set. With exception of *VANGL2*, up-regulation of the genes involved in Wnt signaling pathway, namely *WNT5A*, *MSX2*, *TCF7L2* and *TNFRSF11B*, was confirmed in the validation set, further emphasizing the important role of the Wnt signaling pathway in *PIK3CA*-mutated breast cancer. Up-regulation was also confirmed for genes related to breast cancer (*MAPT*, *HMGCS2*, *NR2F2* and *TFAP2B*), genes involved in metal ion binding (*CYP4Z1*, *CYP4Z2P*, *SLC40A1*, *LTF* and *LIMCH1*) and also *NRIP3, NTN4, REEP1, SEC14L2* and *TMC5*. Deregulation of these genes was not related to ERα status or proliferation since similar expression levels of *ER*α and *MKI67* were observed in *PIK3CA*-mutated and -non mutated tumors (Table 4). Only 2 of the 29 selected genes showed significantly different expression between *PIK3CA* exon 9- and exon 20-mutated tumors, namely *TFAP2B* and *NRIP3* (Table S5). Interestingly, *TFAP2B* was over-expressed in exon 20-mutated tumors and *NRIP3* in exon 9-mutated tumors.

**Table 4:** Relative mRNA expression levels of the 29 genes in 249 ERα-positive breast tumors.

Symbol Gene	GenBank	*PIK3CA* non mutated (n = 157)	*PIK3CA* mutated (n = 92)	FC	*P* value	Rank in PAM
UP-REGULATED GENES						
*ANPEP*	NM_001150	0.46 (0.00–154)	0.39 (0.06–18.3)	−0.84	ns	15
*CYP4B1*	NM_000779	6.59 (0.00–222)	5.72 (0.00–178)	−1.12	ns	20
*CYP4X1*	NM_178033	2.34 (0.02–59)	3.78 (0.05–101)	1.62	ns	11
*CYP4Z1*	NM_171834	1.15 (0.01–140)	2.97 (0.01–254)	2.58	0.0134	4
*CYP4Z2P*	NR_002788	38.3 (0.00–1815)	66.4 (0.00–1069)	1.74	0.0060	8
*HMGCS2*	NM_005518	0.29 (0.00–24.8)	0.60 (0.00–25.7)	2.09	0.0487	10
*ID4*	NM_001546	0.13 (0.00–9.10)	0.17 (0.02–9.57)	1.30	ns	28
*LIMCH1*	NM_014988	0.73 (0.05–6.59)	1.09 (0.08–8.58)	1.49	0.0007	19
*LTF*	NM_002343	0.08 (0.00–14.7)	0.14 (0.00–41.8)	1.74	0.0036	7
*MAPT*	NM_016835	3.03 (0.07–71.1)	4.52 (0.15–26.2)	1.49	0.0039	14
*MSX2*	NM_002449	2.26 (0.00–13.9)	3.69 (0.11–39.3)	1.63	0.0003	5
*NR2F2*	NM_021005	0.79 (0.06–10.8)	1.00 (0.11–7.27)	1.25	0.0415	24
*NRIP3*	NM_020645	1.55 (0.00–168)	2.69 (0.10–105)	1.73	0.0250	16
*NTN4*	NM_021229	0.75 (0.03–5.47)	1.17 (0.04–10.2)	1.57	0.0002	12
*PIK3R1*	NM_181523	0.32 (0.06–1.38)	0.37 (0.08–1.30)	1.16	ns	27
*REEP1*	NM_022912	1.85 (0.00–12.1)	2.59 (0.19–21.8)	1.40	0.0053	6
*SEC14L2*	NM_012429	2.49 (0.00–24.0)	4.51 (0.16–39.1)	1.81	<0.0001	2
*SLC4A4*	NM_003759	0.29 (0.00–178)	0.42 (0.00–128)	1.43	ns	17
*SLC40A1*	NM_014585	0.88 (0.03–7.81)	1.22 (0.00–17.9)	1.38	0.0311	13
*TCF7L2*	NM_030756	0.26 (0.03–1.05)	0.32 (0.06–1.26)	1.21	0.0373	26
*TFAP2B*	NM_003221	1.28 (0.00–35.7)	5.53 (0.00–179)	4.31	0.0055	3
*TMC5*	NM_024780	4.79 (0.01–69.0)	5.77 (0.11–46.2)	1.20	0.0331	9
*TNFRSF11B*	NM_002546	1.25 (0.00–50.3)	1.90 (0.15–21.8)	1.52	0.0068	21
*VANGL2*	NM_020335	0.73 (0.03–4.64)	0.82 (0.07–9.09)	1.12	ns	23
*VTCN1*	NM_024626	0.61 (0.00–10.3)	0.64 (0.01–15.4)	1.05	ns	22
*WNT5A*	NM_003392	0.74 (0.03–12.4)	1.17 (0.18–7.27)	1.59	<0.0001	18
DOWN-REGULATED GENES						
*NKAIN1*	NM_024522	81.1 (0.54–1648)	57.7 (0.71–560)	−1.41	0.0471	1
*TPD52*	NM_005079	6.01 (1.30–115)	5.34 (1.75–80.9)	−1.12	ns	25
*TUSC3*	NM_006765	0.68 (0.08–3.72)	0.63 (0.08–6.31)	−1.09	ns	29
CONTROL GENES						
*ERα*	NM_000125	8.77 (1.27–68.9)	8.86 (1.59–39.8)	1.01	ns	−
*MKI67*	NM_002417	12.1 (0.86–57.2)	11.0 (1.79–313)	0.91	ns	−

ns: not significant.

Results are expressed as the median (range) mRNA level for each gene relative to normal breast tissues. For each gene, we report the fold change (FC) between tumors with and without *PIK3CA* mutations and the PAM rank.

#### Identification of the most discriminatory genes

PAM prediction analysis was then used to test the ability of each gene to classify the 249 ERα-positive breast tumors according to *PIK3CA* mutation status. *NKAIN1* was the most predictive gene (PAM rank) (Table 4). *NKAIN1* was also an essential classifier in supervised hierarchical clustering analysis. Indeed, the 19-gene set including *NKAIN1* classified the 249 breast tumors significantly more accurately than the set of 18 up-regulated genes without *NKAIN1* (accuracy 59% and 57%, Χ^2^ test *P* values of 0.0006 and 0.0141, respectively) (Table S6). Three different minimal sets of 4, 5 and 6 genes, all including both *NKAIN1* and *CYP4Z2P*, showed the same overall clustering accuracy of 59.4% (Table S6). However, the 5-gene group (*NKAIN1*-*CYP4Z2P*-*NRIP3*-*SEC14L2*-*TFAP2B*) had the most significant discriminatory value (MCC = 0.2334, *P = *0.0002) correctly clustering 66 of the 92 mutated tumors and 82 of the 157 non mutated tumors. Notably, this 5-gene set contained the two genes that were differently expressed between exon 9- and exon 20-mutated tumors, and thus had the best capacity to distinguish between these two tumor categories (data not shown). The other two gene sets, both comprising genes involved in Wnt signaling (*NKAIN1*-*CYP4Z2P*-*WNT5A*-*TMC5* and *NKAIN1*-*CYP4Z2P*-*WNT5A*-*MAPT*-*MSX2*-*TFAP2B*), classified 65 mutated and 83 non mutated tumors correctly (MCC = 0.2286, *P = *0.0003).

## Discussion

We used a two-step strategy to identify downstream target genes and signaling pathways affected by *PIK3CA* mutations in breast tumors. We first applied a pangenomic oligonucleotide microarray approach to a series of 43 ERα-positive tumors with and without *PIK3CA* mutations, and then validated genes of interest by RT-PCR in an independent series of 249 ERα-positive tumors. A robust set of 19 genes differentially expressed in *PIK3CA*-mutated and wild-type tumors was thus identified.

Over-expression of several genes involved in Wnt signaling (*WNT5A*, *TCF7L2*, *MSX2* and *TNFRSF11B*), regulation of gene transcription (*SEC14L2*, *MSX2*, *TFAP2B* and *NRIP3*) and metal ion binding (*CYP4Z1*, *CYP4Z2P*, *SLC40A1*, *LTF* and *LIMCH1*) was observed in *PIK3CA*-mutated tumors. Several of these genes have been linked to breast cancer (*MAPT*, *HMGCS2*, *NR2F2*, *TFAP2B*, *NTN4*, *SEC14L2* and *LTF*).

The human Wnt signaling network is important for regulation of proliferation, differentiation, growth and survival from the embryo stage [20], [21]. Crosstalk of complex pathways belonging to Wnt signaling has been observed leading to, when altered, disparate effects in different tumor types [22]-[24]. We observed over-expression of four major genes involved in the Wnt pathway, namely *WNT5A*, *TCF7L2*, *MSX2* and *TNFRSF11B*. *WNT5A* encodes a major Wnt ligand affecting tumor cell motility and metastasis, but its role in breast cancer is controversial [23]. The emerging view is that, in breast cancer, *WNT5A* has a suppressive effect, inhibiting migration and invasion of breast cancer cell lines [24]. Moreover, *WNT5A* over-expression observed in invasive breast tumors has been associated with a favorable outcome [24]. *PIK3CA* mutations have also been associated with favorable outcome of breast cancer patients [1], [3], [4], [25]. We can thus suggest a link between gain-of-function mutation in *PIK3CA*, up-regulation of *WNT5A* and favorable outcome in breast cancer. We also observed over-expression of *TCF7L2,* which encodes one of the four major transcription factors involved in the Wnt signaling pathway [20], [26], as well as two other genes (*MSX2* and *TNFRSF11B*) known to be downstream targets of the Wnt signaling pathway [27]–[29]. Wnt signaling has a major role in cancer stem cell self-renewal and tumor maintenance [20], [30] and contributes to tumor invasion, metastasis and angiogenesis [31]. Recent studies have identified a role of Wnt pathway in epidermal-mesenchymal transition during breast cancer development [32], [33]. Thus, Wnt pathway activation appears to be an important consequence of *PIK3CA* mutations in breast tumors, in keeping with recently observed crosstalk between the PI3K/Akt and Wnt pathways in both physiological (myeloid progenitor cells) [21] and pathological conditions (medulloblastoma) [34].

Better understanding of the biological functions of the Wnt and PI3K/Akt pathways and their interplay could have therapeutic implications for breast cancer. Drugs targeting the PI3K/Akt pathway have given promising preliminary results in human malignancies [35], [36]. However, as the PI3K pathway is crucial for metabolic processes, PI3K inhibitors might also have side effects, especially affecting insulin signaling and cardiac functions [36], [37]. In contrast, targeting of downstream Wnt signaling events might have fewer adverse effects, considering their crucial importance in embryonic development [23], [38].

Genes encoding metal ion-binding proteins were also over-expressed in *PIK3CA*-mutated tumors. Such metal ion-binding proteins have regulatory roles in central cellular processes such as gene expression, proliferation, differentiation and survival. Increased expression of these proteins in ERα-positive breast tumors has also been reported by Abba et al. [39]. We observed over-expression of *LIMCH1*, a gene encoding zinc-binding protein, and also four genes encoding iron-binding proteins (*LTF*, *SLC40A1*, *CYP4Z1* and *CYP4Z2P*) previously linked to breast cancer. *LTF* encodes lactoferrin, a protein involved in non specific immunity and that may inhibit carcinogenesis and tumor growth [40]. *CYP4Z1* and its pseudogene *CYP4Z2P* are two members of cytochrome P450 family 4 which have been found to be over-expressed in about 50% of breast cancers relative to normal breast tissue from the same patients [41]. Here, we confirm that the pseudogene *CYP4Z2P* is expressed in both *PIK3CA-*mutated and -non mutated ERα-positive breast tumors, by using specific primers unambiguously distinguishing *CYP4Z2P* from *CYP4Z1*. Thus, *CYP4Z2P* is transcriptionally active, but its translation remains to be studied. *CYP4Z2P* is located in a head-to-head orientation close to *CYP4Z1* in chromosome region 1p33 [41], raising the possibility that expression of these two genes is co-regulated in *PIK3CA-*mutated breast tumors.

We identified several genes previously implicated in breast cancer development or outcome. The proteins encoded by *TFAP2B*, *NTN4* and *SEC14L2* have been linked to tumors with less aggressive features and better outcome [12], [13], [42]. *MAPT* has been proposed as a predictive marker of taxane responsiveness in breast cancer [43]. *NR2F2* has been also detected up-regulated in breast cancer, but its involvement in tumor development remains elusive because of its ability to affect both pro-oncogenic and anti-oncogenic proteins [44], [45]. *HMGCS2* was recently shown to be regulated in response to hormonal stimulation [18].

*NRIP3*, *TMC5*, *REEP1* and *NKAIN1*, whose expression had not previously been described in breast cancer, were also deregulated in the *PIK3CA*-mutated breast tumors. *NRIP3*, *TMC5* and *REEP1* are differentially expressed in various other tumor types [46]–[48]. Interestingly, *NKAIN1* was the only gene under-expressed in *PIK3CA*-mutated tumors and was also the most discriminatory gene for these tumors. The role of these genes in breast cancer development remains to be evaluated in following studies.

Recently, Loi et al. identified a 278 gene-expression signature associated specifically with *PIK3CA* exon 20-mutated ER-positive/ERBB2-negative tumors [25]. These authors observed an unexpected significant down-expression of some Akt-regulated genes such as *RPS6KB1* in their *PIK3CA*-mutated tumor series, but a normal level of *AKT1* and *mTOR* transcripts. They also showed that phosphor-Akt expression was not significantly up-regulated at the protein level. In the present study, we did not identify *RPS6KB1*, *AKT1* and *mTOR* in our final 19-gene set nor in the list of 6124 genes differentially expressed in *PIK3CA*-mutated tumors (Table S2 and S3). Interestingly, among the 168 significantly up-regulated genes detected by Loi et al., *WNT5A* and *MSX2*, as well as *HMGCS2* and *LTF*, were identified in agreement with our results. The data of Loi et al. [25] confirm thus the positive association between *PIK3CA* mutation and Wnt signaling pathway activation reported in the present manuscript.

In conclusion, this gene expression profiling study suggests that over-expression of genes belonging to the Wnt signaling pathway may play a pivotal role in *PIK3CA*-mutated breast tumors, in particular *WNT5A*. Further studies of biological mechanisms affected by *PIK3CA* mutations may have therapeutic implications.

## Supporting Information

Table S1 **Molecular, pathological and clinical characteristics of patients in relation to metastasis free survival (MFS) in the 43 ERα-positive and 249 ERα-positive patient series.**
(PDF)

Table S2 **2538 probes up-regulated in tumors with *PIK3CA* mutations (Mutated) compared to tumors without *PIK3CA* mutation (Normal) with a *P* value <0.05 identified by a parametric *t* test using BRB ArrayTools.** These genes were ranked according to fold change (FC) calculated between expression intensities of tumors with *PIK3CA* mutations and those of tumors without *PIK3CA* mutation. The 216 probes with a FC≥2 are put in bold. In this list, the probes belonging to Wnt signaling pathway are shaded in light grey, and *PIK3R1*, *HMGCS2* and *MAPT* are shaded in dark grey.
(PDF)

Table S3 **3586 probes down-regulated in tumors with *PIK3CA* mutations (Mutated) compared to tumors without *PIK3CA* mutation (Normal) with a *P* value <0.05 identified by a parametric *t* test using BRB ArrayTools.** These genes were ranked according to fold change (FC) calculated between expression intensities of tumors with *PIK3CA* mutations and those of tumors without *PIK3CA* mutation. The 28 probes with a FC≥2 are put in bold.
(PDF)

Table S4 **List of 56 probes (39 unique genes) deregulated in REα-positive breast tumors with *PIK3CA* mutations compared to those without *PIK3CA* mutation identified by PAM.** These genes are presented according to the rank in PAM output. For each gene, we report the fold change (FC) calculated between expression intensities of tumors with and without *PIK3CA* mutations using BRB Arrays Tools. The genes with a FC≥3 are put in bold.
(PDF)

Table S5 **Relative mRNA expression levels of the 29 genes in the 44 ERα-positive breast tumors with exon 9 *PIK3CA* mutations compared to the 47 tumors with exon 20 *PIK3CA* mutations.** The tumor with *PIK3CA* mutations in both exon 9 and exon 20 was excluded from the analysis. For each gene, we report the median (range) of the mRNA levels of each gene relative to normal breast tissue samples, the fold change (FC) between tumors with exon 9-mutated and exon 20-mutated *PIK3CA* and the *P* value associated to Mann-Whitney *U* test.
(PDF)

Table S6 Supervised hierarchical clustering analysis of the 249 ERα-positive breast tumors. Classification performance of discriminating gene sets identified from the 19 significantly deregulated genes in tumors with *PIK3CA* mutations (18 up-regulated genes + *NKAIN1*). Each gene set separates the 157 tumors without *PIK3CA* mutation (N) and the 92 tumors with *PIK3CA* mutations (M) in two main clusters (cluster 1 and 2).
(PDF)
