# Supplementary material for: Gene Expression Profiling Reveals New Aspects of PIK3CA Mutation in ERalpha-Positive Breast Cancer: Major Implication of the Wnt Signaling Pathway
Source: PLoS One. 2010 Dec 30;5(12):e15647. doi: 10.1371/journal.pone.0015647 (PMC3012715; doi:10.1371/journal.pone.0015647)
Supplement: Table S1 — Molecular, pathological and clinical characteristics of patients in relation to metastasis free survival (MFS) in the 43 ERα-positive and 249 ERα-positive patient series. (PDF) [file pone.0015647.s001.pdf]

Characteristic	Screening set (n=43) Number of patients	Number of events (%)	MFS p-value[a]	Validation set (n=249) Number of patients	Number of events (%)	MFS p-value[a]
**Age**			0.037			0.746
≤ 70 years	26	13 (50)		173	72 (42)	
> 70 years	17	3 (18)		76	29 (38)	
**SBR histological grade[b,c]**			0.431			0.136
I + II	31	10 (32)		186	71 (38)	
III	11	6 (55)		59	29 (49)	
**Lymph node status[c]**			0.503			0.022
Negative	28	10 (36)		47	13 (28)	
Positive	15	6 (40)		198	87 (44)	
**Macroscopic tumor size[c]**			0.369			<0.0001
≤ 30mm	32	11 (34)		168	56 (33)	
> 30mm	8	5 (63)		75	43 (57)	
***ERBB2* Status[c]**			0.681			0.681
Negative	38	14 (37)		200	83 (42)	
Positive	5	2 (40)		48	18 (38)	
***PIK3CA* status**			0.167			0.472
Non mutated	29	13 (45)		157	65 (42)	
Mutated	14	3 (21)		92	36 (39)	

[a] Log-rank test

[b] Scarff Bloom Richardson classification

[c] Histological information were not available for all tumors
