# Supplementary material for: Gene Expression Profiling Reveals New Aspects of PIK3CA Mutation in ERalpha-Positive Breast Cancer: Major Implication of the Wnt Signaling Pathway
Source: PLoS One. 2010 Dec 30;5(12):e15647. doi: 10.1371/journal.pone.0015647 (PMC3012715; doi:10.1371/journal.pone.0015647)
Supplement: Table S2 — 2538 probes up-regulated in tumors with PIK3CA mutations (Mutated) compared to tumors without PIK3CA mutation (Normal) with a P value <0.05 identified by a parametric t test using BRB ArrayTools. These genes were ranked according to fold change (FC) calculated between expression intensities of tumors with PIK3CA mutations and those of tumors without PIK3CA mutation. The 216 probes with a FC≥2 are put in bold. In this list, the probes belonging to Wnt signaling pathway are shaded in light grey, and PIK3R1, HMGCS2 and MAPT are shaded in dark grey. (PDF) [file pone.0015647.s002.pdf]

Index	Mutated*	Normal*	FC	*P* value	FDR**	Probe set	Description	Gene symbol
113	1342	128	10,52	0,000503	0,240	202018_s_at	Lactotransferrin	*LTF*
616	362	46,7	7,76	0,003493	0,308	237395_at	Cytochrome P450, family 4, subfamily Z, polypeptide 1	*CYP4Z1*
3073	1009	142	7,10	0,021674	0,385	206378_at	Secretoglobin, family 2A, member 2	*SCGB2A2*
376	119	17,7	6,68	0,001962	0,284	214451_at	Transcription factor AP-2 beta	*TFAP2B*
578	337	60,5	5,57	0,003174	0,300	227702_at	Cytochrome P450, family 4, subfamily X, polypeptide 1	*CYP4X1*
146	210	38,4	5,47	0,000669	0,244	219768_at	V-set domain containing T cell activation inhibitor 1	*VTCN1*
86	129	24,2	5,31	0,000327	0,201	204607_at	3-hydroxy-3-methylglutaryl-Coenzyme A synthase 2 (mitochondrial)	*HMGCS2*
2613	78,7	15,9	4,95	0,017744	0,371	205358_at	Glutamate receptor, ionotropic, AMPA 2	*GRIA2*
116	23,2	4,83	4,81	0,000513	0,240	203908_at	Solute carrier family 4, sodium bicarbonate cotransporter, member 4	*SLC4A4*
117	79,4	18,0	4,42	0,000518	0,240	239723_at	Solute carrier family 40 (iron-regulated transporter), member 1	*SLC40A1*
625	56,6	13,0	4,34	0,003549	0,308	1553394_a_a	Transcription factor AP-2 beta	*TFAP2B*
413	148	34,6	4,28	0,002175	0,286	240304_s_at	Transmembrane channel-like 5	*TMC5*
5181	216	50,9	4,25	0,039946	0,421	223864_at	Ankyrin repeat domain 30A	*ANKRD30A*
179	446	106	4,21	0,000826	0,244	223315_at	Netrin 4	*NTN4*
246	120	29,2	4,12	0,001128	0,251	210096_at	Cytochrome P450, family 4, subfamily B, polypeptide 1	*CYP4B1*
1043	312	80,2	3,89	0,006080	0,319	204041_at	Monoamine oxidase B	*MAOB*
192	44,1	11,6	3,80	0,000899	0,244	1553434_at	Cytochrome P450 4Z2 pseudogene	*CYP4Z2P*
2038	52,4	14,3	3,65	0,013273	0,355	211682_x_at	UDP glucuronosyltransferase 2 family, polypeptide B28	*UGT2B28*
819	69,6	19,6	3,54	0,004678	0,312	218963_s_at	Keratin 23 (histone deacetylase inducible)	*KRT23*
451	56,1	15,9	3,53	0,002410	0,288	230238_at	Ankyrin repeat domain 43	*ANKRD43*
177	85,8	24,3	3,52	0,000819	0,244	202888_s_at	Alanyl (membrane) aminopeptidase	*ANPEP*
1	40,9	11,9	3,43	0,000004	0,099	231227_at	Wingless-type MMTV integration site family, member 5A	*WNT5A*
1208	121	35,3	3,42	0,007161	0,324	219580_s_at	Transmembrane channel-like 5	*TMC5*
118	171	51,7	3,30	0,000526	0,240	204364_s_at	Receptor accessory protein 1	*REEP1*
680	15,2	4,62	3,29	0,003908	0,308	232277_at	Solute carrier family 28 member 3	*SLC28A3*
48	91,7	28,0	3,28	0,000195	0,201	219557_s_at	Nuclear receptor interacting protein 3	*NRIP3*
1871	339	105	3,24	0,012080	0,353	204014_at	Dual specificity phosphatase 4	*DUSP4*
1000	123	38,0	3,23	0,005782	0,316	203355_s_at	Pleckstrin and Sec7 domain containing 3	*PSD3*
521	80,7	25,4	3,18	0,002775	0,291	219630_at	PDZK1 interacting protein 1	*PDZK1IP1*
3570	73,4	23,7	3,10	0,025784	0,394	226147_s_at	Polymeric immunoglobulin receptor	*PIGR*
1001	20,8	6,75	3,08	0,005791	0,316	238133_at	Netrin G1	*NTNG1*
2553	1238	402	3,08	0,017275	0,370	219197_s_at	Signal peptide, CUB domain, EGF-like 2	*SCUBE2*
6088	237	77,6	3,06	0,049699	0,446	229764_at	Tumor protein p63 regulated 1	*TPRG1*
133	66,1	21,7	3,05	0,000589	0,240	230316_at	SEC14-like 2 (S. cerevisiae)	*SEC14L2*
102	79,0	26,1	3,03	0,000402	0,216	204541_at	SEC14-like 2 (S. cerevisiae)	*SEC14L2*
53	19,2	6,32	3,03	0,000205	0,201	209292_at	Inhibitor of DNA binding 4, dominant negative helix-loop-helix	*ID4*
98	28,1	9,36	3,00	0,000382	0,213	204855_at	Serpin peptidase inhibitor, clade B (ovalbumin), member 5	*SERPINB5*
150	1110	371	2,99	0,000691	0,244	227556_at	Nucleoside diphosphate kinase 7	*NME7*
4302	326	110	2,97	0,032016	0,407	212774_at	Zinc finger protein 238	*ZNF238*
1484	65,9	22,2	2,97	0,009119	0,336	220177_s_at	Transmembrane protease, serine 3	*TMPRSS3*
15	129	43,4	2,96	0,000054	0,191	209291_at	Inhibitor of DNA binding 4, dominant negative helix-loop-helix	*ID4*
268	204	68,9	2,96	0,001277	0,260	217966_s_at	Family with sequence similarity 129, member A	*FAM129A*
55	110	37,1	2,96	0,000212	0,201	212325_at	LIM and calponin homology domains 1	*LIMCH1*
987	113	38,1	2,96	0,005695	0,315	222904_s_at	Transmembrane channel-like 5	*TMC5*
227	40,0	13,6	2,93	0,001050	0,250	222862_s_at	Adenylate kinase 5	*AK5*
284	177	60,7	2,92	0,001390	0,263	222288_at	Hypothetical protein FLJ20489	-
392	211	72,6	2,91	0,002058	0,285	227182_at	Sushi domain containing 3	*SUSD3*
389	45,7	15,8	2,89	0,002023	0,284	223949_at	Transmembrane protease, serine 3	*TMPRSS3*
194	26,6	9,28	2,87	0,000900	0,244	205513_at	Transcobalamin I (vitamin B12 binding protein, R binder family)	*TCN1*
1174	283	99,0	2,86	0,006896	0,321	204015_s_at	Dual specificity phosphatase 4	*DUSP4*
555	78,8	27,6	2,85	0,003021	0,296	1553589_a_a	PDZK1 interacting protein 1	*PDZK1IP1*
28	121	42,5	2,84	0,000131	0,201	205990_s_at	Wingless-type MMTV integration site family, member 5A	*WNT5A*
364	83,8	29,7	2,82	0,001899	0,284	203354_s_at	Pleckstrin and Sec7 domain containing 3	*PSD3*
100	144	51,0	2,82	0,000390	0,213	206401_s_at	Microtubule-associated protein tau	*MAPT*
72	46,1	16,4	2,81	0,000277	0,201	213425_at	Wingless-type MMTV integration site family, member 5A	*WNT5A*
27	1769	641	2,76	0,000127	0,201	201242_s_at	ATPase, Na+/K+ transporting, beta 1 polypeptide	*ATP1B1*
2139	167	61,4	2,72	0,014063	0,359	225728_at	Sorbin and SH3 domain containing 2	*SORBS2*

Index	Mutated*	Normal*	FC	*P* value	FDR**	Probe set	Description	Gene symbol
204	183	67,2	2,72	0,000935	0,244	228481_at	Periostin, osteoblast specific factor	*POSTN*
56	873	322	2,71	0,000216	0,201	201243_s_at	ATPase, Na+/K+ transporting, beta 1 polypeptide	*ATP1B1*
280	27,5	10,2	2,71	0,001368	0,263	219308_s_at	Adenylate kinase 5	*AK5*
1117	21,9	8,12	2,70	0,006533	0,319	204455_at	Dystonin	*DST*
548	23,9	8,88	2,69	0,002964	0,295	1563367_at	Hypothetical protein LOC100128977	*LOC100128977*
3020	54,5	20,2	2,69	0,021271	0,385	207981_s_at	Estrogen-related receptor gamma	*ESRRG*
19	273	101	2,69	0,000081	0,201	212328_at	LIM and calponin homology domains 1	*LIMCH1*
1231	188	70,0	2,69	0,007320	0,324	204288_s_at	Sorbin and SH3 domain containing 2	*SORBS2*
1794	144	54,5	2,64	0,011486	0,350	203130_s_at	Kinesin family member 5C	*KIF5C*
714	63,3	24,1	2,63	0,004096	0,308	227154_at	Immunoglobin superfamily, member 21	*IGSF21*
1524	47,2	18,0	2,62	0,009416	0,337	231098_at	Transcribed locus	-
375	43,6	16,7	2,61	0,001960	0,284	226847_at	Follistatin	*FST*
590	59,4	22,9	2,59	0,003254	0,301	243806_at	ST8 alpha-N-acetyl-neuraminide alpha-2,8-sialyltransferase 6	*SIAT8F*
4089	55,1	21,3	2,58	0,030066	0,402	209480_at	Major histocompatibility complex, class II, DQ beta 1	*HLA-DQB1*
2399	588	228	2,58	0,015996	0,365	207847_s_at	Mucin 1, cell surface associated	*MUC1*
1299	85,1	33,4	2,55	0,007740	0,325	219440_at	Retinoic acid induced 2	*RAI2*
256	57,6	22,6	2,55	0,001196	0,255	222900_at	Nuclear receptor interacting protein 3	*NRIP3*
1455	103	40,5	2,53	0,008896	0,334	211000_s_at	Interleukin 6 signal transducer (gp130, oncostatin M receptor)	*IL6ST*
5348	61,6	24,4	2,52	0,041927	0,429	209351_at	Keratin 14	*KRT14*
196	44,4	17,8	2,50	0,000908	0,244	226029_at	Vang-like 2 (van gogh, Drosophila)	*VANGL2*
166	193	77,3	2,49	0,000777	0,244	212327_at	LIM and calponin homology domains 1	*LIMCH1*
141	86,0	34,5	2,49	0,000645	0,244	243030_at	Mitogen-activated protein kinase kinase kinase 1	*MAP3K1*
90	242	97,2	2,49	0,000332	0,201	202149_at	Neural precursor cell expressed, developmentally down-regulated 9	*NEDD9*
1591	1263	509	2,48	0,009901	0,340	201667_at	Gap junction protein, alpha 1, 43kDa	*GJA1*
1408	62,9	25,5	2,47	0,008513	0,330	224358_s_at	Membrane-spanning 4-domains, subfamily A, member 7	*MS4A7*
5145	52,1	21,1	2,46	0,039615	0,421	213695_at	Paraoxonase 3	*PON3*
1310	426	173	2,46	0,007819	0,325	217967_s_at	Family with sequence similarity 129, member A	*FAM129A*
8	359	146	2,46	0,000015	0,099	212240_s_at	Phosphoinositide-3-kinase, regulatory subunit 1 (p85 alpha)	*PIK3R1*
658	41,6	17,1	2,43	0,003814	0,308	239907_at	Transcribed locus	-
2749	794	327	2,43	0,019005	0,378	202357_s_at	Complement factor B	*CFB*
4852	55,2	22,8	2,42	0,036972	0,417	229030_at	Calpain 8	*CAPN8*
2562	48,7	20,1	2,42	0,017375	0,371	209369_at	Annexin A3	*ANXA3*
4	168	69,6	2,41	0,000011	0,099	203407_at	Periplakin	*PPL*
1198	49,1	20,3	2,41	0,007070	0,323	202037_s_at	Secreted frizzled-related protein 1	*SFRP1*
3864	30,6	12,7	2,41	0,028229	0,399	226884_at	Leucine rich repeat neuronal 1	*LRRN1*
2945	125	52,0	2,41	0,020642	0,383	203021_at	Secretory leukocyte peptidase inhibitor	*SLPI*
450	144	60,4	2,38	0,002404	0,288	203928_x_at	Microtubule-associated protein tau	*MAPT*
1985	561	236	2,37	0,012957	0,355	204320_at	Collagen, type XI, alpha 1	*COL11A1*
3267	738	312	2,37	0,023215	0,388	37892_at	Collagen, type XI, alpha 1	*COL11A1*
4326	257	109	2,37	0,032194	0,407	226034_at	cDNA clone IMAGE:3881549	-
101	108	45,5	2,36	0,000401	0,216	229518_at	Family with sequence similarity 46, member B	*FAM46B*
1352	56,7	24,0	2,36	0,008118	0,328	235651_at	-	-
1318	39,3	16,7	2,36	0,007843	0,325	213060_s_at	Chitinase 3-like 2	*CHI3L2*
1191	87,5	37,3	2,35	0,007032	0,322	203240_at	FC fragment of IgG binding protein	*FCGBP*
66	59,8	25,5	2,35	0,000251	0,201	241459_at	LIM and calponin homology domains 1	*LIMCH1*
2729	121	51,6	2,34	0,018795	0,377	225871_at	Six transmembrane epithelial antigen of the prostate 2	*STEAP2*
1053	74,8	32,0	2,34	0,006149	0,319	1555497_a_a	Cytochrome P450, family 4, subfamily B, polypeptide 1	*CYP4B1*
49	101	43,2	2,34	0,000195	0,201	212249_at	Phosphoinositide-3-kinase, regulatory subunit 1 (p85 alpha)	*PIK3R1*
1336	124	53,1	2,33	0,008019	0,328	228648_at	Leucine-rich alpha-2-glycoprotein 1	*LRG1*
1772	94,8	40,7	2,33	0,011296	0,348	218613_at	Pleckstrin and Sec7 domain containing 3	*PSD3*
154	251	108	2,32	0,000703	0,244	206385_s_at	Ankyrin 3, node of Ranvier (ankyrin G)	*ANK3*
1550	110	47,4	2,31	0,009560	0,337	204863_s_at	Interleukin 6 signal transducer (oncostatin M receptor)	*IL6ST*
1876	284	123	2,31	0,012131	0,354	204284_at	Protein phosphatase 1, regulatory (inhibitor) subunit 3C	*PPP1R3C*
2291	11,7	5,10	2,30	0,015235	0,363	244623_at	Potassium voltage-gated channel, KQT-like subfamily, member 5	*KCNQ5*
631	261	114	2,30	0,003570	0,308	226597_at	Receptor accessory protein 6	*REEP6*
2117	101	44,3	2,29	0,013891	0,359	233123_at	Solute carrier family 40 (iron-regulated transporter), member 1	*SLC40A1*
5206	57,5	25,1	2,29	0,040172	0,422	203485_at	Reticulon 1	*RTN1*

Index	Mutated*	Normal*	FC	*P* value	FDR**	Probe set	Description	Gene symbol
857	26,6	11,7	2,28	0,004948	0,313	209900_s_at	Solute carrier family 16, member 1	*SLC16A1*
1366	338	149	2,27	0,008221	0,328	232458_at	Collagen, type III, alpha 1	*COL3A1*
2300	1064	468	2,27	0,015280	0,363	213693_s_at	Mucin 1, cell surface associated	*MUC1*
579	119	52,4	2,27	0,003176	0,300	203929_s_at	Microtubule-associated protein tau	*MAPT*
1376	307	136	2,27	0,008295	0,329	229218_at	Collagen, type I, alpha 2	*COL1A2*
745	141	62,3	2,27	0,004223	0,308	213110_s_at	Collagen, type IV, alpha 5	*COL4A5*
1138	991	438	2,26	0,006647	0,319	223044_at	Solute carrier family 40 (iron-regulated transporter), member 1	*SLC40A1*
933	257	114	2,25	0,005358	0,313	235371_at	Glycosyltransferase 8 domain containing 4	*GLT8D4*
5445	36,7	16,3	2,25	0,042949	0,431	222853_at	Fibronectin leucine rich transmembrane protein 3	*FLRT3*
3180	161	71,7	2,24	0,022552	0,387	203058_s_at	3'-phosphoadenosine 5'-phosphosulfate synthase 2	*PAPSS2*
835	376	168	2,24	0,004769	0,312	223343_at	Membrane-spanning 4-domains, subfamily A, member 7	*MS4A7*
431	29,8	13,3	2,24	0,002272	0,288	211828_s_at	TRAF2 and NCK interacting kinase	*TNIK*
93	27,7	12,4	2,23	0,000347	0,204	229994_at	Glyceronephosphate O-acyltransferase	*GNPAT*
563	122	54,9	2,23	0,003070	0,298	204365_s_at	Receptor accessory protein 1	*REEP1*
3763	189	84,7	2,23	0,027356	0,397	239435_x_at	Shroom family member 1	*SHROOM1*
1093	86,2	38,7	2,23	0,006408	0,319	226992_at	Nitric oxide synthase trafficker	*NOSTRIN*
4697	16,0	7,21	2,23	0,035470	0,413	235763_at	Solute carrier family 44, member 5	*SLC44A5*
203	442	199	2,22	0,000934	0,244	225464_at	FERM domain containing 6	*FRMD6*
18	439	199	2,21	0,000076	0,201	222450_at	Transmembrane, prostate androgen induced RNA	*TMEPAI*
1763	316	143	2,21	0,011236	0,348	209459_s_at	4-aminobutyrate aminotransferase	*ABAT*
134	105	47,9	2,20	0,000589	0,240	203887_s_at	Thrombomodulin	*THBD*
220	399	181	2,20	0,001017	0,250	225481_at	FERM domain containing 6	*FRMD6*
1431	32,4	14,7	2,20	0,008679	0,331	213680_at	Keratin 6B	*KRT6B*
890	287	131	2,20	0,005141	0,313	227811_at	FYVE, RhoGEF and PH domain containing 3	*FGD3*
3541	47,0	21,4	2,20	0,025480	0,393	229659_s_at	Polymeric immunoglobulin receptor	*PIGR*
85	182	83,0	2,19	0,000327	0,201	204446_s_at	Arachidonate 5-lipoxygenase	*ALOX5*
1937	43,7	19,9	2,19	0,012616	0,355	227019_at	Chromosome 1 open reading frame 226	*C1orf226*
4745	12,9	5,89	2,19	0,035948	0,414	213992_at	Collagen, type IV, alpha 6	*COL4A6*
199	14,0	6,40	2,19	0,000924	0,244	243952_at	TPTE pseudogene	*psiTPTE22*
44	49,0	22,4	2,18	0,000181	0,201	211965_at	Zinc finger protein 36, C3H type-like 1	*ZFP36L1*
10	66,8	30,6	2,18	0,000035	0,180	212762_s_at	Transcription factor 7-like 2 (T-cell specific, HMG-box)	*TCF7L2*
343	17,2	7,89	2,18	0,001740	0,277	1558048_x_a	-	-
5803	89,3	41,0	2,18	0,046848	0,441	204864_s_at	Interleukin 6 signal transducer (gp130, oncostatin M receptor)	*IL6ST*
594	54,4	25,0	2,18	0,003271	0,301	1558686_at	cDNA clone YP99D02	-
3682	81,1	37,3	2,17	0,026584	0,395	205542_at	Six transmembrane epithelial antigen of the prostate 1	*STEAP1*
235	53,6	24,7	2,17	0,001089	0,250	210319_x_at	MSH homeobox 2	*MSX2*
732	429	198	2,17	0,004183	0,308	217771_at	Golgi membrane protein 1	*GOLM1*
5833	1106	510	2,17	0,047131	0,442	214440_at	N-acetyltransferase 1 (arylamine N-acetyltransferase)	*NAT1*
7	86,9	40,2	2,16	0,000014	0,099	216511_s_at	Transcription factor 7-like 2 (T-cell specific, HMG-box)	*TCF7L2*
902	93,1	43,0	2,16	0,005222	0,313	213506_at	Coagulation factor II (thrombin) receptor-like 1	*F2RL1*
1696	311	144	2,15	0,010779	0,347	209460_at	4-aminobutyrate aminotransferase	*ABAT*
136	19,3	8,95	2,15	0,000608	0,244	244181_at	Phosphoinositide-3-kinase, regulatory subunit 1 (p85 alpha)	*PIK3R1*
3408	903	419	2,15	0,024308	0,390	214428_x_at	Complement component 4A (Rodgers blood group	*C4A*
3093	164	76,2	2,15	0,021805	0,385	212551_at	CAP, adenylate cyclase-associated protein, 2 (yeast)	*CAP2*
122	47,9	22,3	2,15	0,000566	0,240	219232_s_at	EGL nine homolog 3 (C. elegans)	*EGLN3*
2972	26,5	12,3	2,15	0,020878	0,384	226492_at	Semaphorin 6D	*SEMA6D*
412	63,4	29,6	2,15	0,002172	0,286	240690_at	Homolog of rat pragma of Rnd2	*PRAGMIN*
391	17,8	8,30	2,14	0,002028	0,284	214981_at	Periostin, osteoblast specific factor	*POSTN*
1850	712	334	2,14	0,011902	0,352	222453_at	Cytochrome b reductase 1	*CYBRD1*
258	19,2	8,98	2,13	0,001208	0,256	237839_at	Ankyrin 3, node of Ranvier (ankyrin G)	*ANK3*
441	6,14	2,88	2,13	0,002341	0,288	210738_s_at	Solute carrier family 4, sodium bicarbonate cotransporter, member 4	*SLC4A4*
51	21,0	9,84	2,13	0,000198	0,201	209598_at	Paraneoplastic antigen MA2	*PNMA2*
463	15,4	7,24	2,13	0,002458	0,288	211494_s_at	Solute carrier family 4, sodium bicarbonate cotransporter, member 4	*SLC4A4*
92	251	118	2,13	0,000343	0,204	224975_at	Nuclear factor I/A	*NFIA*
725	83,5	39,3	2,13	0,004146	0,308	221958_s_at	G protein-coupled receptor 177	*GPR177*
1340	316	149	2,12	0,008051	0,328	223344_s_at	Membrane-spanning 4-domains, subfamily A, member 7	*MS4A7*
393	47,6	22,5	2,12	0,002074	0,285	230763_at	Spermatogenesis associated 17	*SPATA17*

Index	Mutated*	Normal*	FC	*P* value	FDR**	Probe set	Description	Gene symbol
**325**	**14,5**	**6,85**	**2,12**	**0,001626**	**0,272**	**240024_at**	**SEC14-like 2 (S. cerevisiae)**	***SEC14L2***
**3988**	**93,1**	**44,0**	**2,12**	**0,029178**	**0,400**	**209706_at**	**NK3 homeobox 1**	***NKX3-1***
**439**	**14,6**	**6,90**	**2,12**	**0,002329**	**0,288**	**204933_s_at**	**Tumor necrosis factor receptor superfamily, member 11b**	***TNFRSF11B***
**205**	**7,77**	**3,67**	**2,12**	**0,000948**	**0,244**	**204932_at**	**Tumor necrosis factor receptor superfamily, member 11b**	***TNFRSF11B***
**4197**	**1672**	**791**	**2,11**	**0,031024**	**0,404**	**208451_s_at**	**Complement component 4A (Rodgers blood group)**	***C4A***
**435**	**207**	**98,1**	**2,11**	**0,002300**	**0,288**	**221584_s_at**	**Calcium-activated potassium channel subunit alpha-1**	***KCNMA1***
**2064**	**192**	**91,6**	**2,10**	**0,013476**	**0,356**	**224996_at**	**Aspartate beta-hydroxylase**	***ASPH***
**5766**	**95,5**	**45,6**	**2,10**	**0,046334**	**0,439**	**204400_at**	**Embryonal Fyn-associated substrate**	***EFS***
**866**	**512**	**245**	**2,09**	**0,005012**	**0,313**	**221666_s_at**	**PYD and CARD domain containing**	***PYCARD***
**5597**	**55,2**	**26,4**	**2,09**	**0,044640**	**0,436**	**206224_at**	**Cystatin SN**	***CST1***
**410**	**17,2**	**8,21**	**2,09**	**0,002158**	**0,286**	**227051_at**	**cDNA clone IMAGE:4393354**	**-**
**2670**	**15,2**	**7,27**	**2,09**	**0,018260**	**0,374**	**221933_at**	**Neuroligin 4, X-linked**	***NLGN4X***
**1077**	**117**	**56,0**	**2,08**	**0,006317**	**0,319**	**215073_s_at**	**Nuclear receptor subfamily 2, group F, member 2**	***NR2F2***
**5**	**121**	**58,3**	**2,08**	**0,000012**	**0,099**	**216035_x_at**	**Transcription factor 7-like 2 (T-cell specific, HMG-box)**	***TCF7L2***
**2491**	**76,8**	**37,0**	**2,08**	**0,016787**	**0,367**	**211695_x_at**	**Mucin 1, cell surface associated**	***MUC1***
**2197**	**58,5**	**28,3**	**2,07**	**0,014525**	**0,361**	**219412_at**	**RAB38, member RAS oncogene family**	***RAB38***
**679**	**28,2**	**13,6**	**2,07**	**0,003899**	**0,308**	**231929_at**	**IKAROS family zinc finger 2 (Helios)**	***IKZF2***
**3566**	**24,6**	**11,9**	**2,07**	**0,025741**	**0,394**	**1564241_at**	**ATPase, Na+/K+ transporting, alpha 4 polypeptide**	***ATP1A4***
**1412**	**42,1**	**20,3**	**2,07**	**0,008532**	**0,330**	**205541_s_at**	**G1 to S phase transition 2**	***GSPT2***
**1543**	**22,6**	**11,0**	**2,06**	**0,009541**	**0,337**	**229510_at**	**Membrane-spanning 4-domains, subfamily A, member 14**	***MS4A14***
**323**	**132**	**64,1**	**2,05**	**0,001612**	**0,272**	**214761_at**	**Zinc finger protein 423**	***ZNF423***
**1226**	**9,02**	**4,39**	**2,05**	**0,007288**	**0,324**	**206522_at**	**Maltase-glucoamylase (alpha-glucosidase)**	***MGAM***
**909**	**66,2**	**32,3**	**2,05**	**0,005249**	**0,313**	**242874_at**	**cDNA FLJ37931 fis, clone CTONG2004397**	**-**
**297**	**46,5**	**22,7**	**2,05**	**0,001451**	**0,263**	**203780_at**	**Myelin protein zero-like 2**	***MPZL2***
**1332**	**27,6**	**13,5**	**2,05**	**0,008004**	**0,328**	**227444_at**	**Armadillo repeat containing, X-linked 4**	***ARMCX4***
**30**	**45,9**	**22,6**	**2,03**	**0,000138**	**0,201**	**222027_at**	**Nuclear casein kinase and cyclin-dependent kinase substrate 1**	***NUCKS1***
**184**	**33,8**	**16,7**	**2,03**	**0,000854**	**0,244**	**232968_at**	**Fibronectin type III and ankyrin repeat domains 1**	***FANK1***
**5840**	**56,5**	**27,9**	**2,03**	**0,047191**	**0,442**	**210222_s_at**	**Reticulon 1**	***RTN1***
**63**	**355**	**175**	**2,02**	**0,000237**	**0,201**	**222449_at**	**Transmembrane, prostate androgen induced RNA**	***TMEPAI***
**271**	**240**	**119**	**2,02**	**0,001298**	**0,261**	**235085_at**	**Homolog of rat pragma of Rnd2**	***PRAGMIN***
**3919**	**16,5**	**8,17**	**2,02**	**0,028625**	**0,399**	**220625_s_at**	**E74-like factor 5 (ets domain transcription factor)**	***ELF5***
**5086**	**68,5**	**33,9**	**2,02**	**0,039151**	**0,420**	**208228_s_at**	**Fibroblast growth factor receptor 2**	***FGFR2***
**130**	**360**	**179**	**2,02**	**0,000581**	**0,240**	**226806_s_at**	**Nuclear factor I/A**	***NFIA***
**2273**	**146**	**72,6**	**2,02**	**0,015097**	**0,363**	**204285_s_at**	**Phorbol-12-myristate-13-acetate-induced protein 1**	***PMAIP1***
**2318**	**75,1**	**37,3**	**2,01**	**0,015395**	**0,363**	**202236_s_at**	**Solute carrier family 16, member 1**	***SLC16A1***
**115**	**222**	**110**	**2,01**	**0,000512**	**0,240**	**212239_at**	**Phosphoinositide-3-kinase, regulatory subunit 1 (p85 alpha)**	***PIK3R1***
**888**	**69,3**	**34,4**	**2,01**	**0,005128**	**0,313**	**204463_s_at**	**Endothelin receptor type A**	***EDNRA***
**34**	**46,6**	**23,2**	**2,01**	**0,000146**	**0,201**	**201340_s_at**	**Ectodermal-neural cortex (with BTB-like domain)**	***ENC1***
**2665**	**17,9**	**8,91**	**2,01**	**0,018221**	**0,374**	**232124_at**	**Family with sequence similarity 198, member A**	***C3orf41***
**842**	**447**	**223**	**2,01**	**0,004825**	**0,313**	**223125_s_at**	**Chromosome 1 open reading frame 21**	***C1orf21***
**4412**	**321**	**160**	**2,01**	**0,032952**	**0,408**	**203060_s_at**	**3'-phosphoadenosine 5'-phosphosulfate synthase 2**	***PAPSS2***
**3319**	**98,2**	**48,9**	**2,01**	**0,023586**	**0,388**	**225123_at**	**Sestrin 3**	***SESN3***
**593**	**128**	**63,9**	**2,00**	**0,003271**	**0,301**	**227514_at**	**Inositol 1,4,5-triphosphate receptor interacting protein-like 2**	***ITPRIPL2***
**3074**	**84,4**	**42,2**	**2,00**	**0,021681**	**0,385**	**230364_at**	**Choline phosphotransferase 1**	***CHPT1***
**864**	**45,6**	**22,8**	**2,00**	**0,004994**	**0,313**	**204363_at**	**Coagulation factor III (thromboplastin, tissue factor)**	***F3***
4951	14,7	7,35	1,99	0,037975	0,419	1552767_a_a	Heparan sulfate 6-O-sulfotransferase 2	*HS6ST2*
785	115	57,8	1,99	0,004493	0,311	212915_at	PDZ domain containing RING finger 3	*PDZRN3*
197	26,3	13,2	1,99	0,000911	0,244	242903_at	Interferon gamma receptor 1	*IFNGR1*
1802	15,1	7,55	1,99	0,011600	0,351	202234_s_at	Solute carrier family 16, member 1	*SLC16A1*
157	44,0	22,1	1,99	0,000708	0,244	222847_s_at	EGL nine homolog 3 (C. elegans)	*EGLN3*
5541	110	55,4	1,99	0,044049	0,435	223278_at	Gap junction protein, beta 2, 26kDa	*GJB2*
148	12,6	6,36	1,99	0,000682	0,244	235952_at	Transcribed locus	-
5819	36,2	18,2	1,98	0,047031	0,442	233814_at	Ephrin-A5	*EFNA5*
336	536	271	1,98	0,001715	0,277	209121_x_at	Nuclear receptor subfamily 2, group F, member 2	*NR2F2*
326	371	188	1,98	0,001629	0,272	230958_s_at	Glyceronephosphate O-acyltransferase	*GNPAT*
1007	51,4	26,0	1,97	0,005834	0,316	230788_at	I beta-1,6-N-acetylglucosaminyltransferase	*GCNT2*
574	40,4	20,5	1,97	0,003154	0,300	1569607_s_a	Ankyrin repeat domain 20 family, member A1	*ANKRD20A1*

Index	Mutated*	Normal*	FC	*P* value	FDR**	Probe set	Description	Gene symbol
206	278	141	1,97	0,000950	0,244	202709_at	Fibromodulin	*FMOD*
327	133	67,8	1,96	0,001630	0,272	226084_at	Microtubule-associated protein 1B	*MAP1B*
4066	254	129	1,96	0,029819	0,401	231766_s_at	Collagen, type XII, alpha 1	*COL12A1*
274	21,8	11,1	1,96	0,001313	0,262	244026_at	Elongation factor, RNA polymerase II, 2	*ELL2*
126	21,6	11,0	1,96	0,000574	0,240	226885_at	Transcribed locus	-
5995	83,6	42,7	1,96	0,048745	0,444	225008_at	Aspartate beta-hydroxylase	*ASPH*
638	162	82,8	1,96	0,003626	0,308	209522_s_at	Carnitine acetyltransferase	*CRAT*
2964	817	418	1,96	0,020824	0,384	209875_s_at	Secreted phosphoprotein 1	*SPP1*
4037	19,6	10,0	1,96	0,029579	0,401	1564358_at	cDNA FLJ22631 fis, clone HSI06451	-
1339	46,5	23,8	1,95	0,008044	0,328	213107_at	TRAF2 and NCK interacting kinase	*TNIK*
67	190	97,1	1,95	0,000252	0,201	224976_at	Nuclear factor I/A	*NFIA*
2711	13,9	7,09	1,95	0,018609	0,375	205044_at	Gamma-aminobutyric acid (GABA) A receptor, pi	*GABRP*
4813	273	140	1,95	0,036577	0,416	203917_at	Coxsackie virus and adenovirus receptor	*CXADR*
211	44,3	22,7	1,95	0,000957	0,244	203888_at	Thrombomodulin	*THBD*
756	160	82,1	1,94	0,004275	0,308	225379_at	Microtubule-associated protein tau	*MAPT*
706	46,6	24,0	1,94	0,004054	0,308	227082_at	cDNA DKFZp586K1922	-
3238	708	365	1,94	0,022982	0,388	212196_at	Interleukin 6 signal transducer (oncostatin M receptor)	*IL6ST*
3165	45,8	23,6	1,94	0,022459	0,387	228221_at	Solute carrier family 44, member 3	*SLC44A3*
4851	11,6	6,01	1,94	0,036961	0,417	204591_at	Cell adhesion molecule L1-like	*CHL1*
1765	23,4	12,1	1,94	0,011260	0,348	205157_s_at	Keratin 17	*KRT17*
162	408	211	1,93	0,000746	0,244	224970_at	Nuclear factor I/A	*NFIA*
556	22,1	11,4	1,93	0,003024	0,296	239768_x_at	G patch domain containing 2	*GPATCH2*
1280	63,7	33,0	1,93	0,007616	0,325	1556126_s_a	G patch domain containing 2	*GPATCH2*
353	49,4	25,6	1,93	0,001807	0,279	233442_at	PDZ domain containing ring finger 3	*PDZRN3*
4192	135	70,0	1,93	0,030967	0,404	212554_at	CAP, adenylate cyclase-associated protein, 2 (yeast)	*CAP2*
654	826	428	1,93	0,003769	0,308	228297_at	Calponin 3, acidic	*CNN3*
418	59,2	30,7	1,93	0,002193	0,286	214807_at	cDNA DKFZp564O0862	-
2379	63,4	32,9	1,93	0,015858	0,364	232113_at	Hypothetical protein LOC399959	*LOC399959*
193	43,0	22,3	1,93	0,000899	0,244	210021_s_at	Cyclin O	*CCNO*
537	12,0	6,22	1,93	0,002890	0,293	1569208_a_a	LIM and calponin homology domains 1	*LIMCH1*
1674	16,5	8,56	1,92	0,010559	0,345	219250_s_at	Fibronectin leucine rich transmembrane protein 3	*FLRT3*
17	364	190	1,92	0,000068	0,201	212761_at	Transcription factor 7-like 2 (T-cell specific, HMG-box)	*TCF7L2*
1044	38,3	20,0	1,92	0,006098	0,319	228949_at	G protein-coupled receptor 177	*GPR177*
3550	157	81,8	1,92	0,025564	0,394	221841_s_at	Kruppel-like factor 4 (gut)	*KLF4*
1792	8,09	4,23	1,91	0,011482	0,350	1569344_a_a	cDNA clone IMAGE:4044872	-
3614	6,01	3,16	1,90	0,026086	0,395	214601_at	Tryptophan hydroxylase 1 (tryptophan 5-monooxygenase)	*TPH1*
1028	61,6	32,4	1,90	0,005949	0,316	215856_at	Sialic acid binding Ig-like lectin 15	*SIGLEC15*
3961	101	53,5	1,90	0,028941	0,399	224221_s_at	Vav 3 guanine nucleotide exchange factor	*VAV3*
1467	48,6	25,7	1,90	0,008976	0,334	206483_at	Leucine rich repeat containing 6	*LRRC6*
68	66,6	35,2	1,90	0,000259	0,201	242829_x_at	F-box and leucine-rich repeat protein 3	*FBXL3*
245	55,1	29,1	1,89	0,001128	0,251	243963_at	Serologically defined colon cancer antigen 8	*SDCCAG8*
3417	65,9	34,8	1,89	0,024406	0,391	205948_at	Protein tyrosine phosphatase, receptor type, T	*PTPRT*
1418	22,2	11,7	1,89	0,008595	0,331	219890_at	C-type lectin domain family 5, member A	*CLEC5A*
5018	530	280	1,89	0,038593	0,420	219087_at	Asporin	*ASPN*
139	42,2	22,4	1,89	0,000631	0,244	239392_s_at	Pogo transposable element with KRAB domain	*POGK*
1447	80,2	42,5	1,89	0,008803	0,333	222529_at	Solute carrier family 25, member 37	*SLC25A37*
2680	128	68,0	1,88	0,018347	0,374	223126_s_at	Chromosome 1 open reading frame 21	*C1orf21*
36	36,6	19,5	1,88	0,000149	0,201	239476_at	Phosphoinositide-3-kinase, regulatory subunit 1 (p85 alpha)	*PIK3R1*
2551	11,5	6,13	1,88	0,017269	0,370	237183_at	Polypeptide N-acetylgalactosaminyltransferase 5	*GALNT5*
1443	21,7	11,6	1,87	0,008776	0,332	1554712_a_a	Glycine-N-acyltransferase-like 2	*GLYATL2*
4613	2309	1236	1,87	0,034825	0,412	202286_s_at	Tumor-associated calcium signal transducer 2	*TACSTD2*
716	81,8	43,9	1,86	0,004115	0,308	226985_at	FYVE, RhoGEF and PH domain containing 5	*FGD5*
3035	53,9	29,0	1,86	0,021394	0,385	209493_at	PDZ domain containing 2	*PDZD2*
1284	65,4	35,1	1,86	0,007646	0,325	241769_at	Vitronectin receptor subunit alpha (antigen CD51)	*ITGAV*
1213	73,7	39,6	1,86	0,007201	0,324	226909_at	KIAA1729 protein	*KIAA1729*
11	115	61,9	1,86	0,000037	0,180	216037_x_at	Transcription factor 7-like 2 (T-cell specific, HMG-box)	*TCF7L2*
123	8,86	4,78	1,86	0,000566	0,240	240361_at	-	-

Index	Mutated*	Normal*	FC	*P* value	FDR**	Probe set	Description	Gene symbol
646	49,9	26,9	1,85	0,003673	0,308	1557527_at	Runt-related transcription factor 1 (aml1 oncogene)	*RUNX1*
2659	52,9	28,6	1,85	0,018154	0,373	243729_at	cDNA FLJ37931 fis, clone CTONG2004397	-
886	78,4	42,4	1,85	0,005117	0,313	212736_at	Chromosome 16 open reading frame 45	*C16orf45*
3172	333	180	1,85	0,022504	0,387	232615_at	Phosphodiesterase 4D interacting protein	*PDE4DIP*
5474	99,5	53,9	1,85	0,043272	0,432	204286_s_at	Phorbol-12-myristate-13-acetate-induced protein 1	*PMAIP1*
128	99,8	54,1	1,85	0,000575	0,240	225265_at	RNA binding motif, single stranded interacting protein 1	*RBMS1*
4993	107	58,1	1,85	0,038307	0,419	205613_at	Synaptotagmin XVII	*SYT17*
1130	14,0	7,62	1,84	0,006590	0,319	220428_at	CD207 molecule, langerin	*CD207*
3771	59,7	32,4	1,84	0,027388	0,397	227812_at	Tumor necrosis factor receptor superfamily, member 19	*TNFRSF19*
742	390	212	1,84	0,004216	0,308	209120_at	Nuclear receptor subfamily 2, group F, member 2	*NR2F2*
4224	126	68,4	1,84	0,031311	0,405	202986_at	Aryl-hydrocarbon receptor nuclear translocator 2	*ARNT2*
96	13,4	7,31	1,84	0,000374	0,212	232125_at	cDNA FLJ12166 fis, clone MAMMA1000616	-
54	266	145	1,84	0,000212	0,201	212614_at	AT rich interactive domain 5B (MRF1-like)	*ARID5B*
2905	908	495	1,83	0,020424	0,383	217763_s_at	RAB31, member RAS oncogene family	*RAB31*
2615	15,8	8,59	1,83	0,017764	0,371	232459_at	Cytochrome b reductase 1	*CYBRD1*
627	35,4	19,3	1,83	0,003559	0,308	240038_at	Elongation factor, RNA polymerase II, 2	*ELL2*
2476	147	80,4	1,83	0,016668	0,367	1553132_a_a	Tandem C2 domains, nuclear	*TC2N*
880	129	70,5	1,83	0,005093	0,313	223519_at	Sterile alpha motif and leucine zipper containing kinase AZK	*ZAK*
380	49,6	27,2	1,83	0,001979	0,284	202150_s_at	Neural precursor cell expressed, developmentally down-regulated 9	*NEDD9*
1414	73,7	40,4	1,83	0,008557	0,331	238447_at	RNA binding motif, single stranded interacting protein	*RBMS3*
1583	16,2	8,86	1,82	0,009862	0,340	39549_at	Neuronal PAS domain protein 2	*NPAS2*
1906	5,29	2,90	1,82	0,012388	0,355	223810_at	Kelch-like 1 (Drosophila)	*KLHL1*
4915	34,1	18,7	1,82	0,037625	0,418	235629_at	Fibronectin 1	*FN1*
1388	19,6	10,7	1,82	0,008374	0,330	211734_s_at	FC fragment of IgE, high affinity I, receptor for; alpha polypeptide	*FCER1A*
950	123	67,7	1,82	0,005442	0,313	219572_at	Ca2+-dependent activator protein for secretion 2	*CADPS2*
3300	100	55,1	1,82	0,023506	0,388	228256_s_at	Erythrocyte membrane protein band 4.1 like 4A	*EPB41L4A*
1726	11,3	6,23	1,82	0,011045	0,348	1566764_at	Metastasis associated in colon cancer 1	*MACC1*
917	144	78,9	1,82	0,005282	0,313	227224_at	Ral GEF with PH domain and SH3 binding motif 2	*RALGPS2*
175	54,7	30,1	1,82	0,000803	0,244	219771_at	TBC1 domain family, member 8B (with GRAM domain)	*TBC1D8B*
499	55,8	30,7	1,82	0,002656	0,289	222075_s_at	Ornithine decarboxylase antizyme 3	*OAZ3*
825	55,9	30,8	1,81	0,004708	0,312	222549_at	Claudin 1	*CLDN1*
1651	35,5	19,6	1,81	0,010394	0,344	232151_at	Metastasis associated in colon cancer 1	*MACC1*
5915	147	81,3	1,81	0,047945	0,443	229872_s_at	Hypothetical protein LOC730257	*LOC730257*
400	30,9	17,0	1,81	0,002090	0,285	233223_at	Neural precursor cell expressed, developmentally down-regulated 9	*NEDD9*
1708	51,8	28,7	1,81	0,010916	0,348	228950_s_at	G protein-coupled receptor 177	*GPR177*
1308	68,0	37,7	1,81	0,007787	0,325	203518_at	Lysosomal trafficking regulator	*LYST*
341	140	77,3	1,80	0,001733	0,277	229943_at	Tripartite motif-containing 13	*TRIM13*
511	171	94,7	1,80	0,002714	0,290	1558080_s_a	DnaJ (Hsp40) homolog, subfamily C, member 3	*DNAJC3*
291	241	134	1,80	0,001423	0,263	231897_at	Leukotriene B4 12-hydroxydehydrogenase	*LTB4DH*
1541	44,6	24,7	1,80	0,009536	0,337	204735_at	Phosphodiesterase 4A, cAMP-specific	*PDE4A*
3117	63,0	35,0	1,80	0,022048	0,387	235733_at	Transcribed locus	-
449	49,0	27,3	1,80	0,002394	0,288	226439_s_at	Neurobeachin	*NBEA*
2159	36,1	20,1	1,80	0,014225	0,360	238455_at	Plexin domain containing 2	*PLXDC2*
4482	46,8	26,1	1,80	0,033716	0,411	201820_at	Keratin 5	*KRT5*
62	31,1	17,3	1,80	0,000228	0,201	212649_at	DEAH (Asp-Glu-Ala-His) box polypeptide 29	*DHX29*
3451	6,99	3,89	1,79	0,024701	0,391	210576_at	Cytochrome P450, family 4, subfamily F, polypeptide 8	*CYP4F8*
717	116	64,8	1,79	0,004116	0,308	205011_at	Loss of heterozygosity, 11, chromosomal region 2, gene A	*LOH11CR2A*
191	67,2	37,6	1,79	0,000891	0,244	207836_s_at	RNA binding protein with multiple splicing	*RBPMS*
719	33,2	18,6	1,79	0,004119	0,308	205555_s_at	MSH homeobox 2	*MSX2*
3724	40,0	22,4	1,79	0,027028	0,397	1555407_s_a	FYVE, RhoGEF and PH domain containing 3	*FGD3*
1216	10,0	5,62	1,79	0,007240	0,324	1556190_s_a	Prion protein (p27-30) (Creutzfeldt-Jakob disease)	*PRNP*
273	49,2	27,6	1,78	0,001310	0,262	203542_s_at	Kruppel-like factor 9	*KLF9*
2055	40,5	22,7	1,78	0,013369	0,356	236114_at	Runt-related transcription factor 1 (aml1 oncogene)	*RUNX1*
4358	14,1	7,90	1,78	0,032416	0,407	206091_at	Matrilin 3	*MATN3*
387	147	82,4	1,78	0,002008	0,284	203120_at	Tumor protein p53 binding protein, 2	*TP53BP2*
700	37,5	21,1	1,78	0,004011	0,308	215536_at	Major histocompatibility complex, class II, DQ beta 2	*HLA-DQB2*
968	20,1	11,3	1,78	0,005615	0,315	241872_at	SH3-domain GRB2-like (endophilin) interacting protein 1	*SGIP1*

Index	Mutated*	Normal*	FC	*P* value	FDR**	Probe set	Description	Gene symbol
236	49,2	27,7	1,78	0,001101	0,250	209632_at	Protein phosphatase 2 (formerly 2A), regulatory subunit B'', alpha	*PPP2R3A*
1112	132	74,2	1,78	0,006509	0,319	205097_at	Solute carrier family 26 (sulfate transporter), member 2	*SLC26A2*
1660	35,4	20,0	1,77	0,010480	0,345	206191_at	Ectonucleoside triphosphate diphosphohydrolase 3	*ENTPD3*
1278	52,6	29,7	1,77	0,007609	0,325	209655_s_at	Transmembrane protein 47	*TMEM47*
2770	30,6	17,3	1,77	0,019221	0,379	1558612_a_a	ATPase, Na+/K+ transporting, alpha 4 polypeptide	*ATP1A4*
495	87,7	49,6	1,77	0,002640	0,289	230906_at	N-acetylgalactosaminyltransferase 10	*GALNT10*
4515	49,9	28,2	1,77	0,034036	0,412	218857_s_at	Asparaginase like 1	*ASRGL1*
4569	220	125	1,77	0,034553	0,412	202464_s_at	6-phosphofructo-2-kinase/fructose-2,6-biphosphatase 3	*PFKFB3*
4703	108	60,9	1,77	0,035548	0,413	214295_at	KIAA0485 protein	*KIAA0485*
4151	247	140	1,77	0,030628	0,403	227384_s_at	Hypothetical protein LOC727820	*LOC727820*
1011	413	234	1,76	0,005857	0,316	226085_at	Chromobox homolog 5 (HP1 alpha homolog, Drosophila)	*CBX5*
5489	344	195	1,76	0,043445	0,433	214329_x_at	Tumor necrosis factor (ligand) superfamily, member 10	*TNFSF10*
5094	54,3	30,8	1,76	0,039195	0,420	229744_at	Sperm specific antigen 2	*SSFA2*
871	200	113	1,76	0,005028	0,313	227740_at	U2AF homology motif (UHM) kinase 1	*UHMK1*
1202	5,58	3,17	1,76	0,007111	0,323	1554027_a_a	Solute carrier family 4, sodium bicarbonate cotransporter, member 4	*SLC4A4*
637	124	70,1	1,76	0,003620	0,308	235003_at	U2AF homology motif (UHM) kinase 1	*UHMK1*
358	45,0	25,5	1,76	0,001833	0,280	222314_x_at	Eosinophil granule ontogeny transcript (non-protein coding)	*EGOT*
896	65,7	37,3	1,76	0,005182	0,313	218451_at	CUB domain containing protein 1	*CDCP1*
270	36,4	20,7	1,76	0,001291	0,261	213462_at	Neuronal PAS domain protein 2	*NPAS2*
1516	19,5	11,1	1,76	0,009365	0,337	243546_at	Sestrin 3	*SESN3*
566	72,1	41,1	1,75	0,003093	0,299	213342_at	Yes-associated protein 1, 65kDa	*YAP1*
4981	35,1	20,0	1,75	0,038216	0,419	205907_s_at	Osteomodulin	*OMD*
390	123	70,2	1,75	0,002025	0,284	226065_at	Prickle homolog 1 (Drosophila)	*PRICKLE1*
200	81,2	46,4	1,75	0,000925	0,244	222734_at	Tryptophanyl tRNA synthetase 2, mitochondrial	*WARS2*
1683	22,8	13,0	1,75	0,010637	0,345	242458_at	Ral GEF with PH domain and SH3 binding motif 2	*RALGPS2*
929	36,0	20,6	1,75	0,005334	0,313	207300_s_at	Coagulation factor VII (serum prothrombin conversion accelerator)	*F7*
3019	22,2	12,7	1,75	0,021268	0,385	1564064_a_a	ATPase, Class VI, type 11B	*ATP11B*
1538	31,6	18,1	1,75	0,009523	0,337	235592_at	Elongation factor, RNA polymerase II, 2	*ELL2*
2	635	364	1,75	0,000007	0,099	211962_s_at	Zinc finger protein 36, C3H type-like 1	*ZFP36L1*
1433	182	104	1,75	0,008691	0,331	201730_s_at	Translocated promoter region (to activated MET oncogene)	*TPR*
697	35,5	20,4	1,74	0,004003	0,308	214269_at	Major facilitator superfamily domain containing 7	*MFSD7*
1636	36,5	21,0	1,74	0,010311	0,344	205729_at	Oncostatin M receptor	*OSMR*
5311	64,8	37,2	1,74	0,041436	0,426	235924_at	cDNA FLJ42287 fis, clone TLIVE2005866	-
1245	371	213	1,74	0,007398	0,324	1568619_s_a	Hypothetical protein LOC162073	*LOC162073*
1605	21,7	12,5	1,74	0,010038	0,342	241837_at	AT rich interactive domain 5B (MRF1-like)	*ARID5B*
1807	162	93,3	1,74	0,011634	0,351	234970_at	Tandem C2 domains, nuclear	*TC2N*
2000	60,8	35,0	1,74	0,013064	0,355	232797_at	Vitronectin receptor subunit alpha (antigen CD51)	*ITGAV*
5188	19,7	11,3	1,74	0,039978	0,421	206595_at	Cystatin E/M	*CST6*
2657	36,9	21,2	1,74	0,018124	0,373	229105_at	G protein-coupled receptor 39	*GPR39*
1640	77,8	45,0	1,73	0,010338	0,344	229530_at	Guanylate cyclase 1, soluble, alpha 3	*GUCY1A3*
894	124	72,0	1,73	0,005167	0,313	226621_at	Fibrinogen gamma chain	*FGG*
3129	72,6	42,0	1,73	0,022184	0,387	236179_at	Cadherin 11, type 2, OB-cadherin (osteoblast)	*CDH11*
2351	66,3	38,4	1,73	0,015686	0,364	229307_at	Ankyrin repeat domain 28	*ANKRD28*
1475	24,6	14,3	1,73	0,009014	0,334	241864_x_at	Transcribed locus	-
1653	15,2	8,80	1,72	0,010426	0,345	241310_at	Transcribed locus	-
2025	13,9	8,07	1,72	0,013199	0,355	240120_at	Sorbin and SH3 domain containing 2	*SORBS2*
142	225	130	1,72	0,000645	0,244	218788_s_at	SET and MYND domain containing 3	*SMYD3*
479	16,4	9,54	1,72	0,002563	0,289	213109_at	TRAF2 and NCK interacting kinase	*TNIK*
1915	1281	743	1,72	0,012500	0,355	212195_at	Interleukin 6 signal transducer (gp130, oncostatin M receptor)	*IL6ST*
1965	15,7	9,10	1,72	0,012849	0,355	225660_at	Semaphorin 6A-1	*SEMA6A*
408	154	89,2	1,72	0,002147	0,286	228824_s_at	Prostaglandin reductase 1	*PTGR1*
1826	7,45	4,33	1,72	0,011762	0,351	204913_s_at	SRY (sex determining region Y)-box 11	*SOX11*
1398	63,2	36,7	1,72	0,008450	0,330	228141_at	Glutathione peroxidase 8 (putative)	*GPX8*
300	87,4	50,8	1,72	0,001461	0,263	207749_s_at	Protein phosphatase 2 (formerly 2A), regulatory subunit B'', alpha	*PPP2R3A*
979	33,5	19,5	1,72	0,005682	0,315	235834_at	Caldesmon 1	*CALD1*
528	11,1	6,45	1,72	0,002818	0,292	221583_s_at	Calcium-activated potassium channel subunit alpha-1	*KCNMA1*
3025	20,1	11,7	1,72	0,021294	0,385	235957_at	Glutamate receptor interacting protein 1	*GRIP1*

Index	Mutated*	Normal*	FC	*P* value	FDR**	Probe set	Description	Gene symbol
1495	19,3	11,3	1,72	0,009209	0,336	242736_at	Sorbin and SH3 domain containing 1	*SORBS1*
549	78,7	45,8	1,72	0,002972	0,295	214734_at	Exophilin 5	*EXPH5*
41	18,8	11,0	1,72	0,000172	0,201	228602_at	Sarcoglycan, delta (35kDa dystrophin-associated glycoprotein)	*SGCD*
488	23,7	13,8	1,72	0,002619	0,289	242931_at	LON peptidase N-terminal domain and ring finger 3	*LONRF3*
2585	92,2	53,8	1,71	0,017536	0,371	206994_at	Cystatin S	*CST4*
231	52,2	30,5	1,71	0,001070	0,250	244533_at	Protein tyrosine phosphatase, non-receptor type 14	*PTPN14*
2554	68,4	40,0	1,71	0,017283	0,370	239597_at	Poly(A) specific ribonuclease subunit homolog (S. cerevisiae)	*PAN3*
3485	55,3	32,3	1,71	0,025017	0,392	223948_s_at	Transmembrane protease, serine 3	*TMPRSS3*
2090	23,9	14,0	1,71	0,013626	0,356	235278_at	MACRO domain containing 2	*MACROD2*
3800	76,6	44,8	1,71	0,027683	0,398	217867_x_at	Beta-site APP-cleaving enzyme 2	*BACE2*
2153	14,8	8,68	1,71	0,014179	0,360	230472_at	Iroquois homeobox 1	*IRX1*
2716	68,1	39,9	1,71	0,018663	0,376	243296_at	Pre-B-cell colony enhancing factor 1	*PBEF1*
3543	848	497	1,71	0,025495	0,393	217762_s_at	RAB31, member RAS oncogene family	*RAB31*
3587	65,1	38,2	1,71	0,025885	0,394	204157_s_at	KIAA0999 protein	*KIAA0999*
940	49,7	29,2	1,70	0,005379	0,313	236561_at	Transforming growth factor, beta receptor I	*TGFBR1*
1771	25,1	14,7	1,70	0,011292	0,348	213929_at	Exophilin 5	*EXPH5*
2056	170	100	1,70	0,013390	0,356	227533_at	Transcribed locus	-
2326	65,8	38,7	1,70	0,015473	0,364	232138_at	Muscleblind-like 2 (Drosophila)	*MBNL2*
1513	9,22	5,42	1,70	0,009319	0,337	220117_at	Zinc finger protein 659	*ZNF659*
4051	61,2	36,0	1,70	0,029701	0,401	241897_at	RNA binding protein with multiple splicing	*RBPMS*
4115	299	176	1,70	0,030261	0,402	228993_s_at	Non-protein coding RNA 81	*NCRNA00081*
6074	7,90	4,65	1,70	0,049585	0,446	231626_at	Tryptophan hydroxylase 1	*TPH1*
3370	128	75,4	1,70	0,023966	0,389	227232_at	Enah/Vasp-like	*EVL*
5709	249	147	1,70	0,045713	0,438	202506_at	Sperm specific antigen 2	*SSFA2*
624	108	63,7	1,70	0,003541	0,308	202962_at	Kinesin family member 13B	*KIF13B*
851	59,2	34,9	1,69	0,004897	0,313	242281_at	Glutamate-ammonia ligase (glutamine synthetase)	*GLUL*
4994	15,4	9,09	1,69	0,038316	0,419	205908_s_at	Osteomodulin	*OMD*
198	14,2	8,38	1,69	0,000917	0,244	233286_at	PDZ domain containing ring finger 3	*PDZRN3*
904	82,7	48,9	1,69	0,005233	0,313	204948_s_at	Follistatin	*FST*
1075	90,7	53,7	1,69	0,006305	0,319	201369_s_at	Zinc finger protein 36, C3H type-like 2	*ZFP36L2*
1741	27,2	16,1	1,69	0,011126	0,348	232112_at	Ral GEF with PH domain and SH3 binding motif 2	*RALGPS2*
1509	308	183	1,69	0,009303	0,337	202820_at	Aryl hydrocarbon receptor	*AHR*
1052	322	191	1,69	0,006147	0,319	218694_at	Armadillo repeat containing, X-linked 1	*ARMCX1*
3488	85,4	50,6	1,69	0,025069	0,392	227997_at	Interleukin 17 receptor D	*IL17RD*
2408	7,59	4,50	1,69	0,016095	0,365	220014_at	Proline rich 16	*PRR16*
388	27,0	16,0	1,69	0,002012	0,284	214366_s_at	Arachidonate 5-lipoxygenase	*ALOX5*
2306	669	397	1,69	0,015300	0,363	1555812_a_a	Rho GDP dissociation inhibitor (GDI) beta	*ARHGDIB*
2407	156	92,5	1,69	0,016084	0,365	241879_at	Transcribed locus	-
3050	86,1	51,1	1,68	0,021466	0,385	223349_s_at	BCL2-related ovarian killer	*BOK*
313	51,8	30,8	1,68	0,001577	0,272	213826_s_at	H3 histone, family 3A	*H3F3A*
3936	79,2	47,1	1,68	0,028758	0,399	203642_s_at	COBL-like 1	*COBLL1*
792	19,8	11,7	1,68	0,004531	0,311	239726_at	Ankyrin 3, node of Ranvier (ankyrin G)	*ANK3*
69	85,7	50,9	1,68	0,000266	0,201	221503_s_at	Karyopherin alpha 3 (importin alpha 4)	*KPNA3*
3832	27,0	16,1	1,68	0,028005	0,399	220266_s_at	Kruppel-like factor 4 (gut)	*KLF4*
1100	42,7	25,4	1,68	0,006453	0,319	209442_x_at	Ankyrin 3, node of Ranvier (ankyrin G)	*ANK3*
287	10,3	6,13	1,68	0,001403	0,263	224463_s_at	Chromosome 11 open reading frame 70	*C11orf70*
723	267	159	1,68	0,004143	0,308	224894_at	Yes-associated protein 1, 65kDa	*YAP1*
1675	89,5	53,4	1,68	0,010563	0,345	205107_s_at	Ephrin-A4	*EFNA4*
147	18,0	10,8	1,68	0,000677	0,244	1557242_at	Similar to Fibroblast growth factor receptor 3 precursor (FGFR-3)	*LOC442200*
514	35,0	21,0	1,67	0,002747	0,291	220272_at	Basonuclin 2	*BNC2*
1795	14,2	8,52	1,67	0,011533	0,351	215047_at	Tripartite motif-containing 58	*TRIM58*
2624	523	314	1,67	0,017825	0,371	206116_s_at	Tropomyosin 1 (alpha)	*TPM1*
3012	302	181	1,67	0,021190	0,385	225496_s_at	Synaptotagmin-like 2	*SYTL2*
2856	368	221	1,67	0,020049	0,383	226534_at	KIT ligand	*KITLG*
3004	720	432	1,67	0,021137	0,385	217202_s_at	Glutamate-ammonia ligase (glutamine synthetase)	*GLUL*
4456	40,4	24,3	1,67	0,033478	0,411	209167_at	Glycoprotein M6B	*GPM6B*
2517	13,6	8,20	1,66	0,016975	0,369	230970_at	Slingshot homolog 2 (Drosophila)	*SSH2*

Index	Mutated*	Normal*	FC	*P* value	FDR**	Probe set	Description	Gene symbol
5930	41,1	24,7	1,66	0,048060	0,443	229125_at	Ankyrin repeat domain 38	*ANKRD38*
2552	135	81,1	1,66	0,017275	0,370	236251_at	Vitronectin receptor subunit alpha (antigen CD51)	*ITGAV*
3330	43,5	26,2	1,66	0,023639	0,388	203641_s_at	COBL-like 1	*COBLL1*
3626	40,4	24,3	1,66	0,026198	0,395	226069_at	Prickle homolog 1 (Drosophila)	*PRICKLE1*
1720	17,3	10,4	1,66	0,011019	0,348	236237_at	KIAA1370 protein	*KIAA1370*
916	26,9	16,2	1,66	0,005275	0,313	222735_at	Transmembrane protein 38B	*TMEM38B*
5056	160	96,2	1,66	0,038950	0,420	202990_at	Phosphorylase, glycogen	*PYGL*
4610	334	201	1,66	0,034821	0,412	212560_at	Sortilin-related receptor, L(DLR class) A repeats-containing	*SORL1*
1623	47,7	28,8	1,66	0,010197	0,344	221207_s_at	Neurobeachin	*NBEA*
352	17,8	10,8	1,66	0,001793	0,278	242321_at	cDNA clone ZE03F06	-
427	106	63,7	1,66	0,002256	0,288	222158_s_at	PPPDE peptidase domain containing 1	*PPPDE1*
571	22,1	13,4	1,66	0,003140	0,300	244571_s_at	Tetratricopeptide repeat domain 12	*TTC12*
1783	67,5	40,8	1,66	0,011373	0,349	232369_at	Muscleblind-like 2 (Drosophila)	*MBNL2*
769	247	149	1,65	0,004380	0,311	201911_s_at	FERM, RhoGEF (ARHGEF) and pleckstrin domain protein 1	*FARP1*
1810	17,0	10,3	1,65	0,011676	0,351	206556_at	Clusterin-like 1 (retinal)	*CLUL1*
160	37,3	22,6	1,65	0,000722	0,244	217875_s_at	Transmembrane, prostate androgen induced RNA	*TMEPAI*
4882	18,1	10,9	1,65	0,037333	0,418	233231_at	Methylcrotonoyl-Coenzyme A carboxylase 2 (beta)	*MCCC2*
6022	315	191	1,65	0,049010	0,445	226279_at	Protease, serine, 23	*PRSS23*
5608	12,0	7,29	1,65	0,044721	0,436	243722_at	PYD (pyrin domain) containing 1	*PYDC1*
558	95,9	58,2	1,65	0,003025	0,296	226252_at	cDNA FLJ34585 fis, clone KIDNE2008758	-
5168	18,2	11,1	1,65	0,039820	0,421	208006_at	Forkhead box I1	*FOXI1*
4305	11,9	7,22	1,65	0,032037	0,407	215506_s_at	DIRAS family, GTP-binding RAS-like 3	*DIRAS3*
543	7,96	4,83	1,65	0,002920	0,294	226933_s_at	Inhibitor of DNA binding 4, dominant negative helix-loop-helix protein	*ID4*
2125	33,1	20,1	1,65	0,013990	0,359	243598_at	Glycerol-3-phosphate dehydrogenase 2 (mitochondrial)	*GPD2*
1798	60,3	36,7	1,65	0,011576	0,351	238465_at	Chromosome 5 open reading frame 35	*C5orf35*
539	53,9	32,8	1,64	0,002895	0,293	223530_at	Tudor and KH domain containing	*TDRKH*
1961	49,3	30,0	1,64	0,012816	0,355	201983_s_at	Avian erythroblastic leukemia viral (v-erb-b) oncogene homolog	*EGFR*
207	464	283	1,64	0,000954	0,244	212294_at	Guanine nucleotide binding protein (G protein), gamma 12	*GNG12*
2263	85,4	52,0	1,64	0,015052	0,363	204036_at	Lysophosphatidic acid receptor 1	*EDG2*
1017	174	106	1,64	0,005886	0,316	213158_at	cDNA clone IMAGE:4214654	-
1242	13,5	8,25	1,64	0,007382	0,324	218772_x_at	Transmembrane protein 38B	*TMEM38B*
3350	84,8	51,7	1,64	0,023787	0,388	212236_x_at	Keratin 17	*KRT17*
1546	750	458	1,64	0,009548	0,337	210139_s_at	Peripheral myelin protein 22	*PMP22*
2437	14,3	8,75	1,64	0,016289	0,365	204914_s_at	SRY (sex determining region Y)-box 11	*SOX11*
4191	33,1	20,2	1,64	0,030965	0,404	226676_at	Zinc finger protein 521	*ZNF521*
3909	96,6	59,1	1,64	0,028531	0,399	202932_at	v-yes-1 Yamaguchi sarcoma viral oncogene homolog 1	*YES1*
4056	50,9	31,1	1,63	0,029751	0,401	238478_at	Basonuclin 2	*BNC2*
3396	11,0	6,76	1,63	0,024222	0,390	220475_at	Solute carrier family 28 member 3	*SLC28A3*
1727	29,9	18,3	1,63	0,011046	0,348	230730_at	Sarcoglycan, delta (35kDa dystrophin-associated glycoprotein)	*SGCD*
498	5,79	3,54	1,63	0,002651	0,289	237134_at	Transcribed locus	-
1194	18,6	11,4	1,63	0,007041	0,322	203930_s_at	Microtubule-associated protein tau	*MAPT*
4495	81,3	49,8	1,63	0,033807	0,411	213135_at	T-cell lymphoma invasion and metastasis 1	*TIAM1*
1473	10,4	6,39	1,63	0,008999	0,334	241762_at	F-box protein 32	*FBXO32*
242	9,93	6,09	1,63	0,001111	0,250	232116_at	Grainyhead-like 3 (Drosophila)	*GRHL3*
4067	53,6	32,9	1,63	0,029824	0,401	230722_at	Basonuclin 2	*BNC2*
5604	93,4	57,3	1,63	0,044711	0,436	211368_s_at	Caspase 1, apoptosis-related cysteine peptidase	*CASP1*
3349	81,3	49,9	1,63	0,023785	0,388	230712_at	Neuroblastoma breakpoint family, member 1	*NBPF1*
2503	13,1	8,04	1,63	0,016843	0,367	209863_s_at	Tumor protein p63	*TP63*
1842	14,5	8,87	1,63	0,011844	0,351	214321_at	Nephroblastoma overexpressed gene	*NOV*
2879	180	111	1,63	0,020209	0,383	209030_s_at	Cell adhesion molecule 1	*CADM1*
3054	137	83,8	1,63	0,021510	0,385	222446_s_at	Beta-site APP-cleaving enzyme 2	*BACE2*
959	30,0	18,4	1,63	0,005539	0,315	241359_at	cDNA FLJ20031 fis, clone ADSU02180	-
4483	44,4	27,3	1,63	0,033718	0,411	202672_s_at	Activating transcription factor 3	*ATF3*
1046	461	283	1,63	0,006113	0,319	227856_at	Chromosome 4 open reading frame 32	*C4orf32*
2988	11,7	7,19	1,63	0,021007	0,384	202859_x_at	Interleukin 8	*IL8*
448	716	440	1,63	0,002392	0,288	201368_at	Zinc finger protein 36, C3H type-like 2	*ZFP36L2*
278	164	101	1,63	0,001357	0,263	226282_at	cDNA clone ZE03F06	-

Index	Mutated*	Normal*	FC	*P* value	FDR**	Probe set	Description	Gene symbol
640	54,8	33,7	1,63	0,003639	0,308	202191_s_at	Growth arrest-specific 7	*GAS7*
1394	1346	827	1,63	0,008433	0,330	208683_at	Calpain 2, (m/II) large subunit	*CAPN2*
1754	388	239	1,63	0,011177	0,348	203509_at	Sortilin-related receptor, L(DLR class) A repeats-containing	*SORL1*
75	116	71,2	1,63	0,000291	0,201	219625_s_at	Collagen, type IV, alpha 3 (Goodpasture antigen) binding protein	*COL4A3BP*
57	21,5	13,2	1,63	0,000216	0,201	214212_x_at	Pleckstrin homology domain containing, family C member 1	*PLEKHC1*
4292	560	345	1,62	0,031900	0,406	227404_s_at	Early growth response 1	*EGR1*
3754	132	81,4	1,62	0,027290	0,397	227628_at	Glutathione peroxidase 8 (putative)	*GPX8*
1577	41,5	25,5	1,62	0,009798	0,340	203543_s_at	Kruppel-like factor 9	*KLF9*
1517	97,6	60,2	1,62	0,009366	0,337	212382_at	Transcription factor 4	*TCF4*
3851	130	80,6	1,62	0,028109	0,399	239246_at	FERM, RhoGEF (ARHGEF) and pleckstrin domain protein 1	*FARP1*
3194	98,0	60,6	1,62	0,022634	0,387	242343_x_at	Zinc finger protein 518A	*ZNF518*
1032	11,1	6,89	1,62	0,005999	0,318	222320_at	Hyperparathyroidism 2 protein	*CDC73*
5328	6,75	4,17	1,62	0,041669	0,428	235831_at	Transcribed locus	-
1109	27,1	16,8	1,62	0,006492	0,319	238909_at	S100 calcium binding protein A10	*S100A10*
319	118	73,2	1,62	0,001596	0,272	214724_at	DIX domain containing 1	*DIXDC1*
5542	130	80,3	1,62	0,044061	0,435	209031_at	Cell adhesion molecule 1	*CADM1*
6046	190	118	1,62	0,049264	0,445	213350_at	Ribosomal protein S11	*RPS11*
480	62,5	38,7	1,61	0,002568	0,289	221042_s_at	Calmin (calponin-like, transmembrane)	*CLMN*
3160	14,2	8,82	1,61	0,022427	0,387	242396_at	Hypothetical protein LOC644192	*LOC644192*
4465	458	284	1,61	0,033537	0,411	232914_s_at	Synaptotagmin-like 2	*SYTL2*
4748	48,0	29,7	1,61	0,035988	0,414	213790_at	ADAM metallopeptidase domain 12	*ADAM12*
3516	178	110	1,61	0,025248	0,392	235501_at	FERM, RhoGEF (ARHGEF) and pleckstrin domain protein 1	*FARP1*
5609	26,4	16,4	1,61	0,044732	0,436	215483_at	A kinase (PRKA) anchor protein (yotiao) 9	*AKAP9*
288	72,6	45,0	1,61	0,001412	0,263	225041_at	M-phase phosphoprotein, mpp8	*HSMPP8*
4729	18,0	11,2	1,61	0,035816	0,414	233109_at	Collagen, type XII, alpha 1	*COL12A1*
5028	296	184	1,61	0,038663	0,420	218806_s_at	Vav 3 guanine nucleotide exchange factor	*VAV3*
2581	70,6	43,9	1,61	0,017505	0,371	213156_at	cDNA clone IMAGE:4214654	-
3269	23,9	14,9	1,61	0,023248	0,388	212177_at	Splicing factor, arginine/serine-rich 18	*SFRS18*
4322	36,4	22,6	1,61	0,032177	0,407	236225_at	Gamma-glutamyltransferase 6 homolog (rat)	*GGT6*
181	1203	748	1,61	0,000838	0,244	208949_s_at	Lectin, galactoside-binding, soluble, 3	*LGALS3*
5636	129	80,0	1,61	0,044981	0,436	214927_at	Integrin, beta-like 1 (with EGF-like repeat domains)	*ITGBL1*
1896	229	143	1,61	0,012326	0,355	228188_at	FOS-like antigen 2	*FOSL2*
3270	53,1	33,1	1,61	0,023250	0,388	220940_at	KIAA1641 protein	*KIAA1641*
5432	117	72,8	1,60	0,042790	0,431	209656_s_at	Transmembrane protein 47	*TMEM47*
5826	80,2	50,0	1,60	0,047075	0,442	229704_at	Androgen-induced proliferation inhibitor	*PDS5B*
691	10,4	6,48	1,60	0,003971	0,308	238067_at	TBC1 domain family, member 8B (with GRAM domain)	*TBC1D8B*
3406	140	87,0	1,60	0,024301	0,390	221824_s_at	Membrane-associated ring finger (C3HC4) 8	*MARCH8*
520	121	75,8	1,60	0,002767	0,291	209633_at	Protein phosphatase 2 (formerly 2A), regulatory subunit B'', alpha	*PPP2R3A*
103	28,7	17,9	1,60	0,000413	0,217	230518_at	Myelin protein zero-like 2	*MPZL2*
214	273	170	1,60	0,000961	0,244	219221_at	Zinc finger and BTB domain containing 38	*ZBTB38*
2322	127	79,2	1,60	0,015439	0,364	222746_s_at	B-box and SPRY domain containing	*BSPRY*
584	187	117	1,60	0,003220	0,301	206061_s_at	Dicer1, Dcr-1 homolog (Drosophila)	*DICER1*
2119	24,9	15,6	1,60	0,013906	0,359	237456_at	RING1 and YY1 binding protein	*RYBP*
395	35,6	22,3	1,60	0,002084	0,285	219469_at	Dynein, cytoplasmic 2, heavy chain 1	*DYNC2H1*
4428	28,1	17,6	1,60	0,033159	0,409	239587_at	Transcribed locus	-
5232	184	115	1,60	0,040493	0,423	200824_at	Glutathione S-transferase pi	*GSTP1*
4845	23,5	14,8	1,59	0,036920	0,417	236587_at	Transcribed locus	-
971	547	343	1,59	0,005626	0,315	212386_at	Transcription factor 4	*TCF4*
1702	159	100,0	1,59	0,010844	0,348	226117_at	TRAF-interacting protein with a forkhead-associated domain	*TIFA*
1881	10,9	6,88	1,59	0,012201	0,355	210739_x_at	Solute carrier family 4, sodium bicarbonate cotransporter, member 4	*SLC4A4*
1464	114	71,4	1,59	0,008959	0,334	229499_at	Calpain 13	*CAPN13*
5622	504	317	1,59	0,044890	0,436	228708_at	RAB27B, member RAS oncogene family	*RAB27B*
529	33,9	21,3	1,59	0,002827	0,292	213239_at	Family with sequence similarity 162, member A	*FAM162A*
2654	94,3	59,3	1,59	0,018094	0,373	212233_at	Microtubule-associated protein 1B	*MAP1B*
5716	32,4	20,4	1,59	0,045772	0,438	206271_at	Toll-like receptor 3	*TLR3*
3487	53,9	33,9	1,59	0,025032	0,392	220751_s_at	Chromosome 5 open reading frame 4	*C5orf4*
279	972	612	1,59	0,001364	0,263	210835_s_at	C-terminal binding protein 2	*CTBP2*

Index	Mutated*	Normal*	FC	*P* value	FDR**	Probe set	Description	Gene symbol
3688	152	95,6	1,59	0,026634	0,395	202933_s_at	v-yes-1 Yamaguchi sarcoma viral oncogene homolog 1	*YES1*
4949	100	63,3	1,59	0,037968	0,419	226225_at	Mutated in colorectal cancers	*MCC*
1397	175	111	1,59	0,008437	0,330	218792_s_at	B-box and SPRY domain containing	*BSPRY*
1914	169	107	1,59	0,012472	0,355	221942_s_at	Guanylate cyclase 1, soluble, alpha 3	*GUCY1A3*
3777	44,8	28,2	1,59	0,027458	0,397	218273_s_at	Protein phosphatase 2C, magnesium-dependent, catalytic subunit	*PPM2C*
567	51,8	32,6	1,59	0,003099	0,299	241233_x_at	Chromosome 21 open reading frame 81	*C21orf81*
4540	26,3	16,6	1,59	0,034300	0,412	210538_s_at	Baculoviral IAP repeat-containing 3	*BIRC3*
1851	10,3	6,49	1,59	0,011917	0,352	233819_s_at	Zinc finger protein 294	*ZNF294*
149	48,4	30,6	1,58	0,000689	0,244	1554106_at	Neurobeachin-like 1	*ALS2CR16*
5389	47,6	30,0	1,58	0,042253	0,429	242920_at	SIK family kinase 3	*SIK3*
1205	53,7	33,9	1,58	0,007132	0,324	214149_s_at	ATPase, H+ transporting, lysosomal 9kDa, V0 subunit e1	*ATP6V0E1*
368	22,8	14,4	1,58	0,001918	0,284	230071_at	Septin 11	*SEPT11*
80	11,4	7,21	1,58	0,000301	0,201	231149_s_at	Unc-51-like kinase 4 (C. elegans)	*ULK4*
961	232	147	1,58	0,005559	0,315	224895_at	Yes-associated protein 1, 65kDa	*YAP1*
3087	489	309	1,58	0,021766	0,385	202336_s_at	Peptidylglycine alpha-amidating monooxygenase	*PAM*
2616	9,71	6,14	1,58	0,017773	0,371	206548_at	Hypothetical protein FLJ23556	*FKJ23556*
426	70,3	44,4	1,58	0,002253	0,288	233540_s_at	CDK5 regulatory subunit associated protein 2	*CDK5RAP2*
1493	66,3	41,9	1,58	0,009199	0,336	222834_s_at	Guanine nucleotide binding protein (G protein), gamma 12	*GNG12*
2438	300	189	1,58	0,016292	0,365	228153_at	Ring finger 144B	*RNF144B*
3034	28,8	18,2	1,58	0,021381	0,385	209318_x_at	Pleiomorphic adenoma gene-like 1	*PLAGL1*
5283	85,1	53,8	1,58	0,041092	0,425	225806_at	Jub, ajuba homolog (Xenopus laevis)	*JUB*
2570	10,7	6,78	1,58	0,017428	0,371	215686_x_at	Transcription factor AP-2 beta	*TFAP2B*
2936	20,4	12,9	1,58	0,020591	0,383	240146_at	Capping protein (actin filament) muscle Z-line, alpha 2	*CAPZA2*
4946	38,9	24,6	1,58	0,037943	0,419	244246_at	Mirror-image polydactyly 1	*MIPOL1*
3365	69,1	43,7	1,58	0,023903	0,388	237064_x_at	Dynein, axonemal, heavy chain 14	*DNAH14*
2219	849	537	1,58	0,014710	0,362	227792_at	Inositol 1,4,5-triphosphate receptor interacting protein-like 2	*ITPRIPL2*
754	149	94,1	1,58	0,004270	0,308	212256_at	N-acetylgalactosaminyltransferase 10	*GALNT10*
1020	71,2	45,1	1,58	0,005898	0,316	241722_x_at	Transcribed locus	-
370	88,4	56,0	1,58	0,001941	0,284	225957_at	Adult retina protein	*LOC153222*
962	253	160	1,58	0,005570	0,315	204373_s_at	Centrosomal protein 350kDa	*CEP350*
4808	30,2	19,1	1,58	0,036508	0,415	222528_s_at	Solute carrier family 25, member 37	*SLC25A37*
4102	30,7	19,5	1,58	0,030147	0,402	229817_at	Zinc finger protein 608	*ZNF608*
1710	27,6	17,5	1,58	0,010940	0,348	239393_at	Poly(A) specific ribonuclease subunit homolog (S. cerevisiae)	*PAN3*
2784	10,3	6,50	1,58	0,019340	0,380	1552405_at	NLR family, pyrin domain containing 5	*NLRP5*
1835	29,5	18,7	1,58	0,011814	0,351	239619_at	Zinc finger protein 395	*ZNF395*
2590	16,7	10,6	1,58	0,017573	0,371	218610_s_at	Hypothetical protein FLJ11151	*FLJ11151*
2114	49,8	31,6	1,58	0,013870	0,359	221276_s_at	Syncoilin, intermediate filament 1	*SYNC1*
2447	10,5	6,66	1,58	0,016391	0,366	202036_s_at	Secreted frizzled-related protein 1	*SFRP1*
3707	28,7	18,2	1,57	0,026801	0,395	236297_at	cDNA FLJ45742 fis, clone KIDNE2016327	-
4440	8,42	5,35	1,57	0,033335	0,410	202235_at	Solute carrier family 16, member 1	*SLC16A1*
2324	57,4	36,5	1,57	0,015461	0,364	233924_s_at	Exocyst complex component 6	*EXOC6*
1733	75,6	48,0	1,57	0,011083	0,348	215338_s_at	Natural killer-tumor recognition sequence	*NKTR*
2631	32,4	20,6	1,57	0,017907	0,372	243395_at	CD55 molecule, decay accelerating factor for complement	*CD55*
5002	27,9	17,7	1,57	0,038404	0,420	244075_at	Hydroxysteroid (17-beta) dehydrogenase 7 pseudogene 2	*HSD17B7P2*
1673	16,5	10,5	1,57	0,010558	0,345	243233_at	Poly(A) specific ribonuclease subunit homolog (S. cerevisiae)	*PAN3*
4275	87,0	55,3	1,57	0,031776	0,406	218344_s_at	REST corepressor 3	*RCOR3*
565	418	266	1,57	0,003091	0,299	202822_at	LIM domain containing preferred translocation partner in lipoma	*LPP*
5220	35,3	22,5	1,57	0,040360	0,423	227816_at	Netrin 1	*NTN1*
4090	24,7	15,8	1,57	0,030069	0,402	243704_at	G patch domain containing 2	*GPATCH2*
2161	41,2	26,2	1,57	0,014281	0,361	207565_s_at	Major histocompatibility complex, class I-related	*MR1*
1951	25,4	16,2	1,57	0,012736	0,355	239296_at	Zinc finger homeobox 1b	*ZFHX1B*
229	11,0	6,99	1,57	0,001058	0,250	238303_at	Oligosaccharyl transferase subunit STT3B	*STT3B*
1747	31,9	20,3	1,57	0,011154	0,348	206527_at	4-aminobutyrate aminotransferase	*ABAT*
2769	54,5	34,8	1,57	0,019219	0,379	238520_at	Transcriptional regulating factor 1	*TRERF1*
2344	31,9	20,3	1,57	0,015645	0,364	1556277_a_a	PAP associated domain containing 4	*PAPD4*
457	11,6	7,43	1,57	0,002445	0,288	240016_at	SUMO1/sentrin specific peptidase 6	*SENP6*
81	45,3	28,9	1,57	0,000306	0,201	222239_s_at	Integrator complex subunit 6	*INTS6*

Index	Mutated*	Normal*	FC	*P* value	FDR**	Probe set	Description	Gene symbol
3523	137	87,2	1,57	0,025286	0,392	226865_at	cDNA DKFZp564O0862	-
809	48,5	30,9	1,57	0,004615	0,311	228315_at	cDNA clone IMAGE:5261213	-
1594	97,5	62,2	1,57	0,009923	0,340	238706_at	PAP associated domain containing 4	*PAPD4*
781	68,0	43,5	1,57	0,004474	0,311	223412_at	Kelch repeat and BTB (POZ) domain containing 7	*KBTBD7*
1457	37,7	24,1	1,57	0,008909	0,334	1558105_a_a	Similar to LOC169932, clone IMAGE:4499203	-
247	28,3	18,1	1,57	0,001140	0,251	223465_at	Collagen, type IV, alpha 3 (Goodpasture antigen) binding protein	*COL4A3BP*
3772	21,4	13,7	1,56	0,027401	0,397	207417_s_at	Zinc finger protein 177	*ZNF177*
109	44,5	28,5	1,56	0,000439	0,220	219278_at	Mitogen-activated protein kinase kinase kinase 6	*MAP3K6*
2032	33,1	21,2	1,56	0,013238	0,355	230708_at	Prickle homolog 1 (Drosophila)	*PRICKLE1*
1049	1657	1060	1,56	0,006138	0,319	210986_s_at	tropomyosin 1 (alpha)	*TPM1*
5310	228	146	1,56	0,041427	0,426	211423_s_at	C-5 sterol desaturase	*SC5DL*
1604	242	155	1,56	0,009995	0,341	202555_s_at	Myosin, light chain kinase	*MYLK*
3552	8,49	5,44	1,56	0,025614	0,394	1568574_x_a	Secreted phosphoprotein 1	*SPP1*
4760	82,7	53,0	1,56	0,036079	0,414	235103_at	Mannosidase, alpha, class 2A, member 1	*MAN2A1*
1512	25,3	16,2	1,56	0,009313	0,337	215314_at	Ankyrin 3, node of Ranvier (ankyrin G)	*ANK3*
344	33,2	21,3	1,56	0,001763	0,278	203263_s_at	Cdc42 guanine nucleotide exchange factor (GEF) 9	*ARHGEF9*
3979	16,0	10,2	1,56	0,029089	0,400	237128_at	Hypothetical protein FLJ20184	*LOC54848*
2991	55,6	35,6	1,56	0,021037	0,385	209209_s_at	Fermitin family homolog 2 (Drosophila)	*FERMT2*
4185	629	403	1,56	0,030945	0,404	201311_s_at	SH3 domain binding glutamic acid-rich protein like	*SH3BGRL*
1788	118	75,6	1,56	0,011446	0,350	224933_s_at	Jumonji domain containing 1C	*JMJD1C*
2887	17,6	11,3	1,56	0,020249	0,383	1563209_a_a	MACRO domain containing 2	*MACROD2*
119	117	75,2	1,56	0,000531	0,240	218819_at	Integrator complex subunit 6	*INTS6*
5040	27,6	17,7	1,56	0,038849	0,420	228427_at	F-box protein 16	*FBXO16*
4673	392	251	1,56	0,035294	0,413	225927_at	Mitogen-activated protein kinase kinase kinase 1	*MAP3K1*
1567	20,8	13,3	1,56	0,009741	0,340	230892_at	Transcribed locus	-
675	235	151	1,56	0,003866	0,308	214352_s_at	v-Ki-ras2 Kirsten rat sarcoma viral oncogene homolog	*KRAS*
1790	25,9	16,6	1,56	0,011463	0,350	230424_at	Chromosome 5 open reading frame 13	*C5orf13*
334	398	256	1,56	0,001676	0,274	209360_s_at	Runt-related transcription factor 1 (aml1 oncogene)	*RUNX1*
316	18,1	11,6	1,56	0,001588	0,272	1559957_a_a	Hypothetical protein LOC642852	*LOC642852*
1981	132	84,8	1,56	0,012927	0,355	222798_at	Phosphotriesterase related	*PTER*
407	5,58	3,59	1,56	0,002145	0,286	1566772_at	Schwannomin interacting protein 1	*SCHIP1*
4875	76,6	49,2	1,56	0,037222	0,417	222513_s_at	Sorbin and SH3 domain containing 1	*SORBS1*
1386	12,0	7,72	1,55	0,008329	0,329	1556770_a_a	Leucine rich repeat containing 17	*LRRC17*
3030	362	233	1,55	0,021326	0,385	212958_x_at	Peptidylglycine alpha-amidating monooxygenase	*PAM*
1952	126	81,1	1,55	0,012748	0,355	202124_s_at	Trafficking protein, kinesin binding 2	*TRAK2*
209	333	214	1,55	0,000955	0,244	209226_s_at	Transportin 1	*TNPO1*
1337	46,7	30,1	1,55	0,008024	0,328	205076_s_at	Myotubularin related protein 11	*MTMR11*
422	124	80,2	1,55	0,002210	0,286	226259_at	Exocyst complex component 6	*EXOC6*
3454	930	599	1,55	0,024746	0,392	210987_x_at	tropomyosin 1 (alpha)	*TPM1*
1709	10,3	6,63	1,55	0,010940	0,348	244443_at	Chromodomain helicase DNA binding protein 2	*CHD2*
2558	86,6	55,8	1,55	0,017356	0,371	231862_at	chromobox homolog 5 (HP1 alpha homolog, Drosophila)	*CBX5*
4052	29,7	19,1	1,55	0,029708	0,401	241838_at	Transcribed locus	-
2518	32,5	20,9	1,55	0,016986	0,369	1566766_a_a	Metastasis associated in colon cancer 1	*MACC1*
2506	41,2	26,6	1,55	0,016850	0,367	228121_at	Transforming growth factor, beta 2	*TGFB2*
1047	23,8	15,3	1,55	0,006120	0,319	236953_s_at	NHL repeat containing 3	*NHLRC3*
1930	28,1	18,1	1,55	0,012581	0,355	214153_at	ELOVL family member 5, elongation of long chain fatty acids	*ELOVL5*
5288	1110	717	1,55	0,041144	0,425	221731_x_at	Versican	*VCAN*
2894	42,3	27,3	1,55	0,020332	0,383	210102_at	Loss of heterozygosity, 11, chromosomal region 2, gene A	*LOH11CR2A*
3108	73,5	47,4	1,55	0,021946	0,386	230229_at	Discs, large homolog 1 (Drosophila)	*DLG1*
23	23,5	15,2	1,55	0,000099	0,201	226688_at	Chromosome 3 open reading frame 23	*C3orf23*
4571	51,9	33,5	1,55	0,034563	0,412	204689_at	Hematopoietically expressed homeobox	*HHEX*
803	21,7	14,0	1,55	0,004579	0,311	208022_s_at	CDC14 cell division cycle 14 homolog B (S. cerevisiae)	*CDC14B*
1632	161	104	1,55	0,010284	0,344	209485_s_at	Oxysterol binding protein-like 1A	*OSBPL1A*
540	225	145	1,55	0,002897	0,293	201960_s_at	MYC binding protein 2	*MYCBP2*
140	117	76,0	1,55	0,000635	0,244	223466_x_at	Collagen, type IV, alpha 3 (Goodpasture antigen) binding protein	*COL4A3BP*
2595	71,9	46,5	1,55	0,017646	0,371	201367_s_at	Zinc finger protein 36, C3H type-like 2	*ZFP36L2*
159	25,0	16,2	1,55	0,000715	0,244	240436_at	Similar to FRAS1 related extracellular matrix protein 2	*LOC729697*

Index	Mutated*	Normal*	FC	*P* value	FDR**	Probe set	Description	Gene symbol
3258	21,7	14,1	1,54	0,023139	0,388	207029_at	KIT ligand	*KITLG*
550	212	137	1,54	0,002973	0,295	214430_at	Galactosidase, alpha	*GLA*
1371	76,0	49,3	1,54	0,008235	0,328	226550_at	Similar to LOC169932	*LOC169932*
474	213	138	1,54	0,002518	0,289	225269_s_at	RNA binding motif, single stranded interacting protein 1	*RBMS1*
1273	880	571	1,54	0,007578	0,325	202371_at	Transcription elongation factor A (SII)-like 4	*TCEAL4*
1374	600	390	1,54	0,008283	0,329	214722_at	Notch homolog 2 (Drosophila) N-terminal like	*NOTCH2NL*
994	119	77,0	1,54	0,005727	0,315	212263_at	Quaking homolog, KH domain RNA binding (mouse)	*QKI*
3347	108	70,1	1,54	0,023768	0,388	222757_s_at	Sterile alpha motif and leucine zipper containing kinase AZK	*ZAK*
3778	159	103	1,54	0,027465	0,397	208935_s_at	Lectin, galactoside-binding, soluble, 8 (galectin 8)	*LGALS8*
1004	225	146	1,54	0,005809	0,316	201959_s_at	MYC binding protein 2	*MYCBP2*
649	82,9	53,9	1,54	0,003689	0,308	223178_s_at	5'-nucleotidase domain containing 1	*NT5DC1*
3306	14,0	9,13	1,54	0,023513	0,388	214375_at	PTPRF interacting protein, binding protein 1 (liprin beta 1)	*PPFIBP1*
2416	302	196	1,54	0,016133	0,365	228487_s_at	RAS responsive element binding protein 1	*RREB1*
2193	34,8	22,6	1,54	0,014511	0,361	225759_x_at	Calmin (calponin-like, transmembrane)	*CLMN*
361	1022	665	1,54	0,001870	0,283	221829_s_at	Transportin 1	*TNPO1*
216	67,9	44,2	1,54	0,000967	0,244	221215_s_at	Receptor-interacting serine-threonine kinase 4	*RIPK4*
5979	65,0	42,3	1,54	0,048543	0,444	242110_at	Rho GTPase activating protein 5	*ARHGAP5*
2195	106	69,2	1,54	0,014516	0,361	242558_at	Catenin (cadherin-associated protein), beta 1, 88kDa	*CTNNB1*
238	170	111	1,53	0,001103	0,250	202304_at	Fibronectin type III domain containing 3A	*FNDC3A*
3486	23,2	15,1	1,53	0,025021	0,392	210172_at	Splicing factor 1	*SF1*
176	67,6	44,1	1,53	0,000804	0,244	224943_at	BTB (POZ) domain containing 7	*BTBD7*
4600	11,2	7,28	1,53	0,034786	0,412	232568_at	hypothetical protein MGC24103	*MGC24103*
4694	1136	741	1,53	0,035453	0,413	200648_s_at	Glutamate-ammonia ligase (glutamine synthetase)	*GLUL*
2121	103	67,5	1,53	0,013913	0,359	212526_at	Spastic paraplegia 20	*SPG20*
4073	253	165	1,53	0,029870	0,401	203753_at	Transcription factor 4	*TCF4*
519	89,5	58,4	1,53	0,002761	0,291	238430_x_at	Schlafen family member 5	*SLFN5*
127	212	139	1,53	0,000574	0,240	215464_s_at	Tax1 (human T-cell leukemia virus type I) binding protein 3	*TAX1BP3*
4526	33,4	21,8	1,53	0,034161	0,412	205525_at	Caldesmon 1	*CALD1*
2172	67,9	44,3	1,53	0,014360	0,361	1566342_at	Superoxide dismutase 2, mitochondrial	*SOD2*
1734	17,6	11,5	1,53	0,011085	0,348	242737_at	RAD51-like 1 (S. cerevisiae)	*RAD51L1*
5855	174	114	1,53	0,047341	0,442	204352_at	TNF receptor-associated factor 5	*TRAF5*
722	62,4	40,8	1,53	0,004137	0,308	235341_at	DnaJ (Hsp40) homolog, subfamily C, member 3	*DNAJC3*
1921	15,4	10,0	1,53	0,012516	0,355	233106_at	Chromosome 14 open reading frame 82	*C14orf82*
5150	68,1	44,5	1,53	0,039641	0,421	218775_s_at	WW and C2 domain containing 2	*WWC2*
3237	22,2	14,5	1,53	0,022975	0,388	238563_at	Spectrin SH3 domain binding protein 1	*SSH3BP1*
516	261	171	1,53	0,002753	0,291	212265_at	Quaking homolog, KH domain RNA binding (mouse)	*QKI*
4799	31,2	20,5	1,53	0,036413	0,415	205529_s_at	Runt-related transcription factor 1; translocated to, 1 (cyclin D-related)	*RUNX1T1*
1592	22,2	14,5	1,53	0,009902	0,340	210325_at	CD1a molecule	*CD1A*
2268	18,2	11,9	1,53	0,015078	0,363	207018_s_at	RAB27B, member RAS oncogene family	*RAB27B*
3292	414	272	1,52	0,023445	0,388	201160_s_at	Cold shock domain protein A	*CSDA*
1119	169	111	1,52	0,006535	0,319	222413_s_at	Myeloid/lymphoid or mixed-lineage leukemia 3	*MLL3*
2989	52,0	34,1	1,52	0,021015	0,384	214720_x_at	Septin 10	*SEPT10*
4480	39,6	26,0	1,52	0,033694	0,411	202504_at	Tripartite motif-containing 29	*TRIM29*
1922	44,9	29,5	1,52	0,012516	0,355	235457_at	Mastermind-like 2 (Drosophila)	*MAML2*
925	10,1	6,62	1,52	0,005329	0,313	1558101_at	Nuclear factor I/A	*NFIA*
3933	48,3	31,7	1,52	0,028740	0,399	1553764_a_a	Jub, ajuba homolog (Xenopus laevis)	*JUB*
332	124	81,2	1,52	0,001654	0,272	222810_s_at	RAS protein activator like 2	*RASAL2*
5466	8,91	5,86	1,52	0,043170	0,432	215028_at	Semaphorin 6A-1	*SEMA6A*
1221	42,6	28,0	1,52	0,007263	0,324	243908_at	Zinc finger protein 638	*ZNF638*
934	31,6	20,8	1,52	0,005358	0,313	204761_at	USP6 N-terminal like	*USP6NL*
6004	57,9	38,1	1,52	0,048789	0,444	235775_at	Transmembrane and tetratricopeptide repeat containing 2	*TMTC2*
1258	174	115	1,52	0,007490	0,325	209488_s_at	RNA binding protein with multiple splicing	*RBPMS*
1200	38,0	25,0	1,52	0,007086	0,323	206734_at	Jerky homolog-like (mouse)	*JRKL*
1243	84,7	55,8	1,52	0,007383	0,324	207357_s_at	N-acetylgalactosaminyltransferase 10	*GALNT10*
2692	131	86,1	1,52	0,018450	0,375	203215_s_at	Myosin VI	*MYO6*
3802	18,7	12,3	1,52	0,027689	0,398	219683_at	Frizzled homolog 3 (Drosophila)	*FZD3*
3162	1052	694	1,52	0,022438	0,387	201288_at	Rho GDP dissociation inhibitor (GDI) beta	*ARHGDIB*

Index	Mutated*	Normal*	FC	*P* value	FDR**	Probe set	Description	Gene symbol
2035	269	177	1,52	0,013254	0,355	222605_at	REST corepressor 3	*RCOR3*
3178	105	69,4	1,51	0,022544	0,387	201656_at	Integrin, alpha 6	*ITGA6*
3078	161	107	1,51	0,021702	0,385	203143_s_at	KIAA0040 protein	*KIAA0040*
202	11,0	7,30	1,51	0,000929	0,244	220302_at	Male germ cell-associated kinase	*MAK*
522	1045	691	1,51	0,002786	0,291	223193_x_at	Family with sequence similarity 162, member A	*FAM162A*
3186	208	137	1,51	0,022596	0,387	224793_s_at	Transforming growth factor, beta receptor I	*TGFBR1*
3441	15,5	10,2	1,51	0,024630	0,391	233364_s_at	Hypothetical protein LOC399959	*LOC399959*
6	158	105	1,51	0,000013	0,099	212402_at	Zinc finger CCCH-type containing 13	*ZC3H13*
2501	14,2	9,38	1,51	0,016838	0,367	208859_s_at	Alpha thalassemia/mental retardation syndrome X-linked	*ATRX*
1801	11,2	7,42	1,51	0,011596	0,351	215164_at	Transcription factor 4	*TCF4*
1330	132	87,7	1,51	0,008001	0,328	217550_at	Activating transcription factor 6	*ATF6*
2080	7,30	4,84	1,51	0,013580	0,356	239301_at	RAS p21 protein activator (GTPase activating protein) 1	*RASA1*
46	66,0	43,7	1,51	0,000186	0,201	235687_at	Zinc finger protein 626	*ZNF626*
263	16,8	11,1	1,51	0,001259	0,260	210255_at	RAD51-like 1 (S. cerevisiae)	*RAD51L1*
1832	22,7	15,1	1,51	0,011798	0,351	221556_at	CDC14 cell division cycle 14 homolog B (S. cerevisiae)	*CDC14B*
2982	59,2	39,3	1,51	0,020965	0,384	238884_at	Catenin (cadherin-associated protein), delta 1	*CTNND1*
1404	11,6	7,70	1,51	0,008477	0,330	1552269_at	Spermatogenesis associated 17	*SPATA17*
6027	280	185	1,51	0,049027	0,445	202887_s_at	DNA-damage-inducible transcript 4	*DDIT4*
1739	140	93,0	1,51	0,011100	0,348	213015_at	Bobby sox homolog (Drosophila)	*BBX*
411	1115	739	1,51	0,002170	0,286	201220_x_at	C-terminal binding protein 2	*CTBP2*
5494	31,2	20,7	1,51	0,043485	0,433	233824_at	cDNA FLJ21428 fis, clone COL04203	-
1026	25,3	16,8	1,51	0,005942	0,316	243709_at	Solute carrier family 38, member 9	*SLC38A9*
95	23,9	15,8	1,51	0,000362	0,208	206636_at	RAS p21 protein activator 2	*RASA2*
2629	8,57	5,69	1,51	0,017897	0,372	213456_at	Sclerostin domain containing 1	*SOSTDC1*
445	26,2	17,4	1,51	0,002370	0,288	206928_at	Zinc finger protein 124	*ZNF124*
5403	138	91,7	1,51	0,042400	0,429	243768_at	SUMO1/sentrin specific peptidase 6	*SENP6*
4363	214	142	1,51	0,032453	0,407	227034_at	Ankyrin repeat domain 57	*ANKRD57*
5379	37,4	24,8	1,51	0,042181	0,429	224851_at	Cyclin-dependent kinase 6	*CDK6*
5690	40,9	27,2	1,50	0,045512	0,437	204624_at	ATPase, Cu++ transporting, beta polypeptide	*ATP7B*
1938	273	182	1,50	0,012623	0,355	235308_at	Zinc finger and BTB domain containing 20	*ZBTB20*
670	838	557	1,50	0,003849	0,308	224345_x_at	Family with sequence similarity 162, member A	*FAM162A*
244	1011	672	1,50	0,001123	0,251	209154_at	Tax1 (human T-cell leukemia virus type I) binding protein 3	*TAX1BP3*
210	44,7	29,7	1,50	0,000956	0,244	230192_at	Tripartite motif-containing 13	*TRIM13*
2728	806	536	1,50	0,018778	0,376	221896_s_at	HIG1 domain family, member 1A	*HIGD1A*
458	23,5	15,7	1,50	0,002449	0,288	239247_at	Hypothetical protein LOC401577	*LOC401577*
1131	86,2	57,4	1,50	0,006605	0,319	210223_s_at	Major histocompatibility complex, class I-related	*MR1*
730	113	75,3	1,50	0,004173	0,308	213528_at	Chromosome 1 open reading frame 156	*C1orf156*
76	484	323	1,50	0,000291	0,201	225132_at	F-box and leucine-rich repeat protein 3	*FBXL3*
2908	56,0	37,3	1,50	0,020442	0,383	1566785_x_a	N-ethylmaleimide-sensitive factor	*NSF*
1210	224	149	1,50	0,007173	0,324	224937_at	Prostaglandin F2 receptor negative regulator	*PTGFRN*
311	45,0	30,0	1,50	0,001536	0,270	1558733_at	Zinc finger and BTB domain containing 38	*ZBTB38*
2811	99,2	66,2	1,50	0,019599	0,381	227276_at	Plexin domain containing 2	*PLXDC2*
79	34,0	22,7	1,50	0,000297	0,201	210943_s_at	Lysosomal trafficking regulator	*LYST*
823	236	158	1,50	0,004700	0,312	209487_at	RNA binding protein with multiple splicing	*RBPMS*
859	32,8	21,9	1,50	0,004964	0,313	219455_at	Chromosome 7 open reading frame 63	*C7orf63*
4623	15,0	9,99	1,50	0,034877	0,412	239487_at	Family with sequence similarity 98, member A	*FAM98A*
790	55,1	36,8	1,50	0,004521	0,311	228825_at	prostaglandin reductase 1	*PTGR1*
5066	103	68,9	1,50	0,038994	0,420	242467_at	Casein kinase 1, alpha 1	*CSNK1A1*
3812	187	125	1,50	0,027781	0,398	200965_s_at	Actin binding LIM protein 1	*ABLIM1*
5281	31,4	21,0	1,50	0,041086	0,425	1554195_a_a	Chromosome 5 open reading frame 46	*C5orf46*
2063	147	98,4	1,50	0,013447	0,356	204633_s_at	Ribosomal protein S6 kinase, 90kDa, polypeptide 5	*RPS6KA5*
3438	16,0	10,7	1,49	0,024617	0,391	240861_at	-	-
5710	7,42	4,96	1,49	0,045721	0,438	238877_at	Eyes absent homolog 4 (Drosophila)	*EYA4*
1712	96,6	64,6	1,49	0,010957	0,348	204072_s_at	Furry homolog (Drosophila)	*FRY*
416	103	69,2	1,49	0,002189	0,286	225623_at	KIAA1737 protein	*KIAA1737*
318	291	195	1,49	0,001592	0,272	208908_s_at	Calpastatin	*CAST*
2103	31,4	21,0	1,49	0,013784	0,358	243851_at	RAB3 GTPase activating protein subunit 2 (non-catalytic)	*RAB3GAP2*

Index	Mutated*	Normal*	FC	*P* value	FDR**	Probe set	Description	Gene symbol
5197	45,0	30,2	1,49	0,040056	0,421	1555370_a_a	Calmodulin binding transcription activator 1	*CAMTA1*
4740	153	103	1,49	0,035879	0,414	218967_s_at	Phosphotriesterase related	*PTER*
2755	54,2	36,3	1,49	0,019071	0,378	1553186_x_a	RAS and EF-hand domain containing	*RASEF*
981	136	91,2	1,49	0,005689	0,315	223177_at	5'-nucleotidase domain containing 1	*NT5DC1*
272	77,2	51,8	1,49	0,001298	0,261	203128_at	Serine palmitoyltransferase, long chain base subunit 2	*SPTLC2*
3929	134	89,8	1,49	0,028733	0,399	209750_at	Nuclear receptor subfamily 1, group D, member 2	*NR1D2*
1158	29,2	19,6	1,49	0,006810	0,321	229603_at	Bardet-Biedl syndrome 12	*BBS12*
21	484	325	1,49	0,000088	0,201	223287_s_at	Forkhead box P1	*FOXP1*
1527	9,65	6,48	1,49	0,009430	0,337	244860_at	SIK family kinase 3	*SIK3*
2315	29,3	19,7	1,49	0,015371	0,363	234604_at	cDNA FLJ21228 fis, clone COL00739	-
5476	70,8	47,6	1,49	0,043292	0,432	239082_at	cDNA clone IMAGE:5311370	-
3100	51,0	34,3	1,49	0,021899	0,386	219033_at	Poly (ADP-ribose) polymerase family, member 8	*PARP8*
5516	28,8	19,4	1,49	0,043749	0,433	210653_s_at	Branched chain keto acid dehydrogenase E1, beta polypeptide	*BCKDHB*
2096	23,7	16,0	1,49	0,013733	0,358	226984_at	FYVE, RhoGEF and PH domain containing 5	*FGD5*
532	102	68,4	1,49	0,002845	0,292	226977_at	Similar to bovine IgA regulatory protein	*LOC492311*
5792	405	272	1,49	0,046712	0,441	222258_s_at	SH3-domain binding protein 4	*SH3BP4*
801	73,9	49,7	1,49	0,004571	0,311	217813_s_at	Spindlin 1	*SPIN1*
5075	99,6	67,0	1,49	0,039043	0,420	203706_s_at	Frizzled homolog 7 (Drosophila)	*FZD7*
3434	547	368	1,49	0,024581	0,391	200907_s_at	Palladin, cytoskeletal associated protein	*PALLD*
4098	97,4	65,6	1,48	0,030117	0,402	226246_at	Potassium channel tetramerisation domain containing 1	*KCTD1*
425	159	107	1,48	0,002236	0,288	214041_x_at	Ribosomal protein L37a	*RPL37A*
2494	137	92,5	1,48	0,016801	0,367	203319_s_at	Zinc finger protein 148	*ZNF148*
4335	81,9	55,2	1,48	0,032270	0,407	207604_s_at	Solute carrier family 4, sodium bicarbonate cotransporter, member 7	*SLC4A7*
1573	34,4	23,2	1,48	0,009768	0,340	206117_at	Tropomyosin 1 (alpha)	*TPM1*
2179	18,5	12,5	1,48	0,014410	0,361	219885_at	schlafen family member 12	*SLFN12*
5482	125	84,5	1,48	0,043380	0,433	203266_s_at	Mitogen-activated protein kinase kinase 4	*MAP2K4*
1036	77,9	52,5	1,48	0,006019	0,318	226722_at	Family with sequence similarity 20, member C	*FAM20C*
2849	448	302	1,48	0,019937	0,383	201324_at	Epithelial membrane protein 1	*EMP1*
4784	10,7	7,20	1,48	0,036271	0,414	210282_at	Zinc finger, MYM-type 2	*ZMYM2*
2269	61,9	41,7	1,48	0,015078	0,363	219390_at	FK506 binding protein 14, 22 kDa	*FKBP14*
1466	135	91,4	1,48	0,008970	0,334	230669_at	RAS p21 protein activator 2	*RASA2*
1932	480	324	1,48	0,012601	0,355	221943_x_at	Ribosomal protein L38	*RPL38*
3784	159	107	1,48	0,027562	0,398	226582_at	Hypothetical gene supported by BC009385	*LOC400043*
3905	1286	869	1,48	0,028521	0,399	200872_at	S100 calcium binding protein A10	*S100A10*
643	271	183	1,48	0,003668	0,308	212698_s_at	Septin 10	*SEPT10*
250	25,5	17,2	1,48	0,001148	0,251	206587_at	Chaperonin containing TCP1, subunit 6B (zeta 2)	*CCT6B*
4710	40,3	27,2	1,48	0,035639	0,413	221826_at	Angel homolog 2 (Drosophila)	*ANGEL2*
4637	48,9	33,1	1,48	0,034974	0,412	201996_s_at	Spen homolog, transcriptional regulator (Drosophila)	*SPEN*
6085	330	223	1,48	0,049690	0,446	225064_at	Rabaptin, RAB GTPase binding effector protein 1	*RABEP1*
1241	286	193	1,48	0,007365	0,324	215127_s_at	RNA binding motif, single stranded interacting protein 1	*RBMS1*
2522	77,8	52,7	1,48	0,017014	0,369	227701_at	Chromosome 10 open reading frame 118	*C10orf118*
3120	59,2	40,0	1,48	0,022086	0,387	228852_at	Endosulfine alpha	*ENSA*
1746	183	124	1,48	0,011149	0,348	208663_s_at	Tetratricopeptide repeat domain 3	*TTC3*
4036	9,30	6,29	1,48	0,029576	0,401	211124_s_at	KIT ligand	*KITLG*
5724	50,6	34,3	1,48	0,045873	0,438	230337_at	SON of sevenless homolog 1 (Drosophila)	*SOS1*
4448	27,8	18,8	1,48	0,033401	0,411	235408_x_at	Zinc finger protein 117	*ZNF117*
2653	11,4	7,69	1,48	0,018089	0,373	241789_at	RNA binding motif, single stranded interacting protein	*RBMS3*
793	15,2	10,3	1,48	0,004532	0,311	244023_at	Spleen tyrosine kinase	*SYK*
471	82,7	56,0	1,48	0,002509	0,289	222632_s_at	Leucine zipper transcription factor-like 1	*LZTFL1*
3809	63,1	42,7	1,48	0,027752	0,398	223134_at	Bobby sox homolog (Drosophila)	*BBX*
2560	39,3	26,6	1,48	0,017359	0,371	219204_s_at	serine racemase	*SRR*
3752	119	81,0	1,48	0,027281	0,397	211783_s_at	Metastasis associated 1	*MTA1*
1865	531	360	1,48	0,012051	0,353	213229_at	Dicer1, Dcr-1 homolog (Drosophila)	*DICER1*
1141	59,4	40,3	1,47	0,006692	0,320	238444_at	Zinc finger protein 618	*ZNF618*
2250	5,69	3,86	1,47	0,014951	0,363	1554557_at	ATPase, Class VI, type 11B	*ATP11B*
4777	280	190	1,47	0,036218	0,414	202133_at	WW domain containing transcription regulator 1	*WWTR1*
4107	31,0	21,0	1,47	0,030190	0,402	219501_at	Ecto-NOX disulfide-thiol exchanger 1	*ENOX1*

Index	Mutated*	Normal*	FC	*P* value	FDR**	Probe set	Description	Gene symbol
1736	40,8	27,7	1,47	0,011097	0,348	228772_at	Histamine N-methyltransferase	*HNMT*
5917	43,5	29,6	1,47	0,047951	0,443	227415_at	Hypothetical protein LOC283508	*LOC283508*
2251	71,0	48,2	1,47	0,014960	0,363	201151_s_at	Muscleblind-like (Drosophila)	*MBNL1*
2871	113	76,8	1,47	0,020167	0,383	211110_s_at	Androgen receptor	*AR*
1560	200	136	1,47	0,009649	0,338	218595_s_at	HEAT repeat containing 1	*HEATR1*
2299	12,3	8,38	1,47	0,015275	0,363	207017_at	RAB27B, member RAS oncogene family	*RAB27B*
2975	114	77,4	1,47	0,020917	0,384	218721_s_at	Chromosome 1 open reading frame 27	*C1orf27*
3546	269	183	1,47	0,025502	0,393	218084_x_at	FXYD domain containing ion transport regulator 5	*FXYD5*
701	45,0	30,6	1,47	0,004023	0,308	1555913_at	Gon-4-like (C. elegans)	*GON4L*
349	21,8	14,9	1,47	0,001778	0,278	238044_at	Tripartite motif-containing 8	*TRIM8*
3366	7,10	4,83	1,47	0,023903	0,388	209866_s_at	Latrophilin 3	*LPHN3*
4591	19,5	13,3	1,47	0,034720	0,412	238575_at	Oxysterol binding protein-like 6	*OSBPL6*
1358	329	224	1,47	0,008193	0,328	224811_at	cDNA FLJ30652 fis, clone DFNES2000011	-
1425	27,2	18,5	1,47	0,008633	0,331	232522_at	Transcription factor 7-like 2 (T-cell specific, HMG-box)	*TCF7L2*
3084	34,7	23,6	1,47	0,021728	0,385	227785_at	Serologically defined colon cancer antigen 8	*SDCCAG8*
2156	24,9	17,0	1,47	0,014207	0,360	240145_at	Transcribed locus	-
2166	85,7	58,4	1,47	0,014337	0,361	1553185_at	RAS and EF-hand domain containing	*RASEF*
3227	110	74,7	1,47	0,022857	0,387	224829_at	Cytoplasmic polyadenylation element binding protein 4	*CPEB4*
3272	181	124	1,47	0,023277	0,388	224252_s_at	FXYD domain containing ion transport regulator 5	*FXYD5*
4202	90,2	61,5	1,47	0,031079	0,404	214620_x_at	Peptidylglycine alpha-amidating monooxygenase	*PAM*
137	71,4	48,7	1,47	0,000619	0,244	209648_x_at	Suppressor of cytokine signaling 5	*SOCS5*
4500	90,0	61,4	1,47	0,033844	0,411	206848_at	Homeobox A7	*HOXA7*
1309	158	108	1,47	0,007815	0,325	206453_s_at	NDRG family member 2	*NDRG2*
4606	264	180	1,47	0,034808	0,412	208936_x_at	Lectin, galactoside-binding, soluble, 8 (galectin 8)	*LGALS8*
4755	463	316	1,46	0,036026	0,414	224694_at	Anthrax toxin receptor 1	*ANTXR1*
1357	549	375	1,46	0,008184	0,328	221478_at	BCL2/adenovirus E1B 19kDa interacting protein 3-like	*BNIP3L*
1857	65,1	44,5	1,46	0,011965	0,352	1555480_a_a	Filamin binding LIM protein 1	*FBLIM1*
599	136	93,0	1,46	0,003295	0,301	207956_x_at	PDS5, regulator of cohesion maintenance, homolog B (S. cerevisiae)	*PDS5B*
1154	55,3	37,8	1,46	0,006778	0,321	231002_s_at	Rabaptin, RAB GTPase binding effector protein 1	*RABEP1*
4334	136	93,0	1,46	0,032264	0,407	224726_at	Mindbomb homolog 1 (Drosophila)	*MIB1*
3027	59,4	40,6	1,46	0,021305	0,385	216202_s_at	Serine palmitoyltransferase, long chain base subunit 2	*SPTLC2*
928	18,4	12,6	1,46	0,005332	0,313	214052_x_at	BAT2 domain containing 1	*BAT2D1*
2334	9,58	6,55	1,46	0,015569	0,364	236558_at	Muscleblind-like (Drosophila)	*MBNL1*
464	322	220	1,46	0,002459	0,288	207657_x_at	Transportin 1	*TNPO1*
530	808	553	1,46	0,002835	0,292	229353_s_at	Nuclear casein kinase and cyclin-dependent kinase substrate 1	*NUCKS1*
2927	18,5	12,7	1,46	0,020511	0,383	1552627_a_a	Rho GTPase activating protein 5	*ARHGAP5*
1971	169	115	1,46	0,012883	0,355	202421_at	Immunoglobulin superfamily, member 3	*IGSF3*
1454	13,4	9,19	1,46	0,008893	0,334	219523_s_at	Odz, odd Oz/ten-m homolog 3 (Drosophila)	*ODZ3*
452	358	245	1,46	0,002417	0,288	207467_x_at	Calpastatin	*CAST*
1169	15,4	10,5	1,46	0,006876	0,321	236446_at	Transcribed locus	-
1419	25,4	17,4	1,46	0,008598	0,331	222237_s_at	Zinc finger protein 228	*ZNF228*
810	297	204	1,46	0,004618	0,311	222619_at	Zinc finger protein 281	*ZNF281*
1345	350	240	1,46	0,008066	0,328	221479_s_at	BCL2/adenovirus E1B 19kDa interacting protein 3-like	*BNIP3L*
4319	76,6	52,5	1,46	0,032160	0,407	1562836_at	DEAD (Asp-Glu-Ala-Asp) box polypeptide 6	*DDX6*
5787	147	101	1,46	0,046602	0,440	228067_at	Chromosome 2 open reading frame 55	*C2orf55*
3941	297	204	1,46	0,028773	0,399	208933_s_at	Lectin, galactoside-binding, soluble, 8 (galectin 8)	*LGALS8*
2691	197	135	1,46	0,018447	0,375	228545_at	Zinc finger protein 148	*ZNF148*
4372	94,4	64,8	1,46	0,032522	0,407	230085_at	Pyruvate dehydrogenase kinase, isozyme 3	*PDK3*
850	9,70	6,66	1,46	0,004893	0,313	236862_at	Golgi associated PDZ and coiled-coil motif containing	*GOPC*
2706	48,7	33,5	1,46	0,018554	0,375	1556373_a_a	Family with sequence similarity 185, member A	*FAM185A*
4563	14,5	9,96	1,46	0,034487	0,412	1557432_at	RAS protein activator like 2	*RASAL2*
883	453	311	1,45	0,005109	0,313	204517_at	Peptidylprolyl isomerase C (cyclophilin C)	*PPIC*
2126	24,2	16,7	1,45	0,013994	0,359	204964_s_at	Sarcospan (Kras oncogene-associated gene)	*SSPN*
942	40,8	28,1	1,45	0,005401	0,313	234912_at	Hypothetical protein LOC651964	*LOC651964*
1393	66,2	45,6	1,45	0,008424	0,330	219542_at	NIMA (never in mitosis gene a)- related kinase 11	*NEK11*
3112	180	124	1,45	0,021978	0,386	202379_s_at	Natural killer-tumor recognition sequence	*NKTR*
3412	1619	1115	1,45	0,024339	0,390	208788_at	ELOVL family member 5, elongation of long chain fatty acids	*ELOVL5*

Index	Mutated*	Normal*	FC	*P* value	FDR**	Probe set	Description	Gene symbol
3279	44,8	30,9	1,45	0,023353	0,388	204255_s_at	Vitamin D (1,25- dihydroxyvitamin D3) receptor	*VDR*
3857	11,4	7,87	1,45	0,028161	0,399	237733_at	KIAA0564 protein	*KIAA0564*
1925	10,9	7,52	1,45	0,012527	0,355	243582_at	SH3 domain containing ring finger 2	*SH3RF2*
5230	386	266	1,45	0,040466	0,423	214433_s_at	Selenium binding protein 1	*SELENBP1*
5807	8,43	5,81	1,45	0,046861	0,441	232541_at	Avian erythroblastic leukemia viral (v-erb-b) oncogene homolog	*EGFR*
3622	17,9	12,4	1,45	0,026148	0,395	215248_at	Growth factor receptor-bound protein 10	*GRB10*
573	696	480	1,45	0,003149	0,300	201218_at	C-terminal binding protein 2	*CTBP2*
303	129	88,9	1,45	0,001465	0,263	210596_at	Magnesium transporter 1	*MAGT1*
1177	156	108	1,45	0,006912	0,321	225295_at	Solute carrier family 39 (zinc transporter), member 10	*SLC39A10*
4695	55,5	38,3	1,45	0,035459	0,413	225178_at	Tetratricopeptide repeat domain 14	*TTC14*
2714	1094	755	1,45	0,018626	0,375	217845_x_at	HIG1 domain family, member 1A	*HIGD1A*
2454	36,6	25,3	1,45	0,016453	0,366	216682_s_at	Family with sequence similarity 48, member A	*FAM48A*
3126	92,1	63,6	1,45	0,022169	0,387	207233_s_at	Microphthalmia-associated transcription factor	*MITF*
5193	9,78	6,75	1,45	0,040022	0,421	226210_s_at	Maternally expressed 3	*MEG3*
174	5,84	4,03	1,45	0,000800	0,244	237951_at	Zinc finger protein 2 homlog (mouse)	*ZFP2*
4005	14,7	10,2	1,45	0,029314	0,400	1566442_at	Chromosome 1 open reading frame 21	*C1orf21*
1389	70,3	48,5	1,45	0,008388	0,330	214056_at	Myeloid cell leukemia sequence 1 (BCL2-related)	*MCL1*
6077	57,6	39,8	1,45	0,049587	0,446	228806_at	RAR-related orphan receptor C	*RORC*
4958	107	74,1	1,45	0,038042	0,419	226245_at	Potassium channel tetramerisation domain containing 1	*KCTD1*
3313	98,6	68,2	1,45	0,023552	0,388	213593_s_at	Transformer-2 alpha	*TRA2A*
2760	309	213	1,45	0,019155	0,379	211202_s_at	Jumonji, AT rich interactive domain 1B	*JARID1B*
4380	11,8	8,16	1,45	0,032570	0,407	209792_s_at	Kallikrein-related peptidase 10	*KLK10*
312	10,6	7,35	1,45	0,001564	0,272	214422_at	RAD23 homolog B (S. cerevisiae)	*RAD23B*
3023	71,5	49,5	1,44	0,021277	0,385	213956_at	Centrosomal protein 350kDa	*CEP350*
4094	263	182	1,44	0,030088	0,402	201616_s_at	Caldesmon 1	*CALD1*
2028	24,1	16,7	1,44	0,013220	0,355	208499_s_at	DnaJ (Hsp40) homolog, subfamily C, member 3	*DNAJC3*
131	52,4	36,3	1,44	0,000586	0,240	218849_s_at	Protein phosphatase 1, regulatory (inhibitor) subunit 13 like	*PPP1R13L*
1809	25,5	17,7	1,44	0,011666	0,351	232776_at	PDZ domain containing ring finger 3	*PDZRN3*
3596	41,8	28,9	1,44	0,025928	0,394	232333_at	Mastermind-like 2 (Drosophila)	*MAML2*
2815	122	84,6	1,44	0,019632	0,381	202321_at	Geranylgeranyl diphosphate synthase 1	*GGPS1*
1585	101	69,8	1,44	0,009867	0,340	202514_at	Discs, large homolog 1 (Drosophila)	*DLG1*
3131	77,0	53,4	1,44	0,022212	0,387	225968_at	Prickle homolog 2 (Drosophila)	*PRICKLE2*
4388	288	200	1,44	0,032699	0,407	201549_x_at	Jumonji, AT rich interactive domain 1B	*JARID1B*
6056	251	174	1,44	0,049403	0,446	226538_at	Mannosidase, alpha, class 2A, member 1	*MAN2A1*
551	109	75,5	1,44	0,002974	0,295	203127_s_at	Serine palmitoyltransferase, long chain base subunit 2	*SPTLC2*
3561	12,7	8,84	1,44	0,025700	0,394	239784_at	SET and MYND domain containing 3	*SMYD3*
1656	325	226	1,44	0,010451	0,345	221816_s_at	PHD finger protein 11	*PHF11*
248	157	109	1,44	0,001141	0,251	225192_at	Chromosome 10 open reading frame 46	*C10orf46*
3394	55,6	38,6	1,44	0,024189	0,390	228173_at	GNAS complex locus	*GNAS*
1556	57,8	40,1	1,44	0,009606	0,337	222679_s_at	DCUN1 domain-containing protein 1	*DCUN1D1*
2832	86,8	60,3	1,44	0,019764	0,381	223380_s_at	LATS, large tumor suppressor, homolog 2 (Drosophila)	*LATS2*
158	346	241	1,44	0,000714	0,244	201152_s_at	Muscleblind-like (Drosophila)	*MBNL1*
2929	69,2	48,1	1,44	0,020535	0,383	222850_s_at	DnaJ (Hsp40) homolog, subfamily B, member 14	*DNAJB14*
2475	103	71,2	1,44	0,016666	0,367	204345_at	Collagen, type XVI, alpha 1	*COL16A1*
3913	48,2	33,5	1,44	0,028564	0,399	229689_s_at	Discs, large homolog 5 (Drosophila)	*DLG5*
6064	35,0	24,3	1,44	0,049456	0,446	235000_at	cDNA FLJ30652 fis, clone DFNES2000011	-
208	57,8	40,2	1,44	0,000955	0,244	214001_x_at	Family with sequence similarity 110, member A	*FAM110A*
3939	187	130	1,44	0,028764	0,399	212387_at	Transcription factor 4	*TCF4*
980	6,13	4,27	1,44	0,005688	0,315	242664_at	-	-
217	754	525	1,44	0,000970	0,244	224596_at	Solute carrier family 44, member 1	*SLC44A1*
4053	10,6	7,37	1,44	0,029723	0,401	243140_at	Actin, alpha 2, smooth muscle, aorta	*ACTA2*
2311	208	145	1,44	0,015361	0,363	227322_s_at	BRCA2 and CDKN1A interacting protein	*BCCIP*
1721	76,0	52,9	1,44	0,011024	0,348	225988_at	Hect domain and RLD 4	*HERC4*
4899	10,5	7,28	1,44	0,037488	0,418	1563022_at	Coiled-coil domain containing 160	*CCDC160*
5926	187	130	1,44	0,048015	0,443	208934_s_at	Lectin, galactoside-binding, soluble, 8 (galectin 8)	*LGALS8*
3044	47,5	33,1	1,44	0,021431	0,385	1565639_a_a	Peripheral myelin protein 22	*PMP22*
2920	53,5	37,2	1,44	0,020492	0,383	212634_at	KIAA0776 protein	*KIAA0776*

Index	Mutated*	Normal*	FC	*P* value	FDR**	Probe set	Description	Gene symbol
84	109	75,7	1,44	0,000313	0,201	208127_s_at	Suppressor of cytokine signaling 5	*SOCS5*
726	5,63	3,92	1,44	0,004148	0,308	1555573_at	Chromosome 10 open reading frame 93	*C10orf93*
963	227	158	1,43	0,005572	0,315	218501_at	Rho guanine nucleotide exchange factor (GEF) 3	*ARHGEF3*
1634	7,77	5,42	1,43	0,010310	0,344	240110_at	3-hydroxy-3-methylglutaryl-Coenzyme A synthase 2 (mitochondrial)	*HMGCS2*
699	146	102	1,43	0,004009	0,308	222424_s_at	Nuclear casein kinase and cyclin-dependent kinase substrate 1	*NUCKS1*
1812	70,7	49,3	1,43	0,011684	0,351	215281_x_at	Pogo transposable element with ZNF domain	*POGZ*
4311	23,9	16,7	1,43	0,032096	0,407	242144_at	PAP associated domain containing 4	*PAPD4*
440	7,19	5,01	1,43	0,002338	0,288	231247_s_at	Hypothetical protein LOC730257	*LOC730257*
2016	523	364	1,43	0,013150	0,355	212586_at	Calpastatin	*CAST*
5620	19,3	13,5	1,43	0,044865	0,436	235121_at	Zinc finger protein 542	*ZNF542*
3522	71,0	49,5	1,43	0,025277	0,392	227931_at	INO80 complex subunit D	*INO80D*
5642	213	149	1,43	0,045018	0,436	201694_s_at	Early growth response 1	*EGR1*
3223	30,0	20,9	1,43	0,022835	0,387	1569353_at	CP110 protein	*CP110*
1196	133	92,8	1,43	0,007063	0,323	207266_x_at	RNA binding motif, single stranded interacting protein 1	*RBMS1*
2186	22,3	15,6	1,43	0,014484	0,361	210281_s_at	Zinc finger, MYM-type 2	*ZMYM2*
3386	1537	1073	1,43	0,024121	0,389	201312_s_at	SH3 domain binding glutamic acid-rich protein like	*SH3BGRL*
77	66,7	46,5	1,43	0,000292	0,201	212746_s_at	Centrosomal protein 170kDa	*CEP170*
4892	169	118	1,43	0,037385	0,418	223681_s_at	InaD-like (Drosophila)	*INADL*
2394	287	200	1,43	0,015979	0,365	206555_s_at	THUMP domain containing 1	*THUMPD1*
5374	13,4	9,33	1,43	0,042164	0,429	205559_s_at	Proprotein convertase subtilisin/kexin type 5	*PCSK5*
1725	145	101	1,43	0,011045	0,348	218149_s_at	Zinc finger protein 395	*ZNF395*
3225	15,0	10,5	1,43	0,022842	0,387	1565566_a_a	Syntaxin 7	*STX7*
1446	156	109	1,43	0,008790	0,332	201917_s_at	Solute carrier family 25, member 36	*SLC25A36*
2077	50,8	35,5	1,43	0,013569	0,356	220296_at	N-acetylgalactosaminyltransferase 10	*GALNT10*
397	482	337	1,43	0,002086	0,285	211967_at	Transmembrane protein 123	*TMEM123*
1803	179	125	1,43	0,011612	0,351	224889_at	Forkhead box O3	*FOXO3*
4435	116	81,2	1,43	0,033215	0,409	227561_at	Discoidin domain receptor family, member 2	*DDR2*
419	297	207	1,43	0,002195	0,286	201846_s_at	RING1 and YY1 binding protein	*RYBP*
286	449	314	1,43	0,001399	0,263	225010_at	Coiled-coil domain containing 6	*CCDC6*
3402	79,1	55,3	1,43	0,024278	0,390	226876_at	Family with sequence similarity 101, member B	*FAM101B*
5495	14,3	10,0	1,43	0,043490	0,433	205006_s_at	N-myristoyltransferase 2	*NMT2*
3530	37,5	26,3	1,43	0,025361	0,393	232994_s_at	Rho-guanine nucleotide exchange factor	*RGNEF*
873	250	175	1,43	0,005045	0,313	202184_s_at	Nucleoporin 133kDa	*NUP133*
855	20,8	14,6	1,43	0,004930	0,313	222565_s_at	Protein kinase D3	*PRKD3*
5158	87,2	61,0	1,43	0,039715	0,421	226899_at	Unc-5 homolog B (C. elegans)	*UNC5B*
2116	13,9	9,70	1,43	0,013875	0,359	236417_at	cDNA clone YI40G07	*MFN1*
2502	46,6	32,6	1,43	0,016840	0,367	214395_x_at	Eukaryotic translation elongation factor 1 delta	*EEF1D*
2688	87,6	61,3	1,43	0,018424	0,375	1568678_s_a	FGFR1 oncogene partner	*FGFR1OP*
5285	510	357	1,43	0,041112	0,425	209684_at	RAS and RAB interactor 2	*RIN2*
1903	171	120	1,43	0,012376	0,355	204290_s_at	Aldehyde dehydrogenase 6 family, member A1	*ALDH6A1*
4282	14,6	10,2	1,43	0,031816	0,406	233607_at	Bicaudal C homolog 1 (Drosophila)	*BICC1*
5555	13,4	9,37	1,43	0,044219	0,435	236322_at	Ring finger protein 145	*RNF145*
5073	338	237	1,43	0,039041	0,420	212492_s_at	Jumonji domain containing 2B	*JMJD2B*
1962	112	78,5	1,43	0,012819	0,355	201219_at	C-terminal binding protein 2	*CTBP2*
1033	942	661	1,43	0,006002	0,318	220942_x_at	Family with sequence similarity 162, member A	*FAM162A*
4593	493	345	1,43	0,034757	0,412	214709_s_at	Kinectin 1 (kinesin receptor)	*KTN1*
465	157	110	1,43	0,002459	0,288	224736_at	Cell division cycle and apoptosis regulator 1	*CCAR1*
2132	15,7	11,0	1,43	0,014015	0,359	221986_s_at	Kelch-like 24 (Drosophila)	*KLHL24*
2509	28,5	20,0	1,43	0,016870	0,368	235959_at	AT rich interactive domain 4B (RBP1-like)	*ARID4B*
346	132	92,8	1,43	0,001775	0,278	218491_s_at	Thymocyte nuclear protein 1	*THYN1*
5567	20,8	14,6	1,43	0,044315	0,435	204324_s_at	Golgi integral membrane protein 4	*GOLIM4*
4869	125	87,8	1,43	0,037158	0,417	217738_at	Pre-B-cell colony enhancing factor 1	*PBEF1*
5587	54,6	38,3	1,42	0,044562	0,436	222592_s_at	Acyl-CoA synthetase long-chain family member 5	*ACSL5*
3532	29,2	20,5	1,42	0,025384	0,393	238493_at	Zinc finger protein 506	*ZNF506*
3174	79,7	56,0	1,42	0,022511	0,387	229732_at	Zinc finger protein 823	*ZNF823*
1953	97,2	68,2	1,42	0,012763	0,355	219553_at	Nucleoside diphosphate kinase 7	*NME7*
1421	533	375	1,42	0,008609	0,331	208661_s_at	Tetratricopeptide repeat domain 3	*TTC3*

Index	Mutated*	Normal*	FC	*P* value	FDR**	Probe set	Description	Gene symbol
5589	69,2	48,7	1,42	0,044579	0,436	223201_s_at	Transmembrane protein 164	*TMEM164*
52	9,29	6,54	1,42	0,000201	0,201	207068_at	Zinc finger protein 37 homolog (mouse)	*ZFP37*
1980	26,2	18,5	1,42	0,012920	0,355	1556049_at	Reticulon 4	*RTN4*
1622	538	379	1,42	0,010180	0,343	201008_s_at	Thioredoxin interacting protein	*TXNIP*
692	6,73	4,74	1,42	0,003985	0,308	1559706_at	Rho-guanine nucleotide exchange factor	*RGNEF*
2777	272	192	1,42	0,019296	0,380	208158_s_at	Oxysterol binding protein-like 1A	*OSBPL1A*
2220	17,5	12,3	1,42	0,014713	0,362	236600_at	Spastic paraplegia 20	*SPG20*
1471	179	126	1,42	0,008991	0,334	221588_x_at	Aldehyde dehydrogenase 6 family, member A1	*ALDH6A1*
2743	28,2	19,9	1,42	0,018927	0,377	1565149_at	Dynein, cytoplasmic 2, heavy chain 1	*DYNC2H1*
4116	55,9	39,4	1,42	0,030270	0,402	205588_s_at	FGFR1 oncogene partner	*FGFR1OP*
3141	105	74,1	1,42	0,022294	0,387	227121_at	cDNA DKFZp586K1922	-
339	43,9	30,9	1,42	0,001733	0,277	227056_at	KIAA0141 protein	*KIAA0141*
846	76,7	54,1	1,42	0,004873	0,313	235889_at	Transcribed locus	-
2170	50,0	35,3	1,42	0,014349	0,361	205786_s_at	Integrin, alpha M (complement component 3 receptor 3 subunit)	*ITGAM*
4642	7,31	5,15	1,42	0,035008	0,412	206713_at	Netrin G1	*NTNG1*
5241	248	175	1,42	0,040595	0,423	218435_at	DnaJ (Hsp40) homolog, subfamily C, member 15	*DNAJC15*
4112	41,3	29,2	1,42	0,030216	0,402	208555_x_at	Cystatin SA	*CST2*
2819	73,3	51,8	1,42	0,019671	0,381	214499_s_at	BCL2-associated transcription factor 1	*BCLAF1*
5122	47,0	33,2	1,41	0,039430	0,421	209781_s_at	KH domain containing, RNA binding, signal transduction associated 3	*KHDRBS3*
4580	17,1	12,1	1,41	0,034614	0,412	239486_at	Transcribed locus	-
4560	25,8	18,3	1,41	0,034463	0,412	229276_at	Immunoglobulin superfamily, member 9	*IGSF9*
302	84,9	60,0	1,41	0,001464	0,263	223711_s_at	Thymocyte nuclear protein 1	*THYN1*
3502	210	148	1,41	0,025172	0,392	209210_s_at	Fermitin family homolog 2 (Drosophila)	*FERMT2*
4599	96,6	68,4	1,41	0,034777	0,412	229238_at	Chromosome 17 open reading frame 97	*C17orf97*
5304	41,6	29,5	1,41	0,041386	0,426	244674_at	RNA binding motif protein 6	*RBM6*
1491	148	105	1,41	0,009194	0,336	219622_at	RAB20, member RAS oncogene family	*RAB20*
4669	37,1	26,3	1,41	0,035252	0,413	234785_at	-	-
5957	231	164	1,41	0,048343	0,444	210480_s_at	Myosin VI	*MYO6*
3602	117	83,1	1,41	0,025977	0,394	225956_at	Adult retina protein	*LOC153222*
1879	24,8	17,6	1,41	0,012162	0,354	204037_at	Lysophosphatidic acid receptor 1	*EDG2*
2477	769	545	1,41	0,016675	0,367	222433_at	Enabled homolog (Drosophila)	*ENAH*
444	10,6	7,52	1,41	0,002366	0,288	212079_s_at	Myeloid/lymphoid or mixed-lineage leukemia	*MLL*
6051	88,0	62,4	1,41	0,049301	0,445	1553575_at	Mitochondrially encoded NADH dehydrogenase 6	*MTND6*
2145	13,2	9,35	1,41	0,014119	0,360	1559361_at	Metastasis associated in colon cancer 1	*MACC1*
4382	19,5	13,8	1,41	0,032602	0,407	227752_at	Serine palmitoyltransferase, long chain base subunit 3	*SPTLC3*
2588	23,7	16,8	1,41	0,017550	0,371	209293_x_at	Inhibitor of DNA binding 4, dominant negative helix-loop-helix protein	*ID4*
3045	43,0	30,5	1,41	0,021431	0,385	239515_at	Sperm autoantigenic protein 17	*SPA17*
3630	15,5	11,0	1,41	0,026233	0,395	231235_at	Natural killer-tumor recognition sequence	*NKTR*
1066	79,8	56,7	1,41	0,006255	0,319	211711_s_at	Phosphatase and tensin homolog	*PTEN*
3382	150	106	1,41	0,024073	0,389	214055_x_at	BAT2 domain containing 1	*BAT2D1*
3040	36,9	26,2	1,41	0,021426	0,385	230179_at	Hypothetical protein LOC285812	*LOC285812*
1009	13,0	9,24	1,41	0,005855	0,316	239606_at	I beta-1,6-N-acetylglucosaminyltransferase	*GCNT2*
1703	10,1	7,18	1,41	0,010860	0,348	232978_at	Muscleblind-like 2 (Drosophila)	*MBNL2*
489	16,7	11,9	1,41	0,002620	0,289	238723_at	Ataxin 3	*ATXN3*
5497	142	101	1,41	0,043511	0,433	213224_s_at	Non-protein coding RNA 81	*NCRNA00081*
3635	12,8	9,10	1,41	0,026290	0,395	236562_at	Zinc finger protein 439	*ZNF439*
2548	236	168	1,41	0,017216	0,369	203318_s_at	Zinc finger protein 148	*ZNF148*
2745	216	154	1,41	0,018992	0,378	205383_s_at	Zinc finger and BTB domain containing 20	*ZBTB20*
1956	246	175	1,41	0,012781	0,355	203640_at	Muscleblind-like 2 (Drosophila)	*MBNL2*
3690	234	166	1,41	0,026655	0,395	225258_at	Filamin binding LIM protein 1	*FBLIM1*
5090	6,45	4,59	1,41	0,039171	0,420	1556125_at	G patch domain containing 2	*GPATCH2*
5851	19,9	14,1	1,41	0,047333	0,442	237107_at	Protein kinase, interferon-inducible double stranded RNA dependent	*PRKRA*
2904	53,9	38,3	1,41	0,020424	0,383	232693_s_at	Zinc finger protein 395	*ZNF395*
111	204	145	1,41	0,000471	0,232	209225_x_at	Transportin 1	*TNPO1*
2067	102	72,3	1,41	0,013504	0,356	231873_at	Bone morphogenetic protein receptor, type II	*BMPR2*
1799	17,8	12,6	1,40	0,011579	0,351	1561181_at	AT rich interactive domain 5B (MRF1-like)	*ARID5B*
2541	441	314	1,40	0,017180	0,369	202028_s_at	Ribosomal protein L38	*RPL38*

Index	Mutated*	Normal*	FC	*P* value	FDR**	Probe set	Description	Gene symbol
5112	66,8	47,6	1,40	0,039357	0,421	200841_s_at	Glutamyl-prolyl-tRNA synthetase	*EPRS*
4709	164	117	1,40	0,035626	0,413	209348_s_at	v-maf musculoaponeurotic fibrosarcoma oncogene homolog (avian)	*MAF*
1013	17,1	12,2	1,40	0,005865	0,316	238693_at	Polyhomeotic homolog 3 (Drosophila)	*PHC3*
4426	21,0	15,0	1,40	0,033150	0,409	240758_at	ArfGAP with GTPase domain, ankyrin repeat and PH domain 1	*AGAP1*
2382	24,8	17,7	1,40	0,015884	0,364	222693_at	Fibronectin type III domain containing 3B	*FNDC3B*
1999	188	134	1,40	0,013063	0,355	203232_s_at	Ataxin 1	*ATXN1*
1584	18,5	13,2	1,40	0,009866	0,340	242814_at	Serpin peptidase inhibitor, clade B (ovalbumin), member 9	*SERPINB9*
1847	150	107	1,40	0,011890	0,352	209868_s_at	RNA binding motif, single stranded interacting protein 1	*RBMS1*
5339	177	126	1,40	0,041804	0,428	224595_at	Solute carrier family 44, member 1	*SLC44A1*
3361	41,4	29,6	1,40	0,023885	0,388	216235_s_at	Endothelin receptor type A	*EDNRA*
4038	4,99	3,56	1,40	0,029589	0,401	221008_s_at	Alanine-glyoxylate aminotransferase 2-like 1	*AGXT2L1*
5566	60,3	43,1	1,40	0,044312	0,435	229694_at	Bromodomain and WD repeat domain containing 2	*BRWD2*
601	147	105	1,40	0,003343	0,303	203656_at	FIG4 homolog (S. cerevisiae)	*FIG4*
3464	68,2	48,7	1,40	0,024821	0,392	218401_s_at	Zinc finger protein 281	*ZNF281*
347	105	75,2	1,40	0,001777	0,278	212604_at	Mitochondrial ribosomal protein S31	*MRPS31*
837	840	601	1,40	0,004801	0,313	210554_s_at	C-terminal binding protein 2	*CTBP2*
4431	7,34	5,24	1,40	0,033187	0,409	205871_at	Plasminogen-like B2	*PLGLB2*
2690	15,8	11,3	1,40	0,018445	0,375	228686_at	Hypothetical protein LOC644873	*FLJ33630*
1146	9,76	6,98	1,40	0,006731	0,321	207264_at	KDEL endoplasmic reticulum protein retention receptor 3	*KDELR3*
2641	57,0	40,7	1,40	0,018009	0,373	213056_at	FERM domain containing 4B	*FRMD4B*
5170	21,1	15,1	1,40	0,039831	0,421	209446_s_at	Chromosome 7 open reading frame 44	*C7orf44*
120	5,60	4,01	1,40	0,000550	0,240	239605_x_at	Transforming growth factor, beta receptor 1	*TGFBR1*
690	111	79,1	1,40	0,003949	0,308	212946_at	KIAA0564 protein	*KIAA0564*
6011	174	124	1,40	0,048880	0,445	214077_x_at	Meis homeobox 3 pseudogene 1	*MEIS3P1*
251	78,9	56,5	1,40	0,001161	0,253	1556000_s_a	BTB (POZ) domain containing 7	*BTBD7*
2806	20,7	14,8	1,40	0,019517	0,380	220638_s_at	Cas-Br-M (murine) ecotropic retroviral transforming sequence c	*CBLC*
4163	35,5	25,4	1,40	0,030772	0,404	217608_at	P18SRP protein	*P18SRP*
1106	163	117	1,40	0,006469	0,319	225243_s_at	Sarcolemma associated protein	*SLMAP*
2181	343	246	1,40	0,014427	0,361	204112_s_at	Histamine N-methyltransferase	*HNMT*
5523	437	313	1,40	0,043808	0,433	201259_s_at	Synaptophysin-like 1	*SYPL1*
2333	165	118	1,40	0,015567	0,364	229420_at	cDNA FLJ37566 fis, clone BRCOC2002085	-
3185	88,5	63,5	1,39	0,022587	0,387	223261_at	Polymerase (DNA directed) kappa	*POLK*
542	405	290	1,39	0,002907	0,293	203044_at	Carbohydrate (chondroitin) synthase 1	*CHSY1*
4327	74,5	53,4	1,39	0,032204	0,407	221123_x_at	Zinc finger protein 395	*ZNF395*
5949	17,3	12,4	1,39	0,048203	0,443	239449_at	Ankylosis, progressive homolog (mouse)	*ANKH*
2526	91,4	65,6	1,39	0,017053	0,369	206854_s_at	Mitogen-activated protein kinase kinase kinase 7	*MAP3K7*
3840	78,4	56,3	1,39	0,028045	0,399	223216_x_at	Huntington's disease gene regulatory region-binding protein 2	*PBF*
1603	57,5	41,3	1,39	0,009994	0,341	202984_s_at	BCL2-associated athanogene 5	*BAG5*
4531	181	130	1,39	0,034211	0,412	225093_at	Utrophin	*UTRN*
1176	670	481	1,39	0,006912	0,321	208991_at	Signal transducer and activator of transcription 3	*STAT3*
3256	18,2	13,1	1,39	0,023114	0,388	242405_at	Mastermind-like 2 (Drosophila)	*MAML2*
501	47,8	34,3	1,39	0,002664	0,289	226267_at	Jun dimerization protein 2	*JDP2*
4057	15,7	11,3	1,39	0,029765	0,401	242384_at	Cytoplasmic polyadenylation element binding protein 4	*CPEB4*
1149	125	90,1	1,39	0,006758	0,321	204716_at	Coiled-coil domain containing 6	*CCDC6*
5384	18,5	13,3	1,39	0,042241	0,429	222844_s_at	Serine racemase	*SRR*
3164	70,8	50,9	1,39	0,022458	0,387	212398_at	Radixin	*RDX*
3874	23,0	16,5	1,39	0,028290	0,399	231183_s_at	Jagged 1 (Alagille syndrome)	*JAG1*
4754	315	226	1,39	0,036020	0,414	206662_at	Glutaredoxin (thioltransferase)	*GLRX*
3413	60,7	43,7	1,39	0,024370	0,390	226682_at	RAR-related orphan receptor A	*RORA*
972	20,5	14,8	1,39	0,005641	0,315	205187_at	SMAD family member 5	*SMAD5*
3926	34,8	25,0	1,39	0,028702	0,399	211621_at	Androgen receptor	*AR*
2783	12,3	8,88	1,39	0,019334	0,380	240498_at	Ets variant 6	*ETV6*
2836	15,9	11,4	1,39	0,019798	0,382	239173_at	InaD-like (Drosophila)	*INADL*
2396	132	95,1	1,39	0,015987	0,365	224950_at	Prostaglandin F2 receptor negative regulator	*PTGFRN*
5359	786	566	1,39	0,042020	0,429	200897_s_at	Palladin, cytoskeletal associated protein	*PALLD*
2192	38,5	27,8	1,39	0,014508	0,361	224963_at	Solute carrier family 26 (sulfate transporter), member 2	*SLC26A2*
911	14,5	10,4	1,39	0,005250	0,313	241336_at	Transcribed locus	-

Index	Mutated*	Normal*	FC	*P* value	FDR**	Probe set	Description	Gene symbol
1562	160	115	1,39	0,009692	0,339	203748_x_at	RNA binding motif, single stranded interacting protein 1	*RBMS1*
805	507	365	1,39	0,004594	0,311	201260_s_at	Synaptophysin-like 1	*SYPL1*
5789	30,0	21,6	1,39	0,046653	0,441	235122_at	Immunodeficiency virus type I enhancer binding protein 3	*HIVEP3*
2540	78,1	56,3	1,39	0,017179	0,369	221045_s_at	Period homolog 3 (Drosophila)	*PER3*
1682	123	88,5	1,39	0,010633	0,345	203774_at	5-methyltetrahydrofolate-homocysteine methyltransferase	*MTR*
513	846	610	1,39	0,002737	0,291	200985_s_at	CD59 molecule, complement regulatory protein	*CD59*
4265	11,2	8,04	1,39	0,031737	0,406	1558447_at	Hypothetical protein LOC100128439	*LOC100128439*
610	8,38	6,04	1,39	0,003449	0,308	241360_at	Coiled-coil domain containing 15	*CCDC15*
1668	9,33	6,72	1,39	0,010537	0,345	229110_at	cDNA clone IMAGE:4794876	-
6071	18,3	13,2	1,39	0,049559	0,446	236696_at	U2-associated SR140 protein	*SR140*
4493	348	251	1,39	0,033779	0,411	202023_at	Ephrin-A1	*EFNA1*
2781	205	148	1,39	0,019305	0,380	204142_at	Enolase superfamily member 1	*ENOSF1*
3649	12,2	8,79	1,39	0,026377	0,395	1556935_at	Laminin, alpha 4	*LAMA4*
1427	96,8	69,8	1,39	0,008650	0,331	1558142_at	Trinucleotide repeat containing 6B	*TNRC6B*
628	164	118	1,39	0,003560	0,308	209106_at	Nuclear receptor coactivator 1	*NCOA1*
5034	169	122	1,39	0,038743	0,420	217739_s_at	Pre-B-cell colony enhancing factor 1	*PBEF1*
2788	29,6	21,4	1,39	0,019385	0,380	228482_at	CMT1A duplicated region transcript 4	*CDRT4*
2895	26,0	18,8	1,39	0,020334	0,383	229541_at	Homeobox containing 1	*HMBOX1*
642	877	633	1,39	0,003657	0,308	201009_s_at	Thioredoxin interacting protein	*TXNIP*
4514	28,4	20,5	1,38	0,034018	0,412	244310_at	RAR-related orphan receptor A	*RORA*
443	47,7	34,5	1,38	0,002360	0,288	213813_x_at	Ferritin, light polypeptide-like 1	*FTL*
3016	57,3	41,4	1,38	0,021240	0,385	223540_at	Poliovirus receptor-related 4	*PVRL4*
4378	166	120	1,38	0,032564	0,407	202211_at	ADP-ribosylation factor GTPase activating protein 3	*ARFGAP3*
4879	654	472	1,38	0,037272	0,418	200915_x_at	Kinectin 1 (kinesin receptor)	*KTN1*
2542	213	154	1,38	0,017180	0,369	211015_s_at	Heat shock 70kDa protein 4	*HSPA4*
4393	196	142	1,38	0,032723	0,407	201548_s_at	Jumonji, AT rich interactive domain 1B	*JARID1B*
4436	35,2	25,4	1,38	0,033216	0,409	233004_x_at	Nuclear factor I/A	*NFIA*
596	4,95	3,58	1,38	0,003281	0,301	240921_at	-	-
3005	104	75,3	1,38	0,021154	0,385	222633_at	Transducin (beta)-like 1X-linked receptor 1	*TBL1XR1*
4236	63,9	46,2	1,38	0,031397	0,405	219138_at	Ribosomal protein L14	*RPL14*
3088	48,4	35,0	1,38	0,021776	0,385	210224_at	Major histocompatibility complex, class I-related	*MR1*
5942	185	134	1,38	0,048160	0,443	225640_at	Hypothetical gene supported by AK091718	*LOC401504*
5624	8,13	5,88	1,38	0,044913	0,436	233720_at	Sorbin and SH3 domain containing 2	*SORBS2*
5267	22,1	16,0	1,38	0,040913	0,425	1569519_at	Neuroblastoma breakpoint family, member 1	*NBPF1*
4723	548	397	1,38	0,035729	0,414	203216_s_at	Myosin VI	*MYO6*
3198	106	76,6	1,38	0,022652	0,387	230618_s_at	HLA-B associated transcript 2-like 2	*BAT2L2*
3583	15,9	11,5	1,38	0,025869	0,394	1552309_a_a	Nexilin (F actin binding protein)	*NEXN*
3332	31,4	22,7	1,38	0,023657	0,388	226765_at	Spectrin, beta, non-erythrocytic 1	*SPTBN1*
2605	354	257	1,38	0,017689	0,371	204131_s_at	Forkhead box O3	*FOXO3*
2970	8,04	5,82	1,38	0,020866	0,384	238317_x_at	RNA binding motif, single stranded interacting protein 1	*RBMS1*
4135	134	96,9	1,38	0,030466	0,403	210621_s_at	RAS p21 protein activator (GTPase activating protein) 1	*RASA1*
2720	140	102	1,38	0,018682	0,376	212798_s_at	Ankyrin repeat and MYND domain containing 2	*ANKMY2*
1744	29,6	21,5	1,38	0,011145	0,348	205415_s_at	Ataxin 3	*ATXN3*
2321	10,6	7,67	1,38	0,015417	0,363	230375_at	Splicing factor, arginine/serine-rich 18	*SFRS18*
1563	14,5	10,5	1,38	0,009712	0,339	232710_at	cDNA FLJ11415 fis, clone HEMBA1000942	-
5546	26,5	19,3	1,38	0,044099	0,435	238311_at	KIAA0776 protein	*KIAA0776*
2238	98,8	71,7	1,38	0,014878	0,363	222692_s_at	Fibronectin type III domain containing 3B	*FNDC3B*
1294	231	167	1,38	0,007702	0,325	204616_at	Ubiquitin carboxyl-terminal esterase L3 (ubiquitin thiolesterase)	*UCHL3*
3501	110	79,9	1,38	0,025158	0,392	218693_at	Tetraspanin 15	*TSPAN15*
24	19,0	13,8	1,38	0,000100	0,201	205215_at	Ring finger protein 2	*RNF2*
600	154	112	1,38	0,003336	0,303	224827_at	Ubiquitin domain containing 2	*UBTD2*
2410	22,9	16,6	1,37	0,016102	0,365	229397_s_at	Glucocorticoid receptor DNA binding factor 1	*GRLF1*
218	11,9	8,67	1,37	0,000978	0,245	235567_at	RAR-related orphan receptor A	*RORA*
1806	56,2	40,9	1,37	0,011624	0,351	1555910_at	Pentatricopeptide repeat domain 2	*PTCD2*
2276	51,1	37,2	1,37	0,015132	0,363	231075_x_at	RAS association (RalGDS/AF-6) and pleckstrin homology domains 1	*RAPH1*
1209	185	135	1,37	0,007164	0,324	209817_at	Protein phosphatase 3 (formerly 2B), catalytic subunit, beta isoform	*PPP3CB*
5442	24,0	17,5	1,37	0,042909	0,431	205578_at	Receptor tyrosine kinase-like orphan receptor 2	*ROR2*

Index	Mutated*	Normal*	FC	*P* value	FDR**	Probe set	Description	Gene symbol
3421	23,0	16,7	1,37	0,024465	0,391	1555240_s_a	Guanine nucleotide binding protein (G protein), gamma 12	*GNG12*
1895	256	186	1,37	0,012316	0,355	212410_at	EF-hand domain family, member A1	*EFHA1*
4419	132	96,0	1,37	0,033065	0,409	212917_x_at	RecQ protein-like (DNA helicase Q1-like)	*RECQL*
5017	4,89	3,56	1,37	0,038592	0,420	238103_at	CDNA FLJ37936 fis, clone CTONG2005468	-
4475	38,6	28,1	1,37	0,033648	0,411	234192_s_at	G kinase anchoring protein 1	*GKAP1*
1568	20,5	14,9	1,37	0,009751	0,340	1554004_a_a	Rho-guanine nucleotide exchange factor	*RGNEF*
656	116	84,5	1,37	0,003784	0,308	212936_at	Family with sequence similarity 172, member A	*C5orf21*
2754	138	100	1,37	0,019070	0,378	225674_at	B-cell receptor-associated protein 29	*BCAP29*
4778	107	78,2	1,37	0,036230	0,414	204518_s_at	Peptidylprolyl isomerase C (cyclophilin C)	*PPIC*
5939	21,8	15,9	1,37	0,048147	0,443	1569142_at	Tripartite motif-containing 13	*TRIM13*
2759	256	187	1,37	0,019144	0,379	225273_at	WWC family member 3	*WWC3*
2564	49,8	36,3	1,37	0,017391	0,371	227733_at	Transmembrane protein 63C	*TMEM63C*
750	117	85,4	1,37	0,004249	0,308	209647_s_at	Suppressor of cytokine signaling 5	*SOCS5*
4800	20,2	14,7	1,37	0,036425	0,415	230102_at	Ets variant gene 5 (ets-related molecule)	*ETV5*
2008	693	506	1,37	0,013093	0,355	212600_s_at	Ubiquinol-cytochrome c reductase core protein II	*UQCRC2*
1097	208	152	1,37	0,006432	0,319	204690_at	Syntaxin 8	*STX8*
4510	17,2	12,5	1,37	0,034001	0,412	242188_at	Protein tyrosine phosphatase, receptor type, G	*PTPRG*
636	7,59	5,54	1,37	0,003619	0,308	231109_at	CUG triplet repeat, RNA binding protein 2	*CUGBP2*
4277	26,9	19,6	1,37	0,031784	0,406	237383_at	Transcribed locus	-
2601	25,3	18,4	1,37	0,017665	0,371	214493_s_at	InaD-like (Drosophila)	*INADL*
4303	24,4	17,8	1,37	0,032033	0,407	1554919_s_a	Chromosome 7 open reading frame 63	*C7orf63*
4025	38,5	28,1	1,37	0,029474	0,400	203836_s_at	Mitogen-activated protein kinase kinase kinase 5	*MAP3K5*
2252	32,0	23,4	1,37	0,014997	0,363	236754_at	Protein phosphatase 1, regulatory (inhibitor) subunit 2	*PPP1R2*
333	307	225	1,37	0,001675	0,274	202808_at	Chromosome 10 open reading frame 26	*C10orf26*
4121	30,5	22,3	1,37	0,030300	0,402	205395_s_at	MRE11 meiotic recombination 11 homolog A (S. cerevisiae)	*MRE11A*
5320	423	309	1,37	0,041550	0,427	217869_at	Hydroxysteroid (17-beta) dehydrogenase 12	*HSD17B12*
1136	60,8	44,5	1,37	0,006643	0,319	226787_at	Zinc finger protein 18	*ZNF18*
3265	156	114	1,37	0,023193	0,388	218594_at	HEAT repeat containing 1	*HEATR1*
5827	30,2	22,0	1,37	0,047078	0,442	222858_s_at	Dual adaptor of phosphotyrosine and 3-phosphoinositides	*DAPP1*
4001	113	82,6	1,37	0,029287	0,400	221763_at	Jumonji domain containing 1C	*JMJD1C*
5244	159	116	1,37	0,040599	0,423	212397_at	Radixin	*RDX*
4425	18,7	13,7	1,37	0,033119	0,409	244464_at	Ubiquitin-conjugating enzyme E2E 2 (UBC4/5 homolog, yeast)	*UBE2E2*
2053	14,4	10,5	1,37	0,013345	0,355	242243_at	TATA element modulatory factor 1	*TMF1*
3940	92,2	67,5	1,37	0,028764	0,399	219757_s_at	Chromosome 14 open reading frame 101	*C14orf101*
3295	203	149	1,37	0,023478	0,388	212420_at	E74-like factor 1 (ets domain transcription factor)	*ELF1*
3157	31,6	23,1	1,37	0,022409	0,387	204225_at	Histone deacetylase 4	*HDAC4*
3310	42,9	31,4	1,37	0,023546	0,388	238476_at	Adult retina protein	*C5orf41*
1984	27,4	20,1	1,37	0,012953	0,355	215239_x_at	Zinc finger protein 273	*ZNF273*
5934	6,53	4,78	1,37	0,048111	0,443	241708_at	Dedicator of cytokinesis 1	*DOCK1*
1101	97,1	71,1	1,37	0,006456	0,319	1557370_s_a	MYC binding protein 2	*MYCBP2*
5386	39,2	28,7	1,37	0,042245	0,429	226380_at	Protein tyrosine phosphatase, non-receptor type 21	*PTPN21*
5136	412	302	1,37	0,039515	0,421	200883_at	Ubiquinol-cytochrome c reductase core protein II	*UQCRC2*
5428	12,4	9,11	1,37	0,042706	0,430	1554741_s_a	Fibroblast growth factor 7 (keratinocyte growth factor)	*FGF7*
4147	45,9	33,7	1,37	0,030601	0,403	228251_at	UBX domain containing 1	*UBXD1*
1387	258	189	1,36	0,008348	0,329	217858_s_at	Armadillo repeat containing, X-linked 3	*ARMCX3*
2272	11,1	8,17	1,36	0,015090	0,363	1558184_s_a	Zinc finger protein 17	*ZNF17*
1460	96,0	70,4	1,36	0,008919	0,334	204703_at	Intraflagellar transport 88 homolog (Chlamydomonas)	*IFT88*
4159	72,9	53,5	1,36	0,030734	0,404	211944_at	BAT2 domain containing 1	*BAT2D1*
2174	61,0	44,8	1,36	0,014364	0,361	226203_at	cDNA clone IMAGE:5299888	-
5582	121	88,8	1,36	0,044509	0,436	206695_x_at	Zinc finger protein 43	*ZNF43*
5116	49,3	36,2	1,36	0,039390	0,421	227405_s_at	Frizzled homolog 8 (Drosophila)	*FZD8*
3582	63,8	46,9	1,36	0,025868	0,394	216905_s_at	Suppression of tumorigenicity 14 (colon carcinoma)	*ST14*
2652	20,8	15,3	1,36	0,018082	0,373	227987_at	Vacuolar protein sorting 13 homolog A (S. cerevisiae)	*VPS13A*
60	504	370	1,36	0,000226	0,201	224837_at	Forkhead box P1	*FOXP1*
5701	39,3	28,8	1,36	0,045646	0,438	209119_x_at	Nuclear receptor subfamily 2, group F, member 2	*NR2F2*
667	24,2	17,8	1,36	0,003838	0,308	244813_at	RAD51-like 1 (S. cerevisiae)	*RAD51L1*
6094	220	161	1,36	0,049743	0,446	209444_at	RAP1, GTP-GDP dissociation stimulator 1	*RAP1GDS1*

Index	Mutated*	Normal*	FC	*P* value	FDR**	Probe set	Description	Gene symbol
4939	60,7	44,6	1,36	0,037891	0,419	241425_at	Nucleoporin like 1	*NUPL1*
4818	211	155	1,36	0,036618	0,416	212423_at	Zinc finger, CCHC domain containing 24	*ZCCHC24*
3505	58,8	43,2	1,36	0,025182	0,392	242669_at	Ubiquitin-fold modifier 1	*UFM1*
3029	66,7	49,0	1,36	0,021321	0,385	204635_at	Ribosomal protein S6 kinase, 90kDa, polypeptide 5	*RPS6KA5*
447	29,5	21,7	1,36	0,002391	0,288	221555_x_at	CDC14 cell division cycle 14 homolog B (S. cerevisiae)	*CDC14B*
4245	14,1	10,4	1,36	0,031505	0,406	205222_at	L-3-hydroxyacyl-CoA dehydrogenase	*EHHADH*
5360	9,21	6,78	1,36	0,042032	0,429	243041_s_at	RNA binding motif, single stranded interacting protein	*RBMS3*
1874	29,3	21,6	1,36	0,012125	0,354	242878_at	-	-
4558	30,0	22,1	1,36	0,034449	0,412	229654_at	Zinc finger protein 44	*ZNF44*
1532	141	104	1,36	0,009477	0,337	221787_at	Chromosome 6 open reading frame 120	*C6orf120*
3965	42,0	30,9	1,36	0,028965	0,399	230886_at	Transcribed locus	-
88	11,8	8,65	1,36	0,000330	0,201	236849_at	Vesicle transport through interaction with t-SNAREs homolog 1A	*VTI1A*
3132	39,2	28,9	1,36	0,022214	0,387	33494_at	Electron-transferring-flavoprotein dehydrogenase	*ETFDH*
4790	35,7	26,3	1,36	0,036330	0,415	202669_s_at	Ephrin-B2	*EFNB2*
317	236	174	1,36	0,001588	0,272	203704_s_at	RAS responsive element binding protein 1	*RREB1*
5952	20,4	15,0	1,36	0,048288	0,444	220840_s_at	Chromosome 1 open reading frame 112	*C1orf112*
688	35,9	26,5	1,36	0,003940	0,308	208876_s_at	p21 (CDKN1A)-activated kinase 2	*PAK2*
2682	21,3	15,7	1,36	0,018363	0,374	242722_at	LIM domain 7	*LMO7*
798	12,0	8,87	1,36	0,004555	0,311	243469_at	E74-like factor 2 (ets domain transcription factor)	*ELF2*
3478	6,79	5,00	1,36	0,024976	0,392	240866_at	LIM domain containing preferred translocation partner in lipoma	*LPP*
1214	29,9	22,0	1,36	0,007213	0,324	243772_at	Serologically defined colon cancer antigen 8	*SDCCAG8*
789	43,9	32,4	1,36	0,004507	0,311	1554003_at	Rho-guanine nucleotide exchange factor	*RGNEF*
3221	5,28	3,89	1,36	0,022830	0,387	239370_at	cDNA clone ZE01A04	-
4855	22,4	16,5	1,36	0,037023	0,417	242431_at	Ubiquitin-conjugating enzyme E2-binding protein 1	*ARIH1*
2485	56,8	41,9	1,36	0,016748	0,367	209105_at	Nuclear receptor coactivator 1	*NCOA1*
2033	82,9	61,1	1,36	0,013238	0,355	201746_at	Tumor protein p53 (Li-Fraumeni syndrome)	*TP53*
3111	415	306	1,36	0,021974	0,386	202743_at	Phosphoinositide-3-kinase, regulatory subunit 3 (p55, gamma)	*PIK3R3*
3605	26,9	19,8	1,36	0,026000	0,394	233977_at	Growth regulation by estrogen in breast cancer-like	*KIAA1772*
2465	136	100	1,36	0,016563	0,367	218379_at	RNA binding motif protein 7	*RBM7*
5161	69,2	51,0	1,36	0,039739	0,421	207606_s_at	Rho GTPase activating protein 12	*ARHGAP12*
773	139	103	1,36	0,004427	0,311	202305_s_at	Fasciculation and elongation protein zeta 2 (zygin II)	*FEZ2*
239	162	119	1,36	0,001109	0,250	203614_at	UTP14, U3 small nucleolar ribonucleoprotein, homolog C (yeast)	*UTP14C*
4390	87,1	64,3	1,36	0,032708	0,407	1569057_s_a	Melanoma inhibitory activity family, member 3	*MIA3*
617	14,3	10,6	1,36	0,003494	0,308	241709_s_at	Dedicator of cytokinesis 1	*DOCK1*
5599	63,4	46,8	1,35	0,044658	0,436	221946_at	Chromosome 9 open reading frame 116	*C9orf116*
1488	138	102	1,35	0,009179	0,336	233952_s_at	Zinc finger protein 295	*ZNF295*
4219	26,3	19,4	1,35	0,031255	0,405	231403_at	Triple functional domain (PTPRF interacting)	*TRIO*
4627	21,7	16,1	1,35	0,034900	0,412	213623_at	Kinesin family member 3A	*KIF3A*
865	309	228	1,35	0,004995	0,313	218229_s_at	Pogo transposable element with KRAB domain	*POGK*
4290	558	412	1,35	0,031890	0,406	202351_at	Vitronectin receptor subunit alpha (antigen CD51)	*ITGAV*
1905	130	96,3	1,35	0,012386	0,355	201918_at	Solute carrier family 25, member 36	*SLC25A36*
2854	11,9	8,76	1,35	0,020010	0,383	232110_at	Polypeptide N-acetylgalactosaminyltransferase 5	*GALNT5*
4200	12,1	8,93	1,35	0,031059	0,404	233087_at	F-box and leucine-rich repeat protein 17	*FBXL17*
3455	66,4	49,1	1,35	0,024747	0,392	212858_at	Progestin and adipoQ receptor family member IV	*PAQR4*
5883	150	111	1,35	0,047590	0,442	201325_s_at	Epithelial membrane protein 1	*EMP1*
2304	72,5	53,6	1,35	0,015297	0,363	233936_s_at	Gametogenetin binding protein 2	*GGNBP2*
2480	98,6	73,0	1,35	0,016719	0,367	227413_at	Ubiquitin-like domain containing CTD phosphatase 1	*UBLCP1*
3273	78,5	58,1	1,35	0,023279	0,388	1554351_a_a	TIP41, TOR signaling pathway regulator-like (S. cerevisiae)	*TIPRL*
2985	45,0	33,3	1,35	0,020986	0,384	214175_x_at	PDZ and LIM domain 4	*PDLIM4*
623	84,1	62,3	1,35	0,003529	0,308	229009_at	SIX homeobox 5	*SIX5*
1463	9,71	7,19	1,35	0,008945	0,334	1569796_s_a	Attractin-like 1	*ATRNL1*
6114	407	301	1,35	0,049896	0,446	201876_at	Paraoxonase 2	*PON2*
1786	256	189	1,35	0,011410	0,349	209579_s_at	Methyl-CpG binding domain protein 4	*MBD4*
5600	106	78,6	1,35	0,044661	0,436	213025_at	THUMP domain containing 1	*THUMPD1*
5173	84,2	62,4	1,35	0,039846	0,421	226647_at	Transmembrane protein 25	*TMEM25*
2358	9,54	7,07	1,35	0,015729	0,364	216147_at	Septin 11	*SEPT11*
2122	19,6	14,6	1,35	0,013955	0,359	207950_s_at	Ankyrin 3, node of Ranvier (ankyrin G)	*ANK3*

Index	Mutated*	Normal*	FC	*P* value	FDR**	Probe set	Description	Gene symbol
4721	96,1	71,3	1,35	0,035710	0,413	226541_at	F-box protein 30	*FBXO30*
1300	471	349	1,35	0,007742	0,325	200984_s_at	CD59 molecule, complement regulatory protein	*CD59*
1086	220	163	1,35	0,006364	0,319	209107_x_at	Nuclear receptor coactivator 1	*NCOA1*
2328	163	121	1,35	0,015505	0,364	226247_at	Pleckstrin homology domain containing, family A member 1	*PLEKHA1*
3595	21,0	15,6	1,35	0,025928	0,394	222354_at	F11 receptor	*FR11*
4848	54,7	40,6	1,35	0,036936	0,417	229173_at	KIAA1715 protein	*KIAA1715*
2586	217	161	1,35	0,017543	0,371	224955_at	TEA domain family member 1 (SV40 transcriptional enhancer factor)	*TEAD1*
2420	7,43	5,52	1,35	0,016179	0,365	1553672_at	Enabled homolog (Drosophila)	*ENAH*
5114	24,5	18,2	1,35	0,039378	0,421	220918_at	Chromosome 21 open reading frame 96	*C21orf96*
2644	42,7	31,7	1,35	0,018026	0,373	218006_s_at	Zinc finger protein 22 (KOX 15)	*ZNF22*
3670	13,0	9,64	1,35	0,026522	0,395	233224_at	Karyopherin alpha 3 (importin alpha 4)	*KPNA3*
3755	23,7	17,6	1,34	0,027293	0,397	218833_at	sterile alpha motif and leucine zipper containing kinase AZK	*ZAK*
930	25,0	18,6	1,34	0,005346	0,313	223713_at	Radial spoke head 3 homolog (Chlamydomonas)	*RSPH3*
2608	185	138	1,34	0,017707	0,371	203173_s_at	Esophageal cancer associated protein	*MGC16824*
4214	190	141	1,34	0,031226	0,405	201910_at	FERM, RhoGEF (ARHGEF) and pleckstrin domain protein 1	*FARP1*
4653	54,4	40,5	1,34	0,035147	0,413	206240_s_at	Zinc finger protein 136	*ZNF136*
473	106	78,9	1,34	0,002514	0,289	1553103_at	Nuclear transcription factor, X-box binding 1	*NFX1*
4139	111	82,5	1,34	0,030549	0,403	217952_x_at	PHD finger protein 3	*PHF3*
3243	82,9	61,7	1,34	0,022996	0,388	205405_at	Semaphorin 5A	*SEMA5A*
3398	605	451	1,34	0,024247	0,390	202442_at	Adaptor-related protein complex 3, sigma 1 subunit	*AP3S1*
1593	2123	1581	1,34	0,009902	0,340	215001_s_at	Glutamate-ammonia ligase (glutamine synthetase)	*GLUL*
2027	435	324	1,34	0,013216	0,355	208992_s_at	Signal transducer and activator of transcription 3	*STAT3*
3484	268	200	1,34	0,025016	0,392	200842_s_at	Glutamyl-prolyl-tRNA synthetase	*EPRS*
4810	38,9	29,0	1,34	0,036541	0,415	218766_s_at	Tryptophanyl tRNA synthetase 2, mitochondrial	*WARS2*
4762	221	165	1,34	0,036100	0,414	211948_x_at	BAT2 domain containing 1	*BAT2D1*
3254	178	133	1,34	0,023110	0,388	202180_s_at	Major vault protein	*MVP*
582	205	153	1,34	0,003194	0,300	212742_at	Zinc finger protein 364	*ZNF364*
4699	10,1	7,57	1,34	0,035478	0,413	222180_at	v-yes-1 Yamaguchi sarcoma viral oncogene homolog 1	*YES1*
3257	73,2	54,6	1,34	0,023129	0,388	201083_s_at	BCL2-associated transcription factor 1	*BCLAF1*
2508	144	108	1,34	0,016854	0,367	203238_s_at	Notch homolog 3 (Drosophila)	*NOTCH3*
1383	49,5	36,9	1,34	0,008328	0,329	227438_at	Alpha-kinase 1	*ALPK1*
360	55,7	41,5	1,34	0,001852	0,281	219362_at	MAK10 homolog, amino-acid N-acetyltransferase subunit, (S.	*MAK10*
2176	28,9	21,5	1,34	0,014396	0,361	1568594_s_a	Tripartite motif-containing 52	*TRIM52*
5472	7,55	5,63	1,34	0,043265	0,432	214524_at	Growth hormone releasing hormone	*GHRH*
3943	139	104	1,34	0,028790	0,399	221761_at	Adenylosuccinate synthase	*ADSS*
4459	13,3	9,95	1,34	0,033508	0,411	236704_at	Phosphodiesterase 4D interacting protein	*PDE4DIP*
1564	50,8	37,9	1,34	0,009715	0,339	221496_s_at	Transducer of ERBB2, 2	*TOB2*
5315	6,67	4,98	1,34	0,041471	0,427	207120_at	Zinc finger protein 667	*ZNF667*
2701	11,9	8,91	1,34	0,018527	0,375	1558815_at	Sorbin and SH3 domain containing 2	*SORBS2*
5371	58,1	43,4	1,34	0,042156	0,429	201888_s_at	Interleukin 13 receptor, alpha 1	*IL13RA1*
662	9,18	6,86	1,34	0,003823	0,308	1556235_at	cDNA FLJ34369 fis, clone FEBRA2017098	-
2171	186	139	1,34	0,014359	0,361	221664_s_at	F11 receptor	*F11R*
1808	445	333	1,34	0,011663	0,351	201413_at	Hydroxysteroid (17-beta) dehydrogenase 4	*HSD17B4*
3720	83,0	62,0	1,34	0,027005	0,397	219156_at	synaptojanin 2 binding protein	*SYNJ2BP*
3931	87,3	65,2	1,34	0,028738	0,399	206829_x_at	Zinc finger protein 430	*ZNF430*
5020	28,4	21,2	1,34	0,038621	0,420	223730_at	Glypican 6	*GPC6*
4715	1502	1123	1,34	0,035668	0,413	201666_at	TIMP metallopeptidase inhibitor 1	*TIMP1*
4621	46,9	35,1	1,34	0,034870	0,412	202516_s_at	Discs, large homolog 1 (Drosophila)	*DLG1*
4264	14,3	10,7	1,34	0,031728	0,406	236983_at	Transmembrane channel-like 5	*TMC5*
4698	226	169	1,34	0,035476	0,413	202656_s_at	SERTA domain containing 2	*SERTAD2*
2022	523	391	1,34	0,013188	0,355	224891_at	Forkhead box O3	*FOXO3*
2602	27,2	20,3	1,34	0,017666	0,371	212027_at	RNA binding motif protein 25	*RBM25*
6098	207	155	1,34	0,049784	0,446	223272_s_at	Chromosome 1 open reading frame 57	*C1orf57*
4477	242	181	1,34	0,033671	0,411	223223_at	ARV1 homolog (S. cerevisiae)	*ARV1*
6069	28,2	21,1	1,34	0,049529	0,446	243759_at	Splicing factor, arginine/serine-rich 15	*SFRS15*
620	16,2	12,1	1,34	0,003506	0,308	232206_at	Unc-51-like kinase 4 (C. elegans)	*ULK4*
2362	61,9	46,4	1,34	0,015765	0,364	206554_x_at	SET domain and mariner transposase fusion gene	*SETMAR*

Index	Mutated*	Normal*	FC	*P* value	FDR**	Probe set	Description	Gene symbol
4060	83,6	62,6	1,34	0,029796	0,401	229810_at	Transcribed locus	-
2189	39,4	29,5	1,34	0,014493	0,361	213494_s_at	YY1 transcription factor	*YY1*
2223	77,6	58,1	1,34	0,014765	0,363	240044_x_at	Trinucleotide repeat containing 6B	*TNRC6B*
4626	6,06	4,54	1,34	0,034891	0,412	235547_at	NEDD4 binding protein 2-like 2	*N4BP2L2*
3473	84,8	63,5	1,34	0,024915	0,392	214264_s_at	Chromosome 14 open reading frame 143	*C14orf143*
1197	34,4	25,7	1,33	0,007066	0,323	204180_s_at	Zinc finger and BTB domain containing 43	*ZBTB43*
1157	182	137	1,33	0,006801	0,321	224857_s_at	Polymerase (RNA) I polypeptide D, 16kDa	*POLR1D*
1212	305	228	1,33	0,007185	0,324	201133_s_at	Praja 2, RING-H2 motif containing	*PJA2*
281	93,4	70,0	1,33	0,001372	0,263	54632_at	Thyroid adenoma associated	*THADA*
4820	136	102	1,33	0,036650	0,416	202052_s_at	Retinoic acid induced 14	*RAI14*
4026	208	156	1,33	0,029485	0,400	214693_x_at	Neuroblastoma breakpoint family, member 14	*NBPF14*
2097	30,6	22,9	1,33	0,013739	0,358	232097_at	TOX high mobility group box family member 4	*TOX4*
6000	49,1	36,8	1,33	0,048771	0,444	216835_s_at	Docking protein 1, 62kDa (downstream of tyrosine kinase 1)	*DOK1*
2874	52,8	39,6	1,33	0,020184	0,383	207688_s_at	Inhibin, beta C	*INHBC*
4127	49,9	37,4	1,33	0,030364	0,402	235938_at	Transcribed locus	-
4366	114	85,5	1,33	0,032479	0,407	213341_at	Fem-1 homolog c (C. elegans)	*FEM1C*
3385	131	98,4	1,33	0,024097	0,389	218578_at	Hyperparathyroidism 2 protein	*CDC73*
1576	23,2	17,4	1,33	0,009792	0,340	206429_at	Coagulation factor II (thrombin) receptor-like 1	*F2RL1*
3524	11,9	8,96	1,33	0,025289	0,392	210365_at	Runt-related transcription factor 1 (aml1 oncogene)	*RUNX1*
5571	99,3	74,5	1,33	0,044377	0,435	217985_s_at	Bromodomain adjacent to zinc finger domain, 1A	*BAZ1A*
5667	212	159	1,33	0,045223	0,436	214305_s_at	Splicing factor 3b, subunit 1, 155kDa	*SF3B1*
2017	36,0	27,0	1,33	0,013160	0,355	202972_s_at	Family with sequence similarity 13, member A1	*FAM13A1*
1624	318	239	1,33	0,010206	0,344	212463_at	CD59 molecule, complement regulatory protein	*CD59*
1005	520	391	1,33	0,005813	0,316	201251_at	Pyruvate kinase, muscle	*PKM2*
2183	105	78,6	1,33	0,014447	0,361	222754_at	tRNA nucleotidyl transferase, CCA-adding, 1	*TRNT1*
5305	1046	786	1,33	0,041390	0,426	201403_s_at	Microsomal glutathione S-transferase 3	*MGST3*
4047	69,0	51,8	1,33	0,029663	0,401	221277_s_at	Pseudouridylate synthase 3	*PUS3*
442	335	252	1,33	0,002345	0,288	214170_x_at	Fumarate hydratase	*FH*
3949	69,1	52,0	1,33	0,028839	0,399	1559038_at	Septin 2	*SEPT2*
357	83,5	62,8	1,33	0,001832	0,280	225757_s_at	Calmin (calponin-like, transmembrane)	*CLMN*
1884	4,78	3,59	1,33	0,012232	0,355	242033_at	Ring finger protein 180	*RNF180*
420	203	152	1,33	0,002205	0,286	218198_at	DEAH (Asp-Glu-Ala-His) box polypeptide 32	*DHX32*
1015	225	169	1,33	0,005873	0,316	204313_s_at	cAMP responsive element binding protein 1	*CREB1*
2338	46,9	35,3	1,33	0,015605	0,364	231016_s_at	Aryl hydrocarbon receptor nuclear translocator	*ARNT*
3400	124	93,6	1,33	0,024254	0,390	222667_s_at	Ash1 (absent, small, or homeotic)-like (Drosophila)	*ASH1L*
3648	55,8	42,0	1,33	0,026358	0,395	230734_x_at	Striatin, calmodulin binding protein	*STRN*
3298	118	88,6	1,33	0,023502	0,388	216246_at	Ribosomal protein S20	*RPS20*
5196	11,1	8,32	1,33	0,040056	0,421	237491_at	Myosin, heavy chain 10, non-muscle	*MYH10*
3673	159	120	1,33	0,026526	0,395	221589_s_at	Aldehyde dehydrogenase 6 family, member A1	*ALDH6A1*
4968	14,8	11,2	1,33	0,038109	0,419	208200_at	Interleukin 1, alpha	*IL1A*
241	529	398	1,33	0,001111	0,250	224838_at	Forkhead box P1	*FOXP1*
4215	58,5	44,0	1,33	0,031226	0,405	204085_s_at	Ceroid-lipofuscinosis, neuronal 5	*CLN5*
1121	36,6	27,6	1,33	0,006545	0,319	222599_s_at	Neuron navigator 2	*NAV2*
2190	29,8	22,4	1,33	0,014502	0,361	243790_at	Zinc finger protein 585A	*ZNF585A*
4744	28,7	21,6	1,33	0,035936	0,414	205809_s_at	Wiskott-Aldrich syndrome-like	*WASL*
5223	55,4	41,8	1,33	0,040397	0,423	228466_at	GA binding protein transcription factor, beta subunit 2	*GABPB2*
5051	121	91,5	1,33	0,038920	0,420	209515_s_at	RAB27A, member RAS oncogene family	*RAB27A*
1901	71,3	53,8	1,33	0,012362	0,355	203329_at	Protein tyrosine phosphatase, receptor type, M	*PTPRM*
1996	9,36	7,06	1,33	0,013038	0,355	237133_at	Sterile alpha motif and leucine zipper containing kinase AZK	*ZAK*
4347	118	89,1	1,33	0,032377	0,407	238002_at	Golgi integral membrane protein 4	*GOLIM4*
4166	160	121	1,33	0,030780	0,404	202040_s_at	Jumonji, AT rich interactive domain 1A	*JARID1A*
653	91,7	69,2	1,32	0,003728	0,308	221771_s_at	M-phase phosphoprotein, mpp8	*HSMPP8*
1534	63,7	48,1	1,32	0,009505	0,337	219348_at	SNARE-like tail-anchored protein 1 homolog	*USE1*
5194	197	149	1,32	0,040040	0,421	212044_s_at	Ribosomal protein L27a	*RPL27A*
3509	209	158	1,32	0,025212	0,392	226447_at	Ash1 (absent, small, or homeotic)-like (Drosophila)	*ASH1L*
228	73,2	55,3	1,32	0,001054	0,250	220212_s_at	Thyroid adenoma associated	*THADA*
1579	95,6	72,2	1,32	0,009820	0,340	225769_at	Component of oligomeric golgi complex 6	*COG6*

Index	Mutated*	Normal*	FC	*P* value	FDR**	Probe set	Description	Gene symbol
1185	42,8	32,3	1,32	0,006976	0,322	215153_at	Nitric oxide synthase 1 (neuronal) adaptor protein	*NOS1AP*
2519	35,1	26,5	1,32	0,016999	0,369	213575_at	Transformer-2 alpha	*TRA2A*
4716	6,36	4,81	1,32	0,035671	0,413	1569348_at	TPTE pseudogene	*psiTPTE22*
1461	95,4	72,1	1,32	0,008929	0,334	225507_at	Splicing factor, arginine/serine-rich 18	*SFRS18*
1156	9,67	7,31	1,32	0,006789	0,321	227766_at	Ligase IV, DNA, ATP-dependent	*LIG4*
4911	69,4	52,4	1,32	0,037558	0,418	215221_at	Forkhead box P1	*FOXP1*
2493	8,83	6,68	1,32	0,016799	0,367	241026_at	ADAM metallopeptidase domain 12	*ADAM12*
3175	149	113	1,32	0,022527	0,387	222763_s_at	WD repeat domain 33	*WDR33*
3837	91,7	69,3	1,32	0,028033	0,399	204312_x_at	cAMP responsive element binding protein 1	*CREB1*
3553	59,2	44,8	1,32	0,025615	0,394	243931_at	CD58 molecule	*CD58*
3066	42,4	32,1	1,32	0,021616	0,385	222487_s_at	Ribosomal protein S27-like	*RPS27L*
4162	10,4	7,88	1,32	0,030770	0,404	1560171_at	SNF2 histone linker PHD RING helicase	*SHPRH*
4674	22,8	17,3	1,32	0,035295	0,413	1554438_at	KIAA1217 protein	*KIAA1217*
783	28,0	21,2	1,32	0,004489	0,311	224023_s_at	Chromosome 3 open reading frame 10	*C3orf10*
5765	41,4	31,4	1,32	0,046330	0,439	216521_s_at	BRCA1/BRCA2-containing complex, subunit 3	*BRCC3*
1288	134	101	1,32	0,007671	0,325	218204_s_at	FYVE and coiled-coil domain containing 1	*FYCO1*
4728	128	97,1	1,32	0,035787	0,414	213642_at	Ribosomal protein L27	*RPL27*
4583	9,00	6,82	1,32	0,034643	0,412	207345_at	Follistatin	*FST*
2129	60,4	45,8	1,32	0,014003	0,359	214513_s_at	cAMP responsive element binding protein 1	*CREB1*
1218	121	92,0	1,32	0,007243	0,324	230440_at	Zinc finger protein 469	*ZNF469*
3325	822	623	1,32	0,023623	0,388	208662_s_at	Tetratricopeptide repeat domain 3	*TTC3*
182	6,02	4,56	1,32	0,000839	0,244	239539_at	NIMA (never in mitosis gene a)-related kinase 3	*NEK3*
1181	15,0	11,3	1,32	0,006957	0,322	1561689_at	cDNA DKFZp547G1518	-
2977	20,0	15,2	1,32	0,020921	0,384	243050_at	Peripheral myelin protein 22	*PMP22*
5448	51,0	38,6	1,32	0,042968	0,431	204645_at	Cyclin T2	*CCNT2*
1928	838	636	1,32	0,012570	0,355	218205_s_at	MAP kinase interacting serine/threonine kinase 2	*MKNK2*
4237	13,5	10,3	1,32	0,031402	0,405	242576_x_at	NEDD4 binding protein 2-like 2	*N4BP2L2*
3856	322	244	1,32	0,028153	0,399	218242_s_at	suppressor of variegation 4-20 homolog 1 (Drosophila)	*SUV420H1*
609	14,1	10,7	1,32	0,003435	0,308	236557_at	Zinc finger and BTB domain containing 38	*ZBTB38*
3342	6,90	5,24	1,32	0,023724	0,388	236930_at	Numb homolog (Drosophila)	*NUMB*
3069	7,22	5,48	1,32	0,021663	0,385	1558199_at	Fibronectin 1	*FN1*
4675	65,0	49,3	1,32	0,035307	0,413	202699_s_at	Transmembrane protein 63A	*TMEM63A*
6047	122	92,8	1,32	0,049281	0,445	227447_at	Superkiller viralicidic activity 2-like 2 (S. cerevisiae)	*SKIV2L2*
544	1223	928	1,32	0,002926	0,294	217802_s_at	Nuclear casein kinase and cyclin-dependent kinase substrate 1	*NUCKS1*
4833	239	181	1,32	0,036829	0,417	218618_s_at	Fibronectin type III domain containing 3B	*FNDC3B*
4430	24,1	18,3	1,32	0,033173	0,409	232716_at	cDNA FLJ11971 fis, clone HEMBB1001208	-
2011	1441	1094	1,32	0,013111	0,355	228222_at	Protein phosphatase 1, catalytic subunit, beta isoform	*PPP1CB*
1268	364	277	1,32	0,007544	0,325	224571_at	Interferon regulatory factor 2 binding protein 2	*IRF2BP2*
2999	34,2	26,0	1,32	0,021113	0,385	230395_at	Methyltransferase like 9	*METTL9*
3021	178	135	1,32	0,021274	0,385	214004_s_at	Vestigial like 4 (Drosophila)	*VGLL4*
3861	116	88,3	1,32	0,028187	0,399	202778_s_at	Zinc finger, MYM-type 2	*ZMYM2*
320	169	129	1,31	0,001600	0,272	225695_at	Chromosome 2 open reading frame 18	*C2orf18*
2708	10,9	8,26	1,31	0,018567	0,375	238967_at	Claudin 1	*CLDN1*
3709	137	104	1,31	0,026822	0,395	225181_at	AT rich interactive domain 1B (SWI1-like)	*ARID1B*
3507	64,4	49,0	1,31	0,025199	0,392	220992_s_at	Chromosome 1 open reading frame 25	*C1orf25*
3776	139	106	1,31	0,027447	0,397	224097_s_at	F11 receptor	*F11R*
2006	84,0	63,9	1,31	0,013086	0,355	223211_at	2-hydroxyacyl-CoA lyase 1	*HACL1*
2845	74,9	57,1	1,31	0,019914	0,383	232902_s_at	Arginyl-tRNA synthetase 2, mitochondrial (putative)	*RARS2*
3263	52,0	39,6	1,31	0,023162	0,388	236160_at	Thyroid hormone receptor interactor 11	*TRIP11*
2225	77,6	59,1	1,31	0,014790	0,363	218092_s_at	HIV-1 Rev binding protein	*HRB*
4676	73,8	56,2	1,31	0,035307	0,413	205084_at	B-cell receptor-associated protein 29	*BCAP29*
780	10,0	7,63	1,31	0,004474	0,311	232599_at	Exocyst complex component 6	*EXOC6*
4588	63,9	48,8	1,31	0,034690	0,412	230619_at	Aryl hydrocarbon receptor nuclear translocator	*ARNT*
2705	15,3	11,6	1,31	0,018551	0,375	210290_at	Zinc finger protein 174	*ZNF174*
1858	32,8	25,0	1,31	0,011974	0,352	214057_at	Myeloid cell leukemia sequence 1 (BCL2-related)	*MCL1*
2041	151	115	1,31	0,013276	0,355	208772_at	Ankyrin repeat and KH domain containing 1	*ANKHD1*
5921	8,31	6,34	1,31	0,047993	0,443	228577_x_at	Outer dense fiber of sperm tails 2-like	*ODF2L*

Index	Mutated*	Normal*	FC	*P* value	FDR**	Probe set	Description	Gene symbol
3463	50,1	38,3	1,31	0,024811	0,392	204167_at	Biotinidase	*BTD*
794	57,5	43,9	1,31	0,004535	0,311	205340_at	Zinc finger and BTB domain containing 24	*ZBTB24*
3534	19,5	14,9	1,31	0,025404	0,393	236094_at	Transcription factor 7-like 2 (T-cell specific, HMG-box)	*TCF7L2*
3199	26,7	20,4	1,31	0,022658	0,387	228184_at	Dispatched homolog 1 (Drosophila)	*DISP1*
4811	11,9	9,05	1,31	0,036542	0,415	222242_s_at	Kallikrein-related peptidase 5	*KLK5*
4173	75,7	57,8	1,31	0,030852	0,404	202440_s_at	Suppression of tumorigenicity 5	*ST5*
2829	67,6	51,6	1,31	0,019735	0,381	226981_at	Myeloid/lymphoid or mixed-lineage leukemia	*MLL*
3278	86,0	65,7	1,31	0,023351	0,388	218713_at	NMDA receptor regulated 2	*NARG2*
984	169	129	1,31	0,005692	0,315	203156_at	A kinase (PRKA) anchor protein 11	*AKAP11*
4681	27,3	20,9	1,31	0,035344	0,413	238028_at	Chromosome 6 open reading frame 132	*C6orf132*
5093	223	171	1,31	0,039187	0,420	202373_s_at	RAB3 GTPase activating protein subunit 2 (non-catalytic)	*RAB3GAP2*
4180	230	176	1,31	0,030902	0,404	212418_at	E74-like factor 1 (ets domain transcription factor)	*ELF1*
2049	221	169	1,31	0,013309	0,355	214594_x_at	ATPase, Class I, type 8B, member 1	*ATP8B1*
5159	51,8	39,6	1,31	0,039732	0,421	217653_x_at	-	-
3750	155	119	1,31	0,027267	0,397	218323_at	RAS homolog gene family, member T1	*RHOT1*
605	36,1	27,6	1,31	0,003356	0,303	235240_at	Ataxin 3	*ATXN3*
3666	1006	770	1,31	0,026498	0,395	223000_s_at	F11 receptor	*F11R*
2383	9,37	7,16	1,31	0,015893	0,364	244286_at	Formin binding protein 1	*FNBP1*
3106	262	201	1,31	0,021931	0,386	203137_at	Wilms tumor 1 associated protein	*WTAP*
4662	28,7	22,0	1,31	0,035198	0,413	243492_at	Thioesterase superfamily member 4	*THEM4*
5207	37,1	28,4	1,31	0,040181	0,422	1552656_s_a	U2AF homology motif (UHM) kinase 1	*UHMK1*
4844	87,7	67,1	1,31	0,036919	0,417	203883_s_at	RAB11 family interacting protein 2 (class I)	*RAB11FIP2*
4564	96,7	74,0	1,31	0,034503	0,412	202165_at	Protein phosphatase 1, regulatory (inhibitor) subunit 2	*PPP1R2*
223	16,1	12,3	1,31	0,001029	0,250	238841_at	Protein tyrosine phosphatase domain containing 1	*PTPDC1*
4893	372	285	1,31	0,037386	0,418	1554241_at	Coagulation factor C homolog, cochlin	*COCH*
912	78,1	59,8	1,31	0,005252	0,313	203187_at	Dedicator of cytokinesis 1	*DOCK1*
3399	60,0	45,9	1,31	0,024249	0,390	203375_s_at	Tripeptidyl peptidase II	*TPP2*
4012	71,5	54,7	1,31	0,029386	0,400	215698_at	Jumonji, AT rich interactive domain 1A	*JARID1A*
1399	290	222	1,31	0,008450	0,330	200931_s_at	Vinculin	*VCL*
5854	23,6	18,1	1,31	0,047338	0,442	222736_s_at	Transmembrane protein 38B	*TMEM38B*
3942	20,0	15,4	1,31	0,028788	0,399	222134_at	D-aspartate oxidase	*DDO*
4973	9,85	7,54	1,31	0,038156	0,419	203699_s_at	Deiodinase, iodothyronine, type II	*DIO2*
1676	83,0	63,6	1,31	0,010574	0,345	215136_s_at	Exosome component 8	*EXOSC8*
5658	60,8	46,6	1,31	0,045133	0,436	218503_at	KIAA1797 protein	*KIAA1797*
5842	113	86,5	1,31	0,047213	0,442	215450_at	Small nuclear ribonucleoprotein polypeptide E	*SNRPE*
6038	45,6	34,9	1,31	0,049156	0,445	219687_at	Hedgehog acyltransferase	*HHAT*
2762	76,0	58,2	1,30	0,019163	0,379	220925_at	N(alpha)-acetyltransferase 35, NatC auxiliary subunit	*MAK10*
1081	179	137	1,30	0,006326	0,319	223028_s_at	Sorting nexin 9	*SNX9*
5977	46,9	35,9	1,30	0,048541	0,444	232046_at	KIAA1217 protein	*KIAA1217*
1250	911	699	1,30	0,007424	0,325	224569_s_at	Interferon regulatory factor 2 binding protein 2	*IRF2BP2*
5137	6,22	4,77	1,30	0,039544	0,421	241885_at	68 kDa TATA-binding protein-associated factor	*TAF15*
3741	45,3	34,7	1,30	0,027224	0,397	214221_at	Alstrom syndrome 1	*ALMS1*
2673	37,0	28,4	1,30	0,018275	0,374	1558996_at	Forkhead box P1	*FOXP1*
3890	76,6	58,8	1,30	0,028433	0,399	213788_s_at	Non-protein coding RNA 94	*FLJ35348*
2907	80,3	61,6	1,30	0,020432	0,383	203971_at	Solute carrier family 31 (copper transporters), member 1	*SLC31A1*
171	106	81,4	1,30	0,000791	0,244	227026_at	M-phase phosphoprotein, mpp8	*HSMPP8*
3214	11,4	8,76	1,30	0,022771	0,387	244669_at	Small nucleolar RNA, C/D box 50A	*SNHG5*
1321	8,87	6,81	1,30	0,007877	0,326	238859_at	Family with sequence similarity 149, member B1	*FAM149B1*
1407	180	138	1,30	0,008500	0,330	219083_at	SHQ1 homolog (S. cerevisiae)	*SHQ1*
4929	44,4	34,1	1,30	0,037797	0,419	227891_s_at	68 kDa TATA-binding protein-associated factor	*TAF15*
1396	151	116	1,30	0,008434	0,330	203132_at	Retinoblastoma 1 (including osteosarcoma)	*RB1*
4807	38,5	29,6	1,30	0,036495	0,415	212366_at	Zinc finger protein 292	*ZNF292*
3640	168	129	1,30	0,026326	0,395	223189_x_at	Myeloid/lymphoid or mixed-lineage leukemia 5	*MLL5*
3711	266	205	1,30	0,026866	0,396	208815_x_at	Heat shock 70kDa protein 4	*HSPA4*
1118	292	224	1,30	0,006533	0,319	202181_at	KIAA0247 protein	*KIAA0247*
1958	186	143	1,30	0,012798	0,355	202515_at	Discs, large homolog 1 (Drosophila)	*DLG1*
1969	32,6	25,0	1,30	0,012861	0,355	201905_s_at	CTD small phosphatase-like protein	*CTDSPL*

Index	Mutated*	Normal*	FC	*P* value	FDR**	Probe set	Description	Gene symbol
2555	94,7	72,8	1,30	0,017292	0,370	212923_s_at	Chromosome 6 open reading frame 145	*C6orf145*
5628	240	185	1,30	0,044933	0,436	208999_at	Septin 8	*SEPT8*
1483	23,4	18,0	1,30	0,009115	0,336	243350_at	Chromosome 5 open reading frame 15	*C5orf15*
2226	21,6	16,6	1,30	0,014798	0,363	231995_at	Chromosome 9 open reading frame 82	*C9orf82*
2496	488	375	1,30	0,016821	0,367	229394_s_at	Glucocorticoid receptor DNA binding factor 1	*GRLF1*
5666	114	87,7	1,30	0,045208	0,436	220419_s_at	Ubiquitin specific peptidase 25	*USP25*
4315	4,43	3,41	1,30	0,032137	0,407	242906_at	cDNA DKFZp313B1017	-
2484	204	157	1,30	0,016747	0,367	204314_s_at	cAMP responsive element binding protein 1	*CREB1*
1222	12,1	9,33	1,30	0,007269	0,324	201335_s_at	Rho guanine nucleotide exchange factor (GEF) 12	*ARHGEF12*
4492	111	85,7	1,30	0,033777	0,411	201883_s_at	UDP-Gal:betaGlcNAc beta 1,4- galactosyltransferase, polypeptide 1	*B4GALT1*
2376	74,8	57,6	1,30	0,015843	0,364	205606_at	Low density lipoprotein receptor-related protein 6	*LRP6*
1089	26,2	20,2	1,30	0,006385	0,319	212759_s_at	Transcription factor 7-like 2 (T-cell specific, HMG-box)	*TCF7L2*
2488	10,3	7,93	1,30	0,016766	0,367	225807_at	Jub, ajuba homolog (Xenopus laevis)	*JUB*
91	134	103	1,30	0,000335	0,201	220355_s_at	Polybromo 1	*PBRM1*
4153	46,5	35,8	1,30	0,030632	0,403	224631_at	Zinc finger protein 91 homolog (mouse)	*ZFP91*
2045	15,9	12,3	1,30	0,013297	0,355	231933_at	Membrane-associated ring finger (C3HC4) 8	*MARCH8*
3284	206	159	1,30	0,023406	0,388	223190_s_at	Myeloid/lymphoid or mixed-lineage leukemia 5	*MLL5*
3796	209	161	1,30	0,027652	0,398	212888_at	Dicer1, Dcr-1 homolog (Drosophila)	*DICER1*
4995	98,1	75,5	1,30	0,038339	0,420	225954_s_at	Midnolin	*MIDN*
2218	9,64	7,43	1,30	0,014709	0,362	217951_s_at	PHD finger protein 3	*PHF3*
2105	11,5	8,89	1,30	0,013791	0,358	239930_at	UDP-GalNAc:polypeptide N-acetylgalactosaminyltransferase 2	*GALNT2*
4101	253	195	1,30	0,030146	0,402	202271_at	F-box protein 28	*FBXO28*
603	22,8	17,6	1,30	0,003349	0,303	205135_s_at	nuclear fragile X mental retardation protein interacting protein 1	*NUFIP1*
5735	101	78,1	1,30	0,046000	0,438	234491_s_at	Salvador homolog 1 (Drosophila)	*SAV1*
6113	4633	3570	1,30	0,049881	0,446	215076_s_at	Collagen, type III, alpha 1	*COL3A1*
5249	287	221	1,30	0,040640	0,423	228785_at	Zinc finger protein 281	*ZNF281*
5708	140	108	1,30	0,045701	0,438	205178_s_at	Retinoblastoma binding protein 6	*RBBP6*
5775	78,8	60,8	1,30	0,046449	0,440	204066_s_at	Centaurin, gamma 2	*CENTG2*
3555	755	582	1,30	0,025641	0,394	200983_x_at	CD59 molecule, complement regulatory protein	*CD59*
5524	13,0	10,0	1,30	0,043811	0,433	230126_s_at	Jumonji domain containing 2B	*JMJD2B*
3999	123	95,0	1,30	0,029269	0,400	228980_at	Ring finger and FYVE-like domain containing 1	*RFFL*
1420	58,5	45,2	1,30	0,008606	0,331	235728_at	Zinc finger protein 3 homolog (mouse)	*ZFP3*
2468	46,1	35,6	1,30	0,016587	0,367	239035_at	5,10-methylenetetrahydrofolate reductase (NADPH)	*MTHFR*
1229	294	227	1,29	0,007311	0,324	201302_at	Annexin A4	*ANXA4*
4333	187	144	1,29	0,032255	0,407	214177_s_at	Pre-B-cell leukemia homeobox interacting protein 1	*PBXIP1*
2280	240	186	1,29	0,015155	0,363	225629_s_at	Zinc finger and BTB domain containing 4	*ZBTB4*
2530	185	143	1,29	0,017088	0,369	202372_at	RAB3 GTPase activating protein subunit 2 (non-catalytic)	*RAB3GAP2*
5392	195	151	1,29	0,042279	0,429	225334_at	Chromosome 10 open reading frame 32	*C10orf32*
2135	5,02	3,88	1,29	0,014029	0,359	1564699_at	cDNA clone IMAGE:4297077	-
2589	80,1	61,9	1,29	0,017551	0,371	202414_at	DNA excision repair protein ERCC-5	*ERCC5*
2257	24,7	19,1	1,29	0,015020	0,363	229298_at	Kelch repeat and BTB (POZ) domain containing 7	*KBTBD7*
1390	195	150	1,29	0,008404	0,330	210249_s_at	Nuclear receptor coactivator 1	*NCOA1*
2761	20,1	15,5	1,29	0,019156	0,379	230937_at	Hypothetical protein LOC285835	*LOC285835*
982	307	237	1,29	0,005690	0,315	211503_s_at	RAB14, member RAS oncogene family	*RAB14*
1713	4,34	3,36	1,29	0,010984	0,348	221805_at	neurofilament, light polypeptide 68kDa	*NEFL*
4539	170	132	1,29	0,034291	0,412	212648_at	DEAH (Asp-Glu-Ala-His) box polypeptide 29	*DHX29*
4157	108	83,4	1,29	0,030721	0,404	218802_at	Coiled-coil domain containing 109B	*CCDC109B*
4212	298	231	1,29	0,031187	0,405	213233_s_at	Kelch-like 9 (Drosophila)	*KLHL9*
5340	334	259	1,29	0,041822	0,428	201015_s_at	Junction plakoglobin	*JUP*
4249	281	218	1,29	0,031552	0,406	202593_s_at	Membrane interacting protein of RGS16	*MIR16*
5621	61,7	47,8	1,29	0,044867	0,436	218644_at	Pleckstrin 2	*PLEK2*
2345	28,6	22,2	1,29	0,015647	0,364	207492_at	N-glycanase 1	*NGLY1*
1967	1471	1140	1,29	0,012857	0,355	226880_at	Nuclear casein kinase and cyclin-dependent kinase substrate 1	*NUCKS1*
1630	6,92	5,36	1,29	0,010280	0,344	235133_at	Zinc finger, DHHC domain containing 14	*ZDHHC14*
1179	13,7	10,6	1,29	0,006930	0,321	244536_at	Tumor protein p53 binding protein, 2	*TP53BP2*
5096	57,9	44,9	1,29	0,039207	0,420	210731_s_at	Lectin, galactoside-binding, soluble, 8 (galectin 8)	*LGALS8*
1068	333	258	1,29	0,006266	0,319	203033_x_at	Fumarate hydratase	*FH*

Index	Mutated*	Normal*	FC	*P* value	FDR**	Probe set	Description	Gene symbol
1436	6,50	5,04	1,29	0,008701	0,331	222936_s_at	PPPDE peptidase domain containing 1	*PPPDE1*
4562	5,57	4,32	1,29	0,034472	0,412	1559360_at	Ephrin-A5	*EFNA5*
3460	8,20	6,36	1,29	0,024786	0,392	1559343_at	Small nuclear ribonucleoprotein polypeptide N	*SNRPN*
2575	273	212	1,29	0,017479	0,371	203045_at	Ninjurin 1	*NINJ1*
385	245	190	1,29	0,002005	0,284	223238_s_at	Polybromo 1	*PBRM1*
4238	23,7	18,4	1,29	0,031402	0,405	1555082_a_a	NIMA (never in mitosis gene a)- related kinase 11	*NEK11*
1599	22,5	17,5	1,29	0,009976	0,341	224375_at	-	-
1385	15,4	12,0	1,29	0,008329	0,329	1558755_x_a	Zinc finger protein 763	*ZNF763*
2978	48,7	37,8	1,29	0,020942	0,384	221985_at	Kelch-like 24 (Drosophila)	*KLHL24*
4865	11,1	8,63	1,29	0,037107	0,417	1556423_at	Vasohibin 1	*VASH1*
5215	164	127	1,29	0,040292	0,422	225399_at	Chromosome 1 open reading frame 19	*C1orf19*
2283	443	344	1,29	0,015178	0,363	201816_s_at	Glioblastoma amplified sequence	*GBAS*
1219	23,7	18,4	1,29	0,007250	0,324	241874_at	Chromosome 5 open reading frame 53	*C5orf53*
6086	285	222	1,29	0,049690	0,446	206989_s_at	Splicing factor, arginine/serine-rich 2, interacting protein	*SFRS2IP*
5857	90,8	70,7	1,29	0,047366	0,442	58780_s_at	Hypothetical protein FLJ10357	*FLJ10357*
1082	175	136	1,29	0,006332	0,319	212104_s_at	RNA binding motif protein 9	*RBM9*
5890	25,8	20,1	1,29	0,047638	0,442	236109_at	RNA pseudouridylate synthase domain containing 4	*RPUSD4*
3537	94,5	73,6	1,29	0,025444	0,393	210028_s_at	Origin recognition complex, subunit 3-like (yeast)	*ORC3L*
2918	43,8	34,1	1,28	0,020483	0,383	234660_s_at	DIS3 mitotic control homolog (S. cerevisiae)	*DIS3*
3039	105	81,9	1,28	0,021421	0,385	218298_s_at	Chromosome 14 open reading frame 159	*C14orf159*
492	73,8	57,5	1,28	0,002634	0,289	230623_x_at	Ubiquitin specific peptidase 28	*USP28*
4337	36,7	28,6	1,28	0,032273	0,407	219307_at	Prenyl (decaprenyl) diphosphate synthase, subunit 2	*PDSS2*
2969	626	488	1,28	0,020864	0,384	212773_s_at	Translocase of outer mitochondrial membrane 20 homolog (yeast)	*TOMM20*
1667	132	103	1,28	0,010536	0,345	212676_at	Neurofibromin 1	*NF1*
4377	80,7	62,9	1,28	0,032561	0,407	223679_at	Catenin (cadherin-associated protein), beta 1, 88kDa	*CTNNB1*
2813	123	95,6	1,28	0,019620	0,381	202005_at	Suppression of tumorigenicity 14 (colon carcinoma)	*ST14*
29	4,61	3,60	1,28	0,000134	0,201	234330_at	cDNA FLJ14081 fis, clone HEMBB1002280	-
4967	17,2	13,4	1,28	0,038108	0,419	210251_s_at	RUN and FYVE domain containing 3	*RUFY3*
2127	169	132	1,28	0,013995	0,359	201411_s_at	Pleckstrin homology domain-containing family B member 2	*PLEKHB2*
4029	52,3	40,8	1,28	0,029509	0,400	213271_s_at	Dopey family member 1	*DOPEY1*
5237	62,9	49,0	1,28	0,040542	0,423	204831_at	Cyclin-dependent kinase 8	*CDK8*
2335	27,4	21,4	1,28	0,015586	0,364	213298_at	Nuclear factor I/C (CCAAT-binding transcription factor)	*NFIC*
4434	89,9	70,2	1,28	0,033210	0,409	241993_x_at	Forkhead box P1	*FOXP1*
4908	395	308	1,28	0,037540	0,418	226128_at	Chromosome 1 open reading frame 58	*C1orf58*
3568	18,7	14,6	1,28	0,025750	0,394	228748_at	CD59 molecule, complement regulatory protein	*CD59*
3586	87,3	68,2	1,28	0,025882	0,394	1553677_a_a	TIP41, TOR signaling pathway regulator-like (S. cerevisiae)	*TIPRL*
2621	2180	1702	1,28	0,017810	0,371	217807_s_at	Glioma tumor suppressor candidate region gene 2	*GLTSCR2*
4536	172	134	1,28	0,034269	0,412	217954_s_at	PHD finger protein 3	*PHF3*
5798	53,9	42,1	1,28	0,046770	0,441	242750_at	Methylmalonic aciduria (cobalamin deficiency) cblA type	*MMAA*
3493	10,0	7,85	1,28	0,025104	0,392	224044_at	RAS homolog gene family, member T1	*RHOT1*
5800	14,2	11,1	1,28	0,046781	0,441	206448_at	Zinc finger protein 365	*ZNF365*
4907	5,77	4,51	1,28	0,037524	0,418	1557961_s_a	N-terminal EF-hand calcium binding protein 1	*NECAB1*
1811	10,5	8,23	1,28	0,011679	0,351	244272_s_at	Membrane targeting (tandem) C2 domain containing 1	*MTAC2D1*
1064	62,4	48,8	1,28	0,006244	0,319	227895_at	Family with sequence similarity 120B	*FAM120B*
4008	93,5	73,1	1,28	0,029322	0,400	223021_x_at	Vps20-associated 1 homolog (S. cerevisiae)	*VTA1*
5687	34,5	27,0	1,28	0,045389	0,436	239364_at	Ets variant 6	*ETV6*
5847	12,8	9,97	1,28	0,047281	0,442	228948_at	EPH receptor A4	*EPHA4*
3872	210	164	1,28	0,028285	0,399	223151_at	DCUN1 domain-containing protein 5	*DCUN1D5*
486	205	160	1,28	0,002589	0,289	223329_x_at	SGT1, suppressor of G2 allele of SKP1 (S. cerevisiae)	*SUGT1*
1743	13,1	10,2	1,28	0,011131	0,348	1569020_at	Neural precursor cell expressed, developmentally down-regulated 9	*NEDD9*
2449	110	85,9	1,28	0,016399	0,366	226277_at	Collagen, type IV, alpha 3 (Goodpasture antigen) binding protein	*COL4A3BP*
3232	90,8	71,0	1,28	0,022914	0,388	225838_at	Enhancer of polycomb homolog 2 (Drosophila)	*EPC2*
938	42,2	33,0	1,28	0,005368	0,313	204786_s_at	Interferon (alpha, beta and omega) receptor 2	*IFNAR2*
5498	98,2	76,8	1,28	0,043512	0,433	235196_at	Hyperparathyroidism 2 protein	*CDC73*
1329	266	208	1,28	0,007999	0,328	217885_at	Importin 9	*IPO9*
1370	10,8	8,42	1,28	0,008234	0,328	205460_at	neuronal PAS domain protein 2	*NPAS2*
5016	5,70	4,46	1,28	0,038592	0,420	206032_at	Desmocollin 3	*DSC3*

Index	Mutated*	Normal*	FC	*P* value	FDR**	Probe set	Description	Gene symbol
1006	24,5	19,2	1,28	0,005833	0,316	217230_at	Villin 2 (ezrin)	*VIL2*
5307	6,06	4,74	1,28	0,041399	0,426	1557895_at	FLJ35934 protein	*FLJ35934*
577	7,63	5,98	1,28	0,003171	0,300	217538_at	Small G protein signaling modulator 2	*SGSM2*
3138	105	82,6	1,28	0,022259	0,387	210776_x_at	Transcription factor 3	*TCF3*
6105	106	83,2	1,28	0,049843	0,446	223304_at	Solute carrier family 37 member 3	*SLC37A3*
5891	27,4	21,4	1,28	0,047647	0,442	230759_at	Sorting nexin 14	*SNX14*
2267	48,1	37,7	1,28	0,015072	0,363	240166_x_at	RNA (guanine-9-) methyltransferase domain containing 3	*RG9MTD3*
5963	7,83	6,13	1,28	0,048431	0,444	241198_s_at	Chromosome 11 open reading frame 70	*C11orf70*
3865	60,8	47,7	1,28	0,028230	0,399	225539_at	Zinc finger protein 295	*ZNF295*
3527	74,1	58,0	1,28	0,025327	0,393	209798_at	Nuclear protein, ataxia-telangiectasia locus	*NPAT*
2689	123	96,5	1,28	0,018430	0,375	238761_at	Mediator complex subunit 28	*MED28*
2565	11,8	9,27	1,28	0,017400	0,371	236149_at	THO complex 3	*THOC3*
4541	60,7	47,6	1,28	0,034311	0,412	219303_at	Chromosome 13 open reading frame 7	*C13orf7*
97	5,86	4,60	1,28	0,000377	0,212	1560776_at	Family with sequence similarity 18, member B2	*FAM18B2*
681	153	120	1,28	0,003912	0,308	224309_s_at	SGT1, suppressor of G2 allele of SKP1 (S. cerevisiae)	*SUGT1*
5349	359	282	1,28	0,041933	0,429	202322_s_at	Geranylgeranyl diphosphate synthase 1	*GGPS1*
747	5,76	4,52	1,28	0,004234	0,308	37170_at	BMP2 inducible kinase	*BMP2K*
4602	24,9	19,5	1,28	0,034797	0,412	236268_at	SEC22 vesicle trafficking protein homolog C (S. cerevisiae)	*SEC22C*
5570	6,29	4,93	1,28	0,044354	0,435	232885_at	Non-protein coding RNA 81	*NCRNA00081*
3163	59,1	46,4	1,28	0,022450	0,387	222607_s_at	DIS3 mitotic control homolog (S. cerevisiae)	*DIS3*
5397	101	79,3	1,28	0,042322	0,429	210285_x_at	Wilms tumor 1 associated protein	*WTAP*
5559	30,4	23,9	1,27	0,044255	0,435	219011_at	Pleckstrin homology domain-containing family A member 4	*PLEKHA4*
3471	140	110	1,27	0,024902	0,392	231807_at	KIAA1217 protein	*KIAA1217*
1166	18,7	14,6	1,27	0,006860	0,321	242136_x_at	Hypothetical protein LOC403340	*MGC70870*
1342	7,76	6,09	1,27	0,008057	0,328	240465_at	Chromosome 4 open reading frame 32	*C4orf32*
820	178	140	1,27	0,004683	0,312	207643_s_at	Tumor necrosis factor receptor superfamily, member 1A	*TNFRSF1A*
1519	67,7	53,1	1,27	0,009389	0,337	213463_s_at	Family with sequence similarity 149, member B1	*FAM149B1*
3664	342	269	1,27	0,026486	0,395	202677_at	RAS p21 protein activator (GTPase activating protein) 1	*RASA1*
5076	13,7	10,8	1,27	0,039052	0,420	232279_at	PHD finger protein 15	*PHF15*
2154	62,3	48,9	1,27	0,014186	0,360	212076_at	Myeloid/lymphoid or mixed-lineage leukemia	*MLL*
2549	139	110	1,27	0,017262	0,370	227569_at	Ligand of numb-protein X 2	*LNX2*
4470	220	173	1,27	0,033588	0,411	202790_at	Claudin 7	*CLDN7*
4216	4,20	3,30	1,27	0,031239	0,405	235075_at	Desmoglein 3 (pemphigus vulgaris antigen)	*DSG3*
6044	78,0	61,3	1,27	0,049239	0,445	225504_at	Homeobox containing 1	*HMBOX1*
2630	38,4	30,2	1,27	0,017905	0,372	204838_s_at	MUTL homolog 3 (E. coli)	*MLH3*
3657	60,0	47,2	1,27	0,026413	0,395	209378_s_at	KIAA1128 protein	*KIAA1128*
1745	12,0	9,44	1,27	0,011145	0,348	232457_at	LIM and calponin homology domains 1	*LIMCH1*
5850	47,5	37,3	1,27	0,047331	0,442	226783_at	Alanine-glyoxylate aminotransferase 2-like 2	*AGXT2L2*
2785	10,0	7,89	1,27	0,019343	0,380	237262_at	Family with sequence similarity 59, member A	*FAM59A*
4049	26,4	20,7	1,27	0,029667	0,401	204054_at	Phosphatase and tensin homolog	*PTEN*
4278	91,3	71,8	1,27	0,031792	0,406	222136_x_at	Zinc finger protein 43	*ZNF43*
5169	23,7	18,7	1,27	0,039825	0,421	222364_at	Solute carrier family 44, member 1	*SLC44A1*
4042	30,0	23,6	1,27	0,029635	0,401	228091_at	Syntaxin 17	*STX17*
3593	632	497	1,27	0,025913	0,394	201334_s_at	Rho guanine nucleotide exchange factor (GEF) 12	*ARHGEF12*
3047	176	138	1,27	0,021453	0,385	224703_at	WD repeat domain 22	*WDR22*
4156	170	134	1,27	0,030678	0,404	225080_at	Myosin IC	*MYO1C*
4575	18,8	14,8	1,27	0,034585	0,412	205180_s_at	ADAM metallopeptidase domain 8	*ADAM8*
3906	116	91,0	1,27	0,028522	0,399	218244_at	Nucleolar protein 8	*NOL8*
3854	6,52	5,13	1,27	0,028142	0,399	1556650_at	Autism susceptibility candidate 2	*AUTS2*
6065	77,8	61,3	1,27	0,049473	0,446	240277_at	Solute carrier family 30 (zinc transporter), member 7	*SLC30A7*
814	10,4	8,23	1,27	0,004655	0,312	222438_at	Mediator complex subunit 4	*MED4*
4399	12,1	9,55	1,27	0,032786	0,407	238342_at	Hypothetical protein LOC283846	*LOC283846*
1183	4,53	3,57	1,27	0,006957	0,322	1557036_at	Zinc finger and BTB domain containing 1	*ZBTB1*
3006	11,1	8,77	1,27	0,021167	0,385	230542_at	Zinc finger protein 597	*ZNF597*
3925	11,3	8,87	1,27	0,028693	0,399	206652_at	Zinc finger, MYM-type 5	*ZMYM5*
6055	144	113	1,27	0,049379	0,446	213026_at	ATG12 autophagy related 12 homolog (S. cerevisiae)	*ATG12*
2800	67,8	53,4	1,27	0,019466	0,380	219024_at	Pleckstrin homology domain containing, family A member 1	*PLEKHA1*

Index	Mutated*	Normal*	FC	*P* value	FDR**	Probe set	Description	Gene symbol
4196	20,6	16,3	1,27	0,030996	0,404	240723_at	Enah/Vasp-like	*EVL*
3499	136	107	1,27	0,025144	0,392	214773_x_at	TIP41, TOR signaling pathway regulator-like (S. cerevisiae)	*TIPRL*
5810	374	295	1,27	0,046927	0,442	226214_at	Membrane interacting protein of RGS16	*MIR16*
1635	6,21	4,90	1,27	0,010310	0,344	240829_at	Peroxisomal biogenesis factor 3	*PEX3*
4342	30,1	23,8	1,27	0,032332	0,407	206348_s_at	Pyruvate dehydrogenase kinase, isozyme 3	*PDK3*
2342	78,3	61,8	1,27	0,015631	0,364	225398_at	RNA pseudouridylate synthase domain containing 4	*RPUSD4*
1283	161	127	1,27	0,007640	0,325	218243_at	RUN and FYVE domain containing 1	*RUFY1*
1924	129	102	1,27	0,012518	0,355	224945_at	BTB (POZ) domain containing 7	*BTBD7*
3439	143	113	1,27	0,024624	0,391	222414_at	Myeloid/lymphoid or mixed-lineage leukemia 3	*MLL3*
5755	23,3	18,4	1,27	0,046191	0,439	230164_at	Zinc finger protein 621	*ZNF621*
4724	78,3	61,8	1,27	0,035738	0,414	215616_s_at	Jumonji domain containing 2B	*JMJD2B*
3536	18,9	14,9	1,27	0,025437	0,393	216621_at	Rho-associated, coiled-coil containing protein kinase 1	*ROCK1*
4735	69,8	55,1	1,27	0,035844	0,414	203481_at	Chromosome 10 open reading frame 6	*C10orf6*
3314	7,15	5,65	1,27	0,023558	0,388	243555_at	Endothelin receptor type A	*EDNRA*
1291	12,7	10,1	1,27	0,007697	0,325	211094_s_at	Neurofibromin 1	*NF1*
4313	144	114	1,27	0,032102	0,407	209024_s_at	Synaptotagmin binding, cytoplasmic RNA interacting protein	*SYNCRIP*
3782	6,86	5,42	1,26	0,027532	0,398	1559697_a_a	cDNA clone YW24B11	-
3072	148	117	1,26	0,021670	0,385	234950_s_at	Ring finger and WD repeat domain 2	*RFWD2*
2142	170	135	1,26	0,014101	0,360	202386_s_at	KIAA0430 protein	*KIAA0430*
3765	35,0	27,7	1,26	0,027366	0,397	213100_at	Unc-5 homolog B (C. elegans)	*UNC5B*
3948	77,1	60,9	1,26	0,028828	0,399	217496_s_at	Insulin-degrading enzyme	*IDE*
5317	79,5	62,9	1,26	0,041490	0,427	204084_s_at	Ceroid-lipofuscinosis, neuronal 5	*CLN5*
5368	39,4	31,2	1,26	0,042143	0,429	219565_at	Cytochrome P450, family 20, subfamily A, polypeptide 1	*CYP20A1*
454	352	278	1,26	0,002430	0,288	222389_s_at	WW domain containing adaptor with coiled-coil	*WAC*
1018	74,9	59,3	1,26	0,005886	0,316	63009_at	SHQ1 homolog (S. cerevisiae)	*SHQ1*
3631	45,3	35,9	1,26	0,026255	0,395	214220_s_at	Alstrom syndrome 1	*ALMS1*
4269	44,1	34,9	1,26	0,031745	0,406	1556034_s_a	Myotubularin related protein 11	*MTMR11*
1027	301	238	1,26	0,005943	0,316	217836_s_at	YY1 associated protein 1	*YY1AP1*
2901	152	121	1,26	0,020371	0,383	201613_s_at	Adaptor-related protein complex 1, gamma 2 subunit	*AP1G2*
4898	22,3	17,7	1,26	0,037472	0,418	204742_s_at	PDS5, regulator of cohesion maintenance, homolog B (S. cerevisiae)	*PDS5B*
4831	35,8	28,4	1,26	0,036802	0,416	219610_at	Rho-guanine nucleotide exchange factor	*RGNEF*
3881	323	256	1,26	0,028341	0,399	209102_s_at	HMG-box transcription factor 1	*HBP1*
5677	82,9	65,7	1,26	0,045324	0,436	203972_s_at	Peroxisomal biogenesis factor 3	*PEX3*
3958	104	82,4	1,26	0,028901	0,399	203035_s_at	Protein inhibitor of activated STAT, 3	*PIAS3*
472	11,8	9,34	1,26	0,002513	0,289	241152_at	Family with sequence similarity 120B	*FAM120B*
6017	11,7	9,29	1,26	0,048969	0,445	213067_at	Myosin, heavy chain 10, non-muscle	*MYH10*
3964	308	244	1,26	0,028956	0,399	201132_at	Heterogeneous nuclear ribonucleoprotein H2	*HNRPH2*
2462	354	281	1,26	0,016522	0,367	201301_s_at	Annexin A4	*ANXA4*
3089	329	261	1,26	0,021783	0,385	201153_s_at	Muscleblind-like (Drosophila)	*MBNL1*
4614	286	227	1,26	0,034830	0,412	224570_s_at	Interferon regulatory factor 2 binding protein 2	*IRF2BP2*
2072	285	227	1,26	0,013542	0,356	234969_s_at	Enhancer of polycomb homolog 1 (Drosophila)	*EPC1*
3853	9,22	7,32	1,26	0,028139	0,399	235784_at	Mitochondrial transcription termination factor	*MTERF*
2482	7,45	5,91	1,26	0,016728	0,367	206010_at	Hyaluronan binding protein 2	*HABP2*
4742	226	179	1,26	0,035914	0,414	225284_at	DnaJ (Hsp40) homolog, subfamily C, member 3	*DNAJC3*
4449	56,4	44,8	1,26	0,033417	0,411	200702_s_at	DEAD (Asp-Glu-Ala-Asp) box polypeptide 24	*DDX24*
4491	559	445	1,26	0,033777	0,411	212952_at	Calreticulin	*CALR*
5063	6,69	5,32	1,26	0,038964	0,420	242007_at	Primary neuroblastoma cDNA, clone:Nbla03526, full insert sequence	-
3834	833	662	1,26	0,028012	0,399	200810_s_at	Cold inducible RNA binding protein	*CIRBP*
5563	14,7	11,7	1,26	0,044296	0,435	226637_at	Ubiquitin-conjugating enzyme E2H (UBC8 homolog, yeast)	*UBE2H*
1821	7,77	6,18	1,26	0,011725	0,351	219834_at	Calcium-response factor	*ALS2CR8*
3897	25,0	19,9	1,26	0,028465	0,399	238722_x_at	N-acyl-phosphatidylethanolamine-hydrolyzing phospholipase D	*NAPE-PLD*
1618	128	102	1,26	0,010167	0,343	203530_s_at	Syntaxin 4	*STX4*
4158	808	643	1,26	0,030724	0,404	201121_s_at	Progesterone receptor membrane component 1	*PGRMC1*
4707	137	109	1,26	0,035583	0,413	201529_s_at	Replication protein A1, 70kDa	*RPA1*
2443	157	125	1,26	0,016345	0,366	224834_at	Ubiquitin domain containing 2	*UBTD2*
2177	5,74	4,57	1,26	0,014405	0,361	242985_x_at	Ring finger protein 180	*RNF180*
1831	12,2	9,71	1,26	0,011795	0,351	1565620_at	Centaurin, gamma-like family, member 1	*CTGLF1*

Index	Mutated*	Normal*	FC	*P* value	FDR**	Probe set	Description	Gene symbol
5478	9,00	7,17	1,26	0,043318	0,432	229669_at	Hypothetical gene supported by BC072410	*LOC440416*
3255	152	121	1,26	0,023112	0,388	227864_s_at	Family with sequence similarity 125, member A	*FAM125A*
5185	71,5	57,0	1,25	0,039966	0,421	212840_at	UBX domain containing 7	*UBXD7*
1262	8,22	6,55	1,25	0,007513	0,325	232722_at	Ribonuclease T2	*RNASET2*
5693	39,0	31,1	1,25	0,045552	0,437	238783_at	Transmembrane protein 161B	*TMEM161B*
5157	365	291	1,25	0,039709	0,421	201887_at	Interleukin 13 receptor, alpha 1	*IL13RA1*
5105	15,4	12,3	1,25	0,039278	0,421	223400_s_at	Polybromo 1	*PBRM1*
5138	285	227	1,25	0,039549	0,421	223306_at	Emopamil binding protein-like	*EBPL*
2239	176	140	1,25	0,014886	0,363	218142_s_at	Cereblon	*CRBN*
3167	8,58	6,85	1,25	0,022473	0,387	1560026_at	Hypothetical protein LOC149402	*LOC149401*
6013	25,4	20,3	1,25	0,048892	0,445	213252_at	SH3 and PX domains 2A	*SH3PXD2A*
921	4,73	3,77	1,25	0,005307	0,313	206033_s_at	Desmocollin 3	*DSC3*
3601	43,8	35,0	1,25	0,025975	0,394	204423_at	Muskelin 1, intracellular mediator containing kelch motifs	*MKLN1*
4371	168	134	1,25	0,032522	0,407	218528_s_at	Ring finger protein 38	*RNF38*
3305	117	93,5	1,25	0,023512	0,388	209175_at	SEC23 interacting protein	*SEC23IP*
4247	192	153	1,25	0,031536	0,406	225264_at	Arginyl-tRNA synthetase 2, mitochondrial (putative)	*RARS2*
5378	6,05	4,83	1,25	0,042175	0,429	216683_at	Tubulin folding cofactor A	*TBCA*
4170	12,3	9,82	1,25	0,030811	0,404	235471_at	Chromosome 10 open reading frame 72	*C10orf72*
4143	19,4	15,5	1,25	0,030568	0,403	243659_at	Elongation factor, RNA polymerase II, 2	*ELL2*
5324	611	488	1,25	0,041620	0,427	208623_s_at	Villin 2 (ezrin)	*VIL2*
749	30,1	24,0	1,25	0,004248	0,308	1556067_a_a	Jumonji domain containing 3	*JMJD3*
4584	71,5	57,2	1,25	0,034644	0,412	226323_at	Coiled-coil domain containing 16	*CCDC16*
3041	170	136	1,25	0,021427	0,385	201813_s_at	TBC1 domain family, member 5	*TBC1D5*
1639	247	197	1,25	0,010332	0,344	203250_at	RNA binding motif protein 16	*RBM16*
2736	7,87	6,29	1,25	0,018875	0,377	237208_at	WD repeat domain 61	*WDR61*
5216	12,0	9,60	1,25	0,040304	0,422	239957_at	SET domain containing 5	*SETD5*
143	9,42	7,53	1,25	0,000653	0,244	244168_s_at	Unc-51-like kinase 4 (C. elegans)	*ULK4*
5760	68,9	55,1	1,25	0,046272	0,439	209533_s_at	Phospholipase A2-activating protein	*PLAA*
3204	288	230	1,25	0,022686	0,387	225032_at	Fibronectin type III domain containing 3B	*FNDC3B*
1163	11,0	8,83	1,25	0,006851	0,321	242280_x_at	Cytoplasmic polyadenylation element binding protein 4	*CPEB4*
3494	10,7	8,52	1,25	0,025115	0,392	1565567_at	Syntaxin 7	*STX7*
5572	179	143	1,25	0,044389	0,435	209432_s_at	cAMP responsive element binding protein 3	*CREB3*
5423	279	223	1,25	0,042677	0,430	212287_at	Suppressor of zeste 12 homolog (Drosophila)	*SUZ12*
4941	70,1	56,0	1,25	0,037913	0,419	219530_at	Partner and localizer of BRCA2	*PALB2*
5722	186	149	1,25	0,045857	0,438	221825_at	Angel homolog 2 (Drosophila)	*ANGEL2*
5645	62,1	49,7	1,25	0,045052	0,436	213412_at	Tight junction protein 3 (zona occludens 3)	*TJP3*
3830	102	81,5	1,25	0,027976	0,399	226073_at	Transmembrane protein 218	*TMEM218*
282	14,3	11,4	1,25	0,001387	0,263	220171_x_at	KIAA1704 protein	*KIAA1704*
1902	195	156	1,25	0,012370	0,355	218082_s_at	Upstream binding protein 1 (LBP-1a)	*UBP1*
518	249	199	1,25	0,002756	0,291	202122_s_at	Mannose-6-phosphate receptor binding protein 1	*M6PRBP1*
5092	154	123	1,25	0,039177	0,420	222148_s_at	RAS homolog gene family, member T1	*RHOT1*
2577	77,8	62,3	1,25	0,017485	0,371	201085_s_at	SON DNA binding protein	*SON*
2866	21,9	17,5	1,25	0,020114	0,383	204501_at	Nephroblastoma overexpressed gene	*NOV*
2147	9,32	7,46	1,25	0,014131	0,360	205503_at	Protein tyrosine phosphatase, non-receptor type 14	*PTPN14*
4916	104	82,9	1,25	0,037628	0,418	226155_at	Family with sequence similarity 160, member B1	*FAM161B1*
4103	27,7	22,2	1,25	0,030150	0,402	205205_at	V-rel reticuloendotheliosis viral oncogene homolog B	*RELB*
3480	88,8	71,1	1,25	0,024981	0,392	202725_at	Polymerase (RNA) II (DNA directed) polypeptide A, 220kDa	*POLR2A*
6029	23,2	18,6	1,25	0,049057	0,445	230592_at	NSL1, MIND kinetochore complex component, homolog (S. cerevisiae)	*NSL1*
6035	14,3	11,4	1,25	0,049118	0,445	229794_at	Zinc finger protein 585A	*ZNF585A*
5721	83,6	67,0	1,25	0,045828	0,438	235056_at	Ets variant gene 6 (TEL oncogene)	*ETV6*
2884	231	185	1,25	0,020237	0,383	209484_s_at	NSL1, MIND kinetochore complex component, homolog (S. cerevisiae)	*NSL1*
434	9,57	7,67	1,25	0,002286	0,288	238851_at	Ankyrin repeat domain 13A	*ANKRD13A*
5670	384	308	1,25	0,045249	0,436	213455_at	Family with sequence similarity 114, member A1	*FAM114A1*
3465	182	146	1,25	0,024833	0,392	231968_at	UDP-glucose glycoprotein glucosyltransferase 1	*UGCGL1*
4133	74,1	59,4	1,25	0,030413	0,402	218221_at	Aryl hydrocarbon receptor nuclear translocator	*ARNT*
3415	6,87	5,51	1,25	0,024385	0,390	1569512_at	Supervillin	*SVIL*
2739	218	175	1,25	0,018899	0,377	200596_s_at	Eukaryotic translation initiation factor 3, subunit A	*EIF3A*

Index	Mutated*	Normal*	FC	*P* value	FDR**	Probe set	Description	Gene symbol
3821	10,8	8,66	1,25	0,027890	0,399	234151_at	CUG triplet repeat, RNA binding protein 2	*CUGBP2*
4321	66,2	53,1	1,25	0,032173	0,407	238653_at	Leucine-rich repeats and immunoglobulin-like domains 2	*LRIG2*
4595	105	84,4	1,25	0,034766	0,412	225317_at	Acyl-Coenzyme A binding domain containing 6	*ACBD6*
3899	14,7	11,8	1,25	0,028482	0,399	226627_at	Septin 8	*SEPT8*
4922	39,2	31,4	1,25	0,037708	0,419	206853_s_at	Mitogen-activated protein kinase kinase kinase 7	*MAP3K7*
4023	117	94,0	1,25	0,029457	0,400	239891_x_at	RAB12, member RAS oncogene family	*RAB12*
4229	12,5	10,1	1,25	0,031335	0,405	238243_at	cDNA clone YI43B12	-
4324	299	240	1,25	0,032183	0,407	202038_at	Ubiquitination factor E4A (UFD2 homolog, yeast)	*UBE4A*
2037	6,11	4,91	1,25	0,013271	0,355	223867_at	Tektin 3	*TEKT3*
1045	121	97,4	1,25	0,006113	0,319	201473_at	Jun B proto-oncogene	*JUNB*
5222	1013	814	1,24	0,040381	0,423	201170_s_at	Basic helix-loop-helix domain containing, class B, 2	*BHLHB2*
5426	564	453	1,24	0,042688	0,430	200760_s_at	ADP-ribosylation-like factor 6 interacting protein 5	*ARL6IP5*
3500	82,7	66,4	1,24	0,025153	0,392	203723_at	Inositol 1,4,5-trisphosphate 3-kinase B	*ITPKB*
5907	123	99,0	1,24	0,047874	0,443	212538_at	Dedicator of cytokinesis 9	*DOCK9*
1652	341	274	1,24	0,010398	0,344	215088_s_at	Succinate dehydrogenase cytochrome b560 subunit, mitochondrial	*SDHC*
5003	261	210	1,24	0,038419	0,420	201845_s_at	RING1 and YY1 binding protein	*RYBP*
553	5,31	4,27	1,24	0,003007	0,296	239571_at	Myocyte enhancer factor 2A	*MEF2A*
462	253	204	1,24	0,002457	0,288	212086_x_at	Lamin A/C	*LMNA*
1322	8,33	6,70	1,24	0,007893	0,326	39548_at	Neuronal PAS domain protein 2	*NPAS2*
3453	116	93,6	1,24	0,024725	0,391	221786_at	Chromosome 6 open reading frame 120	*C6orf120*
4039	123	98,6	1,24	0,029594	0,401	225050_at	Zinc finger protein 512	*ZNF512*
5410	189	152	1,24	0,042490	0,429	226126_at	TBC1 domain containing kinase	*TBCK*
5637	94,9	76,3	1,24	0,044983	0,436	226178_at	Suppressor of cytokine signaling 4	*SOCS4*
5777	103	82,5	1,24	0,046501	0,440	223269_at	Polymerase (RNA) III (DNA directed) polypeptide G (32kD)-like	*POLR3GL*
3445	104	83,8	1,24	0,024655	0,391	212603_at	Mitochondrial ribosomal protein S31	*MRPS31*
1270	58,8	47,3	1,24	0,007545	0,325	204711_at	KIAA0753 protein	*KIAA0753*
3431	93,5	75,3	1,24	0,024567	0,391	214429_at	Myotubularin related protein 6	*MTMR6*
2015	58,6	47,2	1,24	0,013146	0,355	222955_s_at	Family with sequence similarity 45, member A	*FAM45A*
5198	137	110	1,24	0,040105	0,422	223007_s_at	Chromosome 9 open reading frame 5	*C9orf5*
1305	13,4	10,8	1,24	0,007774	0,325	221854_at	Plakophilin 1	*PKP1*
3236	113	90,7	1,24	0,022942	0,388	207513_s_at	Zinc finger protein 189	*ZNF189*
4701	9,74	7,85	1,24	0,035496	0,413	207016_s_at	Aldehyde dehydrogenase 1 family, member A2	*ALDH1A2*
2474	16,0	12,9	1,24	0,016644	0,367	228913_at	Hypothetical protein LOC100190939	*LOC100190939*
2357	216	174	1,24	0,015726	0,364	203338_at	Protein phosphatase 2, regulatory subunit B', epsilon isoform	*PPP2R5E*
5704	134	108	1,24	0,045657	0,438	204771_s_at	Transcription termination factor, RNA polymerase I	*TTF1*
3182	181	146	1,24	0,022556	0,387	224445_s_at	Zinc finger, FYVE domain containing 21	*ZFYVE21*
3887	92,3	74,4	1,24	0,028395	0,399	1555789_s_a	Nucleoporin 98kDa	*NUP98*
1152	4,69	3,78	1,24	0,006776	0,321	1553894_at	Coiled-coil domain containing 122	*CCDC122*
3173	79,3	64,0	1,24	0,022506	0,387	213940_s_at	Formin binding protein 1	*FNBP1*
2775	109	87,6	1,24	0,019266	0,380	205917_at	Zinc finger protein 264	*ZNF264*
5607	79,7	64,3	1,24	0,044717	0,436	223221_at	SCO cytochrome oxidase deficient homolog 1 (yeast)	*SCO1*
606	8,30	6,70	1,24	0,003362	0,303	215428_at	tight junction protein 1 (zona occludens 1)	*TJP1*
2511	73,0	58,9	1,24	0,016885	0,368	209073_s_at	Numb homolog (Drosophila)	*NUMB*
478	9,72	7,85	1,24	0,002562	0,289	227226_at	Chromosome 6 open reading frame 117	*C6orf117*
4797	41,6	33,6	1,24	0,036388	0,415	224304_x_at	Ninein (GSK3B interacting protein)	*NIN*
4789	47,4	38,3	1,24	0,036311	0,415	1554762_a_a	WW and C2 domain containing 2	*WWC2*
497	24,2	19,5	1,24	0,002648	0,289	240095_at	Transcribed locus	-
4442	96,7	78,0	1,24	0,033367	0,411	204908_s_at	B-cell CLL/lymphoma 3	*BCL3*
3792	153	123	1,24	0,027619	0,398	213123_at	Microfibrillar-associated protein 3	*MFAP3*
4118	234	189	1,24	0,030275	0,402	224572_s_at	Interferon regulatory factor 2 binding protein 2	*IRF2BP2*
5973	17,0	13,7	1,24	0,048503	0,444	227839_at	Methyl-CpG binding domain protein 5	*MBD5*
1834	4,51	3,65	1,24	0,011808	0,351	232958_at	phosphorylase, glycogen	*PYGL*
4512	135	109	1,24	0,034012	0,412	219276_x_at	Chromosome 9 open reading frame 82	*C9orf82*
4553	493	398	1,24	0,034415	0,412	200765_x_at	Catenin (cadherin-associated protein), alpha 1, 102kDa	*CTNNA1*
5084	51,1	41,3	1,24	0,039131	0,420	213736_at	Cytochrome c oxidase subunit Vb	*COX5B*
2666	12,0	9,70	1,24	0,018229	0,374	239162_at	Death-associated protein kinase 1	*DAPK1*
5673	236	191	1,24	0,045279	0,436	228992_at	Mediator complex subunit 28	*MED28*

Index	Mutated*	Normal*	FC	*P* value	FDR**	Probe set	Description	Gene symbol
1641	202	163	1,24	0,010340	0,344	221699_s_at	DEAD (Asp-Glu-Ala-Asp) box polypeptide 50	*DDX50*
3391	57,7	46,7	1,24	0,024157	0,389	214672_at	Tubulin tyrosine ligase-like family, member 5	*TTLL5*
1836	143	115	1,24	0,011814	0,351	218572_at	Chromatin modifying protein 4A	*CHMP4A*
5475	33,0	26,7	1,24	0,043287	0,432	232724_at	Membrane-spanning 4-domains, subfamily A, member 6A	*MS4A6A*
2922	30,5	24,7	1,24	0,020495	0,383	216174_at	Hepatocellular carcinoma-related HCRP1	*HCRP1*
4656	172	139	1,24	0,035159	0,413	203447_at	Proteasome (prosome, macropain) 26S subunit, non-ATPase, 5	*PSMD5*
5885	33,0	26,7	1,24	0,047614	0,442	241348_at	Zinc finger protein 654	*ZNF654*
2860	92,4	74,7	1,24	0,020095	0,383	223182_s_at	1-acylglycerol-3-phosphate O-acyltransferase 3	*AGPAT3*
5331	9,85	7,97	1,24	0,041741	0,428	1560112_at	WD repeat and FYVE domain containing 2	*WDFY2*
3794	95,6	77,4	1,24	0,027639	0,398	220326_s_at	Hypothetical protein FLJ10357	*FLJ10357*
5186	21,6	17,5	1,24	0,039970	0,421	239266_at	Small nucleolar RNA host gene 5 (non-protein coding)	*SNHG5*
3512	26,2	21,2	1,23	0,025218	0,392	226220_at	Methyltransferase like 9	*METTL9*
665	32,1	26,0	1,23	0,003832	0,308	227411_at	Wilms tumor 1 interacting protein	*WTIP*
788	5,27	4,27	1,23	0,004507	0,311	239661_at	AF4/FMR2 family, member 1	*AFF1*
3974	879	712	1,23	0,029043	0,400	210844_x_at	Catenin (cadherin-associated protein), alpha 1, 102kDa	*CTNNA1*
5046	179	145	1,23	0,038889	0,420	212694_s_at	Propionyl Coenzyme A carboxylase, beta polypeptide	*PCCB*
186	38,8	31,5	1,23	0,000862	0,244	223401_at	Chromosome 17 open reading frame 48	*C17orf48*
3259	128	104	1,23	0,023143	0,388	222531_s_at	Chromosome 14 open reading frame 108	*C14orf108*
1864	7,63	6,19	1,23	0,012028	0,353	243826_at	Nuclear receptor coactivator 1	*NCOA1*
6043	126	102	1,23	0,049222	0,445	225159_s_at	Mediator complex subunit 28	*MED28*
3517	133	108	1,23	0,025251	0,392	218616_at	Integrator complex subunit 12	*INTS12*
1815	227	184	1,23	0,011710	0,351	226347_at	Fucosyltransferase 11 (alpha (1,3) fucosyltransferase)	*FUT11*
2933	44,5	36,1	1,23	0,020572	0,383	219169_s_at	Transcription factor B1, mitochondrial	*TFB1M*
2883	867	703	1,23	0,020225	0,383	200797_s_at	Myeloid cell leukemia sequence 1 (BCL2-related)	*MCL1*
2962	795	645	1,23	0,020812	0,384	224731_at	High-mobility group box 1	*HMGB1*
4318	137	111	1,23	0,032157	0,407	203719_at	DNA excision repair protein ERCC-1	*ERCC1*
1261	187	152	1,23	0,007505	0,325	202225_at	v-crk sarcoma virus CT10 oncogene homolog	*CRK*
3559	130	105	1,23	0,025693	0,394	212264_s_at	Wings apart-like homolog (Drosophila)	*WAPAL*
4360	63,7	51,8	1,23	0,032427	0,407	218873_at	Gon-4-like (C. elegans)	*GON4L*
3971	23,8	19,3	1,23	0,029015	0,399	212758_s_at	Zinc finger E-box binding homeobox 1	*ZEB1*
5250	41,7	33,9	1,23	0,040672	0,423	203254_s_at	Talin 1	*TLN1*
1800	27,0	22,0	1,23	0,011584	0,351	204136_at	Collagen, type VII, alpha 1	*COL7A1*
1022	12,7	10,4	1,23	0,005924	0,316	230305_at	Transcribed locus	-
2360	40,8	33,2	1,23	0,015746	0,364	213728_at	Lysosomal-associated membrane protein 1	*LAMP1*
4805	7,07	5,75	1,23	0,036468	0,415	1555123_at	ST6 beta-galactosamide alpha-2,6-sialyltranferase 2	*ST6GAL2*
3728	14,3	11,6	1,23	0,027064	0,397	1558445_at	cDNA clone IMAGE:5277883	-
3783	118	95,6	1,23	0,027558	0,398	201624_at	Aspartyl-tRNA synthetase	*DARS*
4781	223	181	1,23	0,036268	0,414	212066_s_at	Ubiquitin specific peptidase 34	*USP34*
3538	253	206	1,23	0,025451	0,393	225144_at	Bone morphogenetic protein receptor, type II	*BMPR2*
6120	446	362	1,23	0,049977	0,446	222431_at	Spindlin 1	*SPIN1*
3590	6,34	5,16	1,23	0,025896	0,394	242856_at	cDNA FLJ10247 fis, clone HEMBB1000705	-
4579	43,9	35,7	1,23	0,034610	0,412	203592_s_at	Follistatin-like 3 (secreted glycoprotein)	*FSTL3*
2618	5,28	4,30	1,23	0,017785	0,371	206983_at	Chemokine (C-C motif) receptor 6	*CCR6*
1429	7,96	6,48	1,23	0,008672	0,331	243648_at	Zinc finger CCCH-type containing 11A	*ZC3H11A*
6025	468	381	1,23	0,049022	0,445	200798_x_at	Myeloid cell leukemia sequence 1 (BCL2-related)	*MCL1*
5744	89,6	72,9	1,23	0,046078	0,439	213063_at	Zinc finger CCCH-type containing 14	*ZC3H14*
1470	4,76	3,88	1,23	0,008989	0,334	233139_at	Ataxin 2-binding protein 1	*A2BP1*
383	300	245	1,23	0,001992	0,284	218571_s_at	Chromatin modifying protein 4A	*CHMP4A*
2740	57,2	46,6	1,23	0,018900	0,377	220935_s_at	CDK5 regulatory subunit associated protein 2	*CDK5RAP2*
4962	185	151	1,23	0,038055	0,419	218139_s_at	Chromosome 14 open reading frame 108	*C14orf108*
3746	24,9	20,3	1,23	0,027243	0,397	232637_at	Septin 2	*SEPT2*
6057	642	523	1,23	0,049408	0,446	208636_at	Actinin, alpha 1	*ACTN1*
5176	27,7	22,5	1,23	0,039888	0,421	209203_s_at	Bicaudal D homolog 2 (Drosophila)	*BICD2*
2876	9,83	8,01	1,23	0,020194	0,383	231471_at	zinc finger protein 638	*ZNF638*
5960	247	201	1,23	0,048384	0,444	225336_at	Splicing factor, arginine/serine-rich 2, interacting protein	*SFRS2IP*
997	4,72	3,85	1,23	0,005755	0,316	238297_at	Phosphatase and actin regulator 1	*PHACTR1*
572	212	173	1,23	0,003144	0,300	243_g_at	Microtubule-associated protein 4	*MAP4*

Index	Mutated*	Normal*	FC	*P* value	FDR**	Probe set	Description	Gene symbol
3109	405	330	1,23	0,021947	0,386	215096_s_at	Esterase D/formylglutathione hydrolase	*ESD*
1805	23,5	19,1	1,23	0,011624	0,351	227587_at	KRI1 homolog (S. cerevisiae)	*KRI1*
4398	16,5	13,4	1,23	0,032783	0,407	232890_at	-	-
5741	40,4	33,0	1,23	0,046067	0,439	210210_at	Myelin protein zero-like 1	*MPZL1*
2899	83,4	68,1	1,23	0,020348	0,383	223356_s_at	Mitochondrial translational initiation factor 3	*MTIF3*
2757	97,3	79,4	1,23	0,019126	0,379	208878_s_at	p21 (CDKN1A)-activated kinase 2	*PAK2*
5088	26,6	21,7	1,23	0,039159	0,420	241460_at	Ribosomal protein S6 kinase, 90 kDa, polypeptide 3	*RPS6KA2*
2723	7,24	5,91	1,23	0,018710	0,376	232964_at	Williams Beuren syndrome chromosome region 19	*WBSCR19*
2469	390	318	1,23	0,016588	0,367	208631_s_at	3-ketoacyl-Coenzyme A (CoA) thiolase, alpha subunit	*HADHA*
3653	695	567	1,22	0,026402	0,395	208684_at	Coatomer protein complex, subunit alpha	*COPA*
4860	11,4	9,35	1,22	0,037078	0,417	236808_at	FGFR1 oncogene partner 2	*FGFR1OP2*
4381	9,21	7,52	1,22	0,032594	0,407	217655_at	FXYD domain containing ion transport regulator 5	*FXYD5*
3567	5,39	4,40	1,22	0,025744	0,394	216563_at	Ankyrin repeat domain 12	*ANKRD12*
830	4,40	3,59	1,22	0,004743	0,312	239788_at	Suppressor of mec-8 and unc-52 homolog (C. elegans)	*SMU1*
2313	327	268	1,22	0,015366	0,363	202232_s_at	Eukaryotic translation initiation factor 3, subunit M	*EIF3M*
2309	96,1	78,5	1,22	0,015326	0,363	218153_at	Cysteinyl-tRNA synthetase 2, mitochondrial (putative)	*CARS2*
4306	6,00	4,90	1,22	0,032039	0,407	1560201_at	Zinc finger protein 713	*ZNF713*
800	24,3	19,9	1,22	0,004560	0,311	1553286_at	Zinc finger protein 555	*ZNF555*
924	472	386	1,22	0,005324	0,313	205480_s_at	UDP-glucose pyrophosphorylase 2	*UGP2*
3461	329	269	1,22	0,024790	0,392	212377_s_at	Notch homolog 2 (Drosophila)	*NOTCH2*
1452	538	440	1,22	0,008870	0,334	201007_at	Hydroxyacyl-Coenzyme A (CoA) dehydrogenase, beta subunit	*HADHB*
5668	54,6	44,7	1,22	0,045235	0,436	226251_at	Additional sex combs like 2 (Drosophila)	*ASXL2*
5314	93,1	76,2	1,22	0,041467	0,427	229650_s_at	Chromosome 19 open reading frame 42	*C19orf42*
5055	144	118	1,22	0,038950	0,420	225477_s_at	Nuclear receptor subfamily 2, group C, member 2	*NR2C2*
607	5,87	4,81	1,22	0,003400	0,306	1570165_at	carbohydrate (chondroitin 4) sulfotransferase 11	*CHST11*
5954	263	216	1,22	0,048297	0,444	200764_s_at	Catenin (cadherin-associated protein), alpha 1, 102kDa	*CTNNA1*
3694	13,5	11,1	1,22	0,026711	0,395	237398_at	Transcribed locus	-
4578	65,8	53,9	1,22	0,034604	0,412	218588_s_at	Chromosome 5 open reading frame 3	*C5orf3*
5027	22,7	18,6	1,22	0,038646	0,420	213328_at	NIMA (never in mitosis gene a)-related kinase 1	*NEK1*
5740	62,8	51,5	1,22	0,046057	0,439	220720_x_at	Family with sequence similarity 128, member B	*FAM128B*
5634	9,47	7,76	1,22	0,044977	0,436	1552261_at	WAP four-disulfide core domain 2	*WFDC2*
4577	122	100	1,22	0,034602	0,412	243916_x_at	Ubiquitin-like domain containing CTD phosphatase 1	*UBLCP1*
4284	63,5	52,0	1,22	0,031841	0,406	235013_at	Solute carrier family 31 (copper transporters), member 1	*SLC31A1*
5313	21,1	17,3	1,22	0,041461	0,427	242167_at	Chromosome 10 open reading frame 119	*C10orf119*
3420	83,0	68,1	1,22	0,024456	0,391	203822_s_at	E74-like factor 2 (ets domain transcription factor)	*ELF2*
4231	6,49	5,33	1,22	0,031356	0,405	235761_at	Tyrosylprotein sulfotransferase 2	*TPST2*
249	7,53	6,18	1,22	0,001142	0,251	231698_at	Hypothetical protein LOC647115	*FLJ36848*
4547	63,3	52,0	1,22	0,034366	0,412	213302_at	Phosphoribosylformylglycinamidine synthase	*PFAS*
1328	7,41	6,08	1,22	0,007996	0,328	233817_at	Neuroblastoma breakpoint family, member 10	*NBPF10*
4109	6,14	5,05	1,22	0,030199	0,402	232924_at	Leucine rich repeat containing 17	*LRRC17*
3833	34,5	28,3	1,22	0,028012	0,399	205665_at	Tetraspanin 9	*TSPAN9*
5125	98,2	80,8	1,22	0,039444	0,421	203334_at	DEAH (Asp-Glu-Ala-His) box polypeptide 8	*DHX8*
3888	30,8	25,4	1,22	0,028397	0,399	242981_at	Cytochrome P450, family 3, subfamily A, polypeptide 5	*CYP3A5*
2152	64,5	53,1	1,22	0,014179	0,360	228075_x_at	Transcription factor B1, mitochondrial	*TFB1M*
1442	14,1	11,6	1,22	0,008770	0,332	227575_s_at	Chromosome 14 open reading frame 102	*C14orf102*
5728	34,9	28,7	1,22	0,045939	0,438	37462_i_at	Splicing factor 3a, subunit 2, 66kDa	*SF3A2*
2861	345	284	1,22	0,020096	0,383	213531_s_at	RAB3 GTPase activating protein subunit 1 (catalytic)	*RAB3GAP1*
843	8,48	6,98	1,21	0,004829	0,313	233689_at	Transcription factor 7-like 2 (T-cell specific, HMG-box)	*TCF7L2*
4416	314	259	1,21	0,033028	0,409	218659_at	Additional sex combs like 2 (Drosophila)	*ASXL2*
2380	62,4	51,4	1,21	0,015858	0,364	217579_x_at	Atlastin GTPase 2	*ATL2*
3759	75,9	62,6	1,21	0,027317	0,397	226958_s_at	Mediator complex subunit 11	*MED11*
4896	265	219	1,21	0,037418	0,418	202160_at	CREB binding protein	*CREBBP*
5767	42,4	35,0	1,21	0,046339	0,439	218489_s_at	Aminolevulinate, delta-, dehydratase	*ALAD*
4822	45,9	37,8	1,21	0,036674	0,416	223330_s_at	SGT1, suppressor of G2 allele of SKP1 (S. cerevisiae)	*SUGT1*
3721	7,26	5,98	1,21	0,027006	0,397	1552536_at	Vesicle transport v-SNARE protein Vti1-like 2	*VTI1A*
2537	23,4	19,3	1,21	0,017162	0,369	216751_at	cDNA FLJ21226 fis, clone COL00721	-
5966	13,7	11,3	1,21	0,048449	0,444	211002_s_at	Tripartite motif-containing 29	*TRIM29*

Index	Mutated*	Normal*	FC	*P* value	FDR**	Probe set	Description	Gene symbol
5212	143	118	1,21	0,040252	0,422	226339_at	TruB pseudouridine (psi) synthase homolog 1 (E. coli)	*TRUB1*
4567	584	481	1,21	0,034549	0,412	209009_at	Esterase D/formylglutathione hydrolase	*ESD*
4217	12,4	10,2	1,21	0,031244	0,405	1558469_at	LIM domain containing preferred translocation partner in lipoma	*LPP*
3154	48,8	40,2	1,21	0,022373	0,387	205652_s_at	Tubulin tyrosine ligase-like family, member 1	*TTLL1*
2359	6,84	5,64	1,21	0,015733	0,364	227136_s_at	Chromosome 10 open reading frame 46	*C10orf46*
1814	6,52	5,38	1,21	0,011689	0,351	220623_s_at	Testis specific, 10	*TSGA10*
4391	185	153	1,21	0,032717	0,407	207196_s_at	TNFAIP3 interacting protein 1	*TNIP1*
2450	7,66	6,32	1,21	0,016409	0,366	214319_at	Furry homolog (Drosophila)	*FRY*
5253	46,6	38,5	1,21	0,040706	0,424	227335_at	Death inducer-obliterator 1	*DIDO1*
5641	105	86,7	1,21	0,045014	0,436	209367_at	Syntaxin binding protein 2	*STXBP2*
4775	509	420	1,21	0,036211	0,414	202004_x_at	Succinate dehydrogenase cytochrome b560 subunit, mitochondrial	*SDHC*
3819	172	142	1,21	0,027872	0,399	201573_s_at	Eukaryotic translation termination factor 1	*ETF1*
3669	139	115	1,21	0,026520	0,395	202930_s_at	Succinate-CoA ligase, ADP-forming, beta subunit	*SUCLA2*
3549	5,01	4,14	1,21	0,025535	0,393	206862_at	Zinc finger protein 254	*ZNF254*
3268	59,9	49,5	1,21	0,023220	0,388	221822_at	Coiled-coil domain containing 101	*CCDC101*
5743	113	93,3	1,21	0,046078	0,439	224768_at	IWS1 homolog (S. cerevisiae)	*IWS1*
4780	174	143	1,21	0,036246	0,414	229322_at	Protein phosphatase 2, regulatory subunit B', epsilon isoform	*PPP2R5E*
3991	12,2	10,0	1,21	0,029187	0,400	1563512_at	Nitric oxide synthase 1 (neuronal) adaptor protein	*NOS1AP*
6018	6,08	5,02	1,21	0,048981	0,445	238185_at	RNA binding motif, single stranded interacting protein 1	*RBMS1*
6040	42,9	35,5	1,21	0,049189	0,445	212786_at	C-type lectin domain family 16, member A	*CLEC16A*
5274	9,48	7,84	1,21	0,040984	0,425	1565811_at	Protein phosphatase 2B regulatory subunit 1	*PPP3R1*
5706	181	150	1,21	0,045693	0,438	201706_s_at	Peroxisomal biogenesis factor 19	*PEX19*
5440	53,3	44,1	1,21	0,042880	0,431	223555_at	Gon-4-like (C. elegans)	*GON4L*
3828	76,4	63,2	1,21	0,027934	0,399	217843_s_at	Mediator complex subunit 4	*MED4*
5033	8,24	6,81	1,21	0,038702	0,420	234036_x_at	cDNA FLJ21384 fis, clone COL03354	-
2124	17,6	14,6	1,21	0,013985	0,359	235654_at	Transcribed locus	-
2963	5,74	4,75	1,21	0,020822	0,384	1553646_at	Highly divergent homeobox	*HDX*
970	69,3	57,4	1,21	0,005625	0,315	204568_at	KIAA0831 protein	*KIAA0831*
4527	91,7	75,9	1,21	0,034174	0,412	221006_s_at	sorting nexin family member 27	*SNX27*
3749	9,58	7,94	1,21	0,027264	0,397	242171_at	Platelet derived growth factor C	*PDGFC*
1787	9,78	8,10	1,21	0,011441	0,350	237026_at	Sorbin and SH3 domain containing 1	*SORBS1*
1227	122	101	1,21	0,007294	0,324	226054_at	Bromodomain containing 4	*BRD4*
2510	27,4	22,7	1,21	0,016885	0,368	227215_at	Golgi associated PDZ and coiled-coil motif containing	*GOPC*
4629	7,04	5,83	1,21	0,034913	0,412	222924_at	Sarcolemma associated protein	*SLMAP*
5539	62,3	51,6	1,21	0,044020	0,435	221256_s_at	Haloacid dehalogenase-like hydrolase domain containing 3	*HDHD3*
3462	635	527	1,21	0,024800	0,392	225312_at	COMM domain containing 6	*COMMD6*
2001	164	136	1,21	0,013067	0,355	224699_s_at	Extended synaptotagmin-like protein 2	*ESYT2*
5770	281	233	1,21	0,046353	0,439	202026_at	Succinate dehydrogenase complex subunit D	*SDHD*
4250	132	109	1,21	0,031565	0,406	202102_s_at	Bromodomain containing 4	*BRD4*
6048	40,4	33,5	1,21	0,049281	0,445	227579_at	-	-
4534	435	361	1,21	0,034252	0,412	223006_s_at	Chromosome 9 open reading frame 5	*C9orf5*
4655	229	190	1,21	0,035159	0,413	225229_at	AF4/FMR2 family, member 4	*AFF4*
3969	68,8	57,1	1,21	0,029000	0,399	203328_x_at	Insulin-degrading enzyme	*IDE*
2877	150	124	1,20	0,020196	0,383	226468_at	Zinc finger protein 364	*ZNF364*
3858	6,30	5,23	1,20	0,028173	0,399	205560_at	Proprotein convertase subtilisin/kexin type 5	*PCSK5*
3738	115	95,1	1,20	0,027140	0,397	218102_at	2-deoxyribose-5-phosphate aldolase homolog (C. elegans)	*DERA*
1633	11,2	9,31	1,20	0,010290	0,344	231366_at	MGC44478	*FDPSL2A*
5569	234	194	1,20	0,044333	0,435	225519_at	Protein phosphatase 4, regulatory subunit 2	*PPP4R2*
3945	4,14	3,44	1,20	0,028807	0,399	220666_at	-	-
4846	9,82	8,16	1,20	0,036920	0,417	220273_at	Interleukin 17B	*IL17B*
3600	10,2	8,51	1,20	0,025962	0,394	231167_at	Solute carrier family 44, member 1	*SLC44A1*
1900	77,6	64,4	1,20	0,012354	0,355	204812_at	ZW10, kinetochore associated, homolog (Drosophila)	*ZW10*
3955	155	129	1,20	0,028895	0,399	225417_at	Enhancer of polycomb homolog 1 (Drosophila)	*EPC1*
4074	6,33	5,26	1,20	0,029882	0,401	232427_at	Zinc finger protein 224	*ZNF224*
2807	9,30	7,73	1,20	0,019529	0,380	231369_at	Zinc finger protein 333	*ZNF333*
5838	63,8	53,1	1,20	0,047180	0,442	206782_s_at	DnaJ (Hsp40) homolog, subfamily C, member 4	*DNAJC4*
2155	5,24	4,36	1,20	0,014200	0,360	237248_at	Phosphodiesterase 11A	*PDE11A*

Index	Mutated*	Normal*	FC	*P* value	FDR**	Probe set	Description	Gene symbol
16	3,66	3,04	1,20	0,000056	0,191	239129_at	Transforming growth factor, beta receptor 1	*TGFBR1*
5011	5,59	4,65	1,20	0,038515	0,420	1557113_at	Hypothetical protein LOC283588	*LOC283588*
575	12,7	10,6	1,20	0,003159	0,300	236199_at	Arachidonate 5-lipoxygenase	*ALOX5*
3011	21,6	18,0	1,20	0,021185	0,385	214757_at	Postmeiotic segregation increased 2-like 2 pseudogene	*PMS2L2*
2520	67,0	55,8	1,20	0,017004	0,369	213349_at	Transmembrane and coiled-coil domain family 1	*TMCC1*
4872	90,3	75,2	1,20	0,037190	0,417	212080_at	Myeloid/lymphoid or mixed-lineage leukemia	*MLL*
1899	126	105	1,20	0,012352	0,355	219526_at	Chromosome 14 open reading frame 169	*C14orf169*
4927	14,5	12,1	1,20	0,037792	0,419	216685_s_at	Methylthioadenosine phosphorylase	*MTAP*
2593	5,01	4,18	1,20	0,017627	0,371	237376_at	Cullin 3	*CUL3*
4945	24,8	20,7	1,20	0,037937	0,419	201333_s_at	Rho guanine nucleotide exchange factor (GEF) 12	*ARHGEF12*
4764	57,7	48,1	1,20	0,036108	0,414	227541_at	WD repeat domain 20	*WDR20*
2199	181	151	1,20	0,014533	0,361	223081_at	PHD finger protein 23	*PHF23*
1769	9,42	7,86	1,20	0,011275	0,348	240176_at	Hypothetical protein LOC541471	*LOC541471*
913	7,10	5,93	1,20	0,005256	0,313	232930_at	Dedicator of cytokinesis 1	*DOCK1*
5848	5,98	4,99	1,20	0,047290	0,442	243352_at	Alpha-kinase 1	*ALPK1*
3213	13,6	11,3	1,20	0,022767	0,387	232940_s_at	Myeloid/lymphoid or mixed-lineage leukemia 3	*MLL3*
5227	24,0	20,0	1,20	0,040437	0,423	1559249_at	Ataxin 1	*ATXN1*
3577	16,9	14,1	1,20	0,025825	0,394	243201_at	Heterogeneous nuclear ribonucleoprotein H2	*HNRPH2*
262	5,23	4,37	1,20	0,001237	0,258	227939_s_at	Transformer-2 alpha	*TRA2A*
5262	5,05	4,22	1,20	0,040873	0,425	232303_at	Zinc finger protein 608	*ZNF608*
3807	23,0	19,2	1,20	0,027721	0,398	219075_at	Yip1 domain family, member 2	*YIPF2*
4592	4,61	3,86	1,20	0,034744	0,412	238811_at	ATPase, Class VI, type 11B	*ATP11B*
4664	9,19	7,69	1,20	0,035223	0,413	1554867_a_a	Proline rich 16	*PRR16*
3836	9,18	7,68	1,20	0,028032	0,399	238865_at	Similar to poly(A) binding protein, cytoplasmic 4 (inducible form)	*LOC132430*
5007	4,69	3,92	1,20	0,038469	0,420	219936_s_at	G protein-coupled receptor 87	*GPR87*
4652	60,4	50,5	1,19	0,035132	0,413	202584_at	Nuclear transcription factor, X-box binding 1	*NFX1*
2858	39,5	33,1	1,19	0,020070	0,383	228917_at	Zinc finger protein 510	*ZNF510*
2209	10,5	8,83	1,19	0,014633	0,362	237418_at	CDK5 regulatory subunit associated protein 2	*CDK5RAP2*
6123	10,7	8,98	1,19	0,049992	0,446	205894_at	Arylsulfatase E (chondrodysplasia punctata 1)	*ARSE*
5618	190	159	1,19	0,044833	0,436	201877_s_at	Protein phosphatase 2, regulatory subunit B', gamma isoform	*PPP2R5C*
5999	338	283	1,19	0,048771	0,444	201528_at	Replication protein A1, 70kDa	*RPA1*
2816	16,9	14,2	1,19	0,019638	0,381	212650_at	EH domain binding protein 1	*EHBP1*
3151	13,9	11,7	1,19	0,022368	0,387	237252_at	Thrombomodulin	*THBD*
3615	207	173	1,19	0,026088	0,395	221502_at	Karyopherin alpha 3 (importin alpha 4)	*KPNA3*
5039	17,3	14,5	1,19	0,038830	0,420	238827_at	SH3 domain containing ring finger 2	*SH3RF2*
5477	5,79	4,85	1,19	0,043305	0,432	214967_at	cDNA FLJ12055 fis, clone HEMBB1002049	-
4325	7,41	6,21	1,19	0,032186	0,407	238614_x_at	Zinc finger protein 430	*ZNF430*
5292	8,31	6,97	1,19	0,041199	0,426	1553216_at	Zinc finger protein 41	*ZNF41*
4361	7,35	6,16	1,19	0,032432	0,407	1557797_a_a	Zinc finger E-box binding homeobox 2	*ZEB2*
4307	46,5	39,0	1,19	0,032045	0,407	214783_s_at	Annexin A11	*ANXA11*
2742	64,4	54,0	1,19	0,018920	0,377	204928_s_at	Solute carrier family 10, member 3	*SLC10A3*
2702	5,18	4,35	1,19	0,018540	0,375	1566825_at	Rho GTPase activating protein 24	*ARHGAP24*
4424	62,2	52,2	1,19	0,033118	0,409	225234_at	Cas-Br-M (murine) ecotropic retroviral transforming sequence	*CBL*
4108	504	423	1,19	0,030195	0,402	206200_s_at	Annexin A11	*ANXA11*
3883	52,9	44,4	1,19	0,028348	0,399	237868_x_at	FK506 binding protein 15, 133kDa	*FKBP15*
5964	65,7	55,2	1,19	0,048437	0,444	233775_x_at	cDNA FLJ13242 fis, clone OVARC1000578	-
5388	8,98	7,55	1,19	0,042252	0,429	236241_at	Mediator complex subunit 31	*MED31*
2606	162	137	1,19	0,017699	0,371	235142_at	Zinc finger and BTB domain containing 8	*ZBTB8*
3241	497	418	1,19	0,022991	0,388	224936_at	Eukaryotic translation initiation factor 2, subunit 3 gamma	*EIF2S3*
4003	41,2	34,7	1,19	0,029304	0,400	238510_at	Zinc finger protein 720	*ZNF720*
5831	90,2	75,9	1,19	0,047123	0,442	214902_x_at	Hypothetical protein FLJ42393	*FLJ42393*
1533	31,0	26,1	1,19	0,009503	0,337	212078_s_at	Myeloid/lymphoid or mixed-lineage leukemia	*MLL*
1058	4,61	3,88	1,19	0,006206	0,319	232332_at	KIAA1210 protein	*RP13-347D8.3*
3424	46,6	39,2	1,19	0,024470	0,391	244406_at	Zinc finger protein 20	*ZNF20*
4088	5,15	4,33	1,19	0,030065	0,402	241242_at	Family with sequence similarity 120A	*FAM120A*
4019	13,1	11,1	1,19	0,029426	0,400	239023_at	CDC42 small effector 1	*CDC42SE1*
4919	49,2	41,4	1,19	0,037662	0,419	236027_at	Chromosome 10 open reading frame 78	*C10orf78*

Index	Mutated*	Normal*	FC	*P* value	FDR**	Probe set	Description	Gene symbol
3508	82,8	69,8	1,19	0,025201	0,392	205745_x_at	ADAM metallopeptidase domain 17	*ADAM17*
2718	5,75	4,85	1,19	0,018671	0,376	244655_at	Hypothetical protein LOC644192	*LOC644192*
3998	24,2	20,4	1,19	0,029266	0,400	203348_s_at	Ets variant gene 5 (ets-related molecule)	*ETV5*
2111	525	443	1,19	0,013848	0,358	202231_at	Eukaryotic translation initiation factor 3, subunit M	*EIF3M*
2787	120	102	1,19	0,019363	0,380	218147_s_at	Glycosyltransferase 8 domain containing 1	*GLT8D1*
5540	1104	931	1,19	0,044029	0,435	200761_s_at	ADP-ribosylation-like factor 6 interacting protein 5	*ARL6IP5*
4894	7,25	6,11	1,19	0,037400	0,418	1569538_at	Clone FLB8310 PRO2225	-
5975	20,2	17,1	1,19	0,048531	0,444	242297_at	RAS responsive element binding protein 1	*RREB1*
4141	9,44	7,97	1,18	0,030562	0,403	1558306_at	Thyroid adenoma associated	*THADA*
2138	44,7	37,7	1,18	0,014054	0,359	218466_at	TBC1 domain family, member 17	*TBC1D17*
5364	11,7	9,87	1,18	0,042082	0,429	232588_at	Stromal antigen 1	*STAG1*
4458	9,99	8,43	1,18	0,033495	0,411	227913_at	Exosome component 3	*EXOSC3*
3687	791	668	1,18	0,026627	0,395	211938_at	Eukaryotic translation initiation factor 4B	*EIF4B*
2490	10,8	9,10	1,18	0,016784	0,367	214605_x_at	G protein-coupled receptor 1	*GPR1*
988	8,17	6,90	1,18	0,005696	0,315	230790_x_at	Forkhead box N3	*FOXN3*
560	4,96	4,19	1,18	0,003054	0,298	234605_at	CDC14 cell division cycle 14 homolog B (S. cerevisiae)	*CDC14B*
5951	655	553	1,18	0,048273	0,444	226801_s_at	Axin interactor, dorsalization associated	*AIDA*
4161	10,7	9,04	1,18	0,030766	0,404	232428_at	Monoacylglycerol O-acyltransferase 2	*MOGAT2*
4085	10,6	8,97	1,18	0,030000	0,402	238027_at	Hypothetical protein LOC202051	*LOC202051*
5421	633	535	1,18	0,042622	0,430	208942_s_at	Translocation protein 1	*TLOC1*
2463	94,4	79,8	1,18	0,016526	0,367	204765_at	Rho guanine nucleotide exchange factor (GEF) 5	*ARHGEF5*
1503	459	388	1,18	0,009241	0,336	217874_at	succinate-CoA ligase, GDP-forming, alpha subunit	*SUCLG1*
4124	296	250	1,18	0,030332	0,402	224777_s_at	PAF acetylhydrolase 30 kDa subunit	*PAFAH1B2*
1172	6,82	5,77	1,18	0,006886	0,321	1557158_s_a	Myeloid/lymphoid or mixed-lineage leukemia 3	*MLL3*
1545	5,52	4,68	1,18	0,009545	0,337	1558754_at	Zinc finger protein 763	*ZNF763*
1855	9,53	8,07	1,18	0,011950	0,352	232465_at	Transcription factor 7-like 2 (T-cell specific, HMG-box)	*TCF7L2*
3122	5,95	5,04	1,18	0,022089	0,387	1567044_s_a	LSM14A, SCD6 homolog A (S. cerevisiae)	*LSM14A*
3357	9,84	8,34	1,18	0,023837	0,388	229274_at	GNAS complex locus	*GNAS*
6039	75,5	64,0	1,18	0,049159	0,445	225565_at	cAMP responsive element binding protein 1	*CREB1*
3531	5,44	4,61	1,18	0,025382	0,393	1565638_at	Peripheral myelin protein 22	*PMP22*
2698	11,4	9,64	1,18	0,018502	0,375	214000_s_at	Regulator of G-protein signalling 10	*RGS10*
1486	4,29	3,64	1,18	0,009150	0,336	220328_at	Polyhomeotic homolog 3 (Drosophila)	*PHC3*
1301	3738	3169	1,18	0,007743	0,325	211940_x_at	H3 histone, family 3A	*H3F3A*
5534	171	145	1,18	0,043938	0,434	204028_s_at	RAB GTPase activating protein 1	*RABGAP1*
664	286	243	1,18	0,003828	0,308	226052_at	Bromodomain containing 4	*BRD4*
6102	17,4	14,8	1,18	0,049810	0,446	226890_at	WD repeat domain 35	*WDR35*
2426	7,69	6,53	1,18	0,016220	0,365	1562059_at	Transmembrane 9 superfamily member 3	*TM9SF3*
5646	31,3	26,6	1,18	0,045055	0,436	213896_x_at	Family with sequence similarity 149, member B1	*FAM149B1*
5779	153	130	1,18	0,046525	0,440	202537_s_at	Chromatin modifying protein 2B	*CHMP2B*
1785	10,5	8,93	1,18	0,011402	0,349	203969_at	Peroxisomal biogenesis factor 3	*PEX3*
5151	30,0	25,4	1,18	0,039647	0,421	215718_s_at	PHD finger protein 3	*PHF3*
2377	6,55	5,56	1,18	0,015844	0,364	230258_at	GLIS family zinc finger 3	*GLIS3*
4691	204	173	1,18	0,035438	0,413	201434_at	Tetratricopeptide repeat domain 1	*TTC1*
2622	8,58	7,29	1,18	0,017816	0,371	235283_at	Integrator complex subunit 6	*INTS6*
4087	11,8	10,1	1,18	0,030029	0,402	232621_at	Ubiquitin specific peptidase 48	*USP48*
4970	96,7	82,2	1,18	0,038124	0,419	206551_x_at	Kelch-like 24 (Drosophila)	*KLHL24*
3119	12,9	11,0	1,18	0,022053	0,387	244698_at	CMT1A duplicated region transcript 4	*CDRT4*
1894	5,37	4,57	1,18	0,012314	0,355	214829_at	Aminoadipate-semialdehyde synthase	*AASS*
331	4,70	4,00	1,18	0,001647	0,272	242258_at	Zinc finger and BTB domain containing 1	*ZBTB1*
3676	27,6	23,5	1,18	0,026571	0,395	1557690_x_a	Neuronal PAS domain protein 2	*NPAS2*
5293	6,26	5,32	1,18	0,041222	0,426	242006_at	Leber congenital amaurosis 5	*LCA5*
5916	4,12	3,51	1,18	0,047949	0,443	206543_at	ATP-dependent helicase SMARCA2	*SMARCA2*
1756	5,67	4,83	1,18	0,011197	0,348	1562815_at	EF-hand calcium binding domain 6	*EFCAB6*
2440	5,29	4,50	1,17	0,016309	0,365	210697_at	Zinc finger protein 257	*ZNF257*
4902	76,0	64,7	1,17	0,037505	0,418	214316_x_at	Calreticulin	*CALR*
4075	3,44	2,93	1,17	0,029884	0,401	240292_x_at	Ankyrin repeat and sterile alpha motif domain containing 1B	*ANKS1B*
2607	5,75	4,89	1,17	0,017706	0,371	244691_at	SET domain containing 5	*SETD5*

Index	Mutated*	Normal*	FC	*P* value	FDR**	Probe set	Description	Gene symbol
4213	920	784	1,17	0,031202	0,405	217768_at	Chromosome 14 open reading frame 166	*C14orf166*
4021	6,36	5,42	1,17	0,029430	0,400	233253_at	cDNA FLJ14147 fis, clone MAMMA1002973	-
977	57,5	49,0	1,17	0,005671	0,315	235828_at	PRELI domain containing 2	*PRELID2*
3426	5,12	4,36	1,17	0,024496	0,391	243361_at	Splicing factor, arginine/serine-rich 12	*SFRS12*
2744	18,6	15,8	1,17	0,018935	0,377	219172_at	Ubiquitin domain containing 1	*UBTD1*
2930	19,8	16,8	1,17	0,020548	0,383	219380_x_at	Polymerase (DNA directed), eta	*POLH*
4920	257	219	1,17	0,037673	0,419	219598_s_at	RWD domain containing 1	*RWDD1*
2528	10,9	9,30	1,17	0,017077	0,369	214980_at	Ubiquitin protein ligase E3A	*UBE3A*
1963	4,35	3,71	1,17	0,012834	0,355	1558560_s_a	Basic leucine zipper nuclear factor 1 (JEM-1)	*BLZF1*
4040	19,6	16,7	1,17	0,029597	0,401	203945_at	Arginase, type II	*ARG2*
3747	5,82	4,96	1,17	0,027245	0,397	242456_at	MRE11 meiotic recombination 11 homolog A (S. cerevisiae)	*MRE11A*
5924	6,41	5,47	1,17	0,047997	0,443	239889_at	Chromosome 13 open reading frame 21	*C13orf21*
5488	84,2	71,8	1,17	0,043438	0,433	207268_x_at	Abl interactor 2	*ABI2*
5943	98,4	83,9	1,17	0,048165	0,443	1558511_s_a	Extended synaptotagmin-like protein 2	*ESYT2*
592	1152	984	1,17	0,003269	0,301	200677_at	Pituitary tumor-transforming 1 interacting protein	*PTTG1IP*
5042	35,6	30,4	1,17	0,038856	0,420	219247_s_at	Zinc finger, DHHC-type containing 14	*ZDHHC14*
1837	5,16	4,41	1,17	0,011815	0,351	1567457_at	RAS-related C3 botulinum toxin substrate 1	*RAC1*
5102	5,67	4,84	1,17	0,039232	0,420	208268_at	ADAM metallopeptidase domain 28	*ADAM28*
1502	49,6	42,4	1,17	0,009238	0,336	222195_s_at	Chromosome 9 open reading frame 156	*C9orf156*
5050	10,6	9,08	1,17	0,038907	0,420	214008_at	Twinfilin, actin-binding protein, homolog 1 (Drosophila)	*TWF1*
5357	132	113	1,17	0,042012	0,429	203057_s_at	PR domain containing 2, with ZNF domain	*PRDM2*
3043	28,3	24,2	1,17	0,021428	0,385	214028_x_at	Tudor domain containing 3	*TDRD3*
406	7,45	6,38	1,17	0,002133	0,286	214031_s_at	Keratin 7	*KRT7*
588	190	163	1,17	0,003249	0,301	1555945_s_a	Family with sequence similarity 120A	*FAM120A*
1701	51,4	44,0	1,17	0,010831	0,348	222492_at	Pyridoxal (pyridoxine, vitamin B6) kinase	*PDXK*
875	4,17	3,57	1,17	0,005050	0,313	236409_at	Lysophospholipase-like 1	*LYPLAL1*
5931	25,5	21,9	1,17	0,048084	0,443	209920_at	Bone morphogenetic protein receptor, type II	*BMPR2*
5947	211	181	1,17	0,048195	0,443	212399_s_at	Vestigial like 4 (Drosophila)	*VGLL4*
3277	523	448	1,17	0,023346	0,388	201807_at	Vacuolar protein sorting 26 homolog A (S. pombe)	*VPS26A*
455	4,29	3,68	1,17	0,002434	0,288	240485_at	Tetratricopeptide repeat domain 8	*TTC8*
4996	261	224	1,16	0,038359	0,420	202985_s_at	BCL2-associated athanogene 5	*BAG5*
4030	18,6	15,9	1,16	0,029511	0,400	231548_at	Forkhead box O3	*FOXO3*
5675	5,36	4,61	1,16	0,045287	0,436	205092_x_at	Zinc finger and BTB domain containing 1	*ZBTB1*
4167	46,6	40,0	1,16	0,030793	0,404	222649_at	Exportin 4	*XPO4*
4816	33,8	29,0	1,16	0,036601	0,416	220938_s_at	Glucocorticoid modulatory element binding protein 1	*GMEB1*
2424	2177	1871	1,16	0,016211	0,365	214938_x_at	High-mobility group box 1	*HMGB1*
1281	4,74	4,07	1,16	0,007629	0,325	1562775_at	Nudix (nucleoside diphosphate linked moiety X)-type motif 12	*NUDT12*
3798	5,25	4,51	1,16	0,027669	0,398	1557553_at	Protein phosphatase 1, regulatory (inhibitor) subunit 12B	*PPP1R12B*
4344	5,23	4,49	1,16	0,032339	0,407	239461_at	Polypeptide N-acetylgalactosaminyltransferase-like protein 2	*GALNTL2*
3188	27,0	23,3	1,16	0,022597	0,387	209126_x_at	Keratin 6B	*KRT6B*
4273	11,7	10,0	1,16	0,031762	0,406	228132_at	Actin binding LIM protein family, member 2	*ABLIM2*
3148	4,94	4,25	1,16	0,022347	0,387	202035_s_at	Secreted frizzled-related protein 1	*SFRP1*
1777	16,2	14,0	1,16	0,011306	0,348	213863_s_at	Ornithine decarboxylase antizyme 3	*OAZ3*
587	6,19	5,33	1,16	0,003244	0,301	1565628_at	Leucine-rich repeat kinase 1	*LRRK1*
2868	52,4	45,1	1,16	0,020138	0,383	221192_x_at	Major facilitator superfamily domain containing 11	*MFSD11*
3079	7,67	6,60	1,16	0,021704	0,385	230392_at	cDNA FLJ31096 fis, clone IMR321000207	-
5933	122	105	1,16	0,048100	0,443	221564_at	Protein arginine methyltransferase 2	*PRMT2*
5695	7,34	6,33	1,16	0,045566	0,437	209800_at	Keratin 16	*KRT16*
1685	53,7	46,2	1,16	0,010651	0,345	47530_at	Chromosome 9 open reading frame 156	*C9orf156*
3477	7,37	6,35	1,16	0,024971	0,392	1563947_a_a	ELKS/RAB6-interacting/CAST family member 1	*ERC1*
4866	30,7	26,4	1,16	0,037112	0,417	232867_at	Serine palmitoyltransferase, long chain base subunit 2	*SPTLC2*
3866	190	164	1,16	0,028243	0,399	212245_at	Multiple coagulation factor deficiency 2	*MCFD2*
2415	15,6	13,5	1,16	0,016131	0,365	205203_at	Phospholipase D1, phosphatidylcholine-specific	*PLD1*
5422	1707	1473	1,16	0,042629	0,430	200680_x_at	High-mobility group box 1	*HMGB1*
2393	8,96	7,72	1,16	0,015975	0,365	241701_at	Rho GTPase activating protein 21	*ARHGAP21*
3169	36,3	31,3	1,16	0,022488	0,387	214253_s_at	Dystrobrevin, beta	*DTNB*
3719	6,79	5,87	1,16	0,026999	0,397	237173_at	Similar to phosphodiesterase 4D interacting protein isoform 1	*LOC647500*

Index	Mutated*	Normal*	FC	*P* value	FDR**	Probe set	Description	Gene symbol
4279	3,90	3,37	1,16	0,031801	0,406	232980_at	LMBR1 domain containing 1	*LMBRD1*
1644	9,14	7,89	1,16	0,010357	0,344	239769_at	Cadherin 11, type 2, OB-cadherin (osteoblast)	*CDH11*
5531	31,1	26,9	1,16	0,043887	0,434	227823_at	Retrotransposon gag domain containing 4	*RGAG4*
3475	6,13	5,30	1,16	0,024960	0,392	1565887_at	Transient receptor potential cation channel, subfamily M, member 7	*TRPM7*
5425	21,0	18,1	1,16	0,042688	0,430	243656_at	Hypothetical protein LOC642852	*LOC642852*
4555	6,00	5,18	1,16	0,034440	0,412	231061_at	La ribonucleoprotein domain family, member 7	*LARP7*
2134	5,38	4,65	1,16	0,014029	0,359	221697_at	Microtubule-associated protein 1 light chain 3 gamma	*MAP1LC3C*
1520	12,8	11,0	1,16	0,009394	0,337	1555752_at	Saitohin	*STH*
5650	1931	1671	1,16	0,045067	0,436	208646_at	Ribosomal protein S14	*RPS14*
2178	8,96	7,75	1,16	0,014409	0,361	241627_x_at	Hypothetical protein FLJ10357	*FLJ10357*
1521	59,8	51,7	1,16	0,009399	0,337	202640_s_at	RAN binding protein 3	*RANBP3*
2802	5,17	4,48	1,15	0,019474	0,380	1553300_a_a	Diacylglycerol kinase, eta	*DGKH*
5623	3,99	3,46	1,15	0,044911	0,436	204748_at	Prostaglandin-endoperoxide synthase 2	*PTGS2*
6096	80,8	70,0	1,15	0,049756	0,446	203291_at	CCR4-NOT transcription complex, subunit 4	*CNOT4*
955	10,6	9,16	1,15	0,005504	0,315	210535_at	B9 protein	*EPPB9*
5015	19,2	16,6	1,15	0,038580	0,420	1570078_a_a	Dedicator of cytokinesis 5	*DOCK5*
1323	3,68	3,19	1,15	0,007915	0,327	215339_at	Natural killer-tumor recognition sequence	*NKTR*
409	6,64	5,76	1,15	0,002148	0,286	1558874_a_a	cDNA clone YP97H11	-
3103	4,08	3,54	1,15	0,021909	0,386	216997_x_at	Transducin-like enhancer of split 4 (E(sp1) homolog, Drosophila)	*TLE4*
618	8,68	7,52	1,15	0,003500	0,308	232878_at	Hypothetical protein LOC644192	*LOC644192*
1657	4330	3757	1,15	0,010453	0,345	213828_x_at	H3 histone, family 3A	*H3F3A*
5731	22,9	19,9	1,15	0,045976	0,438	241757_x_at	Dynein, cytoplasmic 2, light intermediate chain 1	*DYNC2LI1*
4639	6,10	5,29	1,15	0,034982	0,412	241776_at	Transcribed locus	-
3907	5,79	5,02	1,15	0,028526	0,399	244755_at	Xenotropic and polytropic retrovirus receptor	*XPR1*
3617	26,0	22,6	1,15	0,026116	0,395	220215_at	Zinc finger protein 669	*ZNF669*
1878	6,59	5,72	1,15	0,012155	0,354	235492_at	Ring finger protein 217	*RNF217*
304	9,32	8,10	1,15	0,001467	0,263	1563283_at	cDNA clone IMAGE:4828909	-
1643	5,61	4,88	1,15	0,010350	0,344	1553211_at	Ankyrin-repeat and fibronectin type III domain containing 1	*ANKFN1*
5568	5,38	4,68	1,15	0,044332	0,435	237622_at	Transcribed locus	-
5870	4,16	3,62	1,15	0,047434	0,442	235575_at	Succinate dehydrogenase complex, subunit A, flavoprotein	*SDHALP2*
1571	8,63	7,51	1,15	0,009762	0,340	204680_s_at	Rap guanine nucleotide exchange factor (GEF) 5	*RAPGEF5*
3713	209	182	1,15	0,026924	0,396	219097_x_at	Chromosome 19 open reading frame 42	*C19orf42*
1267	4345	3782	1,15	0,007544	0,325	208755_x_at	H3 histone, family 3A	*H3F3A*
4437	5,33	4,64	1,15	0,033242	0,410	242916_at	Centrosomal protein 110kDa	*CEP110*
4868	6,57	5,73	1,15	0,037156	0,417	230451_at	DISC1 disrupted in schizophrenia 1	*DISC1*
399	6,36	5,54	1,15	0,002090	0,285	236306_at	KIAA2026 protein	*KIAA2026*
5557	14,7	12,8	1,15	0,044236	0,435	232566_at	Nucleolar protein family 6 (RNA-associated)	*NOL6*
3042	22,5	19,6	1,15	0,021428	0,385	240494_at	CTD small phosphatase-like protein	*CTDSPL*
1523	19,6	17,1	1,15	0,009416	0,337	233065_at	Ring finger protein 207	*RNF207*
4301	8,35	7,29	1,15	0,032011	0,407	238608_at	Laminin, beta 1	*LAMB1*
4455	5,15	4,50	1,15	0,033465	0,411	206035_at	v-rel reticuloendotheliosis viral oncogene homolog (avian)	*REL*
4727	6,16	5,38	1,15	0,035769	0,414	236678_at	Jagged 1 (Alagille syndrome)	*JAG1*
4000	12,4	10,8	1,15	0,029274	0,400	227225_at	Hypothetical gene supported by AK125693	*LOC439985*
5801	18,9	16,5	1,14	0,046784	0,441	219617_at	Chromosome 2 open reading frame 34	*C2orf34*
4365	9,04	7,90	1,14	0,032466	0,407	209359_x_at	Runt-related transcription factor 1 (aml1 oncogene)	*RUNX1*
6095	5,42	4,73	1,14	0,049745	0,446	235540_at	Gonadotropin-releasing hormone 1	*GNRH1*
4077	17,1	15,0	1,14	0,029919	0,401	240134_at	WWC family member 3	*WWC3*
5424	5,20	4,54	1,14	0,042684	0,430	220842_at	Abelson helper integration site 1	*AHI1*
5730	1453	1271	1,14	0,045972	0,438	222229_x_at	Similar to 60S ribosomal protein L26	*LOC392501*
1566	5,21	4,56	1,14	0,009729	0,340	1556113_at	cDNA DKFZp451A211 protein	*DKFZp451A211*
3788	8,76	7,66	1,14	0,027581	0,398	215415_s_at	Lysosomal trafficking regulator	*LYST*
5685	157	138	1,14	0,045379	0,436	222207_x_at	Williams Beuren syndrome chromosome region 19 pseudogene	*LOC389517*
5041	19,7	17,2	1,14	0,038851	0,420	236629_at	Chromosome 1 open reading frame 69	*C1orf69*
2188	5,56	4,86	1,14	0,014490	0,361	244340_x_at	-	-
3242	4,96	4,34	1,14	0,022993	0,388	219685_at	Transmembrane protein 35	*TMEM35*
3058	6,77	5,93	1,14	0,021531	0,385	228656_at	Prospero homeobox 1	*PROX1*
330	3,65	3,20	1,14	0,001639	0,272	1558920_at	Solute carrier family 8 (sodium/calcium exchanger), member 1	*SCL8A1*

Index	Mutated*	Normal*	FC	*P* value	FDR**	Probe set	Description	Gene symbol
5553	16,5	14,5	1,14	0,044202	0,435	229392_s_at	Phosphoinositide-3-kinase, regulatory subunit 2 (p85 beta)	*PIK3R2*
5946	44,1	38,6	1,14	0,048181	0,443	201213_at	Protein phosphatase 1, regulatory (inhibitor) subunit 7	*PPP1R7*
6097	3,64	3,19	1,14	0,049760	0,446	220474_at	Solute carrier family 25 member 21	*SLC25A21*
2656	6,25	5,47	1,14	0,018114	0,373	204110_at	Histamine N-methyltransferase	*HNMT*
5060	9,64	8,45	1,14	0,038956	0,420	214600_at	TEA domain family member 1 (SV40 transcriptional enhancer factor)	*TEAD1*
4507	4,66	4,09	1,14	0,033953	0,412	234020_x_at	Copper metabolism (Murr1) domain containing 1	*COMMD1*
3518	9,93	8,71	1,14	0,025254	0,392	239306_at	Chromosome 14 open reading frame 93	*C14orf93*
4404	318	279	1,14	0,032875	0,408	223060_at	Chromosome 14 open reading frame 119	*C14orf119*
2100	4,12	3,61	1,14	0,013760	0,358	237963_x_at	KIAA1529 protein	*KIAA1529*
898	5,51	4,83	1,14	0,005203	0,313	232858_at	Sex comb on midleg homolog 1 (Drosophila)	*SCMH1*
2973	5,53	4,85	1,14	0,020879	0,384	1560332_at	Carbohydrate (chondroitin) sulfotransferase 11	*CHST11*
3797	3843	3374	1,14	0,027660	0,398	200012_x_at	Ribosomal protein L21	*RPL21*
3156	7,97	7,00	1,14	0,022400	0,387	205783_at	Kallikrein-related peptidase 13	*KLK13*
1298	4,16	3,66	1,14	0,007724	0,325	222172_at	Neuronal PAS domain protein 3	*NPAS3*
1818	4,02	3,53	1,14	0,011714	0,351	209125_at	Keratin 6A	*KRT6A*
5661	8,37	7,36	1,14	0,045180	0,436	205520_at	Striatin, calmodulin binding protein	*STRN*
5006	27,6	24,3	1,14	0,038454	0,420	1556066_at	Jumonji domain containing 3	*JMJD3*
2039	24,9	21,9	1,14	0,013274	0,355	1556014_at	Mesoderm posterior 2 homolog (mouse)	*MESP2*
6072	197	173	1,14	0,049585	0,446	203043_at	Zinc finger, BED-type containing 1	*ZBED1*
4770	10,3	9,02	1,14	0,036157	0,414	233117_at	Microtubule-associated protein tau	*MAPT*
3927	236	207	1,14	0,028727	0,399	226024_at	Copper metabolism (Murr1) domain containing 1	*COMMD1*
6100	14,6	12,8	1,14	0,049799	0,446	211993_at	WNK lysine deficient protein kinase 1	*WNK1*
1065	4,65	4,09	1,14	0,006246	0,319	239980_at	Chromosome 22 open reading frame 28	*C22orf28*
4509	4,84	4,27	1,14	0,033981	0,412	220986_s_at	Tigger transposable element derived 6	*TIGD6*
5948	4,33	3,81	1,14	0,048197	0,443	236277_at	p21 protein (Cdc42/Rac)-activated kinase 3	*PAK3*
1186	7,05	6,21	1,14	0,006976	0,322	207552_at	ATP synthase proteolipid P2	*ATP5G2*
2182	11,6	10,2	1,13	0,014441	0,361	1558484_s_a	Leucine rich repeat containing 27	*LRRC27*
5647	15,2	13,4	1,13	0,045057	0,436	214492_at	Sarcoglycan, delta (35kDa dystrophin-associated glycoprotein)	*SGCD*
4733	3,55	3,13	1,13	0,035831	0,414	237825_x_at	Transcribed locus	-
4379	5,24	4,62	1,13	0,032568	0,407	1559394_a_a	Receptor tyrosine kinase-like orphan receptor 1	*ROR1*
4943	5,25	4,63	1,13	0,037929	0,419	242636_at	Prolylcarboxypeptidase (angiotensinase C)	*PRCP*
2667	4,33	3,82	1,13	0,018241	0,374	232538_at	FLJ23573 fis, clone LNG12520	-
648	4,60	4,06	1,13	0,003681	0,308	215635_at	Phosphodiesterase 8A	*PDE8A*
5602	11,8	10,4	1,13	0,044708	0,436	1556229_at	Carboxylate kinase-like	*CARLK*
5585	6,75	5,97	1,13	0,044528	0,436	230067_at	Family with sequence similarity 124A	*FAM124A*
5103	5,21	4,61	1,13	0,039244	0,420	241041_at	Solute carrier family 8 (sodium/calcium exchanger), member 1	*SLC8A1*
2533	24,5	21,7	1,13	0,017131	0,369	226836_at	SFT2 domain containing 1	*SFT2D1*
3962	311	275	1,13	0,028950	0,399	1555948_s_a	Family with sequence similarity 120A	*FAM120A*
4092	43,6	38,6	1,13	0,030080	0,402	219128_at	Chromosome 2 open reading frame 42	*C2orf42*
3619	5,35	4,74	1,13	0,026136	0,395	221625_at	cDNA FLJ46609 fis, clone TKIDN2010200	-
398	3,84	3,41	1,13	0,002087	0,285	1554413_s_a	RUN domain containing 2B	*RUNDC2B*
1773	5868	5198	1,13	0,011296	0,348	206559_x_at	Eukaryotic translation elongation factor 1 alpha 1	*EEF1A1*
4078	11,6	10,2	1,13	0,029924	0,401	209327_s_at	NOP16 nucleolar protein homolog (yeast)	*NOP16*
4722	42,3	37,5	1,13	0,035711	0,413	217142_at	Eukaryotic translation elongation factor 1 alpha 1	*EEF1A1*
5143	13,0	11,5	1,13	0,039594	0,421	231658_x_at	Ribosomal protein L36	*RPL36*
4222	19,9	17,6	1,13	0,031290	0,405	228663_x_at	FLT3-interacting zinc finger 1	*FIZ1*
3825	5,00	4,44	1,13	0,027925	0,399	236354_at	Zinc finger, MYND domain containing 17	*ZMYND17*
4776	8,93	7,92	1,13	0,036212	0,414	229859_at	Intercellular adhesion molecule 3	*ICAM3*
6052	6,45	5,72	1,13	0,049331	0,446	1569608_x_a	Similar to ankyrin repeat domain 20A	*LOC643187*
2140	7,37	6,55	1,13	0,014073	0,360	214401_at	Paired box 1	*PAX1*
1360	4,63	4,11	1,13	0,008199	0,328	1560230_at	Supervillin	*SVIL*
5465	228	203	1,13	0,043161	0,432	202469_s_at	Cleavage and polyadenylation specific factor 6, 68kDa	*CPSF6*
4918	95,9	85,2	1,13	0,037660	0,419	200767_s_at	Family with sequence similarity 120A	*FAM120A*
1852	3,70	3,29	1,13	0,011938	0,352	236999_at	Peptidylprolyl isomerase domain and WD repeat containing 1	*PPWD1*
3405	9,70	8,62	1,13	0,024293	0,390	1553169_at	Chromosome 20 open reading frame 75	*C20orf75*
5786	31,4	27,9	1,13	0,046596	0,440	220411_x_at	Podocan-like 1	*PODNL1*
1192	6,06	5,38	1,13	0,007032	0,322	214445_at	Elongation factor, RNA polymerase II, 2	*ELL2*

Index	Mutated*	Normal*	FC	*P* value	FDR**	Probe set	Description	Gene symbol
5894	5,66	5,03	1,13	0,047683	0,442	237671_at	RAD51-like 1 (S. cerevisiae)	*RAD51L1*
5038	19,4	17,2	1,13	0,038809	0,420	214525_x_at	MutL homolog 3 (E. coli)	*MLH3*
3155	6,96	6,19	1,13	0,022379	0,387	229188_s_at	Zinc and ring finger 2	*ZNRF2*
2882	7,89	7,02	1,12	0,020225	0,383	206012_at	Left-right determination factor 2	*LEFTY2*
1316	4303	3828	1,12	0,007836	0,325	213347_x_at	Ribosomal protein S4, X-linked	*RPS4X*
2363	575	511	1,12	0,015774	0,364	207988_s_at	Actin related protein 2/3 complex, subunit 2, 34kDa	*ARPC2*
4226	5340	4753	1,12	0,031322	0,405	216520_s_at	Tumor protein, translationally-controlled 1	*TPT1*
215	3,91	3,48	1,12	0,000963	0,244	214034_at	Endoplasmic reticulum aminopeptidase 1	*ARTS-1*
3345	7,84	6,99	1,12	0,023759	0,388	236688_at	FERM and PDZ domain containing 3	*FRMPD3*
5938	13,8	12,3	1,12	0,048146	0,443	1563529_at	Hydrocephalus inducing homolog 2 (mouse)	*HYDIN2*
4184	6,51	5,80	1,12	0,030944	0,404	241494_at	Serine incorporator 5	*SERINC5*
2583	3,86	3,44	1,12	0,017527	0,371	241913_at	Kelch-like 8 (Drosophila)	*KLHL8*
534	5,12	4,57	1,12	0,002857	0,292	238786_at	Transcribed locus	-
5490	6,77	6,04	1,12	0,043450	0,433	236779_at	Mitochondrial ribosomal protein S5	*MRPS5*
99	3,49	3,12	1,12	0,000386	0,213	208054_at	Hect domain and RLD 4	*HERC4*
6060	6,42	5,73	1,12	0,049414	0,446	234697_x_at	Chromosome 3 open reading frame 31	*C3orf31*
3228	8,41	7,52	1,12	0,022897	0,388	236869_at	Transcribed locus	-
4300	12,7	11,3	1,12	0,032007	0,407	1553346_a_a	Trinucleotide repeat containing 6A	*TNRC6A*
1949	4,07	3,64	1,12	0,012725	0,355	219195_at	PPAR-gamma coactivator 1-alpha	*PPARGC1A*
772	3,70	3,31	1,12	0,004423	0,311	1563389_at	cDNA clone IMAGE:4836780	-
839	4,06	3,63	1,12	0,004807	0,313	1557275_a_a	cDNA FLJ20031 fis, clone ADSU02180	-
3879	5,03	4,50	1,12	0,028335	0,399	244206_at	AN1, ubiquitin-like, homolog (Xenopus laevis)	*ANUBL1*
1774	11,6	10,4	1,12	0,011299	0,348	219674_s_at	High density lipoprotein binding protein (vigilin)	*HDLBP*
4871	6,08	5,44	1,12	0,037185	0,417	207653_at	Forkhead box D2	*FOXD2*
3876	4,81	4,31	1,12	0,028314	0,399	211086_x_at	NIMA (never in mitosis gene a)-related kinase 1	*NEK1*
5238	4,09	3,67	1,12	0,040574	0,423	217057_s_at	GNAS complex locus	*GNAS*
1982	3,45	3,09	1,12	0,012935	0,355	1566787_at	Similar to poly (ADP-ribose) glycohydrolase	*PARG*
4711	10,2	9,13	1,12	0,035646	0,413	177_at	Phospholipase D1, phosphatidylcholine-specific	*PLD1*
2696	7138	6404	1,11	0,018486	0,375	204892_x_at	Eukaryotic translation elongation factor 1 alpha 1	*EEF1A1*
3253	4,23	3,80	1,11	0,023081	0,388	243008_at	RAS homolog enriched in brain	*RHEB*
2949	14,9	13,4	1,11	0,020711	0,384	219626_at	MAP7 domain containing 3	*MAP7D3*
2859	4,55	4,09	1,11	0,020072	0,383	236944_at	Transcribed locus	-
481	4,28	3,85	1,11	0,002572	0,289	1556800_a_a	Glutamate receptor, metabotropic 8	*GRM8*
1684	3,91	3,51	1,11	0,010646	0,345	1558964_at	FAT tumor suppressor homolog 3 (Drosophila)	*FAT3*
5087	5,80	5,21	1,11	0,039159	0,420	229311_at	G kinase anchoring protein 1	*GKAP1*
821	3,96	3,56	1,11	0,004689	0,312	1553630_at	Chromosome 10 open reading frame 107	*C10orf107*
2205	5,54	4,99	1,11	0,014575	0,361	222337_at	Oxysterol binding protein-like 9	*OSBPL9*
257	3,17	2,86	1,11	0,001202	0,256	215647_at	RNA binding motif protein 26	*RBM26*
2774	6,94	6,25	1,11	0,019258	0,380	234079_at	Rho-guanine nucleotide exchange factor	*RGNEF*
1908	3,78	3,40	1,11	0,012416	0,355	230081_at	Phosphatidylinositol-specific phospholipase C, X domain containing 3	*PLCXD3*
4815	4,00	3,60	1,11	0,036593	0,416	220719_at	Hypothetical protein FLJ13769	*FLJ13769*
1363	3,57	3,22	1,11	0,008207	0,328	1567527_at	Ubiquitin B (UBB) mRNA, 3' UTR and genetic suppressor element	-
354	6207	5593	1,11	0,001808	0,279	212869_x_at	Tumor protein, translationally-controlled 1	*TPT1*
5376	10,1	9,07	1,11	0,042168	0,429	224458_at	Chromosome 9 open reading frame 125	*C9orf125*
3544	415	374	1,11	0,025498	0,393	200829_x_at	Zinc finger protein 207	*ZNF207*
3742	10,1	9,08	1,11	0,027228	0,397	233862_at	cDNA clone IMAGE:4698949	-
5626	24,0	21,6	1,11	0,044919	0,436	243860_at	Ataxin 1	*ATXN1*
2180	5,19	4,68	1,11	0,014410	0,361	237628_at	Tryptophanyl tRNA synthetase 2, mitochondrial	*WARS2*
580	4,96	4,48	1,11	0,003183	0,300	233240_at	cDNA FLJ10255 fis, clone HEMBB1000852	-
5846	4,32	3,90	1,11	0,047265	0,442	238279_x_at	Collagen, type IV, alpha 3 (Goodpasture antigen) binding protein	*COL4A3BP*
6091	5114	4616	1,11	0,049719	0,446	224594_x_at	Actin, beta	*ACTB*
581	3,30	2,98	1,11	0,003186	0,300	242642_at	NudE nuclear distribution gene E homolog (A. nidulans)-like 1	*NDEL1*
5747	5,56	5,02	1,11	0,046116	0,439	217201_at	RAS protein activator like 2	*RASAL2*
1960	11,4	10,3	1,11	0,012812	0,355	237331_s_at	Hypothetical protein LOC100128977	*LOC100128977*
4867	7,21	6,51	1,11	0,037151	0,417	239701_at	Ectonucleoside triphosphate diphosphohydrolase 1	*ENTPD1*
3247	3,59	3,24	1,11	0,023061	0,388	232945_at	pseudouridylate synthase 10	*PUS10*
4528	5,53	5,00	1,11	0,034179	0,412	241711_at	Transducin (beta)-like 1 X-linked receptor 1	*TBL1XR1*

Index	Mutated*	Normal*	FC	*P* value	FDR**	Probe set	Description	Gene symbol
4439	5,07	4,58	1,11	0,033326	0,410	1559672_a_a	Chromosome 9 open reading frame 93	*C9orf93*
1143	3,42	3,09	1,11	0,006695	0,320	1558107_at	FSHD region gene 1 family, member B	*FRG1B*
3425	47,2	42,7	1,11	0,024487	0,391	209547_s_at	Splicing factor 4	*SF4*
87	3,61	3,27	1,10	0,000328	0,201	234588_at	-	-
854	5215	4721	1,10	0,004916	0,313	211943_x_at	Tumor protein, translationally-controlled 1	*TPT1*
201	5945	5384	1,10	0,000925	0,244	212284_x_at	Tumor protein, translationally-controlled 1	*TPT1*
3171	3,66	3,31	1,10	0,022504	0,387	231925_at	CDNA FLJ23006 fis, clone LNG00414	-
5925	4,65	4,21	1,10	0,048007	0,443	237322_at	Myocardial infarction associated transcript (non-protein coding)	*MIAT*
1910	4,54	4,12	1,10	0,012436	0,355	231610_at	Tau tubulin kinase 2	*TTBK2*
3584	5,81	5,26	1,10	0,025871	0,394	1566609_at	cDNA FLJ25907 fis, clone CBR04607	-
2423	3,70	3,36	1,10	0,016202	0,365	220729_at	-	-
5592	4,13	3,75	1,10	0,044609	0,436	234243_at	Brix domain containing 5	*BXDC5*
4345	10,6	9,63	1,10	0,032356	0,407	201205_at	Ribosome binding protein 1 homolog 180kDa	*RRBP1*
1435	4,22	3,83	1,10	0,008700	0,331	241228_at	Transcribed locus	-
6093	7,75	7,04	1,10	0,049730	0,446	1562946_at	cDNA clone IMAGE:3503939	-
5299	4,02	3,65	1,10	0,041287	0,426	1568777_at	Echinoderm microtubule associated protein like 5	*EML5*
849	3,93	3,57	1,10	0,004889	0,313	1556925_at	Structural maintenance of chromosomes 3	*SMC3*
5446	3,82	3,47	1,10	0,042957	0,431	1560264_at	Mastermind-like 3 (Drosophila)	*MAML3*
4271	4,08	3,71	1,10	0,031749	0,406	211022_s_at	Alpha thalassemia/mental retardation syndrome X-linked	*ATRX*
3816	4684	4261	1,10	0,027829	0,399	200781_s_at	Ribosomal protein S15a	*RPS15A*
1723	4,47	4,07	1,10	0,011039	0,348	1561106_at	Chromosome 6 open reading frame 217	*C6orf217*
4542	5,12	4,66	1,10	0,034324	0,412	1555151_s_a	L-threonine dehydrogenase	*TDH*
1939	6,27	5,71	1,10	0,012633	0,355	206248_at	Protein kinase C, epsilon	*PRKCE*
2707	14,3	13,0	1,10	0,018561	0,375	227879_at	AlkB, alkylation repair homolog 7 (E. coli)	*ALKBH7*
5211	3,34	3,05	1,10	0,040226	0,422	233945_at	UDP-glucose ceramide glucosyltransferase-like 2	*UGCGL2*
2890	5,84	5,32	1,10	0,020289	0,383	1553506_at	Carboxypeptidase O	*CPO*
2817	3,84	3,50	1,10	0,019664	0,381	234805_at	ATPase family, AAA domain containing 1	*ATAD1*
3316	7,61	6,95	1,10	0,023566	0,388	223953_s_at	Zinc finger and BTB domain containing 37	*ZBTB37*
2955	5,01	4,57	1,10	0,020746	0,384	239690_at	Transcribed locus	-
2734	5,23	4,77	1,10	0,018849	0,377	206962_x_at	Zinc finger protein 638	*ZNF638*
4686	4622	4218	1,10	0,035378	0,413	213583_x_at	Eukaryotic translation elongation factor 1 alpha 1	*EEF1A1*
4205	4,13	3,77	1,10	0,031091	0,404	1566106_at	Mitofusin 2	*MFN2*
5887	16,5	15,0	1,10	0,047624	0,442	239381_at	Kallikrein-related peptidase 7	*KLK7*
1232	5,30	4,84	1,10	0,007329	0,324	228926_s_at	ATP-dependent helicase SMARCA2	*SMARCA2*
5025	7,92	7,24	1,09	0,038639	0,420	242624_at	Actin binding LIM protein family, member 2	*ABLIM2*
568	2167	1981	1,09	0,003107	0,299	214327_x_at	Tumor protein, translationally-controlled 1	*TPT1*
1472	3,23	2,96	1,09	0,008998	0,334	221057_at	Spermatogenesis associated 1	*SPATA1*
685	3,27	2,99	1,09	0,003927	0,308	239712_at	Chromosome 9 open reading frame 93	*C9orf93*
2083	4,30	3,93	1,09	0,013595	0,356	1558518_at	TGF-beta activated kinase 1/MAP3K7 binding protein 3	*MAP3K7IP3*
3882	5272	4827	1,09	0,028346	0,399	213614_x_at	Eukaryotic translation elongation factor 1 alpha 1	*EEF1A1*
5225	3,83	3,52	1,09	0,040414	0,423	244275_at	Transcribed locus	-
4677	4,21	3,86	1,09	0,035310	0,413	1559695_a_a	DLGAP1, Discs, large (Drosophila) homolog-associated protein 1	*DLGAP1*
5864	5,00	4,59	1,09	0,047415	0,442	1561631_at	Spermatogenesis associated, serine-rich 2-like	*SPATS2L*
4079	3,72	3,41	1,09	0,029936	0,401	240990_at	RAB5A, member RAS oncogene family	*RAB5A*
2236	3,57	3,28	1,09	0,014852	0,363	243316_x_at	Vacuolar protein sorting 26 homolog A (S. pombe)	*VPS26A*
2071	4,21	3,86	1,09	0,013541	0,356	229171_at	CENPB DNA-binding domains containing 1	*CENPBD1*
1346	4,46	4,09	1,09	0,008069	0,328	233227_at	KIAA1109 protein	*KIAA1109*
5380	10,6	9,73	1,09	0,042186	0,429	204250_s_at	Centrosomal protein 164kDa	*CEP164*
2165	3,79	3,49	1,09	0,014334	0,361	244146_at	DTW domain containing 1	*DTWD1*
5990	3,50	3,21	1,09	0,048718	0,444	216595_at	DnaJ (Hsp40) homolog, subfamily C, member 22	*DNAJC22*
6026	3,63	3,34	1,09	0,049024	0,445	231930_at	ELMO/CED-12 domain containing 1	*ELMOD1*
5749	3,73	3,43	1,09	0,046135	0,439	1559369_at	Chromosome 5 open reading frame 44	*C5orf44*
4484	4,35	4,01	1,09	0,033740	0,411	1556323_at	CUG triplet repeat, RNA binding protein 2	*CUGBP2*
2486	5,03	4,63	1,09	0,016751	0,367	216536_at	Olfactory receptor, family 7, subfamily E, member 19 pseudogene	*OR7E19P*
3003	9,32	8,59	1,09	0,021133	0,385	239085_at	Jun dimerization protein 2	*JDP2*
6124	3701	3412	1,08	0,049993	0,446	229563_s_at	Ribosomal protein L10a	*RPL10A*
713	3,01	2,77	1,08	0,004092	0,308	1558667_at	Spermatogenesis associated 22	*SPATA22*

Index	Mutated*	Normal*	FC	*P* value	FDR**	Probe set	Description	Gene symbol
3638	3,86	3,56	1,08	0,026309	0,395	235380_at	Arachidonate 5-lipoxygenase	*ALOX5*
5347	3,59	3,31	1,08	0,041919	0,429	1555038_at	Erythrocyte membrane protein band 4.1 like 4A	*EPB41L4A*
396	3,17	2,93	1,08	0,002086	0,285	238203_at	-	-
3355	4,68	4,32	1,08	0,023825	0,388	232953_at	Chromosome 20 open reading frame 69	*C20orf69*
4708	3,94	3,65	1,08	0,035603	0,413	217571_at	-	-
3810	4,82	4,46	1,08	0,027763	0,398	211347_at	CDC14 cell division cycle 14 homolog B (S. cerevisiae)	*CDC14B*
3067	6,32	5,85	1,08	0,021654	0,385	1563098_at	EEF1A2 binding protein	*EEF1A2BP1*
378	2,82	2,61	1,08	0,001972	0,284	1554072_s_a	Coiled-coil domain containing 67	*CCDC67*
212	3,11	2,88	1,08	0,000958	0,244	240768_x_at	UBX domain protein 4	*UBXD2*
2515	4,92	4,56	1,08	0,016944	0,368	1566040_at	cDNA DKFZp667F083	-
5473	3,15	2,92	1,08	0,043268	0,432	1568916_at	Spire homolog 2 (Drosophila)	*SPIRE2*
3989	4,08	3,78	1,08	0,029178	0,400	210500_at	Ubiquitin associated protein 2-like	*UBAP2L*
802	3,24	3,01	1,08	0,004575	0,311	231391_at	Cortexin 3	*CTXN3*
5631	3,83	3,55	1,08	0,044947	0,436	239770_at	Family with sequence similarity 62 (C2 domain containing), member C	*FAM62C*
4980	3,30	3,06	1,08	0,038210	0,419	217175_at	UDP glucuronosyltransferase 2 family, polypeptide B15	*UGT2B15*
4819	5425	5039	1,08	0,036637	0,416	200933_x_at	Ribosomal protein S4, X-linked	*RPS4X*
5429	10,8	10,0	1,08	0,042726	0,430	241203_at	-	-
5654	4,69	4,36	1,07	0,045099	0,436	232179_at	Hypothetical protein LOC158863	*LOC158863*
1508	3,62	3,37	1,07	0,009285	0,337	233393_at	Methionine sulfoxide reductase A	*MSRA*
4517	4,06	3,78	1,07	0,034044	0,412	1563044_at	cDNA clone IMAGE:5164837	-
5648	3,69	3,43	1,07	0,045065	0,436	240034_at	Transcribed locus	-
6106	6,57	6,13	1,07	0,049851	0,446	210760_x_at	Thyroid hormone receptor interactor 11	*TRIP11*
3309	3,30	3,07	1,07	0,023531	0,388	222246_at	Syntaxin binding protein 3	*STXBP3*
5679	3,00	2,79	1,07	0,045343	0,436	1560775_at	cDNA FLJ25185 fis, clone CBR09429	-
337	2,84	2,64	1,07	0,001719	0,277	234432_at	Metastasis related protein (MB2)	-
1597	5094	4754	1,07	0,009955	0,341	207783_x_at	HECT, UBA and WWE domain containing 1	*HUWE1*
4933	4,96	4,63	1,07	0,037824	0,419	242038_at	Leucine rich repeat containing 8 family, member B	*LRRC8B*
155	2,66	2,49	1,07	0,000705	0,244	1569810_at	cDNA clone IMAGE:5271710	-
5362	8,52	7,96	1,07	0,042063	0,429	215500_at	Similar to RIKEN cDNA 4933437K13	*LOC92017*
5656	3,11	2,91	1,07	0,045103	0,436	1561362_at	cDNA FLJ36285 fis, clone THYMU2003470	-
5953	5,29	4,94	1,07	0,048296	0,444	217004_s_at	MCF.2 cell line derived transforming sequence	*MCF2*
860	3,08	2,88	1,07	0,004964	0,313	1563053_at	Coiled-coil domain containing 13	*CCDC13*
6087	3,07	2,87	1,07	0,049696	0,446	243262_at	-	-
3714	5,01	4,69	1,07	0,026935	0,396	240641_at	Transcribed locus	-
1936	3,25	3,05	1,07	0,012615	0,355	1564273_at	Jelch domain containing 1	*KLHDC1*
5077	3,16	2,96	1,07	0,039053	0,420	237830_at	-	-
5312	3,51	3,29	1,07	0,041437	0,426	1557869_at	Mitochondrial ribosomal protein L55	*MRPL55*
1693	3,68	3,45	1,07	0,010710	0,346	1562139_a_a	Forkhead box P2	*FOXP2*
4619	3,79	3,55	1,07	0,034847	0,412	241264_at	WD repeat domain 7	*WDR7*
2347	3,58	3,35	1,07	0,015661	0,364	1566867_at	Ataxin 2-binding protein 1	*A2BP1*
4666	2,97	2,78	1,07	0,035242	0,413	244817_at	Inhibitor of growth family, member 1	*ING1*
3166	2,88	2,70	1,07	0,022463	0,387	216097_at	PHD finger protein 14	*PHF14*
1313	2,79	2,62	1,07	0,007827	0,325	1561331_at	Chromosome 1 open reading frame 99	*C1orf99*
5246	3,97	3,73	1,07	0,040612	0,423	1568826_at	Hypothetical protein LOC389372	*LOC389372*
3371	3,35	3,15	1,06	0,023978	0,389	219962_at	Angiotensin I converting enzyme (peptidyl-dipeptidase A) 2	*ACE2*
5812	3,75	3,53	1,06	0,046940	0,442	209638_x_at	Regulator of G-protein signaling 12	*RGS12*
3820	4,17	3,92	1,06	0,027890	0,399	234893_s_at	Dynein, axonemal, heavy chain 6	*DNHL1*
3401	3,27	3,07	1,06	0,024260	0,390	216619_at	GTP-binding protein 10 (putative)	*GTPBP10*
5610	3,45	3,24	1,06	0,044735	0,436	1570408_at	cDNA clone pp9953	-
4668	3,99	3,76	1,06	0,035244	0,413	1559109_a_a	Vacuolar protein sorting 53 homolog (S. cerevisiae)	*VPS53*
3775	3,80	3,57	1,06	0,027438	0,397	1568932_at	cDNA clone IMAGE:4830452	-
5932	3,34	3,15	1,06	0,048096	0,443	239905_at	YTH domain containing 1	*YTHDC1*
4193	6,73	6,35	1,06	0,030973	0,404	1561579_at	Hypothetical protein LOC728445	*LOC728445*
5734	3,04	2,87	1,06	0,045997	0,438	1569669_at	Forkhead box R2	*FOXR2*
1907	2,98	2,81	1,06	0,012409	0,355	244412_at	-	-
5261	3,34	3,15	1,06	0,040870	0,425	234765_at	Brain-specific angiogenesis inhibitor 3	*BAI3*
1187	3,01	2,84	1,06	0,006997	0,322	1565877_at	Nucleoporin 153kDa	*NUP153*

Index	Mutated*	Normal*	FC	*P* value	FDR**	Probe set	Description	Gene symbol
4934	3,95	3,73	1,06	0,037845	0,419	242904_x_at	Transmembrane protein 66	*TMEM66*
4136	3,39	3,20	1,06	0,030482	0,403	1570111_at	Chromosome 14 open reading frame 48	*C14orf48*
5875	3,32	3,13	1,06	0,047461	0,442	1555284_at	Amyotrophic lateral sclerosis 2 (juvenile)	*ALS2*
4570	3,01	2,84	1,06	0,034557	0,412	236220_at	Transcribed locus	-
3806	2,94	2,78	1,06	0,027714	0,398	1559035_a_a	Aryl hydrocarbon receptor	*ARH*
1854	3,00	2,84	1,06	0,011943	0,352	1562860_at	cDNA clone IMAGE:5744121	-
2229	3,08	2,91	1,06	0,014807	0,363	1569823_at	hypothetical protein LOC440346	*LOC440346*
5204	3,44	3,25	1,06	0,040145	0,422	216632_at	Neuron navigator 3	*NAV3*
3104	3,49	3,30	1,06	0,021916	0,386	1552851_at	Zinc finger and SCAN domain containing 4	*ZSCAN4*
4771	2,98	2,82	1,06	0,036158	0,414	1570103_at	Golgi autoantigen, golgin subfamily a, 6-like 9	*GOLGA6L9*
5784	3,18	3,01	1,06	0,046585	0,440	1566899_at	Succinate dehydrogenase flavoprotein subunit	*HSSUCCDH*
5345	3,32	3,14	1,06	0,041904	0,429	240992_at	Transcribed locus	-
3972	2,61	2,47	1,06	0,029040	0,400	1564631_at	EF-hand calcium binding domain 3	*EFCAB3*
3091	2,73	2,58	1,06	0,021795	0,385	241909_at	Tankyrase, TRF1-interacting ankyrin-related ADP-ribose polymerase 2	*TNKS2*
4524	3,05	2,89	1,06	0,034133	0,412	1561737_at	cDNA DKFZp667L064	-
2764	3,17	3,00	1,05	0,019197	0,379	1555839_a_a	Chromosome 3 open reading frame 79	*C3orf79*
3080	3,11	2,95	1,05	0,021707	0,385	1566632_at	cDNA clone IMAGE:3950788	-
6062	3,69	3,50	1,05	0,049435	0,446	232585_at	Tousled-like kinase 2	*TLK2*
5342	2,99	2,84	1,05	0,041860	0,428	207879_at	-	-
3209	4,04	3,84	1,05	0,022721	0,387	1560358_at	Phosphorylase kinase, alpha 2	*PHKA2*
2850	2,79	2,65	1,05	0,019957	0,383	1566202_at	Protein phosphatase 3 (formerly 2B), catalytic subunit, alpha isoform	*PPP3CA*
4803	3,02	2,88	1,05	0,036455	0,415	1560516_at	Transmembrane protein 26	*TMEM26*
4304	2,74	2,61	1,05	0,032035	0,407	242198_at	Transcribed locus	-
4123	2,83	2,70	1,05	0,030324	0,402	215112_x_at	MCF.2 cell line derived transforming sequence-like 2	*MCF2L2*
4221	2,81	2,69	1,05	0,031284	0,405	1556873_at	Mex-3 homolog C (C. elegans)	*MEX3C*
5562	2,68	2,57	1,04	0,044295	0,435	234779_at	-	-
4596	2,85	2,73	1,04	0,034768	0,412	1567878_at	Defensin, beta 114	*DEFB114*
4665	2,72	2,61	1,04	0,035228	0,413	1554507_at	N-acetylated alpha-linked acidic dipeptidase 2	*NAALAD2*
5991	2,70	2,59	1,04	0,048724	0,444	1563040_s_a	cDNA clone IMAGE:5170410	-
5707	2,71	2,61	1,04	0,045697	0,438	1566930_at	Transcription factor B2, mitochondrial	*TFB2M*

*** Geometric mean of intensities**

**** False Discovery Rate**
