# Supplementary material for: Gene Expression Profiling Reveals New Aspects of PIK3CA Mutation in ERalpha-Positive Breast Cancer: Major Implication of the Wnt Signaling Pathway
Source: PLoS One. 2010 Dec 30;5(12):e15647. doi: 10.1371/journal.pone.0015647 (PMC3012715; doi:10.1371/journal.pone.0015647)
Supplement: Table S3 — 3586 probes down-regulated in tumors with PIK3CA mutations (Mutated) compared to tumors without PIK3CA mutation (Normal) with a P value <0.05 identified by a parametric t test using BRB ArrayTools. These genes were ranked according to fold change (FC) calculated between expression intensities of tumors with PIK3CA mutations and those of tumors without PIK3CA mutation. The 28 probes with a FC≥2 are put in bold. (PDF) [file pone.0015647.s003.pdf]

Index	Mutated*	Normal*	FC	*P* value	FDR**	Probe set	Description	Gene symbol
**4530**	**17,7**	**71,0**	**-4,01**	**0,03420**	**0,412**	**233059_at**	**Potassium inwardly-rectifying channel, subfamily J, member 3**	***KCNJ3***
**138**	**32,2**	**114**	**-3,52**	**0,00063**	**0,244**	**219438_at**	**Na+/K+ transporting ATPase interacting 1**	***NKAIN1***
**3114**	**4,70**	**16,3**	**-3,47**	**0,02202**	**0,387**	**209988_s_at**	**Achaete-scute complex homolog 1 (Drosophila)**	***ASCL1***
**5630**	**8,01**	**23,3**	**-2,91**	**0,04494**	**0,436**	**207142_at**	**Potassium inwardly-rectifying channel, subfamily J, member 3**	***KCNJ3***
**4210**	**14,9**	**41,0**	**-2,76**	**0,03116**	**0,405**	**207141_s_at**	**Potassium inwardly-rectifying channel, subfamily J, member 3**	***KCNJ3***
**967**	**6,75**	**17,6**	**-2,60**	**0,00559**	**0,315**	**1552575_a_at**	**Chromosome 6 open reading frame 141**	***C6orf141***
**1303**	**11,7**	**30,1**	**-2,57**	**0,00775**	**0,325**	**204637_at**	**Glycoprotein hormones, alpha polypeptide**	***CGA***
**3604**	**16,1**	**40,3**	**-2,51**	**0,02600**	**0,394**	**206110_at**	**Histone cluster 1, H3h**	***HIST1H3H***
**482**	**62,0**	**154**	**-2,48**	**0,00257**	**0,289**	**213423_x_at**	**Tumor suppressor candidate 3**	***TUSC3***
**1833**	**15,0**	**36,6**	**-2,44**	**0,01181**	**0,351**	**219312_s_at**	**Zinc finger and BTB domain containing 10**	***ZBTB10***
**715**	**18,3**	**44,6**	**-2,43**	**0,00410**	**0,308**	**231729_s_at**	**Calcyphosine**	***CAPS***
**4909**	**4,37**	**10,5**	**-2,41**	**0,03755**	**0,418**	**209987_s_at**	**Achaete-scute complex homolog 1 (Drosophila)**	***ASCL1***
**2721**	**20,5**	**47,9**	**-2,34**	**0,01870**	**0,376**	**233899_x_at**	**Zinc finger and BTB domain containing 10**	***ZBTB10***
**668**	**53,0**	**121**	**-2,29**	**0,00384**	**0,308**	**209228_x_at**	**Tumor suppressor candidate 3**	***TUSC3***
**2640**	**58,6**	**133**	**-2,26**	**0,01800**	**0,373**	**228562_at**	**Zinc finger and BTB domain containing 10**	***ZBTB10***
**3143**	**33,2**	**74,8**	**-2,25**	**0,02230**	**0,387**	**205352_at**	**Serpin peptidase inhibitor, clade I (neuroSerpin), member 1**	***SERPINI1***
**1620**	**6,92**	**15,5**	**-2,24**	**0,01017**	**0,343**	**209227_at**	**Tumor suppressor candidate 3**	***TUSC3***
**630**	**31,0**	**67,9**	**-2,19**	**0,00357**	**0,308**	**201691_s_at**	**Tumor protein D52**	***TPD52***
**5030**	**8,84**	**19,3**	**-2,19**	**0,03867**	**0,420**	**206134_at**	**ADAM-like, decysin 1**	***ADAMDEC1***
**2632**	**4,98**	**10,8**	**-2,18**	**0,01793**	**0,372**	**241436_at**	**Sodium channel, nonvoltage-gated 1, gamma**	***SCNN1G***
**290**	**54,7**	**119**	**-2,17**	**0,00142**	**0,263**	**236655_at**	**Tumor protein D52**	***TPD52***
**3718**	**30,8**	**66,0**	**-2,14**	**0,02698**	**0,397**	**213201_s_at**	**Troponin T type 1 (skeletal, slow)**	***TNNT1***
**1456**	**10,0**	**21,5**	**-2,14**	**0,00890**	**0,334**	**231728_at**	**Calcyphosine**	***CAPS***
**975**	**31,7**	**67,0**	**-2,12**	**0,00567**	**0,315**	**203608_at**	**Aldehyde dehydrogenase 5 family, member A1**	***ALDH5A1***
**696**	**9,70**	**20,5**	**-2,11**	**0,00400**	**0,308**	**220936_s_at**	**H2A histone family, member J**	***H2AFJ***
**2086**	**11,3**	**23,6**	**-2,09**	**0,01361**	**0,356**	**222863_at**	**Zinc finger and BTB domain containing 10**	***ZBTB10***
**1868**	**22,7**	**47,1**	**-2,07**	**0,01207**	**0,353**	**219607_s_at**	**Membrane-spanning 4-domains, subfamily A, member 4**	***MS4A4A***
**1511**	**37,0**	**74,2**	**-2,00**	**0,00931**	**0,337**	**239847_at**	**cDNA clone IMAGE:6186815**	**-**
2925	30,3	60,6	-1,99	0,02051	0,383	242245_at	cDNA FLJ90705 fis, clone PLACE1007591	-
2061	12,6	25,0	-1,98	0,01344	0,356	239278_at	cDNA clone IMAGE:5301129	-
1092	20,2	39,9	-1,98	0,00641	0,319	207469_s_at	Pirin (iron-binding nuclear protein)	*PIR*
721	7,27	14,4	-1,98	0,00412	0,308	230782_at	Similar to Sorbitol dehydrogenase (L-iditol 2-dehydrogenase)	*LOC653381*
1970	8,59	17,0	-1,98	0,01287	0,355	227644_at	Regulating synaptic membrane exocytosis 4	*RIMS4*
3815	10,5	20,5	-1,95	0,02783	0,399	223565_at	Hypothetical protein MGC29506	*MGC29506*
1102	6,40	12,5	-1,95	0,00646	0,319	206214_at	2-acetyl-1-alkylglycerophosphocholine esterase	*PLA2G7*
879	44,4	86,4	-1,95	0,00509	0,313	226424_at	Calcyphosine	*CAPS*
5208	9,34	18,1	-1,93	0,04019	0,422	213906_at	v-Myb myeloblastosis viral oncogene homolog (avian)-like 1	*MYBL1*
712	23,0	44,3	-1,93	0,00409	0,308	218954_s_at	RNA polymerase III transcription initiation factor BRFU	*BRF2*
744	41,2	79,2	-1,92	0,00422	0,308	1558290_a_at	Pvt1 oncogene homolog, MYC activator (mouse)	*PVT1*
1324	14,2	27,2	-1,92	0,00793	0,327	229139_at	Junctophilin 1	*JPH1*
626	14,4	27,1	-1,89	0,00355	0,308	220016_at	AHNAK nucleoprotein	*AHNAK*
1012	37,1	69,9	-1,89	0,00586	0,316	222312_s_at	cDNA clone IMAGE:6186815	-
4227	27,5	51,7	-1,88	0,03132	0,405	214038_at	Chemokine (C-C motif) ligand 8	*CCL8*
2492	8,69	16,3	-1,87	0,01679	0,367	213768_s_at	Achaete-scute complex homolog 1 (Drosophila)	*ASCL1*
2288	9,72	18,1	-1,86	0,01520	0,363	237031_at	Full length insert cDNA clone YP08F12	-
3458	37,7	69,8	-1,85	0,02478	0,392	222731_at	Zinc finger, DHHC-type containing 2	*ZDHHC2*
2767	12,3	22,5	-1,83	0,01921	0,379	1554333_at	DnaJ (Hsp40) homolog, subfamily A, member 4	*DNAJA4*
2804	4,96	9,08	-1,83	0,01950	0,380	235801_at	-	-
1062	9,29	17,0	-1,83	0,00623	0,319	236828_at	Transcribed locus	-
112	91,1	165	-1,81	0,00050	0,240	202779_s_at	Ubiquitin-conjugating enzyme E2S	*UBE2S*
817	648	1176	-1,81	0,00467	0,312	225245_x_at	H2A histone family, member J	*H2AFJ*
3705	36,0	65,4	-1,81	0,02679	0,395	201195_s_at	Solute carrier family 7, member 5	*SLC7A5*
554	35,8	64,6	-1,81	0,00301	0,296	206066_s_at	RAD51 homolog C (S. cerevisiae)	*RAD51C*
868	111	200	-1,80	0,00502	0,313	202954_at	Ubiquitin-conjugating enzyme E2C	*UBE2C*
1761	28,1	50,5	-1,80	0,01123	0,348	221286_s_at	Hypothetical protein MGC29506	*MGC29506*
795	693	1241	-1,79	0,00454	0,311	224301_x_at	H2A histone family, member J	*H2AFJ*
5280	9,64	17,2	-1,79	0,04106	0,425	238646_at	Transcribed locus	-

Index	Mutated*	Normal*	FC	*P* value	FDR**	Probe set	Description	Gene symbol
5108	7,75	13,8	-1,79	0,03930	0,421	205697_at	Secretagogin, EF-hand calcium binding protein	*SCGN*
1008	35,2	62,9	-1,79	0,00585	0,316	204033_at	Thyroid hormone receptor interactor 13	*TRIP13*
2580	53,2	94,7	-1,78	0,01750	0,371	229796_at	SIX homeobox 4	*SIX4*
1883	21,4	37,8	-1,76	0,01223	0,355	222730_s_at	Zinc finger, DHHC-type containing 2	*ZDHHC2*
3745	3,00	5,27	-1,75	0,02724	0,397	235947_at	Similar to XP_001165016.1	-
3378	24,8	43,3	-1,75	0,02404	0,389	231069_at	Transcribed locus	-
915	371	644	-1,74	0,00527	0,313	200641_s_at	14-3-3 protein/cytosolic phospholipase A2	*YWHAZ*
1239	15,6	27,1	-1,73	0,00736	0,324	235330_at	Coiled-coil domain containing 117	*CCDC117*
822	4,90	8,47	-1,73	0,00469	0,312	238898_at	Transcribed locus	-
2457	5,97	10,3	-1,72	0,01647	0,366	209631_s_at	G protein-coupled receptor 37 (endothelin receptor type B-like)	*GPR37*
710	10,4	17,9	-1,72	0,00407	0,308	238624_at	Nemo-like kinase	*NLK*
1217	87,3	150	-1,72	0,00724	0,324	226384_at	Phosphatidic acid Phosphatase type 2 domain containing 1B	*PPAPDC1B*
1700	39,9	68,3	-1,71	0,01082	0,348	220588_at	Breast carcinoma amplified sequence 4	*BCAS4*
3691	3,56	6,07	-1,71	0,02666	0,395	1554314_at	Chromosome 6 open reading frame 141	*C6orf141*
3065	29,1	49,5	-1,70	0,02161	0,385	212314_at	KIAA0746 protein	*KIAA0746*
2204	5,88	9,96	-1,69	0,01457	0,361	1556097_at	cDNA DKFZp686A11113	-
5191	10,2	17,3	-1,69	0,03999	0,421	242338_at	Transmembrane protein 64	*TMEM64*
2284	7,04	11,9	-1,69	0,01518	0,363	236514_at	Acyl-CoA thioesterase 8	*ACOT8*
1666	23,1	39,1	-1,69	0,01053	0,345	228999_at	Chromodomain helicase DNA binding protein 2	*CHD2*
2262	70,8	120	-1,69	0,01505	0,363	212690_at	DDHD domain containing 2	*DDHD2*
2397	18,6	31,4	-1,69	0,01599	0,365	1555728_a_at	Membrane-spanning 4-domains, subfamily A, member 4	*MS4A4A*
523	350	589	-1,68	0,00279	0,291	201688_s_at	Tumor protein D52	*TPD52*
2810	57,0	95,8	-1,68	0,01955	0,380	221521_s_at	GINS complex subunit 2 (Psf2 homolog)	*GINS2*
2627	6,05	10,1	-1,68	0,01787	0,372	236105_at	Transcribed locus	-
456	53,0	88,8	-1,68	0,00244	0,288	239069_s_at	Similar to KIAA0825 protein [Pan troglodytes]	
1327	108	181	-1,67	0,00799	0,328	226568_at	Family with sequence similarity 102, member B	*FAM102B*
1024	48,9	81,8	-1,67	0,00593	0,316	1554334_a_at	DnaJ (Hsp40) homolog, subfamily A, member 4	*DNAJA4*
2085	11,0	18,4	-1,67	0,01360	0,356	214481_at	Histone cluster 1, H2am	*HIST1H2AM*
2405	77,5	129	-1,66	0,01607	0,365	218245_at	Tsukushin	*TSKU*
4633	13,9	23,1	-1,66	0,03495	0,412	213470_s_at	Heterogeneous nuclear ribonucleoprotein H1	*HNRPH1*
2685	162	269	-1,66	0,01840	0,375	210519_s_at	NAD(P)H dehydrogenase, quinone 1	*NQO1*
1606	72,0	119	-1,66	0,01006	0,342	223568_s_at	Phosphatidic acid Phosphatase type 2 domain containing 1B	*PPAPDC1B*
1302	60,3	99,7	-1,65	0,00775	0,325	218955_at	RNA polymerase III transcription initiation factor BRFU	*BRF2*
2821	14,7	24,3	-1,65	0,01969	0,381	212706_at	RAS p21 protein activator 4	*RASA4*
4835	15,2	25,0	-1,65	0,03687	0,417	213562_s_at	Squalene epoxidase	*SQLE*
3170	37,3	61,4	-1,65	0,02250	0,387	203145_at	Sperm associated antigen 5	*SPAG5*
1098	132	217	-1,65	0,00644	0,319	202431_s_at	v-Myc myelocytomatosis viral oncogene homolog (avian)	*MYC*
559	24,5	40,2	-1,64	0,00305	0,298	242304_at	Within bgcn homolog (Drosophila)	*WIBG*
3317	6,24	10,2	-1,64	0,02357	0,388	244779_at	cDNA FLJ34038 fis, clone FCBBF2005645	-
3346	170	279	-1,64	0,02376	0,388	226150_at	Phosphatidic acid Phosphatase type 2 domain containing 1B	*PPAPDC1B*
2948	18,2	29,8	-1,64	0,02068	0,384	212311_at	KIAA0746 protein	*KIAA0746*
1654	213	349	-1,64	0,01044	0,345	203381_s_at	Apolipoprotein E	*APOE*
106	361	590	-1,64	0,00043	0,217	218059_at	Zinc finger protein 706	*ZNF706*
3739	3,38	5,49	-1,63	0,02717	0,397	206757_at	Phosphodiesterase 5A, cGMP-specific	*PDE5A*
2187	16,7	27,0	-1,62	0,01449	0,361	230449_x_at	Similar toUbiquitin-conjugating enzyme E2 variant 1 isoform c	-
4174	14,5	23,4	-1,62	0,03086	0,404	239862_at	Transcribed locus	-
753	141	228	-1,62	0,00426	0,308	203382_s_at	Apolipoprotein E	*APOE*
1244	7,30	11,8	-1,61	0,00739	0,324	230361_at	Hypothetical protein KIAA1833	*KIAA1833*
2414	11,9	19,2	-1,61	0,01613	0,365	238694_at	Transcribed locus	-
1845	213	344	-1,61	0,01187	0,352	203534_at	LSM1 homolog, U6 small nuclear RNA associated (S. cerevisiae)	*LSM1*
2960	65,1	105	-1,61	0,02079	0,384	203362_s_at	MAD2 mitotic arrest deficient-like 1 (yeast)	*MAD2L1*
4603	9,86	15,9	-1,61	0,03480	0,412	1560101_at	Synapse defective 1, Rho GTPase, homolog 2 (C. elegans)	*SYDE2*
2479	110	177	-1,61	0,01670	0,367	204416_x_at	Apolipoprotein C-I	*APOC1*
1730	42,8	68,7	-1,61	0,01105	0,348	231784_s_at	WD repeats and SOF1 domain containing	*WDSOF1*
4462	42,7	68,5	-1,60	0,03352	0,411	202095_s_at	Baculoviral IAP repeat-containing 5 (survivin)	*BIRC5*
806	641	1026	-1,60	0,00460	0,311	225547_at	Small nucleolar RNA host gene (non-protein coding) 6	*SNHG6*
2891	9,33	14,9	-1,60	0,02030	0,383	223574_x_at	Protein phosphatase 2A1 B gamma subunit	*PPP2R2C*

Index	Mutated*	Normal*	FC	*P* value	FDR**	Probe set	Description	Gene symbol
1091	60,2	96,3	-1,60	0,00640	0,319	227765_at	cDNA clone IMAGE:4820809	-
371	788	1259	-1,60	0,00194	0,284	200640_at	14-3-3 protein/cytosolic phospholipase A2	*YWHAZ*
1535	52,3	83,7	-1,60	0,00951	0,337	225857_s_at	Hypothetical LOC388796	*LOC388796*
3470	14,3	22,9	-1,60	0,02486	0,392	219519_s_at	Sialic acid binding Ig-like lectin 1, sialoadhesin	*SIGLEC1*
436	13,6	21,8	-1,60	0,00230	0,288	221908_at	Transmembrane protein 118	*TMEM118*
999	412	656	-1,59	0,00578	0,316	201689_s_at	Tumor protein D52	*TPD52*
4713	32,5	51,7	-1,59	0,03566	0,413	230422_at	Formyl peptide receptor-like 2	*FPRL2*
3859	6,94	11,0	-1,59	0,02818	0,399	221345_at	Free fatty acid receptor 2	*FFAR2*
4446	4,16	6,61	-1,59	0,03340	0,411	1561817_at	cDNA FLJ35294 fis, clone PROST2008724	-
5258	222	352	-1,58	0,04077	0,424	201563_at	Sorbitol dehydrogenase	*SORD*
6067	15,5	24,6	-1,58	0,04948	0,446	228645_at	Small nucleolar RNA host gene (non-protein coding) 9	*SNHG9*
3302	18,4	28,9	-1,58	0,02351	0,388	229417_at	Transcribed locus	-
4076	112	176	-1,57	0,02989	0,401	209218_at	Squalene epoxidase	*SQLE*
3033	33,1	52,0	-1,57	0,02138	0,385	65588_at	Hypothetical LOC388796	*LOC388796*
324	12,0	18,8	-1,57	0,00161	0,272	230016_at	Chromosome 8 open reading frame 38	*C8orf38*
1292	679	1065	-1,57	0,00770	0,325	200638_s_at	14-3-3 protein/cytosolic phospholipase A2	*YWHAZ*
4604	42,0	65,8	-1,57	0,03480	0,412	227804_at	TLC domain containing 1	*TLCD1*
3849	7,38	11,6	-1,57	0,02810	0,399	204920_at	Carbamoyl-phosphate synthetase 1, mitochondrial	*CPS1*
2481	31,5	49,3	-1,57	0,01672	0,367	1554768_a_at	MAD2 mitotic arrest deficient-like 1 (yeast)	*MAD2L1*
2026	45,2	70,7	-1,57	0,01320	0,355	227132_at	Zinc finger protein 706	*ZNF706*
2298	106	165	-1,56	0,01527	0,363	216336_x_at	Metallothionein 1A	*MT1A*
4938	97,1	151	-1,56	0,03789	0,419	201467_s_at	NAD(P)H dehydrogenase, quinone 1	*NQO1*
2082	64,2	99,9	-1,56	0,01358	0,356	208002_s_at	Acyl-CoA thioesterase 7	*ACOT7*
5213	6,59	10,3	-1,56	0,04026	0,422	239752_at	cDNA FLJ38271 fis, clone FCBBF3002782	-
2013	11,1	17,3	-1,55	0,01313	0,355	228213_at	H2A histone family, member J	*H2AFJ*
3008	191	296	-1,55	0,02118	0,385	206461_x_at	Metallothionein 1H	*MT1H*
321	744	1155	-1,55	0,00161	0,272	200639_s_at	14-3-3 protein/cytosolic phospholipase A2	*YWHAZ*
5141	34,9	54,1	-1,55	0,03959	0,421	208079_s_at	Aurora kinase A	*AURKA*
2569	548	850	-1,55	0,01743	0,371	212185_x_at	Metallothionein 2A	*MT2A*
484	634	982	-1,55	0,00258	0,289	201690_s_at	Tumor protein D52	*TPD52*
433	32,2	49,8	-1,55	0,00228	0,288	221586_s_at	E2F transcription factor 5, p130-binding	*E2F5*
1889	179	277	-1,55	0,01225	0,355	225061_at	DnaJ (Hsp40) homolog, subfamily A, member 4	*DNAJA4*
5865	6,76	10,4	-1,54	0,04742	0,442	219159_s_at	SLAM family member 7	*SLAMF7*
4885	28,2	43,5	-1,54	0,03736	0,418	223253_at	Ependymin related protein 1 (zebrafish)	*EPDR1*
2664	149	230	-1,54	0,01822	0,374	235509_at	Chromosome 8 open reading frame 38	*C8orf38*
2307	19,2	29,5	-1,54	0,01532	0,363	226791_at	Kinesin family member C2	*KIFC2*
4266	57,2	87,9	-1,54	0,03174	0,406	202705_at	Cyclin B2	*CCNB2*
1450	163	249	-1,53	0,00886	0,334	202634_at	Polymerase (RNA) II (DNA directed) polypeptide K, 7.0kDa	*POLR2K*
2047	312	478	-1,53	0,01331	0,355	211456_x_at	Metallothionein 1 pseudogene 2	*MT1P2*
4171	21,4	32,8	-1,53	0,03082	0,404	217080_s_at	Homer homolog 2 (Drosophila)	*HOMER2*
2109	18,0	27,6	-1,53	0,01383	0,358	238012_at	Dipeptidyl-peptidase 7	*DPP7*
4873	43,0	65,6	-1,52	0,03720	0,417	223569_at	Phosphatidic acid Phosphatase type 2 domain containing 1B	*PPAPDC1B*
3697	79,2	121	-1,52	0,02674	0,395	226803_at	Chromatin modifying protein 4C	*CHMP4C*
1067	59,3	90,3	-1,52	0,00626	0,319	227451_s_at	Coiled-coil domain containing 90A	*CCDC90A*
1132	47,6	72,5	-1,52	0,00661	0,319	222155_s_at	G protein-coupled receptor 172A	*GPR172A*
2735	88,8	135	-1,52	0,01887	0,377	218514_at	Chromosome 17 open reading frame 71	*C17orf71*
5126	30,0	45,7	-1,52	0,03946	0,421	210117_at	Sperm associated antigen 1	*SPAG1*
3094	10,2	15,6	-1,52	0,02181	0,385	236113_at	Transcribed locus	-
1542	51,1	77,6	-1,52	0,00954	0,337	219188_s_at	MACRO domain containing 1	*MACROD1*
3580	28,4	43,1	-1,52	0,02586	0,394	221194_s_at	PTD016 protein	*LOC51136*
2352	65,1	98,7	-1,52	0,01569	0,364	201853_s_at	Cell division cycle 25 homolog B (S. pombe)	*CDC25B*
1295	8,69	13,2	-1,52	0,00771	0,325	230330_at	cDNA FLJ30011 fis, clone 3NB692000276	-
1526	22,2	33,6	-1,52	0,00943	0,337	221909_at	Transmembrane protein 118	*TMEM118*
4849	12,6	19,1	-1,52	0,03694	0,417	238818_at	KIAA1429 protein	*KIAA1429*
826	98,8	149	-1,51	0,00471	0,312	203011_at	Inositol(myo)-1(or 4)-monophosphatase 1	*IMPA1*
1796	259	392	-1,51	0,01156	0,351	202635_s_at	Polymerase (RNA) II (DNA directed) polypeptide K, 7.0kDa	*POLR2K*
1998	79,4	120	-1,51	0,01306	0,355	219363_s_at	MTERF domain containing 1	*MTERFD1*

Index	Mutated*	Normal*	FC	*P* value	FDR**	Probe set	Description	Gene symbol
2432	21,3	32,1	-1,51	0,01626	0,365	209573_s_at	Chromosome 18 open reading frame 1	*C18orf1*
4252	115	173	-1,51	0,03157	0,406	217165_x_at	Metallothionein 1F	*MT1F*
2676	5,02	7,57	-1,51	0,01831	0,374	222309_at	Chromosome 6 open reading frame 62	*C6orf62*
2941	4,19	6,31	-1,51	0,02062	0,383	213609_s_at	Seizure related 6 homolog (mouse)-like	*SEZ6L*
2636	19,5	29,3	-1,51	0,01794	0,372	210334_x_at	Baculoviral IAP repeat-containing 5 (survivin)	*BIRC5*
2741	7,79	11,7	-1,51	0,01891	0,377	219403_s_at	Heparanase	*HPSE*
4616	11,6	17,5	-1,51	0,03484	0,412	219697_at	Heparan sulfate (glucosamine) 3-O-sulfotransferase 2	*HS3ST2*
3007	45,5	68,4	-1,50	0,02117	0,385	225203_at	Protein phosphatase 1, regulatory (inhibitor) subunit 16A	*PPP1R16A*
1627	61,5	92,6	-1,50	0,01023	0,344	225378_at	Vacuolar protein sorting 37 homolog A (S. cerevisiae)	*VPS37A*
3557	108	163	-1,50	0,02565	0,394	224356_x_at	Membrane-spanning 4-domains, subfamily A, member 6A	*MS4A6A*
1096	7,40	11,1	-1,50	0,00642	0,319	1556613_s_at	Hypothetical protein LOC203107	*LOC203107*
4441	63,7	95,5	-1,50	0,03333	0,410	218009_s_at	Protein regulator of cytokinesis 1	*PRC1*
5480	77,2	116	-1,50	0,04336	0,433	235010_at	Hypothetical protein LOC729013	*LOC729013*
4289	113	169	-1,50	0,03189	0,406	201484_at	Suppressor of Ty 4 homolog 1 (S. cerevisiae)	*SUPT4H1*
583	28,2	42,1	-1,50	0,00319	0,300	227935_s_at	Polycomb group ring finger 5	*PCGF5*
1331	24,8	36,9	-1,49	0,00800	0,328	212563_at	Block of proliferation 1	*BOP1*
1482	131	195	-1,49	0,00911	0,336	200924_s_at	Solute carrier family 3, member 2	*SLC3A2*
5681	331	494	-1,49	0,04535	0,436	208581_x_at	Metallothionein 1X	*MT1X*
3589	34,3	51,0	-1,49	0,02590	0,394	228024_at	Vacuolar protein sorting 37 homolog A (S. cerevisiae)	*VPS37A*
310	7,50	11,2	-1,49	0,00150	0,265	241957_x_at	Lin-7 homolog B (C. elegans)	*LIN7B*
848	11,3	16,7	-1,48	0,00489	0,313	212619_at	KIAA0286 protein	*KIAA0286*
4312	51,2	75,9	-1,48	0,03210	0,407	226943_at	Keratin, hair, basic, 5	*KRTHB5*
1904	44,0	65,2	-1,48	0,01238	0,355	217499_x_at	Olfactory receptor, family 7, subfamily E, member 37 pseudogene	*OR7E37P*
3996	7,81	11,6	-1,48	0,02921	0,400	1555606_a_at	Glycerophosphodiester phosphodiesterase domain containing 1	*GDPD1*
2054	4,56	6,74	-1,48	0,01336	0,356	228047_at	Small nucleolar RNA, H/ACA box 72	*SNORA72*
2880	22,8	33,6	-1,48	0,02021	0,383	1555461_at	-	-
2521	54,3	80,1	-1,47	0,01701	0,369	228488_at	TBC1 domain family, member 16	*TBC1D16*
5834	145	213	-1,47	0,04713	0,442	209185_s_at	Insulin receptor substrate 2	*IRS2*
5303	689	1015	-1,47	0,04139	0,426	200800_s_at	Heat shock 70kDa protein 1A	*HSPA1A*
3389	33,6	49,6	-1,47	0,02414	0,389	202823_at	Transcription elongation factor B (SIII), polypeptide 1	*TCEB1*
308	134	198	-1,47	0,00148	0,263	218680_x_at	Huntingtin interacting protein K	*HYPK*
3659	6,09	8,97	-1,47	0,02642	0,395	239562_at	NADP-dependent methylenetetrahydrofolate dehydrogenase 2-like protein	*MTHFD2L*
3096	43,1	63,5	-1,47	0,02182	0,385	222912_at	Arrestin, beta 1	*ARRB1*
3898	14,8	21,8	-1,47	0,02848	0,399	214560_at	Formyl peptide receptor-like 2	*FPRL2*
4720	148	218	-1,47	0,03570	0,413	204745_x_at	Metallothionein 1G	*MT1G*
5517	3,53	5,18	-1,47	0,04375	0,433	217564_s_at	Carbamoyl-phosphate synthetase 1, mitochondrial	*CPS1*
2150	25,3	37,2	-1,47	0,01415	0,360	222026_at	RNA binding motif (RNP1, RRM) protein 3	*RBM3*
1615	5,69	8,34	-1,47	0,01013	0,343	217599_s_at	MyoD family inhibitor domain containing	*MDFIC*
2266	53,6	78,5	-1,47	0,01507	0,363	202481_at	Dehydrogenase/reductase (SDR family) member 3	*DHRS3*
3731	265	388	-1,47	0,02709	0,397	224447_s_at	Chromosome 17 open reading frame 37	*C17orf37*
1588	98,9	145	-1,47	0,00988	0,340	221539_at	Eukaryotic translation initiation factor 4E binding protein 1	*EIF4EBP1*
5996	92,7	136	-1,46	0,04875	0,444	209380_s_at	ATP-binding cassette, sub-family C (CFTR/MRP), member 5	*ABCC5*
1499	65,1	95,1	-1,46	0,00923	0,336	229050_s_at	Small nucleolar RNA host gene (non-protein coding) 7	*SNHG7*
1223	14,2	20,7	-1,46	0,00728	0,324	204126_s_at	CDC45 cell division cycle 45-like (S. cerevisiae)	*CDC45L*
372	130	189	-1,46	0,00195	0,284	233746_x_at	Huntingtin interacting protein K	*HYPK*
3404	6,62	9,64	-1,46	0,02428	0,390	1562904_s_at	Similar to CG7889-PA	*FLJ10661*
2242	184	268	-1,46	0,01489	0,363	201433_s_at	Phosphatidylserine synthase 1	*PTDSS1*
3183	6,40	9,30	-1,45	0,02256	0,387	210341_at	Myelin transcription factor 1	*MYT1*
4533	16,4	23,9	-1,45	0,03425	0,412	213599_at	Opa interacting protein 5	*OIP5*
2107	17,0	24,7	-1,45	0,01383	0,358	40020_at	Cadhetrin, EGF LAG seven-pass G-type receptor 3	*CELSR3*
4680	18,3	26,6	-1,45	0,03534	0,413	1552623_at	Hematopoietic SH2 domain containing	*HSH2D*
1782	27,6	40,0	-1,45	0,01137	0,349	228357_at	Unkempt homolog (Drosophila)	*UNK*
4830	62,2	89,9	-1,45	0,03679	0,416	201801_s_at	Solute carrier family 29, member 1	*SLC29A1*
2578	31,1	45,0	-1,45	0,01749	0,371	206574_s_at	Protein tyrosine phosphatase type IVA, member 3	*PTP4A3*
5119	34,1	49,3	-1,45	0,03941	0,421	204092_s_at	Aurora kinase A	*AURKA*
597	36,6	52,9	-1,44	0,00329	0,301	215030_at	G-rich RNA sequence binding factor 1	*GRSF1*
734	58,6	84,7	-1,44	0,00419	0,308	215905_s_at	WD repeat domain 57	*WDR57*

Index	Mutated*	Normal*	FC	*P* value	FDR**	Probe set	Description	Gene symbol
1246	25,7	37,2	-1,44	0,00740	0,324	205870_at	Bradykinin receptor B2	*BDKRB2*
1875	12,1	17,5	-1,44	0,01213	0,354	230454_at	Islet cell autoantigen 1,69kDa-like	*ICA1L*
1554	37,1	53,5	-1,44	0,00959	0,337	227787_s_at	Mediator complex subunit 30	*MED30*
1333	140	202	-1,44	0,00801	0,328	202433_at	Solute carrier family 35, member B1	*SLC35B1*
2791	43,3	62,5	-1,44	0,01941	0,380	202219_at	Solute carrier family 6, member 8	*SLC6A8*
2395	57,3	82,7	-1,44	0,01599	0,365	212983_at	v-HA-RAS Harvey rat sarcoma viral oncogene homolog	*HRAS*
960	22,4	32,4	-1,44	0,00554	0,315	218832_x_at	Arrestin, beta 1	*ARRB1*
1272	176	254	-1,44	0,00756	0,325	218049_s_at	Mitochondrial ribosomal protein L13	*MRPL13*
533	158	227	-1,44	0,00285	0,292	219449_s_at	Transmembrane protein 70	*TMEM70*
3565	4,68	6,75	-1,44	0,02574	0,394	1553175_s_at	Phosphodiesterase 5A, cGMP-specific	*PDE5A*
3125	26,4	38,1	-1,44	0,02214	0,387	226446_at	Hairy and enhancer of split 6 (Drosophila)	*HES6*
2369	16,6	23,8	-1,44	0,01580	0,364	209753_s_at	Thymopoietin	*TMPO*
1544	209	301	-1,44	0,00954	0,337	200661_at	Cathepsin A	*CTSA*
818	87,4	126	-1,44	0,00467	0,312	225723_at	Chromosome 6 open reading frame 129	*C6orf129*
6001	8,26	11,9	-1,44	0,04877	0,444	205692_s_at	CD38 molecule	*CD38*
3710	111	160	-1,44	0,02684	0,396	223922_x_at	Membrane-spanning 4-domains, subfamily A, member 6A	*MS4A6A*
169	17,9	25,7	-1,44	0,00078	0,244	235689_at	Mitochondrial methionyl-tRNA formyltransferase	*MTFMT*
6036	4,37	6,28	-1,44	0,04915	0,445	241412_at	Betacellulin	*BTC*
3896	67,2	96,4	-1,44	0,02846	0,399	1558703_at	Solute carrier family 46, member 1	*SLC46A1*
2546	161	231	-1,43	0,01720	0,369	226223_at	Transcribed locus	-
4549	69,1	99,0	-1,43	0,03439	0,412	209849_s_at	RAD51 homolog C (S. cerevisiae)	*RAD51C*
784	90,7	130	-1,43	0,00449	0,311	221020_s_at	Solute carrier family 25, member 32	*SLC25A32*
1856	164	235	-1,43	0,01195	0,352	223156_at	Mitochondrial ribosomal protein S23	*MRPS23*
2889	10,1	14,4	-1,43	0,02027	0,383	215731_s_at	M-phase phosphoprotein 9	*MPHOSPH9*
234	18,8	26,9	-1,43	0,00108	0,250	235015_at	Placenta Cot 25-normalized	-
3818	25,2	35,9	-1,42	0,02786	0,399	227936_at	Transmembrane protein 68	*TMEM68*
4759	62,0	88,2	-1,42	0,03608	0,414	225436_at	Family with sequence similarity 108, member C1	*FAM108C1*
4063	20,4	29,0	-1,42	0,02980	0,401	219000_s_at	Defective in sister chromatid cohesion homolog 1 (S. cerevisiae)	*DCC1*
1252	4,20	5,98	-1,42	0,00746	0,325	238566_at	Transcribed locus	-
746	83,7	119	-1,42	0,00423	0,308	202204_s_at	Autocrine motility factor receptor	*AMFR*
2005	34,8	49,4	-1,42	0,01308	0,355	221520_s_at	Cell division cycle associated 8	*CDCA8*
1912	52,3	74,3	-1,42	0,01244	0,355	202070_s_at	Isocitrate dehydrogenase 3 (NAD+) alpha	*IDH3A*
4523	80,3	114	-1,42	0,03413	0,412	226319_s_at	THO complex 4	*THOC4*
1190	118	167	-1,42	0,00703	0,322	212056_at	KIAA0182 protein	*KIAA0182*
5276	108	153	-1,42	0,04100	0,425	218187_s_at	Chromosome 8 open reading frame 33	*C8orf33*
771	154	218	-1,42	0,00442	0,311	224281_s_at	Neugrin, neurite outgrowth associated	*NGRN*
2241	113	160	-1,41	0,01489	0,363	218695_at	Exosome component 4	*EXOSC4*
230	48,1	68,1	-1,41	0,00106	0,250	218906_x_at	Kinesin light chain 2	*KLC2*
5714	180	255	-1,41	0,04574	0,438	201483_s_at	Suppressor of Ty 4 homolog 1 (S. cerevisiae)	*SUPT4H1*
4461	88,3	125	-1,41	0,03352	0,411	225374_at	Chromosome 17 open reading frame 32	*C17orf32*
900	19,1	26,9	-1,41	0,00521	0,313	1568596_a_at	Trophinin associated protein (tastin)	*TROAP*
4183	10,6	14,9	-1,41	0,03092	0,404	209924_at	Chemokine (C-C motif) ligand 18	*CCL18*
4385	31,3	44,1	-1,41	0,03267	0,407	214542_x_at	Histone cluster 1, H2ai	*HIST1H2AI*
1211	14,4	20,2	-1,41	0,00718	0,324	239699_s_at	Postmeiotic segregation increased 2-like 1	*PMS2L1*
853	13,7	19,2	-1,41	0,00491	0,313	222276_at	Transcribed locus	-
3393	335	471	-1,41	0,02419	0,390	208808_s_at	High-mobility group box 2	*HMGB2*
3930	15,6	21,9	-1,40	0,02873	0,399	206753_at	Retinol dehydrogenase 16 (all-trans)	*RDH16*
5129	7,00	9,83	-1,40	0,03946	0,421	1555216_a_at	Hypothetical LOC645722	*LOC645722*
3805	5,85	8,21	-1,40	0,02771	0,398	32128_at	Chemokine (C-C motif) ligand 18	*CCL18*
547	517	726	-1,40	0,00296	0,295	223880_x_at	Chromosome 20 open reading frame 24	*C20orf24*
2822	35,2	49,4	-1,40	0,01970	0,381	218045_x_at	Parathymosin	*PTMS*
1003	391	548	-1,40	0,00580	0,316	200078_s_at	ATPase, H+ transporting, lysosomal 21kDa, V0 subunit b	*ATP6V0B*
2470	49,2	69,0	-1,40	0,01659	0,367	200676_s_at	Ubiquitin-conjugating enzyme E2L 3	*UBE2L3*
4402	18,8	26,3	-1,40	0,03285	0,408	219751_at	SET domain containing 6	*SETD6*
671	15,6	21,8	-1,40	0,00386	0,308	1553043_a_at	CD300 molecule-like family member f	*CD300LF*
812	28,0	39,2	-1,40	0,00462	0,311	224980_at	LEM domain containing 2	*LEMD2*
3715	6,82	9,55	-1,40	0,02694	0,396	204534_at	Vitronectin	*VTN*

Index	Mutated*	Normal*	FC	*P* value	FDR**	Probe set	Description	Gene symbol
1348	33,3	46,6	-1,40	0,00809	0,328	205695_at	Serine dehydratase	*SDS*
1715	12,4	17,3	-1,40	0,01099	0,348	238780_s_at	cDNA DKFZp779F2345	-
2106	5,76	8,04	-1,40	0,01379	0,358	207746_at	Polymerase (DNA directed), theta	*POLQ*
4339	20,9	29,2	-1,39	0,03231	0,407	218521_s_at	Ubiquitin-conjugating enzyme E2W	*UBE2W*
4054	82,3	115	-1,39	0,02974	0,401	203790_s_at	Heat-responsive protein 12	*HRSP12*
2953	10,8	15,0	-1,39	0,02073	0,384	223845_at	Hypothetical protein BC001437	*LOC144305*
3448	9,48	13,2	-1,39	0,02469	0,391	1557147_a_at	Full length insert cDNA YH73H08	-
779	113	157	-1,39	0,00447	0,311	203109_at	Ubiquitin-conjugating enzyme E2M	*UBE2M*
4961	47,7	66,5	-1,39	0,03805	0,419	209408_at	Kinesin family member 2C	*KIF2C*
3037	6,19	8,62	-1,39	0,02140	0,385	1554020_at	Bicaudal D homolog 1 (Drosophila)	*BICD1*
2069	5,06	7,03	-1,39	0,01353	0,356	214874_at	Plakophilin 4	*PKP4*
813	21,8	30,2	-1,39	0,00465	0,312	215728_s_at	Acyl-CoA thioesterase 7	*ACOT7*
6034	5,34	7,41	-1,39	0,04911	0,445	220484_at	Mucolipin 3	*MCOLN3*
4409	28,9	40,1	-1,39	0,03292	0,408	201710_at	v-Myb myeloblastosis viral oncogene homolog (avian)-like 2	*MYBL2*
4546	62,4	86,5	-1,39	0,03436	0,412	202392_s_at	Phosphatidylserine decarboxylase	*PISD*
3150	174	241	-1,39	0,02235	0,387	221504_s_at	ATPase, H+ transporting, lysosomal 50/57kDa, V1 subunit H	*ATP6V1H*
2467	466	645	-1,39	0,01658	0,367	202824_s_at	Transcription elongation factor B (SIII), polypeptide 1	*TCEB1*
2367	84,4	117	-1,39	0,01579	0,364	226805_at	Chromosome 20 open reading frame 142	*C20orf142*
345	241	333	-1,39	0,00177	0,278	227291_s_at	BolA homolog 3 (E. coli)	*BOLA3*
4587	13,4	18,6	-1,38	0,03469	0,412	221638_s_at	Syntaxin 16	*STX16*
3704	4,23	5,85	-1,38	0,02679	0,395	233446_at	One cut homeobox 2	*ONECUT2*
4176	20,9	29,0	-1,38	0,03086	0,404	231952_at	Hypothetical protein LOC731450	*LOC731450*
3116	16,7	23,0	-1,38	0,02203	0,387	238728_at	Similar to KIAA0825 protein	-
4955	14,5	20,0	-1,38	0,03801	0,419	235006_at	CDKN2A interacting protein N-terminal like	*CDKN2AIPNL*
379	64,2	88,6	-1,38	0,00198	0,284	218647_s_at	YrdC domain containing (E. coli)	*YRDC*
292	11,0	15,2	-1,38	0,00143	0,263	209544_at	Receptor-interacting serine-threonine kinase 2	*RIPK2*
2472	19,9	27,4	-1,38	0,01661	0,367	221965_at	M-phase phosphoprotein 9	*MPHOSPH9*
3159	123	170	-1,38	0,02242	0,387	202353_s_at	Proteasome (prosome, macropain) 26S subunit, non-ATPase, 12	*PSMD12*
937	4,19	5,78	-1,38	0,00537	0,313	226462_at	Syntaxin binding protein 6 (amisyn)	*STXBP6*
350	60,7	83,6	-1,38	0,00179	0,278	219675_s_at	UDP-glucuronate decarboxylase 1	*UXS1*
4516	129	178	-1,38	0,03404	0,412	201266_at	Thioredoxin reductase 1	*TXNRD1*
2066	62,3	85,8	-1,38	0,01349	0,356	232662_x_at	Chromosome 10 open reading frame 58	*C10orf58*
6041	97,1	134	-1,38	0,04919	0,445	225355_at	cDNA DKFZP761M1511	-
2957	183	251	-1,37	0,02076	0,384	204426_at	Transmembrane emp24 domain trafficking protein 2	*TMED2*
4670	225	309	-1,37	0,03526	0,413	209517_s_at	Ash2 (absent, small, or homeotic)-like (Drosophila)	*ASH2L*
3296	1244	1709	-1,37	0,02349	0,388	200799_at	Heat shock 70kDa protein 1A	*HSPA1A*
2910	26,5	36,4	-1,37	0,02045	0,383	225747_at	Coenzyme Q10 homolog A (S. cerevisiae)	*COQ10A*
5005	8,83	12,1	-1,37	0,03844	0,420	222376_at	Transcribed locus	-
887	559	767	-1,37	0,00512	0,313	217835_x_at	Chromosome 20 open reading frame 24	*C20orf24*
3062	170	234	-1,37	0,02157	0,385	224743_at	Inositol monophosphatase domain containing 1	*IMPAD1*
5761	19,1	26,3	-1,37	0,04629	0,439	229106_at	Dynein, light chain, LC8-type 2	*DYNLL2*
213	6,72	9,22	-1,37	0,00096	0,244	236478_at	Interferon (alpha, beta and omega) receptor 1	*IFNAR1*
644	19,7	27,1	-1,37	0,00367	0,308	228627_at	Tousled-like kinase 2	*TLK2*
4317	11,1	15,3	-1,37	0,03215	0,407	230591_at	Hypothetical protein LOC729887	*LOC729887*
2703	115	158	-1,37	0,01854	0,375	58696_at	Exosome component 4	*EXOSC4*
3354	50,7	69,5	-1,37	0,02382	0,388	209065_at	Ubiquinol-cytochrome c reductase binding protein	*UQCRB*
4730	194	265	-1,37	0,03582	0,414	225676_s_at	WD repeats and SOF1 domain containing	*WDSOF1*
1813	304	416	-1,37	0,01169	0,351	224603_at	cDNA clone IMAGE:3831740	-
3677	172	236	-1,37	0,02657	0,395	210296_s_at	Peroxisomal membrane protein 3, 35kDa	*PXMP3*
3938	155	212	-1,37	0,02876	0,399	201584_s_at	DEAD (Asp-Glu-Ala-Asp) box polypeptide 39	*DDX39*
5579	10,3	14,1	-1,37	0,04449	0,436	1558906_a_at	cDNA clone IMAGE:5265020	-
2587	12,0	16,4	-1,37	0,01755	0,371	206109_at	Fucosyltransferase 1	*FUT1*
3902	9,30	12,7	-1,37	0,02851	0,399	212427_at	KIAA0368 protein	*KIAA0368*
2878	61,0	83,4	-1,37	0,02020	0,383	218923_at	Chitobiase, di-N-acetyl-	*CTBS*
2753	4,56	6,23	-1,37	0,01905	0,378	237246_at	Transcribed locus	-
5515	53,2	72,8	-1,37	0,04371	0,433	229444_at	Hypothetical protein LOC729776	*LOC729776*
1266	355	486	-1,37	0,00753	0,325	224376_s_at	Chromosome 20 open reading frame 24	*C20orf24*

Index	Mutated*	Normal*	FC	*P* value	FDR**	Probe set	Description	Gene symbol
5619	200	273	-1,37	0,04484	0,436	228123_s_at	Abhydrolase domain containing 12	*ABHD12*
5643	11,2	15,3	-1,36	0,04502	0,436	216985_s_at	Syntaxin 3	*STX3*
5240	30,1	41,0	-1,36	0,04059	0,423	220488_s_at	Breast carcinoma amplified sequence 3	*BCAS3*
3240	12,0	16,4	-1,36	0,02299	0,388	220233_at	F-box protein 17	*FBXO17*
5945	25,6	34,9	-1,36	0,04817	0,443	227037_at	Similar to CG12314 gene product	*LOC201164*
711	393	535	-1,36	0,00408	0,308	201923_at	Peroxiredoxin 4	*PRDX4*
4206	25,0	34,1	-1,36	0,03110	0,404	228517_at	Chromosome 1 open reading frame 149	*C1orf149*
4672	13,6	18,6	-1,36	0,03529	0,413	208033_s_at	Zinc finger homeobox 3	*ZFHX3*
4717	7,75	10,6	-1,36	0,03569	0,413	209535_s_at	-	-
3388	251	342	-1,36	0,02414	0,389	203880_at	COX17 cytochrome c oxidase assembly homolog (S. cerevisiae)	*COX17*
4625	15,2	20,7	-1,36	0,03489	0,412	202094_at	Baculoviral IAP repeat-containing 5 (survivin)	*BIRC5*
1256	18,7	25,5	-1,36	0,00748	0,325	227245_at	Chromosome 12 open reading frame 30	*C12orf30*
4806	97,0	132	-1,36	0,03648	0,415	222673_x_at	Transmembrane protein 57	*TMEM57*
5502	7,43	10,1	-1,36	0,04357	0,433	238214_at	Solute carrier family 26, member 7	*SLC26A7*
4511	38,4	52,2	-1,36	0,03401	0,412	216125_s_at	RAN binding protein 9	*RANBP9*
2285	48,0	65,3	-1,36	0,01519	0,363	218805_at	GTPase, IMAP family member 5	*GIMAP5*
6007	125	171	-1,36	0,04882	0,444	218831_s_at	Fc fragment of IgG, receptor, transporter, alpha	*FCGRT*
3038	35,1	47,7	-1,36	0,02140	0,385	217191_x_at	-	-
172	9,67	13,2	-1,36	0,00080	0,244	239067_s_at	Pannexin 2	*PANX2*
2864	34,4	46,8	-1,36	0,02011	0,383	222665_at	Family with sequence similarity 82, member B	*FAM82B*
2296	38,9	52,8	-1,36	0,01527	0,363	209695_at	Protein tyrosine phosphatase type IVA, member 3	*PTP4A3*
1607	241	327	-1,36	0,01006	0,342	219402_s_at	Der1-like domain family, member 1	*DERL1*
3606	194	263	-1,36	0,02600	0,394	224601_at	cDNA clone IMAGE:3831740	-
4359	63,6	86,4	-1,36	0,03242	0,407	226287_at	Coiled-coil domain containing 34	*CCDC34*
1561	224	304	-1,36	0,00968	0,339	217956_s_at	Enolase-phosphatase 1	*ENOPH1*
1465	251	341	-1,36	0,00897	0,334	218165_at	Chromosome 1 open reading frame 149	*C1orf149*
1537	6,94	9,42	-1,36	0,00952	0,337	205262_at	Potassium voltage-gated channel subfamily H member 2	*KCNH2*
5799	507	687	-1,36	0,04677	0,441	201577_at	Non-metastatic cells 1, protein (NM23A) expressed in	*NME1*
466	24,3	33,0	-1,36	0,00246	0,288	204817_at	Extra spindle pole bodies homolog 1 (S. cerevisiae)	*ESPL1*
4373	119	162	-1,36	0,03252	0,407	209567_at	RRS1 ribosome biogenesis regulator homolog (S. cerevisiae)	*RRS1*
2245	66,8	90,4	-1,35	0,01493	0,363	227696_at	Exosome component 6	*EXOSC6*
1990	7,42	10,0	-1,35	0,01302	0,355	1560620_at	Potassium channel, subfamily K, member 15	*KCNK15*
6103	5,74	7,77	-1,35	0,04983	0,446	222773_s_at	UDP-GalNAc:polypeptide N-acetylgalactosaminyltransferase 12	*GALNT12*
2222	21,4	29,0	-1,35	0,01476	0,363	204629_at	Parvin, beta	*PARVB*
4011	107	144	-1,35	0,02937	0,400	218447_at	Chromosome 16 open reading frame 61	*C16orf61*
3860	38,6	52,2	-1,35	0,02819	0,399	227605_at	Transcribed locus	-
266	23,7	32,0	-1,35	0,00126	0,260	229321_s_at	cDNA FLJ35002 fis, clone OCBBF2011914	-
4693	17,2	23,3	-1,35	0,03545	0,413	222218_s_at	Paired immunoglobin-like type 2 receptor alpha	*PILRA*
766	47,0	63,5	-1,35	0,00434	0,310	1558044_s_at	Exosome component 6	*EXOSC6*
831	37,2	50,2	-1,35	0,00475	0,312	222703_s_at	YrdC domain containing (E. coli)	*YRDC*
2050	52,0	70,2	-1,35	0,01332	0,355	218455_at	NFS1 nitrogen fixation 1 homolog (S. cerevisiae)	*NFS1*
2912	5,28	7,13	-1,35	0,02045	0,383	220310_at	Tubulin, alpha-like 3	*TUBAL3*
405	16,5	22,3	-1,35	0,00213	0,286	223628_at	Transmembrane protein 191A	*TMEM191A*
305	7,76	10,5	-1,35	0,00147	0,263	1563445_x_at	Cathepsin L-like 3	*CTSLL3*
386	5,23	7,06	-1,35	0,00201	0,284	227491_at	cDNA FLJ22539 fis, clone HRC13227	-
3191	288	389	-1,35	0,02262	0,387	219549_s_at	Reticulon 3	*RTN3*
1401	51,9	70,0	-1,35	0,00847	0,330	209383_at	DNA-damage-inducible transcript 3	*DDIT3*
1968	90,7	122	-1,35	0,01286	0,355	203931_s_at	Mitochondrial ribosomal protein L12	*MRPL12*
5341	71,9	97,0	-1,35	0,04184	0,428	226413_at	Hypothetical gene supported by BC047417	*LOC400027*
1505	41,4	55,8	-1,35	0,00925	0,336	218308_at	Transforming, acidic coiled-coil containing protein 3	*TACC3*
2946	118	159	-1,35	0,02065	0,383	213553_x_at	Apolipoprotein C-I	*APOC1*
5332	47,5	64,0	-1,35	0,04174	0,428	218951_s_at	PI-PLC X domain-containing protein 1	*PLCXD1*
5500	11,9	16,1	-1,35	0,04354	0,433	223874_at	Actin-related Arp11	*ARP11*
1035	15,4	20,7	-1,35	0,00601	0,318	211825_s_at	Ewing sarcoma breakpoint region 1	*EWSR1*
1084	127	170	-1,35	0,00634	0,319	229742_at	Hypothetical LOC145853	*LOC145853*
2020	174	234	-1,35	0,01317	0,355	218074_at	Family with sequence similarity 96, member B	*FAM96B*
5904	4,64	6,24	-1,35	0,04784	0,443	1554614_a_at	Polypyrimidine tract binding protein 2	*PTBP2*

Index	Mutated*	Normal*	FC	*P* value	FDR**	Probe set	Description	Gene symbol
293	13,1	17,6	-1,35	0,00144	0,263	1555943_at	Phosphoglycerate mutase family member 5	*PGAM5*
4965	7,71	10,4	-1,35	0,03810	0,419	242473_at	TNF receptor-associated factor 4	*TRAF4*
752	25,5	34,3	-1,35	0,00425	0,308	225233_at	Musashi homolog 2 (Drosophila)	*MSI2*
5228	19,9	26,8	-1,35	0,04045	0,423	203713_s_at	Lethal giant larvae homolog 2 (Drosophila)	*LLGL2*
3127	327	440	-1,34	0,02217	0,387	208836_at	ATPase, Na+/K+ transporting, beta 3 polypeptide	*ATP1B3*
476	11,8	15,8	-1,34	0,00255	0,289	238802_at	Trypsin domain containing 1	*TYSND1*
2314	98,6	132	-1,34	0,01537	0,363	200684_s_at	Ubiquitin-conjugating enzyme E2L 3	*UBE2L3*
3877	325	437	-1,34	0,02832	0,399	200708_at	Glutamic-oxaloacetic transaminase 2, mitochondrial	*GOT2*
1260	80,5	108	-1,34	0,00750	0,325	216977_x_at	Small nuclear ribonucleoprotein polypeptide A'	*SNRPA1*
3610	41,8	56,1	-1,34	0,02606	0,394	202039_at	TGFB1-induced anti-apoptotic factor 1	*TIAF1*
1529	13,5	18,1	-1,34	0,00947	0,337	244721_at	Tumor protein p53 inducible nuclear protein 1	*TP53INP1*
2123	29,4	39,4	-1,34	0,01398	0,359	38158_at	Extra spindle pole bodies homolog 1 (S. cerevisiae)	*ESPL1*
384	28,7	38,5	-1,34	0,00200	0,284	228064_at	Chromosome 22 open reading frame 36	*C22orf36*
5530	88,3	118	-1,34	0,04389	0,434	202583_s_at	RAN binding protein 9	*RANBP9*
5671	37,0	49,5	-1,34	0,04525	0,436	224753_at	Cell division cycle associated 5	*CDCA5*
5818	20,4	27,3	-1,34	0,04703	0,442	222756_s_at	Arrestin, beta 1	*ARRB1*
5521	45,0	60,2	-1,34	0,04380	0,433	219517_at	Elongation factor RNA polymerase II-like 3	*ELL3*
5205	15,2	20,3	-1,34	0,04017	0,422	1557626_at	cDNA FLJ39805 fis, clone SPLEN2007951	-
1144	5,97	7,98	-1,34	0,00670	0,320	206486_at	Lymphocyte-activation gene 3	*LAG3*
2455	143	192	-1,34	0,01646	0,366	224739_at	Pim-3 oncogene	*PIM3*
4332	6,90	9,22	-1,34	0,03225	0,407	238114_at	Protein-L-isoaspartate O-methyltransferase domain-containing protein 1	*PCMTD1*
25	9,20	12,3	-1,34	0,00011	0,201	238554_at	Cytochrome b5 type B (outer mitochondrial membrane)	*CYB5B*
70	22,6	30,2	-1,34	0,00027	0,201	213072_at	Cysteine/histidine-rich 1	*CYHR1*
2201	32,3	43,2	-1,34	0,01456	0,361	224462_s_at	Coiled-coil-helix-Coiled-coil-helix domain containing 6	*CHCHD6*
2828	186	248	-1,34	0,01973	0,381	211985_s_at	Calmodulin 1 (phosphorylase kinase, delta)	*CALM1*
693	6,09	8,13	-1,34	0,00399	0,308	229685_at	cDNA clone IMAGE:5263734	-
2550	4,95	6,61	-1,34	0,01726	0,370	228826_at	Clone IMAGE:5215917, mRNA	-
1094	332	443	-1,34	0,00641	0,319	223020_at	CLPTM1-like	*CLPTM1L*
264	159	212	-1,33	0,00126	0,260	201174_s_at	Telomeric repeat binding factor 2, interacting protein	*TERF2IP*
1781	41,8	55,8	-1,33	0,01137	0,349	214848_at	Clone 23548 mRNA sequence	-
3290	72,2	96,4	-1,33	0,02344	0,388	228787_s_at	Breast carcinoma amplified sequence 4	*BCAS4*
4960	5,19	6,92	-1,33	0,03805	0,419	1552546_a_at	Leucine zipper-EF-hand containing transmembrane protein 2	*LETM2*
991	4,76	6,35	-1,33	0,00571	0,315	1556301_at	cDNA clone IMAGE:5288145	-
5049	4,87	6,49	-1,33	0,03889	0,420	232195_at	G protein-coupled receptor 158	*GPR158*
2958	15,8	21,1	-1,33	0,02078	0,384	208776_at	Proteasome (prosome, macropain) 26S subunit, non-ATPase, 11	*PSMD11*
2019	7,20	9,59	-1,33	0,01317	0,355	228875_at	Chromosome 6 open reading frame 189	*C6orf189*
1911	62,3	83,0	-1,33	0,01244	0,355	222532_at	Signal recognition particle receptor, B subunit	*SRPRB*
2297	72,8	97,0	-1,33	0,01527	0,363	203219_s_at	Adenine phosphoribosyltransferase	*APRT*
2305	64,6	86,0	-1,33	0,01530	0,363	218607_s_at	SDA1 domain containing 1	*SDAD1*
796	127	170	-1,33	0,00455	0,311	228009_x_at	Zinc ribbon domain containing 1	*ZNRD1*
4362	27,5	36,6	-1,33	0,03245	0,407	225458_at	Hypothetical LOC25845	*LOC25845*
4798	24,0	31,9	-1,33	0,03641	0,415	209556_at	Neurochondrin	*NCDN*
2372	66,5	88,6	-1,33	0,01583	0,364	203271_s_at	Unc-119 homolog (C. elegans)	*UNC119*
3217	86,1	115	-1,33	0,02279	0,387	218919_at	Zinc finger, AN1-type domain 1	*ZFAND1*
5120	131	175	-1,33	0,03941	0,421	225051_at	Erythrocyte membrane protein band 4.1	*EPB41*
2427	63,4	84,4	-1,33	0,01623	0,365	219076_s_at	Peroxisomal membrane protein 2, 22kDa	*PXMP2*
1059	6,17	8,20	-1,33	0,00621	0,319	214536_at	Secreted LY6/PLAUR domain containing 1	*SLURP1*
1351	9,25	12,3	-1,33	0,00812	0,328	210530_s_at	Nuclear receptor subfamily 2, group C, member 1	*NR2C1*
5896	62,6	83,2	-1,33	0,04771	0,442	224368_s_at	NDRG family member 3	*NDRG3*
4889	4,33	5,75	-1,33	0,03738	0,418	242370_at	NADP-dependent methylenetetrahydrofolate dehydrogenase 2-like protein	*MTHFD2L*
4522	23,3	30,9	-1,33	0,03411	0,412	202873_at	ATPase, H+ transporting, lysosomal 42kDa, V1 subunit C1	*ATP6V1C1*
3660	54,2	71,9	-1,33	0,02644	0,395	226149_at	Chromosome 20 open reading frame 198	*C20orf198*
5504	52,5	69,7	-1,33	0,04358	0,433	218284_at	SMAD family member 3	*SMAD3*
2573	17,4	23,0	-1,33	0,01744	0,371	229572_at	Transcribed locus	-
5202	111	148	-1,33	0,04011	0,422	217749_at	Coatomer protein complex, subunit gamma	*COPG*
3779	56,9	75,5	-1,33	0,02751	0,398	213264_at	Poly(rC) binding protein 2	*PCBP2*
6061	25,5	33,8	-1,33	0,04943	0,446	225752_at	Non imprinted in Prader-Willi/Angelman syndrome 1	*NIPA1*

Index	Mutated*	Normal*	FC	*P* value	FDR**	Probe set	Description	Gene symbol
5496	120	159	-1,33	0,04349	0,433	213263_s_at	Poly(rC) binding protein 2	*PCBP2*
661	25,2	33,5	-1,33	0,00382	0,308	208725_at	Eukaryotic translation initiation factor 2, subunit 2 beta, 38kDa	*EIF2S2*
1269	208	275	-1,33	0,00754	0,325	212114_at	Hypothetical LOC552889	*LOC552889*
2534	121	160	-1,33	0,01713	0,369	213892_s_at	Adenine phosphoribosyltransferase	*APRT*
4220	104	138	-1,33	0,03128	0,405	202397_at	Nuclear transport factor 2	*NUTF2*
1793	608	806	-1,33	0,01148	0,350	209066_x_at	Ubiquinol-cytochrome c reductase binding protein	*UQCRB*
2647	19,6	25,9	-1,32	0,01804	0,373	220137_at	Hypothetical protein FLJ20674	*FLJ20674*
1042	24,0	31,8	-1,32	0,00608	0,319	226156_at	v-AKT murine thymoma viral oncogene homolog 2	*AKT2*
3331	7,97	10,6	-1,32	0,02364	0,388	205495_s_at	Granulysin	*GNLY*
3768	36,5	48,3	-1,32	0,02738	0,397	225300_at	Chromosome 15 open reading frame 23	*C15orf23*
1479	10,1	13,3	-1,32	0,00907	0,335	244627_at	Dihydroxyacetone kinase 2 homolog (S. cerevisiae)	*DAK*
4488	167	221	-1,32	0,03376	0,411	202087_s_at	Cathepsin L1	*CTSL1*
3699	27,9	37,0	-1,32	0,02675	0,395	205024_s_at	RAD51 homolog (RecA homolog, E. coli) (S. cerevisiae)	*RAD51*
5104	134	178	-1,32	0,03927	0,421	209448_at	HIV-1 Tat interactive protein 2, 30kDa	*HTATIP2*
3427	26,9	35,6	-1,32	0,02452	0,391	236381_s_at	WD repeat domain 8	*WDR8*
1107	553	731	-1,32	0,00649	0,319	217722_s_at	Neugrin, neurite outgrowth associated	*NGRN*
5824	17,2	22,7	-1,32	0,04706	0,442	211519_s_at	Kinesin family member 2C	*KIF2C*
4705	6,52	8,62	-1,32	0,03558	0,413	1554508_at	Phosphoinositide-3-kinase adaptor protein 1	*PIK3AP1*
1282	55,9	73,8	-1,32	0,00764	0,325	209445_x_at	Chromosome 7 open reading frame 44	*C7orf44*
663	62,2	82,1	-1,32	0,00382	0,308	227008_at	HD domain containing 3	*HDDC3*
5763	48,4	63,9	-1,32	0,04630	0,439	208955_at	dUTP pyrophosphatase	*DUT*
3322	32,7	43,1	-1,32	0,02359	0,388	238032_at	Transcribed locus	-
4022	19,3	25,5	-1,32	0,02945	0,400	1553947_at	Exosome component 6	*EXOSC6*
4991	89,6	118	-1,32	0,03830	0,419	219071_x_at	Chromosome 8 open reading frame 30A	*C8orf30A*
3774	58,3	76,9	-1,32	0,02742	0,397	229192_s_at	Tubulin folding cofactor D	*TBCD*
6073	393	518	-1,32	0,04959	0,446	204427_s_at	Transmembrane emp24 domain trafficking protein 2	*TMED2*
1909	109	144	-1,32	0,01242	0,355	212751_at	Ubiquitin-conjugating enzyme E2N	*UBE2N*
1476	13,6	17,9	-1,32	0,00902	0,334	219154_at	RAS homolog gene family, member F (in filopodia)	*RHOF*
1056	139	183	-1,32	0,00619	0,319	201000_at	Alanyl-tRNA synthetase	*AARS*
2237	15,3	20,2	-1,32	0,01486	0,363	235389_at	PHD finger protein 20	*PHF20*
797	11,7	15,4	-1,32	0,00456	0,311	206273_at	Slowmo homolog 1 (Drosophila)	*SLMO1*
3075	35,0	46,1	-1,32	0,02169	0,385	203217_s_at	ST3 beta-galactoside alpha-2,3-sialyltransferase 5	*ST3GAL5*
2881	12,0	15,8	-1,32	0,02022	0,383	227887_at	Hypothetical LOC400236	*LOC400236*
2203	39,6	52,1	-1,32	0,01457	0,361	1554451_s_at	Cytokine induced protein 29 kDa	*CIP29*
3542	7,13	9,37	-1,31	0,02549	0,393	205920_at	Solute carrier family 6, member 6	*SLC6A6*
2310	3,99	5,25	-1,31	0,01535	0,363	237105_at	cDNA FLJ46701 fis, clone TRACH3014063	-
5337	22,9	30,1	-1,31	0,04178	0,428	238903_at	Hypothetical protein LOC137886	*LOC137886*
2244	9,26	12,2	-1,31	0,01492	0,363	231046_at	cDNA clone IMAGE:4329532	-
3753	85,9	113	-1,31	0,02729	0,397	218549_s_at	Family with sequence similarity 82, member B	*FAM82B*
2232	385	505	-1,31	0,01483	0,363	223209_s_at	Selenoprotein S	*SELS*
5733	17,1	22,5	-1,31	0,04599	0,438	214877_at	CDK5 regulatory subunit associated protein 1-like 1	*CDKAL1*
1766	7,54	9,91	-1,31	0,01126	0,348	237065_s_at	Similar to Hepatitis B virus x-interacting protein	-
2270	7,41	9,74	-1,31	0,01508	0,363	235846_at	cDNA FLJ23692 fis, clone HEP10227	-
908	11,4	14,9	-1,31	0,00524	0,313	244411_at	Similar to Syntaxin binding protein 1	-
1255	6,82	8,94	-1,31	0,00747	0,325	235814_at	Similar to Genethonin 1	-
5035	7,10	9,30	-1,31	0,03878	0,420	219502_at	Nei endonuclease VIII-like 3 (E. coli)	*NEIL3*
3520	141	185	-1,31	0,02527	0,392	217932_at	Mitochondrial ribosomal protein S7	*MRPS7*
4287	152	200	-1,31	0,03185	0,406	221637_s_at	Chromosome 11 open reading frame 48	*C11orf48*
1057	110	144	-1,31	0,00621	0,319	212884_x_at	Apolipoprotein E	*APOE*
5149	46,9	61,4	-1,31	0,03964	0,421	213592_at	Angiotensin II receptor-like 1	*AGTRL1*
4232	52,8	69,2	-1,31	0,03136	0,405	203446_s_at	Oculocerebrorenal syndrome of Lowe	*OCRL*
2128	7,74	10,1	-1,31	0,01400	0,359	214747_at	Zinc finger, BED-type containing 4	*ZBED4*
3953	18,9	24,8	-1,31	0,02888	0,399	227786_at	Mediator complex subunit 30	*MED30*
2512	9,16	12,0	-1,31	0,01690	0,368	206106_at	Mitogen-activated protein kinase 12	*MAPK12*
2435	136	178	-1,31	0,01628	0,365	225480_at	Chromosome 1 open reading frame 122	*C1orf122*
6119	5,44	7,11	-1,31	0,04996	0,446	238369_s_at	Similar to XP_001091806.1	-
3981	6,48	8,48	-1,31	0,02911	0,400	211077_s_at	Tousled-like kinase 1	*TLK1*

Index	Mutated*	Normal*	FC	*P* value	FDR**	Probe set	Description	Gene symbol
4827	3,17	4,14	-1,31	0,03675	0,416	243880_at	Golgi SNAP receptor complex member 2	*GOSR2*
704	9,94	13,0	-1,31	0,00405	0,308	236958_at	cDNA FLJ25271 fis, clone STM05584	-
3511	19,8	25,9	-1,31	0,02522	0,392	228444_at	Transcribed locus	-
3390	39,4	51,4	-1,31	0,02415	0,389	224728_at	ATP synthase mitochondrial F1 complex assembly factor 1	*ATPAF1*
3910	34,0	44,4	-1,31	0,02854	0,399	36564_at	Ring finger protein 19B	*RNF19B*
1976	138	180	-1,31	0,01291	0,355	226679_at	Solute carrier family 26, member 11	*SLC26A11*
2563	99,6	130	-1,31	0,01738	0,371	218151_x_at	G protein-coupled receptor 172A	*GPR172A*
1054	98,4	128	-1,30	0,00616	0,319	203342_at	Translocase of inner mitochondrial membrane 17 homolog B (yeast)	*TIMM17B*
1694	210	274	-1,30	0,01072	0,346	219121_s_at	RNA binding motif protein 35A	*RBM35A*
4559	7,30	9,52	-1,30	0,03445	0,412	203626_s_at	S-phase kinase-associated protein 2 (p45)	*SKP2*
1500	23,9	31,2	-1,30	0,00923	0,336	212707_s_at	RAS p21 protein activator 4	*RASA4*
2442	24,8	32,3	-1,30	0,01632	0,365	1554321_a_at	NFS1 nitrogen fixation 1 homolog (S. cerevisiae)	*NFS1*
3249	58,1	75,7	-1,30	0,02307	0,388	225779_at	Solute carrier family 27, member 4	*SLC27A4*
2535	44,6	58,1	-1,30	0,01714	0,369	204287_at	Synaptogyrin 1	*SYNGR1*
1069	15,8	20,6	-1,30	0,00628	0,319	229577_at	Transcribed locus	-
1570	8,53	11,1	-1,30	0,00976	0,340	204100_at	Thyroid hormone receptor alpha	*THRA*
5853	37,6	49,0	-1,30	0,04734	0,442	1559399_s_at	Zinc finger, CCHC domain containing 10	*ZCCHC10*
1677	6,87	8,95	-1,30	0,01058	0,345	241972_at	Hypothetical LOC401588	*LOC401588*
5577	9,47	12,3	-1,30	0,04447	0,436	205165_at	Cadhetrin, EGF LAG seven-pass G-type receptor 3 (flamingo homolog, Drosop	*CELSR3*
4999	7,12	9,26	-1,30	0,03839	0,420	233057_at	Heat shock 22kDa protein 8	*HSPB8*
5491	33,1	43,0	-1,30	0,04346	0,433	210154_at	Malic enzyme 2, NAD(+)-dependent, mitochondrial	*ME2*
5527	82,6	107	-1,30	0,04382	0,434	212090_at	Glutamate receptor, NMDA subtype, Glutamate-binding subunit	*GRINA*
3801	238	310	-1,30	0,02769	0,398	218206_x_at	SCAN domain containing 1	*SCAND1*
289	356	463	-1,30	0,00142	0,263	201698_s_at	Splicing factor, arginine/serine-rich 9	*SFRS9*
2525	364	474	-1,30	0,01704	0,369	225846_at	RNA binding motif protein 35A	*RBM35A*
5061	64,2	83,5	-1,30	0,03896	0,420	202069_s_at	Isocitrate dehydrogenase 3 (NAD+) alpha	*IDH3A*
4837	12,3	16,0	-1,30	0,03688	0,417	206612_at	Calcium channel, voltage-dependent, gamma subunit 1	*CACNG1*
893	10,2	13,3	-1,30	0,00517	0,313	239941_at	Similar to Mitochondrial ribosomal protein S23	-
5909	106	137	-1,30	0,04789	0,443	222500_at	Peptidylprolyl isomerase (cyclophilin)-like 1	*PPIL1*
3387	47,5	61,6	-1,30	0,02413	0,389	1552279_a_at	Solute carrier family 46, member 1	*SLC46A1*
4690	7,01	9,11	-1,30	0,03543	0,413	236312_at	Transcribed locus	-
1565	16,1	20,9	-1,30	0,00972	0,339	210240_s_at	Cyclin-dependent kinase inhibitor 2D	*CDKN2D*
4207	180	234	-1,30	0,03110	0,404	201212_at	Legumain	*LGMN*
4316	243	315	-1,30	0,03214	0,407	218482_at	Enhancer of yellow 2 homolog (Drosophila)	*ENY2*
1150	3,98	5,16	-1,30	0,00677	0,321	1557448_a_at	cDNA clone IMAGE:5298376	-
2710	22,3	28,9	-1,30	0,01860	0,375	205733_at	Bloom syndrome	*BLM*
2258	40,5	52,5	-1,30	0,01502	0,363	234972_at	ADP-ribosylation factor-like 16	*ARL16*
2196	6,81	8,84	-1,30	0,01452	0,361	230932_at	Transcribed locus	-
4679	128	166	-1,30	0,03533	0,413	222998_at	MAF1 homolog (S. cerevisiae)	*MAF1*
3831	24,5	31,8	-1,30	0,02798	0,399	238472_at	F-box protein 9	*FBXO9*
2539	25,6	33,2	-1,30	0,01717	0,369	205432_at	Oviductal glycoprotein 1, 120kDa (mucin 9, oviductin)	*OVGP1*
4138	33,7	43,7	-1,30	0,03052	0,403	204368_at	Solute carrier organic anion transporter family, member 2A1	*SLCO2A1*
824	204	265	-1,30	0,00470	0,312	217898_at	Chromosome 15 open reading frame 24	*C15orf24*
2422	118	153	-1,30	0,01620	0,365	221597_s_at	HSPC171 protein	*HSPC171*
4415	139	180	-1,30	0,03301	0,409	232652_x_at	SCAN domain containing 1	*SCAND1*
3135	19,5	25,2	-1,30	0,02222	0,387	221837_at	Kelch-like 22 (Drosophila)	*KLHL22*
5919	62,2	80,6	-1,30	0,04798	0,443	204278_s_at	Estrogen receptor binding site associated, antigen, 9	*EBAG9*
1257	36,2	46,8	-1,29	0,00749	0,325	204970_s_at	Basic leucine zipper transcription factor MafG	*MAFG*
1825	93,6	121	-1,29	0,01176	0,351	223107_s_at	Zinc finger, CCHC domain containing 17	*ZCCHC17*
3397	127	164	-1,29	0,02424	0,390	206055_s_at	Small nuclear ribonucleoprotein polypeptide A'	*SNRPA1*
3015	19,1	24,7	-1,29	0,02122	0,385	218727_at	Amino acid transporter	*FLJ10815*
694	46,4	60,1	-1,29	0,00400	0,308	225067_at	Unc-51-like kinase 3 (C. elegans)	*ULK3*
1375	20,1	26,0	-1,29	0,00829	0,329	226687_at	PRP40 pre-mRNA processing factor 40 homolog A (S. cerevisiae)	*PRPF40A*
2271	89,2	115	-1,29	0,01508	0,363	204565_at	Thioesterase superfamily member 2	*THEM2*
2843	29,1	37,7	-1,29	0,01988	0,382	35846_at	Thyroid hormone receptor alpha	*THRA*
2996	26,0	33,5	-1,29	0,02110	0,385	44673_at	Sialic acid binding Ig-like lectin 1, sialoadhesin	*SIGLEC1*
9	28,9	37,3	-1,29	0,00002	0,117	218415_at	Vacuolar protein sorting 33 homolog B (yeast)	*VPS33B*

Index	Mutated*	Normal*	FC	*P* value	FDR**	Probe set	Description	Gene symbol
365	5,97	7,71	-1,29	0,00190	0,284	239848_at	cDNA FLJ41316 fis, clone BRAMY2043314	-
5956	17,1	22,0	-1,29	0,04832	0,444	204887_s_at	Polo-like kinase 4 (Drosophila)	*PLK4*
4105	28,1	36,3	-1,29	0,03017	0,402	220178_at	Chromosome 19 open reading frame 28	*C19orf28*
5961	69,6	89,8	-1,29	0,04839	0,444	227527_at	Myeloid/lymphoid or mixed-lineage leukemia 2	*MLL2*
3147	629	812	-1,29	0,02234	0,387	205849_s_at	Ubiquinol-cytochrome c reductase binding protein	*UQCRB*
2246	12,5	16,1	-1,29	0,01493	0,363	228894_at	Nuclear receptor subfamily 6, group A, member 1	*NR6A1*
5296	49,6	63,9	-1,29	0,04127	0,426	205089_at	Zinc finger protein 7	*ZNF7*
5070	38,3	49,4	-1,29	0,03901	0,420	213704_at	RAB geranylgeranyltransferase, beta subunit	*RABGGTB*
4739	24,6	31,7	-1,29	0,03588	0,414	204649_at	Trophinin associated protein (tastin)	*TROAP*
5886	68,6	88,5	-1,29	0,04762	0,442	210010_s_at	Solute carrier family 25, member 1	*SLC25A1*
3130	33,0	42,5	-1,29	0,02221	0,387	203777_s_at	Ribosomal protein S6 kinase, 70kDa, polypeptide 2	*RPS6KB2*
4906	37,1	47,7	-1,29	0,03751	0,418	229453_at	Protein disulfide isomerase family A, member 3	*PDIA3*
3729	9,47	12,2	-1,29	0,02708	0,397	228828_at	Clone IMAGE:5215917, mRNA	-
4988	54,8	70,5	-1,29	0,03828	0,419	91684_g_at	Exosome component 4	*EXOSC4*
61	11,3	14,5	-1,29	0,00023	0,201	227201_at	Hypothetical protein LOC643837	*LOC643837*
1364	162	208	-1,29	0,00821	0,328	217957_at	Chromosome 16 open reading frame 80	*C16orf80*
4586	34,0	43,8	-1,29	0,03467	0,412	242790_at	SNF8, ESCRT-II complex subunit, homolog (S. cerevisiae)	*SNF8*
3716	4,16	5,36	-1,29	0,02694	0,396	215943_at	KIAA1661 protein	*KIAA1661*
4240	35,5	45,8	-1,29	0,03145	0,406	1552612_at	CDC42 small effector 2	*CDC42SE2*
1704	45,9	59,0	-1,29	0,01087	0,348	208316_s_at	Oculocerebrorenal syndrome of Lowe	*OCRL*
2148	24,6	31,6	-1,29	0,01414	0,360	217998_at	Pleckstrin homology-like domain, family A, member 1	*PHLDA1*
990	490	630	-1,29	0,00571	0,315	217962_at	Nucleolar protein family A, member 3	*NOLA3*
2686	49,9	64,1	-1,29	0,01840	0,375	215722_s_at	Small nuclear ribonucleoprotein polypeptide A'	*SNRPA1*
5308	40,3	51,7	-1,29	0,04141	0,426	225901_at	Protein tyrosine phosphatase, mitochondrial 1	*PTPMT1*
1359	10,2	13,1	-1,28	0,00819	0,328	235574_at	Guanylate binding protein 4	*GBP4*
5373	14,8	19,0	-1,28	0,04216	0,429	235625_at	Vacuolar protein sorting 41 homolog (S. cerevisiae)	*VPS41*
3092	22,2	28,6	-1,28	0,02180	0,385	223811_s_at	Chromosome 7 open reading frame 20	*C7orf20*
3966	21,3	27,4	-1,28	0,02897	0,399	216698_x_at	Olfactory receptor, family 7, subfamily E, member 47 pseudogene	*OR7E47P*
5014	29,1	37,4	-1,28	0,03856	0,420	214081_at	Plexin domain containing 1	*PLXDC1*
5941	9,56	12,3	-1,28	0,04816	0,443	207043_s_at	Solute carrier family 6, member 9	*SLC6A9*
3627	18,0	23,1	-1,28	0,02621	0,395	207458_at	Chromosome 8 open reading frame 51	*C8orf51*
3297	244	313	-1,28	0,02349	0,388	217748_at	Adiponectin receptor 1	*ADIPOR1*
161	13,0	16,7	-1,28	0,00073	0,244	238087_at	RNA terminal phosphate cyclase domain 1	*RTCD1*
3024	12,1	15,5	-1,28	0,02129	0,385	220491_at	Hepcidin antimicrobial peptide	*HAMP*
3692	6,42	8,24	-1,28	0,02666	0,395	237369_at	Transcribed locus	-
2429	40,3	51,7	-1,28	0,01624	0,365	203320_at	SH2B adaptor protein 3	*SH2B3*
1437	13,6	17,4	-1,28	0,00870	0,331	211434_s_at	Chemokine (C-C motif) receptor-like 2	*CCRL2*
4548	114	146	-1,28	0,03438	0,412	223120_at	Fucosidase, alpha-L- 2, plasma	*FUCA2*
5167	46,5	59,6	-1,28	0,03982	0,421	229232_at	Leucine rich repeat containing 57	*LRRC57*
2419	51,2	65,6	-1,28	0,01617	0,365	227456_s_at	Chromosome 6 open reading frame 136	*C6orf136*
5044	7,31	9,37	-1,28	0,03886	0,420	205581_s_at	Nitric oxide synthase 3 (endothelial cell)	*NOS3*
3287	31,0	39,8	-1,28	0,02342	0,388	204819_at	FYVE, RhoGEF and PH domain containing 1	*FGD1*
5416	404	518	-1,28	0,04256	0,430	202306_at	Polymerase (RNA) II (DNA directed) polypeptide G	*POLR2G*
5692	109	139	-1,28	0,04553	0,437	202541_at	Small inducible cytokine subfamily E, member 1	*SCYE1*
1343	15,5	19,9	-1,28	0,00806	0,328	238507_at	Transcribed locus	-
4423	95,3	122	-1,28	0,03311	0,409	224742_at	Abhydrolase domain containing 12	*ABHD12*
3057	7,37	9,44	-1,28	0,02153	0,385	239493_at	Ribosomal protein L7	*RPL7*
4503	246	315	-1,28	0,03389	0,411	212250_at	Metadherin	*MTDH*
1304	62,6	80,1	-1,28	0,00777	0,325	212434_at	GrpE-like 1, mitochondrial (E. coli)	*GRPEL1*
2374	226	290	-1,28	0,01583	0,364	224957_at	Chromosome 18 open reading frame 32	*C18orf32*
355	67,1	85,8	-1,28	0,00182	0,280	1553987_at	Chromosome 12 open reading frame 47	*C12orf47*
5508	5,47	7,00	-1,28	0,04364	0,433	221195_at	PTD016 protein	*LOC51136*
3452	11,1	14,2	-1,28	0,02471	0,391	206655_s_at	Glycoprotein Ib (platelet), beta polypeptide	*GP1BB*
4573	3,36	4,30	-1,28	0,03457	0,412	220022_at	Zinc finger protein 334	*ZNF334*
4291	5,92	7,56	-1,28	0,03190	0,406	238427_at	GrpE-like 2, mitochondrial (E. coli)	*GRPEL2*
5725	135	172	-1,28	0,04591	0,438	209397_at	Malic enzyme 2, NAD(+)-dependent, mitochondrial	*ME2*
1863	54,8	70,1	-1,28	0,01203	0,353	224804_s_at	Chromosome 15 open reading frame 17	*C15orf17*

Index	Mutated*	Normal*	FC	*P* value	FDR**	Probe set	Description	Gene symbol
424	15,3	19,5	-1,28	0,00223	0,288	209552_at	Paired box 8	*PAX8*
2303	70,9	90,4	-1,28	0,01530	0,363	1558136_s_at	Transcription initiation factor TFIID 28 kD subunit	*TAF11*
4450	16,4	21,0	-1,27	0,03343	0,411	232186_at	Chromosome 20 open reading frame 142	*C20orf142*
2212	19,1	24,3	-1,27	0,01466	0,362	220200_s_at	SET domain containing (lysine methyltransferase) 8	*SETD8*
2649	26,4	33,6	-1,27	0,01806	0,373	228566_at	Hypothetical protein FLJ10656	*P15RS*
5242	8,23	10,5	-1,27	0,04060	0,423	232422_at	Hypothetical protein BC004360	-
2971	14,0	17,8	-1,27	0,02087	0,384	220291_at	Glycerophosphodiester phosphodiesterase domain containing 2	*GDPD2*
4043	3,66	4,65	-1,27	0,02964	0,401	210447_at	Glutamate dehydrogenase 2	*GLUD2*
5593	8,17	10,4	-1,27	0,04461	0,436	205568_at	Aquaporin 9	*AQP9*
1234	7,73	9,83	-1,27	0,00734	0,324	208511_at	Pituitary tumor-transforming 3	*PTTG3*
2295	42,0	53,4	-1,27	0,01527	0,363	227093_at	Ubiquitin specific peptidase 36	*USP36*
4543	8,49	10,8	-1,27	0,03434	0,412	37145_at	Granulysin	*GNLY*
4055	183	232	-1,27	0,02975	0,401	231059_x_at	SCAN domain containing 1	*SCAND1*
3685	8,14	10,3	-1,27	0,02661	0,395	1552737_s_at	WW domain containing E3 ubiquitin protein ligase 2	*WWP2*
2803	6,08	7,72	-1,27	0,01949	0,380	210360_s_at	Metastasis suppressor 1	*MTSS1*
847	86,4	110	-1,27	0,00488	0,313	225208_s_at	Family with sequence similarity 103, member A1	*FAM103A1*
4469	78,6	99,8	-1,27	0,03357	0,411	225592_at	Nurim (nuclear envelope membrane protein)	*NRM*
598	31,8	40,3	-1,27	0,00329	0,301	225212_at	Solute carrier family 25, member 25	*SLC25A25*
4272	9,65	12,2	-1,27	0,03175	0,406	223570_at	Minichromosome maintenance complex component 10	*MCM10*
1021	54,0	68,4	-1,27	0,00591	0,316	1554553_s_at	Yip1 interacting factor homolog B (S. cerevisiae)	*YIF1B*
683	30,3	38,4	-1,27	0,00392	0,308	209777_s_at	Solute carrier family 19, member 1	*SLC19A1*
5174	59,4	75,3	-1,27	0,03985	0,421	225637_at	Differentially expressed in FDCP 8 homolog (mouse)	*DEF8*
2939	42,7	54,2	-1,27	0,02062	0,383	225699_at	Chromosome 7 open reading frame 40	*C7orf40*
5338	19,9	25,2	-1,27	0,04179	0,428	31799_at	Clone 24627 mRNA sequence	-
4259	43,9	55,6	-1,27	0,03166	0,406	202104_s_at	Spastic paraplegia 7 protein	*SPG7*
4590	122	155	-1,27	0,03472	0,412	212228_s_at	Coenzyme Q9 homolog (S. cerevisiae)	*COQ9*
2290	24,6	31,1	-1,27	0,01523	0,363	211320_s_at	Protein tyrosine phosphatase, receptor type, U	*PTPRU*
510	108	137	-1,27	0,00270	0,289	225434_at	Death effector domain containing 2	*DEDD2*
3885	7,66	9,70	-1,27	0,02837	0,399	1556227_at	Valosin containing protein (p97)/p47 complex interacting protein 1	*VCPIP1*
6012	8,25	10,4	-1,27	0,04888	0,445	239652_at	Transcribed locus	-
1478	28,3	35,8	-1,27	0,00905	0,335	227042_at	LOC150223 protein	*LOC150223*
2234	15,4	19,5	-1,26	0,01484	0,363	230039_at	-	-
1072	23,2	29,4	-1,26	0,00628	0,319	1562228_s_at	Phosphodiesterase 5A, cGMP-specific	*PDE5A*
2365	316	399	-1,26	0,01579	0,364	211984_at	Calmodulin 1 (phosphorylase kinase, delta)	*CALM1*
1490	16,6	21,0	-1,26	0,00919	0,336	205256_at	Zinc finger and BTB domain containing 39	*ZBTB39*
4769	4,96	6,27	-1,26	0,03614	0,414	206925_at	ST8 alpha-N-acetyl-neuraminide alpha-2,8-sialyltransferase 4	*ST8SIA4*
3795	43,1	54,5	-1,26	0,02765	0,398	218813_s_at	SH3-domain GRB2-like endophilin B2	*SH3GLB2*
4628	6,73	8,50	-1,26	0,03490	0,412	1559051_s_at	Chromosome 6 open reading frame 150	*C6orf150*
2388	92,3	117	-1,26	0,01592	0,364	217543_s_at	Membrane-bound transcription factor peptidase, site 1	*MBTPS1*
703	37,7	47,6	-1,26	0,00403	0,308	56821_at	Amino acid transporter	*FLJ10815*
4684	94,5	119	-1,26	0,03536	0,413	221736_at	KIAA1219 protein	*KIAA1219*
4401	8,59	10,8	-1,26	0,03281	0,408	210786_s_at	Friend leukemia virus integration 1	*FLI1*
1957	29,7	37,5	-1,26	0,01279	0,355	227103_s_at	Endothelin converting enzyme 2	*ECE2*
5045	40,2	50,6	-1,26	0,03888	0,420	218821_at	Aminopeptidase-like 1	*NPEPL1*
2282	202	255	-1,26	0,01516	0,363	224748_at	WD repeat domain 68	*WDR68*
2098	16,6	21,0	-1,26	0,01375	0,358	229570_at	Laminin, alpha 5	*LAMA5*
764	39,2	49,5	-1,26	0,00432	0,309	224907_s_at	SH3-domain GRB2-like endophilin B2	*SH3GLB2*
167	16,9	21,3	-1,26	0,00078	0,244	224433_s_at	DEAD (Asp-Glu-Ala-Asp) box polypeptide 54	*DDX54*
43	93,3	118	-1,26	0,00018	0,201	221777_at	Chromosome 12 open reading frame 52	*C12orf52*
1259	106	134	-1,26	0,00750	0,325	208996_s_at	Polymerase (RNA) II (DNA directed) polypeptide C, 33kDa	*POLR2C*
918	16,0	20,1	-1,26	0,00529	0,313	213449_at	Ribonucleases P/MRP protein subunit POP1	*POP1*
2763	146	184	-1,26	0,01920	0,379	1555780_a_at	RAS homolog enriched in brain	*RHEB*
3097	71,7	90,2	-1,26	0,02187	0,386	223639_s_at	Zinc ribbon domain containing 1	*ZNRD1*
2060	68,3	85,9	-1,26	0,01344	0,356	218592_s_at	Cat eye syndrome chromosome region, candidate 5	*CECR5*
423	10,1	12,7	-1,26	0,00222	0,287	227358_at	Zinc finger and BTB domain containing 46	*ZBTB46*
4100	44,8	56,4	-1,26	0,03013	0,402	225704_at	KIAA1545 protein	*KIAA1545*
2044	5,20	6,54	-1,26	0,01329	0,355	232794_at	Hypothetical protein LOC153682	*LOC153682*

Index	Mutated*	Normal*	FC	*P* value	FDR**	Probe set	Description	Gene symbol
5506	40,8	51,4	-1,26	0,04361	0,433	208424_s_at	Cytokine induced apoptosis inhibitor 1	*CIAPIN1*
3364	58,0	72,9	-1,26	0,02390	0,388	64064_at	GTPase, IMAP family member 5	*GIMAP5*
187	11,5	14,5	-1,26	0,00087	0,244	236537_at	cDNA FLJ23896 fis, clone LNG15157	-
4932	46,6	58,6	-1,26	0,03782	0,419	202974_at	Membrane protein, palmitoylated 1, 55kDa	*MPP1*
5746	38,0	47,8	-1,26	0,04609	0,439	226793_at	Hypothetical protein LOC283267	*LOC283267*
5908	19,8	24,9	-1,26	0,04789	0,443	209052_s_at	Wolf-Hirschhorn syndrome candidate 1	*WHSC1*
5897	10,4	13,1	-1,26	0,04772	0,442	219742_at	Proline rich 7 (synaptic)	*PRR7*
1887	14,9	18,7	-1,26	0,01225	0,355	230974_at	DEAD (Asp-Glu-Ala-As) box polypeptide 19B	*DDX19B*
4696	870	1092	-1,26	0,03547	0,413	214359_s_at	Heat shock protein 90kDa alpha (cytosolic), class B member 1	*HSP90AB1*
4832	28,2	35,4	-1,26	0,03681	0,416	219491_at	Leucine rich repeat and fibronectin type III domain containing 4	*LRFN4*
298	11,2	14,1	-1,26	0,00145	0,263	230025_at	Gap junction protein, chi 1, 31.9kDa	*GJC1*
5111	43,1	54,1	-1,26	0,03934	0,421	221011_s_at	Limb bud and heart development homolog (mouse)	*LBH*
5271	51,7	64,8	-1,25	0,04096	0,425	219394_at	PhosphatidylglyceroPhosphate synthase 1	*PGS1*
173	6,22	7,81	-1,25	0,00080	0,244	1569452_at	Hypothetical locus LOC692247	*LOC692247*
1325	28,9	36,2	-1,25	0,00795	0,328	213861_s_at	Family with sequence similarity 119, member B	*FAM119B*
3786	30,2	37,9	-1,25	0,02758	0,398	227818_at	Coiled-coil domain containing 21	*CCDC21*
1380	11,6	14,5	-1,25	0,00830	0,329	232959_at	Similar to Septin 7	*LOC645513*
1356	25,1	31,4	-1,25	0,00817	0,328	228870_at	Family with sequence similarity 84, member B	*FAM84B*
2378	87,7	110	-1,25	0,01586	0,364	210153_s_at	Malic enzyme 2, NAD(+)-dependent, mitochondrial	*ME2*
3383	4,70	5,89	-1,25	0,02409	0,389	222881_at	Heparanase	*HPSE*
3472	11,0	13,8	-1,25	0,02491	0,392	206617_s_at	Renin binding protein	*RENBP*
5843	13,6	17,0	-1,25	0,04722	0,442	1553971_a_at	Opposite strand transcription unit to STAG3	*GATS*
2820	110	138	-1,25	0,01969	0,381	208848_at	Alcohol dehydrogenase 5 (class III), chi polypeptide	*ADH5*
3013	43,7	54,8	-1,25	0,02119	0,385	226063_at	VAV2 guanine nucleotide exchange factor	*VAV2*
252	5,30	6,65	-1,25	0,00117	0,254	236916_at	Serine/threonine kinase receptor associated protein	*STRAP*
2750	104	130	-1,25	0,01901	0,378	208785_s_at	Microtubule-associated protein 1 light chain 3 beta	*MAP1LC3B*
4969	35,6	44,6	-1,25	0,03812	0,419	230606_at	Gap junction protein, chi 1, 31.9kDa	*GJC1*
1991	1572	1969	-1,25	0,01302	0,355	200064_at	Heat shock protein 90kDa alpha (cytosolic), class B member 1	*HSP90AB1*
5772	7,65	9,58	-1,25	0,04639	0,439	210606_x_at	Killer cell lectin-like receptor subfamily D, member 1	*KLRD1*
5711	401	502	-1,25	0,04573	0,438	202836_s_at	Thioredoxin-like 4A	*TXNL4A*
1950	222	277	-1,25	0,01273	0,355	202483_s_at	RAN binding protein 1	*RANBP1*
2021	10,5	13,1	-1,25	0,01317	0,355	236200_at	Fas (TNFRSF6) associated factor 1	*FAF1*
4809	108	135	-1,25	0,03652	0,415	208968_s_at	Cytokine induced apoptosis inhibitor 1	*CIAPIN1*
219	45,3	56,7	-1,25	0,00100	0,250	218660_at	Dysferlin, limb girdle muscular dystrophy 2B	*DYSF*
4336	3,47	4,34	-1,25	0,03227	0,407	1559072_a_at	Leucine rich repeat containing 62	*LRRC62*
168	7,28	9,10	-1,25	0,00078	0,244	231777_at	Casein kinase 2, beta polypeptide	*CSNK2B*
5509	4,15	5,19	-1,25	0,04365	0,433	210146_x_at	Leukocyte immunoglobulin-like receptor, subfamily B member 2	*LILRB2*
4796	58,9	73,6	-1,25	0,03638	0,415	219952_s_at	Mucolipin 1	*MCOLN1*
5335	9,83	12,3	-1,25	0,04177	0,428	214775_at	Nedd4 binding protein 3	*N4BP3*
5997	4,69	5,85	-1,25	0,04875	0,444	210567_s_at	S-phase kinase-associated protein 2 (p45)	*SKP2*
1940	13,5	16,9	-1,25	0,01264	0,355	205396_at	SMAD family member 3	*SMAD3*
1755	8,07	10,1	-1,25	0,01118	0,348	214530_x_at	Erythrocyte membrane protein band 4.1	*EPB41*
4114	11,1	13,9	-1,25	0,03025	0,402	230271_at	One cut homeobox 2	*ONECUT2*
2628	34,1	42,6	-1,25	0,01790	0,372	213324_at	v-src sarcoma (Schmidt-Ruppin A-2) viral oncogene homolog (avian)	*SRC*
4095	4,00	5,00	-1,25	0,03009	0,402	208388_at	Nuclear receptor subfamily 2, group E, member 3	*NR2E3*
2233	18,1	22,6	-1,25	0,01484	0,363	219760_at	Lin-7 homolog B (C. elegans)	*LIN7B*
4468	28,5	35,6	-1,25	0,03357	0,411	229535_at	Protein tyrosine phosphatase, mitochondrial 1	*PTPMT1*
1663	9,06	11,3	-1,25	0,01050	0,345	231110_at	Transcribed locus	-
2471	33,6	41,9	-1,25	0,01660	0,367	219202_at	Rhomboid 5 homolog 2 (Drosophila)	*RHBDF2*
4496	13,4	16,8	-1,25	0,03382	0,411	215285_s_at	Putative homeodomain transcription factor 1	*PHTF1*
755	19,3	24,0	-1,25	0,00427	0,308	228364_at	Zinc finger protein 784	*ZNF784*
3211	52,0	64,8	-1,25	0,02274	0,387	209680_s_at	Kinesin family member C1	*KIFC1*
2459	6,03	7,52	-1,25	0,01651	0,367	218851_s_at	WD repeat domain 33	*WDR33*
993	52,0	64,8	-1,25	0,00572	0,315	38157_at	Dom-3 homolog Z (C. elegans)	*DOM3Z*
5988	8,38	10,4	-1,25	0,04865	0,444	1557667_at	cDNA FLJ36588 fis, clone TRACH2013991	-
740	12,9	16,1	-1,25	0,00420	0,308	219194_at	Semaphorin-4G	*SEMA4G*
2658	10,5	13,1	-1,25	0,01813	0,373	238572_at	Hypothetical protein MGC16211	*MGC16211*

Index	Mutated*	Normal*	FC	*P* value	FDR**	Probe set	Description	Gene symbol
3723	10,3	12,8	-1,24	0,02702	0,397	240297_at	Transcribed locus	-
1135	60,1	74,8	-1,24	0,00664	0,319	223954_x_at	Neuronal calcium-binding protein NECAB3	*APBA2BP*
359	73,6	91,6	-1,24	0,00184	0,281	203536_s_at	Cytosolic iron-sulfur protein assembly 1 homolog (S. cerevisiae)	*CIAO1*
33	8,19	10,2	-1,24	0,00015	0,201	227057_at	Rho GTPase activating protein 27	*ARHGAP27*
3727	253	315	-1,24	0,02706	0,397	212248_at	cDNA FLJ41088 fis, clone ASTRO2002459 (Metadherin)	*MTDH*
363	4,49	5,59	-1,24	0,00190	0,284	239184_at	Transcribed locus	-
3674	32,4	40,3	-1,24	0,02654	0,395	214955_at	Transmembrane protease, serine 6	*TMPRSS6*
3312	137	171	-1,24	0,02355	0,388	223108_s_at	Zinc finger, CCHC domain containing 17	*ZCCHC17*
5275	6,07	7,56	-1,24	0,04099	0,425	219264_s_at	Protein phosphatase 2A 48 kDa regulatory subunit	*PPP2R3B*
1184	32,0	39,8	-1,24	0,00697	0,322	216397_s_at	Block of proliferation 1	*BOP1*
1188	20,1	25,0	-1,24	0,00700	0,322	204974_at	RAB3A, member RAS oncogene family	*RAB3A*
2348	22,9	28,4	-1,24	0,01566	0,364	213520_at	RecQ protein-like 4	*RECQL4*
4474	43,0	53,5	-1,24	0,03364	0,411	207845_s_at	Anaphase promoting complex subunit 10	*ANAPC10*
309	29,9	37,1	-1,24	0,00149	0,264	217199_s_at	Signal transducer and activator of transcription 2	*STAT2*
5726	9,13	11,3	-1,24	0,04591	0,438	1558369_at	M-phase phosphoprotein 9	*MPHOSPH9*
4650	48,2	59,9	-1,24	0,03508	0,413	219214_s_at	5', 3'-nucleotidase, cytosolic	*NT5C*
4270	140	174	-1,24	0,03175	0,406	223649_s_at	Solute carrier family 25, member 39	*SLC25A39*
2661	9,47	11,8	-1,24	0,01817	0,373	229892_at	EP400 N-terminal like	*EP400NL*
3433	21,0	26,1	-1,24	0,02457	0,391	336_at	Thromboxane A2 receptor	*TBXA2R*
180	23,4	29,0	-1,24	0,00084	0,244	238628_s_at	Trafficking protein particle complex 2-like	*TRAPPC2L*
2453	65,0	80,7	-1,24	0,01645	0,366	219953_s_at	Chromosome 11 open reading frame 17	*C11orf17*
1575	6,61	8,20	-1,24	0,00979	0,340	242308_at	Mucolipin 3	*MCOLN3*
2942	40,0	49,6	-1,24	0,02062	0,383	212528_at	cDNA clone IMAGE:3878236	-
3937	586	726	-1,24	0,02876	0,399	200750_s_at	RAN, member RAS oncogene family	*RAN*
2002	10,1	12,5	-1,24	0,01307	0,355	230616_at	Similar to Laminin, beta 2 (laminin S) isoform 2	-
3260	236	292	-1,24	0,02314	0,388	202109_at	ADP-ribosylation factor interacting protein 2 (arfaptin 2)	*ARFIP2*
1650	9,55	11,8	-1,24	0,01038	0,344	238146_at	Transcribed locus	-
3000	29,3	36,2	-1,24	0,02112	0,385	232675_s_at	Uridine-cytidine kinase 1-like 1	*UCKL1*
629	28,4	35,2	-1,24	0,00356	0,308	225577_at	HLA complex group 18	*HCG18*
89	63,1	78,1	-1,24	0,00033	0,201	121_at	Paired box 8	*PAX8*
3762	7,93	9,82	-1,24	0,02735	0,397	1558123_at	Hypothetical protein FLJ35390	*FLJ35390*
1719	7,21	8,92	-1,24	0,01101	0,348	1554677_s_at	CKLF-like MARVEL transmembrane domain containing 4	*CMTM4*
2163	25,7	31,8	-1,24	0,01431	0,361	229031_at	cDNA FLJ25763 fis, clone TST06294	-
3280	7,52	9,30	-1,24	0,02336	0,388	238425_at	Transcribed locus	-
899	306	379	-1,24	0,00521	0,313	202579_x_at	High mobility group nucleosomal binding domain 4	*HMGN4*
4971	37,8	46,8	-1,24	0,03813	0,419	231808_at	Keratin associated protein 4-7	*KRTAP4-7*
1944	7,97	9,85	-1,24	0,01267	0,355	228747_at	Sec61 alpha 2 subunit (S. cerevisiae)	*SEC61A2*
3868	10,9	13,5	-1,24	0,02825	0,399	222748_s_at	Thioredoxin-like 4B	*TXNL4B*
2944	5,39	6,66	-1,24	0,02064	0,383	234211_at	cDNA FLJ21436 fis, clone COL04279	-
1828	15,8	19,5	-1,24	0,01178	0,351	229426_at	Cytochrome c oxidase subunit Va	*COX5A*
1287	7,89	9,75	-1,24	0,00765	0,325	238798_at	Transmembrane anterior posterior transformation 1	*TAPT1*
1103	115	142	-1,23	0,00646	0,319	207396_s_at	Asparagine-linked glycosylation 3 homolog	*ALG3*
2141	24,0	29,7	-1,23	0,01408	0,360	209495_at	centrosomal protein 250kDa	*CEP250*
5322	29,2	36,0	-1,23	0,04161	0,427	204857_at	MAD1 mitotic arrest deficient-like 1 (yeast)	*MAD1L1*
3842	89,7	111	-1,23	0,02805	0,399	218836_at	Ribonuclease P/MRP 21kDa subunit	*RPP21*
261	5,81	7,17	-1,23	0,00123	0,257	239856_at	Similar to XP_001175123.1	-
2808	7,16	8,83	-1,23	0,01954	0,380	1560577_at	cDNA clone IMAGE:4827370	-
2765	64,5	79,5	-1,23	0,01920	0,379	226437_at	Yip1 interacting factor homolog B (S. cerevisiae)	*YIF1B*
3436	6,44	7,94	-1,23	0,02459	0,391	1556039_s_at	G protein-coupled receptor 173	*GPR173*
490	18,6	22,9	-1,23	0,00262	0,289	202835_at	Thioredoxin-like 4A	*TXNL4A*
3984	212	262	-1,23	0,02914	0,400	201520_s_at	G-rich RNA sequence binding factor 1	*GRSF1*
1228	34,9	43,0	-1,23	0,00730	0,324	203402_at	Potassium channel shaker chain beta 2	*KCNAB2*
4254	5,96	7,34	-1,23	0,03158	0,406	1556545_at	cDNA FLJ32379 fis, clone SKMUS1000030	-
2495	13,1	16,1	-1,23	0,01682	0,367	213669_at	FCH domain only 1	*FCHO1*
1885	34,7	42,8	-1,23	0,01224	0,355	223222_at	Solute carrier family 25, member 19	*SLC25A19*
4685	35,9	44,3	-1,23	0,03537	0,413	209833_at	CASP2 and RIPK1 domain containing adaptor with death domain	*CRADD*
5652	19,4	23,9	-1,23	0,04509	0,436	208603_s_at	Mitogen-activated protein kinase 8 interacting protein 2	*MAPK8IP2*

Index	Mutated*	Normal*	FC	*P* value	FDR**	Probe set	Description	Gene symbol
414	8,71	10,7	-1,23	0,00218	0,286	1569428_at	Within bgcn homolog (Drosophila)	*WIBG*
1757	6,18	7,61	-1,23	0,01120	0,348	203578_s_at	Solute carrier family 7, member 6	*SLC7A6*
45	12,4	15,3	-1,23	0,00018	0,201	211888_x_at	Caspase 10, apoptosis-related cysteine peptidase	*CASP10*
2782	66,8	82,3	-1,23	0,01933	0,380	225593_at	LSM10, U7 small nuclear RNA associated	*LSM10*
552	24,5	30,2	-1,23	0,00299	0,296	235663_at	-	-
5526	27,5	33,8	-1,23	0,04381	0,433	224798_s_at	Chromosome 15 open reading frame 17	*C15orf17*
1317	57,2	70,4	-1,23	0,00784	0,325	212133_at	Non imprinted in Prader-Willi/Angelman syndrome 2	*NIPA2*
3904	18,1	22,3	-1,23	0,02851	0,399	224357_s_at	Membrane-spanning 4-domains, subfamily A, member 4	*MS4A4A*
4160	7,79	9,59	-1,23	0,03076	0,404	1554063_at	Chromosome 8 open reading frame 76	*C8orf76*
3654	28,3	34,8	-1,23	0,02641	0,395	232025_at	Synaptotagmin VII	*SYT7*
4020	62,6	77,1	-1,23	0,02943	0,400	200861_at	CCR4-NOT transcription complex, subunit 1	*CNOT1*
996	128	158	-1,23	0,00575	0,316	208722_s_at	Anaphase promoting complex subunit 5	*ANAPC5*
296	57,3	70,5	-1,23	0,00145	0,263	44111_at	Vacuolar protein sorting 33 homolog B (yeast)	*VPS33B*
5417	3,58	4,40	-1,23	0,04256	0,430	231797_at	SIX homeobox 4	*SIX4*
2597	42,6	52,3	-1,23	0,01765	0,371	233168_s_at	Selenoprotein O	*SELO*
3416	58,0	71,2	-1,23	0,02439	0,390	221629_x_at	Chromosome 8 open reading frame 30A	*C8orf30A*
4286	10,1	12,4	-1,23	0,03185	0,406	227055_at	Methyltransferase like 7B	*METTL7B*
2370	56,5	69,4	-1,23	0,01580	0,364	226488_at	RCC1 domain containing 1	*RCCD1*
5856	12,5	15,3	-1,23	0,04735	0,442	1565483_at	Receptor tyrosine-protein kinase erbB-1	*EGFR*
1254	7,17	8,81	-1,23	0,00747	0,325	1555617_x_at	-	-
686	24,7	30,4	-1,23	0,00393	0,308	213847_at	Peripherin	*PRPH*
2316	118	145	-1,23	0,01538	0,363	1554149_at	Claudin domain containing 1	*CLDND1*
1207	204	250	-1,23	0,00716	0,324	202529_at	Phosphoribosyl pyrophosphate synthetase-associated protein 1	*PRPSAP1*
2286	5,83	7,15	-1,23	0,01520	0,363	242941_x_at	T-box 1	*TBX1*
5469	7,69	9,44	-1,23	0,04324	0,432	205057_s_at	Iduronidase, alpha-L-	*IDUA*
1233	320	392	-1,23	0,00733	0,324	202567_at	Small nuclear ribonucleoprotein D3 polypeptide 18kDa	*SNRPD3*
1238	42,7	52,4	-1,23	0,00736	0,324	223931_s_at	Checkpoint with forkhead and ring finger domains	*CHFR*
4979	65,6	80,4	-1,23	0,03821	0,419	204247_s_at	Cyclin-dependent kinase 5	*CDK5*
614	7,84	9,60	-1,23	0,00348	0,308	223564_s_at	Guanine nucleotide binding protein (G protein), beta polypeptide 1-like	*GNB1L*
1631	22,0	27,0	-1,23	0,01028	0,344	238690_at	-	-
4743	551	676	-1,23	0,03593	0,414	207809_s_at	ATPase, H+ transporting, lysosomal accessory protein 1	*ATP6AP1*
2361	7,21	8,84	-1,23	0,01575	0,364	205805_s_at	Receptor tyrosine kinase-like orphan Receptor 1	*ROR1*
1515	23,5	28,8	-1,23	0,00936	0,337	218670_at	Pseudouridylate synthase 1	*PUS1*
221	28,7	35,1	-1,23	0,00102	0,250	205142_x_at	ATP-binding cassette, sub-family D (ALD), member 1	*ABCD1*
5554	12,7	15,6	-1,23	0,04421	0,435	242938_s_at	Forkhead box K2	*FOXK2*
1602	19,0	23,2	-1,22	0,00999	0,341	214228_x_at	Tumor necrosis factor receptor superfamily, member 4	*TNFRSF4*
3681	4,60	5,64	-1,22	0,02658	0,395	216959_x_at	Neuronal cell adhesion molecule	*NRCAM*
5203	57,8	70,7	-1,22	0,04013	0,422	203612_at	Bystin-like	*BYSL*
641	9,76	12,0	-1,22	0,00365	0,308	204239_s_at	Neuronatin	*NNAT*
3514	13,6	16,7	-1,22	0,02522	0,392	228525_at	Similar to Low density lipoprotein receptor-related protein 3	-
5867	103	127	-1,22	0,04742	0,442	217913_at	Vacuolar protein sorting 4 homolog A (S. cerevisiae)	*VPS4A*
3036	66,5	81,4	-1,22	0,02140	0,385	209895_at	Protein tyrosine phosphatase, non-receptor type 11	*PTPN11*
1891	168	205	-1,22	0,01228	0,355	212231_at	F-box protein 21	*FBXO21*
4631	26,1	31,9	-1,22	0,03493	0,412	222517_at	Adaptor-related protein complex 3, mu 1 subunit	*AP3M1*
969	133	163	-1,22	0,00562	0,315	217796_s_at	Nuclear protein localization 4 homolog (S. cerevisiae)	*NPLOC4*
4795	13,3	16,3	-1,22	0,03637	0,415	214755_at	UDP-N-acteylglucosamine pyrophosphorylase 1-like 1	*UAP1L1*
5447	13,8	16,9	-1,22	0,04297	0,431	206186_at	Membrane protein, palmitoylated 3	*MPP3*
4930	33,4	40,9	-1,22	0,03781	0,419	225616_at	SPRY domain containing 4	*SPRYD4*
5058	7,19	8,79	-1,22	0,03895	0,420	220577_at	GTPase, very large interferon inducible 1	*GVIN1*
3977	100	123	-1,22	0,02908	0,400	225879_at	tRNA splicing endonuclease 54 homolog (S. cerevisiae)	*TSEN54*
4817	11,6	14,2	-1,22	0,03662	0,416	221158_at	Chromosome 21 open reading frame 66	*C21orf66*
3483	276	337	-1,22	0,02501	0,392	210213_s_at	Eukaryotic translation initiation factor 6	*EIF6*
5768	10,1	12,3	-1,22	0,04634	0,439	228449_at	Similar to LOC150291 protein	-
5483	498	608	-1,22	0,04339	0,433	209046_s_at	GABA(A) receptor-associated protein-like 2	*GABARAPL2*
2848	3,91	4,77	-1,22	0,01994	0,383	222270_at	SMEK homolog 2, suppressor of mek1 (Dictyostelium)	*SMEK2*
6020	49,4	60,3	-1,22	0,04899	0,445	202111_at	Solute carrier family 4, anion exchanger, member 2	*SLC4A2*
3252	11,9	14,6	-1,22	0,02308	0,388	227876_at	KIAA1688 protein	*KIAA1688*

Index	Mutated*	Normal*	FC	*P* value	FDR**	Probe set	Description	Gene symbol
32	12,6	15,3	-1,22	0,00014	0,201	230169_at	THAP domain containing 6	*THAP6*
5635	21,1	25,7	-1,22	0,04498	0,436	226564_at	ZFAT zinc finger 1	*ZFAT1*
4508	13,1	16,0	-1,22	0,03397	0,412	228676_at	Oral cancer overexpressed 1	*ORAOV1*
1506	5,62	6,85	-1,22	0,00927	0,336	238782_at	cDNA FLJ41633 fis, clone FCBBF3003435	-
2428	32,0	39,0	-1,22	0,01623	0,365	217924_at	Chromosome 6 open reading frame 106	*C6orf106*
2373	180	220	-1,22	0,01583	0,364	226935_s_at	CLPTM1-like	*CLPTM1L*
6107	20,7	25,2	-1,22	0,04985	0,446	236989_at	Transcribed locus	-
5613	48,5	59,1	-1,22	0,04477	0,436	218927_s_at	Carbohydrate (chondroitin 4) sulfotransferase 12	*CHST12*
1248	11,5	14,1	-1,22	0,00741	0,324	227901_at	Hypothetical LOC648987	*LOC648987*
4632	31,8	38,8	-1,22	0,03494	0,412	213688_at	Calmodulin 1 (phosphorylase kinase, delta)	*CALM1*
2660	11,5	14,0	-1,22	0,01816	0,373	230293_at	MSTP088 (MST088)	-
2065	14,3	17,4	-1,22	0,01348	0,356	214819_at	IQ motif and Sec7 domain 2	*IQSEC2*
483	11,8	14,4	-1,22	0,00258	0,289	1554616_at	Serpin peptidase inhibitor, clade B (ovalbumin), member 8	*SERPINB8*
5187	14,6	17,8	-1,22	0,03997	0,421	217124_at	IQ motif containing E	*IQCE*
5544	6,35	7,75	-1,22	0,04407	0,435	208253_at	Sialic acid binding Ig-like lectin 8	*SIGLEC8*
4978	69,0	84,1	-1,22	0,03820	0,419	209217_s_at	WD repeat domain 45	*WDR45*
2211	9,47	11,5	-1,22	0,01465	0,362	227726_at	Ring finger protein 166	*RNF166*
4096	8,28	10,1	-1,22	0,03010	0,402	228641_at	Caspase recruitment domain family, member 8	*CARD8*
5821	31,5	38,3	-1,22	0,04704	0,442	229741_at	Hypothetical protein MGC3260	*MGC3260*
2543	16,1	19,6	-1,22	0,01719	0,369	227799_at	Myosin IG	*MYO1G*
4198	58,3	70,9	-1,22	0,03103	0,404	202407_s_at	PRP31 pre-mRNA processing factor 31 homolog (S. cerevisiae)	*PRPF31*
3789	25,9	31,5	-1,22	0,02759	0,398	229121_at	cDNA FLJ44441 fis, clone UTERU2020242	-
3525	14,3	17,4	-1,22	0,02529	0,392	241640_at	B-cell receptor-associated protein 29	*BCAP29*
132	6,48	7,89	-1,22	0,00059	0,240	220138_at	Heart and neural crest derivatives expressed 1	*HAND1*
3368	5,77	7,02	-1,22	0,02393	0,388	235291_s_at	Hypothetical protein LOC643977	*FLJ32255*
4611	10,1	12,3	-1,22	0,03482	0,412	215938_s_at	Phospholipase A2, group VI (cytosolic, calcium-independent)	*PLA2G6*
4766	45,4	55,3	-1,22	0,03611	0,414	221752_at	Slingshot homolog 1 (Drosophila)	*SSH1*
6115	45,3	55,1	-1,22	0,04992	0,446	227815_at	Transcribed locus	-
183	4,12	5,02	-1,22	0,00085	0,244	207226_at	Histone cluster 1, H2bn	*HIST1H2BN*
5398	5,43	6,60	-1,22	0,04234	0,429	239696_at	Transcribed locus	-
2638	6,80	8,26	-1,22	0,01798	0,373	216929_x_at	ABO blood group	*ABO*
145	6,30	7,66	-1,22	0,00066	0,244	207510_at	Bradykinin receptor B1	*BDKRB1*
3641	774	940	-1,22	0,02633	0,395	208689_s_at	Ribophorin II	*RPN2*
856	6,71	8,15	-1,22	0,00494	0,313	243017_at	Transcribed locus	-
3443	35,0	42,5	-1,21	0,02463	0,391	205448_s_at	Mitogen-activated protein kinase kinase kinase 12	*MAP3K12*
1038	15,1	18,3	-1,21	0,00604	0,318	218457_s_at	DNA (cytosine-5-)-methyltransferase 3 alpha	*DNMT3A*
1848	23,7	28,8	-1,21	0,01190	0,352	238760_at	Tyrosyl-tRNA synthetase	*YARS*
107	57,7	70,1	-1,21	0,00043	0,217	225271_at	Transmembrane protein 63B	*TMEM63B*
2681	13,5	16,3	-1,21	0,01836	0,374	226442_at	Ankyrin repeat and BTB (POZ) domain containing 1	*ABTB1*
799	28,3	34,3	-1,21	0,00456	0,311	1553015_a_at	RecQ protein-like 4	*RECQL4*
2594	42,2	51,2	-1,21	0,01765	0,371	210647_x_at	Phospholipase A2, group VI (cytosolic, calcium-independent)	*PLA2G6*
538	19,7	23,9	-1,21	0,00289	0,293	229408_at	Histone deacetylase 5	*HDAC5*
3467	10,9	13,3	-1,21	0,02484	0,392	235614_at	Transmembrane protein 151	*TMEM151*
2684	94,0	114	-1,21	0,01839	0,375	200913_at	Protein phosphatase 2, catalytic subunit, gamma isoform	*PPM1G*
634	5,51	6,69	-1,21	0,00361	0,308	236787_at	cDNA FLJ35091 fis, clone PLACE6005786	-
5036	16,4	19,9	-1,21	0,03880	0,420	231003_at	Solute carrier family 35, member B3	*SLC35B3*
2956	20,4	24,8	-1,21	0,02076	0,384	215283_at	Hypothetical gene supported by BC041875	*LOC400642*
4242	47,7	57,9	-1,21	0,03148	0,406	215982_s_at	Dom-3 homolog Z (C. elegans)	*DOM3Z*
404	11,4	13,8	-1,21	0,00213	0,286	1570156_s_at	Formin 1	*FMN1*
777	7,80	9,46	-1,21	0,00445	0,311	244707_at	Transcribed locus	-
3372	21,7	26,3	-1,21	0,02399	0,389	204947_at	E2F transcription factor 1	*E2F1*
3918	154	186	-1,21	0,02861	0,399	218132_s_at	tRNA splicing endonuclease 34 homolog (S. cerevisiae)	*TSEN34*
1031	6,42	7,78	-1,21	0,00597	0,317	238829_at	Transcribed locus	-
367	27,5	33,3	-1,21	0,00191	0,284	218725_at	Solute carrier family 25, member 22	*SLC25A22*
5981	340	412	-1,21	0,04856	0,444	201011_at	Ribophorin I	*RPN1*
2674	62,8	76,1	-1,21	0,01828	0,374	90265_at	Centaurin, alpha 1	*CENTA1*
1626	12,4	15,0	-1,21	0,01023	0,344	205484_at	Signaling threshold regulating transmembrane adaptor 1	*SIT1*

Index	Mutated*	Normal*	FC	*P* value	FDR**	Probe set	Description	Gene symbol
5172	124	150	-1,21	0,03984	0,421	1564207_at	Hypothetical protein FLJ35390	*FLJ35390*
5460	28,8	34,9	-1,21	0,04313	0,432	221991_at	Neurexophilin 3	*NXPH3*
5226	5,52	6,69	-1,21	0,04043	0,423	224525_s_at	Obg-like ATPase 1	*OLA1*
5309	40,6	49,1	-1,21	0,04143	0,426	212871_at	Mitogen-activated protein kinase-activated protein kinase 5	*MAPKAPK5*
1090	6,00	7,27	-1,21	0,00640	0,319	212272_at	Lipin 1	*LPIN1*
3932	5,02	6,08	-1,21	0,02874	0,399	238598_s_at	cDNA FLJ44201 fis, clone THYMU3000841	-
4119	9,99	12,1	-1,21	0,03028	0,402	211210_x_at	SH2 domain protein 1A, Duncan's disease	*SH2D1A*
2349	3,89	4,70	-1,21	0,01567	0,364	244224_x_at	Ly6/neurotoxin 1	*LYNX1*
3855	163	198	-1,21	0,02815	0,399	212361_s_at	ATPase, Ca++ transporting, cardiac muscle, slow twitch 2	*ATP2A2*
4086	8,51	10,3	-1,21	0,03002	0,402	1553568_a_at	Histone cluster 1, H1t	*HIST1H1T*
1750	13,2	16,0	-1,21	0,01117	0,348	217187_at	Mucin 5AC, oligomeric mucus/gel-forming	*MUC5AC*
5004	1493	1805	-1,21	0,03843	0,420	200925_at	Cytochrome c oxidase subunit VIa polypeptide 1	*COX6A1*
4982	299	362	-1,21	0,03822	0,419	211759_x_at	Tubulin folding cofactor B	*TBCB*
1897	10,3	12,5	-1,21	0,01233	0,355	239484_at	cDNA clone IMAGE:4795796	-
3823	15,9	19,2	-1,21	0,02790	0,399	242048_at	Prefoldin subunit 6	*PFDN6*
892	10,8	13,0	-1,21	0,00516	0,313	220730_at	Hypothetical protein FLJ12986	*FLJ12986*
4654	28,9	34,9	-1,21	0,03515	0,413	221634_at	Ribosomal protein L23a pseudogene 7	*RPL23AP7*
1275	23,2	28,0	-1,21	0,00759	0,325	228419_at	cDNA FLJ32491 fis, clone SKNSH1000308	-
4525	41,3	49,9	-1,21	0,03414	0,412	203099_s_at	Chromodomain protein, Y-like	*CDYL*
267	8,65	10,4	-1,21	0,00127	0,260	210659_at	Chemokine-like receptor 1	*CMKLR1*
3884	12,0	14,5	-1,21	0,02836	0,399	205865_at	AT rich interactive domain 3A (BRIGHT-like)	*ARID3A*
4843	22,4	27,0	-1,21	0,03692	0,417	230334_at	cDNA clone IMAGE:40078968	-
678	19,3	23,3	-1,21	0,00390	0,308	222648_at	TBC1 domain family, member 16	*TBC1D16*
6066	19,9	24,0	-1,21	0,04948	0,446	203036_s_at	Metastasis suppressor 1	*MTSS1*
3367	10,1	12,2	-1,21	0,02392	0,388	234955_at	Similar to Zinc-alpha-2-glycoprotein precursor	*LOC401393*
1601	5,70	6,88	-1,21	0,00998	0,341	222019_at	Prefoldin subunit 6	*PFDN6*
1489	495	597	-1,21	0,00918	0,336	201527_at	ATPase, H+ transporting, lysosomal 14kDa, V1 subunit F	*ATP6V1F*
1277	4,22	5,09	-1,21	0,00760	0,325	215149_at	cDNA clone IMAGE:5260262	-
5053	37,2	44,9	-1,21	0,03893	0,420	218777_at	Receptor accessory protein 4	*REEP4*
3867	13,0	15,6	-1,21	0,02824	0,399	228753_at	Similar to XP_001148836.1	-
2915	8,37	10,1	-1,21	0,02047	0,383	240295_at	Transcribed locus	-
1504	176	212	-1,21	0,00924	0,336	201050_at	Phospholipase D family, member 3	*PLD3*
5021	10,8	13,0	-1,21	0,03862	0,420	207697_x_at	Leukocyte immunoglobulin-like receptor, subfamily B member 2	*LILRB2*
546	30,8	37,1	-1,20	0,00295	0,295	220036_s_at	Limb region 1 homolog (mouse)-like	*LMBR1L*
20	5,29	6,37	-1,20	0,00008	0,201	239198_at	Enhancer of zeste homolog 1 (Drosophila)	*EZH1*
2579	11,3	13,6	-1,20	0,01750	0,371	222087_at	Pvt1 oncogene homolog, MYC activator (mouse)	*PVT1*
71	8,38	10,1	-1,20	0,00028	0,201	1552887_at	Chromosome 10 open reading frame 27	*C10orf27*
4620	12,5	15,0	-1,20	0,03485	0,412	227342_s_at	Myeloma overexpressed gene (hypothetical protein LOC26579)	*MYEOV*
1882	31,2	37,6	-1,20	0,01222	0,355	227907_at	cDNA clone IMAGE:6470300	-
3055	5,25	6,32	-1,20	0,02152	0,385	204714_s_at	Coagulation factor V (proaccelerin, labile factor)	*F5*
4354	16,1	19,3	-1,20	0,03241	0,407	213884_s_at	Tripartite motif-containing 3	*TRIM3*
430	15,4	18,5	-1,20	0,00227	0,288	211693_at	Immunoglobulin heavy constant alpha 1	*IGHA1*
2847	8,43	10,1	-1,20	0,01992	0,383	227704_at	Chromosome 19 open reading frame 12	*C19orf12*
768	401	482	-1,20	0,00437	0,311	223026_s_at	Vacuolar protein sorting 29 homolog (S. cerevisiae)	*VPS29*
2115	145	174	-1,20	0,01387	0,359	222460_s_at	NEFA-interacting nuclear protein NIP30	*NIP30*
5131	7,50	9,02	-1,20	0,03949	0,421	207795_s_at	Killer cell lectin-like receptor subfamily D, member 1	*KLRD1*
402	10,2	12,2	-1,20	0,00211	0,286	217017_at	Oxysterol binding protein-like 10	*OSBPL10*
2993	19,2	23,1	-1,20	0,02109	0,385	221293_s_at	Differentially expressed in FDCP 6 homolog (mouse)	*DEF6*
254	6,60	7,93	-1,20	0,00118	0,254	221331_x_at	Cytotoxic T-lymphocyte-associated protein 4	*CTLA4*
4983	15,0	18,1	-1,20	0,03823	0,419	214957_at	Actin-like 8	*ACTL8*
6078	15,0	18,0	-1,20	0,04962	0,446	230139_at	cDNA FLJ43345 fis, clone NT2RI3008228	-
3497	5,05	6,07	-1,20	0,02514	0,392	232965_at	Hypothetical gene supported by BC000922	*LOC400684*
2938	4,39	5,27	-1,20	0,02060	0,383	235607_at	Similar to Heterogeneous nuclear ribonucleoprotein D0	-
3620	46,8	56,3	-1,20	0,02614	0,395	219447_s_at	Solute carrier family 35, member C2	*SLC35C2*
5659	7,52	9,03	-1,20	0,04514	0,436	1562953_s_at	Chromosome 4 open reading frame 12	*C4orf12*
515	11,8	14,2	-1,20	0,00275	0,291	224805_s_at	Chromosome 15 open reading frame 17	*C15orf17*
2023	53,0	63,6	-1,20	0,01320	0,355	225287_s_at	Transmembrane protein 55B	*TMEM55B*

Index	Mutated*	Normal*	FC	*P* value	FDR**	Probe set	Description	Gene symbol
6003	21,9	26,2	-1,20	0,04879	0,444	241792_x_at	cDNA FLJ33355 fis, clone BRACE2005151	-
3510	2053	2465	-1,20	0,02522	0,392	213187_x_at	Ferritin, light polypeptide	*FTL*
2330	7,20	8,64	-1,20	0,01553	0,364	215195_at	Protein kinase C, alpha	*PRKCA*
4260	77,9	93,5	-1,20	0,03166	0,406	217427_s_at	HIR histone cell cycle regulation defective homolog A	*HIRA*
4481	8,01	9,61	-1,20	0,03370	0,411	217486_s_at	Zinc finger, DHHC-type containing 17	*ZDHHC17*
5750	51,3	61,5	-1,20	0,04614	0,439	1558304_s_at	tRNA splicing endonuclease 54 homolog (S. cerevisiae)	*TSEN54*
2668	20,7	24,9	-1,20	0,01825	0,374	218939_at	Leucine zipper-EF-hand containing transmembrane protein 1	*LETM1*
1264	6,94	8,33	-1,20	0,00752	0,325	235686_at	Chromosome 2 open reading frame 60	*C2orf60*
4253	15,3	18,3	-1,20	0,03157	0,406	201808_s_at	Endoglin (Osler-Rendu-Weber syndrome 1)	*ENG*
4658	306	367	-1,20	0,03518	0,413	201804_x_at	Tubulin folding cofactor B	*TBCB*
4689	201	241	-1,20	0,03541	0,413	213404_s_at	RAS homolog enriched in brain	*RHEB*
2799	7,16	8,59	-1,20	0,01944	0,380	219873_at	Collectin sub-family member 11	*COLEC11*
22	11,7	14,1	-1,20	0,00009	0,201	219246_s_at	2-oxoglutarate and iron-dependent oxygenase domain-containing protein 2	*OGFOD2*
1935	30,3	36,3	-1,20	0,01261	0,355	215873_x_at	ATP-binding cassette, sub-family C (CFTR/MRP), member 10	*ABCC10*
4281	10,3	12,3	-1,20	0,03181	0,406	229112_at	Hypothetical protein LOC285813	*LOC285813*
1071	6,74	8,08	-1,20	0,00628	0,319	234668_at	cDNA FLJ23175 fis, clone LNG10438	-
1557	7,39	8,85	-1,20	0,00961	0,337	239366_at	Transcribed locus	-
621	7,00	8,39	-1,20	0,00352	0,308	240761_at	Transcribed locus	-
4208	12,9	15,5	-1,20	0,03111	0,404	205243_at	Solute carrier family 13, member 3	*SLC13A3*
2833	19,3	23,1	-1,20	0,01977	0,381	203507_at	CD68 molecule	*CD68*
709	3,56	4,27	-1,20	0,00407	0,308	211006_s_at	Potassium voltage-gated channel subfamily B member 1	*KCNB1*
804	4,30	5,15	-1,20	0,00459	0,311	239588_s_at	-	-
151	5,07	6,07	-1,20	0,00069	0,244	1552915_at	Interleukin 28A (Interferon, lambda 2)	*IL28A*
265	6,24	7,47	-1,20	0,00126	0,260	207778_at	Regenerating islet-derived 1 pseudogene	*REGL*
4407	96,5	116	-1,20	0,03290	0,408	222451_s_at	Zinc finger, DHHC-type containing 9	*ZDHHC9*
1691	15,7	18,8	-1,20	0,01070	0,346	222304_x_at	Olfactory receptor, family 7, subfamily E, member 47 pseudogene	*OR7E47P*
1780	16,0	19,2	-1,20	0,01135	0,349	226232_at	cDNA FLJ33772 fis, clone BRSSN2000175	-
718	11,4	13,6	-1,20	0,00412	0,308	220671_at	CCR4 carbon catabolite repression 4-like (S. cerevisiae)	*CCRN4L*
3558	35,7	42,7	-1,20	0,02568	0,394	218907_s_at	Leucine rich repeat containing 61	*LRRC61*
5057	14,1	16,9	-1,20	0,03895	0,420	239324_at	cDNA clone IMAGE:2960540	-
611	13,8	16,5	-1,20	0,00346	0,308	219907_at	Fibroblast growth factor receptor substrate 3	*FRS3*
3717	80,1	95,8	-1,20	0,02695	0,396	224735_at	Cytochrome b, ascorbate dependent 3	*CYBASC3*
2425	44,6	53,4	-1,20	0,01622	0,365	204706_at	Inositol polyphosphate-5-phosphatase, 72 kDa	*INPP5E*
5877	461	552	-1,20	0,04749	0,442	205353_s_at	Phosphatidylethanolamine binding protein 1	*PEBP1*
26	3,16	3,78	-1,20	0,00012	0,201	1568656_at	cDNA clone IMAGE:5265210	-
3540	374	447	-1,20	0,02547	0,393	224779_s_at	Family with sequence similarity 96, member A	*FAM96A*
861	12,0	14,4	-1,20	0,00497	0,313	210264_at	G protein-coupled receptor 35	*GPR35*
2215	5,36	6,41	-1,20	0,01467	0,362	231025_at	Transcribed locus	-
2713	6,77	8,10	-1,20	0,01862	0,375	224399_at	Programmed cell death 1 ligand 2	*PDCD1LG2*
808	39,6	47,3	-1,20	0,00461	0,311	220762_s_at	Guanine nucleotide binding protein (G protein), beta polypeptide 1-like	*GNB1L*
3351	301	359	-1,20	0,02380	0,388	226177_at	Glycolipid transfer protein	*GLTP*
3827	86,4	103	-1,20	0,02793	0,399	215631_s_at	Breast cancer metastasis suppressor 1	*BRMS1*
3056	4,64	5,54	-1,20	0,02152	0,385	239470_at	Hypothetical protein LOC644809	*FLJ38596*
4179	56,9	68,0	-1,20	0,03089	0,404	205061_s_at	Exosome component 9	*EXOSC9*
6033	26,2	31,3	-1,19	0,04910	0,445	206351_s_at	Peroxisome biogenesis factor 10	*PEX10*
3903	14,6	17,5	-1,19	0,02851	0,399	210821_x_at	Centromere protein A	*CENPA*
2137	21,2	25,3	-1,19	0,01405	0,359	64432_at	Chromosome 12 open reading frame 47	*C12orf47*
5435	9,41	11,2	-1,19	0,04283	0,431	221640_s_at	Leucine-rich repeats and death domain containing	*LRDD*
964	4,87	5,82	-1,19	0,00559	0,315	239041_at	Histone cluster 1, H2ak	*HIST1H2AK*
3539	41,3	49,3	-1,19	0,02547	0,393	1554721_a_at	Transcription initiation factor TFIID 150 kDa subunit	*TAF2*
1477	4,36	5,20	-1,19	0,00905	0,335	232827_at	Clone L50 polyadenylated HERV LTR sequence	-
3632	12,4	14,8	-1,19	0,02626	0,395	207873_x_at	Seizure related 6 homolog (mouse)-like	*SEZ6L*
1625	32,0	38,2	-1,19	0,01022	0,344	202879_s_at	Pleckstrin homology, Sec7 and coiled-coil domains 1	*PSCD1*
3328	31,7	37,8	-1,19	0,02363	0,388	227146_at	Quiescin Q6 sulfhydryl oxidase 2	*QSOX2*
42	4,19	5,00	-1,19	0,00017	0,201	214248_s_at	Tripartite motif-containing 2	*TRIM2*
5913	272	324	-1,19	0,04794	0,443	218462_at	Brix domain containing 5	*BXDC5*
595	39,9	47,6	-1,19	0,00328	0,301	221686_s_at	RecQ protein-like 5	*RECQL5*

Index	Mutated*	Normal*	FC	*P* value	FDR**	Probe set	Description	Gene symbol
3375	14,4	17,1	-1,19	0,02402	0,389	212844_at	Ribosomal RNA processing 1 homolog B (S. cerevisiae)	*RRP1B*
2112	362	432	-1,19	0,01385	0,358	224129_s_at	Dpy-30-like protein	*HDPY-30*
5836	5,80	6,92	-1,19	0,04715	0,442	240615_at	cDNA FLJ34894 fis, clone NT2NE2017982	-
2916	4,76	5,68	-1,19	0,02047	0,383	206004_at	Transglutaminase 3	*TGM3*
1422	20,6	24,6	-1,19	0,00862	0,331	1564031_a_at	RELT-like 2	*RELL2*
5326	138	165	-1,19	0,04163	0,427	202084_s_at	SEC14-like 1 (S. cerevisiae)	*SEC14L1*
948	42,2	50,3	-1,19	0,00544	0,313	210225_x_at	Leukocyte immunoglobulin-like receptor, subfamily B member 3	*LILRB3*
5209	9,87	11,8	-1,19	0,04020	0,422	231312_at	Transcribed locus	-
4856	4,82	5,75	-1,19	0,03703	0,417	244074_at	Full-length cDNA clone CS0DI026YO05	-
5493	5,75	6,85	-1,19	0,04348	0,433	209776_s_at	Solute carrier family 19, member 1	*SLC19A1*
2732	18,1	21,5	-1,19	0,01884	0,377	204522_at	Dom-3 homolog Z (C. elegans)	*DOM3Z*
5814	15,8	18,8	-1,19	0,04698	0,442	206818_s_at	Cyclin M2	*CNNM2*
233	6,48	7,71	-1,19	0,00108	0,250	1554367_at	Family with sequence similarity 26, member C	*FAM26C*
3645	7,12	8,48	-1,19	0,02634	0,395	228395_at	Glycosyltransferase 8 domain containing 1	*GLT8D1*
1947	17,7	21,1	-1,19	0,01272	0,355	203977_at	Tafazzin	*TAZ*
4280	12,9	15,4	-1,19	0,03180	0,406	241427_x_at	cDNA DKFZP434I0714	-
841	7,89	9,40	-1,19	0,00482	0,313	1557532_at	NADH-ubiquinone oxidoreductase subunit B14.5a	*NDUFA7*
2536	6,43	7,65	-1,19	0,01715	0,369	227321_at	Opposite strand transcription unit to STAG3	*GATS*
4499	8,71	10,4	-1,19	0,03384	0,411	216253_s_at	Parvin, beta	*PARVB*
4714	47,5	56,6	-1,19	0,03567	0,413	213473_at	BRCA1 associated protein	*BRAP*
4248	14,0	16,6	-1,19	0,03154	0,406	241839_at	Transcribed locus	-
260	18,4	21,9	-1,19	0,00123	0,257	1566775_at	Dynein, axonemal, heavy chain 1	*DNAH1*
3032	13,2	15,8	-1,19	0,02137	0,385	227965_at	Full-length cDNA clone CS0DJ011YE02	-
2611	6,53	7,77	-1,19	0,01773	0,371	229052_at	Ankyrin repeat domain 39	*ANKRD39*
1558	23,4	27,8	-1,19	0,00964	0,338	230062_at	KIAA1666 protein	*KIAA1666*
3793	7,07	8,41	-1,19	0,02762	0,398	228646_at	Protein phosphatase 1, regulatory (inhibitor) subunit 1C	*PPP1R1C*
1168	7,70	9,16	-1,19	0,00687	0,321	1568706_s_at	Advillin	*AVIL*
3838	12,4	14,8	-1,19	0,02804	0,399	210008_s_at	Mitochondrial ribosomal protein S12	*MRPS12*
1977	3,09	3,67	-1,19	0,01291	0,355	1556776_a_at	Full length insert cDNA YN57B01	-
5459	357	424	-1,19	0,04312	0,432	201013_s_at	Multifunctional protein ADE2	*PAICS*
5479	16,4	19,5	-1,19	0,04335	0,433	235359_at	Leucine rich repeat containing 33	*LRRC33*
3105	7,43	8,84	-1,19	0,02192	0,386	226205_at	Ankyrin repeat domain 13 family, member D	*ANKRD13D*
3200	13,8	16,3	-1,19	0,02266	0,387	211894_x_at	Seizure related 6 homolog (mouse)-like	*SEZ6L*
6021	78,9	93,8	-1,19	0,04901	0,445	224807_at	GRAM domain containing 1A	*GRAMD1A*
5123	28,6	34,0	-1,19	0,03944	0,421	225094_at	SET domain containing (lysine methyltransferase) 8	*SETD8*
882	6,50	7,73	-1,19	0,00510	0,313	221336_at	Atonal homolog 1 (Drosophila)	*ATOH1*
914	10,4	12,4	-1,19	0,00526	0,313	1552348_at	Protease, serine, 33	*PRSS33*
5928	12,5	14,8	-1,19	0,04804	0,443	211082_x_at	MAP/microtubule affinity-regulating kinase 2	*MARK2*
2687	12,0	14,3	-1,19	0,01841	0,375	207671_s_at	Bestrophin 1	*BEST1*
2081	9,73	11,6	-1,19	0,01358	0,356	1553540_a_at	Solute carrier family 29, member 2	*SLC29A2*
108	7,41	8,81	-1,19	0,00043	0,217	223779_at	Hypothetical protein MGC10981	*MGC10981*
5861	11,4	13,5	-1,19	0,04739	0,442	207509_s_at	Leukocyte-associated immunoglobulin-like receptor 2	*LAIR2*
3444	17,5	20,7	-1,19	0,02465	0,391	229612_at	Transcribed locus	-
4787	30,6	36,4	-1,19	0,03631	0,415	221809_at	RAN binding protein 10	*RANBP10*
156	9,15	10,9	-1,19	0,00071	0,244	206938_at	Steroid-5-alpha-reductase, alpha polypeptide 2	*SRD5A2*
2842	4,25	5,05	-1,19	0,01987	0,382	230776_at	cDNA FLJ36181 fis, clone TESTI2026794	-
2274	5,73	6,81	-1,19	0,01511	0,363	1559509_at	cDNA FLJ35328 fis, clone PROST2013531	-
335	5,18	6,15	-1,19	0,00170	0,277	220437_at	Hepatocellular carcinoma-associated gene TD26	*LOC55908*
2340	247	293	-1,19	0,01562	0,364	221984_s_at	Family with sequence similarity 134, member A	*FAM134A*
1108	10,1	12,0	-1,19	0,00649	0,319	211096_at	Pre-B-cell leukemia homeobox 2	*PBX2*
1614	82,3	97,6	-1,19	0,01012	0,343	202264_s_at	Translocase of outer mitochondrial membrane 40 homolog (yeast)	*TOMM40*
3323	11,7	13,9	-1,19	0,02360	0,388	232476_at	cDNA FLJ21452 fis, clone COL04505	-
5980	170	202	-1,19	0,04856	0,444	224615_x_at	Histocompatibility (minor) 13	*HM13*
3409	23,0	27,3	-1,19	0,02433	0,390	211037_s_at	Leukocyte receptor cluster (LRC) member 4	*LENG4*
4028	9,28	11,0	-1,19	0,02951	0,400	215989_at	Chromobox homolog 2 (Pc class homolog, Drosophila)	*CBX2*
509	18,2	21,6	-1,19	0,00269	0,289	233939_at	REX1, RNA exonuclease 1 homolog (S. cerevisiae)	*REXO1*
2633	97,3	115	-1,19	0,01793	0,372	201933_at	Chromatin modifying protein 1A	*CHMP1A*

Index	Mutated*	Normal*	FC	*P* value	FDR**	Probe set	Description	Gene symbol
5190	16,5	19,6	-1,19	0,03998	0,421	2028_s_at	E2F transcription factor 1	*E2F1*
2331	45,5	54,0	-1,19	0,01556	0,364	1552473_at	Guanidinoacetate N-methyltransferase	*GAMT*
5663	175	207	-1,19	0,04519	0,436	209786_at	High mobility group nucleosomal binding domain 4	*HMGN4*
4989	9,57	11,3	-1,19	0,03829	0,419	235528_at	Guanylate cyclase activator 1B (retina)	*GUCA1B*
1175	3,41	4,04	-1,18	0,00690	0,321	207793_s_at	Erythrocyte membrane protein band 4.1	*EPB41*
338	6,56	7,77	-1,18	0,00173	0,277	232320_at	cDNA FLJ22706 fis, clone HSI13163	-
4283	19,2	22,7	-1,18	0,03182	0,406	219817_at	Chromosome 12 open reading frame 47	*C12orf47*
2992	26,1	30,9	-1,18	0,02105	0,385	227633_at	RAS homolog enriched in brain	*RHEB*
3528	5,48	6,48	-1,18	0,02535	0,393	239016_at	Transcribed locus	-
5330	22,8	27,0	-1,18	0,04169	0,428	225476_at	HLA-B associated transcript 4	*BAT4*
3230	9,28	11,0	-1,18	0,02291	0,388	229855_at	Ring finger protein 34	*RNF34*
1966	23,5	27,9	-1,18	0,01285	0,355	213402_at	Zinc finger protein 787	*ZNF787*
2240	9,04	10,7	-1,18	0,01489	0,363	238072_at	cDNA FLJ38016 fis, clone CTONG2012724	-
5144	4,97	5,89	-1,18	0,03961	0,421	240869_at	Full length insert cDNA clone ZE05A03	-
684	22,6	26,8	-1,18	0,00392	0,308	209957_s_at	Natriuretic peptide precursor A	*NPPA*
689	10,9	12,9	-1,18	0,00395	0,308	232922_s_at	Chromosome 20 open reading frame 59	*C20orf59*
836	18,1	21,4	-1,18	0,00478	0,312	211173_at	Cholecystokinin A receptor	*CCKAR*
833	4,96	5,87	-1,18	0,00476	0,312	1555377_at	Olfactory receptor, family 4, subfamily D, member 2	*OR4D2*
306	5,36	6,34	-1,18	0,00148	0,263	237827_at	-	-
5411	50,3	59,6	-1,18	0,04251	0,430	229758_at	Tigger transposable element derived 5	*TIGD5*
2012	12,4	14,7	-1,18	0,01311	0,355	205575_at	Complement component 1, q subcomponent-like 1	*C1QL1*
78	7,13	8,44	-1,18	0,00030	0,201	224001_at	Family with sequence similarity 135, member B	*FAM135B*
5993	5,43	6,43	-1,18	0,04874	0,444	223573_s_at	Protein phosphatase 2A1 B gamma subunit	*PPP2R2C*
3585	4,14	4,90	-1,18	0,02588	0,394	211303_x_at	Growth-inhibiting protein 26	*PSMAL*
1941	28,3	33,5	-1,18	0,01266	0,355	237257_at	RAB4B, member RAS oncogene family	*RAB4B*
301	28,4	33,6	-1,18	0,00146	0,263	220659_s_at	Chromosome 7 open reading frame 43	*C7orf43*
604	4,81	5,69	-1,18	0,00335	0,303	216065_at	Similar to XP_530916.1	-
5715	293	346	-1,18	0,04575	0,438	208799_at	Proteasome (prosome, macropain) subunit, beta type, 5	*PSMB5*
2773	17,7	20,9	-1,18	0,01924	0,379	229864_at	Transmembrane protein 103	*TMEM103*
5329	11,1	13,2	-1,18	0,04167	0,428	237186_at	cDNA DKFZp779F2345	-
3468	10,2	12,0	-1,18	0,02485	0,392	235194_at	Two pore segment channel 2	*TPCN2*
1740	11,9	14,1	-1,18	0,01111	0,348	219996_at	Ankyrin repeat and SOCS box-containing 7	*ASB7*
4692	23,5	27,8	-1,18	0,03544	0,413	228405_at	Rhophilin, Rho GTPase binding protein 1	*RHPN1*
674	16,1	19,0	-1,18	0,00386	0,308	229637_at	Synembryn-B	*RIC8B*
5139	7,49	8,85	-1,18	0,03958	0,421	237220_at	Transcribed locus	-
1548	7,21	8,52	-1,18	0,00956	0,337	220130_x_at	Leukotriene B4 receptor 2	*LTB4R2*
2497	29,6	34,9	-1,18	0,01682	0,367	1554050_at	Sphingomyelin phosphodiesterase, acid-like 3B	*SMPDL3B*
2717	27,1	32,0	-1,18	0,01867	0,376	213098_at	RCD1 required for cell differentiation1 homolog (S. pombe)	*RQCD1*
243	4,37	5,16	-1,18	0,00111	0,250	215882_at	Centrosomal protein 152kDa	*CEP152*
2935	24,8	29,2	-1,18	0,02059	0,383	1570402_at	Kinesin light chain 3	*KLC3*
1171	7,72	9,12	-1,18	0,00688	0,321	241644_at	BolA homolog 2 (E. coli)	*BOLA2*
4985	25,9	30,6	-1,18	0,03826	0,419	223055_s_at	Exportin 5	*XPO5*
5868	76,2	89,9	-1,18	0,04743	0,442	202281_at	Cyclin G associated kinase	*GAK*
569	15,2	18,0	-1,18	0,00311	0,299	236863_at	Chromosome 17 open reading frame 67	*C17orf67*
37	28,2	33,3	-1,18	0,00015	0,201	216678_at	Intraflagellar transport 122 homolog (Chlamydomonas)	*IFT122*
121	3914	4618	-1,18	0,00056	0,240	200062_s_at	Ribosomal protein L30	*RPL30*
3736	62,7	74,0	-1,18	0,02713	0,397	221821_s_at	Chromosome 12 open reading frame 41	*C12orf41*
4636	4,14	4,88	-1,18	0,03497	0,412	217651_at	Transcribed locus	-
6079	4,52	5,33	-1,18	0,04963	0,446	1569190_at	Sodium channel and clathrin linker 1	*SCLT1*
188	115	135	-1,18	0,00087	0,244	200070_at	Chromosome 2 open reading frame 24	*C2orf24*
1860	8,51	10,0	-1,18	0,01199	0,353	228912_at	Villin 1	*VIL1*
165	19,6	23,1	-1,18	0,00076	0,244	237367_x_at	CASP8 and FADD-like apoptosis regulator	*CFLAR*
1898	20,2	23,8	-1,18	0,01234	0,355	202571_s_at	Discs, large (Drosophila) homolog-associated protein 4	*DLGAP4*
5816	586	691	-1,18	0,04698	0,442	202857_at	Transmembrane protein 4	*TMEM4*
2317	20,5	24,1	-1,18	0,01539	0,363	224079_at	Interleukin 17C	*IL17C*
4862	951	1120	-1,18	0,03710	0,417	208628_s_at	Y box binding protein 1	*YBX1*
1718	65,7	77,4	-1,18	0,01101	0,348	225789_at	Centaurin, gamma 3	*CENTG3*

Index	Mutated*	Normal*	FC	*P* value	FDR**	Probe set	Description	Gene symbol
3289	40,3	47,5	-1,18	0,02342	0,388	225437_s_at	Chromosome 7 open reading frame 27	*C7orf27*
351	4,02	4,74	-1,18	0,00179	0,278	224004_at	Zinc finger protein 226	*ZNF226*
1253	16,0	18,8	-1,18	0,00746	0,325	239592_at	Transcribed locus	-
2934	43,1	50,8	-1,18	0,02058	0,383	219611_s_at	Coiled-coil domain containing 21	*CCDC21*
2794	48,8	57,5	-1,18	0,01942	0,380	1564208_x_at	Hypothetical protein FLJ35390	*FLJ35390*
1829	6,88	8,10	-1,18	0,01179	0,351	1559064_at	Nucleoporin 153kDa	*NUP153*
1867	26,6	31,4	-1,18	0,01206	0,353	211135_x_at	Leukocyte immunoglobulin-like receptor, subfamily B member 3	*LILRB3*
1124	386	455	-1,18	0,00657	0,319	211036_x_at	Anaphase promoting complex subunit 5	*ANAPC5*
3059	5,15	6,06	-1,18	0,02153	0,385	220448_at	Potassium channel, subfamily K, member 12	*KCNK12*
659	17,5	20,6	-1,18	0,00382	0,308	217324_at	cDNA DKFZp434B1521	-
2574	38,2	45,0	-1,18	0,01745	0,371	216008_s_at	Ariadne homolog 2 (Drosophila)	*ARIH2*
59	3,71	4,37	-1,18	0,00022	0,201	241654_at	Transcribed locus	-
897	11,9	14,0	-1,18	0,00519	0,313	222094_at	Sulfotransferase family, cytosolic, 1A, phenol-preferring, member 3	*SULT1A3*
5852	24,2	28,5	-1,18	0,04733	0,442	53987_at	RAN binding protein 10	*RANBP10*
2254	40,1	47,3	-1,18	0,01500	0,363	219149_x_at	Debranching enzyme homolog 1 (S. cerevisiae)	*DBR1*
2339	7,31	8,60	-1,18	0,01561	0,364	230847_at	Werner helicase interacting protein 1	*WRNIP1*
1247	19,1	22,5	-1,18	0,00741	0,324	207408_at	Solute carrier family 22, member 14	*SLC22A14*
525	3,89	4,58	-1,18	0,00280	0,291	1553533_at	Junctophilin 1	*JPH1*
956	4,11	4,83	-1,18	0,00552	0,315	243804_at	Myotubularin related protein 7	*MTMR7*
2747	8,20	9,65	-1,18	0,01900	0,378	1554095_at	RNA binding motif protein 33	*RBM33*
931	23,9	28,1	-1,18	0,00535	0,313	211773_s_at	Zinc finger with KRAB and SCAN domains 3	*ZKSCAN3*
1498	12,7	15,0	-1,18	0,00922	0,336	223812_at	Hypothetical protein LOC51252	*LOC51252*
727	5,74	6,75	-1,18	0,00415	0,308	244709_at	Clone IMAGE:5200887, mRNA	-
5632	14,7	17,3	-1,18	0,04496	0,436	240379_at	Transcribed locus	-
74	4,88	5,74	-1,18	0,00028	0,201	206843_at	Crystallin, beta A4	*CRYBA4*
1582	118	139	-1,18	0,00985	0,340	218037_at	Family with sequence similarity 134, member A	*FAM134A*
5399	110	130	-1,18	0,04234	0,429	218126_at	Family with sequence similarity 82, member C	*FAM82C*
2959	6,53	7,68	-1,18	0,02079	0,384	242618_at	Tripartite motif-containing 26	*TRIM26*
4241	10,9	12,8	-1,18	0,03148	0,406	238226_at	Family with sequence similarity 70, member B	*FAM70B*
2162	5,16	6,06	-1,18	0,01429	0,361	235992_s_at	Hypothetical protein LOC606495	*LOC606495*
4863	6,68	7,85	-1,18	0,03710	0,417	1561595_x_at	Full length insert cDNA YN57B01	-
508	4,39	5,16	-1,18	0,00269	0,289	211614_at	C33.6 unnamed HERV-H protein	-
5664	26,7	31,4	-1,18	0,04519	0,436	212735_at	KIAA0226 protein	*KIAA0226*
1319	15,8	18,6	-1,18	0,00786	0,326	242885_at	-	-
1555	8,53	10,0	-1,18	0,00960	0,337	1559121_s_at	Ariadne homolog 2 (Drosophila)	*ARIH2*
4682	7,60	8,93	-1,18	0,03535	0,413	243680_at	Similar to XP_001152474.1	-
3624	44,6	52,4	-1,18	0,02616	0,395	212059_s_at	TNF-receptor ubiquitous scaffolding/signaling protein	*TRPC4AP*
5415	18,7	21,9	-1,17	0,04256	0,430	204795_at	Proline rich 3	*PRR3*
3326	6,55	7,69	-1,17	0,02362	0,388	220692_at	HSPC047 protein	-
2651	10,1	11,9	-1,17	0,01808	0,373	243774_at	Mucin 20, cell surface associated	*MUC20*
4964	15,2	17,9	-1,17	0,03808	0,419	244765_at	Transcribed locus	-
1768	28,7	33,7	-1,17	0,01127	0,348	208947_s_at	UPF1 regulator of nonsense transcripts homolog (yeast)	*UPF1*
3725	4,83	5,67	-1,17	0,02704	0,397	1554356_at	GINS complex subunit 4 (Sld5 homolog)	*GINS4*
5353	903	1061	-1,17	0,04200	0,429	221619_s_at	Mitochondrial carrier homolog 1 (C. elegans)	*MTCH1*
1670	6,52	7,66	-1,17	0,01054	0,345	220961_s_at	Transforming growth factor beta regulator 4	*TBRG4*
2057	13,2	15,5	-1,17	0,01340	0,356	206650_at	IQ motif containing C	*IQCC*
4900	6,57	7,71	-1,17	0,03749	0,418	210359_at	Metastasis suppressor 1	*MTSS1*
927	36,3	42,6	-1,17	0,00533	0,313	210651_s_at	EPH receptor B2	*EPHB2*
2070	4,63	5,44	-1,17	0,01353	0,356	1555868_at	Hypothetical protein LOC729070	*LOC729070*
5318	60,0	70,5	-1,17	0,04151	0,427	219022_at	Chromosome 12 open reading frame 43	*C12orf43*
4356	6,07	7,13	-1,17	0,03241	0,407	227844_at	Formin-like 3	*FMNL3*
1381	6,48	7,61	-1,17	0,00831	0,329	240257_at	Synaptojanin 2	*SYNJ2*
3852	18,1	21,3	-1,17	0,02814	0,399	212618_at	Zinc finger protein 609	*ZNF609*
178	6,04	7,09	-1,17	0,00082	0,244	1560410_at	Clone IMAGE:5241870, mRNA sequence	-
5334	6,88	8,07	-1,17	0,04176	0,428	216150_at	cDNA FLJ11547 fis, clone HEMBA1002934	-
2795	22,2	26,1	-1,17	0,01943	0,380	224064_s_at	Dehydrodolichyl diphosphate synthase	*DHDDS*
4340	47,5	55,7	-1,17	0,03233	0,407	228257_at	Ankyrin repeat domain 52	*ANKRD52*

Index	Mutated*	Normal*	FC	*P* value	FDR**	Probe set	Description	Gene symbol
2826	6,73	7,89	-1,17	0,01973	0,381	231232_at	Transcribed locus	-
5082	31,6	37,1	-1,17	0,03911	0,420	235181_at	Chromosome 2 open reading frame 60	*C2orf60*
31	4,09	4,79	-1,17	0,00014	0,201	219724_s_at	KIAA0748 protein	*KIAA0748*
3202	72,9	85,5	-1,17	0,02268	0,387	1554093_a_at	Small nuclear RNA activating complex, polypeptide 5, 19kDa	*SNAPC5*
1913	9,70	11,4	-1,17	0,01247	0,355	210100_s_at	ATP-binding cassette, sub-family A (ABC1), member 2	*ABCA2*
5444	25,0	29,3	-1,17	0,04294	0,431	1556340_at	Mitogen-activated protein kinase 12	*MAPK12*
6092	33,4	39,2	-1,17	0,04973	0,446	44065_at	Chromosome 12 open reading frame 52	*C12orf52*
5935	9,29	10,9	-1,17	0,04812	0,443	205881_at	Zinc finger protein 74	*ZNF74*
1391	12,1	14,2	-1,17	0,00841	0,330	240443_at	Transcribed locus	-
2694	109	127	-1,17	0,01848	0,375	232209_x_at	Histocompatibility (minor) 13	*HM13*
5972	21,5	25,2	-1,17	0,04849	0,444	213615_at	Membrane bound O-acyltransferase domain containing 5	*MBOAT5*
5118	119	139	-1,17	0,03940	0,421	212034_s_at	Exocyst complex component 7	*EXOC7*
3862	13,1	15,4	-1,17	0,02822	0,399	228522_at	Hypothetical protein LOC642031	*LOC642031*
417	22,0	25,8	-1,17	0,00219	0,286	209588_at	EPH receptor B2	*EPHB2*
2051	9,44	11,1	-1,17	0,01333	0,355	217349_s_at	LIM domain only 6	*LMO6*
4998	18,7	21,9	-1,17	0,03839	0,420	232106_s_at	Coiled-coil domain containing 123	*CCDC123*
237	548	642	-1,17	0,00110	0,250	208736_at	Actin related protein 2/3 complex, subunit 3, 21kDa	*ARPC3*
895	3,40	3,98	-1,17	0,00518	0,313	238361_s_at	Transcribed locus	-
4255	7,20	8,43	-1,17	0,03161	0,406	237611_at	Transcribed locus	-
5906	104	122	-1,17	0,04785	0,443	219162_s_at	Mitochondrial ribosomal protein L11	*MRPL11*
932	17,0	20,0	-1,17	0,00536	0,313	1568718_at	Chromosome 6 open reading frame 85	*C6orf85*
5511	14,0	16,4	-1,17	0,04366	0,433	216047_x_at	Seizure related 6 homolog (mouse)-like	*SEZ6L*
2473	6,19	7,25	-1,17	0,01662	0,367	208059_at	Chemokine (C-C motif) receptor 8	*CCR8*
496	6,15	7,20	-1,17	0,00265	0,289	1559754_at	Lymphotoxin beta (TNF superfamily, member 3)	*LTB*
1402	4,67	5,47	-1,17	0,00847	0,330	243220_at	Transcribed locus	-
2786	18,2	21,4	-1,17	0,01935	0,380	238627_at	Trafficking protein particle complex 2-like	*TRAPPC2L*
3264	104	121	-1,17	0,02317	0,388	223420_at	DnaJ (Hsp40) homolog, subfamily C, member 14	*DNAJC14*
460	9,63	11,3	-1,17	0,00245	0,288	231637_at	Transcribed locus	-
2184	6,47	7,58	-1,17	0,01447	0,361	244890_at	-	-
760	21,5	25,2	-1,17	0,00429	0,308	1553657_at	von Willebrand factor A domain containing 3A	*VWA3A*
4410	20,0	23,4	-1,17	0,03294	0,408	218933_at	Spermatogenesis associated 5-like 1	*SPATA5L1*
2287	18,9	22,1	-1,17	0,01520	0,363	1559870_at	Clone IMAGE:5225645, mRNA	-
1717	5,31	6,22	-1,17	0,01101	0,348	230572_at	Chromosome 4 open reading frame 38	*C4orf38*
5134	32,6	38,2	-1,17	0,03950	0,421	221846_s_at	CASK interacting protein 2	*CASKIN2*
3787	8,70	10,2	-1,17	0,02758	0,398	1556555_at	cDNA clone IMAGE:4825327	-
4004	95,5	112	-1,17	0,02930	0,400	212861_at	Major facilitator superfamily domain containing 5	*MFSD5*
2327	4,72	5,52	-1,17	0,01549	0,364	231095_at	cDNA FLJ42857 fis, clone BRHIP2009340	-
1954	115	134	-1,17	0,01276	0,355	221983_at	Family with sequence similarity 134, member A	*FAM134A*
3307	22,2	26,0	-1,17	0,02352	0,388	239368_at	Family with sequence similarity 111, member A	*FAM111A*
5535	12,0	14,0	-1,17	0,04394	0,434	215919_s_at	Mitochondrial ribosomal protein S11	*MRPS11*
4520	131	153	-1,17	0,03406	0,412	213604_at	Transcription elongation factor B (SIII), polypeptide 3	*TCEB3*
5081	38,7	45,2	-1,17	0,03910	0,420	225587_at	Transmembrane protein 129	*TMEM129*
4836	3,92	4,58	-1,17	0,03688	0,417	238977_at	Minichromosome maintenance complex component 6	*MCM6*
1128	9,05	10,6	-1,17	0,00659	0,319	237735_at	cDNA clone IMAGE:5296164	-
3271	20,4	23,8	-1,17	0,02326	0,388	227841_at	Cementum protein 1	*CEMP1*
5395	6,58	7,70	-1,17	0,04231	0,429	1554465_s_at	Zinc finger protein 673	*ZNF673*
2364	127	148	-1,17	0,01578	0,364	31837_at	Transmembrane protein 112B	*TMEM112B*
5528	65,5	76,6	-1,17	0,04384	0,434	203855_at	WD repeat domain 47	*WDR47*
5688	102	119	-1,17	0,04542	0,437	214263_x_at	Polymerase (RNA) II (DNA directed) polypeptide C, 33kDa	*POLR2C*
5268	10,4	12,1	-1,17	0,04091	0,425	229952_at	Similar to Spectrin, beta, non-erythrocytic 1 isoform 4	-
2796	9,32	10,9	-1,17	0,01943	0,380	228705_at	Calpain 12	*CAPN12*
4239	98,4	115	-1,17	0,03141	0,405	215293_s_at	FGF receptor activating protein 1	*FRAG1*
5492	3,85	4,50	-1,17	0,04347	0,433	1552897_a_at	Potassium voltage-gated channel, subfamily G, member 3	*KCNG3*
2772	19,3	22,6	-1,17	0,01924	0,379	235525_at	cDNA FLJ39926 fis, clone SPLEN2021157	-
2385	50,7	59,3	-1,17	0,01590	0,364	229164_s_at	Ankyrin repeat and BTB (POZ) domain containing 1	*ABTB1*
517	4,61	5,39	-1,17	0,00275	0,291	220447_at	Histamine receptor H3	*HRH3*
2650	7,19	8,41	-1,17	0,01807	0,373	233530_at	(clone 33) macronuclear mRNA	-

Index	Mutated*	Normal*	FC	*P* value	FDR**	Probe set	Description	Gene symbol
1711	24,0	28,0	-1,17	0,01095	0,348	1557450_s_at	Clone IMAGE:241447, mRNA sequence	-
4617	44,4	51,9	-1,17	0,03484	0,412	222732_at	Tripartite motif-containing 39	*TRIM39*
1050	10,6	12,4	-1,17	0,00614	0,319	234113_at	cDNA DKFZp313A1612	-
3422	50,8	59,4	-1,17	0,02447	0,391	218427_at	Serologically defined colon cancer antigen 3	*SDCCAG3*
3960	48,9	57,2	-1,17	0,02892	0,399	204300_at	PET112-like (yeast)	*PET112L*
5950	17,7	20,7	-1,17	0,04826	0,443	243731_at	Similar to Solute carrier family 27, member 5	-
2733	11,0	12,9	-1,17	0,01884	0,377	208544_at	Adrenergic, alpha-2B-, receptor	*ADRA2B*
493	13,8	16,1	-1,17	0,00264	0,289	205387_s_at	Chorionic gonadotropin, beta polypeptide	*CGB*
2108	4,48	5,23	-1,17	0,01383	0,358	1560021_at	cDNA FLJ37333 fis, clone BRAMY2020106	-
5273	582	679	-1,17	0,04098	0,425	200675_at	CD81 molecule	*CD81*
3193	444	518	-1,17	0,02262	0,387	223857_x_at	Transmembrane protein 85	*TMEM85*
1296	56,2	65,7	-1,17	0,00772	0,325	204189_at	Retinoic acid receptor, gamma	*RARG*
6101	7,39	8,63	-1,17	0,04981	0,446	230818_at	Transcribed locus	-
5595	3,62	4,22	-1,17	0,04461	0,436	214540_at	Histone cluster 1, H2bo	*HIST1H2BO*
5164	48,5	56,7	-1,17	0,03980	0,421	212170_at	RNA binding motif protein 12	*RBM12*
2143	62,5	73,0	-1,17	0,01411	0,360	221708_s_at	Unc-45 homolog A (C. elegans)	*UNC45A*
4880	15,9	18,6	-1,17	0,03732	0,418	237044_s_at	-	-
905	12,8	14,9	-1,17	0,00523	0,313	239785_at	DAZ interacting protein 1-like	*DZIP1L*
545	3,45	4,03	-1,17	0,00294	0,295	207874_s_at	Complement factor H-related 4	*CFHR4*
1530	29,2	34,1	-1,17	0,00947	0,337	1570197_at	Clone IMAGE:5111803, mRNA	-
3245	7,67	8,95	-1,17	0,02301	0,388	226570_at	ATPase, Na+/K+ transporting, beta 3 polypeptide	*ATP1B3*
1311	18,6	21,7	-1,17	0,00782	0,325	221981_s_at	WD repeat domain 59	*WDR59*
467	16,0	18,6	-1,17	0,00246	0,288	231723_at	Sorting nexin 12	*SNX12*
945	5,62	6,56	-1,17	0,00543	0,313	1558758_s_at	cDNA FLJ23791 fis, clone HEP21711	-
1010	24,7	28,8	-1,17	0,00586	0,316	1557105_a_at	cDNA DKFZp686P1116	-
3662	60,8	70,9	-1,17	0,02648	0,395	1552615_at	Acetyl-Coenzyme A carboxylase beta	*ACACB*
2914	3,78	4,41	-1,17	0,02047	0,383	233688_at	Kv channel interacting protein 3, calsenilin	*KCNIP3*
739	10,1	11,8	-1,17	0,00420	0,308	240763_at	Transcribed locus	-
1289	44,1	51,4	-1,17	0,00767	0,325	203677_s_at	TAR (HIV-1) RNA binding protein 2	*TARBP2*
998	6,74	7,86	-1,17	0,00576	0,316	243156_at	Transcribed locus	-
541	7,87	9,17	-1,17	0,00290	0,293	236998_at	Coiled-coil domain containing 56	*CCDC56*
3411	6,42	7,49	-1,17	0,02434	0,390	208087_s_at	Z-DNA binding protein 1	*ZBP1*
946	15,6	18,2	-1,17	0,00543	0,313	233694_at	Heat shock 70kDa protein 1-like	*HSPA1L*
299	5,70	6,64	-1,17	0,00145	0,263	230806_s_at	Family with sequence similarity 65, member A	*FAM65A*
453	8,54	9,95	-1,17	0,00243	0,288	220057_at	X antigen family, member 1D	*XAGE1D*
2997	14,9	17,4	-1,17	0,02111	0,385	235239_at	Quiescin Q6 sulfhydryl oxidase 2	*QSOX2*
3428	42,2	49,2	-1,17	0,02454	0,391	204691_x_at	Phospholipase A2, group VI (cytosolic, calcium-independent)	*PLA2G6*
1760	17,3	20,2	-1,17	0,01122	0,348	1554523_a_at	Cyclin M2	*CNNM2*
40	4,68	5,45	-1,17	0,00017	0,201	1557879_at	Coiled-coil domain containing 108	*CCDC108*
645	14,3	16,7	-1,17	0,00367	0,308	243266_at	Transcribed locus	-
1629	7,28	8,49	-1,17	0,01026	0,344	234133_s_at	Hypothetical protein LOC728543	*LOC728543*
891	17,5	20,4	-1,17	0,00515	0,313	219707_at	Copine VII	*CPNE7*
2461	21,0	24,5	-1,16	0,01652	0,367	219986_s_at	Acyl-Coenzyme A dehydrogenase family, member 10	*ACAD10*
5457	26,4	30,7	-1,16	0,04312	0,432	218141_at	Ubiquitin-conjugating enzyme E2O	*UBE2O*
5783	10,3	12,0	-1,16	0,04656	0,440	215660_s_at	Microtubule associated serine/threonine kinase 2	*MAST2*
613	6,71	7,81	-1,16	0,00346	0,308	217376_at	Similar to Signal-regulatory protein gamma precursor	*LOC441938*
5914	14,8	17,3	-1,16	0,04794	0,443	224406_s_at	Fc receptor-like 5	*FCRL5*
901	69,2	80,6	-1,16	0,00522	0,313	218083_at	Prostaglandin E synthase 2	*PTGES2*
3231	6,33	7,37	-1,16	0,02291	0,388	215087_at	Chromosome 15 open reading frame 39	*C15orf39*
255	7,50	8,73	-1,16	0,00119	0,254	232430_at	Hypothetical LOC148696	*LOC148696*
3337	45,3	52,7	-1,16	0,02368	0,388	212035_s_at	Exocyst complex component 7	*EXOC7*
2789	370	430	-1,16	0,01939	0,380	200098_s_at	Anaphase promoting complex subunit 5	*ANAPC5*
608	19,3	22,4	-1,16	0,00340	0,306	1567166_at	-	-
1595	5,42	6,30	-1,16	0,00992	0,340	210746_s_at	Erythrocyte membrane protein band 4.2	*EPB42*
1251	15,7	18,3	-1,16	0,00744	0,325	207366_at	Potassium voltage-gated channel subfamily S member 1	*KCNS1*
4576	99,3	115	-1,16	0,03459	0,412	203694_s_at	DEAH (Asp-Glu-Ala-His) box polypeptide 16	*DHX16*
2151	6,43	7,49	-1,16	0,01417	0,360	1565254_s_at	Elongation factor RNA polymerase II	*ELL*

Index	Mutated*	Normal*	FC	*P* value	FDR**	Probe set	Description	Gene symbol
1699	9,19	10,7	-1,16	0,01082	0,348	222080_s_at	cDNA clone IMAGE:4865340	-
2487	3,57	4,15	-1,16	0,01677	0,367	208382_s_at	DMC1 dosage suppressor of mck1 homolog	*DMC1*
1405	5,63	6,55	-1,16	0,00849	0,330	238023_at	-	-
3152	12,3	14,4	-1,16	0,02237	0,387	220805_at	Histamine receptor H2	*HRH2*
4678	6,72	7,82	-1,16	0,03532	0,413	207495_at	RAB28, member RAS oncogene family	*RAB28*
1942	3,04	3,54	-1,16	0,01266	0,355	214586_at	G protein-coupled receptor 37 (endothelin receptor type B-like)	*GPR37*
5552	17,7	20,6	-1,16	0,04418	0,435	1555083_at	Ribosomal protein L13A pseudogene	*MGC34774*
3318	60,0	69,8	-1,16	0,02358	0,388	225620_at	RAB35, member RAS oncogene family	*RAB35*
14	3,64	4,23	-1,16	0,00005	0,191	1553447_at	ATP/GTP binding protein-like 1	*AGBL1*
5363	3,79	4,41	-1,16	0,04208	0,429	244313_at	Complement component (3b/4b) receptor 1 (Knops blood group)	*CR1*
5287	116	135	-1,16	0,04113	0,425	218089_at	Chromosome 20 open reading frame 4	*C20orf4*
2400	35,4	41,1	-1,16	0,01600	0,365	202099_s_at	DiGeorge syndrome critical region gene 2	*DGCR2*
1137	5,46	6,35	-1,16	0,00664	0,319	1553044_at	Connexin 62	*CX62*
3811	36,3	42,2	-1,16	0,02777	0,398	221250_s_at	MAX dimerization protein 3	*MXD3*
195	6,87	7,99	-1,16	0,00090	0,244	220645_at	Family with sequence similarity 55, member D	*FAM55D*
2872	11,6	13,5	-1,16	0,02018	0,383	214340_at	Arachidonate 12-lipoxygenase pseudogene 2	*ALOX12P2*
342	15,6	18,1	-1,16	0,00174	0,277	227832_at	Methyl-CpG binding domain protein 6	*MBD6*
5372	6,80	7,90	-1,16	0,04216	0,429	244294_at	cDNA FLJ26260 fis, clone DMC05193	-
374	6,54	7,60	-1,16	0,00196	0,284	216825_s_at	Myeloproliferative leukemia virus oncogene	*MPL*
3226	5,40	6,27	-1,16	0,02286	0,387	238945_at	-	-
4726	24,6	28,6	-1,16	0,03576	0,414	202385_s_at	Treacher Collins-Franceschetti syndrome 1	*TCOF1*
4125	30,1	35,0	-1,16	0,03034	0,402	236667_at	Transmembrane protein 120B	*TMEM120B*
536	6,29	7,31	-1,16	0,00286	0,292	230325_at	cDNA clone IMAGE:4393471	-
2004	5,48	6,37	-1,16	0,01308	0,355	1557196_a_at	cDNA DKFZp547B198	-
1074	7,69	8,92	-1,16	0,00630	0,319	207260_at	FEV (ETS oncogene family)	*FEV*
2875	9,86	11,5	-1,16	0,02019	0,383	231958_at	Chromosome 3 open reading frame 31	*C3orf31*
4574	16,0	18,6	-1,16	0,03457	0,412	1558529_s_at	Clone IMAGE:4778855, mRNA	-
5793	11,3	13,1	-1,16	0,04672	0,441	204465_s_at	Internexin neuronal Intermediate filament protein, alpha	*INA*
2700	17,3	20,1	-1,16	0,01852	0,375	211521_s_at	Pleckstrin homology, Sec7 and coiled-coil domains 4	*PSCD4*
4582	7,79	9,04	-1,16	0,03461	0,412	209697_at	Protein phosphatase 2B, catalytic subunit, gamma isoform	*PPP3CC*
3975	11,3	13,1	-1,16	0,02906	0,400	235939_at	cDNA FLJ36815 fis, clone ASTRO2003740	-
6075	13,3	15,4	-1,16	0,04959	0,446	213200_at	Synaptophysin	*SYP*
2869	6,34	7,36	-1,16	0,02014	0,383	236257_at	CD2-associated protein	*CD2AP*
3336	4,35	5,04	-1,16	0,02367	0,388	242183_at	cDNA FLJ38849 fis, clone MESAN2008936	-
4421	9,37	10,9	-1,16	0,03310	0,409	213078_x_at	1-acylglycerol-3-phosphate O-acyltransferase 7 (lysophosphatidic acid acyltra	*AGPAT7*
1355	7,95	9,22	-1,16	0,00814	0,328	216409_at	Acyl-CoA synthetase long-chain family member 6	*ACSL6*
954	5,47	6,34	-1,16	0,00549	0,314	1560255_at	Chromosome 10 open reading frame 31	*C10orf31*
5922	4,99	5,79	-1,16	0,04799	0,443	205955_at	-	-
5522	9,97	11,6	-1,16	0,04381	0,433	241671_x_at	Hypothetical locus LOC401237	*FLJ22536*
903	3,62	4,20	-1,16	0,00523	0,313	207433_at	Interleukin 10	*IL10*
2846	12,3	14,2	-1,16	0,01992	0,383	1554332_a_at	mRNA similar to LOC149651	-
4529	314	364	-1,16	0,03419	0,412	203462_x_at	Eukaryotic translation initiation factor 3, subunit B	*EIF3B*
1552	14,7	17,0	-1,16	0,00957	0,337	231189_at	Hypothetical protein MGC40574	*MGC40574*
4649	21,0	24,3	-1,16	0,03508	0,413	234465_at	Essential meiotic endonuclease 1 homolog 1 (S. pombe)	*EME1*
2248	9,12	10,6	-1,16	0,01494	0,363	210039_s_at	Protein kinase C, theta	*PRKCQ*
5192	10,2	11,8	-1,16	0,04001	0,421	214300_s_at	Topoisomerase (DNA) III alpha	*TOP3A*
2279	7,85	9,10	-1,16	0,01515	0,363	231664_at	Solute carrier family 25, member 29	*SLC25A29*
4234	48,5	56,2	-1,16	0,03139	0,405	218758_s_at	Ribosomal RNA processing 1 homolog (S. cerevisiae)	*RRP1*
3134	26,1	30,2	-1,16	0,02222	0,387	211133_x_at	Leukocyte immunoglobulin-like receptor, subfamily B member 3	*LILRB3*
3780	14,2	16,4	-1,16	0,02752	0,398	239933_x_at	-	-
35	4,89	5,66	-1,16	0,00015	0,201	1563743_at	Chromosome 1 open reading frame 180	*C1orf180*
1617	6,95	8,06	-1,16	0,01014	0,343	206119_at	Betaine-homocysteine methyltransferase	*BHMT*
951	8,71	10,1	-1,16	0,00545	0,314	227867_at	Hypothetical protein LOC129293	*LOC129293*
5052	9,96	11,5	-1,16	0,03892	0,420	224088_at	Neuromedin U receptor 2	*NMUR2*
4972	33,1	38,3	-1,16	0,03816	0,419	205333_s_at	RCE1 homolog, prenyl protein peptidase (S. cerevisiae)	*RCE1*
4706	29,8	34,5	-1,16	0,03558	0,413	226369_at	Hypothetical locus LOC338799	*LOC338799*
2092	4,28	4,96	-1,16	0,01367	0,357	1554086_at	Tubulin, gamma complex associated protein 3	*TUBGCP3*

Index	Mutated*	Normal*	FC	*P* value	FDR**	Probe set	Description	Gene symbol
3224	6,48	7,51	-1,16	0,02284	0,387	207600_at	Potassium voltage-gated channel subfamily C member 3	*KCNC3*
632	5,88	6,81	-1,16	0,00357	0,308	207064_s_at	Amine oxidase, copper containing 2 (retina-specific)	*AOC2*
2113	5,37	6,22	-1,16	0,01385	0,358	228022_at	Coiled-coil domain containing 18	*CCDC18*
2332	9,35	10,8	-1,16	0,01556	0,364	1557729_at	G protein-coupled receptor kinase 5	*GRK5*
1678	14,6	16,9	-1,16	0,01059	0,345	238473_at	cDNA clone IMAGE:4432160	-
1164	17,0	19,7	-1,16	0,00685	0,321	240607_at	Myocardial infarction associated transcript	*MIAT*
1014	18,6	21,6	-1,16	0,00587	0,316	238789_at	Ankyrin repeat domain 15	*ANKRD15*
5665	21,5	24,9	-1,16	0,04520	0,436	218855_at	G protein-coupled receptor 175	*GPR175*
1441	3,89	4,50	-1,16	0,00876	0,332	1562458_at	Ubiquitin-conjugating enzyme E2W	*UBE2W*
2600	6,70	7,75	-1,16	0,01766	0,371	242437_at	Similar to XP_529777.1	-
269	7,07	8,19	-1,16	0,00128	0,260	242757_at	MAM domain containing glycosylphosphatidylinositol anchor 1	*MDGA1*
1762	4,89	5,66	-1,16	0,01123	0,348	206341_at	Interleukin 2 receptor, alpha	*IL2RA*
5872	3,52	4,08	-1,16	0,04744	0,442	243689_s_at	Chromosome 20 open reading frame 80	*C20orf80*
845	5,01	5,80	-1,16	0,00485	0,313	210451_at	Pyruvate kinase, liver and RBC	*PKLR*
2133	12,5	14,5	-1,16	0,01402	0,359	205944_s_at	Clathrin, heavy chain-like 1	*CLTCL1*
5696	10,7	12,3	-1,16	0,04559	0,438	230936_at	DnaJ (Hsp40) related, subfamily B, member 13	*DNAJB13*
1665	13,5	15,6	-1,16	0,01053	0,345	232550_at	Abhydrolase domain containing 1	*ABHD1*
2043	52,9	61,2	-1,16	0,01329	0,355	34225_at	Wolf-Hirschhorn syndrome candidate 2	*WHSC2*
4947	316	365	-1,16	0,03797	0,419	208688_x_at	Eukaryotic translation initiation factor 3, subunit B	*EIF3B*
446	19,5	22,6	-1,16	0,00238	0,288	1555215_a_at	Histamine receptor H3	*HRH3*
329	8,12	9,39	-1,16	0,00164	0,272	234698_at	N-6 adenine-specific DNA methyltransferase 1 (putative)	*N6AMT1*
4367	51,3	59,3	-1,16	0,03248	0,407	201945_at	Furin (paired basic amino acid cleaving enzyme)	*FURIN*
1195	64,4	74,5	-1,16	0,00705	0,323	1552749_a_at	Kinesin light chain 3	*KLC3*
5012	7,39	8,55	-1,16	0,03852	0,420	207440_at	Solute carrier family 35, member A2	*SLC35A2*
4914	15,6	18,1	-1,16	0,03762	0,418	232134_at	cDNA FLJ11652 fis, clone HEMBA1004461	-
531	21,7	25,0	-1,16	0,00284	0,292	237630_s_at	Clone PP1195 unknown mRNA	-
1695	7,06	8,17	-1,16	0,01077	0,347	214558_at	G protein-coupled receptor 12	*GPR12*
3142	60,2	69,6	-1,16	0,02229	0,387	209490_s_at	Palmitoyl-protein thioesterase 2	*PPT2*
5510	146	168	-1,16	0,04366	0,433	211630_s_at	Glutathione synthetase	*GSS*
4737	21,7	25,1	-1,16	0,03585	0,414	1563318_s_at	MAGI family member, X-linked	*MAGIX*
4944	10,2	11,8	-1,16	0,03793	0,419	208352_x_at	Ankyrin 1, erythrocytic	*ANK1*
5231	13,3	15,4	-1,16	0,04047	0,423	205813_s_at	Methionine adenosyltransferase I, alpha	*MAT1A*
2110	5,89	6,81	-1,16	0,01384	0,358	1553789_a_at	Chromosome 21 open reading frame 58	*C21orf58*
724	4,85	5,60	-1,16	0,00414	0,308	232139_s_at	KIAA1919 protein	*KIAA1919*
4413	13,9	16,1	-1,16	0,03300	0,409	232274_at	Cyclin L2	*CCNL2*
2430	6,98	8,07	-1,16	0,01625	0,365	239800_at	Transcribed locus	-
366	13,9	16,1	-1,16	0,00191	0,284	231306_at	Lysozyme-like 4	*LYZL4*
4093	6,02	6,96	-1,16	0,03009	0,402	236242_at	-	-
2568	12,7	14,7	-1,16	0,01742	0,371	223563_at	Guanine nucleotide binding protein (G protein), beta polypeptide 1-like	*GNB1L*
1400	5,01	5,79	-1,16	0,00846	0,330	240620_at	Transcribed locus	-
4888	9,31	10,8	-1,16	0,03737	0,418	236132_at	-	-
4145	19,8	22,9	-1,16	0,03059	0,403	1556797_at	Hypothetical protein LOC386597	*LOC386597*
3031	16,0	18,5	-1,15	0,02136	0,385	236073_at	EPH receptor A10	*EPHA10*
153	7,77	8,97	-1,15	0,00070	0,244	241087_at	-	-
2144	7,56	8,73	-1,15	0,01412	0,360	204330_s_at	Mitochondrial ribosomal protein S12	*MRPS12*
4857	22,5	26,0	-1,15	0,03704	0,417	206827_s_at	Transient receptor potential cation channel subfamily V member 6	*TRPV6*
1459	7,09	8,19	-1,15	0,00891	0,334	228388_at	Nuclear factor of kappa light polypeptide gene enhancer in B-cells inhibitor, b	*NFKBIB*
1193	13,6	15,7	-1,15	0,00703	0,322	208128_x_at	Kinesin family member 25	*KIF25*
5235	12,3	14,2	-1,15	0,04051	0,423	209767_s_at	Glycoprotein Ib (platelet), beta polypeptide	*GP1BB*
3970	8,35	9,64	-1,15	0,02900	0,399	204696_s_at	Cell division cycle 25 homolog A (S. pombe)	*CDC25A*
3285	8,08	9,33	-1,15	0,02341	0,388	1554646_at	Oxysterol binding protein-like 1A	*OSBPL1A*
733	8,61	9,94	-1,15	0,00419	0,308	233196_at	cDNA FLJ11777 fis, clone HEMBA1005909	-
5402	117	135	-1,15	0,04238	0,429	213203_at	Small nuclear RNA activating complex, polypeptide 5, 19kDa	*SNAPC5*
3124	4,31	4,98	-1,15	0,02214	0,387	215056_at	Clone 23695 mRNA sequence	-
3923	4,11	4,75	-1,15	0,02868	0,399	1569315_s_at	Hypothetical protein FLJ44451	*FLJ44451*
3303	12,3	14,2	-1,15	0,02351	0,388	213870_at	Collagen, type XI, alpha 2	*COL11A2*
5333	13,3	15,4	-1,15	0,04175	0,428	230439_at	Hypothetical gene supported by BC031661	*LOC389458*

Index	Mutated*	Normal*	FC	*P* value	FDR**	Probe set	Description	Gene symbol
5217	50,1	57,8	-1,15	0,04031	0,422	203412_at	Leucine-zipper-like transcription regulator 1	*LZTR1*
3573	59,0	68,1	-1,15	0,02580	0,394	226428_at	Transportin 2 (importin 3, karyopherin beta 2b)	*TNPO2*
4667	24,2	27,9	-1,15	0,03524	0,413	227060_at	RELT tumor necrosis factor receptor	*RELT*
2678	9,52	11,0	-1,15	0,01832	0,374	233138_at	Chromosome 18 open reading frame 1	*C18orf1*
770	6,49	7,49	-1,15	0,00442	0,311	215511_at	Transcription factor 20	*TCF20*
2355	20,5	23,7	-1,15	0,01572	0,364	208242_at	Retina and anterior neural fold homeobox	*RAX*
253	5,04	5,82	-1,15	0,00117	0,254	208553_at	Histone cluster 1, H1e	*HIST1H1E*
1039	15,9	18,3	-1,15	0,00604	0,318	211231_x_at	Cytochrome P450, family 4, subfamily A, polypeptide 11	*CYP4A11*
259	7,38	8,52	-1,15	0,00122	0,257	232708_at	Galactose-1-phosphate uridylyltransferase	*GALT*
94	7,50	8,65	-1,15	0,00035	0,206	237726_at	Transcribed locus	-
1716	7,82	9,02	-1,15	0,01100	0,348	236482_at	Transmembrane channel-like 6	*TMC6*
1167	7,75	8,94	-1,15	0,00687	0,321	1565879_at	Full length insert cDNA clone YQ73D03	-
5124	30,7	35,4	-1,15	0,03944	0,421	226514_at	Zinc finger protein 71	*ZNF71*
13	4,13	4,76	-1,15	0,00005	0,191	241819_at	Tumor necrosis factor (ligand) superfamily, member 8	*TNFSF8*
1621	6,29	7,26	-1,15	0,01018	0,343	238416_x_at	Conserved nuclear protein NHN1	*NHN1*
2034	11,9	13,8	-1,15	0,01324	0,355	204095_s_at	Elongation factor RNA polymerase II	*ELL*
1613	5,37	6,19	-1,15	0,01011	0,343	1552833_at	UDP-GlcNAc:betaGal beta-1,3-N-acetylglucosaminyltransferase 6	*B3GNT6*
4601	13,7	15,8	-1,15	0,03480	0,412	219883_at	Potassium channel, subfamily K, member 4	*KCNK4*
1861	16,8	19,3	-1,15	0,01201	0,353	1555998_at	ATP synthase D chain, mitochondrial	*ATP5H*
240	5,81	6,69	-1,15	0,00111	0,250	236061_at	PR domain containing 15	*PRDM15*
114	3,63	4,19	-1,15	0,00051	0,240	241085_at	Similar to Adrenergic, alpha-1A-, receptor	-
39	9,63	11,1	-1,15	0,00016	0,201	236392_at	p53-associated parkin-like cytoplasmic protein	*PARC*
3545	13,9	16,0	-1,15	0,02550	0,393	205950_s_at	Carbonic anhydrase I	*CA1*
5839	11,9	13,7	-1,15	0,04718	0,442	209334_s_at	Proteasome (prosome, macropain) 26S subunit, non-ATPase, 9	*PSMD9*
5481	183	211	-1,15	0,04338	0,433	201342_at	Small nuclear ribonucleoprotein polypeptide C	*SNRPC*
322	4,97	5,73	-1,15	0,00161	0,272	1561916_at	GA binding protein transcription factor, alpha subunit pseudogene	*GABPAP*
3184	8,38	9,67	-1,15	0,02257	0,387	228800_x_at	Aurora kinase A interacting protein 1	*AURKAIP1*
816	25,7	29,6	-1,15	0,00466	0,312	1555478_at	Chromosome 17 open reading frame 82	*C17orf82*
2712	7,07	8,15	-1,15	0,01861	0,375	205468_s_at	Interferon regulatory factor 5	*IRF5*
5717	59,9	69,0	-1,15	0,04578	0,438	227951_s_at	Family with sequence similarity 98, member C	*FAM98C*
1063	6,88	7,93	-1,15	0,00624	0,319	223929_s_at	-	-
920	6,68	7,69	-1,15	0,00530	0,313	232590_at	cDNA FLJ33454 fis, clone BRAMY2000231	-
852	13,1	15,1	-1,15	0,00490	0,313	231151_at	Discs, large (Drosophila) homolog-associated protein 3	*DLGAP3*
232	7,56	8,70	-1,15	0,00108	0,250	211848_s_at	Carcinoembryonic antigen-related cell adhesion molecule 7	*CEACAM7*
5983	6,03	6,94	-1,15	0,04859	0,444	1555953_at	Collagen, type XVIII, alpha 1	*COL18A1*
4137	18,8	21,6	-1,15	0,03049	0,403	219620_x_at	Chromosome 9 open reading frame 167	*C9orf167*
2841	11,3	13,0	-1,15	0,01984	0,382	241825_at	Chromosome 2 open reading frame 60	*C2orf60*
4825	376	433	-1,15	0,03670	0,416	200055_at	Transcription initiation factor TFIID 30 kD subunit	*TAF10*
340	30,6	35,3	-1,15	0,00173	0,277	239607_at	G protein-coupled receptor 156	*GPR156*
1145	14,7	16,9	-1,15	0,00671	0,321	207685_at	Crystallin, beta B3	*CRYBB3*
735	7,99	9,20	-1,15	0,00419	0,308	220971_at	Interleukin 25	*IL25*
470	10,7	12,3	-1,15	0,00251	0,289	1552652_at	Hermansky-Pudlak syndrome 4	*HPS4*
2451	20,4	23,5	-1,15	0,01641	0,366	204371_s_at	KH-type splicing regulatory protein (FUSE binding protein 2)	*KHSRP*
3498	7,70	8,87	-1,15	0,02514	0,392	211211_x_at	SH2 domain protein 1A, Duncan's disease	*SH2D1A*
3597	19,9	22,9	-1,15	0,02595	0,394	207188_at	Cyclin-dependent kinase 3	*CDK3*
834	7,30	8,41	-1,15	0,00476	0,312	240818_at	Similar to Ovochymase 1	-
3381	16,7	19,2	-1,15	0,02406	0,389	217058_at	GNAS complex locus	*GNAS*
1841	8,35	9,61	-1,15	0,01183	0,351	208602_x_at	CD6 molecule	*CD6*
2210	19,1	21,9	-1,15	0,01464	0,362	230756_at	Zinc finger protein 683	*ZNF683*
3291	9,44	10,9	-1,15	0,02344	0,388	211469_s_at	Chemokine (C-X-C motif) receptor 6	*CXCR6*
3334	7,81	8,99	-1,15	0,02366	0,388	223600_s_at	KIAA1683 protein	*KIAA1683*
1724	14,5	16,6	-1,15	0,01104	0,348	227743_at	Myosin XVB pseudogene	*MYO15B*
1276	12,2	14,0	-1,15	0,00759	0,325	241166_at	Transcribed locus	-
3646	56,2	64,7	-1,15	0,02634	0,395	239053_at	Cytosolic iron-sulfur protein assembly 1 homolog (S. cerevisiae)	*CIAO1*
2231	8,12	9,34	-1,15	0,01482	0,363	224273_at	Chromosome 3 open reading frame 20	*C3orf20*
4120	30,0	34,6	-1,15	0,03028	0,402	219019_at	-	-
2523	3,41	3,92	-1,15	0,01702	0,369	232026_at	Hect domain and RLD 4	*HERC4*

Index	Mutated*	Normal*	FC	*P* value	FDR**	Probe set	Description	Gene symbol
5279	18,0	20,7	-1,15	0,04105	0,425	224466_s_at	Basic leucine zipper transcription factor MafG	*MAFG*
4990	8,96	10,3	-1,15	0,03830	0,419	216014_s_at	Zinc finger, X-linked, duplicated A	*ZXDA*
3360	5,58	6,42	-1,15	0,02384	0,388	1557567_a_at	Hypothetical protein LOC148987	*LOC148987*
815	10,7	12,3	-1,15	0,00466	0,312	1566821_at	cDNA FLJ32664 fis, clone TESTI1000088	-
1776	30,1	34,6	-1,15	0,01130	0,348	1554170_a_at	Similar to Hypothetical protein MGC49416	*LOC255374*
1438	8,82	10,1	-1,15	0,00871	0,331	221454_at	BCL2-related ovarian killer	*BOK*
3327	9,00	10,3	-1,15	0,02363	0,388	203806_s_at	Fanconi anemia, complementation group A	*FANCA*
3675	7,83	9,00	-1,15	0,02656	0,395	239248_at	(clone 33) macronuclear mRNA	-
1384	9,38	10,8	-1,15	0,00833	0,329	238655_at	Acyl-Coenzyme A dehydrogenase family, member 10	*ACAD10*
129	6,19	7,12	-1,15	0,00058	0,240	235624_at	High density lipoprotein binding protein (vigilin)	*HDLBP*
862	30,0	34,4	-1,15	0,00497	0,313	204046_at	Phospholipase C, beta 2	*PLCB2*
5820	5,66	6,51	-1,15	0,04703	0,442	229992_at	Hypothetical LOC149837	*LOC149837*
5452	10,5	12,1	-1,15	0,04301	0,431	1556422_at	cDNA FLJ33357 fis, clone BRACE2005189	-
3168	15,6	17,9	-1,15	0,02248	0,387	208594_x_at	Leukocyte immunoglobulin-like receptor subfamily A member 6	*LILRA6*
3895	8,94	10,3	-1,15	0,02846	0,399	215491_at	Myc-related gene from lung cancer	*MYCL1*
702	18,7	21,5	-1,15	0,00403	0,308	208397_x_at	Potassium inwardly-rectifying channel, subfamily J, member 5	*KCNJ5*
4027	16,8	19,3	-1,15	0,02951	0,400	214339_s_at	Mitogen-activated protein kinase kinase kinase kinase 1	*MAP4K1*
5163	16,8	19,4	-1,15	0,03978	0,421	204856_at	UDP-GlcNAc:betaGal beta-1,3-N-acetylglucosaminyltransferase 3	*B3GNT3*
1497	39,3	45,2	-1,15	0,00921	0,336	1554442_at	Bestrophin 1	*BEST1*
1019	15,8	18,2	-1,15	0,00590	0,316	221162_at	HERV-H LTR-associating 1	*HHLA1*
2968	23,3	26,8	-1,15	0,02085	0,384	218964_at	AT rich interactive domain 3B (BRIGHT-like)	*ARID3B*
1872	6,31	7,25	-1,15	0,01210	0,353	238822_at	Transcribed locus	-
2075	6,34	7,28	-1,15	0,01356	0,356	209999_x_at	Suppressor of cytokine signaling 1	*SOCS1*
2353	5,43	6,24	-1,15	0,01569	0,364	216914_at	Cell division cycle 25 homolog C (S. pombe)	*CDC25C*
4683	3,67	4,22	-1,15	0,03535	0,413	1569736_at	cDNA clone IMAGE:5264099	-
3423	10,5	12,0	-1,15	0,02447	0,391	211165_x_at	EPH receptor B2	*EPHB2*
1306	7,52	8,64	-1,15	0,00778	0,325	240784_at	Chromosome 7 open reading frame 52	*C7orf52*
5813	27,4	31,5	-1,15	0,04696	0,442	226530_at	BCL2 modifying factor	*BMF*
328	10,9	12,5	-1,15	0,00163	0,272	240664_at	Transcribed locus	-
4182	6,94	7,97	-1,15	0,03091	0,404	228626_at	Transcribed locus	-
5010	1959	2249	-1,15	0,03848	0,420	201090_x_at	Tubulin, alpha 1b	*TUBA1B*
1578	17,4	20,0	-1,15	0,00981	0,340	231378_at	Similar to XP_001095369.1	-
3395	13,7	15,7	-1,15	0,02419	0,390	235483_at	cDNA FLJ30906 fis, clone FEBRA2006055	-
1647	4,54	5,21	-1,15	0,01037	0,344	238872_at	cDNA FLJ43312 fis, clone NT2RI2012659	-
4408	12,1	13,9	-1,15	0,03290	0,408	244808_at	GRAM domain containing 1A	*GRAMD1A*
477	12,3	14,2	-1,15	0,00256	0,289	235135_at	Transcription initiation factor TFIID 30 kD subunit	*TAF10*
3678	3,32	3,82	-1,15	0,02658	0,395	215767_at	Zinc finger protein 804A	*ZNF804A*
1286	5,17	5,93	-1,15	0,00765	0,325	1564822_at	cDNA DKFZp434J2111	-
2194	3,93	4,51	-1,15	0,01452	0,361	220545_s_at	Testis-specific kinase substrate	*TSKS*
3764	3767	4324	-1,15	0,02736	0,397	212788_x_at	Ferritin, light polypeptide	*FTL*
276	4,98	5,71	-1,15	0,00133	0,263	210472_at	Metallothionein 1G	*MT1G*
1926	20,6	23,6	-1,15	0,01253	0,355	211222_s_at	huntingtin-associated protein 1 (neuroan 1)	*HAP1*
1312	5,96	6,83	-1,15	0,00782	0,325	238442_at	Hexamethylene bis-acetamide inducible 1	*HEXIM1*
2771	9,07	10,4	-1,15	0,01923	0,379	207096_at	Serum amyloid A4, constitutive	*SAA4*
4823	6,88	7,89	-1,15	0,03668	0,416	214556_at	Somatostatin receptor 4	*SSTR4*
5691	32,3	37,0	-1,15	0,04552	0,437	215903_s_at	Microtubule associated serine/threonine kinase 2	*MAST2*
5471	6,66	7,65	-1,15	0,04325	0,432	206804_at	CD3g molecule, gamma (CD3-TCR complex)	*CD3G*
858	4,43	5,08	-1,15	0,00496	0,313	1569681_at	cDNA clone IMAGE:5209417	-
135	5,54	6,35	-1,15	0,00060	0,244	1555339_at	RAP1A, member of RAS oncogene family	*RAP1A*
1116	21,7	24,9	-1,15	0,00653	0,319	37796_at	Leucine rich repeat neuronal 4	*LRCH4*
3548	5,31	6,10	-1,15	0,02552	0,393	214245_at	Ribosomal protein S14	*RPS14*
1315	9,12	10,5	-1,15	0,00783	0,325	237188_x_at	Sperm associated antigen 4-like	*SPAG4L*
5106	6,66	7,63	-1,15	0,03929	0,421	1570007_at	Leucine rich repeat containing 8 family, member C	*LRRC8C*
64	5,29	6,07	-1,15	0,00025	0,201	1561705_at	cDNA clone IMAGE:5272626	-
1955	4,48	5,13	-1,15	0,01277	0,355	207008_at	Interleukin 8 receptor, beta	*IL8RB*
3374	5,79	6,64	-1,15	0,02402	0,389	222944_s_at	MAGI family member, X-linked	*MAGIX*
1753	7,82	8,96	-1,15	0,01118	0,348	220560_at	Chromosome 11 open reading frame 21	*C11orf21*

Index	Mutated*	Normal*	FC	*P* value	FDR**	Probe set	Description	Gene symbol
4878	4,63	5,31	-1,15	0,03724	0,417	1560058_at	Hypothetical gene supported by AK093779	*LOC399900*
976	11,4	13,1	-1,15	0,00567	0,315	204311_at	ATPase, Na+/K+ transporting, beta 2 polypeptide	*ATP1B2*
602	7,41	8,49	-1,15	0,00335	0,303	204551_s_at	Alpha-2-HS-glycoprotein	*AHSG*
3149	201	231	-1,15	0,02235	0,387	213153_at	SET domain containing 1B	*SETD1B*
2387	37,8	43,3	-1,15	0,01591	0,364	1556748_x_at	cDNA FLJ39784 fis, clone SPLEN2002314	-
2375	11,9	13,6	-1,15	0,01584	0,364	231619_at	Transcribed locus	-
774	6,48	7,43	-1,15	0,00443	0,311	208321_s_at	Calcium binding protein 1	*CABP1*
2571	6,82	7,82	-1,15	0,01743	0,371	240358_at	Transcribed locus	-
4473	5,03	5,76	-1,15	0,03362	0,411	236213_at	Similar to Proteasome 26S subunit, non-ATPase, 14	-
2724	5,00	5,72	-1,15	0,01871	0,376	1556471_at	Sex comb on midleg-like 4 (Drosophila)	*SCML4*
1539	12,1	13,9	-1,15	0,00953	0,337	1569426_at	cDNA clone IMAGE:4822128	-
881	4,20	4,82	-1,15	0,00510	0,313	243396_at	Transcribed locus	-
4758	455	522	-1,15	0,03608	0,414	62987_r_at	Calcium channel, voltage-dependent, gamma subunit 4	*CACNG4*
437	4,48	5,14	-1,15	0,00231	0,288	221084_at	5-hydroxytryptamine (serotonin) receptor 3B	*HTR3B*
1587	5,44	6,23	-1,15	0,00987	0,340	234362_s_at	Cytotoxic T-lymphocyte-associated protein 4	*CTLA4*
3603	7,26	8,32	-1,15	0,02599	0,394	241314_at	Transcribed locus	-
3651	21,9	25,1	-1,15	0,02639	0,395	206220_s_at	RAS p21 protein activator 3	*RASA3*
2175	4,58	5,24	-1,15	0,01439	0,361	237513_at	Trypsin X3	*TRY1*
3639	24,1	27,6	-1,15	0,02633	0,395	226798_at	BCL2-like 13 (apoptosis facilitator)	*BCL2L13*
941	11,8	13,5	-1,15	0,00538	0,313	217184_s_at	Leukocyte tyrosine kinase	*LTK*
1888	42,6	48,8	-1,15	0,01225	0,355	1556042_s_at	Hypothetical locus LOC338799	*LOC338799*
1933	6,74	7,72	-1,15	0,01261	0,355	224547_at	Surfactant protein B-binding protein	-
110	3,31	3,79	-1,15	0,00044	0,221	216277_at	BUB1 budding uninhibited by benzimidazoles 1 homolog (yeast)	*BUB1*
3419	7,22	8,27	-1,15	0,02443	0,391	222930_s_at	Agmatine ureohydrolase (agmatinase)	*AGMAT*
1162	7,46	8,55	-1,15	0,00685	0,321	1561446_at	cDNA clone IMAGE:4829179	-
4651	8,27	9,48	-1,15	0,03510	0,413	207087_x_at	Ankyrin 1, erythrocytic	*ANK1*
2185	10,0	11,5	-1,15	0,01448	0,361	229562_at	Ribosomal protein L10a	*RPL10A*
1480	11,7	13,4	-1,15	0,00907	0,335	232933_at	KIAA1656 protein	*KIAA1656*
3110	18,6	21,3	-1,14	0,02197	0,386	221578_at	RAS association (RalGDS/AF-6) domain family 4	*RASSF4*
3201	19,5	22,4	-1,14	0,02266	0,387	220510_at	Rh family, B glycoprotein	*RHBG*
2834	6,81	7,79	-1,14	0,01979	0,382	236876_at	H1 histone family, member N, testis-specific	*H1FNT*
1410	7,52	8,61	-1,14	0,00852	0,330	1556962_at	cDNA FLJ90125 fis, clone HEMBA1007291	-
1369	8,28	9,48	-1,14	0,00823	0,328	232223_at	Clone 24863 mRNA sequence	-
1099	8,61	9,86	-1,14	0,00644	0,319	1560472_at	Hypothetical protein LOC338588	*LOC338588*
4451	5,88	6,73	-1,14	0,03343	0,411	234894_at	Inter-alpha (globulin) inhibitor H5-like	*ITIH5L*
5745	58,3	66,7	-1,14	0,04609	0,439	219538_at	WD repeat domain 5B	*WDR5B*
2078	4,58	5,24	-1,14	0,01357	0,356	233731_at	cDNA DKFZp564D123	-
4032	11,6	13,3	-1,14	0,02954	0,401	229452_at	Transmembrane protein 88	*TMEM88*
5791	6,28	7,19	-1,14	0,04668	0,441	1562247_at	Hypothetical protein LOC286058	*LOC286058*
1686	37,5	42,9	-1,14	0,01066	0,345	218475_at	HpaII tiny fragments locus 9C	*HTF9C*
3324	31,4	36,0	-1,14	0,02362	0,388	219068_x_at	ATPase family, AAA domain containing 3A	*ATAD3A*
2738	9,99	11,4	-1,14	0,01889	0,377	222245_s_at	Fer-1-like 4 (C. elegans)	*FER1L4*
5381	33,3	38,1	-1,14	0,04219	0,429	209346_s_at	Phosphatidylinositol 4-kinase type 2 alpha	*PI4K2A*
5965	4,87	5,57	-1,14	0,04844	0,444	215035_at	Immunoglobulin lambda variable 6-57	*IGLV6-57*
6083	2505	2865	-1,14	0,04966	0,446	213646_x_at	Tubulin, alpha 1b	*TUBA1B*
73	11,2	12,8	-1,14	0,00028	0,201	236719_at	Similar to XP_001086437.1	-
2441	23,6	27,0	-1,14	0,01632	0,365	238150_at	-	-
2131	32,5	37,1	-1,14	0,01401	0,359	239026_x_at	Centaurin, gamma 3	*CENTG3*
487	3,30	3,77	-1,14	0,00259	0,289	1563169_at	Clone IMAGE:4499056, mRNA	-
6023	9,88	11,3	-1,14	0,04901	0,445	207129_at	Carbonic anhydrase VB, mitochondrial	*CA5B*
2812	18,6	21,2	-1,14	0,01961	0,381	212545_s_at	Zinc fingers and homeoboxes 3	*ZHX3*
2892	11,1	12,7	-1,14	0,02030	0,383	220714_at	PR domain containing 14	*PRDM14*
2024	11,2	12,9	-1,14	0,01320	0,355	238180_at	Transcribed locus	-
3900	48,6	55,6	-1,14	0,02849	0,399	223607_x_at	Zinc finger, SWIM-type containing 1	*ZSWIM1*
5879	36,9	42,2	-1,14	0,04753	0,442	1569834_at	cDNA clone IMAGE:5295705	-
3251	10,1	11,5	-1,14	0,02308	0,388	244701_at	cDNA FLJ33712 fis, clone BRAWH2008255	-
1973	48,0	54,9	-1,14	0,01291	0,355	227237_x_at	ATPase family, AAA domain containing 3B	*ATAD3B*

Index	Mutated*	Normal*	FC	*P* value	FDR**	Probe set	Description	Gene symbol
1572	8,99	10,3	-1,14	0,00977	0,340	222712_s_at	Mucin 13, cell surface associated	*MUC13*
5900	6,76	7,73	-1,14	0,04780	0,443	242288_s_at	Elastin microfibril interfacer 2	*EMILIN2*
1023	4,93	5,64	-1,14	0,00593	0,316	228712_at	WNK lysine deficient protein kinase 1	*WNK1*
708	8,59	9,81	-1,14	0,00406	0,308	234376_at	v-Myc myelocytomatosis viral related oncogene, neuroblastoma derived (avia	*MYCN*
4395	9,31	10,6	-1,14	0,03276	0,407	1569206_at	T-complex 11 (mouse)-like 2	*TCP11L2*
647	3,92	4,49	-1,14	0,00368	0,308	217259_at	cDNA DKFZp586H0123	-
2634	7,76	8,87	-1,14	0,01794	0,372	230080_at	Homeobox A4	*HOXA4*
5682	32,6	37,2	-1,14	0,04536	0,436	1569949_at	G protein-coupled receptor kinase 5	*GRK5*
5742	21,0	24,0	-1,14	0,04607	0,439	218786_at	5'-nucleotidase domain containing 3	*NT5DC3*
2987	22,7	26,0	-1,14	0,02101	0,384	213633_at	SH3-domain binding protein 1	*SH3BP1*
878	11,4	13,1	-1,14	0,00509	0,313	234688_x_at	Centrobin, centrosomal BRCA2 interacting protein	*CNTROB*
1439	8,88	10,2	-1,14	0,00876	0,332	237036_at	F-box protein 10	*FBXO10*
765	13,3	15,2	-1,14	0,00433	0,309	243912_x_at	DNA dC->dU-editing enzyme APOBEC-3F	*APOBEC3F*
3083	5,14	5,87	-1,14	0,02173	0,385	243114_at	Transcribed locus	-
2592	9,88	11,3	-1,14	0,01760	0,371	223635_s_at	Single stranded DNA binding protein 3	*SSBP3*
2292	14,2	16,2	-1,14	0,01524	0,363	208224_at	Homeobox B1	*HOXB1*
1279	7,85	8,98	-1,14	0,00761	0,325	233467_s_at	Tetraspanin 32	*TSPAN32*
5615	12,1	13,8	-1,14	0,04479	0,436	234130_at	LIM domain binding 3	*LDB3*
1430	23,7	27,1	-1,14	0,00867	0,331	214335_at	Ribosomal protein L18	*RPL18*
1095	30,5	34,9	-1,14	0,00642	0,319	206911_at	Tripartite motif-containing 25	*TRIM25*
3987	1677	1915	-1,14	0,02915	0,400	211750_x_at	Tubulin, alpha 1c	*TUBA1C*
47	7,10	8,12	-1,14	0,00019	0,201	230725_at	-	-
953	6,81	7,79	-1,14	0,00549	0,314	233217_at	HSPC102 protein	-
104	4,20	4,80	-1,14	0,00042	0,217	241449_at	Similar to XP_001174538.1	-
4070	14,6	16,7	-1,14	0,02986	0,401	233846_at	Coiled-coil domain containing 85A	*CCDC85A*
2191	15,0	17,1	-1,14	0,01451	0,361	235799_at	Kinetochore-associated protein NSL1 homolog	*NSL1*
3643	4,92	5,62	-1,14	0,02634	0,395	239668_at	Transcribed locus	-
5778	13,2	15,1	-1,14	0,04652	0,440	220427_at	Obscurin, cytoskeletal calmodulin and titin-interacting RhoGEF	*OBSCN*
4034	14,1	16,1	-1,14	0,02955	0,401	220747_at	HSPC072 protein	*HSPC072*
4187	11,5	13,2	-1,14	0,03095	0,404	217373_x_at	Mdm2 p53 binding protein homolog (mouse)	*MDM2*
526	2,68	3,06	-1,14	0,00281	0,291	1552430_at	WD repeat domain 17	*WDR17*
1680	14,9	17,0	-1,14	0,01060	0,345	233679_at	TGF-beta-activated kinase 1-binding protein 1	*MAP3K7IP1*
1428	6,99	7,99	-1,14	0,00867	0,331	223967_at	Angiopoietin-like 6	*ANGPTL6*
3814	9,92	11,3	-1,14	0,02779	0,398	211266_s_at	G protein-coupled receptor 4	*GPR4*
3450	17,8	20,4	-1,14	0,02470	0,391	221687_s_at	Family with sequence similarity 125, member B	*FAM125B*
3744	12,2	14,0	-1,14	0,02724	0,397	207921_x_at	Paired box 8	*PAX8*
1285	8,02	9,16	-1,14	0,00765	0,325	207457_s_at	Lymphocyte antigen 6 complex, locus G6D	*LY6G6D*
1748	10,8	12,3	-1,14	0,01116	0,348	1570239_a_at	Mast cell immunoreceptor signal transducer	*MIST*
125	3,57	4,07	-1,14	0,00057	0,240	1565780_at	ATP-binding cassette, sub-family A (ABC1), member 8	*ABCA8*
2213	13,4	15,3	-1,14	0,01466	0,362	234506_at	cDNA DKFZp564M163	-
5269	24,0	27,4	-1,14	0,04093	0,425	205247_at	Notch homolog 4 (Drosophila)	*NOTCH4*
4622	12,1	13,9	-1,14	0,03488	0,412	211660_at	POU class 2 homeobox 2	*POU2F2*
2391	12,2	14,0	-1,14	0,01595	0,365	211554_s_at	Apoptotic peptidase activating factor 1	*APAF1*
3628	4,76	5,43	-1,14	0,02621	0,395	205359_at	A kinase (PRKA) anchor protein 6	*AKAP6*
2448	6,38	7,28	-1,14	0,01640	0,366	239165_at	Transcribed locus	-
952	12,7	14,5	-1,14	0,00547	0,314	232310_at	Hypothetical LOC388889	*LOC388889*
5573	16,7	19,1	-1,14	0,04439	0,435	241201_at	KIAA1530 protein	*KIAA1530*
3637	7,62	8,69	-1,14	0,02629	0,395	227343_at	Myeloma overexpressed gene (hypothetical protein LOC26579)	*MYEOV*
1367	9,41	10,7	-1,14	0,00822	0,328	241490_s_at	PiggyBac transposable element derived 2	*PGBD2*
5409	4,00	4,57	-1,14	0,04249	0,429	1558452_at	Transmembrane protein 144	*TMEM144*
1434	5,36	6,12	-1,14	0,00869	0,331	239909_at	ADAMTS-like 1	*ADAMTSL1*
3	3,29	3,76	-1,14	0,00001	0,099	243460_at	Transcribed locus	-
4106	23,5	26,8	-1,14	0,03018	0,402	216694_at	-	-
876	15,3	17,5	-1,14	0,00505	0,313	240305_at	cDNA clone IMAGE:5285563	-
6045	6,05	6,90	-1,14	0,04924	0,445	1554376_s_at	Protein-tyrosine phosphatase-like member A	*PTPLA*
535	12,2	13,9	-1,14	0,00286	0,292	220490_at	-	-
2619	12,6	14,3	-1,14	0,01780	0,371	239157_at	Zinc finger and SCAN domain containing 12-like 1	*ZSCAN12L1*

Index	Mutated*	Normal*	FC	*P* value	FDR**	Probe set	Description	Gene symbol
105	5886	6713	-1,14	0,00042	0,217	1553569_at	Phosphatidylinositol glycan anchor biosynthesis, class F	*PIGF*
2855	5,55	6,33	-1,14	0,02003	0,383	231415_at	Williams Beuren syndrome chromosome region 22	*WBSCR22*
3239	22,3	25,4	-1,14	0,02298	0,388	244157_x_at	-	-
751	5,21	5,94	-1,14	0,00425	0,308	238817_at	RIMS binding protein 2	*RIMBP2*
1580	5,66	6,45	-1,14	0,00983	0,340	206225_at	Zinc finger protein 507	*ZNF507*
775	5,42	6,18	-1,14	0,00443	0,311	241241_at	Ribosomal protein S14	*RPS14*
5214	26,5	30,2	-1,14	0,04027	0,422	234471_s_at	Nuclear receptor coactivator 5	*NCOA5*
4924	36,4	41,5	-1,14	0,03775	0,419	211512_s_at	Opioid growth factor receptor	*OGFR*
673	8,48	9,67	-1,14	0,00386	0,308	217658_at	THAP domain containing, apoptosis associated protein 3	*THAP3*
1840	11,5	13,1	-1,14	0,01183	0,351	229148_at	-	-
2381	5,51	6,28	-1,14	0,01587	0,364	205605_at	Homeobox D9	*HOXD9*
622	18,7	21,3	-1,14	0,00353	0,308	1561019_at	Full length insert cDNA clone ZD95A07	-
3061	4,99	5,69	-1,14	0,02154	0,385	204830_x_at	Pregnancy specific beta-1-glycoprotein 5	*PSG5*
1344	8,55	9,75	-1,14	0,00806	0,328	237595_at	LIM homeobox transcription factor 1, alpha	*LMX1A*
1778	17,7	20,2	-1,14	0,01132	0,348	206467_x_at	Tumor necrosis factor receptor superfamily, member 6b, decoy	*TNFRSF6B*
226	3,65	4,16	-1,14	0,00105	0,250	1565306_a_at	Solute carrier family 8, member 1	*SLC8A1*
1549	17,9	20,3	-1,14	0,00956	0,337	234647_at	Kv channel interacting protein 3, calsenilin	*KCNIP3*
3770	7,29	8,31	-1,14	0,02739	0,397	215449_at	Benzodiazapine receptor (peripheral)-like 1	*BZRPL1*
2346	9,66	11,0	-1,14	0,01566	0,364	205589_at	Myosin, light chain 3, alkali; ventricular, skeletal, slow	*MYL3*
4887	5,92	6,74	-1,14	0,03737	0,418	208360_s_at	-	-
3076	8,66	9,86	-1,14	0,02169	0,385	220684_at	T-box 21	*TBX21*
432	4,44	5,06	-1,14	0,00228	0,288	1558995_at	Zinc finger protein 547	*ZNF547*
1104	6,56	7,47	-1,14	0,00646	0,319	243344_at	Clone IMAGE:5194137, mRNA	-
3115	97,5	111	-1,14	0,02203	0,387	53912_at	Sorting nexin 11	*SNX11*
189	3,12	3,56	-1,14	0,00088	0,244	211491_at	Adrenergic, alpha-1A-, receptor	*ADRA1A*
741	7,09	8,08	-1,14	0,00421	0,308	229224_x_at	Hypothetical LOC643085	*LOC643085*
1662	7,24	8,25	-1,14	0,01049	0,345	220727_at	Potassium channel, subfamily K, member 10	*KCNK10*
791	11,9	13,6	-1,14	0,00452	0,311	206798_x_at	Deleted in lung and esophageal cancer 1	*DLEC1*
224	11,6	13,2	-1,14	0,00104	0,250	220379_at	Fascin homolog 3, actin-bundling protein, testicular	*FSCN3*
2235	8,64	9,84	-1,14	0,01485	0,363	233137_at	Clone IMAGE:121687 mRNA sequence	-
1997	4,54	5,17	-1,14	0,01306	0,355	217677_at	Pleckstrin homology domain-containing family A member 2	*PLEKHA2*
2003	19,8	22,6	-1,14	0,01307	0,355	1555359_at	Phosphatidylinositol glycan anchor biosynthesis, class Q	*PIGQ*
2146	5,76	6,56	-1,14	0,01412	0,360	229993_at	Zinc finger, CCHC domain containing 3	*ZCCHC3*
428	8,07	9,19	-1,14	0,00226	0,288	232577_at	Hypothetical protein LOC145945	*LOC145945*
222	8,62	9,81	-1,14	0,00103	0,250	231893_at	KIAA1755 protein	*KIAA1755*
2040	4,57	5,20	-1,14	0,01328	0,355	209738_x_at	Pregnancy specific beta-1-glycoprotein 6	*PSG6*
1453	4,04	4,60	-1,14	0,00889	0,334	1570650_at	Cysteine conjugate-beta lyase; cytoplasmic	*CCBL1*
1609	5,99	6,82	-1,14	0,01008	0,342	234811_at	Centromere protein N	*CENPN*
5638	50,6	57,6	-1,14	0,04499	0,436	202140_s_at	CDC-like kinase 3	*CLK3*
1120	27,8	31,6	-1,14	0,00654	0,319	239720_at	Similar to RIKEN cDNA B230118G17 gene	*MGC19604*
1361	10,3	11,7	-1,14	0,00820	0,328	234499_at	cDNA DKFZp566K203	-
2790	10,2	11,6	-1,14	0,01940	0,380	220476_s_at	Chromosome 1 open reading frame 183	*C1orf183*
867	16,6	18,9	-1,14	0,00501	0,313	231743_at	Wingless-type MMTV integration site family, member 3	*WNT3*
2088	5,61	6,38	-1,14	0,01362	0,356	237324_s_at	Hexokinase domain containing 1	*HKDC1*
2099	6,05	6,88	-1,14	0,01375	0,358	236852_at	F-box protein 43	*FBXO43*
1820	23,9	27,1	-1,14	0,01172	0,351	1555754_s_at	Atrophin 1	*ATN1*
3299	6,02	6,85	-1,14	0,02350	0,388	231877_at	RNA (guanine-9-) methyltransferase domain containing 2	*RG9MTD2*
6037	8,06	9,17	-1,14	0,04915	0,445	234696_at	Similar to Testicular serine protease 2	*LOC138652*
3380	3,55	4,04	-1,14	0,02405	0,389	210531_at	Nuclear receptor subfamily 2, group C, member 1	*NR2C1*
4122	6,96	7,92	-1,14	0,03031	0,402	229548_at	Similar to UNC-84 homolog B	-
889	5,48	6,24	-1,14	0,00513	0,313	237599_at	Similar to Finger protein 286	-
2599	11,8	13,5	-1,14	0,01765	0,371	1561133_at	Full length insert cDNA clone ZD60A10	-
1048	11,8	13,5	-1,14	0,00612	0,319	1562256_at	NLR family, pyrin domain containing 1	*NLRP1*
3848	163	185	-1,14	0,02810	0,399	201805_at	Protein kinase, AMP-activated, gamma 1 non-catalytic subunit	*PRKAG1*
3009	10,1	11,5	-1,14	0,02118	0,385	241820_at	RAP1 interacting factor homolog (yeast)	*RIF1*
2456	10,6	12,0	-1,14	0,01647	0,366	224498_x_at	Axin 2 (conductin, axil)	*AXIN2*
1822	11,0	12,5	-1,14	0,01173	0,351	232400_at	SEC14-like 2 (S. cerevisiae)	*SEC14L2*

Index	Mutated*	Normal*	FC	*P* value	FDR**	Probe set	Description	Gene symbol
3335	39,3	44,7	-1,14	0,02366	0,388	201796_s_at	Valyl-tRNA synthetase	*VARS*
4505	3,16	3,59	-1,14	0,03391	0,412	236575_at	cDNA clone IMAGE:4823793	-
1406	4,46	5,07	-1,14	0,00850	0,330	1556536_at	HCG2044152 protein	-
786	6,18	7,03	-1,14	0,00449	0,311	1565580_s_at	TatD DNase domain containing 2	*TATDN2*
163	26,1	29,7	-1,14	0,00075	0,244	210043_at	FERM domain containing 8	*FRMD8*
1378	19,2	21,9	-1,14	0,00830	0,329	240587_x_at	Transcribed locus	-
4110	5,61	6,38	-1,14	0,03021	0,402	223987_at	Chordin-like 2	*CHRDL2*
1830	4,63	5,26	-1,14	0,01179	0,351	231051_at	Full length insert cDNA clone ZD51E06	-
4802	40,6	46,2	-1,14	0,03644	0,415	89476_r_at	Aminopeptidase-like 1	*NPEPL1*
4975	9,94	11,3	-1,14	0,03818	0,419	211900_x_at	CD6 molecule	*CD6*
1779	30,8	35,0	-1,14	0,01134	0,349	241918_at	Transmembrane protein 16G	*TMEM16G*
758	6,49	7,37	-1,14	0,00428	0,308	243058_at	-	-
3244	8,02	9,11	-1,14	0,02300	0,388	222301_at	Chromosome 1 open reading frame 61	*C1orf61*
3203	8,26	9,39	-1,14	0,02268	0,387	1554382_at	Hypothetical protein LOC200261	*LOC200261*
5713	8,05	9,15	-1,14	0,04574	0,438	235863_at	Junctional sarcoplasmic reticulum protein 1	*JSRP1*
1522	6,54	7,44	-1,14	0,00940	0,337	239947_at	Transcribed locus	-
124	7,37	8,37	-1,14	0,00057	0,240	242651_at	Transcribed locus	-
2167	4,35	4,94	-1,14	0,01434	0,361	219393_s_at	v-AKT murine thymoma viral oncogene homolog	*AKT3*
5401	5,34	6,07	-1,14	0,04236	0,429	230170_at	Oncostatin M	*OSM*
3594	58,0	65,9	-1,14	0,02592	0,394	202958_at	Protein tyrosine phosphatase, non-receptor type 9	*PTPN9*
1987	4,28	4,87	-1,14	0,01298	0,355	1555626_a_at	Signaling lymphocytic activation molecule family member 1	*SLAMF1*
1417	3,57	4,06	-1,14	0,00858	0,331	223550_s_at	Carbonic anhydrase X	*CA10*
776	4,59	5,22	-1,14	0,00443	0,311	216471_x_at	Synovial sarcoma, X breakpoint 2	*SSX2*
3233	7,37	8,38	-1,14	0,02291	0,388	1561960_at	Hypothetical gene supported by AB067507	*LOC390627*
3722	25,2	28,7	-1,14	0,02702	0,397	221306_at	G protein-coupled receptor 27	*GPR27*
2216	7,68	8,73	-1,14	0,01468	0,362	1570133_at	Clone IMAGE:5172579, mRNA	-
811	11,0	12,5	-1,14	0,00462	0,311	241437_s_at	EP400 N-terminal like	*EP400NL*
1839	4,57	5,19	-1,14	0,01183	0,351	231589_at	-	-
3060	16,5	18,8	-1,14	0,02154	0,385	207004_at	B-cell CLL/lymphoma 2	*BCL2*
2042	17,0	19,3	-1,14	0,01328	0,355	1559629_at	Clone IMAGE:5744974, mRNA	-
782	17,7	20,1	-1,14	0,00448	0,311	1559461_at	Clone IMAGE:3852236, mRNA	-
491	3,43	3,89	-1,14	0,00262	0,289	1569746_s_at	cDNA clone IMAGE:5265020	-
3803	14,8	16,9	-1,14	0,02770	0,398	1568743_at	ATPase, Class V, type 10A	*ATP10A*
1659	6,12	6,96	-1,14	0,01048	0,345	1553757_at	IQ motif containing G	*IQCG*
3447	35,2	40,0	-1,14	0,02467	0,391	1556479_at	cDNA FLJ40474 fis, clone TESTI2042928	-
3560	4,90	5,57	-1,14	0,02570	0,394	222520_s_at	Intraflagellar transport 57 homolog (Chlamydomonas)	*IFT57*
728	4,43	5,03	-1,14	0,00416	0,308	1561221_x_at	Hypothetical protein LOC728099	*LOC728099*
3476	7,27	8,25	-1,14	0,02496	0,392	206353_at	Cytochrome c oxidase subunit VIa polypeptide 2	*COX6A2*
2091	6,50	7,39	-1,14	0,01365	0,357	240590_at	Hypothetical protein LOC348761	*LOC348761*
503	4,79	5,44	-1,14	0,00267	0,289	239308_at	Dual-specificity tyrosine-(Y)-phosphorylation regulated kinase 1A	*DYRK1A*
2102	33,6	38,1	-1,14	0,01378	0,358	213323_s_at	Zinc finger CCCH-type containing 7B	*ZC3H7B*
5239	7,25	8,23	-1,14	0,04059	0,423	229622_at	Family with sequence similarity 132, member B	*FAM132B*
1134	17,3	19,7	-1,14	0,00663	0,319	1568667_s_at	Lipid storage droplet protein 5	*LSDP5*
3490	3,98	4,51	-1,14	0,02509	0,392	244489_at	-	-
4609	7,10	8,06	-1,14	0,03482	0,412	232464_at	Tripartite motif-containing pseudogene 1	*TRIMP1*
527	5,26	5,97	-1,14	0,00281	0,291	220227_at	Cadhetrin 4, type 1, R-Cadhetrin (retinal)	*CDH4*
5627	7,76	8,80	-1,14	0,04492	0,436	223510_at	Neuropilin 2	*NRP2*
677	5,88	6,67	-1,14	0,00389	0,308	220343_at	Phosphodiesterase 7B	*PDE7B*
2389	12,4	14,1	-1,13	0,01592	0,364	210442_at	Interleukin 1 receptor-like 1	*IL1RL1*
2336	5,19	5,89	-1,13	0,01559	0,364	216747_at	Amyloid beta precursor protein-binding, family B, member 2	*APBB2*
4225	9,61	10,9	-1,13	0,03132	0,405	1564373_a_at	Hypothetical protein LOC283887	*LOC283887*
5184	4,51	5,12	-1,13	0,03996	0,421	235325_at	Spastic paraplegia 7 protein	*SPG7*
382	2,90	3,29	-1,13	0,00199	0,284	1552765_x_at	Transmembrane protein 67	*TMEM67*
5259	24,4	27,7	-1,13	0,04080	0,424	206926_s_at	Interleukin 11	*IL11*
5893	51,7	58,7	-1,13	0,04767	0,442	227035_x_at	Retinitis pigmentosa 9 pseudogene	*LOC441212*
5140	6,07	6,89	-1,13	0,03958	0,421	216327_s_at	Sialic acid binding Ig-like lectin 8	*SIGLEC8*
5583	3,18	3,60	-1,13	0,04452	0,436	235230_at	cDNA FLJ34317 fis, clone FEBRA2008475	-

Index	Mutated*	Normal*	FC	*P* value	FDR**	Probe set	Description	Gene symbol
2921	5,29	6,01	-1,13	0,02049	0,383	220095_at	Chromosome 9 open reading frame 39	*C9orf39*
1893	6,19	7,02	-1,13	0,01231	0,355	216117_at	Exosome component 2	*EXOSC2*
4976	12,0	13,6	-1,13	0,03819	0,419	239415_at	Microtubule-associated protein 9	*MAP9*
3491	8,49	9,63	-1,13	0,02510	0,392	243083_at	Hypothetical gene supported by BC040831	*LOC401351*
2356	6,55	7,43	-1,13	0,01572	0,364	238014_at	-	-
4853	12,0	13,6	-1,13	0,03698	0,417	1567257_at	Olfactory receptor, family 1, subfamily J, member 2	*OR1J2*
2932	11,0	12,5	-1,13	0,02056	0,383	234099_at	cDNA FLJ13494 fis, clone PLACE1004384	-
1679	7,80	8,84	-1,13	0,01060	0,345	235865_at	CUG triplet repeat, RNA binding protein 1	*CUGBP1*
3161	14,0	15,9	-1,13	0,02243	0,387	232988_at	KIAA0182 protein	*KIAA0182*
3158	7,53	8,54	-1,13	0,02241	0,387	206178_at	Phospholipase A2, group V	*PLA2G5*
2559	7,22	8,19	-1,13	0,01736	0,371	1556453_at	cDNA FLJ35971 fis, clone TESTI2013257	-
507	6,99	7,93	-1,13	0,00269	0,289	1562818_at	Nicotinamide nucleotide adenylyltransferase 2	*NMNAT2*
164	10,7	12,2	-1,13	0,00076	0,244	1554759_at	SNAP25-interacting protein	*SNIP*
1444	12,5	14,2	-1,13	0,00878	0,332	217622_at	Rhomboid domain containing 3	*RHBDD3*
2604	10,5	11,9	-1,13	0,01768	0,371	206802_at	Paired box 5	*PAX5*
4881	18,1	20,5	-1,13	0,03733	0,418	229668_at	Hypothetical protein LOC90393	*LOC90393*
3968	4,04	4,58	-1,13	0,02898	0,399	236660_at	cDNA FLJ45600 fis, clone BRTHA3020369	-
3515	23,2	26,3	-1,13	0,02525	0,392	1553444_a_at	Chromosome 1 open reading frame 127	*C1orf127*
4829	66,3	75,1	-1,13	0,03679	0,416	221956_at	Leucine rich repeat neuronal 4	*LRCH4*
1274	6,11	6,93	-1,13	0,00758	0,325	1553936_a_at	Hypothetical protein MGC2848	*MGC2848*
5177	10,7	12,1	-1,13	0,03990	0,421	218600_at	LIM domain containing 2	*LIMD2*
1127	20,4	23,1	-1,13	0,00657	0,319	242145_at	Transcribed locus	-
635	3,10	3,52	-1,13	0,00361	0,308	240138_at	Transcribed locus	-
650	6,66	7,55	-1,13	0,00369	0,308	1565559_at	MAGI family member, X-linked	*MAGIX*
1817	5,90	6,69	-1,13	0,01171	0,351	1563045_at	cDNA clone IMAGE:4827232	-
1347	17,5	19,8	-1,13	0,00807	0,328	211791_s_at	Potassium channel shaker chain beta 2	*KCNAB2*
2499	4,80	5,44	-1,13	0,01684	0,367	243238_at	Phosphorylase, glycogen; brain	*PYGB*
4554	6,32	7,16	-1,13	0,03442	0,412	230827_at	Similar to HBxAg transactivated protein 2 isoform 5	-
4774	7,27	8,23	-1,13	0,03621	0,414	207346_at	Syntaxin 2	*STX2*
1353	5,82	6,59	-1,13	0,00812	0,328	221380_at	Mahlavu hepatocellular carcinoma	*HHCM*
12	2,95	3,34	-1,13	0,00004	0,180	242682_at	-	-
415	3,55	4,02	-1,13	0,00219	0,286	230910_s_at	Lymphocyte antigen 6 complex, locus K	*LY6K*
3430	34,9	39,5	-1,13	0,02455	0,391	223462_at	Transmembrane protein 175	*TMEM175*
1804	12,4	14,0	-1,13	0,01162	0,351	208489_at	Gap junction protein, alpha 8, 50kDa	*GJA8*
3706	10,3	11,6	-1,13	0,02680	0,395	206892_at	Anti-Mullerian hormone receptor, type II	*AMHR2*
3757	23,0	26,0	-1,13	0,02731	0,397	224125_at	Pleckstrin homology domain containing, family N member 1	*PLEKHN1*
1002	4,93	5,58	-1,13	0,00579	0,316	1560862_at	cDNA DKFZp434I235	-
1731	6,98	7,91	-1,13	0,01105	0,348	241725_at	E2F transcription factor 7	*E2F7*
1671	20,5	23,2	-1,13	0,01055	0,345	209559_at	Huntingtin interacting protein 1 related	*HIP1R*
2726	13,1	14,8	-1,13	0,01874	0,376	231360_at	Chromosome 20 open reading frame 141	*C20orf141*
348	11,1	12,5	-1,13	0,00178	0,278	244152_at	Transcribed locus	-
2402	6,36	7,20	-1,13	0,01602	0,365	211195_s_at	Tumor protein p63	*TP63*
4341	7,62	8,63	-1,13	0,03233	0,407	208191_x_at	Pregnancy specific beta-1-glycoprotein 4	*PSG4*
5431	33,2	37,6	-1,13	0,04279	0,431	219997_s_at	COP9 constitutive photomorphogenic homolog subunit 7B	*COPS7B*
4154	10,8	12,2	-1,13	0,03064	0,403	216892_at	Immunoglobulin heavy constant gamma 1 (G1m marker)	*IGHG1*
295	3,73	4,23	-1,13	0,00144	0,263	1562983_at	Hypothetical gene supported by BC039667	*LOC441025*
4048	7,17	8,12	-1,13	0,02967	0,401	206154_at	Retinaldehyde binding protein 1	*RLBP1*
652	3,43	3,88	-1,13	0,00371	0,308	235777_at	Ankyrin repeat domain 44	*ANKRD44*
2943	7,51	8,50	-1,13	0,02063	0,383	243429_at	Transcribed locus	-
1372	4,07	4,61	-1,13	0,00824	0,328	243073_at	Transcribed locus	-
5354	161	183	-1,13	0,04201	0,429	215399_s_at	Amplified in osteosarcoma	*OS9*
2337	6,34	7,17	-1,13	0,01560	0,364	237715_at	AT-hook transcription factor	*AKNA*
1122	5,16	5,84	-1,13	0,00655	0,319	236171_at	ATPase, Ca++ transporting, type 2C, member 2	*ATP2C2*
759	8,24	9,32	-1,13	0,00429	0,308	219144_at	Dual specificity phosphatase 26 (putative)	*DUSP26*
83	4,35	4,92	-1,13	0,00031	0,201	229791_at	Acyltransferase like 1	*AYTL1*
1468	7,96	9,01	-1,13	0,00898	0,334	237151_s_at	Similar to Hypothetical protein	*MGC33657*
2413	26,3	29,8	-1,13	0,01612	0,365	230345_at	Transcribed locus	-

Index	Mutated*	Normal*	FC	*P* value	FDR**	Probe set	Description	Gene symbol
1368	7,93	8,97	-1,13	0,00823	0,328	232813_s_at	Golgin-like protein	*GOLGA*
5321	34,0	38,4	-1,13	0,04158	0,427	1552863_a_at	Calcium channel, voltage-dependent, gamma subunit 6	*CACNG6*
1373	3,55	4,01	-1,13	0,00825	0,329	220834_at	Membrane-spanning 4-domains, subfamily A, member 12	*MS4A12*
377	4,99	5,65	-1,13	0,00196	0,284	1561472_at	Hypothetical protein LOC728805	*LOC728805*
3017	18,4	20,8	-1,13	0,02126	0,385	47105_at	Dihydrouridine synthase 2-like, SMM1 homolog (S. cerevisiae)	*DUS2L*
2513	18,4	20,8	-1,13	0,01691	0,368	220573_at	Kallikrein-related peptidase 14	*KLK14*
3684	13,0	14,7	-1,13	0,02660	0,395	1552845_at	Claudin 15	*CLDN15*
3333	39,4	44,6	-1,13	0,02366	0,388	205021_s_at	Forkhead box N3	*FOXN3*
787	25,5	28,8	-1,13	0,00450	0,311	1561355_at	Hypothetical locus LOC692247	*LOC692247*
5441	16,5	18,7	-1,13	0,04288	0,431	206807_s_at	Adducin 2 (beta)	*ADD2*
1224	9,22	10,4	-1,13	0,00729	0,324	211873_s_at	¨Protocadherin gamma subfamily A, 9	*PCDHGA9*
985	26,2	29,6	-1,13	0,00569	0,315	234956_at	Hypothetical protein LOC93444	*LOC93444*
1649	4,83	5,47	-1,13	0,01037	0,344	221360_s_at	Growth hormone secretagogue receptor	*GHSR*
38	3,48	3,93	-1,13	0,00015	0,201	1561353_at	cDNA clone IMAGE:4816369	-
1142	17,4	19,6	-1,13	0,00669	0,320	233378_at	Hypothetical protein LOC731419	*LOC731419*
1946	8,06	9,11	-1,13	0,01272	0,355	228689_at	NADH-ubiquinone oxidoreductase subunit B14.7	*NDUFA11*
4565	28,0	31,7	-1,13	0,03451	0,412	205331_s_at	Receptor accessory protein 2	*REEP2*
2198	5,26	5,95	-1,13	0,01453	0,361	215124_at	Zinc finger protein 550	*ZNF550*
2431	5,27	5,96	-1,13	0,01625	0,365	243043_at	Transcribed locus	-
3304	5,66	6,39	-1,13	0,02351	0,388	241516_at	-	-
5564	10,9	12,3	-1,13	0,04430	0,435	211263_s_at	Proprotein convertase subtilisin/kexin type 6	*PCSK6*
2893	5,38	6,08	-1,13	0,02031	0,383	237913_at	Transcribed locus	-
3735	3,46	3,92	-1,13	0,02712	0,397	1561430_s_at	Chromosome 3 open reading frame 15	*C3orf15*
3262	8,92	10,1	-1,13	0,02316	0,388	232787_at	PPAR-alpha-interacting complex protein 285	*PRIC285*
2149	24,2	27,3	-1,13	0,01415	0,360	1563273_at	Full length insert cDNA clone ZA89G05	-
4309	24,6	27,8	-1,13	0,03208	0,407	215670_s_at	SCAN domain containing 2	*SCAND2*
4876	5,21	5,89	-1,13	0,03723	0,417	234965_at	Apoptosis related protein APR-4	-
4071	16,3	18,4	-1,13	0,02986	0,401	231255_at	Myosin phosphatase-Rho interacting protein	*M-RIP*
1403	19,0	21,5	-1,13	0,00847	0,330	206444_at	Phosphodiesterase 1B, calmodulin-dependent	*PDE1B*
3429	10,8	12,2	-1,13	0,02455	0,391	222515_x_at	Transmembrane protein 165	*TMEM165*
1979	7,86	8,89	-1,13	0,01292	0,355	221083_at	Potassium voltage-gated channel, KQT-like subfamily, member 4	*KCNQ4*
65	4,03	4,55	-1,13	0,00025	0,201	1566500_at	-	-
5802	17,6	19,9	-1,13	0,04682	0,441	226469_s_at	Gamma-glutamyltransferase-like 3	*GGTL3*
2301	10,1	11,4	-1,13	0,01528	0,363	241849_at	Transcribed locus	-
5414	8,16	9,22	-1,13	0,04255	0,430	238887_at	Clone IMAGE:3901628, mRNA	-
2319	17,8	20,1	-1,13	0,01541	0,363	1553399_a_at	Chromosome 17 open reading frame 69	*C17orf69*
502	3,31	3,74	-1,13	0,00267	0,289	235906_at	Transcription initiation factor TFIID 30 kD subunit	*TAF10*
1514	4,44	5,01	-1,13	0,00936	0,337	240540_at	-	-
283	7,67	8,66	-1,13	0,00139	0,263	230801_at	Chromosome 20 open reading frame 77	*C20orf77*
1199	11,4	12,9	-1,13	0,00708	0,323	214896_at	mRNA full length insert cDNA clone EUROIMAGE 29222	-
1738	4,53	5,12	-1,13	0,01110	0,348	1558021_at	RAB9 effector protein with kelch motifs	*RABEPK*
275	6,30	7,12	-1,13	0,00132	0,262	1562689_at	Hypothetical protein LOC151484	*LOC151484*
5817	14,1	15,9	-1,13	0,04701	0,442	1570295_at	Clone IMAGE:6059689, mRNA	-
3612	5,72	6,46	-1,13	0,02607	0,394	230924_at	Tubulin tyrosine ligase-like family, member 6	*TTLL6*
2529	10,4	11,8	-1,13	0,01708	0,369	1554219_at	-	-
5394	2100	2371	-1,13	0,04230	0,429	212639_x_at	Tubulin, alpha 1b	*TUBA1B*
762	4,02	4,54	-1,13	0,00430	0,308	220288_at	Myosin XVA	*MYO15A*
1111	17,1	19,3	-1,13	0,00650	0,319	243138_at	Transcribed locus	-
3086	7,72	8,72	-1,13	0,02176	0,385	236568_at	Coiled-coil and C2 domain containing 1B	*CC2D1B*
1869	13,5	15,2	-1,13	0,01207	0,353	233761_at	cDNA DKFZp762F0616	-
4352	37,3	42,1	-1,13	0,03241	0,407	223153_x_at	Transmembrane and ubiquitin-like domain containing 1	*TMUB1*
2844	4,67	5,28	-1,13	0,01989	0,382	233069_at	Protein phosphatase 4, regulatory subunit 1-like	*PPP4R1L*
4750	9,37	10,6	-1,13	0,03600	0,414	241326_at	Adenylate kinase 7	*AK7*
1945	6,66	7,52	-1,13	0,01267	0,355	240347_at	Transcribed locus	-
373	5,12	5,78	-1,13	0,00196	0,284	233188_at	Polypeptide GalNAc transferase-like protein 4	*GALNTL4*
3098	8,06	9,10	-1,13	0,02187	0,386	230662_at	Ring finger protein 187	*RNF187*
50	5984	6754	-1,13	0,00020	0,201	1553570_x_at	Phosphatidylinositol glycan anchor biosynthesis, class F	*PIGF*

Index	Mutated*	Normal*	FC	*P* value	FDR**	Probe set	Description	Gene symbol
5974	69,4	78,3	-1,13	0,04852	0,444	202556_s_at	Microspherule protein 1	*MCRS1*
562	5,03	5,67	-1,13	0,00306	0,298	1564203_at	Hypothetical protein LOC147004	*LOC147004*
285	3,37	3,80	-1,13	0,00139	0,263	1566734_at	Hypothetical protein LOC283454	*LOC283454*
2009	6,88	7,77	-1,13	0,01310	0,355	1560513_at	Hypothetical LOC400568	*LOC400568*
4422	9,17	10,3	-1,13	0,03310	0,409	232435_at	Chromosome X open reading frame 45	*CXorf45*
3343	18,7	21,1	-1,13	0,02374	0,388	229445_at	Cytochrome b-245, alpha polypeptide	*CYBA*
4521	8,93	10,1	-1,13	0,03410	0,412	1570531_at	cDNA clone IMAGE:4344985	-
1290	32,2	36,3	-1,13	0,00769	0,325	1565611_at	Dynein, light chain, roadblock-type 1	*DYNLRB1*
3064	35,9	40,5	-1,13	0,02160	0,385	205313_at	Transcription factor 2, hepatic; LF-B3	*TCF2*
4634	15,1	17,1	-1,13	0,03495	0,412	214311_at	Zinc finger protein-like 1	*ZFPL1*
1151	4,75	5,36	-1,13	0,00677	0,321	238273_at	Solute carrier family 13, member 4	*SLC13A4*
144	23,8	26,9	-1,13	0,00065	0,244	1553136_at	Potassium channel tetramerisation domain containing 18	*KCTD18*
2312	7,86	8,86	-1,13	0,01536	0,363	234914_at	Zinc finger protein 7	*ZNF7*
5889	16,1	18,1	-1,13	0,04764	0,442	243261_at	Hypothetical protein LOC154822	*LOC154822*
5072	8,06	9,10	-1,13	0,03903	0,420	230297_x_at	Synaptic Ras GTPase activating protein 1 homolog (rat)	*SYNGAP1*
5532	8,39	9,46	-1,13	0,04392	0,434	208240_s_at	Fibroblast growth factor 1 (acidic)	*FGF1*
3026	33,0	37,2	-1,13	0,02129	0,385	55583_at	Dedicator of cytokinesis 6	*DOCK6*
5970	2563	2891	-1,13	0,04848	0,444	211058_x_at	Tubulin, alpha 1b	*TUBA1B*
1948	4,52	5,09	-1,13	0,01272	0,355	236183_at	Dynein, cytoplasmic 1, heavy chain 1	*DYNC1H1*
2990	5,05	5,69	-1,13	0,02102	0,384	206162_x_at	Synaptotagmin V	*SYT5*
1153	16,6	18,7	-1,13	0,00678	0,321	1557723_at	Hypothetical protein LOC285847	*LOC285847*
4891	4,61	5,20	-1,13	0,03738	0,418	223951_at	Chromosome 21 open reading frame 116	*C21orf116*
4262	7,59	8,56	-1,13	0,03168	0,406	210197_at	Inositol 1,3,4-triphosphate 5/6 kinase	*ITPK1*
5135	6,45	7,28	-1,13	0,03950	0,421	222797_at	Dihydropyrimidinase-like 5	*DPYSL5*
2591	64,4	72,6	-1,13	0,01758	0,371	1562022_s_at	RAD9 homolog A (S. pombe)	*RAD9A*
906	14,0	15,7	-1,13	0,00524	0,313	239838_at	Zinc finger protein 776	*ZNF776*
1688	4,28	4,83	-1,13	0,01067	0,345	208514_at	Potassium voltage-gated channel, Isk-related family, member 1	*KCNE1*
585	6,09	6,86	-1,13	0,00323	0,301	230983_at	Family with sequence similarity 129, member C	*FAM129C*
2814	6,50	7,33	-1,13	0,01963	0,381	213777_s_at	-	-
4605	39,6	44,6	-1,13	0,03480	0,412	225360_at	TraB domain containing	*TRABD*
5606	1750	1973	-1,13	0,04471	0,436	209251_x_at	Tubulin, alpha 1c	*TUBA1C*
1215	4,61	5,20	-1,13	0,00722	0,324	234931_at	AYP1 pseudogene 1	*AYP1p1*
3353	7,60	8,57	-1,13	0,02382	0,388	211403_x_at	Variable charge, Y-linked	*VCY*
4403	3,81	4,30	-1,13	0,03287	0,408	1552518_s_at	MDM2 (mouse double minute 2)-binding protein, 104kD	*MTBP*
619	6,95	7,83	-1,13	0,00350	0,308	1556994_at	cDNA clone IMAGE:5296604	-
5835	12,6	14,2	-1,13	0,04714	0,442	220207_at	Hypothetical protein LOC541469	*LOC541469*
5694	14,5	16,3	-1,13	0,04556	0,437	210752_s_at	MAX-like protein X	*MLX*
1424	3,96	4,46	-1,13	0,00863	0,331	207285_x_at	Chorionic somatomammotropin hormone-like 1	*CSHL1*
2350	3,89	4,38	-1,13	0,01568	0,364	216569_at	-	-
1432	4,82	5,43	-1,13	0,00868	0,331	234553_at	cDNA FLJ20012 fis, clone ADKA03438	-
4368	15,1	17,0	-1,13	0,03249	0,407	230853_at	Similar to XP_529987.1	-
4485	11,4	12,8	-1,13	0,03374	0,411	229489_at	Transcribed locus	-
4235	13,3	15,0	-1,13	0,03139	0,405	239821_at	Hypothetical protein LOC644975	*FLJ30064*
1030	5,61	6,32	-1,13	0,00597	0,317	206760_s_at	Fc fragment of IgE, low affinity II, receptor for (CD23)	*FCER2*
1365	3,91	4,40	-1,13	0,00822	0,328	211108_s_at	Janus kinase 3 (a protein tyrosine kinase, leukocyte)	*JAK3*
2052	9,27	10,4	-1,13	0,01334	0,355	1553248_at	Coiled-coil domain containing 57	*CCDC57*
5676	107	121	-1,13	0,04530	0,436	201640_x_at	Cleft lip and palate associated transmembrane protein 1	*CLPTM1*
2208	7,47	8,41	-1,13	0,01461	0,362	1553168_at	Glutamate receptor, ionotropic, kainate 5	*GRIK5*
1975	8,04	9,05	-1,13	0,01291	0,355	220571_at	PR domain containing 11	*PRDM11*
4476	4,74	5,34	-1,13	0,03365	0,411	1553478_at	Proline rich 10	*PRR10*
5830	10,1	11,3	-1,13	0,04712	0,442	232042_at	Tweety homolog 2 (Drosophila)	*TTYH2*
3282	13,4	15,0	-1,13	0,02339	0,388	231922_at	Zinc finger protein 276	*ZNF276*
3068	5,71	6,43	-1,13	0,02165	0,385	243719_at	Serine/threonine kinase 19	*STK19*
5257	13,3	14,9	-1,13	0,04075	0,424	235375_x_at	Tetratricopeptide repeat domain 9B	*TTC9B*
505	4,25	4,79	-1,13	0,00268	0,289	206803_at	Prodynorphin	*PDYN*
1147	12,8	14,4	-1,13	0,00674	0,321	1556727_at	Progressive rod-cone degeneration	*PRCD*
3870	9,54	10,7	-1,13	0,02828	0,399	239074_at	GRB2-related adaptor protein-like	*LOC400581*

Index	Mutated*	Normal*	FC	*P* value	FDR**	Probe set	Description	Gene symbol
1349	5,95	6,70	-1,13	0,00810	0,328	234328_at	-	-
5876	9,87	11,1	-1,13	0,04747	0,442	237092_at	Transcribed locus	-
3210	5,16	5,81	-1,13	0,02273	0,387	1555039_a_at	ATP-binding cassette, sub-family C (CFTR/MRP), member 4	*ABCC4*
5032	10,9	12,2	-1,13	0,03868	0,420	216789_at	cDNA FLJ22786 fis, clone KAIA2150	-
2253	25,9	29,1	-1,13	0,01500	0,363	232892_at	Chromosome 20 open reading frame 166	*C20orf166*
5484	408	459	-1,13	0,04340	0,433	200714_x_at	Amplified in osteosarcoma	*OS9*
3014	7,56	8,51	-1,13	0,02121	0,385	229474_at	Flavoprotein oxidoreductase MICAL3	*MICAL3*
3956	5,87	6,61	-1,13	0,02889	0,399	240406_at	Similar to Ubiquitin specific protease 16 isoform a	-
1029	10,1	11,4	-1,13	0,00596	0,317	224543_at	Selectin-like osteoblast-derived protein	*SVEP1*
657	7,81	8,79	-1,13	0,00380	0,308	214233_at	ADP-ribosylation factor-binding protein GGA2	*GGA2*
4405	87,3	98,3	-1,13	0,03288	0,408	396_f_at	Erythropoietin receptor	*EPOR*
4931	4,78	5,38	-1,13	0,03782	0,419	1557720_s_at	Myosin XVI	*MYO16*
1235	3,49	3,93	-1,13	0,00734	0,324	203170_at	KIAA0409 protein	*KIAA0409*
4702	15,0	16,8	-1,13	0,03550	0,413	1567255_at	Olfactory receptor, family 10, subfamily D, member 1 pseudogene	*OR10D1P*
2293	7,98	8,98	-1,13	0,01524	0,363	233143_at	Chromosome 20 open reading frame 173	*C20orf173*
2048	4,82	5,42	-1,13	0,01331	0,355	211743_s_at	Proteoglycan 2, bone marrow	*PRG2*
757	3,91	4,40	-1,13	0,00428	0,308	228715_at	Zinc finger, CCHC domain containing 12	*ZCCHC12*
1204	5,38	6,05	-1,13	0,00713	0,324	222007_s_at	FK506 binding protein 8, 38kDa	*FKBP8*
5756	4,45	5,01	-1,13	0,04621	0,439	220399_at	Hypothetical protein FLJ22639	*FLJ22639*
936	11,2	12,6	-1,13	0,00536	0,313	1552602_at	Calcium channel, voltage-dependent, gamma subunit 5	*CACNG5*
5844	2473	2783	-1,13	0,04722	0,442	211072_x_at	Tubulin, alpha 1b	*TUBA1B*
1870	4,69	5,27	-1,13	0,01208	0,353	1562830_at	Methyltransferase 5 domain containing 1	*METT5D1*
4389	8,37	9,41	-1,13	0,03270	0,407	208223_s_at	Activin A receptor, type IB	*ACVR1B*
1735	8,94	10,1	-1,13	0,01109	0,348	1560059_at	Vacuolar protein sorting 37 homolog C (S. cerevisiae)	*VPS37C*
1110	7,56	8,51	-1,13	0,00650	0,319	223919_at	p53-regulated apoptosis-inducing protein 1	*P53AIP1*
3992	10,2	11,5	-1,12	0,02919	0,400	214479_at	GDNF family receptor alpha 3	*GFRA3*
4828	11,1	12,4	-1,12	0,03676	0,416	208414_s_at	Homeobox B3	*HOXB3*
1669	114	128	-1,12	0,01054	0,345	218381_s_at	U2 small nuclear RNA auxiliary factor 2	*U2AF2*
3506	5,13	5,78	-1,12	0,02518	0,392	220871_at	-	-
1496	7,15	8,05	-1,12	0,00921	0,336	222374_at	Beta-transducin repeat containing	*BTRC*
2979	24,7	27,7	-1,12	0,02095	0,384	220989_s_at	Amnionless homolog (mouse)	*AMN*
743	30,3	34,1	-1,12	0,00422	0,308	224555_x_at	Interleukin 1 family, member 7 (zeta)	*IL1F7*
877	4,56	5,12	-1,12	0,00506	0,313	224006_at	cDNA clone IMAGE:3831123	-
1079	29,1	32,7	-1,12	0,00632	0,319	206437_at	Endothelial differentiation G-protein coupled receptor 6	*EDG6*
4164	7,99	8,98	-1,12	0,03077	0,404	232462_s_at	BC040926	*FLJ23569*
1697	30,2	33,9	-1,12	0,01079	0,348	1570362_at	cDNA clone IMAGE:5749586	-
705	14,8	16,6	-1,12	0,00405	0,308	1561538_at	cDNA clone IMAGE:5271875	-
2994	6,56	7,38	-1,12	0,02109	0,385	210182_at	Cortistatin	*CORT*
3656	4,48	5,04	-1,12	0,02641	0,395	241444_at	Transcribed locus	-
676	8,37	9,41	-1,12	0,00387	0,308	207967_at	Vacuolar protein sorting 45 homolog (S. cerevisiae)	*VPS45*
1338	5,88	6,61	-1,12	0,00804	0,328	1553505_at	Alpha-2-macroglobulin-like 1	*A2ML1*
3667	6,19	6,96	-1,12	0,02651	0,395	1570336_at	3-hydroxybutyrate dehydrogenase, type 1	*BDH1*
3235	7,97	8,96	-1,12	0,02293	0,388	1557569_at	Mannose-P-dolichol utilization defect 1	*MPDU1*
4858	8,36	9,40	-1,12	0,03707	0,417	211484_s_at	Down syndrome cell adhesion molecule	*DSCAM*
3599	4,74	5,32	-1,12	0,02596	0,394	1559926_at	Hypothetical protein LOC728353	*LOC728353*
874	8,26	9,29	-1,12	0,00505	0,313	241361_at	Transcribed locus	-
4648	22,4	25,2	-1,12	0,03505	0,412	214699_x_at	WD repeat domain, phosphoinositide interacting 2	*WIPI2*
2544	9,10	10,2	-1,12	0,01720	0,369	236424_at	Centrosomal protein 250kDa	*CEP250*
2498	14,4	16,2	-1,12	0,01683	0,367	231401_s_at	-	-
2896	12,8	14,3	-1,12	0,02034	0,383	216143_at	cDNA DKFZp434L092	-
2906	4,48	5,04	-1,12	0,02042	0,383	234444_at	cDNA DKFZp434B0735	-
2341	9,93	11,2	-1,12	0,01562	0,364	241540_at	-	-
2275	6,19	6,95	-1,12	0,01513	0,363	1561944_at	cDNA clone IMAGE:5287444	-
356	4,17	4,69	-1,12	0,00183	0,280	207448_at	Protein O-fucosyltransferase 2	*POFUT2*
2831	4,54	5,10	-1,12	0,01976	0,381	213371_at	LIM domain binding 3	*LDB3*
5029	12,1	13,6	-1,12	0,03866	0,420	243045_at	SET and MYND domain containing 1	*SMYD1*
5301	31,4	35,3	-1,12	0,04138	0,426	37424_at	Coiled-coil alpha-helical rod protein 1	*CCHCR1*

Index	Mutated*	Normal*	FC	*P* value	FDR**	Probe set	Description	Gene symbol
6008	13,5	15,2	-1,12	0,04883	0,444	1553162_x_at	Chromosome 19 open reading frame 55	*C19orf55*
1016	5,44	6,11	-1,12	0,00588	0,316	221362_at	5-hydroxytryptamine (serotonin) receptor 5A	*HTR5A*
1055	10,3	11,5	-1,12	0,00618	0,319	1556586_x_at	cDNA clone IMAGE:5310697	-
3459	20,0	22,5	-1,12	0,02478	0,392	1563632_at	Hypothetical protein LOC220980	*LOC220980*
3951	4,41	4,96	-1,12	0,02887	0,399	233277_at	Deleted in lymphocytic leukemia, 7	*DLEU7*
2557	6,24	7,01	-1,12	0,01731	0,370	239187_at	Chromosome 4 open reading frame 30	*C4orf30*
4081	12,4	14,0	-1,12	0,02995	0,401	238266_at	Transcribed locus	-
2825	7,32	8,23	-1,12	0,01972	0,381	1555196_at	cDNA clone IMAGE:4825558	-
5391	11,4	12,8	-1,12	0,04227	0,429	231226_at	Hypothetical protein LOC728142	*LOC728142*
1616	4,70	5,28	-1,12	0,01013	0,343	240805_at	Transcribed locus	-
3732	21,6	24,3	-1,12	0,02709	0,397	221099_at	-	-
2818	4,20	4,72	-1,12	0,01967	0,381	240958_at	Unc-5 homolog C (C. elegans)	*UNC5C*
2886	8,08	9,08	-1,12	0,02024	0,383	228890_at	Atonal homolog 8 (Drosophila)	*ATOH8*
1423	6,13	6,89	-1,12	0,00863	0,331	1559948_at	cDNA FLJ20447 fis, clone KAT05276	-
4660	23,1	25,9	-1,12	0,03518	0,413	231228_at	BCL2-like 1	*BCL2L1*
5686	3,91	4,39	-1,12	0,04539	0,436	206930_at	Glycine-N-acyltransferase	*GLYAT*
5083	114	128	-1,12	0,03913	0,420	202495_at	Tubulin folding cofactor C	*TBCC*
4285	20,1	22,6	-1,12	0,03184	0,406	240508_at	-	-
1749	5,50	6,18	-1,12	0,01117	0,348	222345_at	Transcribed locus	-
1481	5,40	6,06	-1,12	0,00909	0,335	232241_at	-	-
3449	18,4	20,7	-1,12	0,02469	0,391	234214_at	cDNA FLJ39333 fis, clone OCBBF2017306	-
2524	7,84	8,80	-1,12	0,01704	0,369	214546_s_at	Purinergic receptor P2Y, G-protein coupled, 11	*P2RY11*
4763	7,50	8,42	-1,12	0,03610	0,414	207742_s_at	Nuclear receptor subfamily 6, group A, member 1	*NR6A1*
1448	15,6	17,5	-1,12	0,00881	0,333	1569361_a_at	Similar to Serine/arginine repetitive matrix 1	-
485	3,67	4,12	-1,12	0,00259	0,289	244001_at	Nucleosome assembly protein 1-like 4	*NAP1L4*
5633	10,2	11,4	-1,12	0,04497	0,436	242497_at	TRAF-type zinc finger domain containing 1	*TRAFD1*
5074	11,4	12,8	-1,12	0,03904	0,420	242684_at	Zinc finger protein 425	*ZNF425*
5079	13,7	15,3	-1,12	0,03907	0,420	210431_at	Alkaline phosphatase, placental-like 2	*ALPPL2*
6016	5,61	6,30	-1,12	0,04896	0,445	210015_s_at	Microtubule-associated protein 2	*MAP2*
3101	7,89	8,86	-1,12	0,02190	0,386	206610_s_at	Coagulation factor XI (plasma thromboplastin antecedent)	*F11*
314	3,82	4,29	-1,12	0,00159	0,272	231515_at	Transcribed locus	-
687	5,56	6,24	-1,12	0,00393	0,308	239584_at	cDNA FLJ35805 fis, clone TESTI2005982	-
738	3,88	4,36	-1,12	0,00420	0,308	243230_at	-	-
1335	8,30	9,32	-1,12	0,00802	0,328	1560068_a_at	Clone IMAGE:5762932, mRNA	-
3980	7,82	8,78	-1,12	0,02910	0,400	232243_at	Microcephaly, primary autosomal recessive 1	*MCPH1*
2368	9,16	10,3	-1,12	0,01580	0,364	230796_at	Hypothetical LOC440900	*LOC440900*
5712	7,44	8,34	-1,12	0,04574	0,438	234024_at	Cerebellin 4 precursor	*CBLN4*
2637	27,2	30,5	-1,12	0,01798	0,373	224923_at	Tetratricopeptide repeat domain 7A	*TTC7A*
1263	31,6	35,5	-1,12	0,00752	0,325	219333_s_at	Calpain 10	*CAPN10*
1180	4,72	5,30	-1,12	0,00693	0,321	240976_at	Transcribed locus	-
4942	5,48	6,15	-1,12	0,03793	0,419	234439_at	cDNA FLJ10235 fis, clone HEMBB1000339	-
838	4,22	4,73	-1,12	0,00480	0,313	214863_at	Full length insert cDNA clone ZC35F11	-
3935	4,27	4,79	-1,12	0,02875	0,399	238365_s_at	Hypothetical LOC339541	*MGC33556*
3338	6,18	6,94	-1,12	0,02369	0,388	217603_at	ATPase, H+ transporting, lysosomal V0 subunit a2	*ATP6V0A2*
1126	3,72	4,17	-1,12	0,00657	0,319	1564539_at	Hypothetical protein LOC647323	*LOC647323*
2504	5,16	5,79	-1,12	0,01685	0,367	237679_at	Tripartite motif-containing 66	*TRIM66*
698	3,46	3,88	-1,12	0,00401	0,308	243972_at	Transcribed locus	-
3208	8,16	9,15	-1,12	0,02272	0,387	229102_at	HIV-1 Tat interactive protein 2, 30kDa	*HTATIP2*
5561	6,42	7,20	-1,12	0,04429	0,435	220634_at	T-box 4	*TBX4*
2746	5,64	6,32	-1,12	0,01899	0,378	208376_at	Chemokine (C-C motif) receptor 4	*CCR4*
4841	10,2	11,5	-1,12	0,03690	0,417	233983_at	Transglutaminase 6	*TGM6*
2366	12,4	13,9	-1,12	0,01579	0,364	243576_at	-	-
1598	4,10	4,60	-1,12	0,00997	0,341	238308_at	Transcribed locus	-
576	3,77	4,23	-1,12	0,00316	0,300	207592_s_at	Brain cyclic nucleotide gated channel 2	*HCN2*
2130	5,37	6,03	-1,12	0,01401	0,359	237098_at	Chromosome 1 open reading frame 21	*C1orf21*
6090	12,8	14,3	-1,12	0,04972	0,446	1568719_s_at	Chromosome 6 open reading frame 85	*C6orf85*
3642	5,18	5,81	-1,12	0,02633	0,395	1553809_a_at	Chromosome 9 open reading frame 71	*C9orf71*

Index	Mutated*	Normal*	FC	*P* value	FDR**	Probe set	Description	Gene symbol
4992	7,36	8,25	-1,12	0,03831	0,419	233909_at	cDNA DKFZp564C0962	-
5994	4,31	4,84	-1,12	0,04874	0,444	207514_s_at	Guanine nucleotide binding protein (G protein), alpha transducing activity pol	*GNAT1*
3756	9,41	10,6	-1,12	0,02730	0,397	1569419_at	Clone IMAGE:4994945, mRNA	-
1237	6,87	7,70	-1,12	0,00736	0,324	240227_at	Mesoderm induction early response 1 homolog (Xenopus laevis)	*MIER1*
1487	7,26	8,15	-1,12	0,00916	0,336	221368_at	Sialidase 2 (cytosolic sialidase)	*NEU2*
58	3,08	3,45	-1,12	0,00022	0,201	1562699_at	Neural cell adhesion molecule 1	*NCAM1*
2058	5,69	6,38	-1,12	0,01343	0,356	1559867_at	cDNA FLJ32420 fis, clone SKMUS2000898	-
5080	9,01	10,1	-1,12	0,03908	0,420	1559522_at	cDNA FLJ40173 fis, clone TESTI2016922	-
2095	4,19	4,70	-1,12	0,01373	0,358	239126_at	Chromosome 19 open reading frame 23	*C19orf23*
381	4,15	4,65	-1,12	0,00199	0,284	207617_at	DEAD (Asp-Glu-Ala-Asp) box polypeptide 3, X-linked	*DDX3X*
4429	22,0	24,7	-1,12	0,03316	0,409	1566207_at	Transcription elongation factor A (SII), 1	*TCEA1*
4937	5,15	5,78	-1,12	0,03789	0,419	240672_at	-	-
4144	6,39	7,16	-1,12	0,03057	0,403	1561273_at	-	-
5366	12,0	13,5	-1,12	0,04212	0,429	241568_at	Transcribed locus	-
6116	11,3	12,7	-1,12	0,04993	0,446	216760_at	HRAS-like suppressor 2	*HRASLS2*
3113	5,11	5,73	-1,12	0,02199	0,386	1560589_a_at	Clone IMAGE:4657243, mRNA	-
4645	12,2	13,7	-1,12	0,03505	0,412	243435_at	KCNQ1 overlapping transcript 1	*KCNQ1OT1*
1770	7,40	8,29	-1,12	0,01129	0,348	213240_s_at	Keratin 4	*KRT4*
4838	8,41	9,42	-1,12	0,03689	0,417	228984_at	KIAA1394 protein	*KIAA1394*
4246	7,08	7,94	-1,12	0,03151	0,406	233445_at	Transcribed locus	-
5278	17,6	19,8	-1,12	0,04104	0,425	224203_at	Suppressor of fused homolog (Drosophila)	*SUFU*
1170	9,75	10,9	-1,12	0,00688	0,321	208354_s_at	Solute carrier family 12, member 3	*SLC12A3*
2010	7,62	8,54	-1,12	0,01311	0,355	236008_at	Chromosome 9 open reading frame 73	*C9orf73*
2838	9,44	10,6	-1,12	0,01981	0,382	211410_x_at	Killer cell immunoglobulin-like receptor 2DL5A	*KIR2DL5A*
5992	11,3	12,6	-1,12	0,04873	0,444	237507_at	Keratin 73	*KRT73*
2074	9,60	10,8	-1,12	0,01356	0,356	235495_at	Coiled-coil domain containing 97	*CCDC97*
5462	44,6	50,0	-1,12	0,04314	0,432	232581_x_at	Human immunodeficiency virus type I enhancer binding protein 3	*HIVEP3*
1661	3,45	3,86	-1,12	0,01049	0,345	1565838_at	cDNA FLJ35990 fis, clone TESTI2014415	-
2561	6,77	7,58	-1,12	0,01737	0,371	1553802_a_at	SRY (sex determining region Y)-box 3	*SOX3*
1589	7,64	8,56	-1,12	0,00988	0,340	237730_at	cDNA clone IMAGE:5269981	-
1411	7,97	8,93	-1,12	0,00852	0,330	206008_at	Transglutaminase 1	*TGM1*
4080	4,67	5,23	-1,12	0,02994	0,401	214498_at	Agouti signaling protein, nonagouti homolog (mouse)	*ASIP*
3748	6,16	6,90	-1,12	0,02725	0,397	240917_at	-	-
1547	4,33	4,86	-1,12	0,00955	0,337	1566680_at	cDNA FLJ37017 fis, clone BRACE2010642	-
1862	10,4	11,7	-1,12	0,01202	0,353	219748_at	Triggering receptor expressed on myeloid cells-like 2	*TREML2*
3822	12,7	14,2	-1,12	0,02789	0,399	1555650_at	Kelch-like 17 (Drosophila)	*KLHL17*
3689	6,09	6,82	-1,12	0,02665	0,395	224457_at	Hypothetical protein MGC12982	*MGC12982*
3205	6,93	7,76	-1,12	0,02269	0,387	209937_at	Transmembrane 4 L six family member 4	*TM4SF4*
3376	6,37	7,14	-1,12	0,02404	0,389	1568248_x_at	Small nucleolar RNA, H/ACA box 71B	*SNORA71B*
3195	4,62	5,17	-1,12	0,02264	0,387	1556505_at	cDNA clone IMAGE:4826545	-
2500	6,24	6,99	-1,12	0,01684	0,367	215340_at	Adenylate cyclase 1 (brain)	*ADCY1*
2354	38,8	43,4	-1,12	0,01571	0,364	219640_at	Claudin 15	*CLDN15*
5863	10,6	11,9	-1,12	0,04741	0,442	1558174_at	Family with sequence similarity 43, member A	*FAM43A*
5127	5,09	5,70	-1,12	0,03946	0,421	1556879_at	cDNA FLJ39461 fis, clone PROST2011660	-
3377	11,7	13,1	-1,12	0,02404	0,389	230887_at	CDC14 cell division cycle 14 homolog B (S. cerevisiae)	*CDC14B*
1165	8,54	9,56	-1,12	0,00685	0,321	231006_at	Spermatogenesis associated 8	*SPATA8*
729	7,41	8,30	-1,12	0,00417	0,308	221165_s_at	Interleukin 22	*IL22*
1596	15,0	16,8	-1,12	0,00995	0,341	234882_at	-	-
978	5,91	6,62	-1,12	0,00567	0,315	244309_at	Transcribed locus	-
5529	4,13	4,62	-1,12	0,04387	0,434	244879_at	-	-
6084	19,3	21,6	-1,12	0,04967	0,446	217159_x_at	Sialic acid binding Ig-like lectin 7	*SIGLEC7*
3712	5,02	5,62	-1,12	0,02691	0,396	220534_at	Tripartite motif-containing 48	*TRIM48*
2445	7,33	8,21	-1,12	0,01635	0,366	234246_at	-	-
2617	7,37	8,25	-1,12	0,01778	0,371	233395_at	cDNA FLJ12353 fis, clone MAMMA1002322	-
1729	11,4	12,7	-1,12	0,01105	0,348	1569212_at	Scavenger receptor protein family member	*LOC619207*
1849	4,46	4,99	-1,12	0,01190	0,352	207251_at	Meprin A, beta	*MEP1B*
1866	14,5	16,3	-1,12	0,01205	0,353	213337_s_at	Suppressor of cytokine signaling 1	*SOCS1*

Index	Mutated*	Normal*	FC	*P* value	FDR**	Probe set	Description	Gene symbol
1458	39,6	44,4	-1,12	0,00891	0,334	1553361_x_at	F-box and leucine-rich repeat protein 18	*FBXL18*
2596	6,03	6,75	-1,12	0,01765	0,371	240760_at	CMT1A duplicated region transcript 15	*CDRT15*
506	9,60	10,8	-1,12	0,00269	0,289	239608_at	1-acylglycerol-3-phosphate O-acyltransferase 7 (lysophosphatidic acid acyltra	*AGPAT7*
5898	6,02	6,73	-1,12	0,04775	0,443	242554_at	Two pore segment channel 2	*TPCN2*
1838	78,3	87,7	-1,12	0,01183	0,351	202513_s_at	Protein phosphatase 2, regulatory subunit B', delta isoform	*PPP2R5D*
3529	10,8	12,1	-1,12	0,02536	0,393	206064_s_at	Peptidylprolyl isomerase (cyclophilin)-like 2	*PPIL2*
3403	6,41	7,17	-1,12	0,02428	0,390	237757_at	Transcribed locus	-
4736	6,94	7,76	-1,12	0,03585	0,414	227885_at	Hypothetical LOC400236	*LOC400236*
3053	4,03	4,51	-1,12	0,02148	0,385	235809_at	Lin-54 homolog (C. elegans)	*LIN54*
3863	4,26	4,77	-1,12	0,02822	0,399	1559222_at	cDNA FLJ30725 fis, clone FCBBF4000415	-
5233	3,63	4,07	-1,12	0,04049	0,423	244459_at	Transcribed locus	-
3986	10,1	11,4	-1,12	0,02914	0,400	241276_at	-	-
2068	3,69	4,13	-1,12	0,01352	0,356	215746_at	(clone B3B3E13) chromosome 4p16.3 DNA fragment	-
1758	11,8	13,2	-1,12	0,01121	0,348	208539_x_at	Small proline-rich protein 2B	*SPRR2B*
4859	6,70	7,50	-1,12	0,03707	0,417	217471_at	cDNA DKFZp586B1324	-
4925	9,76	10,9	-1,12	0,03777	0,419	230268_at	Transcribed locus	-
3028	5,74	6,42	-1,12	0,02131	0,385	239323_at	cDNA clone IMAGE:5302680	-
5110	14,5	16,2	-1,12	0,03931	0,421	242933_at	Transcribed locus	-
3578	11,4	12,8	-1,12	0,02583	0,394	229972_at	Transcribed locus	-
1574	4,88	5,47	-1,12	0,00978	0,340	1561557_at	Lipase A, lysosomal acid, cholesterol esterase	*LIPA*
3437	6,91	7,73	-1,12	0,02460	0,391	243881_at	cDNA FLJ45325 fis, clone BRHIP3006717	-
5454	37,1	41,5	-1,12	0,04304	0,432	238594_x_at	Dual specificity phosphatase 8	*DUSP8*
1494	5,63	6,29	-1,12	0,00920	0,336	228085_at	cDNA clone IMAGE:4818335	-
1160	6,58	7,36	-1,12	0,00682	0,321	236190_at	Xylosyltransferase I	*XYLT1*
6042	10,3	11,5	-1,12	0,04921	0,445	216911_s_at	Hypermethylated in cancer 2	*HIC2*
4874	7,27	8,13	-1,12	0,03722	0,417	216597_at	Similar to Cationic trypsin III precursor (Pretrypsinogen III)	*LOC642150*
2995	14,0	15,7	-1,12	0,02110	0,385	1563485_at	cDNA DKFZp451D039	-
2598	4,76	5,33	-1,12	0,01765	0,371	231625_at	Solute carrier family 22, member 9	*SLC22A9*
403	3,67	4,11	-1,12	0,00212	0,286	244465_at	Hypothetical protein LOC283033	*LOC283033*
1060	7,32	8,19	-1,12	0,00621	0,319	1558230_at	Splicing factor 3b, subunit 2, 145kDa	*SF3B2*
737	5,02	5,62	-1,12	0,00420	0,308	1555834_at	Ubiquitin carboxyl-terminal esterase L1	*UCHL1*
5832	29,5	33,0	-1,12	0,04713	0,442	223663_at	Coiled-coil domain containing 88B	*CCDC88B*
3329	80,3	89,8	-1,12	0,02364	0,388	212431_at	KIAA0194 protein	*KIAA0194*
5089	5,95	6,66	-1,12	0,03917	0,420	1562045_at	cDNA FLJ34133 fis, clone FCBBF3010601	-
5133	12,3	13,8	-1,12	0,03950	0,421	233696_at	cDNA FLJ21357 fis, clone COL02835	-
3850	13,2	14,7	-1,12	0,02810	0,399	239707_at	Solute carrier family 5, member 10	*SLC5A10*
277	4,26	4,76	-1,12	0,00135	0,263	216622_at	Laminin, beta 4	*LAMB4*
6014	4,75	5,31	-1,12	0,04891	0,445	1562719_at	cDNA clone IMAGE:5297905	-
5404	58,7	65,7	-1,12	0,04241	0,429	212156_at	Vacuolar protein sorting 39 homolog (S. cerevisiae)	*VPS39*
2160	7,57	8,46	-1,12	0,01424	0,360	229929_at	SplA/ryanodine receptor domain and SOCS box containing 4	*SPSB4*
651	8,92	9,97	-1,12	0,00370	0,308	1563405_at	ATPase, H+/K+ exchanging, beta polypeptide	*ATP4B*
3002	3,77	4,21	-1,12	0,02113	0,385	1558601_at	Hypothetical protein LOC285194	*LOC285194*
3281	5,70	6,37	-1,12	0,02337	0,388	205124_at	Myocyte enhancer factor 2B	*MEF2B*
4417	4,92	5,50	-1,12	0,03305	0,409	206017_at	KIAA0319 protein	*KIAA0319*
1664	12,0	13,4	-1,12	0,01051	0,345	233782_at	WD repeat domain 68	*WDR68*
3308	14,2	15,9	-1,12	0,02353	0,388	241054_at	Transcribed locus	-
4734	5,61	6,27	-1,12	0,03583	0,414	1570541_s_at	Similar to Guanylate binding protein 3	*LOC729936*
4752	87,0	97,2	-1,12	0,03600	0,414	1569105_at	SET domain containing 5	*SETD5*
4295	17,7	19,8	-1,12	0,03193	0,406	210200_at	WW domain containing E3 ubiquitin protein ligase 2	*WWP2*
1553	3,55	3,97	-1,12	0,00958	0,337	216901_s_at	IKAROS family zinc finger 1 (Ikaros)	*IKZF1*
1076	7,31	8,17	-1,12	0,00632	0,319	1564319_at	Chromosome 19 open reading frame 16	*C19orf16*
5455	5,13	5,73	-1,12	0,04305	0,432	219139_s_at	KIAA1922 protein	*KIAA1922*
1707	7,02	7,85	-1,12	0,01091	0,348	211364_at	Methylthioadenosine phosphorylase	*MTAP*
6104	6,90	7,71	-1,12	0,04983	0,446	220902_at	Hypothetical LOC196707	*FLJ12616*
2669	21,5	24,1	-1,12	0,01825	0,374	229507_at	Chromosome 3 open reading frame 54	*C3orf54*
4177	6,91	7,72	-1,12	0,03088	0,404	236709_at	Similar to XP_529699.1	-
3703	12,1	13,5	-1,12	0,02678	0,395	220320_at	Docking protein 3	*DOK3*

Index	Mutated*	Normal*	FC	*P* value	FDR**	Probe set	Description	Gene symbol
1133	5,10	5,70	-1,12	0,00662	0,319	217242_at	Zinc finger protein 154	*ZNF154*
2545	12,6	14,1	-1,12	0,01720	0,369	239676_x_at	Hypothetical protein LOC642031	*LOC642031*
3266	14,4	16,1	-1,12	0,02321	0,388	1559204_x_at	v-KI-Ras2 Kirsten rat sarcoma viral oncogene homolog	*KRAS*
4243	11,1	12,4	-1,12	0,03148	0,406	230107_at	Transcribed locus	-
3442	16,3	18,2	-1,12	0,02463	0,391	239256_at	Transcribed locus	-
949	5,74	6,41	-1,12	0,00544	0,313	1559257_a_at	Membrane associated guanylate kinase inverted-1	*MAGI1*
1123	6,11	6,83	-1,12	0,00655	0,319	231621_at	Transcribed locus	-
4129	8,85	9,88	-1,12	0,03040	0,402	208888_s_at	Nuclear receptor co-repressor 2	*NCOR2*
3466	4,74	5,30	-1,12	0,02484	0,392	230749_s_at	Calcium/calmodulin-dependent protein kinase II delta	*CAMK2D*
5649	11,7	13,0	-1,12	0,04507	0,436	223884_at	Opticin	*OPTC*
3359	12,2	13,6	-1,12	0,02384	0,388	239396_at	cDNA clone IMAGE:4150640	-
2247	23,1	25,8	-1,12	0,01493	0,363	226737_at	Solute carrier family 25, member 42	*SLC25A42*
570	6,17	6,89	-1,12	0,00312	0,300	1560631_at	Calcium binding and coiled-coil domain 2	*CALCOCO2*
4572	6,02	6,72	-1,12	0,03457	0,412	206223_at	Lemur tyrosine kinase 2	*LMTK2*
2076	8,84	9,87	-1,12	0,01357	0,356	214064_at	Transferrin	*TF*
1395	5,17	5,77	-1,12	0,00843	0,330	1553673_at	Serine/threonine kinase 35	*STK35*
5520	16,2	18,0	-1,12	0,04379	0,433	221350_at	Homeobox C8	*HOXC8*
3526	22,7	25,3	-1,12	0,02532	0,393	225015_s_at	Serine/threonine kinase 40	*STK40*
4006	10,8	12,0	-1,12	0,02932	0,400	240623_at	Transcribed locus	-
4155	5,89	6,57	-1,12	0,03065	0,403	236865_at	Transcribed locus	-
1041	3,39	3,79	-1,12	0,00605	0,318	1563277_at	Full length insert cDNA YQ02E12	-
504	3,18	3,55	-1,12	0,00268	0,289	1561288_at	cDNA clone IMAGE:4838258	-
989	5,09	5,68	-1,12	0,00570	0,315	236786_at	Family with sequence similarity 135, member B	*FAM135B*
4132	3,51	3,92	-1,12	0,03041	0,402	242779_at	Transcribed locus	-
2466	17,4	19,5	-1,12	0,01657	0,367	216537_s_at	Sialic acid binding Ig-like lectin 7	*SIGLEC7*
1681	6,65	7,42	-1,12	0,01061	0,345	240809_at	Chromosome 21 open reading frame 121	*C21orf121*
5612	8,59	9,58	-1,12	0,04477	0,436	234287_at	mRNA partial cDNA sequence from cDNA selection, DCR1-8.0	-
3636	7,16	7,99	-1,12	0,02629	0,395	241454_at	Transcribed locus	-
512	45,5	50,7	-1,12	0,00273	0,291	33768_at	Dystrophia myotonica, WD repeat containing	*DMWD*
1161	11,5	12,8	-1,12	0,00683	0,321	217365_at	Similar to PRAME family member 6	*LOC729343*
5387	9,21	10,3	-1,12	0,04224	0,429	232764_at	Cyclin B2	*CCNB2*
1271	7,61	8,49	-1,12	0,00755	0,325	207940_x_at	Cannabinoid receptor 1 (brain)	*CNR1*
3701	5,94	6,63	-1,12	0,02677	0,395	215194_at	Protein kinase C, alpha	*PRKCA*
5427	9,47	10,6	-1,12	0,04270	0,430	240372_at	Transcribed locus	-
401	6,43	7,17	-1,12	0,00209	0,285	244863_at	Transcribed locus	-
3679	5,51	6,15	-1,12	0,02658	0,395	224258_at	Endozepine-like protein type 1 mutant	-
1173	5,18	5,78	-1,12	0,00689	0,321	1560856_at	Clone IMAGE:5742065, mRNA	-
4148	16,4	18,3	-1,12	0,03061	0,403	232091_s_at	Zinc finger, DHHC-type containing 24	*ZDHHC24*
5439	18,5	20,6	-1,12	0,04287	0,431	239628_at	Transcribed locus	-
152	3,19	3,56	-1,12	0,00070	0,244	208481_at	Ankyrin repeat and SOCS box-containing 4	*ASB4*
4072	6,43	7,17	-1,12	0,02986	0,401	234187_at	cDNA FLJ22754 fis, clone KAIA0615	-
2403	10,3	11,5	-1,12	0,01603	0,365	234037_s_at	cDNA FLJ22846 fis, clone KAIA555	-
1823	3,48	3,88	-1,12	0,01174	0,351	237050_at	Similar to Potassium channel tetramerisation domain containing 17 isoform 2	-
1586	15,1	16,9	-1,12	0,00987	0,340	222012_at	Hypothetical protein BC002926	*LOC90379*
4644	17,0	18,9	-1,12	0,03503	0,412	229496_at	CLP1, cleavage and polyadenylation factor I subunit, homolog (S. cerevisiae)	*CLP1*
2202	9,62	10,7	-1,12	0,01457	0,361	239257_at	Mov10l1, Moloney leukemia virus 10-like 1, homolog (mouse)	*MOV10L1*
4688	3,19	3,56	-1,12	0,03540	0,413	214554_at	Histone cluster 1, H2ak	*HIST1H2AK*
557	10,2	11,4	-1,12	0,00302	0,296	1553115_at	Naked cuticle homolog 1 (Drosophila)	*NKD1*
4195	14,6	16,3	-1,12	0,03098	0,404	238276_at	Transcribed locus	-
3875	13,4	15,0	-1,12	0,02829	0,399	230453_s_at	ATPase, Ca++ transporting, ubiquitous	*ATP2A3*
1610	6,58	7,34	-1,12	0,01009	0,342	244438_at	Similar to N-myc proto-oncogene protein	-
1189	4,79	5,34	-1,12	0,00703	0,322	240646_at	GTPase, IMAP family member 8	*GIMAP8*
2157	3,24	3,61	-1,12	0,01422	0,360	230673_at	Polycystic kidney and hepatic disease-like 1	*PKHD1L1*
3121	11,6	13,0	-1,12	0,02209	0,387	1557590_at	Hypothetical protein LOC147299	*LOC147299*
5179	12,1	13,5	-1,12	0,03991	0,421	242341_x_at	Glycerate kinase	*GLYCTK*
2639	4,78	5,33	-1,12	0,01799	0,373	243035_at	Transcribed locus	-
4454	7,00	7,81	-1,12	0,03345	0,411	213978_at	Actin-bundling protein with BAIAP2 homology	*ABBA-1*

Index	Mutated*	Normal*	FC	*P* value	FDR**	Probe set	Description	Gene symbol
4518	4,46	4,98	-1,12	0,03406	0,412	240209_at	Chromosome 16 open reading frame 78	*C16orf78*
829	4,42	4,93	-1,12	0,00474	0,312	215345_x_at	T cell receptor gamma variable 7	*TRGV7*
4954	6,36	7,10	-1,12	0,03800	0,419	217633_at	URB1 ribosome biogenesis 1 homolog (S. cerevisiae)	*URB1*
1140	4,48	4,99	-1,12	0,00668	0,320	1561706_at	cDNA DKFZp547N0516	-
2452	6,07	6,77	-1,12	0,01645	0,366	221186_at	cDNA FLJ12781 fis, clone NT2RP2001861	-
1692	5,22	5,82	-1,11	0,01071	0,346	233523_at	Chromosome 20 open reading frame 186	*C20orf186*
4386	5,46	6,09	-1,11	0,03268	0,407	213949_s_at	Deoxyhypusine hydroxylase/monooxygenase	*DOHH*
2101	8,91	9,93	-1,11	0,01378	0,358	207311_at	Double C2-like domains, beta	*DOC2B*
5892	17,8	19,9	-1,11	0,04766	0,442	223994_s_at	Solute carrier family 12, member 9	*SLC12A9*
4186	5,71	6,37	-1,11	0,03095	0,404	241398_at	Metallophosphoesterase domain containing 1	*MPPED1*
2308	4,52	5,04	-1,11	0,01532	0,363	232352_at	ISL LIM homeobox 2	*ISL2*
1416	5,10	5,69	-1,11	0,00858	0,331	221289_at	Distal-less homeobox 6	*DLX6*
3967	12,3	13,7	-1,11	0,02898	0,399	1554175_at	CD300 molecule-like family member b	*CD300LB*
4058	23,8	26,5	-1,11	0,02977	0,401	1563808_at	MCF.2 cell line derived transforming sequence-like	*MCF2L*
2227	39,4	43,9	-1,11	0,01480	0,363	218551_at	Invasion inhibitory protein 45	*MIIP*
863	4,90	5,46	-1,11	0,00497	0,313	215740_at	cDNA FLJ12573 fis, clone NT2RM4000979	-
5270	4,06	4,52	-1,11	0,04093	0,425	205289_at	Bone morphogenetic protein 2	*BMP2*
4331	5,66	6,31	-1,11	0,03224	0,407	210508_s_at	Potassium voltage-gated channel, KQT-like subfamily, member 2	*KCNQ2*
5823	6,97	7,77	-1,11	0,04705	0,442	1553544_at	G protein-coupled receptor 101	*GPR101*
3785	4,22	4,71	-1,11	0,02757	0,398	217177_s_at	cDNA FLJ13658 fis, clone PLACE1011567	-
4467	6,78	7,56	-1,11	0,03355	0,411	240891_at	Follistatin-like 1	*FSTL1*
5773	11,3	12,6	-1,11	0,04639	0,439	241747_s_at	Cullin 7	*CUL7*
5229	6,31	7,04	-1,11	0,04046	0,423	1570282_at	-	-
1040	4,81	5,36	-1,11	0,00605	0,318	1559568_at	cDNA DKFZp313C0731	-
3177	8,53	9,50	-1,11	0,02254	0,387	244520_at	Transcribed locus	-
1611	4,95	5,52	-1,11	0,01009	0,342	219839_x_at	T-cell leukemia/lymphoma 6	*TCL6*
3829	6,10	6,80	-1,11	0,02795	0,399	239094_at	Hypothetical protein LOC730961	*LOC730961*
3222	15,4	17,1	-1,11	0,02283	0,387	1552995_at	Interleukin 27	*IL27*
5355	7,25	8,08	-1,11	0,04201	0,429	205874_at	Inositol 1,4,5-trisphosphate 3-kinase A	*ITPKA*
4801	23,7	26,5	-1,11	0,03643	0,415	231305_at	Transcribed locus	-
2567	7,11	7,93	-1,11	0,01740	0,371	1555542_at	Actin filament associated protein 1-like 1	*AFAP1L1*
2514	3,78	4,21	-1,11	0,01693	0,368	1563092_at	mRNA full length insert cDNA clone EUROIMAGE 712308	-
5660	14,2	15,9	-1,11	0,04515	0,436	217095_x_at	Natural cytotoxicity triggering receptor 1	*NCR1*
589	4,74	5,29	-1,11	0,00325	0,301	216729_at	cDNA FLJ21735 fis, clone COLF3350	-
4199	22,8	25,4	-1,11	0,03104	0,404	1554516_at	Hypothetical protein LOC203274	*LOC203274*
1992	3,99	4,44	-1,11	0,01303	0,355	240732_at	Transcribed locus	-
4370	11,6	12,9	-1,11	0,03252	0,407	201269_s_at	NudC domain containing 3	*NUDCD3*
6009	6,13	6,83	-1,11	0,04883	0,444	204499_at	ATP/GTP binding protein 1	*AGTPBP1*
4420	45,7	50,8	-1,11	0,03309	0,409	221890_at	Zinc finger protein 335	*ZNF335*
4082	4,85	5,40	-1,11	0,02998	0,402	222055_at	Fumarylacetoacetate hydrolase domain containing 2A	*FAHD2A*
3373	22,6	25,2	-1,11	0,02400	0,389	231355_at	cDNA FLJ30598 fis, clone BRAWH2009263	-
939	3,98	4,44	-1,11	0,00538	0,313	222189_at	Hypothetical LOC646649	*LOC646649*
3432	7,88	8,77	-1,11	0,02457	0,391	231428_at	Transcribed locus	-
5519	24,9	27,8	-1,11	0,04379	0,433	216551_x_at	Phospholipase C, gamma 1	*PLCG1*
3928	5,86	6,52	-1,11	0,02873	0,399	238405_at	Transcribed locus	-
2954	12,7	14,1	-1,11	0,02074	0,384	244182_at	Clone IMAGE:5756056, mRNA	-
3481	16,8	18,7	-1,11	0,02499	0,392	207892_at	CD40 ligand (TNF superfamily, member 5, hyper-IgM syndrome)	*CD40LG*
2779	8,18	9,11	-1,11	0,01930	0,380	1560618_at	Clone IMAGE:5186867, mRNA	-
3886	21,8	24,3	-1,11	0,02838	0,399	223733_s_at	Protein phosphatase 4, regulatory subunit 1-like	*PPP4R1L*
612	4,30	4,79	-1,11	0,00346	0,308	231373_at	Ring finger protein 133	*RNF133*
3769	8,16	9,09	-1,11	0,02738	0,397	223508_at	Notch homolog 1, translocation-associated (Drosophila)	*NOTCH1*
4338	17,1	19,0	-1,11	0,03229	0,407	1561403_at	Spermatogenesis and oogenesis specific basic helix-loop-helix 1	*SOHLH1*
748	2,98	3,31	-1,11	0,00424	0,308	237551_at	URB1 ribosome biogenesis 1 homolog (S. cerevisiae)	*URB1*
5171	9,30	10,4	-1,11	0,03983	0,421	203673_at	Thyroglobulin	*TG*
5759	6,11	6,80	-1,11	0,04626	0,439	211380_s_at	Protein kinase, cGMP-dependent, type I	*PRKG1*
369	3,21	3,57	-1,11	0,00194	0,284	233886_at	cDNA DKFZp564F172	-
5905	26,5	29,5	-1,11	0,04784	0,443	209415_at	fizzy/cell division cycle 20 related 1 (Drosophila)	*FZR1*

Index	Mutated*	Normal*	FC	*P* value	FDR**	Probe set	Description	Gene symbol
5236	13,4	14,9	-1,11	0,04051	0,423	217490_at	cDNA DKFZp564P073	-
3556	4,75	5,29	-1,11	0,02565	0,394	223756_at	Hypothetical protein FLJ10081	*FLJ10081*
2961	8,61	9,59	-1,11	0,02081	0,384	204801_s_at	Dehydrogenase/reductase (SDR family) member 12	*DHRS12*
1225	11,2	12,4	-1,11	0,00729	0,324	223536_at	Pleckstrin and Sec7 domain containing 2	*PSD2*
907	4,37	4,87	-1,11	0,00524	0,313	1562116_at	Clone IMAGE:5191738, mRNA	-
1350	5,04	5,61	-1,11	0,00811	0,328	231150_at	Transcribed locus	-
5558	19,1	21,3	-1,11	0,04425	0,435	232907_at	Ubiquitin protein ligase E3 component n-recognin 4	*UBR4*
633	14,6	16,2	-1,11	0,00361	0,308	1566268_at	Full length insert cDNA YR25G06	-
2731	30,8	34,3	-1,11	0,01881	0,377	89948_at	Chromosome 20 open reading frame 67	*C20orf67*
4411	5,56	6,18	-1,11	0,03294	0,408	1569264_at	Hypothetical gene supported by BC013370	*LOC400655*
4181	9,43	10,5	-1,11	0,03091	0,404	235541_at	LAS1-like (S. cerevisiae)	*LAS1L*
2207	41,0	45,6	-1,11	0,01459	0,361	632_at	Glycogen synthase kinase 3 alpha	*GSK3A*
3133	5,95	6,62	-1,11	0,02222	0,387	217702_at	Interleukin 27 receptor, alpha	*IL27RA*
5776	10,7	11,9	-1,11	0,04647	0,440	1559478_at	cDNA clone IMAGE:5270289	-
5336	3,38	3,76	-1,11	0,04178	0,428	231449_at	Transcribed locus	-
5396	7,22	8,03	-1,11	0,04231	0,429	207792_at	Opioid receptor, delta 1	*OPRD1*
923	3,30	3,67	-1,11	0,00532	0,313	1562216_at	-	-
5805	19,4	21,6	-1,11	0,04685	0,441	223726_at	Potassium voltage-gated channel, subfamily H member 3	*KCNH3*
3680	11,4	12,7	-1,11	0,02658	0,395	1556669_a_at	Full length insert cDNA clone YR71G12	-
1886	5,12	5,70	-1,11	0,01224	0,355	231066_s_at	Chloride channel 4	*CLCN4*
307	3,13	3,48	-1,11	0,00148	0,263	237240_at	Transcribed locus	-
2169	24,2	26,9	-1,11	0,01435	0,361	1562981_at	Hemoglobin, beta	*HBB*
639	5,85	6,51	-1,11	0,00363	0,308	242867_x_at	Transcribed locus	-
1125	4,96	5,52	-1,11	0,00657	0,319	243441_at	Transcribed locus	-
3246	5,28	5,87	-1,11	0,02303	0,388	206417_at	Cyclic nucleotide gated channel alpha 1	*CNGA1*
1362	4,03	4,48	-1,11	0,00820	0,328	231742_at	Cone-rod homeobox	*CRX*
3908	38,9	43,3	-1,11	0,02853	0,399	204692_at	Leucine rich repeat neuronal 4	*LRCH4*
2538	3,30	3,67	-1,11	0,01717	0,369	1561678_at	cDNA clone IMAGE:5266579	-
3616	12,1	13,4	-1,11	0,02611	0,395	212389_at	SET binding factor 1	*SBF1*
4773	26,1	29,0	-1,11	0,03620	0,414	231553_s_at	Flavoprotein oxidoreductase MICAL3	*MICAL3*
2036	4,15	4,61	-1,11	0,01326	0,355	228872_at	Family with sequence similarity 108, member B1	*FAM108B1*
974	3,56	3,96	-1,11	0,00566	0,315	1564209_at	Hypothetical protein LOC282980	*LOC282980*
2805	8,54	9,50	-1,11	0,01952	0,380	207644_at	Forkhead box H1	*FOXH1*
3095	4,32	4,81	-1,11	0,02182	0,385	228659_at	-	-
3847	24,9	27,6	-1,11	0,02808	0,399	1557656_at	Hypothetical gene supported by AK093266	*LOC400238*
3369	11,1	12,3	-1,11	0,02396	0,389	234397_at	-	-
1518	5,12	5,69	-1,11	0,00938	0,337	210107_at	Chloride channel, calcium activated, family member 1	*CLCA1*
5550	14,0	15,5	-1,11	0,04413	0,435	1557630_s_at	cDNA clone IMAGE:5273415	-
2709	6,79	7,54	-1,11	0,01860	0,375	1559578_at	cDNA FLJ31857 fis, clone NT2RP7001156	-
3978	8,16	9,07	-1,11	0,02908	0,400	206986_at	Hypothetical protein LOC728985	*LOC728985*
6122	4,86	5,40	-1,11	0,04998	0,446	229332_at	4-hydroxyphenylpyruvate dioxygenase-like	*HPDL*
225	3,36	3,73	-1,11	0,00104	0,250	1570263_at	-	-
1354	5,01	5,57	-1,11	0,00813	0,328	1557693_at	cDNA clone IMAGE:5303580	-
4624	9,48	10,5	-1,11	0,03488	0,412	234575_at	Zinc finger protein 71	*ZNF71*
5128	3,67	4,08	-1,11	0,03946	0,421	223796_at	Contactin associated protein-like 3	*CNTNAP3*
2663	4,96	5,51	-1,11	0,01819	0,374	207359_at	Calcium/calmodulin-dependent protein kinase kinase 2, beta	*CAMKK2*
3215	6,51	7,24	-1,11	0,02277	0,387	238373_at	H1 histone family, member N, testis-specific	*H1FNT*
3392	5,11	5,68	-1,11	0,02416	0,389	1560153_at	Fraser syndrome 1	*FRAS1*
3535	32,1	35,7	-1,11	0,02543	0,393	1563315_s_at	Glutamate-rich 1	*ERICH1*
5537	4,15	4,61	-1,11	0,04396	0,434	224195_at	Testis-specific transcript, Y-linked 12	*TTTY12*
4545	8,33	9,25	-1,11	0,03436	0,412	228079_at	Chromosome 3 open reading frame 58	*C3orf58*
3206	8,56	9,51	-1,11	0,02270	0,387	243343_at	Transcribed locus	-
2582	14,6	16,2	-1,11	0,01752	0,371	221328_at	Claudin 17	*CLDN17*
5971	9,32	10,4	-1,11	0,04849	0,444	216019_x_at	Pleckstrin homology-like domain, family B, member 1	*PHLDB1*
1732	5,65	6,28	-1,11	0,01105	0,348	216226_at	Transcription initiation factor TFIID 105 kDa subunit	*TAF4B*
4257	7,46	8,28	-1,11	0,03163	0,406	233733_at	cDNA DKFZp434E1920	-
2625	5167	5741	-1,11	0,01784	0,372	1553551_s_at	-	-

Index	Mutated*	Normal*	FC	*P* value	FDR**	Probe set	Description	Gene symbol
5064	7,61	8,45	-1,11	0,03897	0,420	243987_at	Similar to XP_001136680.1	-
4394	5,75	6,39	-1,11	0,03275	0,407	1554115_at	Hypothetical gene LOC133874	*LOC133874*
2392	4,27	4,74	-1,11	0,01596	0,365	237275_at	Similar to Otoconin 90	-
82	3,71	4,12	-1,11	0,00031	0,201	241055_at	Endothelial PAS domain protein 1	*EPAS1*
1931	15,7	17,4	-1,11	0,01258	0,355	1555742_at	-	-
3085	5,96	6,62	-1,11	0,02174	0,385	234456_at	Trafficking protein particle complex 4	*TRAPPC4*
5574	4,61	5,13	-1,11	0,04439	0,435	1560399_a_at	OTU domain containing 7A	*OTUD7A*
4479	6,95	7,73	-1,11	0,03369	0,411	240169_at	Full length insert cDNA clone YO64F11	-
586	7,85	8,72	-1,11	0,00324	0,301	235909_at	Hypothetical gene supported by BC040598	*LOC400960*
5815	4,59	5,10	-1,11	0,04698	0,442	221323_at	UL16 binding protein 1	*ULBP1*
1525	3,71	4,13	-1,11	0,00942	0,337	1558534_at	Hypothetical gene LOC283846	-
2384	6,11	6,79	-1,11	0,01589	0,364	1557908_at	cDNA FLJ37090 fis, clone BRACE2017587	-
5297	6,29	6,99	-1,11	0,04127	0,426	228807_at	-	-
3063	11,4	12,6	-1,11	0,02159	0,385	215420_at	Indian hedgehog homolog (Drosophila)	*IHH*
3446	18,0	20,0	-1,11	0,02466	0,391	1558211_s_at	v-src sarcoma (Schmidt-Ruppin A-2) viral oncogene homolog (avian)	*SRC*
1916	7,71	8,56	-1,11	0,01250	0,355	241892_at	Transcribed locus	-
1440	5,79	6,43	-1,11	0,00876	0,332	222285_at	immunoglobulin heavy constant delta	*IGHD*
3293	8,92	9,91	-1,11	0,02345	0,388	235087_at	Unkempt homolog (Drosophila)-like	*UNKL*
3893	11,2	12,4	-1,11	0,02844	0,399	1555021_a_at	Scavenger receptor class F, member 1	*SCARF1*
1265	20,2	22,4	-1,11	0,00753	0,325	211350_s_at	Chromosome 6 open reading frame 54	*C6orf54*
2898	24,7	27,4	-1,11	0,02035	0,383	223525_at	Delta-like 4 (Drosophila)	*DLL4*
832	3,55	3,95	-1,11	0,00475	0,312	1566279_at	-	-
695	2,91	3,23	-1,11	0,00400	0,308	1562443_at	Retinaldehyde binding protein 1-like 2	*RLBP1L2*
6082	7,91	8,78	-1,11	0,04966	0,446	231454_at	Placenta-specific 4	*PLAC4*
2730	12,2	13,6	-1,11	0,01881	0,377	214898_x_at	Mucin 3B, cell surface associated	*MUC3B*
5849	8,97	9,96	-1,11	0,04730	0,442	1555063_at	Ubiquitin specific peptidase 6	*USP6*
4870	8,19	9,09	-1,11	0,03718	0,417	222695_s_at	Axin 2 (conductin, axil)	*AXIN2*
2675	4,97	5,52	-1,11	0,01830	0,374	237558_at	Transcribed locus	-
2323	4,16	4,61	-1,11	0,01546	0,364	1562965_at	Clone IMAGE:5744268, mRNA	-
5115	16,8	18,7	-1,11	0,03938	0,421	233486_at	Hypothetical protein LOC729940	*LOC729940*
5383	6,03	6,70	-1,11	0,04221	0,429	243376_at	TRAF family member-associated NFKB activator	*TANK*
4561	4,30	4,77	-1,11	0,03446	0,412	206078_at	Kalirin, RhoGEF kinase	*KALRN*
4443	30,3	33,7	-1,11	0,03338	0,411	210737_at	Tubby homolog (mouse)	*TUB*
1687	4,27	4,74	-1,11	0,01066	0,345	207913_at	Similar to Cytochrome P450, family 2, subfamily F, polypeptide 1	*LOC731992*
4188	5,38	5,97	-1,11	0,03096	0,404	240283_at	-	-
5443	14,5	16,1	-1,11	0,04293	0,431	214968_at	DEAD (Asp-Glu-Ala-Asp) box polypeptide 51	*DDX51*
5456	7,50	8,33	-1,11	0,04308	0,432	206839_at	Chromosome 22 open reading frame 31	*C22orf31*
4014	6,71	7,45	-1,11	0,02939	0,400	244577_at	Transcribed locus	-
2837	4,83	5,36	-1,11	0,01980	0,382	224086_at	-	-
4251	18,6	20,6	-1,11	0,03157	0,406	211624_s_at	Dopamine receptor D2	*DRD2*
4663	21,8	24,2	-1,11	0,03520	0,413	216481_at	Glutamate receptor interacting protein 2	*GRIP2*
869	3,70	4,11	-1,11	0,00502	0,313	231501_at	Similar to XP_001141902.1	-
763	5,84	6,49	-1,11	0,00430	0,308	1567540_at	Sperm associated antigen 10	*SPAG10*
4854	24,8	27,5	-1,11	0,03701	0,417	210226_at	Nuclear receptor subfamily 4, group A, member 1	*NR4A1*
3702	6,14	6,82	-1,11	0,02677	0,395	234671_at	Keratin associated protein 4-2	*KRTAP4-2*
438	4,18	4,63	-1,11	0,00232	0,288	240640_at	-	-
3197	7,32	8,13	-1,11	0,02265	0,387	244127_at	Transcribed locus	-
5985	3,81	4,22	-1,11	0,04861	0,444	210492_at	Microfibrillar-associated protein 3-like	*MFAP3L*
2752	10,0	11,1	-1,11	0,01905	0,378	244421_at	Transcribed locus	-
4131	6,27	6,96	-1,11	0,03041	0,402	237207_at	Transcribed locus	-
4268	7,32	8,12	-1,11	0,03174	0,406	1557279_at	intersectin 1 (SH3 domain protein)	*ITSN1*
5590	13,8	15,3	-1,11	0,04458	0,436	210457_x_at	High mobility group AT-hook 1	*HMGA1*
294	3,80	4,22	-1,11	0,00144	0,263	210921_at	-	-
4814	15,1	16,8	-1,11	0,03659	0,416	216183_at	Transglutaminase 2	*TGM2*
3519	4,71	5,23	-1,11	0,02526	0,392	1564443_at	Deleted in lymphocytic leukemia, 2	*DLEU2*
429	6,27	6,96	-1,11	0,00226	0,288	207385_at	Transcription factor Dp family, member 3	*TFDP3*
4035	4,71	5,22	-1,11	0,02956	0,401	214599_at	Involucrin	*IVL*

Index	Mutated*	Normal*	FC	*P* value	FDR**	Probe set	Description	Gene symbol
2173	8,64	9,58	-1,11	0,01436	0,361	240497_at	-	-
5327	8,36	9,28	-1,11	0,04164	0,427	240001_at	-	-
2206	7,64	8,48	-1,11	0,01459	0,361	217077_s_at	Gamma-aminobutyric acid (GABA) B receptor, 2	*GABBR2*
4134	20,9	23,2	-1,11	0,03043	0,402	236441_at	Transcribed locus	-
840	5,31	5,89	-1,11	0,00481	0,313	1554372_at	-	-
3579	3,94	4,37	-1,11	0,02584	0,394	236638_at	Transcribed locus	-
1377	3,76	4,17	-1,11	0,00830	0,329	234603_at	cDNA DKFZp566E213	-
5284	4,36	4,84	-1,11	0,04110	0,425	230480_at	Piwi-like 4 (Drosophila)	*PIWIL4*
2029	4,05	4,50	-1,11	0,01323	0,355	1569283_at	HCG1646157 protein	-
660	12,8	14,2	-1,11	0,00382	0,308	244307_s_at	Synaptotagmin XV	*SYT15*
5260	6,34	7,03	-1,11	0,04085	0,425	240565_at	Zinc finger protein 28 homolog (mouse)	*ZFP28*
3315	3,51	3,89	-1,11	0,02356	0,388	1556476_at	Hypothetical protein LOC283485	*LOC283485*
1206	3,46	3,83	-1,11	0,00715	0,324	231160_at	Transcribed locus	-
2255	6,10	6,77	-1,11	0,01501	0,363	234239_at	cDNA FLJ21668 fis, clone COL08982	-
4204	5,94	6,59	-1,11	0,03109	0,404	1554583_a_at	Hypothetical protein MGC50559	*MGC50559*
4977	5,09	5,64	-1,11	0,03819	0,419	210602_s_at	Cadhetrin 6, type 2, K-Cadhetrin (fetal kidney)	*CDH6*
3693	18,4	20,4	-1,11	0,02668	0,395	208347_at	-	-
1645	3,64	4,04	-1,11	0,01036	0,344	1562712_at	Clone IMAGE:5165425, mRNA	-
4785	13,3	14,8	-1,11	0,03628	0,414	203937_s_at	Transcription initiation factor SL1/TIF-IB subunit C	*TAF1C*
1892	5,82	6,46	-1,11	0,01228	0,355	1553031_at	G protein-coupled receptor 115	*GPR115*
561	3,85	4,27	-1,11	0,00306	0,298	233283_at	cDNA FLJ14025 fis, clone HEMBA1003667	-
910	4,96	5,50	-1,11	0,00525	0,313	208563_x_at	POU class 3 homeobox 3	*POU3F3*
3440	9,16	10,2	-1,11	0,02463	0,391	1556107_at	cDNA DKFZp667C1617	-
5008	64,9	72,0	-1,11	0,03847	0,420	206635_at	Cholinergic receptor, nicotinic, beta 2 (neuronal)	*CHRNB2*
2556	4,81	5,33	-1,11	0,01729	0,370	1563171_at	cDNA clone IMAGE:5165280	-
2277	6,83	7,58	-1,11	0,01514	0,363	215594_at	PTR2 mRNA for repetitive sequence	-
4551	8,32	9,23	-1,11	0,03441	0,412	232913_at	Transmembrane emp24 protein transport domain containing 8	*TMED8*
2671	9,45	10,5	-1,11	0,01826	0,374	231224_x_at	Protein kinase, AMP-activated, gamma 2 non-catalytic subunit	*PRKAG2*
5505	126	140	-1,11	0,04359	0,433	1569522_at	Clone IMAGE:4424208, mRNA	-
1159	9,02	10,0	-1,11	0,00681	0,321	221112_at	Interleukin 1 receptor accessory protein-like 2	*IL1RAPL2*
6070	20,6	22,9	-1,11	0,04955	0,446	1566954_at	cDNA FLJ20891 fis, clone ADKA03345	-
4445	8,76	9,72	-1,11	0,03340	0,411	211147_s_at	Purinergic receptor P2X-like 1, orphan receptor	*P2RXL1*
943	6,47	7,18	-1,11	0,00541	0,313	1558770_a_at	Chromosome 17 open reading frame 38	*C17orf38*
4556	8,79	9,75	-1,11	0,03444	0,412	1559977_a_at	Solute carrier family 25, member 34	*SLC25A34*
5871	29,8	33,0	-1,11	0,04744	0,442	204056_s_at	Mevalonate kinase (mevalonic aciduria)	*MVK*
3321	8,18	9,07	-1,11	0,02359	0,388	1563842_at	Phosphatidylinositol glycan anchor biosynthesis, class G	*PIGG*
1600	5,35	5,93	-1,11	0,00998	0,341	220908_at	Coiled-coil domain containing 33	*CCDC33*
983	3,29	3,64	-1,11	0,00569	0,315	244240_at	-	-
935	6,09	6,75	-1,11	0,00536	0,313	236320_at	Coiled-coil domain containing 17	*CCDC17*
3993	6,68	7,40	-1,11	0,02920	0,400	1562641_at	Family with sequence similarity 122C	*FAM122C*
2695	98,5	109	-1,11	0,01848	0,375	1556507_at	cDNA clone IMAGE:5267328	-
3891	11,5	12,8	-1,11	0,02844	0,399	240744_at	Carboxypeptidase A5	*CPA5*
2505	30,4	33,7	-1,11	0,01685	0,367	221180_at	Yeast Sps1/Ste20-related kinase 4 (S. cerevisiae)	*YSK4*
3920	16,5	18,3	-1,11	0,02863	0,399	1559402_a_at	Chromosome 1 open reading frame 61	*C1orf61*
1983	19,1	21,2	-1,11	0,01295	0,355	227090_at	PHD finger protein 21A	*PHF21A*
3569	10,5	11,6	-1,11	0,02577	0,394	207187_at	Janus kinase 3 (a protein tyrosine kinase, leukocyte)	*JAK3*
2136	61,2	67,8	-1,11	0,01404	0,359	31861_at	Immunoglobulin mu binding protein 2	*IGHMBP2*
5183	5,00	5,54	-1,11	0,03995	0,421	1554940_a_at	Hypothetical LOC388882	*LOC388882*
3320	5,80	6,43	-1,11	0,02359	0,388	1552917_at	Interleukin 29 (Interferon, lambda 1)	*IL29*
5653	14,4	16,0	-1,11	0,04510	0,436	1562434_at	Unkempt homolog (Drosophila)	*UNK*
3839	8,31	9,21	-1,11	0,02804	0,399	1562674_at	Clone IMAGE:5165176, mRNA	-
3407	3,96	4,39	-1,11	0,02430	0,390	215409_at	1-acylglycerol-3-phosphate O-acyltransferase 7 (lysophosphatidic acid acyltra	*AGPAT7*
4009	6,30	6,98	-1,11	0,02934	0,400	242535_at	Transcribed locus	-
2256	8,02	8,89	-1,11	0,01502	0,363	206913_at	Bile acid Coenzyme A: amino acid N-acyltransferase	*BAAT*
2722	5,84	6,47	-1,11	0,01871	0,376	238321_at	TEA domain family member 2	*TEAD2*
1608	4,87	5,40	-1,11	0,01007	0,342	1556999_at	Hypothetical protein LOC285045	*LOC285045*
4041	6,63	7,35	-1,11	0,02962	0,401	234534_at	cDNA FLJ20100 fis, clone COL04648	-

Index	Mutated*	Normal*	FC	*P* value	FDR**	Probe set	Description	Gene symbol
5796	7,09	7,85	-1,11	0,04675	0,441	240157_at	Transcribed locus	-
421	3,11	3,45	-1,11	0,00221	0,286	234581_at	cDNA FLJ21257 fis, clone COL01407	-
5091	10,4	11,5	-1,11	0,03917	0,420	1552646_at	Interleukin 11 receptor, alpha	*IL11RA*
2885	8,54	9,46	-1,11	0,02024	0,383	1567298_at	Olfactory receptor, family 13, subfamily C, member 4	*OR13C4*
1978	3,97	4,39	-1,11	0,01292	0,355	233887_at	G protein-coupled receptor 126	*GPR126*
494	6,31	6,99	-1,11	0,00264	0,289	230138_at	Frequenin homolog (Drosophila)	*FREQ*
3608	5,04	5,58	-1,11	0,02603	0,394	223881_at	Islet cell autoantigen 1,69kDa-like	*ICA1L*
1827	4,01	4,44	-1,11	0,01177	0,351	1562384_at	Chromosome 21 mRNA sequence	-
2867	9,09	10,1	-1,11	0,02011	0,383	219672_at	Erythroid associated factor	*ERAF*
5234	30,1	33,4	-1,11	0,04050	0,423	216791_at	Transmembrane protein 92	*TMEM92*
3892	6,11	6,77	-1,11	0,02844	0,399	217459_at	cDNA DKFZp434L1016	-
4203	25,5	28,3	-1,11	0,03108	0,404	214444_s_at	Poliovirus receptor	*PVR*
2289	6,62	7,33	-1,11	0,01521	0,363	206844_at	Fructose-1,6-bisphosphatase 2	*FBP2*
3414	12,0	13,2	-1,11	0,02437	0,390	215875_at	cDNA FLJ23167 fis, clone LNG09902	-
5000	18,6	20,6	-1,11	0,03839	0,420	217033_x_at	Neurotrophic tyrosine kinase, receptor, type 3	*NTRK3*
3997	15,7	17,4	-1,11	0,02927	0,400	237736_at	Transcribed locus	-
1061	31,3	34,7	-1,11	0,00623	0,319	202523_s_at	SPARC/osteonectin, CWCV, and Kazal-like domains proteoglycan 2	*SPOCK2*
4310	4,82	5,34	-1,11	0,03209	0,407	232920_at	Hypothetical LOC550631	*LOC550631*
3294	6,09	6,75	-1,11	0,02345	0,388	1555051_at	Chromosome 10 open reading frame 53	*C10orf53*
1689	5,32	5,89	-1,11	0,01067	0,345	241046_at	Transcribed locus	-
1775	11,5	12,7	-1,11	0,01130	0,348	206455_s_at	Rhodopsin (opsin 2, rod pigment)	*RHO*
4782	14,5	16,0	-1,11	0,03627	0,414	1555343_at	Multiple EGF-like-domains 10	*MEGF10*
2697	5,00	5,54	-1,11	0,01850	0,375	233752_s_at	Zinc finger homeobox 3	*ZFHX3*
736	18,4	20,4	-1,11	0,00420	0,308	1561171_a_at	FLJ40198 protein	*FLJ36131*
3457	5,19	5,74	-1,11	0,02477	0,392	241013_at	Family with sequence similarity 124A	*FAM124A*
3144	6,61	7,32	-1,11	0,02231	0,387	215476_at	Clone 23726 mRNA sequence	-
2200	4,32	4,79	-1,11	0,01454	0,361	214576_at	Keratin 36	*KRT36*
4984	13,5	15,0	-1,11	0,03825	0,419	230253_at	Signal peptide, CUB domain, EGF-like 3	*SCUBE3*
1155	7,25	8,03	-1,11	0,00678	0,321	239327_at	Transcribed locus	-
4953	9,92	11,0	-1,11	0,03799	0,419	1566484_at	cDNA DKFZp666K2010	-
4349	5,40	5,98	-1,11	0,03239	0,407	216779_at	Cylicin, basic protein of sperm head cytoskeleton 1	*CYLC1*
1326	4,34	4,80	-1,11	0,00798	0,328	1556645_s_at	Clone IMAGE:6016214, mRNA	-
1113	6,59	7,29	-1,11	0,00651	0,319	234206_at	cDNA FLJ21271 fis, clone COL01751	-
1619	6,15	6,81	-1,11	0,01017	0,343	1556914_at	cDNA clone IMAGE:4838482	-
5806	5,43	6,01	-1,11	0,04686	0,441	206454_s_at	Rhodopsin (opsin 2, rod pigment)	*RHO*
3982	3,47	3,84	-1,11	0,02913	0,400	1566140_at	Homeodomain-only protein	*HOP*
1722	4,40	4,87	-1,11	0,01103	0,348	240722_at	Transcribed locus	-
5180	36,1	40,0	-1,11	0,03993	0,421	36888_at	KIAA0841 protein	*KIAA0841*
2261	5,27	5,84	-1,11	0,01505	0,363	230975_at	Transcribed locus	-
3894	7,74	8,56	-1,11	0,02845	0,399	207969_x_at	Acrosomal vesicle protein 1	*ACRV1*
3915	3,52	3,89	-1,11	0,02860	0,399	232562_at	cDNA FLJ11554 fis, clone HEMBA1003037	-
564	5,21	5,77	-1,11	0,00309	0,299	234027_at	Coiled-coil domain containing 129	*CCDC129*
2434	6,26	6,93	-1,11	0,01627	0,365	1559354_a_at	Full length insert cDNA YQ09C04	-
828	3,63	4,02	-1,11	0,00472	0,312	1560298_at	cDNA clone IMAGE:4814133	-
4659	17,3	19,1	-1,11	0,03518	0,413	206998_x_at	Proline-rich protein BstNI subfamily 1	*PRB1*
3934	6,25	6,91	-1,11	0,02874	0,399	1569219_at	Clone IMAGE:4655360, mRNA	-
2464	9,58	10,6	-1,11	0,01654	0,367	214256_at	ATPase, Class V, type 10A	*ATP10A*
4059	9,55	10,6	-1,11	0,02978	0,401	1562239_at	Small G protein signaling modulator 1	*SGSM1*
5001	16,7	18,4	-1,11	0,03840	0,420	208277_at	Paired-like homeodomain 3	*PITX3*
1706	13,4	14,8	-1,11	0,01089	0,348	220045_at	Neurogenic differentiation 6	*NEUROD6*
2320	3,80	4,20	-1,11	0,01541	0,363	230478_at	Oncoprotein induced transcript 3	*OIT3*
2758	5,94	6,57	-1,11	0,01914	0,379	243322_at	Transcribed locus	-
2911	13,1	14,5	-1,11	0,02045	0,383	1566994_at	Clone GCRG114 mRNA sequence	-
5753	8,31	9,19	-1,11	0,04618	0,439	1562681_at	Keratin associated protein 5-2	*KRTAP5-2*
524	3,42	3,79	-1,11	0,00279	0,291	234852_at	Hypothetical protein LOC648859	*LOC648859*
4010	5,53	6,12	-1,11	0,02936	0,400	236148_at	Full length insert cDNA clone YA80A03	-
3140	6,14	6,79	-1,11	0,02227	0,387	243079_x_at	Transcribed locus	-

Index	Mutated*	Normal*	FC	*P* value	FDR**	Probe set	Description	Gene symbol
1178	5,96	6,59	-1,11	0,00692	0,321	236127_at	Transcribed locus	-
2793	4,90	5,42	-1,11	0,01941	0,380	236291_at	Retinol dehydrogenase 5 (11-cis/9-cis)	*RDH5*
2862	12,9	14,3	-1,11	0,02010	0,383	234356_at	cDNA DKFZP434K028	-
2900	7,70	8,52	-1,11	0,02036	0,383	232863_at	Zinc finger protein 815	*ZNF815*
4013	6,55	7,24	-1,11	0,02939	0,400	1560149_at	Solute carrier family 29, member 2	*SLC29A2*
4768	19,1	21,1	-1,11	0,03614	0,414	237378_at	FLJ46321 protein	*FLJ46321*
3922	5,99	6,62	-1,11	0,02864	0,399	237765_at	Chromosome 14 open reading frame 68	*C14orf68*
4099	6,13	6,78	-1,11	0,03013	0,402	231611_at	Similar to XP_001104477.1	-
2030	4,75	5,25	-1,11	0,01323	0,355	236343_at	Transcribed locus	-
1964	8,04	8,89	-1,11	0,01284	0,355	206812_at	Adrenergic, beta-3-, receptor	*ADRB3*
170	3,25	3,59	-1,11	0,00079	0,244	236618_at	Chromosome 20 open reading frame 132	*C20orf132*
5148	4,04	4,47	-1,11	0,03964	0,421	242723_at	Transcribed locus	-
5358	9,15	10,1	-1,11	0,04201	0,429	241053_at	Hypothetical protein LOC730184	*LOC730184*
2865	5,33	5,89	-1,11	0,02011	0,383	206775_at	Cubilin (intrinsic factor-cobalamin receptor)	*CUBN*
459	2,95	3,26	-1,11	0,00245	0,288	237514_at	cDNA clone IMAGE:5267328	-
1220	3,30	3,65	-1,11	0,00726	0,324	1560284_at	cDNA clone IMAGE:5271145	-
1148	18,6	20,6	-1,11	0,00675	0,321	230157_at	Cadhetrin-like 24	*CDH24*
190	2,89	3,20	-1,11	0,00088	0,244	206676_at	Carcinoembryonic antigen-related cell adhesion molecule 8	*CEACAM8*
1129	3,76	4,15	-1,11	0,00659	0,319	224209_s_at	Guanine deaminase	*GDA*
2118	5,70	6,30	-1,11	0,01389	0,359	1554974_at	Aspartoacylase (aminocyclase) 3	*ACY3*
3588	11,0	12,2	-1,11	0,02589	0,394	242848_x_at	Transcribed locus	-
2093	4,57	5,05	-1,11	0,01367	0,357	1562327_at	cDNA clone IMAGE:4794893	-
3758	6,10	6,74	-1,11	0,02731	0,397	1566232_at	cDNA DKFZp667I0318	-
4585	5,00	5,52	-1,11	0,03465	0,412	230859_at	Transcribed locus	-
3813	13,2	14,6	-1,11	0,02779	0,398	208034_s_at	Protein Z, vitamin K-dependent plasma glycoProtein	*PROZ*
767	14,3	15,8	-1,11	0,00437	0,311	1554580_a_at	WW domain containing E3 ubiquitin protein ligase 2	*WWP2*
1528	3,09	3,41	-1,11	0,00945	0,337	1568755_a_at	cDNA clone IMAGE:5267015	-
669	3,68	4,06	-1,11	0,00384	0,308	1560895_at	Hypothetical LOC645188	*LOC645188*
761	5,07	5,60	-1,11	0,00430	0,308	224539_s_at	Protocadherin alpha subfamily C, 2	*PCDHAC2*
3609	57,3	63,3	-1,11	0,02605	0,394	1553359_at	F-box and leucine-rich repeat protein 18	*FBXL18*
1612	3,69	4,08	-1,11	0,01010	0,343	231642_at	Chromosome 6 open reading frame 201	*C6orf201*
3219	7,36	8,13	-1,11	0,02281	0,387	220776_at	Potassium inwardly-rectifying channel, subfamily J, member 14	*KCNJ14*
4861	12,3	13,6	-1,10	0,03709	0,417	244711_at	Transcribed locus	-
4497	6,89	7,62	-1,10	0,03383	0,411	207316_at	Hyaluronan synthase 1	*HAS1*
3983	4,06	4,48	-1,10	0,02914	0,400	1559033_at	Hypothetical protein LOC255167	*LOC255167*
4369	4,25	4,69	-1,10	0,03251	0,407	1561693_at	Hypothetical gene supported by BC030596	*LOC400794*
1986	12,6	13,9	-1,10	0,01297	0,355	232880_at	cDNA DKFZp434E145	-
1536	4,55	5,03	-1,10	0,00951	0,337	240534_at	Transcribed locus	-
5937	23,4	25,9	-1,10	0,04814	0,443	1553572_a_at	Cytoglobin	*CYGB*
2986	3,34	3,69	-1,10	0,02100	0,384	237268_at	Down syndrome cell adhesion molecule	*DSCAM*
5147	12,4	13,7	-1,10	0,03962	0,421	216145_at	Velo-cardio-facial syndrome 22q11 region mRNA sequence	
3176	24,4	27,0	-1,10	0,02253	0,387	1556722_a_at	Hypothetical protein FLJ33706	*FLJ33706*
5588	17,7	19,6	-1,10	0,04457	0,436	1553656_at	Tectorin beta	*TECTB*
4791	5,98	6,60	-1,10	0,03633	0,415	207072_at	Interleukin 18 receptor accessory protein	*IL18RAP*
5451	8,30	9,17	-1,10	0,04300	0,431	234768_at	cDNA FLJ20865 fis, clone ADKA01850	-
1078	9,17	10,1	-1,10	0,00632	0,319	207228_at	Protein kinase, cAMP-dependent, catalytic, gamma	*PRKACG*
1073	4,08	4,51	-1,10	0,00629	0,319	243393_at	Transcribed locus	-
4418	20,6	22,7	-1,10	0,03306	0,409	208568_at	Melanocortin 2 receptor (adrenocorticotropic hormone)	*MC2R*
2863	5,87	6,48	-1,10	0,02010	0,383	210632_s_at	Sarcoglycan, alpha (50kDa dystrophin-associated glycoprotein)	*SGCA*
4024	10,5	11,6	-1,10	0,02946	0,400	1569461_at	-	-
3959	3,15	3,47	-1,10	0,02891	0,399	206177_s_at	Arginase, liver	*ARG1*
1917	3,71	4,09	-1,10	0,01251	0,355	244091_at	Transcribed locus	-
1492	3,79	4,18	-1,10	0,00919	0,336	231122_x_at	Zinc finger, DHHC-type containing 19	*ZDHHC19*
5862	24,7	27,3	-1,10	0,04740	0,442	206817_x_at	Trinucleotide repeat containing 4	*TNRC4*
1737	5,28	5,83	-1,10	0,01110	0,348	236052_at	Hypothetical LOC388610	*LOC388610*
3574	4,06	4,48	-1,10	0,02581	0,394	216015_s_at	NLR family, pyrin domain containing 3	*NLRP3*
4007	4,99	5,51	-1,10	0,02932	0,400	206465_at	Acyl-CoA synthetase bubblegum family member 1	*ACSBG1*

Index	Mutated*	Normal*	FC	*P* value	FDR**	Probe set	Description	Gene symbol
3286	4,27	4,72	-1,10	0,02341	0,388	1556641_at	Hypothetical protein FLJ37228	*FLJ37228*
3621	12,5	13,8	-1,10	0,02614	0,395	237266_at	Similar to Kv channel interacting protein 2 isoform 4	-
4963	5,29	5,84	-1,10	0,03807	0,419	230693_at	ATPase, Ca++ transporting, cardiac muscle, fast twitch 1	*ATP2A1*
1824	3,25	3,59	-1,10	0,01176	0,351	1561278_at	cDNA clone IMAGE:4829538	-
5121	9,66	10,7	-1,10	0,03942	0,421	233623_at	Clone FLB3107 mRNA sequence	-
2446	8,72	9,63	-1,10	0,01637	0,366	1568734_a_at	HCLS1 binding protein 3	*HS1BP3*
4149	4,20	4,63	-1,10	0,03061	0,403	220064_at	Tetratricopeptide repeat domain 21B	*TTC21B*
3352	5,56	6,14	-1,10	0,02380	0,388	221097_s_at	Calcium-activated potassium channel subunit beta-2	*KCNMB2*
3274	5,44	6,00	-1,10	0,02329	0,388	1553555_at	Taste receptor, type 2, member 38	*TAS2R38*
3474	7,21	7,96	-1,10	0,02493	0,392	243769_at	-	-
3479	19,3	21,3	-1,10	0,02498	0,392	207205_at	Carcinoembryonic antigen-related cell adhesion molecule 4	*CEACAM4*
3146	114	126	-1,10	0,02232	0,387	229356_x_at	INO80 complex homolog 1 (S. cerevisiae)	*INOC1*
3873	6,86	7,57	-1,10	0,02829	0,399	207421_at	Carbonic anhydrase VA, mitochondrial	*CA5A*
4987	9,62	10,6	-1,10	0,03827	0,419	1553900_s_at	POM121-like protein	-
2572	15,1	16,7	-1,10	0,01744	0,371	229561_at	Chromosome 14 open reading frame 121	*C14orf121*
4940	4,23	4,67	-1,10	0,03791	0,419	216085_at	cDNA DKFZP434C153	-
844	3,88	4,28	-1,10	0,00484	0,313	1553059_at	Peptidoglycan recognition protein 3	*PGLYRP3*
5107	11,3	12,5	-1,10	0,03929	0,421	234586_at	Sarcosine dehydrogenase	*SARDH*
4647	10,3	11,4	-1,10	0,03505	0,412	1555550_at	ligand-gated ion channel, zinc activated 1	*LGICZ1*
4320	4,57	5,04	-1,10	0,03216	0,407	223372_at	DnaJ (Hsp40) homolog, subfamily C, member 4	*DNAJC4*
2737	5,66	6,25	-1,10	0,01889	0,377	236151_at	KIAA1853 protein	*KIAA1853*
5737	10,5	11,6	-1,10	0,04601	0,438	210155_at	Myocilin	*MYOC*
4375	17,3	19,1	-1,10	0,03256	0,407	241800_x_at	-	-
5453	33,2	36,6	-1,10	0,04302	0,431	206901_at	Chromosome 19 open reading frame 57	*C19orf57*
4353	5,06	5,58	-1,10	0,03241	0,407	211132_at	-	-
5037	4,68	5,17	-1,10	0,03880	0,420	240611_at	-	-
4700	13,9	15,3	-1,10	0,03549	0,413	1554613_a_at	KIAA0226 protein	*KIAA0226*
5263	6,00	6,62	-1,10	0,04089	0,425	233368_s_at	RAB and DnaJ domain containing	*RBJ*
4457	22,7	25,0	-1,10	0,03348	0,411	237401_at	Actinin, alpha 1	*ACTN1*
3773	8,60	9,49	-1,10	0,02741	0,397	1559426_at	cDNA FLJ39210 fis, clone OCBBF2006154	-
2792	5,78	6,38	-1,10	0,01941	0,380	231998_at	-	-
5377	20,1	22,2	-1,10	0,04217	0,429	206883_x_at	Glycoprotein IX (platelet)	*GP9*
2084	3,81	4,21	-1,10	0,01360	0,356	241197_at	Transcribed locus	-
4314	11,4	12,6	-1,10	0,03211	0,407	207770_x_at	Chorionic somatomammotropin hormone 2	*CSH2*
4223	21,6	23,8	-1,10	0,03129	0,405	1554749_s_at	Chloride channel Kb	*CLCNKB*
2224	3,15	3,47	-1,10	0,01477	0,363	243923_at	Similar to XP_529637.1	-
3358	4,82	5,31	-1,10	0,02384	0,388	220381_at	Rho GTPase activating protein 28	*ARHGAP28*
5998	8,00	8,82	-1,10	0,04877	0,444	243891_at	Transcribed locus	-
4504	12,3	13,5	-1,10	0,03390	0,412	214750_at	Placenta-specific 4	*PLAC4*
5683	15,5	17,1	-1,10	0,04537	0,436	244580_at	Clone IMAGE:4871993, mRNA	-
1114	3,41	3,76	-1,10	0,00652	0,319	233043_at	Hypothetical protein LOC221814	*LOC221814*
2715	3,76	4,15	-1,10	0,01865	0,376	240953_at	Transcribed locus	-
5361	3,65	4,02	-1,10	0,04205	0,429	235960_at	Full length insert cDNA YI38B04	-
4364	4,03	4,44	-1,10	0,03245	0,407	1569782_at	cDNA clone IMAGE:5259303	-
185	2,71	2,99	-1,10	0,00086	0,244	1560174_at	Sperm associated antigen 16	*SPAG16*
5178	9,20	10,1	-1,10	0,03990	0,421	1563228_x_at	Hypothetical protein MGC15523	*MGC15523*
5923	5,11	5,63	-1,10	0,04799	0,443	1562099_at	cDNA clone IMAGE:5425336	-
4557	11,0	12,2	-1,10	0,03445	0,412	221382_at	-	-
2418	5,31	5,86	-1,10	0,01617	0,365	1557369_a_at	Hypothetical protein LOC285401	*LOC285401*
4355	4,83	5,33	-1,10	0,03241	0,407	207636_at	Serpin peptidase inhibitor, clade I (pancpin), member 2	*SERPINI2*
3869	4,77	5,26	-1,10	0,02826	0,399	234526_at	Olfactory receptor, family 51, subfamily B, member 6	*OR51B6*
1034	3,63	4,00	-1,10	0,00601	0,318	1566277_at	Olfactory receptor, family 5, subfamily E, member 1 pseudogene	*OR5E1P*
3533	4,94	5,45	-1,10	0,02540	0,393	234460_at	-	-
6059	33,9	37,4	-1,10	0,04941	0,446	226173_at	TBC1 domain family, member 25	*TBC1D25*
1334	3,47	3,83	-1,10	0,00801	0,328	1562163_at	Full length insert cDNA clone YR43F03	-
4274	5,74	6,33	-1,10	0,03177	0,406	223935_at	Long transient receptor potential channel 5	*TRPM5*
4433	6,67	7,35	-1,10	0,03321	0,409	237434_x_at	Hypothetical gene supported by BC043530	*LOC345222*

Index	Mutated*	Normal*	FC	*P* value	FDR**	Probe set	Description	Gene symbol
4233	3,30	3,64	-1,10	0,03138	0,405	235178_x_at	Establishment of cohesion 1 homolog 2 (S. cerevisiae)	*ESCO2*
2609	5,69	6,27	-1,10	0,01772	0,371	237089_at	-	-
4935	5,43	5,99	-1,10	0,03786	0,419	231756_at	Zona pellucida glycoprotein 4	*ZP4*
1485	5,98	6,59	-1,10	0,00912	0,336	202482_x_at	RAN binding protein 1	*RANBP1*
3563	4,15	4,58	-1,10	0,02570	0,394	1555368_x_at	Zinc finger protein 479	*ZNF479*
5365	8,10	8,93	-1,10	0,04211	0,429	1557258_a_at	B-cell CLL/lymphoma 10	*BCL10*
3708	6,34	6,98	-1,10	0,02681	0,395	238275_at	Huntingtin-associated protein 1 (neuroan 1)	*HAP1*
1918	4,83	5,32	-1,10	0,01251	0,355	236056_s_at	Membrane-associated ring finger (C3HC4) 8	
5976	20,5	22,6	-1,10	0,04854	0,444	222960_at	Calcium channel, voltage-dependent, T type, alpha 1H subunit	*CACNA1H*
2411	5,28	5,82	-1,10	0,01610	0,365	1562549_at	cDNA clone IMAGE:5270889	-
2768	4,27	4,71	-1,10	0,01921	0,379	231685_at	-	-
2940	6,86	7,57	-1,10	0,02062	0,383	234441_at	cDNA DKFZp434F1872	-
4438	5,59	6,17	-1,10	0,03324	0,410	206484_s_at	X-prolyl aminopeptidase (aminopeptidase P) 2, membrane-bound	*XPNPEP2*
2623	4,06	4,48	-1,10	0,01782	0,371	238691_at	Small nucleolar RNA host gene (non-protein coding) 10	*SNHG10*
4997	5,25	5,78	-1,10	0,03837	0,420	234530_s_at	Chromosome 6 open reading frame 12	*C6orf12*
5920	6,42	7,07	-1,10	0,04798	0,443	216175_at	cDNA FLJ21623 fis, clone COL07915	-
1087	8,40	9,26	-1,10	0,00637	0,319	1557660_s_at	Hypothetical protein LOC728883	*LOC728883*
2672	97,3	107	-1,10	0,01827	0,374	224429_x_at	Pseudogene MGC10997	*MGC10997*
5054	8,99	9,90	-1,10	0,03893	0,420	244652_at	Transcribed locus	-
4406	23,1	25,4	-1,10	0,03288	0,408	206047_at	Guanine nucleotide binding protein (G protein), beta polypeptide 3	*GNB3*
4732	5,61	6,18	-1,10	0,03583	0,414	229048_at	Full length insert cDNA clone ZD86A03	-
2704	8,22	9,06	-1,10	0,01854	0,375	242632_at	FYVE, RhoGEF and PH domain containing 2	*FGD2*
5962	17,5	19,3	-1,10	0,04841	0,444	240393_at	Transcribed locus	-
4189	21,5	23,7	-1,10	0,03096	0,404	1553839_at	cDNA DKFZp761B107	-
2917	4,05	4,46	-1,10	0,02048	0,383	221361_at	Olfactory marker protein	*OMP*
5449	50,0	55,1	-1,10	0,04298	0,431	1560439_at	HCG2040376 protein	-
5794	13,2	14,6	-1,10	0,04674	0,441	1558590_at	Methyltransferase 11 domain containing 1	*METT11D1*
4974	21,5	23,7	-1,10	0,03816	0,419	227142_at	Pleckstrin homology domain containing family G member 5	*PLEKHG5*
4687	4,51	4,97	-1,10	0,03538	0,413	1554537_at	Transmembrane protein 126B	*TMEM126B*
5748	4,87	5,36	-1,10	0,04612	0,439	1570138_at	Clone IMAGE:5115854, mRNA	-
947	4,21	4,63	-1,10	0,00543	0,313	1561389_at	cDNA clone IMAGE:4825217	-
4506	5,52	6,08	-1,10	0,03393	0,412	207662_at	T-box 1	*TBX1*
3726	5,87	6,46	-1,10	0,02705	0,397	231126_at	Hypothetical protein LOC339778	*LOC339778*
4712	5,51	6,07	-1,10	0,03565	0,413	1563608_a_at	Potassium channel, subfamily T, member 1	*KCNT1*
4901	4,60	5,07	-1,10	0,03750	0,418	1566844_at	Period 4 pseudogene	*PER4*
4414	3,41	3,76	-1,10	0,03300	0,409	1559633_a_at	Cholinergic receptor, muscarinic 3	*CHRM3*
3362	26,2	28,9	-1,10	0,02389	0,388	224243_at	Apolipoprotein A-V	*APOA5*
3102	6,61	7,28	-1,10	0,02191	0,386	1569911_at	Clone IMAGE:3884408, mRNA	-
4427	13,1	14,4	-1,10	0,03316	0,409	210925_at	Class II, major histocompatibility complex, transactivator	*CIITA*
1972	8,04	8,86	-1,10	0,01289	0,355	240455_at	Transcribed locus	-
3301	5,05	5,56	-1,10	0,02351	0,388	1566228_at	cDNA clone IMAGE:2988896	-
3845	9,03	9,95	-1,10	0,02806	0,399	237643_at	Transcribed locus	-
5841	55,2	60,8	-1,10	0,04720	0,442	223617_x_at	ATPase family, AAA domain containing 3B	*ATAD3B*
1314	5,73	6,31	-1,10	0,00783	0,325	240671_at	Full-length cDNA clone CS0DM007YH16	-
4146	6,90	7,60	-1,10	0,03060	0,403	214229_at	Dynein, axonemal, heavy chain 17	*DNAH17*
2531	4,39	4,84	-1,10	0,01710	0,369	242459_at	Transcribed locus	-
1070	3,26	3,58	-1,10	0,00628	0,319	221146_at	-	-
4050	4,75	5,23	-1,10	0,02968	0,401	210770_s_at	Calcium channel, voltage-dependent, P/Q type, alpha 1A subunit	*CACNA1A*
1637	4,05	4,46	-1,10	0,01031	0,344	1555098_a_at	Calcium channel, voltage-dependent, beta 2 subunit	*CACNB2*
6080	5,86	6,45	-1,10	0,04964	0,446	1558378_a_at	AHNAK nucleoprotein 2	*AHNAK2*
362	3,16	3,48	-1,10	0,00189	0,284	1568802_at	Clone IMAGE:3912773, mRNA sequence	-
5068	49,4	54,4	-1,10	0,03901	0,420	1559638_at	Chromosome 1 open reading frame 200	*C1orf200*
2719	4,52	4,98	-1,10	0,01868	0,376	234121_at	cDNA FLJ20486 fis, clone KAT08039	-
3051	8,42	9,27	-1,10	0,02147	0,385	237922_at	Transcribed locus	-
3791	8,29	9,12	-1,10	0,02761	0,398	1569268_at	Glutamate receptor, ionotropic, N-methyl D-aspartate 2C	*GRIN2C*
3363	6,16	6,78	-1,10	0,02389	0,388	236566_at	Transcribed locus	-
4256	5,23	5,76	-1,10	0,03163	0,406	233178_at	TGFB-induced factor homeobox 2-like, Y-linked	*TGIF2LY*

Index	Mutated*	Normal*	FC	*P* value	FDR**	Probe set	Description	Gene symbol
3288	5,38	5,92	-1,10	0,02342	0,388	1561785_at	Mucin 4, cell surface associated	*MUC4*
5751	3,57	3,93	-1,10	0,04615	0,439	1568634_a_at	Similar to Hypothetical protein MGC38937	*LOC339977*
5132	7,58	8,34	-1,10	0,03950	0,421	216492_at	Killer cell immunoglobulin-like receptor, three domains, X1	*KIR3DX1*
5085	8,80	9,68	-1,10	0,03914	0,420	244833_at	Coiled-coil domain containing 63	*CCDC63*
1230	3,86	4,25	-1,10	0,00731	0,324	1569624_at	Clone IMAGE:4248008, mRNA	-
2087	3,52	3,88	-1,10	0,01362	0,356	1556487_a_at	Chromosome 3 open reading frame 15	*C3orf15*
4298	34,1	37,5	-1,10	0,03198	0,407	1568704_a_at	Calcium homeostasis endoplasmic reticulum protein	*CHERP*
2974	3,13	3,45	-1,10	0,02090	0,384	1561543_at	cDNA clone IMAGE:5299981	-
5182	18,7	20,6	-1,10	0,03995	0,421	1559924_at	cDNA clone IMAGE:5303182	-
5525	12,0	13,2	-1,10	0,04381	0,433	241939_at	IQ motif containing GTPase activating protein 3	*IQGAP3*
1474	6,99	7,69	-1,10	0,00900	0,334	216759_at	HRAS-like suppressor 2	*HRASLS2*
1705	10,9	12,0	-1,10	0,01088	0,348	1563090_at	Coiled-coil domain containing 33	*CCDC33*
3070	3,67	4,04	-1,10	0,02166	0,385	209816_at	Patched homolog 1 (Drosophila)	*PTCH1*
3077	3,99	4,39	-1,10	0,02169	0,385	215534_at	cDNA DKFZp586C1923	-
2046	2,73	3,01	-1,10	0,01330	0,355	242502_at	Potassium voltage-gated channel, subfamily H member 5	*KCNH5*
3496	4,07	4,47	-1,10	0,02514	0,392	1560891_a_at	Full length insert cDNA clone YY75G10	-
4957	9,71	10,7	-1,10	0,03802	0,419	221464_at	Olfactory receptor, family 1, subfamily D, member 2	*OR1D2*
4343	10,6	11,7	-1,10	0,03233	0,407	1552768_at	Calcium/calmodulin-dependent protein kinase kinase 1, alpha	*CAMKK1*
4950	26,4	29,0	-1,10	0,03797	0,419	208052_x_at	Carcinoembryonic antigen-related cell adhesion molecule 3	*CEACAM3*
5467	16,3	18,0	-1,10	0,04318	0,432	219899_x_at	NADPH dependent diflavin oxidoreductase 1	*NDOR1*
3547	5,06	5,56	-1,10	0,02551	0,393	244588_at	-	-
2281	8,01	8,81	-1,10	0,01516	0,363	1558340_at	DIX domain containing 1	*DIXDC1*
992	3,16	3,47	-1,10	0,00571	0,315	1555591_at	PIF1 5'-to-3' DNA helicase homolog (S. cerevisiae)	*PIF1*
5470	10,2	11,3	-1,10	0,04324	0,432	243569_at	Transmembrane protein 174	*TMEM174*
3384	8,95	9,84	-1,10	0,02410	0,389	220635_at	Psoriasis susceptibility 1 candidate 2	*PSORS1C2*
3340	6,12	6,73	-1,10	0,02371	0,388	231477_at	Transcribed locus	-
4015	9,30	10,2	-1,10	0,02939	0,400	1561683_at	cDNA clone IMAGE:5270641	-
2089	3,50	3,85	-1,10	0,01363	0,356	1564029_at	Ubiquitin specific peptidase 49	*USP49*
5251	16,5	18,2	-1,10	0,04067	0,423	230041_at	cDNA FLJ14057 fis, clone HEMBB1000337	-
2158	5,33	5,86	-1,10	0,01423	0,360	233145_at	CUB and Sushi multiple domains 2	*CSMD2*
2458	8,58	9,44	-1,10	0,01650	0,367	222323_at	-	-
3652	4,75	5,23	-1,10	0,02640	0,395	237461_at	NLR family, pyrin domain containing 7	*NLRP7*
5156	8,69	9,55	-1,10	0,03970	0,421	244798_at	Similar to XP_001099403.1	-
1816	5,85	6,43	-1,10	0,01171	0,351	1553347_s_at	Potassium voltage-gated channel subfamily A member 6	*KCNA6*
4928	5,23	5,75	-1,10	0,03779	0,419	1561097_at	cDNA clone IMAGE:5270855	-
1025	3,11	3,42	-1,10	0,00593	0,316	232084_at	Small glutamine rich protein with tetratricopeptide repeats 2	*SGTB*
3379	9,28	10,2	-1,10	0,02405	0,389	228447_at	Splicing factor, arginine/serine-rich 17A	*SFRS17A*
6099	2994	3292	-1,10	0,04979	0,446	202029_x_at	Ribosomal protein L38	*RPL38*
1819	4,50	4,95	-1,10	0,01172	0,351	1555245_s_at	Retinitis pigmentosa 1-like 1	*RP1L1*
5782	7,21	7,93	-1,10	0,04656	0,440	224042_at	Ureidopropionase, beta	*UPB1*
3880	3,95	4,34	-1,10	0,02834	0,399	240212_at	cDNA clone IMAGE:5267797	-
1451	5,23	5,74	-1,10	0,00887	0,334	240005_at	Transcribed locus	-
1240	15,9	17,5	-1,10	0,00736	0,324	206477_s_at	Neuro-oncological ventral antigen 2	*NOVA2*
3944	4,84	5,32	-1,10	0,02879	0,399	211171_s_at	Phosphodiesterase 10A	*PDE10A*
2947	4,06	4,46	-1,10	0,02065	0,383	208162_s_at	Hypothetical protein FLJ10232	*FLJ10232*
3220	4,46	4,90	-1,10	0,02283	0,387	1557389_at	cDNA FLJ32222 fis, clone PLACE6004101	-
3551	5,63	6,19	-1,10	0,02559	0,394	1554978_at	Chromosome 8 open reading frame 68	*C8orf68*
4793	6,89	7,58	-1,10	0,03634	0,415	220585_at	Hexokinase domain containing 1	*HKDC1*
5629	5,17	5,68	-1,10	0,04493	0,436	238888_at	Transcribed locus	-
5878	8,66	9,52	-1,10	0,04753	0,442	241847_at	-	-
6050	13,1	14,3	-1,10	0,04930	0,445	243269_s_at	Chromosome 9 open reading frame 144	*C9orf144*
2919	7,41	8,14	-1,10	0,02049	0,383	234048_s_at	KIAA1632 protein	*KIAA1632*
6010	8,81	9,68	-1,10	0,04884	0,444	227714_s_at	-	-
4731	7,31	8,03	-1,10	0,03583	0,414	219776_s_at	-	-
2230	8,81	9,68	-1,10	0,01482	0,363	236501_at	Sal-like 4 (Drosophila)	*SALL4*
3817	3,51	3,85	-1,10	0,02786	0,399	240415_at	Transcribed locus	-
5644	6,44	7,08	-1,10	0,04504	0,436	224551_s_at	Spectrin, beta, non-erythrocytic 4	*SPTBN4*

Index	Mutated*	Normal*	FC	*P* value	FDR**	Probe set	Description	Gene symbol
2801	4,19	4,60	-1,10	0,01947	0,380	234869_at	-	-
5514	9,82	10,8	-1,10	0,04367	0,433	1564251_at	EMI domain containing 1	*EMID1*
965	3,13	3,44	-1,10	0,00559	0,315	1555454_at	Lipopolysaccharide-induced TNF factor	*LITAF*
5804	4,73	5,19	-1,10	0,04685	0,441	207395_at	Butyrophilin, subfamily 1, member A1	*BTN1A1*
1297	4,94	5,43	-1,10	0,00772	0,325	1564138_at	F-box and WD repeat domain containing 12	*FBXW12*
2873	4,30	4,72	-1,10	0,02018	0,383	1555488_at	-	-
6054	36,8	40,4	-1,10	0,04938	0,446	231629_x_at	Kallikrein-related peptidase 3	*KLK3*
3733	6,28	6,90	-1,10	0,02710	0,397	224244_s_at	Apolipoprotein A-V	*APOA5*
3216	24,3	26,7	-1,10	0,02279	0,387	1569986_x_at	Troponin T type 3 (skeletal, fast)	*TNNT3*
3655	5,98	6,57	-1,10	0,02641	0,395	233777_at	cDNA FLJ12075 fis, clone HEMBB1002425	-
3572	6,14	6,75	-1,10	0,02580	0,394	237840_at	Hypothetical gene supported by BC062774	*LOC388948*
4824	9,48	10,4	-1,10	0,03670	0,416	1554989_at	KIAA0317 protein	*KIAA0317*
4959	10,1	11,1	-1,10	0,03804	0,419	230184_at	cDNA FLJ39179 fis, clone OCBBF2004147	-
4657	8,31	9,12	-1,10	0,03516	0,413	237021_at	Hypothetical protein LOC144486	*LOC144486*
5023	3,49	3,84	-1,10	0,03863	0,420	1560946_at	cDNA FLJ32698 fis, clone TESTI2000427	-
5282	6,08	6,68	-1,10	0,04109	0,425	240264_at	-	-
1789	7,13	7,83	-1,10	0,01146	0,350	240350_at	Transcribed locus	-
5130	6,90	7,58	-1,10	0,03948	0,421	227659_at	Pleckstrin homology domain-containing family A member 3	*PLEKHA3*
5669	5,58	6,13	-1,10	0,04524	0,436	223885_at	Calneuron 1	*CALN1*
1501	7,78	8,55	-1,10	0,00923	0,336	1552872_at	Chromosome X and Y open reading frame 2	*CXYorf2*
1844	4,42	4,86	-1,10	0,01186	0,352	214379_at	Transcribed locus	-
5266	7,75	8,51	-1,10	0,04090	0,425	224135_at	WD repeat domain 87	*WDR87*
720	4,14	4,55	-1,10	0,00412	0,308	222247_at	Putative X-linked retinopathy protein	*DXS542*
5369	4,44	4,87	-1,10	0,04214	0,429	244746_at	Semaphorin-4D	*SEMA6D*
2371	13,8	15,2	-1,10	0,01582	0,364	221292_at	Patched homolog 2 (Drosophila)	*PTCH2*
5412	8,07	8,87	-1,10	0,04253	0,430	231231_at	Formin-like 3	*FMNL3*
2404	4,97	5,46	-1,10	0,01606	0,365	236743_at	1-acylglycerol-3-phosphate O-acyltransferase 6 (lysophosphatidic acid acyltra	*AGPAT6*
1767	3,90	4,28	-1,10	0,01127	0,348	1560011_at	Prostate stem cell antigen	*PSCA*
2635	3,89	4,27	-1,10	0,01794	0,372	216748_at	Pyrin and HIN domain family, member 1	*PYHIN1*
4884	7,40	8,13	-1,10	0,03734	0,418	215974_at	HLA complex group 4 pseudogene 6	*HCG4P6*
1742	4,93	5,41	-1,10	0,01113	0,348	1566032_at	cDNA DKFZp686J0929	-
3990	6,56	7,21	-1,10	0,02918	0,400	232479_at	Chromosome 20 open reading frame 42	*C20orf42*
1201	4,24	4,65	-1,10	0,00710	0,323	1569832_at	cDNA clone IMAGE:5264841	-
5758	15,3	16,8	-1,10	0,04624	0,439	222107_x_at	Leucine zipper, putative tumor suppressor 1	*LZTS1*
4834	5,50	6,03	-1,10	0,03686	0,417	239310_at	Transcribed locus	-
6112	4,64	5,09	-1,10	0,04988	0,446	243687_at	Transcribed locus	-
5289	5,47	6,01	-1,10	0,04115	0,425	1553644_at	Chromosome 14 open reading frame 49	*C14orf49*
3049	3,93	4,31	-1,10	0,02146	0,385	240371_at	Hypothetical protein LOC647107	*LOC647107*
1728	9,37	10,3	-1,10	0,01105	0,348	237431_at	Hypothetical protein LOC731851	*LOC731851*
468	3,15	3,45	-1,10	0,00247	0,288	1554784_at	Contactin 1	*CNTN1*
5065	8,65	9,49	-1,10	0,03898	0,420	1557848_at	cDNA clone IMAGE:5272084	-
2398	7,11	7,80	-1,10	0,01600	0,365	1565783_at	cDNA FLJ36796 fis, clone ADRGL2006817	-
2870	4,94	5,42	-1,10	0,02014	0,383	241341_at	-	-
1540	3,74	4,11	-1,10	0,00953	0,337	220191_at	Gastrokine 1	*GKN1*
2412	4,71	5,17	-1,10	0,01612	0,365	236848_s_at	Testis expressed 13A	*TEX13A*
731	3,71	4,07	-1,10	0,00418	0,308	241290_at	Hypothetical protein LOC729766	*LOC729766*
973	13,7	15,0	-1,10	0,00565	0,315	1559989_at	Tropomyosin 4	*TPM4*
3613	7,97	8,75	-1,10	0,02607	0,394	1568981_at	cDNA clone IMAGE:5277449	-
500	3,34	3,67	-1,10	0,00266	0,289	1555433_at	Solute carrier family 39, member 14	*SLC39A14*
1531	5,24	5,75	-1,10	0,00948	0,337	1567027_at	SH3-domain GRB2-like pseudogene 2	*SH3GLP2*
4258	10,0	11,0	-1,10	0,03164	0,406	208303_s_at	Cytokine receptor-like factor 2	*CRLF2*
2094	7,41	8,12	-1,10	0,01370	0,358	235582_at	E2F transcription factor 2	*E2F2*
2645	10,9	12,0	-1,10	0,01804	0,373	228911_at	ADAM metallopeptidase with thrombospondin type 1 motif, 7	*ADAMTS7*
2823	4,13	4,53	-1,10	0,01971	0,381	231323_at	Proteasome (prosome, macropain) subunit, beta type, 2	*PSMB2*
1293	3,91	4,29	-1,10	0,00770	0,325	215998_at	Clone 24533 mRNA sequence	-
3212	5,22	5,73	-1,10	0,02274	0,387	235357_at	D4, zinc and double PHD fingers, family 3	*DPF3*
2950	23,0	25,2	-1,10	0,02071	0,384	215822_x_at	Myelin transcription factor 1	*MYT1*

Index	Mutated*	Normal*	FC	*P* value	FDR**	Probe set	Description	Gene symbol
1341	2,92	3,20	-1,10	0,00805	0,328	1552458_at	Methyl-CpG binding domain protein 3-like 1	*MBD3L1*
3123	12,3	13,4	-1,10	0,02211	0,387	235455_at	Family with sequence similarity 131, member C	*FAM131C*
4767	5,14	5,63	-1,10	0,03611	0,414	1555799_at	Fc receptor-like 5	*FCRL5*
2164	5,84	6,40	-1,10	0,01432	0,361	231390_at	Hypothetical protein LOC730116	*LOC730116*
1751	4,42	4,84	-1,10	0,01117	0,348	237260_at	cDNA clone IMAGE:4838968	-
4641	10,5	11,5	-1,10	0,03499	0,412	227513_s_at	Leucine rich repeat (in FLII) interacting protein 1	*LRRFIP1*
4490	7,78	8,53	-1,10	0,03377	0,411	216992_s_at	Glutamate receptor, metabotropic 8	*GRM8*
5580	5,46	5,99	-1,10	0,04449	0,436	1566486_at	cDNA DKFZp547M116	-
5503	3,98	4,37	-1,10	0,04357	0,433	237820_at	Transcribed locus	-
3644	10,4	11,4	-1,10	0,02634	0,395	206887_at	Chemokine binding protein 2	*CCBP2*
2902	6,23	6,84	-1,10	0,02040	0,383	229922_at	G protein regulated inducer of neurite outgrowth 1	*GPRIN1*
4228	10,2	11,1	-1,10	0,03133	0,405	1562263_at	Lysyl oxidase-like 2	*LOXL2*
1559	8,20	8,99	-1,10	0,00964	0,338	1561270_at	cDNA clone IMAGE:5271318	-
469	7,18	7,87	-1,10	0,00249	0,289	241074_at	Similar to Ig heavy chain V region M315 precursor	-
4640	6,41	7,03	-1,10	0,03499	0,412	1561611_at	Full length insert cDNA clone ZD67H01	-
4230	10,0	11,0	-1,10	0,03134	0,405	1554475_a_at	Chromosome 19 open reading frame 47	*C19orf47*
958	4,30	4,71	-1,10	0,00553	0,315	233033_at	Zinc finger E-box binding homeobox 2	*ZEB2*
3766	3,12	3,42	-1,10	0,02737	0,397	1552875_a_at	CD200 receptor 1	*CD200R1*
3672	4,26	4,67	-1,10	0,02652	0,395	1553796_at	Hypothetical locus FLJ30594	*FLJ30594*
6117	11,5	12,6	-1,10	0,04995	0,446	215055_at	UDP-GlcNAc:betaGal beta-1,3-N-acetylglucosaminyltransferase-like 1	*B3GNTL1*
5967	7,50	8,22	-1,10	0,04846	0,444	221990_at	Paired box 8	*PAX8*
1182	3,95	4,33	-1,10	0,00696	0,322	1567250_at	Olfactory receptor, family 10, subfamily A, member 3	*OR10A3*
3022	4,21	4,62	-1,10	0,02128	0,385	234174_at	-	-
4905	6,79	7,45	-1,10	0,03751	0,418	207960_at	-	-
2626	3,71	4,07	-1,10	0,01787	0,372	216328_at	Sialic acid binding Ig-like lectin 8	*SIGLEC8*
1320	3,41	3,74	-1,10	0,00787	0,326	1561604_at	cDNA clone IMAGE:4796102	-
4519	7,04	7,72	-1,10	0,03406	0,412	239976_at	Acyl-Coenzyme A dehydrogenase family, member 9	*ACAD9*
4065	5,41	5,93	-1,10	0,02982	0,401	234766_at	Olfactory receptor, family 8, subfamily D, member 2	*OR8D2*
5406	12,5	13,7	-1,10	0,04243	0,429	1560001_at	Similar to 40S ribosomal protein S3a	*LOC649314*
4471	3,36	3,68	-1,10	0,03359	0,411	233753_at	Splicing factor, arginine/serine-rich 15	*SFRS15*
5732	4,69	5,14	-1,10	0,04598	0,438	1555738_at	CKLF-like MARVEL transmembrane domain containing 1	*CMTM1*
3917	7,70	8,44	-1,10	0,02861	0,399	1555308_at	Caspase recruitment domain family, member 14	*CARD14*
5461	8,30	9,09	-1,10	0,04313	0,432	231583_at	Keratin 74	*KRT74*
2265	4,51	4,95	-1,10	0,01506	0,363	1562865_at	Clone IMAGE:4279626, mRNA	-
4704	13,0	14,2	-1,10	0,03557	0,413	204356_at	LIM domain kinase 1	*LIMK1*
4392	5,47	5,99	-1,10	0,03272	0,407	228017_s_at	Chromosome 20 open reading frame 58	*C20orf58*
5247	47,5	52,1	-1,10	0,04062	0,423	235817_at	Transmembrane protein 184A	*TMEM184A*
3921	5,19	5,68	-1,10	0,02863	0,399	211018_at	Lanosterol synthase (2,3-oxidosqualene-lanosterol cyclase)	*LSS*
5291	39,4	43,2	-1,10	0,04119	0,426	1553262_a_at	Urotensin 2 receptor	*UTS2R*
6068	4,47	4,90	-1,10	0,04951	0,446	239989_at	Transcribed locus	-
4444	5,64	6,18	-1,10	0,03339	0,411	1563539_at	cDNA DKFZp762C115	-
1415	3,55	3,88	-1,10	0,00856	0,331	233830_at	Hypothetical protein LOC90246	*LOC90246*
2007	3,65	4,00	-1,10	0,01309	0,355	237905_at	Keratin 25	*KRT25*
5611	8,83	9,67	-1,10	0,04474	0,436	233726_at	cDNA DKFZp586C1523	-
3276	5,46	5,98	-1,10	0,02333	0,388	233361_at	Ankyrin repeat domain 44	*ANKRD44*
4895	2,86	3,14	-1,10	0,03741	0,418	216703_at	-	-
5548	8,73	9,57	-1,10	0,04411	0,435	1552503_at	Fraser syndrome 1	*FRAS1*
2693	4,05	4,43	-1,10	0,01847	0,375	239988_at	Transcribed locus	-
3947	9,37	10,3	-1,10	0,02882	0,399	233991_at	Clone 24894 mRNA sequence	-
2566	4,17	4,56	-1,10	0,01740	0,371	240011_at	Tau tubulin kinase 2	*TTBK2*
5153	6,28	6,88	-1,10	0,03965	0,421	1566097_at	cDNA DKFZp761K02121	-
2984	3,51	3,85	-1,10	0,02097	0,384	1558652_at	Hypothetical protein FLJ10081	*FLJ10081*
3629	6,11	6,69	-1,10	0,02623	0,395	217128_s_at	Calcium/calmodulin-dependent protein kinase IG	*CAMK1G*
3976	8,32	9,11	-1,10	0,02907	0,400	222928_s_at	Retinoic acid induced 16	*RAI16*
4550	10,6	11,7	-1,10	0,03440	0,412	216739_at	cDNA FLJ20874 fis, clone ADKA02818	-
3846	6,59	7,21	-1,09	0,02807	0,399	220528_at	Vanin 3	*VNN3*
2079	2,99	3,27	-1,09	0,01358	0,356	1553736_at	Coiled-coil domain containing 131	*CCDC131*

Index	Mutated*	Normal*	FC	*P* value	FDR**	Probe set	Description	Gene symbol
6024	4,17	4,57	-1,09	0,04901	0,445	243822_at	Transcribed locus	-
4877	5,49	6,01	-1,09	0,03724	0,417	223893_at	Enamelin	*ENAM*
3912	8,32	9,11	-1,09	0,02856	0,399	206981_at	Sodium channel, voltage-gated, type IV, alpha subunit	*SCN4A*
5067	12,3	13,5	-1,09	0,03900	0,420	1566474_at	cDNA DKFZp451O1018	-
3737	10,7	11,7	-1,09	0,02713	0,397	1563816_at	GRB2-associated binding protein family, member 4	*GAB4*
3348	10,1	11,0	-1,09	0,02378	0,388	240767_x_at	Vacuolar protein sorting 13 homolog A (S. cerevisiae)	*VPS13A*
2852	3,62	3,96	-1,09	0,02000	0,383	238410_x_at	Transcribed locus	-
2924	4,13	4,53	-1,09	0,02050	0,383	216886_at	Cholinergic receptor, nicotinic, alpha 4	*CHRNA4*
5788	2,97	3,25	-1,09	0,04660	0,440	201062_at	Stomatin	*STOM*
4383	11,5	12,5	-1,09	0,03265	0,407	230488_s_at	BPR	*LOC138948*
4952	91,4	100	-1,09	0,03799	0,419	179_at	Postmeiotic segregation increased 2-like 11	*PMS2L11*
682	4,59	5,02	-1,09	0,00391	0,308	243468_at	Expressed in prostate and testis	*PATE*
2031	5,15	5,64	-1,09	0,01323	0,355	234743_at	LIM domains containing 1	*LIMD1*
4104	25,7	28,1	-1,09	0,03016	0,402	234241_at	-	-
1115	4,89	5,35	-1,09	0,00653	0,319	214979_at	ATP-binding cassette, sub-family C (CFTR/MRP), member 3	*ABCC3*
6108	16,8	18,4	-1,09	0,04986	0,446	232192_at	Hypothetical protein LOC153811	*LOC153811*
4267	4,13	4,52	-1,09	0,03174	0,406	235995_at	-	-
2642	4,24	4,64	-1,09	0,01801	0,373	240396_at	Interleukin 20 receptor, alpha	*IL20RA*
2683	3,91	4,28	-1,09	0,01837	0,374	215762_at	Transcribed locus	-
1642	3,27	3,57	-1,09	0,01035	0,344	223835_x_at	Orthopedia homeobox	*OTP*
5199	14,0	15,3	-1,09	0,04011	0,422	1552419_s_at	Tubulin tyrosine ligase-like family, member 10	*TTLL10*
5859	17,4	19,0	-1,09	0,04737	0,442	243411_at	Polymerase (DNA-directed), delta interacting protein 3	*POLDIP3*
5512	9,92	10,9	-1,09	0,04366	0,433	244858_at	TGFB-induced factor homeobox 1	*TGIF1*
4936	4,77	5,22	-1,09	0,03786	0,419	237669_at	-	-
5754	4,33	4,74	-1,09	0,04619	0,439	1557682_a_at	Hypothetical protein LOC284688	-
3592	33,5	36,7	-1,09	0,02591	0,394	206508_at	CD70 molecule	*CD70*
2483	3,24	3,55	-1,09	0,01673	0,367	224533_s_at	Chromosome 6 open reading frame 142	*C6orf142*
475	3,02	3,30	-1,09	0,00253	0,289	208288_at	ATP-binding cassette, sub-family B (MDR/TAP), member 11	*ABCB11*
2646	5,00	5,47	-1,09	0,01804	0,373	221442_at	Melanocortin 3 receptor	*MC3R*
2976	4,67	5,11	-1,09	0,02092	0,384	229967_at	CKLF-like MARVEL transmembrane domain containing 2	*CMTM2*
5882	9,04	9,89	-1,09	0,04759	0,442	216018_at	Ring finger protein 5	*RNF5*
3513	4,11	4,50	-1,09	0,02522	0,392	242821_at	Chromosome 9 open reading frame 93	*C9orf93*
5598	8,65	9,46	-1,09	0,04464	0,436	1556620_at	Full length insert cDNA clone YT69G03	-
3665	5,34	5,84	-1,09	0,02649	0,395	239030_at	-	-
4083	3,42	3,74	-1,09	0,02999	0,402	230719_at	Transcribed locus	-
3671	4,54	4,97	-1,09	0,02652	0,395	1560896_a_at	Full length insert cDNA clone ZD42A09	-
4084	7,40	8,09	-1,09	0,02999	0,402	237323_at	Hexokinase domain containing 1	*HKDC1*
5419	4,62	5,05	-1,09	0,04258	0,430	1558300_at	EF-hand calcium binding domain 5	*EFCAB5*
5984	9,53	10,4	-1,09	0,04860	0,444	240023_at	Transcribed locus	-
957	3,11	3,40	-1,09	0,00552	0,315	243335_at	-	-
4209	3,38	3,70	-1,09	0,03114	0,404	234839_at	cDNA DKFZp564I103	-
5408	5,33	5,83	-1,09	0,04248	0,429	203784_s_at	DEAD (Asp-Glu-Ala-Asp) box polypeptide 28	*DDX28*
3261	15,3	16,7	-1,09	0,02315	0,388	1557666_s_at	Chromosome 9 open reading frame 98	*C9orf98*
5700	11,0	12,0	-1,09	0,04564	0,438	1561542_at	-	-
5418	4,58	5,01	-1,09	0,04258	0,430	1560870_a_at	Full length insert cDNA clone ZD45F06	-
885	2,75	3,01	-1,09	0,00511	0,313	1552732_at	Actin-binding Rho activating protein	*ABRA*
2909	3,75	4,10	-1,09	0,02045	0,383	216120_s_at	ATPase, Ca++ transporting, plasma membrane 2	*ATP2B2*
5189	6,57	7,18	-1,09	0,03998	0,421	244199_at	Twinfilin, actin-binding protein, homolog 1 (Drosophila)	*TWF1*
3760	6,60	7,22	-1,09	0,02734	0,397	1561293_at	cDNA clone IMAGE:5296640	-
5437	4,16	4,55	-1,09	0,04284	0,431	236747_at	-	-
4751	4,57	5,00	-1,09	0,03600	0,414	1562984_at	Clone IMAGE:5171167, mRNA	-
6110	7,65	8,37	-1,09	0,04987	0,446	228978_at	Hypothetical LOC645722	*LOC645722*
2937	2,88	3,15	-1,09	0,02060	0,383	210498_at	Clathrin, heavy chain (Hc)	*CLTC*
5224	3,70	4,04	-1,09	0,04040	0,423	243368_at	Transcribed locus	-
6002	9,61	10,5	-1,09	0,04877	0,444	244713_at	Transcribed locus	-
1859	35,3	38,6	-1,09	0,01198	0,352	1556900_at	Hypothetical protein LOC149773	*LOC149773*
2243	3,51	3,84	-1,09	0,01492	0,363	238236_at	Transcribed locus	-

Index	Mutated*	Normal*	FC	*P* value	FDR**	Probe set	Description	Gene symbol
5243	3,96	4,33	-1,09	0,04060	0,423	1558845_at	cDNA FLJ40376 fis, clone TESTI2035121	-
5940	4,09	4,47	-1,09	0,04815	0,443	217624_at	PDGFA associated protein 1	*PDAP1*
315	3,13	3,43	-1,09	0,00159	0,272	217345_at	-	-
4772	3,90	4,27	-1,09	0,03619	0,414	1560905_at	Full length insert cDNA clone YB65G12	-
4046	4,57	4,99	-1,09	0,02965	0,401	207637_at	WSC domain containing 2	*WSCD2*
3686	4,24	4,63	-1,09	0,02662	0,395	1565896_at	cDNA clone IMAGE:5266332	-
3607	5,13	5,61	-1,09	0,02603	0,394	217484_at	Complement component (3b/4b) receptor 1 (Knops blood group)	*CR1*
4821	3,92	4,29	-1,09	0,03667	0,416	237590_at	-	-
666	3,81	4,17	-1,09	0,00384	0,308	1557886_at	Coiled-coil domain containing 46	*CCDC46*
2677	4,04	4,42	-1,09	0,01832	0,374	1563018_at	T-box 5	*TBX5*
1088	3,73	4,08	-1,09	0,00637	0,319	233338_at	Similar to Nucleosome-binding protein 1	*LOC653135*
4130	6,35	6,94	-1,09	0,03040	0,402	221415_s_at	Gap junction protein, alpha 10, 59kDa	*GJA10*
6089	5,71	6,24	-1,09	0,04971	0,446	226904_at	Solute carrier family 6, member 10	*SLC6A10P*
5499	4,20	4,59	-1,09	0,04353	0,433	241258_at	Transcribed locus	-
3658	3,86	4,22	-1,09	0,02641	0,395	211690_at	Ribosomal protein S6	*RPS6*
5769	11,6	12,7	-1,09	0,04635	0,439	207609_s_at	Cytochrome P450, family 1, subfamily A, polypeptide 2	*CYP1A2*
3916	4,41	4,82	-1,09	0,02860	0,399	1556578_a_at	Hypothetical gene supported by AK056507	*FLJ31945*
1051	5,43	5,93	-1,09	0,00614	0,319	1560181_at	Clone IMAGE:5752728, mRNA	-
3118	4,47	4,88	-1,09	0,02205	0,387	234618_at	cDNA DKFZp586M151	-
3878	5,36	5,86	-1,09	0,02833	0,399	237468_at	Transcribed locus	-
2390	31,2	34,0	-1,09	0,01594	0,365	213442_x_at	SAM pointed domain containing ets transcription factor	*SPDEF*
4211	4,96	5,42	-1,09	0,03117	0,405	1555792_a_at	Coiled-coil domain containing 116	*CCDC116*
3562	7,33	8,01	-1,09	0,02570	0,394	215816_at	Glucuronidase, beta/immunoglobulin lambda-like polypeptide 1 pseudogene	*LOC91316*
4033	4,20	4,59	-1,09	0,02955	0,401	240678_at	Transcribed locus	-
1037	4,03	4,41	-1,09	0,00602	0,318	208434_at	Myelodysplasia syndrome 1	*MDS1*
4463	3,53	3,86	-1,09	0,03352	0,411	220025_at	T-box, brain, 1	*TBR1*
4447	3,06	3,34	-1,09	0,03340	0,411	206835_at	Statherin	*STATH*
1638	3,96	4,33	-1,09	0,01032	0,344	1569418_at	cDNA clone IMAGE:4894320	-
6121	4,83	5,27	-1,09	0,04998	0,446	1556907_at	Zinc finger protein 474	*ZNF474*
1648	7,68	8,39	-1,09	0,01037	0,344	1553637_s_at	Transmembrane and coiled-coil domains 5	*TMCO5*
4847	4,57	4,99	-1,09	0,03693	0,417	205152_at	Solute carrier family 6, member 1	*SLC6A1*
5929	9,12	9,96	-1,09	0,04806	0,443	1556355_x_at	Ral guanine nucleotide dissociation stimulator-like 3	*RGL3*
5290	15,8	17,3	-1,09	0,04116	0,425	227381_at	Cerebral endothelial cell adhesion molecule 1	*CEECAM1*
5264	17,8	19,4	-1,09	0,04089	0,425	217219_at	cDNA DKFZP434A062	-
2584	4,42	4,83	-1,09	0,01753	0,371	240935_at	-	-
3668	27,3	29,8	-1,09	0,02652	0,395	1560300_a_at	DMRT-like family C1	*DMRTC1*
5698	6,98	7,63	-1,09	0,04560	0,438	242155_x_at	Transcribed locus	-
2104	3,95	4,31	-1,09	0,01379	0,358	240177_at	Transcribed locus	-
5071	6,02	6,57	-1,09	0,03903	0,420	1565772_at	p21/Cdc42/Rac1-activated kinase 1 (STE20 homolog, yeast)	*PAK1*
2417	3,61	3,94	-1,09	0,01617	0,365	216201_at	cDNA FLJ21586 fis, clone COL06920	-
5543	32,7	35,7	-1,09	0,04407	0,435	242151_at	Transcribed locus	-
2439	9,82	10,7	-1,09	0,01630	0,365	239702_x_at	-	-
5989	4,79	5,23	-1,09	0,04866	0,444	240783_at	Transcribed locus	-
1462	3,42	3,73	-1,09	0,00894	0,334	1568647_at	cDNA clone IMAGE:4426835	-
5576	4,60	5,02	-1,09	0,04443	0,436	240650_at	Calcium channel, voltage-dependent, R type, alpha 1E subunit	*CACNA1E*
3081	5,71	6,23	-1,09	0,02171	0,385	237824_at	Transcribed locus	-
884	4,87	5,31	-1,09	0,00511	0,313	1552842_at	Heparan sulfate 6-O-sulfotransferase 3	*HS6ST3*
4357	5,61	6,13	-1,09	0,03241	0,407	207894_s_at	T-cell leukemia/lymphoma 6	*TCL6*
4756	3,90	4,26	-1,09	0,03605	0,414	232655_at	UDP glucuronosyltransferase 1 family, polypeptide A6	*UGT1A6*
3843	14,9	16,3	-1,09	0,02806	0,399	240114_s_at	Transmembrane protein 174	*TMEM174*
1873	3,23	3,52	-1,09	0,01212	0,354	240986_at	Transcribed locus	-
1307	4,08	4,46	-1,09	0,00779	0,325	236374_at	Cortexin 3	*CTXN3*
2965	3,74	4,08	-1,09	0,02083	0,384	1563502_at	Zinc finger, DHHC-type containing 2	*ZDHHC2*
2840	4,43	4,83	-1,09	0,01982	0,382	1553534_at	NLR family, pyrin domain containing 10	*NLRP10*
4296	4,30	4,70	-1,09	0,03194	0,407	1561018_at	Full length insert cDNA clone ZD44H09	-
4607	4,21	4,59	-1,09	0,03481	0,412	231364_at	cDNA clone IMAGE:4796864	-
5809	4,61	5,03	-1,09	0,04690	0,441	240346_at	Hypothetical LOC644222	*LOC644222*

Index	Mutated*	Normal*	FC	*P* value	FDR**	Probe set	Description	Gene symbol
3952	7,21	7,87	-1,09	0,02888	0,399	1563805_a_at	Family with sequence similarity 83, member C	*FAM83C*
5536	5,97	6,51	-1,09	0,04395	0,434	221072_at	Chromosome 9 open reading frame 31	*C9orf31*
1784	4,11	4,49	-1,09	0,01139	0,349	1562066_at	Hypothetical protein LOC286144	*LOC286144*
5221	7,73	8,44	-1,09	0,04037	0,423	221346_at	Olfactory receptor, family 10, subfamily J, member 1	*OR10J1*
2798	4,34	4,74	-1,09	0,01944	0,380	221347_at	Cholinergic receptor, muscarinic 5	*CHRM5*
4218	8,32	9,08	-1,09	0,03125	0,405	205973_at	Fasciculation and elongation protein zeta 1 (zygin I)	*FEZ1*
1105	3,76	4,10	-1,09	0,00646	0,319	AFFX-ThrX-5_a	-	-
1959	2,85	3,12	-1,09	0,01281	0,355	204721_s_at	DnaJ (Hsp40) homolog, subfamily C, member 6	*DNAJC6*
919	3,01	3,28	-1,09	0,00529	0,313	230517_at	Similar to GLI-Kruppel family member HKR1	*tcag7.1196*
5651	6,60	7,21	-1,09	0,04507	0,436	1561060_at	cDNA clone IMAGE:5298862	-
5678	4,00	4,37	-1,09	0,04533	0,436	237889_s_at	Hypothetical LOC553137	*LOC553137*
5487	4,83	5,27	-1,09	0,04343	0,433	1559674_at	Zinc finger protein 333	*ZNF333*
3633	10,7	11,7	-1,09	0,02627	0,395	219746_at	D4, zinc and double PHD fingers, family 3	*DPF3*
5436	5,30	5,78	-1,09	0,04283	0,431	241383_at	Similar to Hypothetical protein A930006D11	*LOC201181*
1413	18,0	19,7	-1,09	0,00854	0,330	204626_s_at	Integrin, beta 3 (platelet glycoprotein IIIa, antigen CD61)	*ITGB3*
4913	4,39	4,79	-1,09	0,03757	0,418	219621_at	Claspin homolog (Xenopus laevis)	*CLSPN*
4031	8,04	8,77	-1,09	0,02952	0,400	231651_at	cDNA FLJ36110 fis, clone TESTI2021939	-
4923	3,81	4,15	-1,09	0,03772	0,419	211319_at	-	-
3973	3,84	4,19	-1,09	0,02904	0,400	1556084_at	Heterogeneous nuclear ribonucleoprotein M	*HNRPM*
3099	2,78	3,03	-1,09	0,02188	0,386	207523_at	Chromosome 6 open reading frame 10	*C6orf10*
926	3,06	3,33	-1,09	0,00533	0,313	241072_s_at	-	-
3985	3,82	4,16	-1,09	0,02914	0,400	241297_at	-	-
4489	11,5	12,6	-1,09	0,03377	0,411	217711_at	TEK tyrosine kinase, endothelial	*TEK*
2547	3,77	4,11	-1,09	0,01721	0,369	1569680_at	cDNA clone IMAGE:4288701	-
3799	3,76	4,10	-1,09	0,02767	0,398	207315_at	CD226 molecule	*CD226*
2409	3,72	4,06	-1,09	0,01610	0,365	231675_s_at	Alcohol dehydrogenase 4 (class II), pi polypeptide	*ADH4*
2725	13,9	15,2	-1,09	0,01872	0,376	239076_at	Septin 13	*SEPT13*
4956	7,05	7,69	-1,09	0,03802	0,419	243883_at	Matrix metallopeptidase 15 (membrane-inserted)	*MMP15*
2923	5,63	6,14	-1,09	0,02050	0,383	242421_at	Transcribed locus	-
591	3,07	3,35	-1,09	0,00327	0,301	1557802_at	Clone IMAGE:5547568, mRNA	-
3924	3,97	4,33	-1,09	0,02868	0,399	233447_at	Chromosome 6 open reading frame 86	*C6orf86*
3804	35,2	38,4	-1,09	0,02770	0,398	1557371_a_at	Hypothetical protein LOC158376	*LOC158376*
5578	11,3	12,3	-1,09	0,04447	0,436	204397_at	Echinoderm microtubule associated protein like 2	*EML2*
4552	4,60	5,02	-1,09	0,03441	0,412	215297_at	Hypothetical locus LOC441204	*LOC441204*
4400	3,39	3,69	-1,09	0,03280	0,408	237462_at	Transcribed locus	-
4840	7,14	7,79	-1,09	0,03690	0,417	215841_at	Guanylate cyclase activator 1B (retina)	*GUCA1B*
5781	5,84	6,37	-1,09	0,04654	0,440	1563324_at	Hypothetical protein LOC389457	*LOC389457*
3841	4,94	5,39	-1,09	0,02805	0,399	211803_at	Cyclin-dependent kinase 2	*CDK2*
1655	4,76	5,19	-1,09	0,01045	0,345	215110_at	Mannose-binding lectin (protein A) 1, pseudogene 1	*MBL1P1*
2983	3,70	4,03	-1,09	0,02097	0,384	237150_at	Transcribed locus	-
1379	4,67	5,10	-1,09	0,00830	0,329	1552854_a_at	Hypothetical protein FLJ32784	*FLJ32784*
3356	4,55	4,96	-1,09	0,02383	0,388	241259_at	GRB2-associated binding protein 3	*GAB3*
4532	4,42	4,82	-1,09	0,03421	0,412	229565_x_at	T-box 3 (ulnar mammary syndrome)	*TBX3*
1080	5,58	6,08	-1,09	0,00632	0,319	1557702_at	cDNA clone IMAGE:5265308	-
3781	4,42	4,82	-1,09	0,02752	0,398	231314_at	Similar to XP_530643.1	-
615	6,80	7,41	-1,09	0,00348	0,308	1561976_at	Chromosome 1 open reading frame 167	*C1orf167*
4062	9,24	10,1	-1,09	0,02980	0,401	242373_at	Transcribed locus	-
2809	5,24	5,71	-1,09	0,01954	0,380	238248_at	Uromodulin (uromucoid, Tamm-Horsfall glycoprotein)	*UMOD*
3751	3,46	3,77	-1,09	0,02728	0,397	224238_at	-	-
1392	2,62	2,85	-1,09	0,00842	0,330	1557030_at	GRB2-associated binding protein 1	*GAB1*
2302	3,79	4,13	-1,09	0,01529	0,363	232506_s_at	Chromosome 15 open reading frame 41	*C15orf41*
1449	6,68	7,28	-1,09	0,00883	0,333	217450_at	-	-
2059	23,7	25,8	-1,09	0,01343	0,356	1562026_at	WNK lysine deficient protein kinase 2	*WNK2*
4718	4,18	4,55	-1,09	0,03570	0,413	1566123_at	Fatty acid binding protein 6, ileal (gastrotropin)	*FABP6*
2018	4,44	4,84	-1,09	0,01317	0,355	238088_at	cDNA clone IMAGE:5259414	-
4244	4,50	4,91	-1,09	0,03149	0,406	239356_at	Clone IMAGE:1257951, mRNA sequence	-
5880	3,66	3,99	-1,09	0,04754	0,442	243517_at	-	-

Index	Mutated*	Normal*	FC	*P* value	FDR**	Probe set	Description	Gene symbol
3018	4,22	4,60	-1,09	0,02127	0,385	1563316_at	Clone IMAGE:5748207, mRNA	-
4466	4,40	4,79	-1,09	0,03355	0,411	207213_s_at	Ubiquitin specific peptidase 2	*USP2*
3889	6,84	7,45	-1,09	0,02843	0,399	230059_at	Deformed epidermal autoregulatory factor 1 (Drosophila)	*DEAF1*
4478	5,68	6,19	-1,09	0,03367	0,411	243975_at	Clone IMAGE:5763888, mRNA	-
2221	4,52	4,92	-1,09	0,01474	0,363	208046_at	Histone cluster 1, H4a	*HIST1H4A*
4804	6,32	6,88	-1,09	0,03647	0,415	1566622_at	cDNA DKFZp667F0113	-
2612	4,28	4,66	-1,09	0,01774	0,371	242179_s_at	Tetraspanin 16	*TSPAN16*
5911	6,26	6,82	-1,09	0,04793	0,443	221105_at	-	-
5146	14,0	15,2	-1,09	0,03962	0,421	224100_s_at	Dihydropyrimidinase-like 5	*DPYSL5*
1929	3,93	4,28	-1,09	0,01257	0,355	1555729_a_at	CD209 molecule	*CD209*
1943	5,16	5,62	-1,09	0,01267	0,355	210753_s_at	EPH receptor B1	*EPHB1*
4172	3,36	3,66	-1,09	0,03084	0,404	221886_at	DENN/MADD domain containing 2A	*DENND2A*
6063	5,67	6,17	-1,09	0,04944	0,446	221707_s_at	Vacuolar protein sorting 53 homolog (S. cerevisiae)	*VPS53*
3911	5,58	6,08	-1,09	0,02855	0,399	1566039_a_at	Hypothetical gene supported by BC036933	*LOC401220*
3179	5,68	6,18	-1,09	0,02254	0,387	1565671_a_at	Hypothetical gene supported by NM_001531	*LOC391142*
3435	6,73	7,33	-1,09	0,02458	0,391	1560935_s_at	Hypothetical protein LOC284669	*LOC284669*
3181	4,24	4,62	-1,09	0,02255	0,387	1555988_a_at	Hypothetical protein LOC126536	*LOC126536*
5166	2,85	3,10	-1,09	0,03981	0,421	1565833_at	-	-
3311	11,0	12,0	-1,09	0,02355	0,388	215487_x_at	Hypothetical gene supported by BC042812	*LOC728882*
1846	3,01	3,28	-1,09	0,01189	0,352	206884_s_at	Sciellin	*SCEL*
2620	3,96	4,32	-1,09	0,01781	0,371	244606_at	ATPase, Na+/K+ transporting, alpha 1 polypeptide	*ATP1A1*
5367	3,59	3,91	-1,09	0,04214	0,429	241121_at	Transcribed locus	-
5720	3,21	3,50	-1,09	0,04580	0,438	224045_x_at	Chromosome 18 open reading frame 2	*C18orf2*
4374	3,55	3,86	-1,09	0,03253	0,407	237805_at	Hypothetical protein LOC729296	*LOC729296*
2756	3,54	3,86	-1,09	0,01910	0,379	237375_at	Similar to XP_530528.1	-
3482	5,25	5,71	-1,09	0,02500	0,392	207990_x_at	Acrosomal vesicle protein 1	*ACRV1*
2062	3,39	3,69	-1,09	0,01345	0,356	1562941_at	cDNA clone IMAGE:5288253	-
2830	3,87	4,21	-1,09	0,01974	0,381	206603_at	Solute carrier family 2, member 4	*SLC2A4*
3048	3,12	3,40	-1,09	0,02146	0,385	1570284_x_at	Clone IMAGE:4696056, mRNA	-
3683	31,2	34,0	-1,09	0,02659	0,395	231792_at	Myosin light chain kinase 2, skeletal muscle	*MYLK2*
5729	5,32	5,79	-1,09	0,04595	0,438	1563255_at	Hypothetical protein LOC728883	*LOC728883*
5117	9,53	10,4	-1,09	0,03940	0,421	207185_at	Solute carrier family 10, member 1	*SLC10A1*
6019	4,33	4,71	-1,09	0,04899	0,445	243425_at	Transcribed locus	-
5100	3,91	4,25	-1,09	0,03923	0,420	241048_at	Transcribed locus	-
3591	3,99	4,34	-1,09	0,02591	0,394	206244_at	Complement component (3b/4b) receptor 1 (Knops blood group)	*CR1*
3250	20,0	21,7	-1,09	0,02307	0,388	1556747_a_at	cDNA FLJ39784 fis, clone SPLEN2002314	-
5286	4,52	4,92	-1,09	0,04112	0,425	231668_x_at	Immunoglobulin heavy constant gamma 1 (G1m marker)	*IGHG1*
5201	8,31	9,05	-1,09	0,04011	0,422	1561336_at	Deoxyribonuclease I-like 3	*DNASE1L3*
5797	24,2	26,4	-1,09	0,04677	0,441	1557964_at	Eukaryotic translation initiation factor 4 gamma, 2	*EIF4G2*
2120	3,70	4,03	-1,09	0,01391	0,359	230953_at	cDNA clone IMAGE:4824446	-
4753	6,22	6,77	-1,09	0,03602	0,414	1562433_at	Hypothetical protein FLJ10489	*FLJ10489*
4064	3,89	4,23	-1,09	0,02981	0,401	1570653_at	Similar to v-Myb myeloblastosis viral oncogene homolog (avian)	-
2478	7,01	7,63	-1,09	0,01669	0,367	234195_at	cDNA FLJ22426 fis, clone HRC08780	-
3283	4,04	4,40	-1,09	0,02339	0,388	1568913_at	NOL1/NOP2/Sun domain family, member 3	*NSUN3*
3128	3,35	3,64	-1,09	0,02218	0,387	1567078_x_at	-	-
1843	4,08	4,44	-1,09	0,01185	0,351	1555000_at	OK/SW-CL.36	-
4152	4,41	4,80	-1,09	0,03063	0,403	1556426_at	Hexosaminidase A (alpha polypeptide)	*HEXA*
5501	3,63	3,95	-1,09	0,04354	0,433	1564491_at	Chromosome X open reading frame 18	*CXorf18*
1759	3,18	3,46	-1,09	0,01122	0,348	236729_at	Caspase 3, apoptosis-related cysteine peptidase	*CASP3*
4598	4,37	4,75	-1,09	0,03477	0,412	235813_at	cDNA FLJ46701 fis, clone TRACH3014063	-
5902	7,87	8,56	-1,09	0,04783	0,443	238194_at	Transcribed locus	-
944	4,37	4,75	-1,09	0,00542	0,313	1562943_at	Clone IMAGE:5742085, mRNA	-
6081	5,83	6,34	-1,09	0,04965	0,446	219845_at	BARX homeobox 1	*BARX1*
4117	15,7	17,1	-1,09	0,03027	0,402	1556397_at	cDNA FLJ37122 fis, clone BRACE2022448	-
966	3,23	3,52	-1,09	0,00559	0,315	1562432_at	cDNA clone IMAGE:4794272	-
3189	3,76	4,09	-1,09	0,02260	0,387	217359_s_at	Neural cell adhesion molecule 1	*NCAM1*
3625	4,12	4,48	-1,09	0,02617	0,395	240790_at	Transcribed locus	-

Index	Mutated*	Normal*	FC	*P* value	FDR**	Probe set	Description	Gene symbol
3229	5,48	5,96	-1,09	0,02290	0,388	239929_at	Hypothetical protein FLJ32569	*FLJ32569*
6049	4,93	5,36	-1,09	0,04929	0,445	1553323_a_at	Cation channel, sperm associated 2	*CATSPER2*
5024	4,06	4,41	-1,09	0,03863	0,420	1570049_at	Clone IMAGE:5176619, mRNA	-
2998	3,70	4,02	-1,09	0,02111	0,385	1553241_at	-	-
5680	11,3	12,3	-1,09	0,04535	0,436	207784_at	Arylsulfatase D	*ARSD*
3647	20,2	21,9	-1,09	0,02635	0,395	1566502_at	cDNA DKFZp434B049	-
5468	5,10	5,55	-1,09	0,04320	0,432	240872_at	Transcribed locus	-
2406	4,88	5,31	-1,09	0,01608	0,365	1560509_at	cDNA DKFZp547H194	-
3824	8,66	9,41	-1,09	0,02792	0,399	234549_at	cDNA DKFZp586G1917	-
4921	11,7	12,7	-1,09	0,03770	0,419	1557342_a_at	Hypothetical protein LOC150381	*LOC150381*
4396	53,3	57,9	-1,09	0,03277	0,407	1556291_at	cDNA DKFZp564N2472	-
1853	3,05	3,32	-1,09	0,01194	0,352	229935_s_at	-	-
4661	41,4	45,0	-1,09	0,03519	0,413	91920_at	Brevican	*BCAN*
6053	5,31	5,77	-1,09	0,04937	0,446	1566476_at	-	-
5959	3,73	4,05	-1,09	0,04837	0,444	1564051_at	-	-
2278	4,01	4,36	-1,09	0,01515	0,363	230594_at	Transcribed locus	-
2516	3,13	3,40	-1,09	0,01695	0,368	1553299_at	Hypothetical protein FLJ31401	-
5858	4,06	4,41	-1,09	0,04737	0,442	216245_at	Interleukin 1 receptor antagonist	*IL1RN*
5407	3,47	3,77	-1,09	0,04244	0,429	244837_at	-	-
5757	3,80	4,13	-1,09	0,04622	0,439	1557446_x_at	Triggering receptor expressed on myeloid cells-like 3	*TREML3*
4494	3,60	3,91	-1,09	0,03380	0,411	241190_at	Transcribed locus	-
4538	3,66	3,98	-1,09	0,03429	0,412	236842_at	cDNA FLJ36110 fis, clone TESTI2021939	-
2386	3,43	3,73	-1,09	0,01591	0,364	1560309_s_at	Full length insert cDNA clone YP59A09	-
922	3,91	4,25	-1,09	0,00531	0,313	210384_at	Protein arginine methyltransferase 2	*PRMT2*
4597	12,8	13,9	-1,09	0,03477	0,412	206657_s_at	Myogenic differentiation 1	*MYOD1*
4150	5,21	5,66	-1,09	0,03062	0,403	227831_at	Chromosome 9 open reading frame 5	*C9orf5*
3554	4,06	4,41	-1,09	0,02562	0,394	231344_at	Transcribed locus	-
1919	3,67	3,99	-1,09	0,01251	0,355	233671_at	cDNA FLJ11179 fis, clone PLACE1007450	-
1551	3,59	3,90	-1,09	0,00956	0,337	211349_at	Solute carrier family 15, member 1	*SLC15A1*
3052	4,45	4,84	-1,09	0,02148	0,385	1569849_at	cDNA clone IMAGE:4838370	-
6005	6,85	7,44	-1,09	0,04880	0,444	234708_at	Single-strand-selective monofunctional uracil-DNA glycosylase 1	*SMUG1*
6028	4,36	4,74	-1,09	0,04903	0,445	238270_x_at	-	-
1672	3,89	4,23	-1,09	0,01055	0,345	1561143_at	Full length insert cDNA clone ZD46F01	-
5601	14,7	15,9	-1,09	0,04468	0,436	216439_at	Tyrosine kinase, non-receptor, 2	*TNK2*
4566	12,4	13,4	-1,09	0,03452	0,412	238201_at	Transcribed locus	-
3946	3,63	3,94	-1,09	0,02882	0,399	240796_at	Transcribed locus	-
2780	3,25	3,53	-1,09	0,01930	0,380	1560810_at	Clone IMAGE:5745158, mRNA	-
2614	4,17	4,53	-1,09	0,01775	0,371	1554030_at	Arylsulfatase B	*ARSB*
5699	3,45	3,75	-1,09	0,04564	0,438	237912_at	-	-
5899	10,8	11,7	-1,09	0,04776	0,443	206152_at	Centaurin, gamma 1	*CENTG1*
4589	4,15	4,50	-1,09	0,03471	0,412	232829_at	Olfactory receptor, family 52, subfamily K, member 3 pseudogene	*OR52K3P*
3492	3,93	4,27	-1,09	0,02510	0,392	239206_at	Complement component (3b/4b) receptor 1-like	*CR1L*
5565	4,41	4,78	-1,09	0,04430	0,435	217433_at	Transforming, acidic coiled-coil containing protein 1	*TACC1*
5450	5,71	6,20	-1,09	0,04299	0,431	1561218_s_at	Hypothetical protein LOC728099	*LOC728099*
3767	4,53	4,91	-1,09	0,02737	0,397	1566737_at	HCG2036596 protein	-
5958	3,91	4,24	-1,09	0,04835	0,444	1564202_at	cDNA FLJ38820 fis, clone LIVER2008473	-
5175	3,69	4,01	-1,09	0,03987	0,421	1560698_a_at	Hypothetical protein LOC283392	*LOC283392*
4276	4,14	4,50	-1,09	0,03178	0,406	1555603_at	B melanoma antigen	*BAGE*
5705	5,34	5,79	-1,09	0,04569	0,438	230969_at	LOC374973 protein	*LOC374973*
5912	4,89	5,31	-1,09	0,04794	0,443	234744_x_at	-	-
2931	6,31	6,85	-1,09	0,02055	0,383	214571_at	Fibroblast growth factor 3	*FGF3*
5113	6,33	6,88	-1,09	0,03937	0,421	211389_x_at	Killer cell immunoglobulin-like receptor 3DS1	*KIR3DS1*
5382	8,32	9,03	-1,09	0,04219	0,429	243670_at	Transcribed locus	-
4348	4,02	4,36	-1,09	0,03238	0,407	237721_s_at	Ankyrin repeat and SOCS box-containing 4	*ASB4*
1426	3,39	3,68	-1,09	0,00865	0,331	206285_at	Nephronophthisis 1 (juvenile)	*NPHP1*
3495	3,60	3,91	-1,09	0,02513	0,392	238073_at	-	-
2727	4,67	5,07	-1,09	0,01877	0,376	241037_at	-	-

Index	Mutated*	Normal*	FC	*P* value	FDR**	Probe set	Description	Gene symbol
3826	6,05	6,56	-1,09	0,02793	0,399	233781_s_at	RAP1 interacting factor homolog (yeast)	*RIF1*
4783	54,5	59,2	-1,09	0,03627	0,414	220962_s_at	Peptidyl arginine deiminase, type I	*PADI1*
4513	14,7	15,9	-1,09	0,03402	0,412	1559675_at	-	-
6030	12,5	13,6	-1,09	0,04906	0,445	1557022_at	cDNA clone IMAGE:5298883	-
5986	5,36	5,82	-1,09	0,04863	0,444	1553350_at	Family with sequence similarity 123C	*FAM123C*
2436	2,69	2,92	-1,09	0,01628	0,365	1568840_at	WD repeats and SOF1 domain containing	*WDSOF1*
3187	4,31	4,68	-1,09	0,02260	0,387	1558856_at	DMRT-like family A2	*DMRTA2*
5586	5,57	6,04	-1,08	0,04455	0,436	220515_at	Dual specificity phosphatase 21	*DUSP21*
461	3,00	3,26	-1,08	0,00245	0,288	1565840_at	cDNA FLJ35990 fis, clone TESTI2014415	-
5703	4,50	4,88	-1,08	0,04565	0,438	208275_x_at	Undifferentiated embryonic cell transcription factor 1	*UTF1*
5352	4,25	4,61	-1,08	0,04198	0,429	241007_at	Transcribed locus	-
1646	3,69	4,00	-1,08	0,01036	0,344	1566739_at	Phospholipase C, epsilon 1	*PLCE1*
3740	3,77	4,09	-1,08	0,02722	0,397	243727_at	Copine VIII	*CPNE8*
4194	5,85	6,35	-1,08	0,03098	0,404	230010_at	KIAA1853 protein	*KIAA1853*
5560	8,10	8,79	-1,08	0,04426	0,435	244012_x_at	-	-
6076	5,31	5,76	-1,08	0,04959	0,446	230574_at	-	-
2655	3,10	3,36	-1,08	0,01811	0,373	215663_at	Muscleblind-like (Drosophila)	*MBNL1*
1993	3,59	3,89	-1,08	0,01303	0,355	1560771_at	cDNA FLJ36254 fis, clone THYMU2002157	-
4792	5,02	5,45	-1,08	0,03633	0,415	1552991_at	Olfactory receptor, family 5, subfamily P, member 2	*OR5P2*
5277	4,54	4,93	-1,08	0,04102	0,425	206281_at	Adenylate cyclase activating polypeptide 1 (pituitary)	*ADCYAP1*
1249	3,26	3,54	-1,08	0,00741	0,325	211341_at	POU class 4 homeobox 1	*POU4F1*
5584	4,36	4,72	-1,08	0,04452	0,436	224181_at	Chromosome 18 open reading frame 2	*C18orf2*
4190	3,60	3,90	-1,08	0,03096	0,404	1555353_at	Low density lipoprotein-related protein 1	*LRP1*
4288	4,10	4,44	-1,08	0,03185	0,406	216217_at	Phospholipase C-like 2	*PLCL2*
5393	4,01	4,35	-1,08	0,04229	0,429	1556456_at	Hypothetical FLJ39739	*FLJ39739*
5927	6,12	6,63	-1,08	0,04803	0,443	238216_at	Clone IMAGE:4340526, mRNA	-
3743	4,40	4,77	-1,08	0,02723	0,397	221271_at	Interleukin 21	*IL21*
3623	3,23	3,51	-1,08	0,02616	0,395	217470_at	cDNA DKFZp434F1011	-
4612	17,0	18,4	-1,08	0,03483	0,412	216371_at	-	-
3153	3,28	3,55	-1,08	0,02237	0,387	239192_at	Par-3 partitioning defective 3 homolog B (C. elegans)	*PARD3B*
5591	12,1	13,1	-1,08	0,04458	0,436	1553865_a_at	cDNA DKFZp761H2121	-
2751	3,75	4,07	-1,08	0,01903	0,378	1562923_at	cDNA clone IMAGE:4838328	-
827	4,41	4,78	-1,08	0,00472	0,312	1562974_at	cDNA clone IMAGE:5302821	-
1083	2,81	3,05	-1,08	0,00634	0,319	1563392_at	Chromosome 21, Down syndrome critical region transcript	-
5009	10,8	11,7	-1,08	0,03848	0,420	217341_at	-	-
2507	3,51	3,81	-1,08	0,01685	0,367	231483_at	Similar to XP_001149380.1	-
3611	31,4	34,1	-1,08	0,02606	0,394	1556718_s_at	cDNA FLJ35483 fis, clone SMINT2008277	-
5603	4,00	4,33	-1,08	0,04471	0,436	1556490_a_at	Hypothetical LOC388946	*LOC388946*
394	2,89	3,13	-1,08	0,00208	0,285	1570486_at	SRY (sex determining region Y)-box 6	*SOX6*
1203	7,40	8,02	-1,08	0,00712	0,323	1553863_at	Chromosome 10 open reading frame 64	*C10orf64*
3871	3,99	4,32	-1,08	0,02828	0,399	1559065_a_at	C-type lectin superfamily 4, member G	*CLEC4G*
4738	5,52	5,98	-1,08	0,03586	0,414	1565583_at	Zinc finger and SCAN domain containing 5	*ZSCAN5*
4594	4,66	5,05	-1,08	0,03476	0,412	1564444_at	cDNA FLJ40178 fis, clone TESTI2017932	-
5350	4,62	5,01	-1,08	0,04196	0,429	1555048_a_at	Chromosome 21 open reading frame 29	*C21orf29*
4293	7,76	8,41	-1,08	0,03192	0,406	1566535_at	cDNA FLJ40701 fis, clone THYMU2025909	-
4725	4,58	4,96	-1,08	0,03576	0,414	1555085_at	-	-
986	3,60	3,90	-1,08	0,00569	0,315	1555116_s_at	Solute carrier family 11, member 1	*SLC11A1*
5901	8,98	9,73	-1,08	0,04780	0,443	1560409_at	Clone IMAGE:5211852, mRNA	-
2460	3,98	4,32	-1,08	0,01652	0,367	1565801_at	cDNA clone IMAGE:4796818	-
3957	3,84	4,16	-1,08	0,02890	0,399	1561099_at	Hypothetical LOC642757	*FLJ32756*
4850	4,33	4,69	-1,08	0,03695	0,417	217507_at	Solute carrier family 11, member 1	*SLC11A1*
5655	5,54	6,00	-1,08	0,04510	0,436	235367_at	Myopalladin	*MYPN*
3469	13,4	14,5	-1,08	0,02485	0,392	1564431_a_at	FLJ45224 protein	*FLJ45224*
872	3,18	3,44	-1,08	0,00504	0,313	207477_at	-	-
5822	27,6	29,9	-1,08	0,04704	0,442	1569679_at	Cadhetrin-like 22	*CDH22*
3107	6,35	6,88	-1,08	0,02193	0,386	237203_at	Clone IMAGE:4214313, mRNA	-
5048	3,98	4,31	-1,08	0,03889	0,420	241679_at	-	-

Index	Mutated*	Normal*	FC	*P* value	FDR**	Probe set	Description	Gene symbol
2827	4,86	5,27	-1,08	0,01973	0,381	1556502_at	cDNA clone IMAGE:4825100	-
3963	5,60	6,07	-1,08	0,02895	0,399	216790_at	cDNA FLJ22812 fis, clone KAIA2955	-
5162	4,99	5,41	-1,08	0,03978	0,421	207665_at	ADAM metallopeptidase domain 21	*ADAM21*
4502	4,86	5,27	-1,08	0,03388	0,411	1560832_at	Clone IMAGE:4063532, mRNA	-
3196	9,86	10,7	-1,08	0,02265	0,387	220996_s_at	Chromosome 1 open reading frame 14	*C1orf14*
2903	2,96	3,21	-1,08	0,02041	0,383	234454_at	-	-
4618	3,94	4,26	-1,08	0,03485	0,412	1556200_a_at	Chromosome 10 open reading frame 90	*C10orf90*
4328	17,8	19,3	-1,08	0,03221	0,407	1558705_at	Atonal homolog 8 (Drosophila)	*ATOH8*
4397	4,77	5,17	-1,08	0,03278	0,407	233606_at	cDNA FLJ11424 fis, clone HEMBA1001026	-
5719	7,79	8,44	-1,08	0,04580	0,438	1566602_at	RNA pseudouridylate synthase domain containing 3	*RPUSD3*
672	2,63	2,85	-1,08	0,00386	0,308	231613_at	MAP/microtubule affinity-regulating kinase 1	*MARK1*
6031	3,72	4,03	-1,08	0,04909	0,445	230631_s_at	Full-length cDNA clone CS0DI051YA02	-
2928	3,47	3,76	-1,08	0,02053	0,383	220984_s_at	Solute carrier organic anion transporter family, member 5A1	*SLCO5A1*
3634	3,48	3,77	-1,08	0,02628	0,395	1566339_at	Small nucleolar RNA, C/D box 8	*SNORD8*
2857	4,90	5,30	-1,08	0,02006	0,383	207924_x_at	Paired box 8	*PAX8*
1920	6,80	7,37	-1,08	0,01251	0,355	1569887_a_at	Hypothetical protein LOC286135	*LOC286135*
1469	3,30	3,58	-1,08	0,00898	0,334	217380_s_at	Clone DT1P1A11 Mrna	-
1923	3,86	4,18	-1,08	0,01252	0,355	1559559_at	Chromosome 9 open reading frame 79	*C9orf79*
5513	5,14	5,56	-1,08	0,04367	0,433	207503_at	T-complex 10 (mouse)	*TCP10*
5155	4,31	4,67	-1,08	0,03969	0,421	234357_at	cDNA FLJ13578 fis, clone PLACE1008757	-
1628	3,24	3,51	-1,08	0,01025	0,344	244602_at	Transcribed locus	-
2679	3,62	3,92	-1,08	0,01833	0,374	1564337_at	cDNA FLJ35701 fis, clone SPLEN2020007	-
3339	6,59	7,13	-1,08	0,02371	0,388	243095_at	Transcribed locus	-
2421	5,87	6,35	-1,08	0,01618	0,365	1555545_at	Kallikrein-related peptidase 2	*KLK2*
5294	6,23	6,74	-1,08	0,04123	0,426	229621_x_at	Early B-cell factor 3	*EBF3*
2329	8,39	9,08	-1,08	0,01551	0,364	1553837_at	Phosphoglycerate mutase family member 5	*PGAM5*
4646	10,3	11,1	-1,08	0,03505	0,412	217637_at	Full length insert cDNA clone ZE05A03	-
1974	3,70	4,00	-1,08	0,01291	0,355	231105_at	Similar to XP_001132401.1	-
2699	3,50	3,78	-1,08	0,01851	0,375	244138_at	Hypothetical protein LOC728904	*LOC728904*
4635	8,21	8,89	-1,08	0,03496	0,412	229239_x_at	Solute carrier organic anion transporter family, member 4A1	*SLCO4A1*
5662	2,88	3,12	-1,08	0,04519	0,436	217443_at	cDNA DKFZp434F104	-
5346	5,77	6,24	-1,08	0,04191	0,429	241476_at	Similar to XP_001130933.1	-
2343	5,28	5,72	-1,08	0,01564	0,364	222965_at	Hypothetical protein PRO2214	*PRO2214*
4387	3,16	3,42	-1,08	0,03268	0,407	214382_at	Unc-93 homolog A (C. elegans)	*UNC93A*
4912	4,13	4,47	-1,08	0,03757	0,418	1556960_a_at	HCG2007354 protein	-
2835	9,22	9,98	-1,08	0,01979	0,382	1554065_at	Family with sequence similarity 125, member B	*FAM125B*
5918	6,39	6,91	-1,08	0,04797	0,443	216059_at	Paired box 3	*PAX3*
4630	4,45	4,81	-1,08	0,03491	0,412	208247_at	Chromosome 3 open reading frame 51	*C3orf51*
2967	3,79	4,10	-1,08	0,02085	0,384	206446_s_at	Elastase 2A	*ELA2A*
5556	4,01	4,34	-1,08	0,04423	0,435	216223_at	Carboxypeptidase N, polypeptide 2	*CPN2*
3521	8,25	8,93	-1,08	0,02527	0,392	207500_at	Caspase 5, apoptosis-related cysteine peptidase	*CASP5*
4544	6,39	6,91	-1,08	0,03434	0,412	214242_at	Mannosidase, alpha, class 1A, member 2	*MAN1A2*
5351	3,57	3,86	-1,08	0,04198	0,429	215249_at	Ribosomal protein L35a	*RPL35A*
5463	8,66	9,37	-1,08	0,04314	0,432	240500_at	-	-
5295	4,59	4,96	-1,08	0,04124	0,426	1567358_at	Neuron navigator 2	*NAV2*
3650	5,95	6,43	-1,08	0,02638	0,395	240716_at	Tetratricopeptide repeat domain 23	*TTC23*
2532	4,48	4,85	-1,08	0,01711	0,369	1566181_at	cDNA FLJ20881 fis, clone ADKA03177	-
5614	9,43	10,2	-1,08	0,04478	0,436	1553688_at	Methyltransferase like 6	*METTL6*
2981	5,18	5,60	-1,08	0,02096	0,384	1555237_at	cDNA clone IMAGE:4336144	-
4140	3,58	3,87	-1,08	0,03056	0,403	224136_at	Mitogen-activated protein kinase 1	*p41*
4178	3,64	3,93	-1,08	0,03089	0,404	237767_at	FERM, RhoGEF (ARHGEF) and pleckstrin domain protein 1	*FARP1*
5869	5,93	6,41	-1,08	0,04743	0,442	243553_x_at	Transcribed locus	-
5866	3,92	4,24	-1,08	0,04742	0,442	216946_at	Major histocompatibility complex, class II, DO alpha	*HLA-DOA*
2797	2,92	3,15	-1,08	0,01943	0,380	1557739_at	cDNA clone IMAGE:5298947	-
5458	5,16	5,58	-1,08	0,04312	0,432	1559049_a_at	BAH domain and coiled-coil containing 1	*BAHCC1*
5718	4,40	4,76	-1,08	0,04580	0,438	232232_s_at	Solute carrier family 22, member 16	*SLC22A16*
3456	3,20	3,45	-1,08	0,02475	0,392	215958_at	cDNA FLJ12003 fis, clone HEMBB1001537	-

Index	Mutated*	Normal*	FC	*P* value	FDR**	Probe set	Description	Gene symbol
5881	13,6	14,7	-1,08	0,04758	0,442	1553163_at	ADP-ribosylhydrolase like 1	*ADPRHL1*
4376	3,45	3,73	-1,08	0,03256	0,407	1561485_at	IQ motif containing with AAA domain	*IQCA*
5795	4,59	4,96	-1,08	0,04675	0,441	233738_at	cDNA DKFZp566E1346	-
4883	3,33	3,59	-1,08	0,03734	0,418	1567076_at	-	-
5874	4,51	4,88	-1,08	0,04745	0,442	1569750_at	cDNA clone IMAGE:4822123	-
4002	3,32	3,58	-1,08	0,02930	0,400	1570101_at	cDNA clone IMAGE:5269204	-
2527	2,71	2,93	-1,08	0,01707	0,369	207901_at	Interleukin 12B	*IL12B*
707	3,23	3,49	-1,08	0,00406	0,308	1569064_at	Hypothetical LOC643338	*LOC643338*
3136	3,53	3,82	-1,08	0,02223	0,387	1552929_at	G protein-coupled receptor kinase 7	*GRK7*
4581	3,23	3,49	-1,08	0,03461	0,412	244446_at	cDNA clone IMAGE:5266114	-
1764	2,79	3,01	-1,08	0,01124	0,348	222966_at	-	-
5736	3,67	3,97	-1,08	0,04601	0,438	1560262_at	Clone IMAGE:5751523, mRNA	-
2264	3,55	3,83	-1,08	0,01505	0,363	240431_at	-	-
5323	5,29	5,71	-1,08	0,04162	0,427	243094_at	Chromosome 13 open reading frame 21	*C13orf21*
3410	3,95	4,27	-1,08	0,02433	0,390	240477_at	Transcribed locus	-
995	3,01	3,25	-1,08	0,00573	0,315	216313_at	Protocadherin beta 17 pseudogene	*PCDHB17*
4746	2,97	3,20	-1,08	0,03595	0,414	1563524_a_at	Chromosome 14 open reading frame 85	*C14orf85*
4472	5,73	6,19	-1,08	0,03360	0,411	217005_at	Low density lipoprotein receptor	*LDLR*
4464	4,70	5,07	-1,08	0,03354	0,411	1560455_at	Hypothetical protein LOC339166	*LOC339166*
1791	3,48	3,76	-1,08	0,01147	0,350	1567270_at	-	-
6058	3,65	3,94	-1,08	0,04941	0,446	220115_s_at	Cadhetrin 10, type 2 (T2-Cadhetrin)	*CDH10*
3503	3,74	4,04	-1,08	0,02517	0,392	202493_x_at	Chorionic somatomammotropin hormone 1	*CSH1*
5485	5,28	5,70	-1,08	0,04341	0,433	232526_at	Inositol 1,4,5-trisphosphate 3-kinase B	*ITPKB*
5356	3,56	3,85	-1,08	0,04201	0,429	1566861_at	Hypothetical protein LOC145663	*LOC145663*
4765	12,8	13,8	-1,08	0,03611	0,414	1554752_a_at	cDNA clone IMAGE:3528357	-
5616	6,13	6,62	-1,08	0,04482	0,436	241515_at	-	-
4886	3,48	3,75	-1,08	0,03737	0,418	1566693_at	cDNA FLJ20947 fis, clone ADSE01841	-
5433	4,83	5,22	-1,08	0,04281	0,431	1555624_a_at	Slingshot homolog 1 (Drosophila)	*SSH1*
4638	2,89	3,12	-1,08	0,03498	0,412	231781_s_at	Leucine rich repeat containing 2	*LRRC2*
3844	3,09	3,34	-1,08	0,02806	0,399	1553120_at	Claspin homolog (Xenopus laevis)	*CLSPN*
5762	4,44	4,79	-1,08	0,04629	0,439	1559561_at	F-box protein, helicase, 18	*FBXO18*
4786	4,34	4,69	-1,08	0,03629	0,415	1553844_a_at	Chromosome 10 open reading frame 67	*C10orf67*
5955	3,49	3,77	-1,08	0,04831	0,444	211617_at	Aldolase A, fructose-bisphosphate pseudogene 2	*ALDOAP2*
1934	3,29	3,55	-1,08	0,01261	0,355	220854_at	-	-
6006	12,9	14,0	-1,08	0,04881	0,444	217557_s_at	Carboxypeptidase M	*CPM*
6111	3,28	3,54	-1,08	0,04987	0,446	1556081_at	cDNA FLJ33441 fis, clone BRACE2021932	-
3618	4,73	5,10	-1,08	0,02612	0,395	1559777_at	cDNA FLJ32866 fis, clone TESTI2003718	-
1995	3,22	3,47	-1,08	0,01304	0,355	241238_at	Transcribed locus	-
3576	3,07	3,31	-1,08	0,02582	0,394	1561009_at	Monoamine oxidase B	*MAOB*
5385	3,99	4,30	-1,08	0,04224	0,429	1568864_at	Hypothetical protein MGC2752	*MGC2752*
5825	5,77	6,22	-1,08	0,04706	0,442	233745_at	cDNA FLJ34894 fis, clone NT2NE2017982	-
4643	3,98	4,29	-1,08	0,03501	0,412	217632_at	Guanine nucleotide binding protein-like 3 (nucleolar)-like	*GNL3L*
3581	12,5	13,5	-1,08	0,02586	0,394	1553917_at	Phosphatidylinositol-3-phosphate 5-kinase type III	*PIP5K3*
2228	5,90	6,37	-1,08	0,01480	0,363	1563488_at	Transmembrane protein 132B	*TMEM132B*
1752	2,83	3,05	-1,08	0,01117	0,348	1566266_at	Full length insert cDNA YQ80D07	-
3344	3,19	3,44	-1,08	0,02375	0,388	207870_at	A kinase (PRKA) anchor protein (yotiao) 9	*AKAP9*
5078	7,12	7,68	-1,08	0,03906	0,420	1561702_at	cDNA clone IMAGE:5268043	-
5978	3,94	4,25	-1,08	0,04854	0,444	222298_at	cDNA FLJ41644 fis, clone FEBRA2009362	-
5420	4,40	4,75	-1,08	0,04261	0,430	1563612_at	-	-
5764	4,38	4,72	-1,08	0,04631	0,439	222177_s_at	SCAN domain containing 2	*SCAND2*
5486	4,86	5,24	-1,08	0,04341	0,433	207615_s_at	Chromosome 16 open reading frame 3	*C16orf3*
2951	3,15	3,39	-1,08	0,02072	0,384	235622_at	Transcribed locus	-
1085	3,29	3,54	-1,08	0,00636	0,319	239460_at	Cytochrome P450, family 19, subfamily A, polypeptide 1	*CYP19A1*
5672	4,42	4,76	-1,08	0,04527	0,436	1562691_at	Clone IMAGE:5723690, mRNA	-
5165	28,6	30,9	-1,08	0,03980	0,421	204292_x_at	Serine/threonine kinase 11	*STK11*
5218	4,07	4,39	-1,08	0,04034	0,423	214648_at	Glutathione peroxidase 5	*GPX5*
5808	3,88	4,18	-1,08	0,04689	0,441	1561455_at	Hypothetical protein LOC284294	*LOC284294*

Index	Mutated*	Normal*	FC	*P* value	FDR**	Probe set	Description	Gene symbol
5298	7,28	7,84	-1,08	0,04127	0,426	240584_at	-	-
1797	4,05	4,37	-1,08	0,01156	0,351	1553446_at	Hypothetical protein FLJ37396	*FLJ37396*
4910	4,74	5,11	-1,08	0,03755	0,418	1563143_at	Clone IMAGE:5172510, mRNA	-
4113	3,26	3,52	-1,08	0,03023	0,402	1559970_at	Hypothetical protein LOC730034	*LOC730034*
5062	4,08	4,40	-1,08	0,03896	0,420	1564595_at	Clone IMAGE:5745181, mRNA	-
5152	5,68	6,12	-1,08	0,03965	0,421	235403_at	Transcribed locus	-
3090	7,43	8,01	-1,08	0,02179	0,385	211827_s_at	Potassium voltage-gated channel subfamily D member 3	*KCND3*
4165	4,03	4,34	-1,08	0,03078	0,404	219841_at	Activation-induced cytidine deaminase	*AICDA*
5790	3,83	4,12	-1,08	0,04667	0,441	215830_at	SH3 and multiple ankyrin repeat domains 2	*SHANK2*
1890	4,05	4,37	-1,08	0,01225	0,355	215872_at	-	-
5154	5,27	5,67	-1,08	0,03967	0,421	205753_at	C-reactive protein, pentraxin-related	*CRP*
3700	10,5	11,3	-1,08	0,02676	0,395	1563641_a_at	Selectin ligand interactor cytoplasmic-1	*SLIC1*
4537	3,63	3,91	-1,08	0,03429	0,412	233581_at	Clone 24850 mRNA sequence	-
2980	3,02	3,25	-1,08	0,02096	0,384	240122_at	DIRAS family, GTP-binding RAS-like 2	*DIRAS2*
4761	3,24	3,49	-1,08	0,03609	0,414	206810_at	Zinc finger protein 44	*ZNF44*
4323	6,31	6,80	-1,08	0,03218	0,407	1557981_at	Ribosomal protein S9	*RPS9*
655	3,40	3,66	-1,08	0,00377	0,308	241421_at	Transcribed locus	-
3139	3,22	3,46	-1,08	0,02226	0,387	1562750_at	cDNA clone IMAGE:4751528	-
5780	15,5	16,7	-1,08	0,04653	0,440	229832_x_at	SH3 domain and tetratricopeptide repeats 1	*SH3TC1*
2643	3,73	4,01	-1,08	0,01802	0,373	1557314_at	cDNA clone IMAGE:5303069	-
5888	5,26	5,66	-1,08	0,04763	0,442	208000_at	GPI anchored molecule like protein	*GML*
5265	6,62	7,13	-1,08	0,04089	0,425	1556369_a_at	Phosphorylase kinase, gamma 2 (testis)	*PHKG2*
4779	3,21	3,46	-1,08	0,03623	0,414	217083_at	Mitogen-activated protein kinase-activated protein kinase 5	*MAPKAPK5*
4017	10,6	11,4	-1,08	0,02942	0,400	214818_at	Coiled-coil domain containing 57	*CCDC57*
2325	4,11	4,42	-1,08	0,01547	0,364	237949_at	Transcribed locus	-
4168	4,33	4,67	-1,08	0,03080	0,404	1560750_at	Hypothetical protein LOC151121	*LOC151121*
3571	2,62	2,82	-1,08	0,02580	0,394	1552522_at	Tigger transposable element derived 4	*TIGD4*
3730	3,07	3,31	-1,08	0,02708	0,397	1554887_at	-	-
5739	8,88	9,56	-1,08	0,04603	0,439	1562411_at	MLCK protein	*MLCK*
5430	12,1	13,1	-1,08	0,04274	0,430	216337_at	(clone Z146) retinal mRNA, 3' end and repeat region	-
4330	4,40	4,73	-1,08	0,03224	0,407	1558177_at	Chromosome 14 open reading frame 83	*C14orf83*
5968	10,5	11,3	-1,08	0,04847	0,444	1569496_s_at	Spondin 2, extracellular matrix protein	*SPON2*
5987	4,41	4,75	-1,08	0,04865	0,444	1562687_x_at	cDNA FLJ20158 fis, clone COL08935	-
2259	3,13	3,37	-1,08	0,01504	0,363	207672_at	-	-
5142	5,29	5,69	-1,08	0,03959	0,421	1556477_a_at	Hypothetical protein LOC283485	*LOC283485*
1988	2,92	3,14	-1,08	0,01301	0,355	1553329_at	Chromosome 7 open reading frame 45	*C7orf45*
2168	3,27	3,52	-1,08	0,01434	0,361	234179_at	cDNA FLJ23200 fis, clone KAIA38871	-
3734	3,21	3,45	-1,08	0,02712	0,397	240581_at	Hypothetical LOC644135	*LOC644135*
5594	5,55	5,97	-1,08	0,04461	0,436	1554322_a_at	Histone deacetylase 4	*HDAC4*
5200	3,59	3,86	-1,08	0,04011	0,422	238713_at	Transcribed locus	-
2662	3,91	4,21	-1,08	0,01818	0,373	1560590_s_at	Clone IMAGE:2905626, mRNA	-
5343	8,37	9,00	-1,08	0,04188	0,429	1553890_s_at	Hypothetical protein BC018697	*LOC126147*
2648	3,83	4,12	-1,08	0,01804	0,373	1562541_at	Hypothetical protein LOC285928	*LOC285928*
4903	2,96	3,18	-1,08	0,03750	0,418	1569407_at	cDNA clone IMAGE:4825059	-
3082	3,48	3,74	-1,08	0,02172	0,385	1559661_at	Odz, odd Oz/ten-m homolog 3 (Drosophila)	*ODZ3*
4926	4,14	4,45	-1,08	0,03779	0,419	1557404_at	cDNA clone IMAGE:5266464	-
5674	3,25	3,50	-1,08	0,04528	0,436	1563376_at	Clone IMAGE:4469683, mRNA	-
3234	3,44	3,70	-1,08	0,02293	0,388	1570394_at	5'-3' exoribonuclease 1	*XRN1*
5936	5,37	5,77	-1,08	0,04814	0,443	233125_at	cDNA FLJ11686 fis, clone HEMBA1004944	-
4747	2,92	3,14	-1,08	0,03596	0,414	1554781_at	Hypothetical protein MGC32805	*MGC32805*
4501	2,98	3,20	-1,08	0,03388	0,411	1552394_a_at	ENTH domain containing 1	*ENTHD1*
778	3,23	3,48	-1,07	0,00446	0,311	1563085_at	-	-
5657	2,80	3,02	-1,07	0,04512	0,436	241986_at	BMP binding endothelial regulator	*BMPER*
3071	3,42	3,68	-1,07	0,02166	0,385	1552453_a_at	WD repeat domain 88	*WDR88*
4948	4,60	4,95	-1,07	0,03797	0,419	1557697_at	cDNA clone IMAGE:5311511	-
4966	2,95	3,17	-1,07	0,03810	0,419	241461_at	Transcribed locus	-
1445	4,20	4,51	-1,07	0,00879	0,332	243066_at	N-acetylneuraminate pyruvate lyase	*NPL*

Index	Mutated*	Normal*	FC	*P* value	FDR**	Probe set	Description	Gene symbol
5464	2,59	2,78	-1,07	0,04316	0,432	1554921_a_at	Sciellin	*SCEL*
5884	3,96	4,26	-1,07	0,04761	0,442	221439_at	Retinoblastoma binding protein 9	*RBBP9*
4890	9,19	9,87	-1,07	0,03738	0,418	1561371_at	Hypothetical gene supported by AK096399	*FLJ39080*
5302	2,84	3,05	-1,07	0,04138	0,426	1553497_at	Chromosome 12 open reading frame 37	*C12orf37*
3994	4,53	4,87	-1,07	0,02921	0,400	1565858_at	Small nucleolar RNA, H/ACA box 71A	*SNORA71A*
4169	3,72	4,00	-1,07	0,03081	0,404	1569257_at	Formin-like 1	*FMNL1*
4142	3,35	3,60	-1,07	0,03056	0,403	1562642_at	cDNA clone IMAGE:5288897	-
3137	3,78	4,06	-1,07	0,02225	0,387	1555430_at	Chromosome 9 open reading frame 97	*C9orf97*
3835	4,31	4,63	-1,07	0,02802	0,399	234933_at	KIAA1345 protein	*KIAA1345*
3248	2,93	3,15	-1,07	0,02307	0,388	211239_s_at	ADAM metallopeptidase domain 7	*ADAM7*
1714	2,73	2,93	-1,07	0,01099	0,348	240251_at	Intraflagellar transport 80 homolog (Chlamydomonas)	*IFT80*
5248	3,23	3,47	-1,07	0,04064	0,423	224126_at	Solute carrier family 10, member 7	*SLC10A7*
4432	3,39	3,64	-1,07	0,03320	0,409	1554965_at	Filamin A interacting protein 1-like	*FILIP1L*
5059	5,43	5,83	-1,07	0,03895	0,420	215366_at	Sorting nexin 13	*SNX13*
4299	8,50	9,13	-1,07	0,03201	0,407	36742_at	Tripartite motif-containing 15	*TRIM15*
4261	4,13	4,44	-1,07	0,03168	0,406	1563822_at	cDNA FLJ37602 fis, clone BRCOC2009380	-
4794	3,32	3,57	-1,07	0,03635	0,415	207544_s_at	Alcohol dehydrogenase 6 (class V)	*ADH6*
3190	3,01	3,23	-1,07	0,02262	0,387	236074_at	cDNA FLJ31443 fis, clone NT2NE2000808	-
5873	7,33	7,87	-1,07	0,04745	0,442	1554591_at	Gene differentially expressed in prostate	*GDEP*
4917	45,1	48,5	-1,07	0,03763	0,418	233307_x_at	cDNA FLJ20066 fis, clone COL01632	-
5518	3,92	4,21	-1,07	0,04378	0,433	220517_at	Vacuolar protein sorting 13 homolog C (S. cerevisiae)	*VPS13C*
5507	7,57	8,13	-1,07	0,04362	0,433	234557_at	Similar to COMM domain containing 4	*LOC440292*
2249	2,85	3,06	-1,07	0,01494	0,363	1554871_at	-	-
5903	3,14	3,38	-1,07	0,04783	0,443	207033_at	Gastric intrinsic factor (vitamin B synthesis)	*GIF*
5738	6,71	7,20	-1,07	0,04601	0,438	236726_at	cDNA FLJ43552 fis, clone PROST2017972	-
3995	3,21	3,45	-1,07	0,02921	0,400	224289_s_at	FKSG83	*FKSG83*
1569	3,27	3,51	-1,07	0,00975	0,340	234622_at	cDNA FLJ21514 fis, clone COL05780	-
5325	4,46	4,79	-1,07	0,04162	0,427	210348_at	Septin 4	*SEPT4*
4068	3,00	3,22	-1,07	0,02983	0,401	241678_at	Transcribed locus	-
2888	3,01	3,23	-1,07	0,02025	0,383	220697_at	cDNA clone IMAGE:40003562	-
5895	5,14	5,52	-1,07	0,04768	0,442	228344_s_at	T-box 3 (ulnar mammary syndrome)	*TBX3*
5099	13,3	14,3	-1,07	0,03922	0,420	230562_at	cDNA FLJ25042 fis, clone CBL03351	-
1382	2,83	3,04	-1,07	0,00832	0,329	1564628_at	cDNA DKFZp564A136	-
5013	3,69	3,96	-1,07	0,03855	0,420	1553785_at	RasGEF domain family, member 1B	*RASGEF1B*
3504	2,71	2,90	-1,07	0,02517	0,392	217400_at	Similar to Proliferating cell nuclear antigen (PCNA) (Cyclin)	*LOC392454*
3564	2,93	3,15	-1,07	0,02572	0,394	1553134_s_at	Chromosome 9 open reading frame 72	*C9orf72*
2576	4,29	4,60	-1,07	0,01748	0,371	1560212_a_at	Transcribed locus	-
3489	4,37	4,68	-1,07	0,02509	0,392	211369_at	PRO2168	-
5723	6,60	7,07	-1,07	0,04586	0,438	1569516_at	Clone IMAGE:5585187, mRNA	-
5640	3,14	3,37	-1,07	0,04500	0,436	232999_at	cDNA DKFZp564M1563	-
807	3,56	3,82	-1,07	0,00460	0,311	231315_at	Transcribed locus	-
4897	2,93	3,14	-1,07	0,03744	0,418	239479_x_at	Transcribed locus	-
3341	3,35	3,59	-1,07	0,02372	0,388	233778_at	Uncharacterized gastric protein ZG16P	
5617	3,92	4,21	-1,07	0,04483	0,436	1557767_at	Nanos homolog 2 (Drosophila)	*NANOS2*
4016	3,31	3,55	-1,07	0,02941	0,400	241620_at	Structural maintenance of chromosomes flexible hinge domain containing 1	*SMCHD1*
4452	5,69	6,10	-1,07	0,03344	0,411	237811_at	Transcribed locus	-
1927	2,76	2,96	-1,07	0,01255	0,355	237978_at	Transcribed locus	-
4297	3,65	3,91	-1,07	0,03195	0,407	243427_at	Transcribed locus	-
1507	2,96	3,18	-1,07	0,00927	0,336	1562520_at	cDNA FLJ37868 fis, clone BRSSN2017297	-
5575	4,11	4,41	-1,07	0,04442	0,436	229406_at	Hypothetical protein LOC146713	*HRNBP3*
5860	4,52	4,85	-1,07	0,04738	0,442	1554842_at	Solute carrier family 12, member 1	*SLC12A1*
2444	3,12	3,34	-1,07	0,01635	0,366	1552715_a_at	Relaxin/insulin-like family peptide receptor 1	*RXFP1*
3663	3,97	4,26	-1,07	0,02648	0,395	1555671_at	Islet cell autoantigen 1,69kDa-like	*ICA1L*
1994	2,75	2,95	-1,07	0,01303	0,355	237987_x_at	-	-
4757	3,88	4,16	-1,07	0,03607	0,414	1554716_s_at	Chromosome 15 open reading frame 50	*C15orf50*
5109	5,58	5,98	-1,07	0,03930	0,421	1564559_at	Hypothetical protein LOC728073	*LOC728073*
4749	3,79	4,06	-1,07	0,03599	0,414	1567139_at	TTY2 gene, clone TTY2L12A	

Index	Mutated*	Normal*	FC	*P* value	FDR**	Probe set	Description	Gene symbol
1877	2,75	2,95	-1,07	0,01214	0,354	1563754_at	Glutamate receptor, ionotropic, kainate 2	*GRIK2*
5684	4,07	4,36	-1,07	0,04538	0,436	239820_at	Ring finger protein 7	*RNF7*
5727	7,30	7,82	-1,07	0,04592	0,438	1570068_at	Clone IMAGE:4294479, mRNA	-
4018	3,25	3,48	-1,07	0,02942	0,400	242047_at	Pleckstrin homology domain containing, family K member 1	*PLEKHK1*
5400	13,2	14,2	-1,07	0,04235	0,429	207488_at	-	-
2748	3,27	3,50	-1,07	0,01900	0,378	209990_s_at	Gamma-aminobutyric acid (GABA) B receptor, 2	*GABBR2*
4069	3,41	3,65	-1,07	0,02984	0,401	216477_at	-	-
1409	3,65	3,91	-1,07	0,00851	0,330	235251_at	cDNA FLJ42786 fis, clone BRAWH3006761	-
5254	3,03	3,25	-1,07	0,04072	0,424	1562850_at	cDNA clone IMAGE:5742601	-
2778	13,7	14,7	-1,07	0,01930	0,380	1563425_at	Clone IMAGE:429302, mRNA sequence	-
2073	3,08	3,30	-1,07	0,01355	0,356	1555345_at	Solute carrier family 38, member 4	*SLC38A4*
5047	3,49	3,73	-1,07	0,03889	0,420	1566219_at	Hypothetical protein LOC338651	*LOC338651*
4294	3,63	3,88	-1,07	0,03193	0,406	1570234_at	Clone IMAGE:5169759, mRNA	-
5300	3,14	3,36	-1,07	0,04131	0,426	240073_at	-	-
2603	10,7	11,5	-1,07	0,01767	0,371	1569300_at	Chromosome 1 open reading frame 148	*C1orf148*
2401	3,53	3,78	-1,07	0,01601	0,365	1553472_at	Hypothetical protein FLJ32955	*FLJ32955*
3950	4,90	5,25	-1,07	0,02884	0,399	217439_at	-	-
5022	5,54	5,93	-1,07	0,03862	0,420	243674_at	-	-
5245	4,81	5,14	-1,07	0,04061	0,423	233556_at	cDNA clone IMAGE:5274560	-
5319	7,29	7,80	-1,07	0,04151	0,427	240567_at	cDNA clone IMAGE:4096591	-
2926	3,91	4,19	-1,07	0,02051	0,383	1555135_at	-	-
**4453**	3,78	4,04	-1,07	0,03344	0,411	215964_at	EST clone 22453 mariner transposon Hsmar1 sequence	-
5944	2,99	3,20	-1,07	0,04817	0,443	233050_at	Chromosome 6 open reading frame 174	*C6orf174*
3575	3,58	3,83	-1,07	0,02581	0,394	203820_s_at	Insulin-like growth factor 2 mRNA binding protein 3	*IGF2BP3*
4904	4,18	4,47	-1,07	0,03751	0,418	1564084_at	CAD trifunctional protein	*CAD*
3661	3,48	3,73	-1,07	0,02647	0,395	235818_at	v-Set and transmembrane domain containing 1	*VSTM1*
4175	4,13	4,42	-1,07	0,03086	0,404	1565882_at	cDNA FLJ12064 fis, clone HEMBB1002232	-
6015	4,36	4,66	-1,07	0,04894	0,445	228265_at	cDNA clone IMAGE:6214748	-
4346	3,31	3,54	-1,07	0,03237	0,407	1559732_at	Negative regulator of ubiquitin-like proteins 1	*NUB1*
4061	3,39	3,62	-1,07	0,02980	0,401	1569721_at	cDNA clone IMAGE:4838541	-
4986	4,63	4,95	-1,07	0,03827	0,419	238857_at	Arginyltransferase 1	*ATE1*
2897	3,55	3,80	-1,07	0,02034	0,383	1566505_at	cDNA FLJ34475 fis, clone HLUNG2003716	-
6118	3,14	3,36	-1,07	0,04996	0,446	220551_at	Solute carrier family 17, member 6	*SLC17A6*
4201	3,25	3,47	-1,07	0,03108	0,404	231127_at	Transcribed locus	-
5252	4,57	4,89	-1,07	0,04068	0,423	1557056_at	Hypothetical protein LOC133491	*LOC133491*
3761	3,00	3,21	-1,07	0,02734	0,397	1566481_at	cDNA DKFZp313L2229	-
1690	2,90	3,10	-1,07	0,01069	0,346	1560063_a_at	cDNA DKFZp667J114	-
2433	3,52	3,77	-1,07	0,01626	0,365	AFFX-LysX-M_a	-	-
2610	3,23	3,45	-1,07	0,01773	0,371	231308_at	Similar to Ankyrin repeat and FYVE domain containing 1 isoform 1	-
5031	3,54	3,79	-1,07	0,03867	0,420	1559069_at	mRNA full length insert cDNA clone EUROIMAGE 240968	-
2824	4,17	4,46	-1,07	0,01972	0,381	1568931_at	cDNA clone IMAGE:4799464	-
2217	2,88	3,08	-1,07	0,01469	0,362	232314_at	Mitochondrial fission regulator 1	*MTFR1*
2853	75,1	80,2	-1,07	0,02001	0,383	38447_at	Ankyrin repeat domain 13 family, member D	*ANKRD13D*
2776	3,05	3,26	-1,07	0,01928	0,380	215542_at	-	-
5019	5,75	6,14	-1,07	0,03861	0,420	1565918_a_at	mRNA from chromosome 5q21-22, clone:LI26	-
1236	5,71	6,10	-1,07	0,00735	0,324	1561392_at	cDNA clone IMAGE:3877950	-
5774	6,67	7,13	-1,07	0,04642	0,440	243248_at	Transcribed locus	-
5639	4,63	4,95	-1,07	0,04499	0,436	217512_at	-	-
5785	4,10	4,39	-1,07	0,04659	0,440	216160_at	cDNA FLJ14169 fis, clone NT2RP2002056	-
2851	3,26	3,49	-1,07	0,01997	0,383	224379_at	Ferritin, heavy polypeptide-like 17	*FTHL17*
3046	3,16	3,38	-1,07	0,02145	0,385	220860_at	Purine-rich element binding protein G	*PURG*
4128	3,77	4,03	-1,07	0,03038	0,402	215463_at	Olfactory receptor, family 7, subfamily E, member 24	*OR7E24*
5910	4,10	4,38	-1,07	0,04792	0,443	224128_at	Chromosome 20 open reading frame 43	*C20orf43*
5697	4,61	4,92	-1,07	0,04559	0,438	220461_at	Pecanex-like 2 (Drosophila)	*PCNXL2*
2766	4,20	4,49	-1,07	0,01921	0,379	1561407_at	Centaurin, delta 1	*CENTD1*
5413	2,98	3,18	-1,07	0,04253	0,430	1559651_at	Similar to Casein kinase I alpha	*LOC161635*
3808	6,33	6,76	-1,07	0,02774	0,398	1565612_at	Dynein, light chain, roadblock-type 1	*DYNLRB1*

Index	Mutated*	Normal*	FC	*P* value	FDR**	Probe set	Description	Gene symbol
5316	4,41	4,71	-1,07	0,04148	0,427	1552459_a_at	Methyl-CpG binding domain protein 3-like 1	*MBD3L1*
4826	3,12	3,33	-1,07	0,03674	0,416	241553_at	Germ cell associated 1	*GSG1*
5101	3,71	3,97	-1,07	0,03923	0,420	238239_at	WD repeat domain 27	*WDR27*
5533	3,38	3,61	-1,07	0,04393	0,434	231335_at	Membrane-spanning 4-domains, subfamily A, member 6E	*MS4A6E*
5272	4,00	4,27	-1,07	0,04096	0,425	214515_at	Olfactory receptor, family 1, subfamily E, member 1	*OR1E1*
3207	3,15	3,37	-1,07	0,02270	0,387	1569585_at	cDNA clone IMAGE:3959751	-
5306	2,80	2,99	-1,07	0,04140	0,426	240050_s_at	Transcribed locus	-
1590	3,11	3,32	-1,07	0,00988	0,340	242964_at	Transcribed locus	-
5837	4,06	4,34	-1,07	0,04717	0,442	1561732_at	Clone IMAGE:5547271, mRNA	-
6109	4,38	4,68	-1,07	0,04987	0,446	241254_at	Transcribed locus	-
1698	3,17	3,38	-1,07	0,01080	0,348	1562193_at	Clone IMAGE:5744109, mRNA	-
5219	3,83	4,09	-1,07	0,04036	0,423	223678_s_at	Surfactant, pulmonary-associated protein A1B	*SFTPA1B*
4308	4,63	4,95	-1,07	0,03208	0,407	207817_at	Interferon, omega 1	*IFNW1*
4487	3,71	3,96	-1,07	0,03375	0,411	224154_at	UDP-GlcNAc:betaGal beta-1,3-N-acetylglucosaminyltransferase 2	*B3GNT2*
5549	4,70	5,02	-1,07	0,04413	0,435	221401_at	Calcium channel, voltage-dependent, gamma subunit 5	*CACNG5*
4839	3,23	3,45	-1,07	0,03689	0,417	241651_at	Transcribed locus	-
5390	5,86	6,25	-1,07	0,04227	0,429	1556003_a_at	Transcribed locus	-
5596	4,47	4,77	-1,07	0,04462	0,436	1553818_x_at	POM121-like protein	-
4535	4,57	4,88	-1,07	0,03427	0,412	1555666_at	Protein tyrosine phosphatase, receptor type, S	*PTPRS*
5689	3,20	3,41	-1,07	0,04548	0,437	1556297_a_at	cDNA clone IMAGE:5295605	-
3275	2,94	3,13	-1,07	0,02331	0,388	1569929_at	-	-
5828	4,30	4,58	-1,07	0,04710	0,442	1557287_at	cDNA clone IMAGE:4837072	-
1581	2,44	2,60	-1,07	0,00984	0,340	1568925_at	MLCK protein	*MLCK*
4842	3,60	3,83	-1,07	0,03691	0,417	1565920_at	mRNA from chromosome 5q21-22, clone:LI26	-
5210	5,18	5,52	-1,07	0,04021	0,422	1558855_at	FERM, RhoGEF and pleckstrin domain protein 2	*FARP2*
4111	3,00	3,20	-1,07	0,03022	0,402	243652_at	Transcribed locus	-
2294	3,03	3,23	-1,07	0,01525	0,363	244225_x_at	-	-
5026	3,22	3,43	-1,07	0,03864	0,420	235262_at	Zinc finger protein 585B	*ZNF585B*
2014	3,43	3,66	-1,07	0,01314	0,355	241543_at	-	-
2913	3,40	3,63	-1,07	0,02046	0,383	206909_at	-	-
5829	3,69	3,93	-1,07	0,04711	0,442	1553861_at	T-complex 11 (mouse)-like 2	*TCP11L2*
5097	4,73	5,04	-1,07	0,03922	0,420	209847_at	Cadhetrin 17, LI Cadhetrin (liver-intestine)	*CDH17*
4864	2,97	3,17	-1,06	0,03710	0,417	1559229_at	cDNA clone IMAGE:3621839	-
5370	2,85	3,04	-1,06	0,04215	0,429	211119_at	Estrogen receptor 2 (ER beta)	*ESR2*
5538	23,7	25,2	-1,06	0,04401	0,434	234481_at	-	-
3695	2,92	3,11	-1,06	0,02673	0,395	1560940_at	cDNA FLJ32834 fis, clone TESTI2003236	-
870	3,09	3,29	-1,06	0,00502	0,313	1561445_at	cDNA clone IMAGE:4821940	-
4615	2,99	3,19	-1,06	0,03484	0,412	242291_at	Eukaryotic translation initiation factor 4E nuclear import factor 1	*EIF4ENIF1*
5702	4,06	4,32	-1,06	0,04565	0,438	1557451_at	DiGeorge syndrome critical region gene 10	*DGCR10*
4812	5,88	6,27	-1,06	0,03656	0,415	1562034_at	Chromosome 21 open reading frame 134	*C21orf134*
3790	3,32	3,54	-1,06	0,02760	0,398	210436_at	Chaperonin containing TCP1, subunit 8 (theta)	*CCT8*
5969	3,46	3,68	-1,06	0,04848	0,444	1562424_at	Hypothetical protein LOC285889	*LOC285889*
5581	2,95	3,14	-1,06	0,04450	0,436	234679_at	Keratin associated protein 9-3	*KRTAP9-3*
5982	3,76	4,00	-1,06	0,04856	0,444	1555476_at	Iron-responsive element binding protein 2	*IREB2*
3010	2,73	2,90	-1,06	0,02118	0,385	1560404_a_at	ATP binding domain 4	*ATPBD4*
3418	2,88	3,07	-1,06	0,02442	0,391	1564392_at	Chromosome 21 open reading frame 131	*C21orf131*
4384	3,07	3,27	-1,06	0,03265	0,407	236888_at	Capping protein (actin filament) muscle Z-line, alpha 3	*CAPZA3*
5069	3,14	3,34	-1,06	0,03901	0,420	1564235_at	HCG1994895 protein	-
5547	3,17	3,37	-1,06	0,04410	0,435	215448_at	Clone 23638 mRNA sequence	-
1139	3,55	3,78	-1,06	0,00666	0,320	234309_at	Testis-specific transcript, Y-linked 7	*TTTY7*
5625	4,18	4,45	-1,06	0,04492	0,436	1564590_a_at	Orofacial cleft 1 candidate 1	*OFCC1*
5545	3,66	3,89	-1,06	0,04408	0,435	220978_at	Keratin associated protein 1-3	*KRTAP1-3*
5811	2,77	2,95	-1,06	0,04694	0,442	1561703_at	cDNA clone IMAGE:5269594	-
2260	2,79	2,97	-1,06	0,01505	0,363	240952_at	Transcribed locus	-
3698	4,30	4,57	-1,06	0,02674	0,395	234427_at	T cell receptor alpha locus	*TRA@*
4671	2,89	3,07	-1,06	0,03526	0,413	215704_at	Filaggrin	*FLG*
5375	3,90	4,14	-1,06	0,04217	0,429	1562597_at	Clone IMAGE:5742729, mRNA	-

Index	Mutated*	Normal*	FC	*P* value	FDR**	Probe set	Description	Gene symbol
4350	3,37	3,58	-1,06	0,03239	0,407	1557672_s_at	cDNA clone IMAGE:5297153	-
5438	4,21	4,47	-1,06	0,04287	0,431	1552414_at	WAP four-disulfide core domain 9	*WFDC9*
2489	2,73	2,90	-1,06	0,01678	0,367	229478_x_at	Basic, immunoglobulin-like variable motif containing	*BIVM*
1989	3,09	3,28	-1,06	0,01301	0,355	210521_s_at	Fetuin B	*FETUB*
5160	2,75	2,92	-1,06	0,03973	0,421	216672_s_at	Myelin transcription factor 1-like	*MYT1L*
5098	3,48	3,70	-1,06	0,03922	0,420	239950_at	Homeo box A11, antisense	*HOXA11S*
2966	3,11	3,30	-1,06	0,02084	0,384	233312_at	Ropporin 1-like	*ROPN1L*
3696	2,73	2,90	-1,06	0,02674	0,395	217452_s_at	UDP-Gal:betaGlcNAc beta 1,3-galactosyltransferase, polypeptide 2	*B3GALT2*
4329	4,80	5,09	-1,06	0,03221	0,407	234416_at	Immunoglobulin lambda variable 2-14	*IGLV2-14*
1880	2,80	2,98	-1,06	0,01219	0,355	243725_at	Transcribed locus	-
1510	2,57	2,73	-1,06	0,00931	0,337	241291_at	cDNA FLJ36657 fis, clone UTERU2001876	-
6032	7,59	8,05	-1,06	0,04910	0,445	231141_at	cDNA clone IMAGE:4838568	-
4608	4,15	4,40	-1,06	0,03481	0,412	241128_at	-	-
5605	3,54	3,76	-1,06	0,04471	0,436	231529_at	Maternally expressed 3	*MEG3*
4263	3,18	3,37	-1,06	0,03171	0,406	1567612_at	Transcript ch132	-
4091	4,22	4,47	-1,06	0,03008	0,402	241056_at	Zinc finger CCCH-type containing 3	*ZC3H3*
3145	2,75	2,91	-1,06	0,02231	0,387	240474_x_at	Transcribed locus	-
3598	2,62	2,78	-1,06	0,02595	0,394	1557206_at	Hypothetical protein FLJ35848	*FLJ35848*
3001	2,77	2,94	-1,06	0,02113	0,385	1568898_at	Clone IMAGE:3921535, mRNA	-
4486	2,70	2,86	-1,06	0,03375	0,411	233295_at	Pheromone receptor (PHRET) pseudogene	-
5551	3,70	3,92	-1,06	0,04414	0,435	220527_at	Mitochondrial ribosomal protein L20	*MRPL20*
3901	2,88	3,05	-1,06	0,02851	0,399	1562640_at	Hypothetical protein LOC285943	*LOC285943*
4351	2,80	2,96	-1,06	0,03240	0,407	231665_at	Arginase, liver	*ARG1*
4126	3,14	3,32	-1,06	0,03036	0,402	1558813_at	Transmembrane emp24 protein transport domain containing 5	*TMED5*
4097	2,69	2,85	-1,06	0,03011	0,402	1556877_at	cDNA clone IMAGE:4799788	-
3218	2,73	2,89	-1,06	0,02280	0,387	237996_at	-	-
4788	2,75	2,90	-1,06	0,03631	0,415	1562313_at	BCL6 co-repressor-like 2	*BCORL2*
2839	2,78	2,94	-1,06	0,01981	0,382	1568711_a_at	cDNA clone IMAGE:4829846	-
5752	3,55	3,75	-1,06	0,04616	0,439	1555369_at	B melanoma antigen	*BAGE*
4460	2,75	2,91	-1,06	0,03351	0,411	241569_at	Transcribed locus	-
3192	3,30	3,48	-1,06	0,02262	0,387	234616_at	cDNA FLJ21341 fis, clone COL02653	-
4719	3,16	3,33	-1,05	0,03570	0,413	1558645_at	Mindbomb homolog 1 (Drosophila)	*MIB1*
4498	3,04	3,21	-1,05	0,03384	0,411	1553022_at	Zinc finger, imprinted 3	*ZIM3*
5405	2,98	3,14	-1,05	0,04243	0,429	1562418_at	Clone IMAGE:5227164, mRNA	-
5043	2,75	2,89	-1,05	0,03886	0,420	234209_at	cDNA FLJ21495 fis, clone COL05614	-
2952	2,46	2,59	-1,05	0,02072	0,384	1569591_at	Coagulation factor XI (plasma thromboplastin antecedent)	*F11*
5845	2,96	3,11	-1,05	0,04722	0,442	1570517_at	Four and a half LIM domains 5	*FHL5*
5434	3,16	3,31	-1,05	0,04281	0,431	1558846_at	Pancreatic lipase-related protein 3	*PNLIPRP3*
5344	2,73	2,86	-1,05	0,04189	0,429	236735_at	-	-
1658	2,58	2,71	-1,05	0,01047	0,345	241557_x_at	Transcribed locus	-
3954	2,80	2,93	-1,05	0,02889	0,399	1563680_at	Hypothetical protein LOC284950	*LOC284950*
5195	2,78	2,91	-1,05	0,04006	0,421	215944_at	-	-
4568	2,59	2,72	-1,05	0,03455	0,412	243281_at	Transcribed locus	-
2214	2,41	2,53	-1,05	0,01467	0,362	241961_at	Steroid 5 alpha-reductase 2-like 2	*SRD5A2L2*
4044	3,16	3,31	-1,05	0,02965	0,401	232817_at	cDNA FLJ13415 fis, clone PLACE1001799	-
5095	2,98	3,12	-1,05	0,03920	0,420	207674_at	Fc fragment of IgA, receptor for	*FCAR*
3914	2,62	2,74	-1,05	0,02858	0,399	1557717_at	Hypothetical protein LOC338862	*LOC338862*
4045	2,65	2,77	-1,05	0,02965	0,401	1561437_at	Hypothetical gene LOC728012	*LOC728012*
5255	2,94	3,07	-1,04	0,04073	0,424	1560989_at	Full length insert cDNA clone YY93H10	-
5771	2,87	2,99	-1,04	0,04637	0,439	1570194_x_at	Clone IMAGE:4480721, mRNA	-
4741	3,30	3,43	-1,04	0,03591	0,414	237938_at	Transcribed locus	-
5256	2,58	2,68	-1,04	0,04075	0,424	1561959_x_at	Full length insert cDNA clone YR17E12	-

*** Geometric mean of intensities**

**** False Discovery Rate**
