# Supplementary material for: Gene Expression Profiling Reveals New Aspects of PIK3CA Mutation in ERalpha-Positive Breast Cancer: Major Implication of the Wnt Signaling Pathway
Source: PLoS One. 2010 Dec 30;5(12):e15647. doi: 10.1371/journal.pone.0015647 (PMC3012715; doi:10.1371/journal.pone.0015647)
Supplement: Table S4 — List of 56 probes (39 unique genes) deregulated in REα-positive breast tumors with PIK3CA mutations compared to those without PIK3CA mutation identified by PAM. These genes are presented according to the rank in PAM output. For each gene, we report the fold change (FC) calculated between expression intensities of tumors with and without PIK3CA mutations using BRB Arrays Tools. The genes with a FC≥3 are put in bold. (PDF) [file pone.0015647.s004.pdf]

Rank in PAM	Symbol Gene	Name Gene	Probe set	FC	GenBank
**1**	***WNT5A***	**Wingless-type mouse mammary tumor virus integration site family, member 5A**	**231227_at**	**3.43**	**NM_003392**
2	*PPL*	Periplakin	203407_at	2.42	NM_002745
3	*PIK3R1*	Phosphoinositide-3-kinase, regulatory subunit 1 (alpha)	212240_s_at	2.45	NM_181523
4	*ID4*	Inhibitor of DNA binding 4, dominant negative helix-loop-helix protein	209291_at	2.96	NM_001546
**5**	***LTF***	**Lactotransferrin**	**202018_at**	**10.52**	**NM_002343**
6	*TCF7L2*	Transcription factor 7-like 2 (T-cell specific, HMG-box)	216551_s_at	2.16	NM_030756
**7**	***HMGCS2***	**3-hydroxy-3-methylglutanyl-Coenzyme A synthase 2 (mitochondrial)**	**204607_at**	**5.31**	**NM_005518**
8	*TCF7L2*	Transcription factor 7-like 2 (T-cell specific, HMG-box)	216035_x_at	2.08	NM_030756
9	*LIMCH1*	LIM and calponin homology domains 1	212328_at	2.69	NM_014988
**10**	***NRIP3***	**Nuclear receptor interacting protein 3**	**219557_s_at**	**3.28**	**NM_020645**
11	*WNT5A*	Wingless-type mouse mammary tumor virus integration site family, member 5A	205990_s_at	2.84	NM_003392
12	*ATP1B1*	ATPase, Na+/K+ transporting, beta 1 polypeptide	201242_s_at	2.76	NM_001677
13	*TCF7L2*	Transcription factor 7-like 2 (T-cell specific, HMG-box)	212762_s_at	2.18	NM_030756
**14**	***ID4***	**Inhibitor of DNA binding 4, dominant negative helix-loop-helix protein**	**209292_at**	**3.03**	**NM_001546**
**15**	***SLC4A4***	**Solute carrier family 4, sodium bicarbonate cotransporter, member 4**	**203908_at**	**4.81**	**NM_003759**
16	*LIMCH1*	LIM and calponin homology domains 1	212325_at	2.96	NM_014988
**17**	***SLC40A1***	**Solute carrier family 40 (iron-regulated transporter), member 1**	**239723_at**	**4.42**	**NM_014585**
**18**	***VTCN1***	**V-set domain containin T cell activity inhibitor 1**	**219768_at**	**5.47**	**NM_024626**
19	*PMEPA1*	Prostate transmembrane protein, androgen induced 1	222450_at	2.21	NM_020182
20	*ATP1B1*	ATPase, Na+/K+ transporting, beta 1 polypeptide	201243_s_at	2.71	NM_001677
21	*WNT5A*	Wingless-type mouse mammary tumor virus integration site family, member 5A	213425_at	2.81	NM_003392
22	*ZFP36L1*	Zinc finger protein 36, C3H type-like 1	211962_s_at	1.75	NM_004926
23	*SERPINB5*	Serpin peptidase inhibitor, clade B (ovalbumin), member 5	204855_at	2.99	NM_002639
**24**	***SEC14L2***	**SEC14-like 2**	**204541_at**	**3.03**	**NM_012429**
**25**	***REEP1***	**Receptor accessory protein 1**	**204364_s_at**	**3.30**	**NM_022912**
**26**	***NTN4***	**Netrin 4**	**223315_at**	**4.21**	**NM_021229**
**27**	***NKAIN1***	**Na+/K+ transporting ATPase interacting 1**	**219438_at**	**-3.52**	**NM_024522**
28	*MAPT*	Microtubule-associated protein tau	206401_s_at	2.82	NM_016835
29	*PIK3R1*	Phosphoinositide-3-kinase, regulatory subunit 1 (alpha)	212249_at	2.34	NM_181523
30	*TCF7L2*	Transcription factor 7-like 2 (T-cell specific, HMG-box)	216037_x_at	1.86	NM_030756
**31**	***SEC14L2***	**SEC14-like 2**	**230316_at**	**3.05**	**NM_012429**
**32**	***CYP4Z2P***	**Cytochrome P450, family 4, subfamily Z, polypeptide 2 pseudogene**	**1553434_at**	**3.80**	**NR_002788**
33	*LIMCH1*	LIM and calponin homology domains 1	241459_at	2.35	NM_014988
**34**	***ANPEP***	**Alanyl (membrane) aminopeptidase**	**202888_s_at**	**3.52**	**NM_001150**
35	*NEDD9*	Neural precursor cell expressed, developmentally down-regulated 9	202149_at	2.48	NM_006403
36	*ZFP36L1*	Zinc finger protein 36, C3H type-like 1	211965_at	2.18	NM_004926

37	*TCF7L2*	Transcription factor 7-like 2 (T-cell specific, HMG-box)	212761_at	1.92	NM_030756
**38**	***CYP4B1***	**Cytochrome P450, family 4, subfamily B, polypeptide 1**	**210096_at**	**4.12**	**NM_000779**
39	*NME7*	Non-metastatic cells 7, protein expressed in (nucleoside-diphosphate kinase)	227556_at	2.99	NM_013330
40	*NUCKS1*	Nuclear casein kinase and cyclin-dependent kinase substrate 1	222027_at	2.03	NM_022731
41	*PNMA2*	Paraneoplastic antigen MA2	209598_at	2.13	NM_007257
**42**	***TFAP2B***	**Transcription factor AP-2 beta (activating enhancer binding protein 2 beta)**	**214451_at**	**6.68**	**NM_003221**
43	*ENC1*	Ectodermal-neural cortex (with BTB-like domain)	201340_s_at	2.01	NM_003633
44	*FAM46B*	Family with sequence similarity 46, member B	229518_at	2.36	NM_052943
45	*GNPAT*	Glyceronephosphate O-acyltransferase	229994_at	2.23	NM_014236
46	*ALOX5*	Arachidonate 5-lipoxygenase	204446_s_at	2.19	NM_000698
47	*TCN1*	Transcobalamin I (vitamin B12 binding protein, R binder family)	205513_at	2.87	NM_001062
48	*MAP3K1*	Mitogen-activated protein kinase kinase kinase 1	243030_at	2.49	NM_005921
49	*PMEPA1*	Prostate transmembrane protein, androgen induced 1	222449_at	2.02	NM_020182
50	*AK5*	Adenylate kinase 5	222862_s_at	2.93	NM_012093
51	*NFIA*	Nuclear factor I/A	224975_at	2.13	NM_005595
52	*POSTN*	Periostin, osteoblast specific factor	228481_at	2.72	NM_006475
53	*PIK3R1*	Phosphoinositide-3-kinase, regulatory subunit 1 (alpha)	239476_at	1.88	NM_181523
54	*LIMCH1*	LIM and calponin homology domains 1	212327_at	2.49	NM_014988
**55**	***TMC5***	**Transmembrane channel-like 5**	**240304_s_at**	**4.27**	**NM_024780**
56	*FAM129A*	Family with sequence similarly 129, member A	217966_s_at	2.96	NM_052966
