# Supplementary material for: Gene Expression Profiling Reveals New Aspects of PIK3CA Mutation in ERalpha-Positive Breast Cancer: Major Implication of the Wnt Signaling Pathway
Source: PLoS One. 2010 Dec 30;5(12):e15647. doi: 10.1371/journal.pone.0015647 (PMC3012715; doi:10.1371/journal.pone.0015647)
Supplement: Table S5 — Relative mRNA expression levels of the 29 genes in the 44 ERα-positive breast tumors with exon 9 PIK3CA mutations compared to the 47 tumors with exon 20 PIK3CA mutations. The tumor with PIK3CA mutations in both exon 9 and exon 20 was excluded from the analysis. For each gene, we report the median (range) of the mRNA levels of each gene relative to normal breast tissue samples, the fold change (FC) between tumors with exon 9-mutated and exon 20-mutated PIK3CA and the P value associated to Mann-Whitney U test. (PDF) [file pone.0015647.s005.pdf]

Symbol Gene	GenBank	Exon 9-mutated *PIK3CA* (n=44)	Exon 20-mutated *PIK3CA* (n=47)	FC	*P* value
**UP-REGULATED GENES**					
ANPEP	NM_001150	0.42 (0.06-14.5)	0.33 (0.07-18.3)	1.29	ns
CYP4B1	NM_000779	8.5 (0.00-178)	4.38 (0.35-82.3)	1.94	ns
CYP4X1	NM_178033	3.56 (0.07-101)	3.99 (0.05-51.5)	-1.12	ns
CYP4Z1	NM_171834	3.00 (0.01-254)	3.01 (0.04-120)	1.00	ns
CYP4Z2P	NR_002788	69.3 (1.43-1069)	66.7 (0.00-486)	1.04	ns
HMGCS2	NM_005518	0.47 (0.00-25.7)	0.76 (0.00-22.6)	-1.62	ns
ID4	NM_001546	0.15 (0.03-2.43)	0.21 (0.02-9.57)	-1.38	ns
LIMCH1	NM_014988	1.09 (0.16-8.58)	1.14 (0.08-5.74)	-1.05	ns
LTF	NM_002343	0.15 (0.01-41.8)	0.13 (0.00-13.6)	1.17	ns
MAPT	NM_016835	4.44 (0.86-15.9)	4.54 (0.15-26.2)	-1.02	ns
MSX2	NM_002449	3.48 (0.32-15.2)	4.07 (0.11-39.3)	-1.17	ns
NR2F2	NM_021005	1.24 (0.17-7.27)	0.92 (0.11-4.40)	1.34	ns
NRIP3	NM_020645	2.91 (0.10-79.5)	1.77 (0.22-105)	1.65	0.0378
NTN4	NM_021229	1.14 (0.04-10.2)	1.29 (0.14-8.77)	-1.14	ns
PIK3R1	NM_181523	0.36 (0.12-0.98)	0.37 (0.08-1.30)	-1.02	ns
REEP1	NM_022912	2.69 (0.20-16.8)	2.57 (0.19-21.8)	1.05	ns
SEC14L2	NM_012429	4.97 (0.48-39.1)	3.69 (0.16-36.9)	1.35	ns
SLC4A4	NM_003759	0.51 (0.00-79.7)	0.39 (0.01-128)	1.29	ns
SLC40A1	NM_014585	1.21 (0.08-10.6)	1.22 (0.00-17.9)	-1.01	ns
TCF7L2	NM_030756	0.30 (0.13-1.22)	0.35 (0.09-1.26)	-1.17	ns
TFAP2B	NM_003221	3.33 (0.00-179)	8.38 (0.00-117)	-2.51	0.0334
TMC5	NM_024780	6.24 (0.11-46.2)	5.42 (0.63-29.3)	1.15	ns
TNFRSF11B	NM_002546	1.93 (0.15-21.8)	1.76 (0.22-12.4)	1.10	ns
VANGL2	NM_020335	0.99 (0.14-9.09)	0.64 (0.07-3.47)	1.54	ns
VTCN1	NM_024626	0.65 (0.01-15.4)	0.64 (0.01-15.1)	1.01	ns
WNT5A	NM_003392	1.15 (0.35-4.77)	1.19 (0.24-7.27)	-1.04	ns
**DOWN-REGULATED GENES**					
NKAIN1	NM_024522	67.7 (0.74-560)	45.4 (0.71-560)	1.49	ns
TPD52	NM_005079	5.40 (2.00-80.9)	5.33 (1.75-34.9)	1.01	ns
TUSC3	NM_006765	0.65 (0.08-6.31)	0.62 (0.13-2.69)	1.04	ns

ns: not significant
