# Supplementary material for: Gene Expression Profiling Reveals New Aspects of PIK3CA Mutation in ERalpha-Positive Breast Cancer: Major Implication of the Wnt Signaling Pathway
Source: PLoS One. 2010 Dec 30;5(12):e15647. doi: 10.1371/journal.pone.0015647 (PMC3012715; doi:10.1371/journal.pone.0015647)
Supplement: Table S6 — Supervised hierarchical clustering analysis of the 249 ERα-positive breast tumors. Classification performance of discriminating gene sets identified from the 19 significantly deregulated genes in tumors with PIK3CA mutations (18 up-regulated genes + NKAIN1). Each gene set separates the 157 tumors without PIK3CA mutation (N) and the 92 tumors with PIK3CA mutations (M) in two main clusters (cluster 1 and 2). (PDF) [file pone.0015647.s006.pdf]

Discriminating gene set	Cluster 1	Cluster 2	Accuracy	MCC[a]	*P* value[b]
18 up-regulated genes	85N+35M	72N+57M	57.0%	0.1555	0.0141
18 up-regulated genes + *NKAIN1*	83N+28M	74N+64M	59.4%	0.2178	0.0006
NKAIN1+CYP4Z2P+WNT5A+TMC5	83N+27M	74N+65M	59.4%	0.2286	0.0003
NKAIN1+CYP4Z2P+WNT5A+MAPT+MSX2+TFAP2B	83N+27M	74N+65M	59.4%	0.2286	0.0003
NKAIN1+CYP4Z2P+NRIP3+SEC14L2+TFAP2B	82N+26M	75N+66M	59.4%	0.2334	0.0002

[a] Matthews' correlation coefficient.
[b] Chi-square test.
